# Estatística Cardiovascular – Brasil 2023

**DOI:** 10.36660/abc.20240079

**Published:** 2024-02-15

**Authors:** Gláucia Maria Moraes de Oliveira, Luisa Campos Caldeira Brant, Carisi Anne Polanczyk, Deborah Carvalho Malta, Andreia Biolo, Bruno Ramos Nascimento, Maria de Fatima Marinho de Souza, Andrea Rocha De Lorenzo, Antonio Aurélio de Paiva Fagundes, Beatriz D. Schaan, Christina Grüne de Souza e Silva, Fábio Morato de Castilho, Fernando Henpin Yue Cesena, Gabriel Porto Soares, Gesner Francisco Xavier, Jose Augusto Soares Barreto, Luiz Guilherme Passaglia, Marcelo Martins Pinto, M. Julia Machline-Carrion, Marcio Sommer Bittencourt, Octavio M. Pontes, Paolo Blanco Villela, Renato Azeredo Teixeira, Ricardo Stein, Roney Orismar Sampaio, Thomaz A. Gaziano, Pablo Perel, Gregory A. Roth, Antonio Luiz Pinho Ribeiro

**Affiliations:** 1 Instituto do Coração Edson Saad Universidade Federal do Rio de Janeiro Rio de Janeiro RJ Brasil Instituto do Coração Edson Saad da Universidade Federal do Rio de Janeiro (UFRJ), Rio de Janeiro, RJ – Brasil; 2 Universidade Federal do Rio de Janeiro Rio de Janeiro RJ Brasil Universidade Federal do Rio de Janeiro (UFRJ), Rio de Janeiro, RJ – Brasil; 3 Universidade Federal de Minas Gerais Belo Horizonte MG Brasil Universidade Federal de Minas Gerais (UFMG), Belo Horizonte, MG – Brasil; 4 Hospital das Clínicas Universidade Federal de Minas Gerais Belo Horizonte MG Brasil Hospital das Clínicas da Universidade Federal de Minas Gerais (UFMG), Belo Horizonte, MG – Brasil; 5 Universidade Federal do Rio Grande do Sul Porto Alegre RS Brasil Universidade Federal do Rio Grande do Sul (UFRGS), Porto Alegre, RS – Brasil; 6 Hospital Moinhos de Vento Porto Alegre RS Brasil HHospital Moinhos de Vento, Porto Alegre, RS – Brasil; 7 Hospital de Clínicas de Porto Alegre Porto Alegre RS Brasil Hospital de Clínicas de Porto Alegre (HCPA), Porto Alegre, RS – Brasil; 8 Hospital Madre Teresa Belo Horizonte MG Brasil Hospital Madre Teresa, Belo Horizonte, MG – Brasil; 9 Vital Strategies New York EUA Vital Strategies,New York – EUA; 10 Instituto Nacional de Cardiologia Rio de Janeiro RJ Brasil Instituto Nacional de Cardiologia, Rio de Janeiro, RJ – Brasil; 11 Instituto D’Or de Pesquisa e Ensino Brasília Brasil Instituto D’Or de Pesquisa e Ensino (IDOR), Brasília – Brasil; 12 Universidade de Brasília Brasília Brasil Universidade de Brasília (UNB), Brasília – Brasil; 13 Hospital DFStar Rede DO´r Brasília Brasil Hospital DFStar, Rede DO´r, Brasília – Brasil; 14 Instituto Dante Pazzanese de Cardiologia São Paulo SP Brasil Instituto Dante Pazzanese de Cardiologia, São Paulo, SP – Brasil; 15 Curso de Medicina Universidade de Vassouras Vassouras RJ Brasil Curso de Medicina da Universidade de Vassouras, Vassouras, RJ – Brasil; 16 Universidade Federal de Sergipe Aracaju SE Brasil Universidade Federal de Sergipe, Aracaju, SE – Brasil; 17 Hospital São Lucas Rede São Luiz D’Or Aracaju SE Brasil Hospital São Lucas Rede São Luiz D’Or, Aracaju, SE – Brasil; 18 epHealth UK Londres Reino Unido epHealth UK, Londres – Reino Unido; 19 Instituto epHealth São Paulo SP Brasil Instituto epHealth, São Paulo, SP – Brasil; 20 Department of Medicine and Radiology University of Pittsburgh Pittsburgh EUA Department of Medicine and Radiology University of Pittsburgh, Pittsburgh – EUA; 21 Faculdade de Medicina de Ribeirão Preto Universidade de São Paulo São Paulo SP Brasil Faculdade de Medicina de Ribeirão Preto da Universidade de São Paulo (USP), São Paulo, SP – Brasil; 22 Faculdade de Medicina Universidade de São Paulo São Paulo SP Brasil Faculdade de Medicina da Universidade de São Paulo (USP), São Paulo, SP – Brasil; 23 Instituto do Coração Hospital das Clínicas da Faculdade de Medicina Universidade de São Paulo São Paulo SP Brasil Instituto do Coração (Incor) do Hospital das Clínicas da Faculdade de Medicina da Universidade de São Paulo (HCFMUSP), São Paulo, SP – Brasil; 24 Division of Cardiovascular Medicine Brigham and Women’s Hospital Boston EUA Division of Cardiovascular Medicine, Brigham and Women’s Hospital, Boston – EUA; 25 Department of Health Policy and Management Harvard T.H. Chan School of Public Health Boston EUA Department of Health Policy and Management, Harvard T.H. Chan School of Public Health, Boston – EUA; 26 World Heart Federation Geneva Suíça World Heart Federation, Geneva – Suíça; 27 Centre for Global Chronic Conditions London School of Hygiene & Tropical Medicine Londres Inglaterra Centre for Global Chronic Conditions, London School of Hygiene & Tropical Medicine, Londres – Inglaterra; 28 Division of Cardiology Department of Medicine University of Washington Washington EUA Division of Cardiology, Department of Medicine, University of Washington, Washington – EUA

**Keywords:** Doenças Cardiovasculares, Trastornos Cerebrovasculares, Doença das Coronárias, Cardiomiopatias, Doenças das Valvas Cardíacas, Insuficiência Cardíaca, Fibrilação Atrial, Flutter Atrial, Hipertensão, Dislipidemias, Diabetes Mellitus, Tabagismo, Obesidade, Sobrepeso, Exercício Físico, Estatística, Brasil

## SOBRE ESTE DOCUMENTO

**Table:** Abreviaturas Usadas nesta Introdução

AVC	Acidente Vascular Cerebral
CID	Classificação Estatística Internacional de Doenças e Problemas Relacionados à Saúde
CINAHL	Cumulative Index to Nursing and Allied Health Literature
COVID-19	Doença do novo coronavírus 2019
CRVM	Cirurgia de Revascularização do Miocárdio
CV	Cardiovascular
DAC	Doença Arterial Coronariana
DALY	Anos de vida perdidos ajustados por incapacidade (do inglês, Disability-Adjusted Life-Year)
DATASUS	Base de dados do Departamento de Informática do Sistema Único de Saúde
DCh	Doença de Chagas
DCR:	Doença Cardíaca Reumática
DCV:	Doença Cardiovascular
DeCS	Descritores em Ciências da Saúde
ECG	Eletrocardiograma
ELSA-Brasil	Estudo Longitudinal de Saúde do Adulto
FA	Fibrilação Atrial
GBD	Global Burden of Disease
IBGE	Instituto Brasileiro de Geografia e Estatística
IC	Intervalo de Confiança
ICP	Intervenção Coronariana Percutânea
IHME	Institute for Health Metrics and Evaluation
II	Intervalo de Incerteza
Int$	dólares internacionais
IPCA	Índice de Preços ao Consumidor Amplo
LILACS	Literatura Latino-Americana e do Caribe em Ciências da Saúde
MEDLINE	Medical Literature Analysis and Retrievel System Online
MeSH	Medical Subject Headings
NYHA	New York Heart Association
PIB	Produto Interno Bruto
PNS	Pesquisa Nacional de Saúde
PPC	Paridade do Poder de Compra R$ Reais
RECALL	Registro Brasileiro de Fibrilação Atrial Crônica
SCA	Síndrome Coronariana Aguda
SCC	Síndrome Coronariana Crônica
SIH	Sistema de Informações Hospitalares
SIM	Sistema de Informações sobre Mortalidade
SUS	Sistema Único de Saúde
TTR	Tempo na Faixa Terapêutica (do inglês, Time in Therapeutic Range)

A publicação ***Estatística Cardiovascular – Brasil*** tem por objetivo fornecer uma compilação anual dos dados e das pesquisas sobre a epidemiologia das DCV no Brasil. Este documento congrega as estatísticas oficiais do Ministério da Saúde do Brasil e outras entidades governamentais ao lado de dados do projeto GBD, coordenado pelo IHME da Universidade de Washington. Além disso, incorpora dados derivados de várias fontes e estudos científicos, inclusive coortes e registros, relacionados às DCV e fatores de risco associados. Esta publicação destina-se a um público variado, incluindo pesquisadores, clínicos, pacientes, formuladores de políticas de saúde, profissionais da mídia, o público em geral e todos aqueles que buscam dados nacionais abrangentes sobre DCV e acidente vascular cerebral. Pesquisadores voluntários de várias universidades e instituições de pesquisa brasileiros realizaram este projeto. O grupo é liderado por um comitê diretivo com cinco membros (ALPR, CAP, DCM, GMMO e LCCB). A Sociedade Brasileira de Cardiologia apoia integralmente esta iniciativa e o projeto recebe colaboração da *Rede GBD Brasil* ^1^ e de um comitê internacional (GAR, PP e TAG) com membros do IHME/Universidade de Washington (GAR) e da *World Heart Federation* (PP e TAG).

A primeira ***Estatística Cardiovascular – Brasil*** foi lançada em 2020^2^ e incluiu capítulos sobre DCV total e cinco condições específicas: doença cerebrovascular, doença arterial coronariana, cardiomiopatia e insuficiência cardíaca e fibrilação atrial. A publicação segue a metodologia usada pela *American Heart Association* na produção anual da ***Heart Disease & Stroke Statistics Update*** ,^3^ que também enfatiza dados epidemiológicos e de saúde pública. A ***Estatística Cardiovascular – Brasil 2021*** ^4^ incluiu os dados mais recentes sobre aquelas doenças e novos capítulos sobre comportamentos e fatores de risco CV, especificamente hipertensão, diabetes, dislipidemia, obesidade e tabagismo e uso de tabaco. Um site de apoio fornece gráficos e mapas baseados nos dados disponíveis. Essas duas primeiras versões da ***Estatística Cardiovascular – Brasil*** ^2 , 4^ logo se tornaram uma referência na área e uma fonte natural de informação para as partes interessadas, profissionais da saúde e pesquisadores, tendo sido acessadas mais de 26 mil vezes (somente através da página da ABC Cardiol) até novembro de 2023.

O advento da pandemia de COVID-19, que assolou o Brasil a partir de fevereiro de 2020 e ‘roubou a cena’ nos dois anos seguintes, mudou nossas vidas e práticas profundamente. O número de mortes no Brasil chegou a inacreditáveis 700 mil e quase 37 milhões de pessoas foram infectadas.^5^ Os recursos de saúde e capacidade de pesquisa no Brasil e no mundo foram massivamente direcionados para atender pacientes de COVID-19 e melhor compreender a doença, assim como preveni-la e tratá-la. Desde o início, ficou clara a existência de uma interação entre COVID-19 e DCV, devido não apenas à concorrência por recursos de saúde e políticas para mitigação, que, embora necessárias, afetaram indiretamente a assistência CV, mas também à possível interação entre o vírus e o sistema CV, sua prevenção através de vacinas e alguns supostos tratamentos.^6^ Entretanto, não podíamos prever que, como detalhado nesta versão da ***Estatística Cardiovascular - Brasil*** , a COVID-19 se tornaria a mais importante causa de morte no Brasil em 2021, superando a síndrome coronariana aguda e o acidente vascular cerebral. Neste documento, cada capítulo traz os dados brasileiros sobre a interação da COVID-19 com as DCV e seus fatores de risco, ressaltando a importância da comunidade brasileira de pesquisa na área.

Além de todos esses aspectos largamente discutidos nas publicações científicas, conferências e mídia em geral, a pandemia de COVID-19 afetou o tempo e a maneira como os dados de saúde foram coletados e relatados, causando atrasos e incertezas na estatística de mortalidade e carga de doença em geral. A coexistência e a maior letalidade da COVID-19 em pacientes com doenças crônicas, como as DCV, assim como a maior frequência de mortes fora do hospital, reduziram a acurácia da informação sobre a causa de morte, tornando a interpretação dos achados complexa e desafiadora.^8 , 13^ Estudos epidemiológicos foram suspensos ou adiados e a divulgação de conjuntos de dados definitivos pelas autoridades de saúde foi postergada devido ao trabalho extenuante de fornecer informação rápida sobre a pandemia e o processo de vacinação. O IHME, que produz uma das principais fontes da ***Estatística Cardiovascular – Brasil*** , cancelou a divulgação do GBD 2020, cujo lançamento era esperado para meados de 2022, e dedicou-se a estimar e prever dados relativos à pandemia.^16 , 17^ Até o momento, não foi disponibilizada ao público uma atualização da última versão do GBD 2019, estando a publicação do GBD 2021 estimada para ocorrer no início de 2024.

A ***Estatística Cardiovascular – Brasil*** usa dados de quatro diferentes fontes: (a) sistemas brasileiros de informação de mortalidade e saúde, disponibilizados pelo governo; (b) as mais recentes estimativas do GBD; (c) revisão sistemática da literatura com ênfase nas publicações dos últimos dez anos; (d) custos do uso dos serviços de saúde, com base nas tabelas de reembolso do Sistema Público de Saúde. A ausência de atualização das estimativas do GBD impactou profundamente nosso trabalho: tivemos que cancelar a divulgação da ***Estatística Cardiovascular – Brasil 2022*** . Decidimos publicar a ***Estatística Cardiovascular – Brasil 2023*** com os dados disponíveis do estudo GBD e outras fontes, acrescentando (a) um novo capítulo sobre atividade física como hábito de saúde relacionado à DCV, (b) seções sobre COVID-19 nas DCV específicas e seus fatores de risco, e (c) uma análise de custo mais detalhada. A ***Estatística Cardiovascular – Brasil 2024*** , a ser divulgada até o final de 2024, trará o conjunto completo de dados do GBD 2021 que será publicado no primeiro semestre de 2024.

Além disso, esta ***Estatística Cardiovascular – Brasil 2023*** enfatiza que a DCV ainda responde por quase um terço das mortes no Brasil, afetando desproporcionalmente a camada mais pobre da população, que tem dificuldades de acesso a cuidados de saúde de alta qualidade.^18 , 19^ A COVID-19 expôs essas desigualdades, sendo o excesso de mortalidade maior na população negra/parda^20^ e o excesso de mortalidade CV maior nas cidades menos desenvolvidas, possivelmente associados com o colapso da assistência à saúde.^8^ A existência de dados nacionais representativos, confiáveis e abrangentes sobre DCV, comportamentos e fatores de risco é um passo obrigatório para a superação dessas desigualdades e promoção do melhor cuidado CV possível para todos os brasileiros. Este estudo reúne essa informação, que é essencial não só para o cuidado individual e para o planejamento dos passos seguintes das políticas de saúde no Brasil,^21^ mas também indica as lacunas no conhecimento a serem preenchidas com estudos adicionais. Todos desejamos que as pessoas vivam mais e melhor; portanto, saber mais sobre a estatística CV para melhor enfrentar as DCV é um bom começo na busca desse objetivo.

### Sistemas de Informação de Mortalidade e de Saúde no Brasil

As principais fontes de dados brasileiros para a ***Estatística Cardiovascular – Brasil*** são os sistemas de informação de morbidade e mortalidade, que compreendem o SIM e o SIH, as pesquisas de saúde periódicas, como a PNS, e as estimativas populacionais oficiais, especificados a seguir:

A. Sistema de Informações sobre Mortalidade: O SIM é responsável por coletar, armazenar, gerenciar e divulgar dados nacionais de mortalidade. O Ministério da Saúde utiliza um modelo de declaração de óbito padrão para coletar informação sobre morte, que emprega a CID para codificar as causas de morte. Além disso, um fluxo para coletar, processar e distribuir a informação sobre morte foi implementado em todos os 5.570 municípios do país.^22 - 27^ A qualidade da estatística sobre causas de morte no Brasil, baixa no início dos anos 2000, em especial em algumas partes do país, melhorou significativamente nas duas últimas décadas.^28^ Por conhecer a heterogeneidade desses indicadores no Brasil e buscando uma estimativa da informação mais próxima da situação real, o relatório ***Estatística Cardiovascular – Brasil*** tratou os dados, realizando a correção para subnotificação e a redistribuição das causas de morte mal definidas.^25^

B. Sistema de Informações Hospitalares: O conjunto de dados do SIH registra todas as hospitalizações financiadas pelo SUS em nível municipal através da ‘Autorização de Internação Hospitalar’, que contém informação sobre as doenças que levaram à hospitalização (usando a CID-10), o tempo de permanência, os procedimentos e os custos.^26^ A informação do SIH-SUS permite o desenvolvimento de metodologias e a definição de indicadores para identificar disparidades geográficas relacionadas aos recursos hospitalares.^27^

C. Pesquisa Nacional de Saúde: A PNS é um inquérito de base domiciliar, representativo do Brasil, de suas grandes regiões e UF, regiões metropolitanas, capitais e de outros municípios em cada UF. A primeira versão da PNS foi conduzida em 2013 com uma amostra de 64.348 domicílios. Em 2019, um segundo inquérito foi conduzido em mais de 94.114 domicílios.^28^ A pesquisa incluiu a maioria dos tópicos de saúde, como doenças não transmissíveis, função renal, idosos, mulheres, crianças, utilização dos serviços de saúde, desigualdades em saúde, características antropométricas, exames laboratoriais, além de aferição da pressão arterial.^29^

D. Para as estimativas populacionais, utilizaram-se no denominador as estimativas populacionais mais atualizadas geradas pelo IBGE ( www.ibge.gov.br ). Para as hospitalizações e análises de custo, utilizou-se a população residente estimada para o Tribunal de Contas da União anualmente, de 2008 a 2021.

### Estudo Global Burden of Disease

O Estudo GBD ( http://www.healthdata.org/gbd ) é o mais abrangente estudo epidemiológico observacional de âmbito mundial até o momento. Descreve mortalidade e morbidade decorrentes das principais doenças, injúrias e fatores de risco em níveis global, nacional e regional. O exame das tendências a partir de 1990 até o presente, assim como as comparações entre populações, permite compreender os desafios em saúde enfrentados pelas pessoas em todo o mundo no século 21. A Rede GBD Brasil^1^ tem colaborado com o IHME, que lidera o projeto em âmbito mundial, para a identificação e a provisão de conjuntos de dados, a revisão de modelos e estimativas, bem como a validação e a publicação de resultados para o Brasil.^30 , 31^ Detalhes de como as estimativas são calculadas podem ser obtidos no *website* do IHME ( http://www.healthdata.org/acting-data/what-we-measure-and-why ). O Estudo GBD 2019 é o último conjunto de dados disponibilizado publicamente.^32^ Em 2022, uma atualização para as DCV com dados limitados atualizados para 2021 foi divulgada e essas estimativas também foram usadas no nosso relatório.^37^ As principais estimativas usadas neste documento estão resumidas a seguir:

A. Estimativas de mortes e de causas de morte. A principal fonte de informação é o SIM, um conjunto de dados do Ministério da Saúde, ajustado para outras fontes nacionais e internacionais. O IHME corrigiu a subnotificação de mortes e as mortes com “código *garbage* ” através da utilização de algoritmos previamente publicados,^38^ atualizados nas versões mais recentes do estudo ( http://www.healthdata.org/acting-data/determining-causes-death-how-we-reclassify-miscoded-deaths ).

B. Os YLLs são os anos perdidos em razão de mortalidade prematura, sendo calculados subtraindo-se a idade à época da morte da maior expectativa de vida possível para uma pessoa em qualquer idade. Por exemplo, se a maior expectativa de vida para os homens em um certo país for de 75 anos e se um homem morre de câncer aos 65 anos nesse país, tem-se 10 anos potenciais de vida perdidos para o câncer.

C. Os YLDs, anos vividos com incapacidade, também podem ser descritos como os anos vividos com saúde inferior à ideal. Estão aqui incluídas condições como influenza, que pode durar apenas uns poucos dias, ou epilepsia, que pode durar uma vida inteira. Os YLDs podem ser calculados ao se multiplicar a prevalência da condição pelo peso da incapacidade por ela gerada. Os pesos da incapacidade refletem a gravidade de diferentes condições e são desenvolvidos através de pesquisas com o público em geral.

D. DALYs é uma abreviatura para anos de vida perdidos ajustados por incapacidade. Os DALYs são uma métrica universal que permite que pesquisadores e formuladores de políticas comparem populações e condições de saúde muito diferentes ao longo do tempo. Os DALYs correspondem à soma dos YLLs e YLDs, sendo 1 DALY igual a 1 ano de vida saudável perdido. Esse índice permite que se estime o número total de anos perdidos devido a causas específicas e fatores de risco em níveis global, nacional e regional.

### Revisão Sistemática da Literatura

Os descritores para a elaboração das estratégias de busca foram selecionados no MeSH e no DeCS, os vocabulários controlados da MEDLINE e da LILACS, respectivamente. O plano da Embase foi desenhado com descritores Emtree em associação com MeSH. Além disso, termos livres foram usados, i.e., palavras-chave significativas e seus sinônimos, variações ortográficas e acrônimos essenciais para a busca no domínio pesquisado, mas que não são descritores controlados (ou não estão na lista de sinônimo desses descritores). É importante lembrar que, para manter a uniformidade, os mesmos descritores foram usados em todas as estratégias de busca. Entretanto, as estratégias foram customizadas conforme as especificidades de cada base de dados. Vale ainda lembrar que o grupo de termos relacionados a ‘Brasil’ foi em geral utilizado em todos os campos de pesquisa (assunto, autor, título, afiliação institucional, nome do periódico, etc.).

As bases selecionadas para busca foram a MEDLINE através da PubMed, Embase, LILACS, CINAHL, *Cochrane Library Scopus* e *Web of Science* . Os seguintes filtros e limites da pesquisa bibliográfica foram utilizados: período de publicação (2004-2022); línguas: português, inglês e espanhol; tipo de estudo/publicação: Revisão, Meta-Análise, Ensaio Clínico, Ensaio Randomizado Controlado, Estudo Comparativo, Diretriz de Prática, Diretriz, Revisão Sistemática, Estudo de Avaliação, Publicação Governamental e Estudo Multicêntrico. Todas as referências foram organizadas usando-se o *EndNote Web* . A partir da busca, os artigos foram incluídos caso os estudos fossem de base populacional ou comunitária. Deu-se preferência aos estudos de âmbito nacional ou estadual. Os estudos conduzidos em serviços de saúde ou hospitais foram incluídos caso fossem multicêntricos e possuíssem tamanho amostral adequado (> 200 participantes foi o ponto de corte sugerido). Além dos artigos identificados na busca sistemática, os autores puderam incluir outros encontrados nas referências dos artigos buscados ou outros de que tivessem conhecimento em suas áreas de especialidade, caso os estudos atendessem aos critérios acima mencionados. Por fim, a decisão de quais estudos incluir em cada capítulo coube principalmente aos especialistas designados para o tema em questão.

### Utilização da Atenção à Saúde

Os estudos sobre o custo da atenção à saúde apresentam grande variabilidade metodológica e precisam ser interpretados com cautela. No presente documento, a maior parte dos dados sobre custo foi obtida das bases de dados de reembolso do Sistema Público de Saúde de 2008 a 2021. Durante esse período, o reajuste pela inflação não foi realizado de maneira regular nem homogênea nos grupos e nos procedimentos de DCV. A taxa de inflação brasileira (baseada no IPCA) de 2008 a 2018 foi 76,3%, enquanto a inflação média para os procedimentos CV foi 43,5%. Para alguns códigos de procedimento, o reajuste foi mínimo, como para a implantação de *stent* coronariano, cujo reajuste foi de 8,7%. Para outros códigos, o reajuste ficou acima da inflação, como para o tratamento de arritmias (83,4%).

Para minimizar o viés na notificação e na interpretação dos dados de custo, aplicou-se uma abordagem sistemática em todos os capítulos. Nas análises de custo geral, foram utilizadas as unidades monetárias originais [R$ ou US$ em um determinado ano] e Int$. Os Int$ foram convertidos em PPC ajustados para US$ 2021 (Int$ 2021) usando-se o conversor de custo do Centro *Campbell and Cochrane Economics Methods Group Evidence for Policy and Practice Information and Coordination* . Nesse método, aplicou-se uma abordagem em duas etapas. Na primeira, ajustou-se a estimativa original de custo no preço-ano original para o preço-ano alvo, usando-se o índice de deflação do PIB, que, para este relatório, foi o IPCA (taxa de inflação brasileira). Na segunda, houve conversão dessa estimativa ajustada da moeda original para a moeda alvo, usando-se as taxas de conversão baseadas em PPC para o PIB (valores PPC).^39^ Para estudos econômicos originais, quando o ano-base da moeda não foi informado ou não pôde ser inferido a partir do manuscrito (p. e., coleta de dados do ano passado), recomendou-se adotar o ano anterior ao da publicação do manuscrito.

## HIGHLIGHTS

### Capítulo 1 – Doença Cardiovascular Total

•As DCV costumam ser a principal causa de morte no Brasil, onde, dentre todas as DCV, a DAC foi a causa número 1 de morte, seguida por AVC, em 1990 e em 2019. De acordo com o SIM, em 2021, a COVID-19 tornou-se a principal causa de morte de homens e mulheres.

•Nos anos iniciais da pandemia de COVID-19, 2020 e 2021, houve redução significativa nas admissões hospitalares para todos os tipos de DCV. Alguns fatores devem ser considerados, tais como o aumento do número de mortes cardiovasculares fora do hospital, a redução da busca por assistência médica e a admissão hospitalar com concomitância de DCV e COVID-19 registrada como diagnóstico primário.

•Mais de R$ 1 bilhão são gastos anualmente no Brasil pelo SUS com procedimentos cardiovasculares.

### Capítulo 2 – AVC (Doenças Cerebrovasculares)

•Dados do GBD mostram que a taxa de mortalidade padronizada por idade de AVC isquêmico por 100 mil em 2021 foi 31,7 (28 a 33,9) ( Tabela 2-1 e Figura 2-1 ). A maior mortalidade foi observada no Maranhão, 49,8 (42,8 a 56), e a menor, no Rio Grande do Norte, 25 (21,1 a 27,9).

**Tabela 2-1:** – Taxas de mortalidade e DALYs padronizadas por idade por AVC por 100 mil, no Brasil e unidades federativas em 2021

	DALY	Mortalidade
Acre	560,4(513,6-606)	36,5(32,4-39,7)
Alagoas	753,8(667,3-828,1)	47(40,3-51,6)
Amapá	650,8(584-731,2)	42,1(35,8-47,8)
Amazonas	542,9(488,9-595,4)	35(31-38,2)
Bahia	523(454,7-580,2)	32,1(27,6-36)
Brasil	511,4(470,6-542,5)	31,7(28-33,9)
Ceará	513,5(445,6-580,7)	33,6(28,4-38,2)
Distrito Federal	573,3(521,3-630,5)	44,5(39,9-48,7)
Espírito Santo	553,3(494,4-602,7)	34,2(29,3-37,4)
Goiás	481,4(442,4-532,4)	30,4(26,7-33,5)
Maranhão	759,8(674,3-858)	49,8(42,8-56)
Mato Grosso	514,2(468,1-566,2)	30,8(27,2-33,7)
Mato Grosso do Sul	505(455,8-558,7)	31,1(26,9-34,8)
Minas Gerais	435,4(389,6-474,2)	26,4(23,3-28,9)
Pará	670,8(580,7-786,5)	41(34-47,8)
Paraíba	464,1(421,5-510,6)	29,1(25,8-31,8)
Paraná	586(532,3-638)	36,9(33,6-40,5)
Pernambuco	505,3(453,8-556,3)	31,8(27,5-34,9)
Piaui	602,4(523,5-672,7)	38,7(31,6-43,7)
Rio de Janeiro	497,1(444,2-551,7)	29,8(26-33)
Rio Grande do Norte	407,1(359,5-449,7)	25(21,1-27,9)
Rio Grande do Sul	566,6(515,1-614,4)	34,5(30,6-37,6)
Rondônia	514,3(451,9-584,8)	32,5(27,6-36,9)
Roraima	716(609,1-849,4)	48,3(41,2-55,7)
Santa Catarina	491,6(434,5-534,6)	32,1(27,7-35,3)
São Paulo	461,3(410,5-505,7)	28,6(24,3-31,6)
Sergipe	555,7(505,6-603,1)	35,1(30,8-38,7)
Tocantins	622,8(563-700,3)	39,5(34,3-44,9)

Fonte: Dados derivados do Global Burden of Disease Collaborative Network. Global Burden of Disease (GBD) Cardiovascular Burden Estimates 1990 and 2021, Institute for Health Metrics and Evaluation, University of Washington. ^48^

**Figura 2-1:**
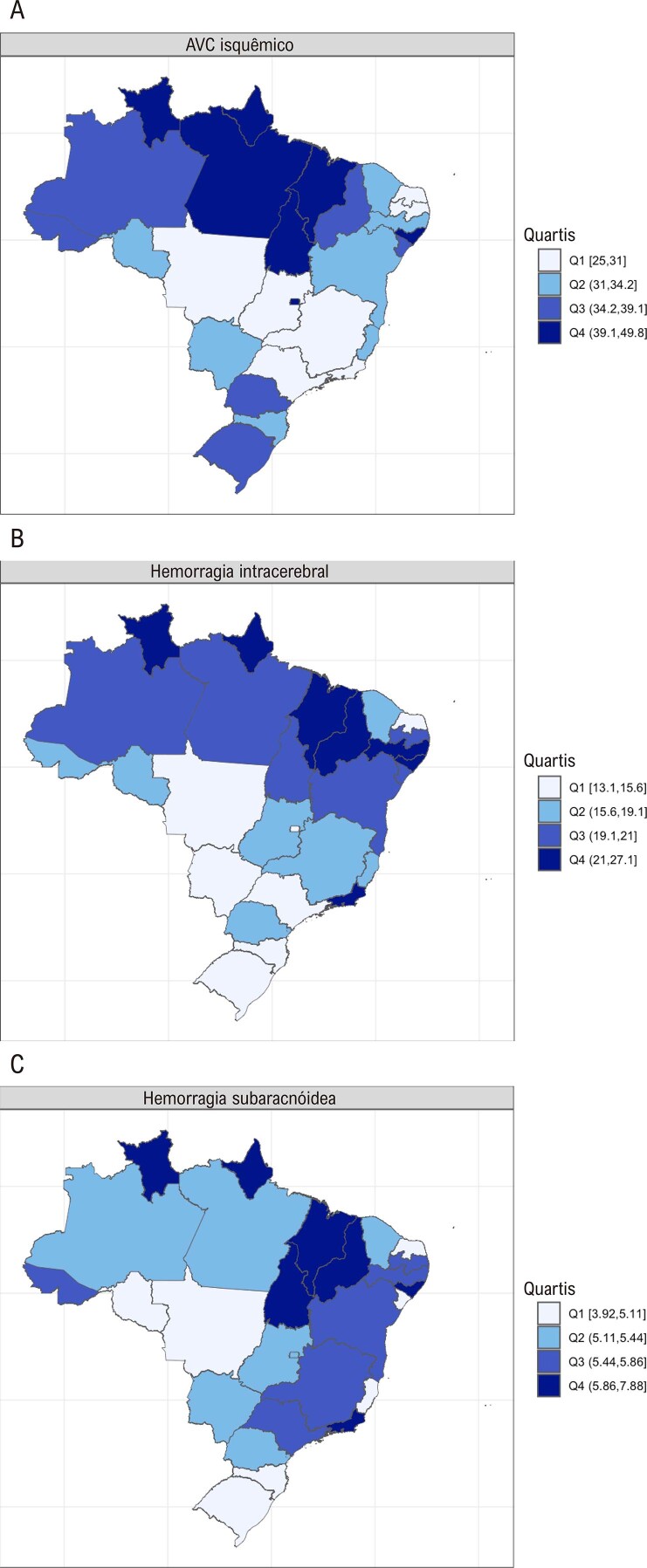
– Taxa de mortalidade padronizada por idade por AVC isquêmico (A), por hemorragia intracerebral (B) e por hemorragia subaracnóidea (C) por 100 mil habitantes, 2021. Dados derivados das estimativas do Estudo Global Burden of Disease 2021. ^48^

•Dados do GBD mostram que a taxa de DALYs padronizada por idade por 100 mil por AVC isquêmico em 2021 foi 511,4 (470,6 a 542,5). A mais alta taxa de DALYs foi observada no Maranhão, 759,8 (674,3 a 858), e a mais baixa, no Rio Grande do Norte, 407,1 (359,5 a 449,7).

•Os custos relacionados a hospitalizações por AVC e ajustados para a inflação de 2008 a 2021 chegaram a US$ 1.195.975.877,98, variando de US$ 28.661.321,88, em 2008, a US$ 66.843.953,39, em 2021.

•Recentemente, ocorreram várias iniciativas para promover a conscientização do público sobre AVC no Brasil, em especial através de campanhas anuais por ocasião do Dia Mundial do AVC (29 de outubro) conduzidas pela Organização Mundial do AVC. Apesar desses esforços, vários estudos mostraram uma alarmante falta de conhecimento sobre os fatores de risco e tratamento do AVC, assim como de reconhecimento dos sintomas de AVC como uma emergência médica.

### Capítulo 3 – Doença Arterial Coronariana Aguda e Crônica

•De acordo com as estimativas do GBD para o ano 2021, a taxa de mortalidade padronizada por idade por DAC foi 67,1 (II 95%, 60,9-71,0) por 100 mil habitantes no Brasil.

•A pandemia de COVID-19 impactou substancialmente a estatística relacionada à DAC no Brasil. Dados do SUS mostraram que as admissões hospitalares por SCC e SCA caíram 12,8% (IC 95%, 12,5%-13,2%) e 13,6% (IC 95%, 13,3%-13,9%), durante a pandemia em comparação aos valores médios nos três anos anteriores (2017-2019). A mortalidade hospitalar e a proporção de óbitos domiciliares relacionadas a SCC e SCA aumentaram durante a pandemia.

•O número de ICP realizadas nos hospitais públicos mais do que dobrou de 2008 a 2022, embora o ticket médio por caso tenha caído à metade (valores ajustados: de R$ 12.916 em 2008 para R$ 6.443 em 2022). O valor ajustado reembolsado por CRVM (ticket médio) diminuiu em ~23% (de R$ 20.339 em 2008 para R$ 15.723 em 2021).

### Capítulo 4 – Cardiomiopatia e Insuficiência Cardíaca

•Um estudo da base de dados DATASUS mostrou uma redução nas hospitalizações por insuficiência cardíaca e cardiomiopatia no Brasil durante a pandemia de COVID-19, associado ao aumento na gravidade clínica dos pacientes hospitalizados e nas mortes hospitalares.

•A despeito da preocupação quanto ao impacto da coinfecção por COVID-19 e DCh, os dados disponíveis não mostram aumento do risco de morte nesses pacientes.

•Estudos recentes confirmam redução na prevalência de DCh e na sua mortalidade no Brasil nas últimas décadas.

### Capítulo 5 – Doença Valvar do Coração

•Enquanto a prevalência de DCR no Brasil e na América do Sul tropical permanece relativamente estável, com aumentos explicados por melhor acesso ao diagnóstico e incorporação da doença latente nos modelos, a mortalidade associada tende a diminuir.

•Há uma crescente conscientização sobre a doença na região, reforçada por iniciativas de pesquisa e programas locais.

•As alterações no perfil epidemiológico e na composição etária no Brasil estão levando a uma crescente carga de DVC não reumática, em especial doenças valvares mitral e aórtica degenerativas, que requerem ações e políticas específicas, principalmente do setor público.

•Houve impacto importante e multifatorial da pandemia de COVID-19 nas hospitalizações associadas com manejo clínico e cirúrgico das DVC, sendo que os números tenderam a retornar aos níveis basais apenas no final de 2021 e 2022. Padrão similar foi observado para os custos associados.

•Considerando as restrições orçamentárias e os crescentes custos dos procedimentos cirúrgicos e percutâneos e dos dispositivos, são necessários investimentos para tratar as DVC no Brasil, em especial para abordar as necessidades específicas que emergiram dos dados administrativos e científicos nos últimos anos.

### Capítulo 6 – Fibrilação Atrial e Flutter Atrial

•No estudo de coorte ELSA-Brasil, que incluiu 14.424 adultos com ECG válidos (45,8% homens; idade média, 51 anos; faixa etária, 35-74 anos), a prevalência de FA e flutter atrial confirmada no ECG ou autorrelatada foi 2,5%.

•No registro multicêntrico prospectivo RECALL que incluiu e acompanhou 4.585 pacientes com FA por 1 ano em 89 sites em todo o Brasil de abril de 2012 a agosto de 2019, ocorreu morte em 8,8/100 pacientes/ano (IC 95%, 8,0-9,6), que, em modelos multivariados, foi associada com idade mais avançada, FA permanente, classe III/IV da NYHA, doença renal crônica, doença arterial periférica, AVC, doença pulmonar obstrutiva crônica e demência. O uso de anticoagulantes foi associado a menor mortalidade. Pacientes com TTR <60% apresentaram maior mortalidade e mais eventos de sangramento maior em comparação aos pacientes com TTR ≥60%. Durante a pandemia de COVID-19, houve redução em hospitalizações e procedimentos para FA, em particular em 2020. Essa redução relacionou-se provavelmente aos efeitos indiretos da pandemia, quando houve uma diminuição nas hospitalizações cardiovasculares em geral no Brasil e no mundo.

### Capítulo 7 – Hipertensão

•A porcentagem de pacientes com idade igual ou superior a 18 anos com diagnóstico autorreferido de hipertensão nas capitais brasileiras (Vigitel) foi de 26,3% em 2021.

•A associação de hipertensão e seu controle com determinantes sociais de saúde, como urbanização, sexo e raça, tem sido repetidas vezes mostrada no Brasil recentemente.

•Durante a pandemia de COVID-19, houve mudança na prevalência de hipertensão, que passou de tendência a estabilidade de 2007 a 2019 para discreta elevação em 2020 e 2021 nas capitais brasileiras, conforme dados do Vigitel.

### Capítulo 8 – Dislipidemia

•A PNS de 2019 utilizou o diagnóstico autorreferido de colesterol alto e, nos 88.531 adultos avaliados, identificou-se uma prevalência de 14,6% de colesterol alto. Os fatores mais fortemente associados a essa condição, medidos pela sua RP foram: sexo feminino, idade ≥ 60 anos, possuir plano de saúde, autoavaliação de saúde ruim ou muito ruim, ter hipertensão, diabetes ou insuficiência renal, ser obeso ou ex-fumante, consumir álcool abusivamente, ser ativo no lazer.

•Estudos contextualizando o papel dos marcadores de aterosclerose subclínica na prática médica e o efeito do uso desses marcadores substitutos de aterosclerose na modificação do tratamento clínico, assim como a custo-efetividade dessa utilização, são necessários.

•A frequência de rastreio, tratamento e controle da dislipidemia no Brasil, de acordo com sexo e grupos etários, precisa ser investigada. Além disso, o impacto da dislipidemia no sistema de saúde, incluindo custos, ainda não foi avaliado de maneira mais abrangente.

### Capítulo 9 – Diabetes Mellitus

•Houve redução no número de casos de diabetes desconhecidos no Brasil, o que pode ter ocorrido por maior taxa de rastreamento e maior acesso ao diagnóstico. No entanto, o acesso ao diagnóstico ainda se caracteriza por desigualdades, sendo maior em mulheres, pessoas autodeclaradas brancas, idosos e com maior nível educacional, e menor em pessoas autodeclaradas pretas, de áreas rurais e naquelas sem plano de saúde privado.

•Os indicadores de cuidado de pessoas com diabetes têm sido monitorados, demonstrando aumento no número de pessoas que receberam tratamento médico, mas piora no percentual de rastreamento de complicações crônicas em homens, pessoas mais jovens, de cor preta e com menores níveis socioeconômicos e educacionais.

•À semelhança dos dados de outros países, os dados brasileiros mostraram que a síndrome respiratória aguda grave por COVID-19 em pessoas com diabetes levou a maior probabilidade de internações em unidade de terapia intensiva e maior mortalidade, quando comparadas a pessoas sem diabetes.

### Capítulo 10 – Tabagismo e Uso de Tabaco

•A prevalência de tabagismo na população adulta apresentou redução total (0,7%) de 2019 a 2021 em ambos os sexos.

•A prevalência do uso de outros produtos derivados do tabaco nos últimos 30 dias entre adolescentes aumentou de 2015 (7,2%; IC 95% 6,1-8,2%) para 2019 (12,4%; IC 95%, 11,8-12,9%).

•A prevalência do uso de cigarros eletrônicos entre indivíduos com idade de 15-65 anos aumentou de 0,45% em 2015 para 0,72% em 2019.

•A carga econômica total atribuível ao tabaco no Brasil em 2020 foi estimada em US$ 24,3 bilhões, representando 1,9% do PIB e 7,8% das despesas nacionais em saúde.

### Capítulo 11 – Obesidade e Sobrepeso

•Houve tendência de aumento nas porcentagens de sobrepeso e obesidade no Brasil de 2006 a 2021, de acordo com dados do VIGITEL. A maioria das capitais mostrou porcentagens mais altas de obesidade do que os valores nacionais para ambos os sexos, exceto Belo Horizonte, Campo Grande, Florianópolis, Palmas, Rio de Janeiro, Salvador, São Luís, Teresina e Vitória.

•Nas capitais brasileiras, a porcentagem de adultos (≥18 anos) com obesidade em 2021 foi 22,4% (22,0% para homens e 22,6% para mulheres). Aumento progressivo de obesidade foi observado com o aumento da idade, variando de 12,2% no grupo etário de 18-24 anos a 26,2% no grupo etário de 45-54 anos. Para o grupo etário de 60+ anos, houve discreta redução na prevalência de obesidade, 21,8%.

•Aumento na prevalência de obesidade também foi observado entre crianças e adolescentes brasileiros.

### Capítulo 12 – Atividade Física

•Apesar do conhecimento crescente em relação aos benefícios cardiovasculares da atividade física regular e uma tendência à redução da inatividade física entre os brasileiros nos últimos anos, quase metade da população brasileira não alcança o nível mínimo recomendado de atividade física, sendo os números mais preocupantes os de mulheres, idosos e aqueles com menor nível de escolaridade.

•A pandemia da COVID-19 impactou negativamente esse cenário, uma vez que foi observado um aumento no tempo dedicado a comportamentos sedentários em todo o país, principalmente entre as mulheres e os adultos jovens com idade entre 18 e 29 anos.

•Dados do GBD 2019 mostram que, apesar de uma redução de 47,6% da taxa de mortalidade por DCV atribuíveis aos baixos níveis de atividade física entre 1990 e 2019, 7,6% do total de mortes por DCV no Brasil ainda é atribuído a esse fator de risco.

## CAPÍTULO 1 – DOENÇA CARDIOVASCULAR TOTAL

### CID-9 390 a 459; CID-10 I00 a I99.

**Ver Tabelas 1-1 até 1-7 e Figuras 1-1 até 1-7**

**Table:** Abreviaturas usadas neste capítulo

AVC	Acidente Vascular Cerebral
CID	Classificação Estatística Internacional de Doenças e Problemas Relacionados à Saúde
COVID-19	Doença do novo coronavírus 2019
DAC	Doença Arterial Coronariana
DALYs	Anos de vida perdidos ajustados por incapacidade (do inglês, * Disability-Adjusted Life-Years* )
DATASUS	Base de dados do Departamento de Informática do Sistema Único de Saúde
DCV	Doença Cardiovascular
DNC	Doenças Não Comunicáveis
ELSA-Brasil	Estudo Longitudinal de Saúde do Adulto
GBD	Global Burden of Disease
IBGE	Instituto Brasileiro de Geografia e Estatística
IC	Intervalo de Confiança
IDH	Índice de Desenvolvimento Humano
IDHm	Índice de Desenvolvimento Humano Municipal
II	Intervalo de Incerteza
NCDP	Doenças Não Comunicáveis associadas à Pobreza (do Inglês, *Non-Communicable Disease of Poverty* )
OMS	Organização Mundial da Saúde
OR	Odds Ratio
PIB	Produto Interno Bruto
PNS	Pesquisa Nacional de Saúde
SIDRA	Sistema IBGE de Recuperação Automática
SIM	Sistema de Informações sobre Mortalidade
SUS	Sistema Único de Saúde
UF	Unidade Federativa

### Panorama

•As DNC constituem o principal grupo de causa de morte em todo o mundo, sendo responsáveis por óbitos prematuros, perda de qualidade de vida, além de impactos econômicos e sociais adversos. As DNC são responsáveis por cerca de 70% das mortes globais, equivalendo a mais de 38 milhões de óbitos por ano, excedendo significativamente as mortes por causas externas e por doenças infecciosas.^40 - 43^ Cerca de 33% de todas as mortes por DNC no mundo, mais de 18 milhões, são causadas por DCV. Distribuição similar é observada no Brasil, onde 72% das mortes resultam de DNC, sendo 30% devidas a DCV e 16% a neoplasias ( **Figura 1-1** ).^31 , 44 , 45^

**Figura 1-1:**
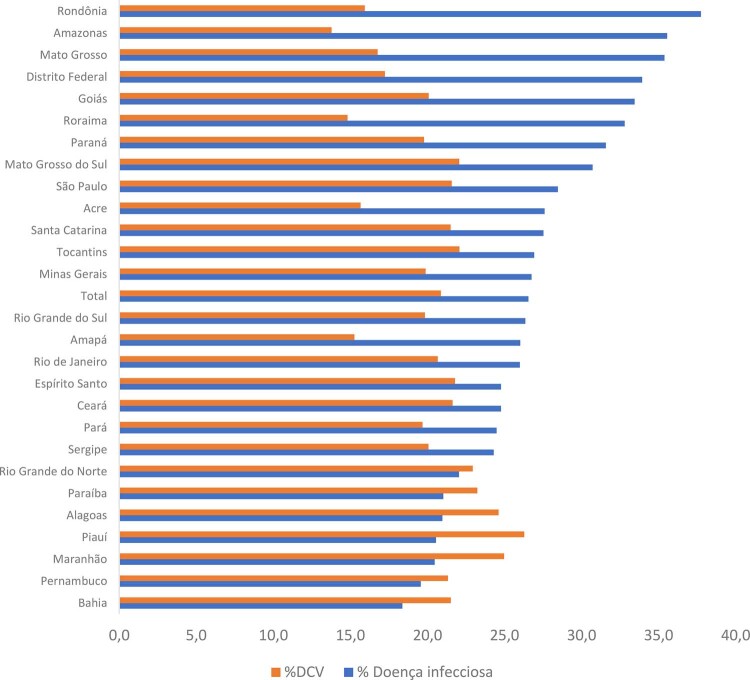
– Mortalidade proporcional por doença infecciosa (CID-10 Cap. I) e doença cardiovascular (Cap. IX) em relação ao total de mortes, Brasil, 2021. DCV: doença cardiovascular. Fonte: Ministério da Saúde do Brasil – Sistema de Informações Hospitalares do Sistema Único de Saúde (SIH/SUS). ^49^

•A definição de DCV pode variar de acordo com o estudo, desde a inclusão de todas as doenças listadas no Capítulo IX da CID-10 até o simples agrupamento das três principais (DAC, AVC e outras doenças cardíacas). Para o GBD, a definição de DCV total engloba dez doenças: cardiopatia reumática, DAC, doença cerebrovascular, cardiopatia hipertensiva, cardiomiopatia, miocardite, fibrilação e *flutter* atrial, aneurisma aórtico, doença vascular periférica e endocardite.^46^

•As DCV são a principal causa de morte prematura no mundo, responsável por aproximadamente um terço de todas as mortes. Espera-se que esse número aumente no futuro.^47^

•As DCV costumavam ser a principal causa de morte no Brasil. De acordo com as estimativas do Estudo GBD 2019, a DAC era a causa número 1 de morte no país, seguida por AVC, em 1990 e 2019. Na verdade, em 2019, a DAC foi a principal causa de morte em todas as UF brasileiras, exceto no Amazonas, na região Norte. Três estados nessa região, Acre, Amapá e Pará, não apresentaram diferença significativa quanto às taxas de mortalidade por DAC e AVC.^4^

•Em 2021, devido à pandemia de COVID-19, as DCV deixaram de ser a principal causa de morte, dando lugar às doenças infecciosas e contagiosas na maioria das UF brasileiras, conforme dados do SIM ( **Figura 1-1** ).^11^

•Em 2021, a COVID-19 foi a principal causa de morte de homens e mulheres. Entre os homens, a segunda principal causa de morte foi DAC, seguida das doenças cerebrovasculares. Entre as mulheres, as doenças cerebrovasculares foram a segunda principal causa de morte e a DAC, a terceira ( **Tabela 1-1** ).^12^

**Tabela 1-1:** – COVID-19 foi a primeira das 10 principais causas de morte no Brasil em 2021

Ranking masculino	Causa	%	Ranking feminino	Causa	%
1	COVID-19	24,3	1	COVID-19	24,0
2	DAC	6,9	2	Doença cerebrovascular	6,4
3	Doença cerebrovascular	5,3	3	DAC	6,0
4	Homicídio	3,9	4	Diabetes mellitus	5,2
5	Infecções respiratórias baixas	3,8	5	Infecções respiratórias baixas	4,4
6	Diabetes mellitus	3,6	6	Alzheimer e outras demências	2,7
7	Acidentes de trânsito	2,6	7	Câncer de mama	2,3
8	Doença pulmonar obstrutiva crônica	2	8	Doença pulmonar obstrutiva crônica	2,0
9	Cirrose hepática	1,7	9	Doença hipertensiva	1,8
10	Câncer de próstata	1,6	10	Cânceres de traqueia, brônquios e pulmão	1,6

DAC: doença arterial coronariana. Fonte: Ministério da Saúde do Brasil Sistema de Informações Hospitalares do Sistema Único de Saúde (SIH/SUS). ^49^

### Prevalência

•A prevalência de DCV aumenta à medida que a idade avança, independentemente do gênero. Entretanto, a diferença na prevalência entre homens e mulheres é mais significativa entre crianças menores de 5 anos de idade. Dos 5 anos aos 44 anos, as mulheres têm maior prevalência do que os homens, mas, depois dos 44 anos, a prevalência entre homens aumenta, sendo a diferença entre os gêneros máxima no grupo etário de 60-69 anos, como mostra a **Figura 1-2** . ^48^

**Figura 1-2:**
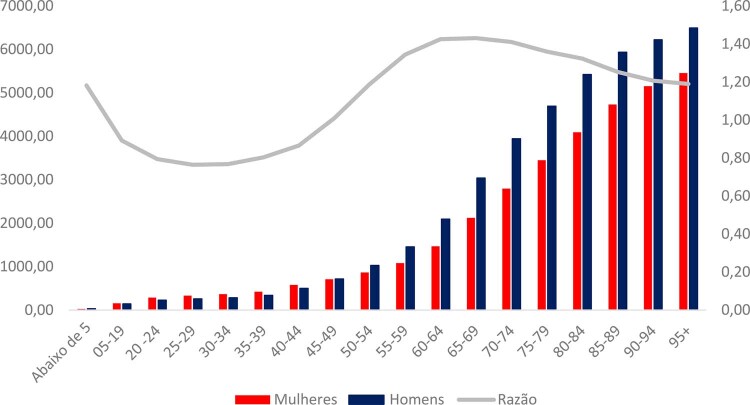
– Prevalência de doença cardiovascular entre homens e mulheres por grupo etário e razão de prevalência, Brasil, 2021.Dados derivados do Global Burden of Disease Collaborative Network. Global Burden of Disease (GBD) Cardiovascular Burden Estimates 1990 and 2021, Institute for Health Metrics and Evaluation, University of Washington. ^48^

•De acordo com uma atualização recente dos dados do GBD, a taxa de prevalência de DCV no Brasil em 2021 foi 6,9% (6,4-7,4) para os dois sexos, sendo maior entre os homens, 7,6% (7,0-8,1), do que entre as mulheres, 6,3% (6,0-6,9).^48 , 49^

•Gonçalves *et al.* publicaram em 2019 um estudo transversal que analisou informação da PNS conduzida em 2013 em uma amostra de 60.202 adultos com mais de 18 anos, estratificados por sexo e grupos etários, usando um modelo de regressão logística binário e hierárquico. O diagnóstico autorreferido de doença cardíaca no Brasil foi de 4,2% (IC 95%: 4,0-4,3 ) e associado com as seguintes características: sexo feminino (OR = 1,1; IC 95%: 1,1-1,1), indivíduos de 65 anos ou mais (OR = 4,7; IC 95%, 3,3-5), hipertensão (OR = 2,4; IC 95%: 2,2-2,7), colesterol elevado (OR = 1,6; IC 95%: 1,5-1,8), sobrepeso (OR = 1,5; IC 95%: 1,4-1,8) ou obesidade (OR = 2,0; IC 95%: 1,7-2,2), sedentarismo (OR = 1,5; IC 95%: 1,02-2,1) e tabagismo (OR = 1,2; IC 95%: 1,03-1,3).^50^

•No estudo ELSA-Brasil, uma coorte que incluiu 15.105 funcionários públicos de seis instituições acadêmicas (54% mulheres, 35-74 anos, com avaliação basal entre 2008 e 2010), a prevalência autorreferida de DCV foi a seguinte: DAC, 4,7% (homens=5,7%, mulheres=4,0%); insuficiência cardíaca, 1,5% (homens=1,9%, mulheres=1,5%); AVC, 1,3% para ambos os sexos; febre reumática, 2,9% (homens=2,2%, mulheres=3,4%); e doença de Chagas, 0,4% para ambos os sexos.^51^

•De acordo com dados da PNS de 2013, a porcentagem de pessoas no Brasil com saúde cardiovascular ideal foi inferior a 1%. Tal porcentagem foi ainda mais baixa entre homens, indivíduos acima dos 60 anos de idade e residentes da região sul do Brasil. Estudos internacionais relataram desfechos similares, indicando que a prevalência de saúde cardiovascular ideal nos países do Cone Sul (Argentina, Chile e Uruguai) e nos Estados Unidos é menor do que 1% e nula, respectivamente.^52^

•De acordo com a Pesquisa Nacional de Saúde, em 2019, haviam 12.946.932 indivíduos com DCV no Brasil, e 51% deles eram homens A taxa de prevalência de DCV diminuiu entre os idosos, mas aumentou entre homens e mulheres no grupo etário de 15-49 anos.^53^

### Incidência

•De acordo com o Estudo GBD 2019, a taxa de incidência de DCV padronizada por idade no Brasil em 2019 foi 475 (II 95%, 447-507) por 100 mil habitantes. De 1990 a 2019, essa taxa diminuiu -20% (-22 a -18).^4^ As últimas estimativas do GBD para DCV são de 2019 e foram discutidas na versão anterior da Estatística Cardiovascular – Brasil (2021).^4^ Estimativas atualizadas do GBD não haviam sido disponibilizadas até a finalização deste documento.

•Globalmente, há uma incidência crescente de doenças não comunicáveis associadas à pobreza (NCDP), incluindo as DCV (principalmente doença cardíaca e AVC), diabetes, osteoartrite e vários tipos de câncer (mama, próstata, fígado, rins e cólon). A despeito da já bem-estabelecida transição epidemiológica de doenças infecciosas para DNC, altos níveis de várias doenças infecciosas continuam a ocorrer concomitantemente com as DNC, as primeiras frequentemente associadas com infraestrutura precária ou limitada, habitações impróprias, aglomerações e condições insalubres. Isso é característico de países pobres, mas também ocorre nas comunidades carentes em contextos de alta renda.^53 , 54^

### Mortalidade

•Dados do Estudo GBD 2021 revelam que, embora as taxas de mortalidade por DCV no Brasil tenham caído significativamente nos últimos anos, o número total de mortes por DCV aumentou devido ao crescimento e envelhecimento populacional ( **Figura 1-4** ). A taxa de mortalidade padronizada por idade por 100 mil habitantes foi 348,5 (325;359,5) em 1990 e 162,2 (145,8;171,5) em 2021, uma redução de -53,5%. As taxas de mortalidade padronizadas por idade foram mais altas entre os homens em todo o período e a redução percentual foi maior nas mulheres (-56%) do que nos homens (-50,5%).^48^

**Figura 1-4:**
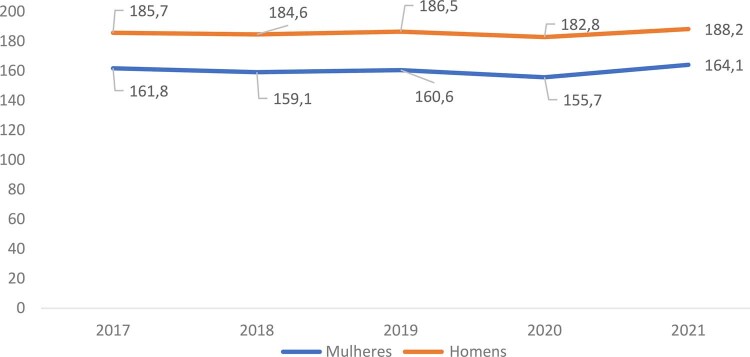
– Tendências das taxas brutas de mortalidade por doença cardiovascular no Brasil, homens e mulheres, 2017-2021.

Dados do Ministério da Saúde do Brasil – Sistema de Informações Hospitalares do Sistema Único de Saúde (SIH/SUS). ^49^

•Foi observada uma variação entre os estados brasileiros, com as cinco maiores taxas de mortalidade padronizada por idade reportadas no Maranhão, Roraima, Amapá, Alagoas, Tocantins, Pernambuco e Rio de Janeiro. As cinco taxas de mortalidade padronizada por idade mais baixas foram observadas no Amazonas, Rio Grande do Sul, Bahia, Rio Grande do Norte, Minas Gerais, Mato Grosso e Santa Catarina ( **Figura 1-3 e Tabela 1-2** ).^48^

**Figura 1-3:**
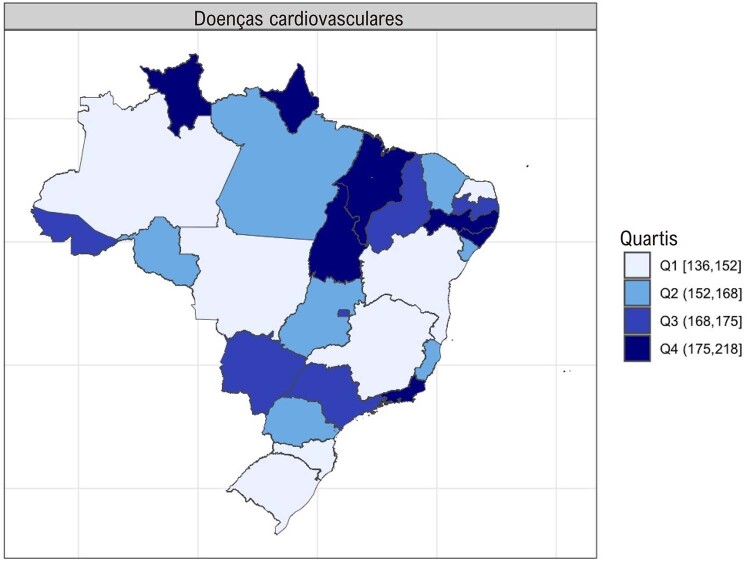
– Taxa de morte padronizada por idade por doença cardiovascular, ambos os sexos, Brasil e unidades federativas, 2021. Dados derivados do Global Burden of Disease Collaborative Network. Global Burden of Disease (GBD) Cardiovascular Burden Estimates 1990 and 2021, Institute for Health Metrics and Evaluation, University of Washington. ^48^

**Tabela 1-2:** – Taxas de mortalidade por doença cardiovascular padronizadas por idade, Brasil e unidades federativas, 2021

Unidades federativas	Mortalidade
Acre	168,8(153,6-181,3)
Alagoas	203,8(178,4-228,3)
Amapá	176(154,9-198,3)
Amazonas	145,2(130,7-157,6)
Bahia	148(130,6-164,3)
Ceará	164(141,1-185,4)
Distrito Federal	172(153,8-188,1)
Espírito Santo	165,9(145,4-180,8)
Goiás	159,2(142,4-174,1)
Maranhão	218,1(196,1-241,4)
Mato Grosso	151,7(133,6-165,8)
Mato Grosso do Sul	170,9(153,5-188,6)
Minas Gerais	135,6(120,4-147,7)
Pará	168,2(143,5-196,1)
Paraíba	169,5(154,2-185,3)
Paraná	158,7(144,9-172,8)
Pernambuco	181,2(158,2-197,7)
Piauí	174(149,3-194,7)
Rio de Janeiro	182,1(160,3-200,7)
Rio Grande do Norte	148,5(130,7-164)
Rio Grande do Sul	147,3(133,5-159,8)
Rondônia	156,2(135,4-176)
Roraima	211,6(182,7-243)
Santa Catarina	150,9(132,4-164,3)
São Paulo	168,9(148,5-186,4)
Sergipe	151,9(135-165,7)
Tocantins	190,2(168,6-214,6)
Brasil	162,2(145,8-171,5)

Fonte: Dados derivados do Global Burden of Disease Collaborative Network. Global Burden of Disease (GBD) Cardiovascular Burden Estimates 1990 and 2021, Institute for Health Metrics and Evaluation, University of Washington. ^48^

•Em 2021, o Brasil passou por uma significativa mudança no seu perfil de mortalidade devido à pandemia. Pela primeira vez, as doenças infecciosas tornaram-se a causa de morte primária, ultrapassando as DCV por mais de 100 mil mortes. Doenças infecciosas foram responsáveis por 27% de todas as mortes, com 486.667 mortes, enquanto as DCV causaram 382.507 mortes, representando 21% de todas as mortes. Entre as doenças infecciosas, a COVID-19 foi a principal causa de morte, com 424.461 mortes em 2021.^48^

•A taxa de mortalidade por DCV é mais alta entre os homens do que entre as mulheres em quase todos os grupos etários. Como mostra a **Figura 1-5** , a razão ‘homens/mulheres’ tem um pico no grupo etário ‘50-69 anos’ e declina até o grupo etário ‘90-94 anos’. Entretanto, depois dos 95 anos, mulheres apresentam taxa de mortalidade mais alta do que homens.^48^

**Figura 1-5:**
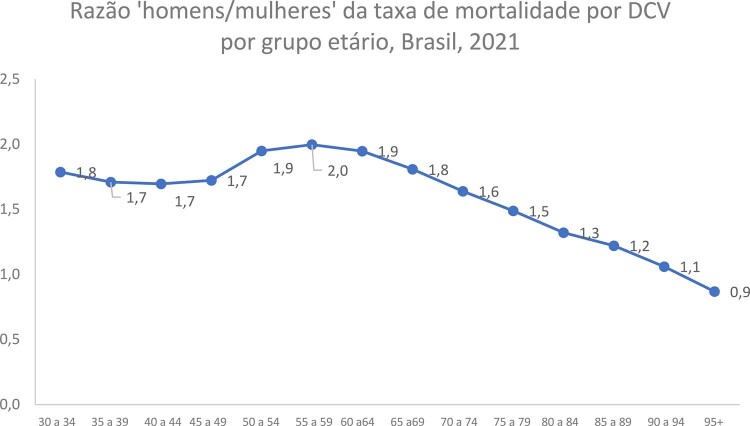
– Razão ‘homens/mulheres’ da taxa de mortalidade por doença cardiovascular por grupo etário, Brasil, 2021. DCV: doença cardiovascular. Dados derivados do Global Burden of Disease Collaborative Network. Global Burden of Disease (GBD) Cardiovascular Burden Estimates 1990 and 2021, Institute for Health Metrics and Evaluation, University of Washington. ^48^

•Antes da pandemia de COVID-19, a DCV era a principal causa de morte em âmbito global e, em 2019, 58% das mortes ocorridas na Ásia foram devidas à DCV. A OMS estabeleceu um plano de ação global para até o ano 2025, visando a reduzir em 25% o número de mortes prematuras por DNC, incluindo DCV.^55^

•No Brasil, Mansur *et al* . relataram que a taxa de mortalidade por DCV padronizada por idade diminuiu significativamente nas últimas décadas. Um estudo de 2016 analisou as taxas de mortalidade por DCV a partir dos 30 anos de idade, por sexo, por 100 mil habitantes. As variações anuais na mortalidade cardiovascular nos períodos 1980-2006 e 2007-2012 foram, respectivamente: -1,5% e -0,8%, para ambos os sexos; -1,4% e -0,6%, para homens; -1,7% e -1,0%, para mulheres.^56^

•Baptista *et al* . investigaram como a composição etária e as taxas de mortalidade específicas por idade se relacionam à diferença observada nas mortes por DCV na população adulta, por sexo, nas microrregiões brasileiras de 1996 a 2015. Aqueles autores sugeriram, após correção para subnotificação das mortes, que houvesse uma redução nas taxas de morte por DCV no período estudado. Entretanto, o principal motivo da mudança nas taxas de mortalidade foi heterogêneo nas microrregiões brasileiras. Em geral, nas áreas mais desenvolvidas socioeconomicamente, a estrutura etária relacionou-se de maneira mais importante às taxas de mortalidade, com as populações mais idosas morrendo por DCV. É interessante notar que os principais motivos de mudança nas taxas de mortalidade por DCV diferiram ainda dentro das regiões e das UF brasileiras.^57^

•Malta *et al.* compararam uma série histórica de taxa de mortalidade por DCV no Brasil, usando a base de dados do SIM com e sem correção e as estimativas do GBD 2017 entre 2000 e 2017. Os autores indicaram que o aumento na taxa de mortalidade observado em 2017 em algumas UF do Norte e Nordeste deveu-se às melhorias nos registros de morte e à melhor classificação das causas básicas de morte nos últimos anos.^25^

•Ao analisarem dados do GBD 2015, Brant *et al.* observaram uma redução na taxa de mortalidade por DCV padronizada por idade de 429,5 (1990) para 256,0 (2015) por 100 mil habitantes (-40,4%), com acentuadas diferenças entre as UF. Essa redução foi mais pronunciada nas UF do Sudeste e Sul e no Distrito Federal, regiões que concentram as maiores populações e renda, sendo mais modesta na maioria dos estados do Norte e Nordeste.^46^

•A cobertura do Programa Saúde da Família foi associada a redução nas hospitalizações e na mortalidade por DCV que foram incluídas na Lista de Condições Sensíveis à Atenção Primária no Brasil, tendo seu efeito aumentado de acordo com a duração da implementação do Programa Saúde da Família no município. Rasella *et al* . relataram reduções nas mortalidades por doença cerebrovascular e doença cardíaca de 0,82 (IC 95%: 0,79-0,86) e 0,79 (IC 95%: 0,75-0,80), respectivamente, chegando a 0,69 (IC 95%: 0,66-0,73) e 0,64 (IC 95%: 0.59-0.68), respectivamente, quando a cobertura do Programa Saúde da Família foi consolidada no total dos 8 anos estudados.^58^

•Estudo mostrou uma forte associação entre baixo *status* socioeconômico e maior carga de DCV. À medida que aumenta o *status* socioeconômico, as taxas de DCV tendem a cair. Isso sugere que melhorias nas condições socioeconômicas locais tenham levado a uma redução na mortalidade por DCV, achado consistente com os de outros estudos.^59^

•Lotufo *et al.* compararam três diferentes níveis de renda domiciliar (alto, médio e baixo) com taxas de mortalidade por DCV, na cidade de São Paulo, de 1996 a 2010. As variações percentuais anuais e os IC 95% para homens residentes em áreas de renda alta, média e baixa foram -4,1 (-4,5 a -3,8), -3,0 (-3,5 a -2,6) e -2,5 (-2,8 a -2,1), respectivamente. As tendências para as taxas de mulheres residentes em áreas de renda alta foram -4,4 (-4,8 a -3,9) em 1996-2005 e -2,6 (-3,8 a -1,4) em 2005-2010. A redução nas mortes por DCV foi mais significativa para homens e mulheres residentes em áreas mais abastadas, com um gradiente decrescente para risco de morte maior para os residentes de áreas mais abastadas em comparação àqueles de áreas mais carentes.^60^

•Observou-se associação inversa do IDHm e da cobertura de saúde suplementar com a mortalidade por DCV, sugerindo uma relação entre fatores socioeconômicos e DCV. O IDHm aumentou entre 2000 e 2010 em todas as UF, sendo 0,7 ou maior na metade das UF. A cobertura de saúde suplementar aumentou no país durante o período estudado e associou-se inversamente com mortalidade por DCV entre 2004 e 2013.^61^

•Soares *et al.* observaram uma diminuição na mortalidade por DCV nos estados do Rio de Janeiro, São Paulo e Rio Grande do Sul que precedeu a melhoria no índice socioeconômico. A evolução do PIB per capita, o declínio da mortalidade infantil, o maior nível educacional (representado pela escolaridade, em anos, dos indivíduos com idade superior a 25 anos) e o IDHm mostraram uma grande correlação com a redução na taxa de mortalidade por DCV. A redução nas taxas de mortalidade por DCV, AVC e DAC no estado do Rio de Janeiro nas últimas décadas foi precedida por um aumento no IDH. Um acréscimo de 0,1 no IDH correlacionou-se com as seguintes reduções no número de mortes por 100 mil habitantes: 53,5 por DCV; 30,2 por AVC; e 10,0 por DAC.^62 , 63^

•Baptista *et al* . investigaram a relação entre a taxa de mortalidade por DCV e o desenvolvimento econômico no tempo e no espaço, medido pelo PIB per capita, nas microrregiões brasileiras, de 2001 a 2015. Os autores, usando as bases de dados SIM-DATASUS e SIDRA do IBGE, observaram um rápido declínio na mortalidade por DCV nas regiões Sul e Sudeste, assim como um declínio mais lento na região Centro-Oeste. Por outro lado, as regiões Norte e Nordeste apresentaram um aumento nas taxas de mortalidade por DCV ao longo do tempo, talvez em decorrência do menor acesso aos cuidados em saúde e dos fatores socioeconômicos.^64^

•Silveira *et al.* , estudando o efeito da temperatura ambiente na mortalidade cardiovascular em 27 cidades brasileiras, observaram maior número de mortes cardiovasculares associado com temperaturas baixas e altas na maioria das cidades brasileiras e nas regiões Centro-Oeste, Norte, Sul e Sudeste. O risco relativo geral para o Brasil foi 1,26 (IC 95%, 1,17–1,35) para o percentil 1 de temperatura e 1,07 (IC 95%, 1,01–1,13) para o percentil 99 de temperatura em comparação ao percentil 79 (27,7 °C), cujo risco relativo foi o menor.^65^

### Carga de Doença

•As DCV não apenas impactam a mortalidade populacional ou reduzem a expectativa de vida, mas também causam incapacidade, dessa forma alongando o período em que um indivíduo afetado se torna improdutivo ou dependente dos outros para realizar suas atividades cotidianas. Ademais, indivíduos podem perder sua capacidade de produção econômica. A **Tabela 1-3** apresenta os DALYs para 2021, categorizados por grupo etário e ajustados por idade, para homens e mulheres no Brasil. ^48^

**Tabela 1-3:** – Taxas de DALYs por doença cardiovascular por idade, sexo e ano, no Brasil, 2021

Grupo etário (anos)	Mulheres	Homens	Ambos
Abaixo de 5	327,8(260,1;394,8)	383,9(308;472,4)	356,5(286,1;432,5)
5 a 19	198,1(167,6;235,1)	231,2(200,4;269,7)	214,9(185,7;251,6)
20 a 24	439,6(384,6;494,9)	608,4(552,8;683,7)	524,6(472,5;586,1)
25 a 29	546,4(480,6;613,8)	793,1(739,1;863,7)	668,4(613,3;733,6)
30 a 34	775,9(698,5;861,4)	1190,5(1111,4;1272,3)	978,9(904,8;1055,6)
35 a 39	1198,1(1096,1;1296,4)	1842,9(1739,1;1970,9)	1513,7(1439,4;1602,8)
40 a 44	1955,9(1814,5;2100,1)	3061,7(2905,8;3293,4)	2493(2381,7;2634,4)
45 a 49	2957,4(2776,7;3140,4)	4816(4573,4;5128)	3857,8(3675,1;4030,6)
50 a 54	4034,7(3829,6;4251,5)	7509,3(7126,1;7954,9)	5699,7(5478,4;5943,6)
55 a 59	5396,1(5096,8;5655,6)	10411,1(9888,5;11071)	7761,3(7446,2;8148,5)
60 a 64	7582,7(7157,8;8013,5)	14377,2(13743,6;15250,1)	10724,5(10227,3;11241,4)
65 a 69	10440,7(9551,5;10999,4)	18468,3(17560,5;19567,3)	14100,1(13252,9;14825,6)
70 a 74	13847,1(12713,4;14787,4)	22435,5(21179,5;23838,4)	17700,4(16549,4;18565)
75 a 79	18640,7(16866,6;19781,3)	27676,6(25780;29487)	22551,3(20604,1;23670,6)
80 a 84	24129(20672,6;26360,9)	32268,2(28825,4;34642,2)	27434,4(24030;29537,3)
85 a 89	30302,1(24656,6;33410)	37590,2(32959,5;40444,5)	33002,3(27682,6;35861)
90 a 94	36517,8(28368,6;41236)	39737,4(33252,6;43645,7)	37646(30075,8;42020,3)
95+	40754,9(30729,3;46726,8)	36987,3(29033,6;41740,1)	39576,6(30225,9;45134,3)
Todas as idades	2810,8(2594,7;2989,8)	4462,4(4215,9;4722,8)	3568,9(3343,4;3729,7)
Padronizadas por idade	3389,8(3126,3;3610,1)	4713,5(4462,7;4992,9)	4035,5(3789,2;4219,2)

Fonte: Dados derivados do Global Burden of Disease Collaborative Network. Global Burden of Disease (GBD) Cardiovascular Burden Estimates 1990 and 2021, Institute for Health Metrics and Evaluation, University of Washington. ^48^

•A distribuição da mortalidade por DNC é extremamente desigual, com 86% das mortes prematuras causadas por DNC antes dos 70 anos de idade encontradas nos países de renda média e baixa. Enquanto houve diminuição global nas taxas de incidência, as DCV permanecem a principal causa de mortalidade por DNC. Além disso, as mortes relacionadas ao diabetes aumentaram em todo o mundo e em todas as regiões nos últimos 30 anos. As variações anuais nas taxas de DALYs e de mortalidade por 100 mil variam entre as regiões com diferentes perfis de desenvolvimento. Isso reforça a diferença do impacto dos vários fatores nos desfechos de saúde.^54^

•

### Utilização e Custo da Atenção à Saúde

•No Brasil, de 2008 a 2021, as internações hospitalares por doenças cardiovasculares aumentaram até 2019 e diminuíram em 2020 e 2021, correspondendo a 89 mil internações hospitalares a menos em 2020 do que em 2019, uma redução de 12%. As internações por condições clínicas apresentaram tendência de diminuição nas últimas décadas, enquanto as relacionadas a procedimentos ou intervenções cirúrgicas aumentaram nesse período. ( **Tabelas 1-4 e 1-5** ).^49^ Doenças isquêmicas do coração, doenças cerebrovasculares, insuficiência cardíaca e arritmia foram responsáveis por 68% de todas as internações hospitalares por doenças cardiovasculares ( **Tabela 1-4** ).^49^

**Tabela 1-4:** – Número total de internações por procedimentos clínicos por doenças cardiovasculares no SUS por ano de competência, Brasil, 2008 a 2021

	2008	2009	2010	2011	2012	2013	2014	2015	2016	2017	2018	2019	2020	2021	Total
DAC	12.393	9.743	9.300	8.497	8.000	7.197	7.581	6.403	6.317	6.171	6.292	6.703	5.099	4.559	104.255
Doença Cerebrovascular	159.545	176.047	181.035	184.751	182.065	183.043	187.110	191.678	195.787	198.068	203.066	211.149	199.126	206.518	2.658.988
Doença Valvar	3.237	4.156	3.526	3.637	3.285	2.996	2.753	2.400	2.244	2.231	2.330	2.289	1.536	1.597	38.217
Fibrilação Atrial	29.034	28.174	28.382	28.583	28.760	28.268	29.799	29.754	29.889	30.265	30.958	32.753	26.764	25.283	406.666
Infarto Agudo do Miocárdio	47.358	50.987	55.513	58.194	59.562	58.552	62.809	66.647	70.441	71.835	74.569	80.614	76.444	81.143	914.668
Insuficiência Cardíaca	298.474	297.763	289.110	284.844	264.469	254.285	243.913	240.832	236.358	230.297	222.394	222.620	187.770	181.441	3.454.570
Cardiomiopatias	2.092	2.363	2.459	2.302	2.357	2.293	2.370	2.230	2.250	1.997	2.251	2.390	1.899	1.868	31.121
Síndrome Coronariana Aguda	63.300	68.833	72.912	71.523	75.734	73.432	76.945	72.686	70.430	70.713	68.413	70.204	56.583	52.827	964.535
Total	615.433	638.066	642.237	642.331	624.232	610.,066	613.280	612.630	613.716	611.577	610.273	628.722	555.221	555.236	8.573.020

Fonte: Ministério da Saúde do Brasil – Sistema de Informações Hospitalares do Sistema Único de Saúde (SIH/SUS). ^49^

•O número de admissões hospitalares por DCV depois de 2020 apresentou significativa redução em todas as regiões brasileiras, como mostra a **Figura 1-6** . A região Sudeste apresentou o mais alto número de admissões hospitalares por DCV, com diminuição de 77 mil admissões em 2020, uma redução de 15%. Todas as regiões apresentaram redução nas admissões hospitalares por DCV em 2020 e 2021.^49^

**Figura 1-6:**
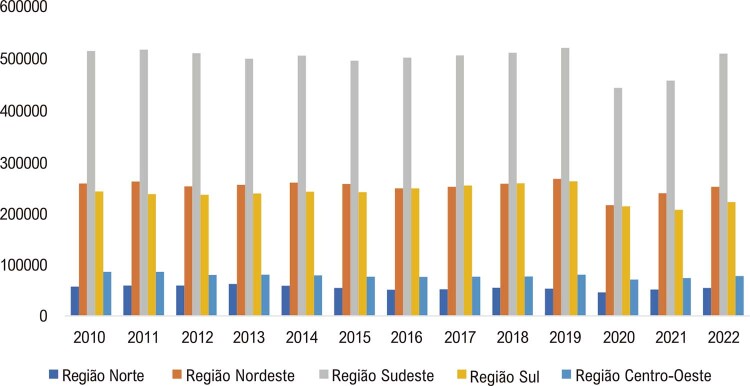
– Admissões hospitalares por doença cardiovascular de acordo com as regiões brasileiras, 2010-2022. Dados do Ministério da Saúde do Brasil – Sistema de Informações Hospitalares do Sistema Único de Saúde (SIH/SUS). ^49^

•Nos anos iniciais da pandemia de COVID-19, 2020 e 2021, houve significativa redução das admissões hospitalares para todos os tipos de doenças do sistema circulatório ( **Figura 1-6** ).^49^ Alguns fatores devem ser considerados. Hipóteses, tais como aumento do número de mortes cardiovasculares fora do hospital, redução da busca por assistência médica e admissão hospitalar com concomitância de DCV e COVID-19 registrada como diagnóstico primário, poderiam explicar tais dados.

•As admissões hospitalares por Infarto agudo do miocárdio aumentaram 50% de 2010 a 2021. Foi a causa de 61 mil admissões hospitalares em 2010 e 93 mil em 2022. As hospitalizações por infarto agudo do miocárdio apresentaram leve diminuição em 2020, mas aumentaram em 2021( **Table 1-4)** .^49^ As angioplastias primárias aumentaram significativamente nos últimos 10 anos no SUS, tanto em números absolutos como ajustados pela população. **( Tabela 1-5 )** .^49^

**Tabela 1-5:** – Número total de internações por procedimentos cirúrgicos por doenças cardiovasculares no SUS por ano de competência, Brasil, 2008 a 2021

	2008	2009	2010	2011	2012	2013	2014	2015	2016	2017	2018	2019	2020	2021	Total
Ablação de Fibrilação atrial	68	72	90	85	123	139	143	161	124	120	125	163	120	161	1.694
Angioplastia coronariana	38.635	45.648	49.492	55.931	60.959	63.838	66.492	66.550	69.802	73.971	78.575	85.518	77.846	80.190	913.447
Revascularização do miocárdio	20.515	22.077	21.225	23.187	23.900	23.249	22.997	22.559	22.248	21.474	20.674	21.018	16.554	15.932	297.609
Cirurgia valvar	12.201	12.664	12.169	13.181	13.435	13.067	12.993	12.624	12.432	12.277	12.088	12.771	9.198	8.759	169.859
Angioplastia Primária	7.648	6.362	6.262	6.033	5.865	6.055	7.135	8.524	10.195	10.774	10.811	11.099	11.253	11.795	119.811
Cardiomiopatia	15	43	13	21	28	23	20	18	32	29	26	24	18	14	324
Outras doenças valvares	451	477	445	486	456	527	515	513	399	427	391	450	399	470	6.406
Valvuloplastia mitral	477	551	478	473	403	431	408	341	206	236	200	195	129	159	4.687
Total	80.010	87.894	90.174	99.397	105.169	107.329	110.703	111.290	115.438	119.308	122.890	131.238	115.517	117.480	1.513.837

Fonte: Ministério da Saúde do Brasil – Sistema de Informações Hospitalares do Sistema Único de Saúde (SIH/SUS). ^49^

•Nos últimos 14 anos, no Brasil, houve significativa redução nas hospitalizações por insuficiência cardíaca e aumento nas hospitalizações anuais por infarto agudo do miocárdio e doenças cerebrovasculares, enquanto os outros grupos de procedimentos clínicos tenderam à estabilidade ( **Table 1-4** ).^49^

•Quanto às abordagens cirúrgicas nos mesmos anos, houve grande aumento no número anual de angioplastias coronarianas e tendência à estabilidade no número dos outros procedimentos cirúrgicos ( **Figura 1-7** ).^49^

**Figura 1-7:**
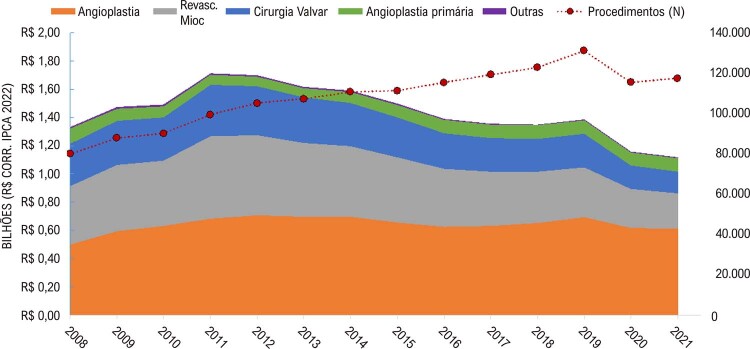
– Valor total pago e número de procedimentos cirúrgicos por ano no SUS, Brasil, de 2008 a 2021. IPCA, Índice Nacional de Preços ao Consumidor Amplo. Dados do Ministério da Saúde do Brasil – Sistema de Informações Hospitalares do Sistema Único de Saúde (SIH/SUS). ^49^

•De 2008 a 2010, o SUS pagou por internações clínicas em média R$ 1,4 bilhão por ano; de 2017 a 2019, em média, R$ 1,1 bilhão, e de 2020 a 2021, foram 993 milhões por ano ( **Tabela 1-6** ). Os procedimentos cirúrgicos receberam US$ 1,4 bilhão, US$ 1,3 bilhão e US$ 1,1 bilhão, respectivamente. Embora o número de internações por doenças cardiovasculares mais do que duplicaram durante esta última década, houve uma redução significativa no reembolso por doenças cardiovasculares ( **Figura 1-7 e Tabela 1-7** ).^49^

**Tabela 1-6:** – Valor pago (em reais) ajustado de internações clínicas por doenças cardiovasculares por ano de competência, Brasil, 2008 a 2021

	2008	2009	2010	2011	2012	2013	2014	2015	2016	2017	2018	2019	2020	2021
DAC	17.473.695	14.515.852	13.375.022	11.971.544	10.232.200	8.917.122	9.968.291	7.750.106	7.256.573	7.090.575	7.061.618	7.770.301	6.019.912	5.239.722
Doença Cerebrovascular	318.306.994	398.722.244	403.265.605	393.470.650	393.157.770	387.626.894	389.295.216	380.584.520	359.325.511	348.788.550	356.413.503	364.584.254	348.288.096	350.863.859
Doença Valvar	2.357.047	3.362.521	2.919.687	3.077.011	2.714.235	2.565.230	2.541.501	2.521.358	2.282.181	2.151.729	2.543.861	2.399.303	1.755.725	1.613.260
Fibrilação Atrial	30.900.437	36.806.646	37.599.982	36.117.396	36.633.135	33.928.480	36.315.140	35.172.431	32.597.899	33.400.513	33.577.340	34.490.032	29.215.902	27.237.974
Infarto Agudo do Miocárdio	145.683.996	178.378.823	188.575.764	186.393.207	188.636.874	180.519.838	191.841.885	194.066.706	183.785.925	174.865.678	178.459.141	181.336.606	168.242.806	167.576.265
Insuficiência Cardíaca	610.079.105	683.083.024	665.130.816	632.953.807	571.113.086	546.610.902	523.214.027	508.987.795	470.752.097	444.528.797	434.269.861	431.135.829	369.639.100	356.801.947
Cardiomiopatias	2.885.134	4.023.341	4.347.884	3.638.104	3.795.299	3.910.733	4.325.566	4.043.157	4.175.497	3.276.496	3.883.812	3.808.453	3.102.123	3.172.783
Síndrome Coronariana Aguda	100.179.911	122.550.378	131.057.092	125.609.896	135.250.270	126.783.127	134.127.142	123.767.713	109.233.624	105.187.837	99.639.789	97.394.487	76.776.561	69.697.477
Total	1.227.866.320	1.441.442.829	1.446.271.852	1.393.231.616	1.341.532.868	1.290.862.326	1.291.628.769	1.256.893.786	1.169.409.306	1.119.290.174	1.115.848.924	1.122.919.263	1.003.040.225	982.203.288

Fonte: Ministério da Saúde do Brasil – Sistema de Informações Hospitalares do Sistema Único de Saúde (SIH/SUS). ^49^

**Tabela 1-7:** – Valor pago (em reais) atualizado por procedimentos clínicos para doenças cardiovasculares por ano de competência, Brasil, 2008 a 2021

	2008	2009	2010	2011	2012	2013	2014	2015	2016	2017	2018	2019	2020	2021
Ablação de Fibrilação atrial	807.686	797.310	955.644	875.098	1.240.832	1.339.634	1.236.360	1.366.392	963.242	885.222	911.289	1.154.269	863.980	1.045.998
Angioplastia coronariana	471.716.260	564.185.839	599.670.065	647.278.961	669.078.423	660.801.300	659.753.883	621.249.372	590.665.315	598.141.041	617.339.631	655.318.815	586.608.702	579.077.077
Revascularização do miocárdio	394.422.557	441.323.860	435.052.405	551.289.001	535.610.518	493.649.155	473.022.182	436.663.808	389.825.203	361.649.521	342.491.428	334.232.757	258.310.185	235.521.656
Cirurgia valvar	282.216.914	297.658.304	288.805.944	343.030.523	329.575.746	303.391.954	288.907.966	266.567.822	238.830.404	225.744.421	221.078.457	224.844.776	158.038.290	144.300.688
Angioplastia Primária	101.427.047	80.162.895	75.278.257	68.136.360	63.921.172	63.354.779	73.608.840	84.578.572	90.611.405	91.797.091	91.413.951	89.883.845	88.171.102	89.814.989
Cardiomiopatia	378.650	1.152.515	390.162	624.198	784.238	601.155	490.184	449.706	717.795	579.036	530.431	485.825	359.755	249.798
Outras doenças valvares	3.403.160	3.515.486	3.483.819	3.674.623	3.363.929	3.485.710	3.414.332	3.144.844	2.171.741	2.420.709	2.103.416	2.351.343	2.088.988	2.393.891
Valvuloplastia mitral	6.980.121	7.585.874	6.383.913	6.181.863	4.888.469	5.046.819	4.505.648	3.607.236	1.876.617	2.205.110	1.819.664	1.716.096	1.062.664	1.137.898
Total	1.261.352.395	1.396.384.093	1.410.020.209	1.621.090.628	1.608.463.326	1.531.670.505	1.504.939.395	1.417.627.751	1.315.661.722	1.283.422.152	1.277.688.268	1.309.987.725	1.095.503.666	1.053.541.996

Fonte: Ministério da Saúde do Brasil – Sistema de Informações Hospitalares do Sistema Único de Saúde (SIH/SUS). ^49^

•Embora o número de internações e procedimentos intervencionistas tenha aumentado na última década, os pagamentos de procedimentos cirúrgicos cardiovasculares pelo SUS nos últimos anos da série diminuíram, principalmente em procedimentos mais complexos, como revascularização do miocárdio e cirurgias de troca valvar, conforme mostrado na **Figura 1-7** .^49^

•Brant *et al* ., analisando mortalidade por DCV durante a pandemia em Belo Horizonte, observaram maior ocorrência de mortes domiciliares por DCV em paralelo a menores taxas de hospitalização. Esses autores concluíram que a “assistência para DCV foi comprometida durante a pandemia de COVID-19, que afetou de forma mais significativa os indivíduos mais idosos e os socialmente vulneráveis, exacerbando as desigualdades na saúde em Belo Horizonte”.^66^

•As instituições públicas atendem mais de 70% dos brasileiros, mas os sistemas de saúde suplementar respondem por quase a mesma parcela dos gastos com saúde. Infelizmente, existem poucos estudos publicados ou bases de dados abertas para calcular a carga económica adicional das doenças cardiovasculares deste segmento. Estimativas globais baseadas na prevalência de doenças cardiovasculares sugerem que o custo direto e indireto das DCV é superior a 4 a 5 vezes o custo direto para o SUS, aproximadamente 10 bilhões de dólares.^67^

### Perspectiva

•O SIM, implementado em 1975, é uma ferramenta essencial para monitorar as estatísticas de mortalidade no Brasil, pois o registro de todas as mortes é obrigatório nas UF, sendo que, em 2017, a cobertura do território nacional foi de 98%, menor na região Norte do que na Sul. O índice de usabilidade da OMS para o SIM é alto, indicando que a informação proveniente do SIM é útil para análise, embora algumas correções de dados possam ser necessárias na série temporal.^68^

•A região Nordeste apresenta a menor cobertura do SIM, ainda inferior a 95%.^54^ Embora o SIM tenha melhorado através de projetos específicos do Ministério da Saúde, ainda persistem problemas, como códigos mal definidos (cerca de 6%), ‘códigos *garbage* ’ (15%) e classificação errada das causas de morte, que geram vieses que podem comprometer a métrica apresentada.^49^

•As questões relacionadas aos ‘códigos *garbage* ’ resultam da má qualidade de algumas declarações de óbito, além daquelas associadas ao acesso limitado aos serviços de saúde, dificuldades no acesso a diagnóstico ou simplesmente má qualidade dos prontuários médicos, dificultando a recuperação da informação.^49^ Quanto a isso, a melhor maneira de garantir a acurácia da causa básica de morte é através do treinamento dos médicos em todo o país para a adequada certificação do óbito.

•Vale mencionar que, devido à falta de dados de incidência primária (coortes) no Brasil, há necessidade de pesquisa que permita compreender como enfrentar a DCV nos estados e nas populações com baixos índices socioeconômicos.

•Devido à redução na tendência de queda da mortalidade por DCV padronizada por idade nos últimos 5 anos, novas estratégias para enfrentar a mortalidade por DCV devem ser estudadas. É fundamental que se compreendam os motivos para tal redução para que se implementem políticas efetivas, em particular ante o envelhecimento da população, o que vai aumentar o número de indivíduos com DCV no país.

•O impacto da COVID-19 e da COVID-19 a longo prazo nas mortes e na carga por DCV não foi completamente estudado e há muitos aspectos a serem investigados. O efeito no sistema de saúde e nas estatísticas de saúde está registrado, mas as incertezas sobre as tendências futuras são muitas. No que diz respeito à saúde cardiovascular, é essencial compreender o efeito a longo prazo da infecção por COVID-19 nas pessoas com fatores de risco e DCV.

## CAPÍTULO 2 – AVC (DOENÇAS CEREBROVASCULARES)

### CID-9 430 a 438; CID-10 I60 a I69.

**Ver Tabela 2-1 e Figuras 2-1 e 2-2**

**Table:** Abreviaturas usadas no capítulo 2

IECA/BRA	Inibidor da enzima de conversão da angiotensina/bloqueador do receptor de angiotensina
AVC	Acidente Vascular Cerebral
BRIDGE-Stroke	Estudo *Brazilian Intervention to Increase Evidence Usage - Stroke*
IC	Intervalo de Confiança
DCV	Doenças Cardiovasculares
DALYs	Anos de vida perdidos ajustados por incapacidade (do inglês, * Disability-Adjusted Life-Years* )
CID-10	Classificação Estatística Internacional de Doenças e Problemas Relacionados à Saúde, 10^a^ Revisão
UTI	Unidade de Terapia Intensiva
IMPACT-AF	Estudo *Improve Treatment with Anticoagulants in Patients with Atrial Fibrillation*
IRR	Razão da taxa de incidência (do inglês, *Incidence Rate Ratio* )
MAPS	Estudo *Matão Preventing Stroke*
OR	Odds Ratio
PURE	Estudo *Prospective Urban Rural Epidemiological Study*
RR	Risco Relativo
SIM	Sistema de Informações sobre Mortalidade
YLDs	Anos vividos com incapacidade (do inglês, *Years Lived with Disability* )
YLLs	Anos potenciais de vida perdidos (do inglês, * Years of Life Lost* )

### Introdução

•O AVC representa uma das principais causas de morbimortalidade no Brasil. Nos últimos anos, uma crescente representação de evidência gerada no Brasil vem contribuindo para a melhor compreensão da carga do AVC. Ademais, a análise das estatísticas de AVC representa um avanço no desenvolvimento do cuidado baseado em evidência.

### Prevalência

•As estimativas de prevalência de AVC podem diferir levemente entre os estudos, pois cada um seleciona e recruta uma amostra de participantes para representar sua população-alvo (estado, região ou país). Em estudos de base comunitária na cidade de São Paulo, a taxa de prevalência ajustada para idade para homens foi 4,6% (IC 95%, 3,5 - 5,7) e para mulheres, 6,5% (IC 95%, 5,5 - 7,5)^69^ em 2011, enquanto, em outro estudo na mesma cidade, de 3.577 indivíduos, 244 (6,82%) sobreviventes de AVC foram identificados.^70^ Na cidade de Coari, os autores relataram prevalência bruta de AVC de 6,3% nas áreas rurais e de 3,7% nas urbanas.^71^ Em uma pesquisa epidemiológica de base comunitária com representatividade nacional (Pesquisa Nacional de Saúde - 2013), Bensenor *et al.* estimaram 2.231.000 casos de AVC, correspondendo a uma prevalência de 1,6% e 1,4% em homens e mulheres, respectivamente.^72^

### Incidência

•No estudo do registro de AVC de Matão, entre os períodos 2003-2004 e 2015-2016, a incidência ajustada por idade diminuiu em 39% (IRR 0,61; IC 95%, 0,46–0,79) e a mortalidade, em 50% (IRR 0,50; IC 95%, 0,31– 0,94), enquanto 7% apresentaram recorrência do AVC.^73^

### Mortalidade

•Dados do Grupo GBD Brasil mostram que a taxa de mortalidade padronizada por idade de AVC isquêmico por 100 mil em 2021 foi 31,7 (28 a 33,9) ( **Tabela 2-1 e Figura 2-1** ). A maior mortalidade foi observada no Maranhão, 49,8 (42,8 a 56), e a menor, no Rio Grande do Norte, 25 (21,1 a 27,9).

•As tendências das taxas de mortalidade e DALYs para doença cerebrovascular (CID-10: I-60-69), segundo o GBD Brasil 2015, mostraram que a redução anual na taxa de mortalidade ajustada para idade, para ambos os sexos, desacelerou entre 2005 e 2015 quando comparada ao período anterior (1990 a 2005).

•Na cidade de São Paulo, de 1996 a 2011, as taxas de mortalidade ajustadas por idade por doenças cerebrovasculares diminuíram 46,6% nos homens e 47,8% nas mulheres. Para ambos os sexos e grupo etário de 35-74 anos, a diminuição nas taxas ajustadas por idade foi mais pronunciada entre os residentes da área de maior renda em comparação àqueles da área de menor renda.^74^ Um estudo usando dados do SIM, de 1990 a 2012, mostrou uma variação de -48,05% no coeficiente de mortalidade por AVC.^75^ No estudo MAPS, entre 2003-2004 e 2015-2016, a mortalidade aumentou em 50% (IRR 0,50; IC 95%, 0,31-0,94). A taxa de letalidade de 1 ano foi 26% e aproximadamente 56% dos pacientes foram funcionalmente independentes,^73 , 76^ enquanto 7% tiveram um AVC recorrente.^73^

•Em um estudo conduzido no Paraná, a taxa de mortalidade específica para idade e sexo relacionada a AVC entre 2007 e 2016 aumentou de 138 para 163 por 100 mil habitantes, sendo que indivíduos com idade superior a 79 anos apresentaram o maior aumento. Entretanto, as taxas de mortalidade diminuíram nos grupos etários de 34-44 anos e de 44-54 anos.^77^

### Carga Global das Doenças Cerebrovasculares

#### YLL

•Um estudo conduzido na região sudeste do Brasil em 2019 usando dados secundários do Sistema de Saúde Pública Brasileiro e do Instituto Brasileiro de Geografia e Estatística mostrou um total estimado de 713.132 DALYs, com 80% atribuídos a YLDs e 20%, a YLLs. Ao se ajustar para o tamanho da população, observou-se maior impacto no sexo masculino em Minas Gerais, particularmente no grupo etário de 70-79 anos.^78^

## DALY

•Dados do Grupo GBD Brasil mostram que a taxa de DALYs padronizada por idade por 100 mil por AVC isquêmico em 2021 foi 511,4 (470,6 a 542,5).^11^ A mais alta taxa de DALYs foi observada no Maranhão, 759,8 (674,3 a 858), e a mais baixa, no Rio Grande do Norte, 407,1 (359,5 a 449,7) ( **Tabela 2-1 e Figura 2-2** ).

**Figura 2-2:**
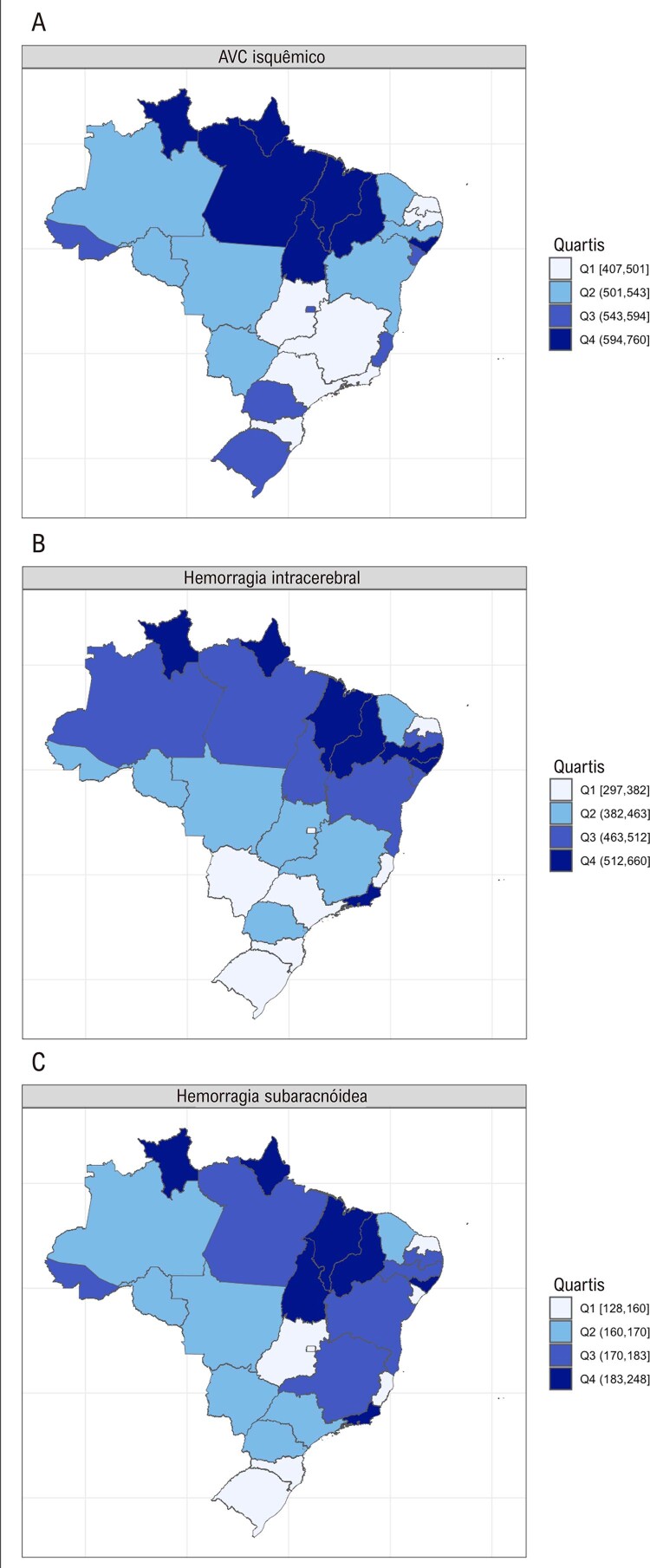
– Taxa de DALYs padronizada por idade por AVC isquêmico (A), por hemorragia intracerebral (B) e por hemorragia subaracnóidea (C) por 100 mil habitantes, 2021. Dados derivados das estimativas do Estudo Global Burden of Disease 2021. ^48^

## Complicações e Doenças Associadas

•Avaliações da pesquisa epidemiológica de base comunitária (Pesquisa Nacional de Saúde-2013) mostraram que a prevalência de incapacidade pós-AVC foi de 29,5% para os homens e 21,5% para as mulheres, com apenas 0,27% dos indivíduos submetidos a fisioterapia para AVC.^79^

•Um estudo transversal de base populacional foi conduzido no Brasil usando dados do Estudo Longitudinal da Saúde dos Idosos Brasileiros 2015-2016. Incluiu 536 indivíduos com idade a partir de 50 anos que haviam tido um AVC.^80^ A prevalência de AVC foi de 5,3% entre aqueles com idade de 50 anos ou mais, aumentando para 8,0% entre aqueles com idade de 75 anos ou mais, com diferenças entre os sexos. Os fatores associados com independência nas atividades cotidianas incluíram velocidade de marcha, atividade física e uso de dispositivos para caminhar. Observou-se significativa interação entre velocidade de marcha, adaptações no domicílio e realização de atividades da vida cotidiana. A probabilidade de independência foi maior entre aqueles que caminhavam mais rápido (> 0,8 m/s) e tinham adaptações no domicílio. Maior velocidade de marcha combinada a adaptações no domicílio emergiu como fatores primários associados com independência no longo prazo após um AVC.

## Qualidade do Cuidado

•Quanto às hospitalizações por AVC, o estudo de Brant *et al* . mostrou redução de 5,1% (RRi 0,949; IC 95%, 0,947-0,952) durante a pandemia de COVID-19 (semana epidemiológica 10 a 21), correspondendo à menor redução relativa no número de hospitalizações no grupo das DCV. Portanto, ainda que AVC seja a segunda causa de hospitalização em números absolutos, a redução no número de hospitalizações foi menor do que para síndrome coronariana aguda. Em paralelo, as admissões em UTI por AVC mostraram aumentos total (RRi 1.154; IC 95%, 1.145-1.163) e proporcional (RRi 1.216; IC 95%, 1.210-1.222) maiores do que as admissões em UTI para as DCV juntas. Entretanto, embora um aumento na mortalidade proporcional tenha sido observado, especialmente na terceira fase da pandemia, esse foi menor do que para só DCV (RRi 1.053; IC 95%, 1.048-1.058) e não se recuperou até o final do período avaliado. Essa progressão de aumento nos números e na proporção de mortes hospitalares durante a pandemia deve ser ressaltada, principalmente porque a proporção de uso de UTI permaneceu maior do que a esperada no período avaliado.^15^

•Dantas *et al* . realizaram estudo para avaliar hospitalização relacionada a AVC no Sistema Brasileiro Unificado de Saúde de 2009 a 2016, quando o número de admissões aumentou de 131.122 para 146.950 e o número absoluto de mortes hospitalares aumentou de 28.731 para 31.937. Idade mais jovem e sexo masculino foram significativamente associados com sobrevida do paciente. As taxas anuais de hospitalização e de mortalidade hospitalar ajustadas para idade apresentaram redução de 11,8% e 12,6%, respectivamente, mas a taxa de letalidade aumentou para os pacientes acima de 70 anos de idade.^81^

•Um estudo avaliando fatores sociodemográficos relacionados à falta de assistência hospitalar para mortes por doença cerebrovascular no estado de São Paulo nos períodos de 1996-1998 e 2013-2015 mostrou que, de 127.319 indivíduos que morreram devido a AVC nos períodos mencionados, 19.362 (15,2%) não tiveram assistência hospitalar. No segundo período, o estudo mostrou que houve maior risco de morte sem assistência em indivíduos da raça amarela (RR = 1,48; IC 95%, 1,25-1,77) e menor, nos indivíduos da raça negra (RR = 0,86; IC 95%, 0,76-0,95), casados (RR = 0,70; IC 95%, 0,64-0,75) e residentes da cidade de São Paulo (RR = 0,92; IC 95%, 0,86-0,98).^82^

•A análise dos indicadores-chave de desempenho requeridos pelo Ministério da Saúde brasileiro para as unidades de AVC em dois centros de Curitiba e Botucatu mostrou que ambos admitiram mais de 80% dos pacientes em suas unidades de AVC.^83^

•Um ensaio randomizado de *cluster* avaliando o efeito de uma intervenção multifacetada de melhoria da qualidade sobre adesão a terapias baseadas em evidência no cuidado de pacientes com AVC isquêmico agudo e ataque isquêmico transitório (BRIDGE-Stroke) mostrou que 402 de 817 pacientes (49,2%) nos hospitais da intervenção receberam todos os tratamentos para os quais eram elegíveis em comparação a 203 de 807 pacientes (25,2%) de hospitais controle (OR, 2,59; IC 95%, 1,22-5,53; P= 0,01).^84^

•O estudo RESILIENT conduzido no sistema brasileiro de saúde pública para avaliar a segurança e a eficácia da trombectomia nesse sistema mostrou que esse procedimento, associado a cuidado padronizado, resultou em melhores desfechos funcionais aos 90 dias em comparação a apenas cuidado padronizado.^85^ A OR padrão para melhor distribuição dos escores na Escala de Rankin modificada foi 2,28, favorecendo trombectomia. A porcentagem de pacientes com déficit neurológico menor ou nenhum foi significativamente maior no grupo submetido a trombectomia.

## Custo

•Os custos relacionados a hospitalizações por AVC e ajustados para a inflação de 2008 a 2021 chegaram a US$ 1.195.975.877,98, variando de US$ 28.661.321,88, em 2008, a US$ 66.843.953,39, em 2021.

•Um estudo de custo-efetividade, avaliando trombolíticos no Brasil, reportou que, para o resultado de 1 ano e para homens, o custo do tratamento com rt-PA foi maior do que o custo do tratamento conservador, sendo o custo da medicação o principal responsável por isso. Parte desse custo adicional é compensado pelo mais baixo custo da reabilitação e menor perda de produtividade já nos dois primeiros anos, pois os pacientes tratados com rt-PA (alteplase) apresentaram menos sequelas do que aqueles que receberam tratamento conservador. Depois do segundo ano do AVC, para ambos os sexos, o tratamento com rt-PA, considerando custos diretos e indiretos, começou a apresentar menor custo se comparado ao tratamento conservador. A partir desse ponto, o custo adicional da medicação começa a ser mais do que compensado pelo maior número de perdas menores de produtividade e menores custos da seguridade social e reabilitação do paciente.^86^

•A análise de custo-utilidade do ensaio RESILIENT comparando trombectomia mecânica associada a cuidado médico padrão com apenas cuidado médico padrão mostrou que os custos incrementais e o número de anos com qualidade de vida ganhos foram estimados em Int$ 7.440 e 1,04, respectivamente, resultando em uma razão custo-efetividade incremental de Int$ 7.153 por ano de vida ajustado para qualidade. A despeito dos maiores custos iniciais, os benefícios a longo prazo da trombectomia mecânica justificaram sua incorporação nos centros de AVC do sistema brasileiro de saúde pública, devido à sua provável custo-efetividade.^87^

## Fatores de Risco e Prevenção

•Dados do estudo PURE, examinando taxas e preditores do uso de medicações de prevenção secundária baseada em evidência, mostraram que um menor número de pacientes com AVC recebeu antiagregantes plaquetários (24,3%), IECA/BRA (37,6%) e estatinas (9,8%) em comparação a pacientes com doença cardíaca crônica (30,1%, 36,0% e 18,0%, respectivamente). Além disso, nenhuma medicação foi utilizada em 30% dos pacientes brasileiros com AVC.^88^

•No estudo IMPACT-AF, um ensaio randomizado em *cluster* para melhorar o tratamento com anticoagulantes de pacientes com fibrilação atrial, 91% dos pacientes do Brasil usavam anticoagulação oral na linha de base e 27%, os novos anticoagulantes orais. De todos os pacientes usando antagonistas da vitamina K no Brasil, 40,3% apresentavam valores do índice internacional normalizado entre 2 e 3 antes da consulta na linha de base.^89^

•Utilizando dados do Estudo GBD, Silva *et al* . avaliaram o impacto dos baixos níveis de atividade física na mortalidade por AVC no Brasil de 1990 a 2019.^24^ Houve redução de 44,0% (homens) e 52,0% (mulheres) nas taxas de mortalidade por AVC padronizadas por idade atribuída aos baixos níveis de atividade física. O estudo sugere que níveis mais altos de atividade física possam ter impedido aproximadamente 6,1% (homens) e 7,3% (mulheres) das mortes por AVC em 2019.^90^

•Em um estudo transversal analisando dados anonimizados coletados de rotina por profissionais da saúde comunitária no Brasil entre maio de 2016 e setembro de 2021, de uma população de 2.133.900 indivíduos na base de dados, 11.628 haviam tido infarto do miocárdio prévio (33,1%) ou AVC (n = 25.925; 73,9%). Apenas 6,7% (2.346) e 0,6% (212) relataram uso de estatinas e de altas doses de estatinas, respectivamente. Idade superior a 60 anos, residir na região sul, diagnósticos prévios de infarto do miocárdio, insuficiência cardíaca, diabetes, dislipidemia e doença renal crônica, além do uso de anti-hipertensivos foram associados ao uso de estatina.^91^

## Conscientização e Tratamento

•Recentemente, tem havido várias iniciativas para promover a conscientização do público sobre AVC no Brasil, em especial através de campanhas anuais por ocasião do Dia Mundial do AVC (29 de outubro) conduzidas pela Organização Mundial do AVC.^92^ Apesar desses esforços, vários estudos mostraram uma falta alarmante de conhecimento sobre os fatores de risco e tratamento do AVC, assim como de reconhecimento dos sintomas de AVC como uma emergência médica. Em um estudo, Pontes-Neto *et al* . mostraram que 32% dos entrevistados não reconheceram qualquer sinal de alarme de AVC, apenas 34,6% dos entrevistados responderam corretamente quando perguntados sobre o número de telefone de emergência no Brasil (#192) e apenas 51,4% dos entrevistados relataram que chamariam uma ambulância para um familiar com sintomas de AVC.^93^

•Em estudo conduzido na cidade de Caxias do Sul, baixa renda e baixo nível educacional foram preditores independentes da incapacidade de reconhecer que AVC afeta o cérebro.^94^ Em outro estudo da cidade de Santa Maria, Rio Grande do Sul, onde 33% dos indivíduos identificaram corretamente o acrônimo “AVC”, cerca de 30% localizaram AVC incorretamente no coração.^95^

•Um estudo conduzido em São Paulo investigou estudantes de diferentes níveis educacionais, incluindo 1.187 do ensino fundamental (idade média, 13 anos; experiência anterior: 14%; 51% mulheres), 806 do ensino médio (idade média, 17 anos; experiência anterior: 13%; 47% mulheres) e 1.961 universitários (idade média, 22 anos; experiência anterior: 9%; 66% mulheres). A conscientização sobre AVC e o conhecimento dos fatores de risco e sinais de alerta variaram de 42% a 66% dos estudantes. Menos de 52% dos estudantes associaram AVC com fatores de risco, como hipercolesterolemia, tabagismo, diabetes e hipertensão. Quando perguntados, 62% a 65% dos estudantes reconheceram fraqueza nos braços, paralisia facial e alteração da fala como sinais de alerta de AVC, mas apenas 43% identificaram cefaleia aguda. Importante notar que 67% dos estudantes conheciam o número do serviço médico de emergência. Nível educacional mais alto, experiência anterior e sexo feminino foram associados a maior escore para identificação dos fatores de risco de AVC (OR = 1,28; IC 95%, 1,10‒1,48; OR = 2,12; IC 95%, 1,87‒2,40; OR = 1,46; IC 95%, 1,16‒1,83; respectivamente) e sinais/sintomas de alerta (OR = 2,22; IC 95%, 1,89‒2,60; OR = 3,30; IC 95%, 2,81‒3,87; OR = 2,04; IC 95%, 1,58‒2,63; respectivamente).^96^

•Em estudo transversal conduzido em escolas do ensino médio no nordeste do Brasil, Rodrigues *et al* . avaliaram o conhecimento sobre AVC entre os alunos.^97^ Esses autores descobriram que 80% dos alunos não tinham o conhecimento mínimo de como agir em uma situação de AVC, enquanto apenas 10% tinham o conhecimento ideal. Os do sexo masculino conheciam menos os fatores de risco e sinais e sintomas de AVC. Os alunos com escolaridade superior a 10 anos e aqueles com 18 anos de idade mostraram maior conhecimento em certos aspectos. Esse estudo ressalta o déficit de conhecimento entre os alunos do ensino médio quanto ao reconhecimento do AVC e a ativação dos serviços médicos de emergência.

## Impacto da Pandemia de COVID-19

•Quanto às hospitalizações por AVC, houve redução de 5,1% no período, correspondendo à mais baixa redução relativa no número de hospitalizações no grupo das DCV. Embora o AVC seja a segunda causa de hospitalização em números absolutos, a redução no número absoluto de hospitalizações foi menor do que a da síndrome coronariana aguda. Em paralelo, houve maior aumento absoluto (15,4% vs. 9,0%) e proporcional (21,6% vs. 8,6%) nas admissões em UTI por AVC em comparação àquelas por DCV combinadas. Entretanto, a despeito do aumento observado na mortalidade proporcional, especialmente na terceira fase da pandemia, ele foi menor do que aquele para DCV apenas (5,3% vs. 14,4%) e não houve recuperação até o final do período avaliado. Tal progressão do aumento nos números e na proporção de mortes hospitalares durante a pandemia merece ser enfatizada, principalmente porque a proporção de uso de UTI permaneceu superior à esperada no período avaliado. Em conjunto, tais achados sugerem que: a) possivelmente devido a sintomas clínicos mais marcantes e exuberantes na fase aguda do AVC, uma maior proporção de pacientes manteve a tendência de buscar as unidades de urgência e emergência, em comparação àqueles com síndrome coronariana aguda, uma condição com mais sintomas inespecíficos, em especial em subgrupos, como mulheres, idosos e indivíduos com diabetes. Isso pode ter contribuído para a menor redução nas hospitalizações durante a pandemia; e b) indivíduos com AVC foram hospitalizados com quadros clínicos mais graves ou tiveram resultados piores nos tratamentos, possivelmente devido ao comprometimento das linhas de cuidado de saúde.^15^

•Quanto à mortalidade por AVC, aquele mesmo estudo mostrou que o AVC foi a causa específica com a menor redução de mortalidade no país e não houve variação nas regiões norte e centro-oeste. Tais achados estão em concordância com a redução relativamente baixa nas hospitalizações, sugerindo um menor impacto real da pandemia nas doenças cerebrovasculares. O fato de os sintomas clínicos serem diferentes daqueles da COVID-19 pode ter permitido um diagnóstico mais preciso. Além disso, a menor relação direta entre o tempo de início do tratamento e a mortalidade no AVC pode ter permitido maior tempo para o paciente reconhecer os sintomas e chegar ao hospital, diferentemente do que ocorre na síndrome coronariana aguda, em que a maioria das mortes ocorre nas primeiras horas após o evento.^15^

## Perspectivas

•O portfólio de pesquisa brasileira em neurologia vascular evoluiu muito nos últimos anos, como ilustram a fundação da Rede Brasil AVC e a crescente evidência de geração direcionada a uma variedade de indivíduos e conduzida por diferentes grupos. Mas há ainda várias oportunidades de desenvolvimento.

•Os estudos comunitários mais expressivos sobre prevalência e incidência de AVC são provenientes principalmente de duas cidades. Embora representem uma importante realização para a epidemiologia do AVC, avaliação mais abrangente se faz necessária, compreendendo a representação de todas as regiões geográficas brasileiras, das diversas culturas e dos diferentes níveis de renda. Como o AVC continua a ser um importante problema de saúde no Brasil, avançar na geração de evidência para novas intervenções efetivas e seguras no AVC é fundamental. Portanto, modelos inovadores, como pesquisa virtual e descentralizada com o paciente em posição central, assim como o uso de dados do mundo real são valiosas estratégias propostas na busca de um ciclo mais efetivo de aprendizado-realização.^98 , 99^

## CAPÍTULO 3 – DOENÇA ARTERIAL CORONARIANA AGUDA E CRÔNICA

### CID-9-CM 410 a 414; CID-10 I10 a I25

#### Ver Tabelas 3-1 a 3-3

**Table:** Abreviaturas Usadas no Capítulo 3

ACCEPT	Brazilian Registry of Clinical Practice in Acute Coronary Syndromes
B-CaRe:QCO	Brasilia Cardiovascular Registry for Quality of Care and Outcomes
BRACE	Brazilian Registry in Acute Coronary Syndromes
BYPASS	Brazilian Registry of Adult Patients Undergoing Cardiovascular Surgery
CAC	Calcificação de Artéria Coronária
CRVM	Cirurgia de Revascularização do Miocárdio
DAC	Doença Arterial Coronariana
DALY	Anos de vida perdidos ajustados por incapacidade (do inglês, * Disability-Adjusted Life-Years* )
DATASUS	Base de dados do Departamento de Informática do Sistema Único de Saúde
DCV	Doença Cardiovascular
DIC	Doença Isquêmica do Coração
ERICO	Strategy of Registry of Acute Coronary Syndrome
GBD	Global Burden of Disease
IAM	Infarto Agudo do Miocárdio
IAMCSST	Infarto Agudo do Miocárdio com Supradesnível do Segmento ST
IAMSSST	Infarto Agudo do Miocárdio sem Supradesnível do Segmento ST
IC	Intervalo de Confiança
ICP	Intervenção Coronariana Percutânea
II	Intervalo de Incerteza
IIQ	Intervalo Interquartil
MASS	Medicine, Angioplasty, or Surgery Study
OR	Odds Ratio
PNS	Pesquisa Nacional de Saúde
REPLICCAR-I	Registro Paulista de Cirurgia Cardiovascular I
REPLICCAR-II	Registro Paulista de Cirurgia Cardiovascular II
RR	Risk Ratio
SAMU	Serviço de Atendimento Médico de Urgência
SCA	Síndrome Coronariana Aguda
SCC	Síndrome Coronariana Crônica
SIH	Sistema de Informações Hospitalares
SIM	Sistema de Informação sobre Mortalidade
SUS	Sistema Único de Saúde
VICTIM	*Via Crucis* para o Tratamento do Infarto do Miocárdio
YLDs	Anos vividos com incapacidade (do inglês, *Years Lived with Disability* )
YLLs	Anos potenciais de vida perdidos (do inglês, * Years of Life Lost* )

## Panorama, Prevalência e Incidência

•A DAC, também conhecida como DIC, compreende um espectro de condições clínicas sintomáticas e assintomáticas tipicamente relacionadas à redução do fluxo sanguíneo para o músculo cardíaco. A causa mais comum é a doença aterosclerótica das coronárias, uma condição crônica de apresentação variável, que progride desde uma longa fase assintomática até angina estável, angina instável e IAM. A DAC é uma causa comum de insuficiência cardíaca, com fração de ejeção ventricular esquerda reduzida ou preservada, arritmias ventriculares e parada cardíaca súbita.

•A DAC foi a principal causa de morte no Brasil na última década, para homens e mulheres. Devido ao seu amplo espectro de apresentação clínica, a prevalência, a incidência e a mortalidade da DAC relatadas variam muito, dependendo da população e do contexto da atenção à saúde estudados.

•Estimativas da prevalência e da incidência de DAC em 2019 e série histórica de 1990 a 2019, de acordo com o estudo GBD, estão disponíveis na versão 2021 da Estatística Cardiovascular - Brasil.^4^

•Na avaliação basal da coorte ELSA-Brasil no período 2008-2010, a prevalência de DAC autorrelatada foi 2,7%. Essa coorte foi composta por mais de 15 mil servidores públicos com idade de 35-74 anos de seis cidades (Salvador, Vitória, Belo Horizonte, Rio de Janeiro, São Paulo e Porto Alegre). A prevalência foi mais alta entre os homens e indivíduos com nível socioeconômico mais baixo.^100^

•De acordo com a PNS 2019, um inquérito epidemiológico de base domiciliar e com representatividade nacional, as taxas de prevalência geral de angina leve (classe I) e moderada/grave (classe II) em adultos foram 8,1% (IC 95%, 7,8%-8,4%) e 4,5% (IC 95%, 4,2%-4,7%). A prevalência autorrelatada de angina foi mais alta em mulheres e inversamente proporcional aos anos de educação formal. Em comparação com indivíduos que se declararam brancos, a angina leve foi mais prevalente nos que se declararam negros, enquanto angina moderada/grave foi mais frequente nos que se declararam pardos.^101^ É importante ressaltar a ocorrência de maiores taxas de prevalência de angina nos inquéritos do que a prevalência de DAC obtida nos estudos epidemiológicos ou estatísticas nacionais. Avaliações autorreferidas de angina são muito sensíveis, mas não específicas para DAC, pois não requerem exames confirmatórios nem relatórios de saúde. Além disso, considerando-se a natureza assintomática da DAC, sua verdadeira epidemiologia pode estar sub-representada nas estatísticas nacionais.

•Em registros e em um estudo randomizado em *cluster* , envolvendo pacientes com SCA recrutados entre 2007 e 2014, as porcentagens de indivíduos com angina instável, IAMSSST e IAMCSST foram 15-30%, 31-36% e 36-54%, respectivamente.^102 - 105^

•A CAC foi avaliada em 3.616 indivíduos sem DCV ou diabetes *mellitus* da coorte multirracial do estudo ELSA-Brasil. Como esperado, a CAC aumentou com a idade e foi mais prevalente em homens do que em mulheres. Não se identificou CAC em 64% dos homens, 83% das mulheres, 93% dos jovens (35-44 anos) e 34% dos mais idosos (65-74 anos). Entretando, CAC >100 variou de 1,2% dos indivíduos com idade de 35-44 anos a 28% daqueles com 65-74 anos. CAC >400 não foi detectada naqueles com idade de 35-44 anos, mas foi em 12% daqueles com idade de 65-74 anos. Diferenças significativas na distribuição de CAC foram observadas de acordo com a raça. Por exemplo, entre os participantes com idade de 65-74 anos, a estimativa pontual para o percentil 50 de CAC foi 14 nos negros, 17 nos pardos e 32 nos brancos.^106^

## Mortalidade

•Estimativas do número de mortes e taxa de mortalidade bruta atribuível a DAC para 2019 e série histórica de 1990 a 2019, de acordo com o estudo GBD, estão disponíveis na versão 2021 da Estatística Cardiovascular - Brasil.^4^

•De acordo com as estimativas do GBD para o ano 2021, a taxa de mortalidade padronizada por idade por DAC foi 67,1 (II 95%, 60,9-71,0) por 100 mil habitantes no Brasil ( **Tabela 3-1** ). As taxas mais baixas estimadas foram observadas em Minas Gerais (50,7 [II 95%, 44,6-55,2]), Amazonas (54,1 [II 95%, 48,3-59,4]) e Bahia (56,0 [II 95%, 49,3-63,0]), enquanto as mais altas foram estimadas nos estados do Rio de Janeiro (81,3 [II 95%, 71,6-89,9]), Pernambuco (81,8 [II 95%, 72,4-89,0]) e Maranhão (89,0 [II 95%, 78,7-99,2]) por 100 mil habitantes ( **Tabela 3-1** ).^48^

**Tabela 3-1:** – Estimativas das taxas de mortalidade e DALYs padronizadas por idade (por 100 mil) por doença arterial coronariana no Brasil e suas unidades federativas em 2021

	Mortalidade	DALYs
Brasil	67,1 (60,9;71,0)	1432,5 (1345,3;1511,2)
Acre	67,3 (61,8;72,6)	1344,1 (1256,4;1451,6)
Alagoas	80,1 (71,0;90,7)	1731,7 (1547,7;1969,4)
Amapá	68,4 (59,8;77,5)	1381,4 (1223,7;1554,6)
Amazonas	54,1 (48,3;59,4)	1117,4 (998,0;1210,4)
Bahia	56,0 (49,3;63,0)	1216,2 (1066,7;1363,9)
Ceará	70,1 (60,2;79,7)	1434,2 (1220,1;1636,2)
Distrito Federal	61,4 (54,2;67,1)	1049,1 (939,5;1153,0)
Espírito Santo	65,6 (57,4;71,5)	1369,2 (1227,2;1499,2)
Goiás	65,3 (58,3;71,6)	1403,7 (1291,0;1548,4)
Maranhão	89,0 (78,7;99,2)	1850,9 (1620,1;2091,6)
Mato Grosso	60,3 (52,9;66,0)	1281,8 (1146,9;1410,7)
Mato Grosso do Sul	75,5 (68,5;84,1)	1610,5 (1470,1;1780,6)
Minas Gerais	50,7 (44,6;55,2)	1111,3 (983,7;1218,0)
Pará	67,9 (57,9;79,8)	1468,3 (1285,2;1728,1)
Paraíba	77,4 (69,9;83,6)	1658,3 (1508,4;1813,6)
Paraná	61,3 (55,7;66,5)	1277,9 (1181,0;1392,8)
Pernambuco	81,8 (72,4;89,0)	1752,5 (1582,0;1921,1)
Piauí	67,3 (58,6;76,5)	1442,9 (1267,8;1654,2)
Rio de Janeiro	81,3 (71,6;89,9)	1762,7 (1583,4;1935,2)
Rio Grande do Norte	71,6 (63,3;79,4)	1548,9 (1392,2;1715,0)
Rio Grande do Sul	58,6 (53,1;64,0)	1195,2 (1082,3;1308,8)
Rondônia	64,3 (56,1;72,0)	1309,8 (1151,0;1474,5)
Roraima	77,0 (66,2;89,1)	1503,6 (1277,2;1777,9)
Santa Catarina	61,2 (54,0;66,7)	1233,4 (1105,8;1349,6)
São Paulo	72,8 (64,4;80,2)	1568,9 (1431,1;1730,3)
Sergipe	57,3 (51,0;62,9)	1208,0 (1105,0;1315,9)
Tocantins	72,6 (63,3;82,5)	1531,7 (1339,5;1735,3)

Fonte: Dados derivados do Global Burden of Disease Collaborative Network. Global Burden of Disease (GBD) Cardiovascular Burden Estimates 1990 and 2021, Institute for Health Metrics and Evaluation, University of Washington. ^48^

•De acordo com um estudo de série temporal, a taxa de mortalidade por IAM padronizada por idade caiu 44% no Brasil de 1996 a 2016, com substanciais diferenças regionais (variações percentuais: +5% na região norte, +11% na nordeste, -35% no centro-oeste, -68% na sudeste e -85% na sul). As taxas de mortalidade foram corrigidas para causas de óbito mal definidas, códigos *garbage* e subnotificação. Variações temporais foram mais marcantes em mulheres do que em homens e nas capitais do que em outros municípios.^107^

•Usando dados do DATASUS, um estudo relatou diminuição nas taxas brutas de mortalidade por DAC no Brasil em ambos os sexos e todos os grupos etários de 2000 a 2018, exceto por um aumento de 1,78% para os homens acima de 85 anos de idade. Nesse período, as taxas de mortalidade por DAC ajustadas para idade diminuíram em ambos os sexos. De 2000-2008 a 2016-2018, a taxa média anual diminuiu de 97,09 para 78,75 por 100 mil considerando-se ambos os sexos, de 115,89 para 97,23 por 100 mil homens e de 79,82 para 61,91 por 100 mil mulheres.^108^

•Usando dados do DATASUS/SIM, Vieira *et al.* relataram uma redução na taxa de mortalidade por IAM ajustada para idade, em Minas Gerais de 35,7 (IC 95%, 35,3-36,1) por 100 mil habitantes em 2008 para 30,4 (IC 95%, 30,1-30,6) por 100 mil em 2016. As taxas de mortalidade por IAM foram mais altas no inverno e mais baixas no verão.^109^

### Mortalidade após síndrome coronariana aguda

•Vários registros brasileiros de SCA relataram os desfechos de indivíduos admitidos com SCA. Em geral, a taxa de mortalidade nos registros é menor do que a informada no SIH/SUS. Vários estudos ressaltaram diferenças regionais nas práticas de tratamento e mortalidade, assim como piores desfechos nos pacientes admitidos nos serviços públicos em comparação aos admitidos em hospitais privados.^102 - 104 , 110^

•Registros e estudos envolvendo pacientes com SCA de 2003 a 2017 relataram taxas de mortalidade hospitalar variando entre 3,3% e 10,9%.^103 , 104 , 110 - 113^ Tais estudos estão descritos em detalhes na versão 2021 da Estatística Cardiovascular - Brasil.^4^Mais recentemente, taxa de mortalidade hospitalar de 5,6% foi relatada entre 2.290 pacientes com IAMCSST submetidos a uma estratégia fármaco-invasiva e admitidos em hospital universitário na cidade de São Paulo.^114^ Em outro estudo, entre 763 pacientes com SCA (66,5% IAMCSST, 20,6% IAMSSST, 12,4% angina instável) admitidos no Hospital das Clínicas da Universidade Federal de Minas Gerais entre maio de 2016 e setembro de 2019, a taxa de mortalidade hospitalar foi 2,9%.^115^

•Usando dados do DATASUS/SIM/SIH, Vieira *et al.* estimaram redução na mortalidade hospitalar ajustada para idade entre pacientes admitidos com IAM de 13,81% em 2008 para 11,43% em 2016 em Minas Gerais.^109^

•Estudos recentes relataram desfechos de longo prazo após SCA. No registro ACCEPT, que incluiu 4.782 pacientes com SCA em 53 hospitais das cinco regiões geopolíticas brasileiras de 2010 a 2014, a taxa de eventos cardiovasculares maiores foi 13,6% após seguimento de 1 ano.^102^ Entre 542 pacientes com IAMCSST arrolados no *Brasilia Heart Study* , a taxa de mortalidade foi 13,2% após um seguimento mediano de 1,7 ano. Baixa renda foi associada com mais alta mortalidade por todas as causas.^116^ No estudo ERICO, entre 800 pacientes com SCA admitidos em hospital universitário na cidade de São Paulo, submetidos a angiografia invasiva de 2009 a 2013, a taxa de mortalidade geral foi 17,5% após seguimento mediano de 4 anos.^117^

•O registro B-CaRe:QCO incluiu indivíduos consecutivos com SCA admitidos em hospitais públicos em Brasília-DF de janeiro de 2011 a fevereiro de 2020. Esses pacientes (n=4.099 com idade >55 anos e n=2.242 com idade ≤55 anos) foram submetidos a angiografia coronariana invasiva até 48 horas após a admissão. Mortes intra-hospitalares ocorreram em 5,7% e 3,6% daqueles >55 anos e ≤55 anos, respectivamente. As taxas de morte pós-alta foram 15,06 e 5,97 por 1.000 pacientes-ano entre aqueles >55 anos e ≤55 anos, respectivamente, após seguimento mediano de 6,67 anos.^118^

### Mortalidade na síndrome coronariana crônica

•No ensaio clínico MASS III de um único centro, entre pacientes com DAC multiarterial, angina estável e função ventricular esquerda preservada submetidos a CRVM entre 2001 e 2006, as taxas de morte, IAM, revascularização ou acidente cerebrovascular em 10 anos foram 30% e 36% para cirurgias com e sem circulação extracorpórea, respectivamente.^119^

•Em análise retrospectiva da base de dados do MASS, entre 1.719 pacientes com DAC multiarterial estável e função ventricular esquerda preservada submetidos a CRVM, ICP, ou tratamento médico entre 2002 e 2015, as taxas de mortalidade por todas as causas em 5 anos foram 7,5%, 7,5% e 12,3% entre aqueles com escore SYNTAX baixo, intermediário e alto, respectivamente.^120^

•Em análise retrospectiva de 1.001 pacientes com DAC multiarterial incluídos na base de dados do MASS entre 1995 e 2018, as taxas de mortalidade geral foram 23% e 22% entre aqueles com ou sem isquemia miocárdica, respectivamente, após seguimento mediano de 8,7 anos.^121^

### Mortalidade relacionada a intervenção coronariana percutânea

•Em registros e estudos envolvendo pacientes submetidos a ICP de 2005 a 2016, a maioria em instituições públicas, a taxa de mortalidade hospitalar variou de 2,3% a 2,6%.^122 , 123^ No registro ICP-BR, a taxa de mortalidade hospitalar variou de 0,2% para pacientes com angina estável a 6,1% para aqueles com IAMCSST.^123^

### Mortalidade relacionada a cirurgia de revascularização do miocárdio

•No registro BYPASS, entre 2.292 pacientes de todas as regiões brasileiras submetidos a CRVM isolada ou combinada até novembro de 2018, a taxa de mortalidade hospitalar foi 2,8%.^124^

•No registro REPLICCAR-I, entre 2.961 pacientes submetidos a CRVM isolada em dez hospitais entre 2013 e 2016, a taxa de mortalidade por todas as causas foi 3,4% em 30 dias e 5,3% aos 4 anos de seguimento.^125^

•No registro prospectivo REPLICCAR II, entre 3.122 pacientes submetidos a CRVM isolada em cinco hospitais no estado de São Paulo entre julho de 2017 e junho de 2019, utilizou-se pareamento por escore de propensão para parear 1.062 pacientes submetidos a tratamento com enxerto arterial único ou múltiplo, ajustado para o risco de mortalidade. A mortalidade operatória foi 1,88% nos dois grupos.^126^

•Em estudo de um único centro no sul do Brasil com 1.767 pacientes submetidos a CRVM isolada entre 2013 e 2018, utilizou-se o pareamento por escore de propensão para criar grupos de pacientes submetidos a CRVM com ou sem circulação extracorpórea (n=1370 e n=397, respectivamente). As taxas de mortalidade em 1 mês foram 2,4% e 1,5% nos grupos com e sem circulação extracorpórea, respectivamente (p=0,401).^127^

•Várias outras experiências de centros únicos, com análises retrospectivas e prospectivas, relataram taxas de mortalidade de curto prazo variando de 1,9% a 11,7% para pacientes submetidos a CRVM.^128 - 131^

## Carga de Doença

•As estimativas do GBD para as taxas de YLDs, YLLs e DALYs atribuídos a DAC para o ano de 2019 e a série histórica de 1990 a 2019 foram publicadas na versão 2021 da Estatística Cardiovascular - Brasil.^4^

•A estimativa do GBD para a taxa de DALYs atribuídos a DAC padronizada por idade foi 1.432,5 (II 95%, 1.345,3-1.511,2) por 100 mil habitantes para 2021 no Brasil. As mais baixas taxas foram estimadas no Distrito Federal (1.049,1 [II 95%, 939,5-1.153,0]), Minas Gerais (1.111,3 [II 95%, 983,7-1.218,0]) e Amazonas (1.117,4 [II 95%, 998,0-1.210,4]), enquanto as mais altas taxas foram observadas em Pernambuco (1.752,5 [II 95%, 1.582,0-1.921,1]), Rio de Janeiro (1.762,7 [II 95%, 1.583,4-1.935,2]) e Maranhão (1.850,9 [II 95%, 1.620,1-2.091,6] por 100 mil habitantes) ( **Tabela 3-1** ).^11^

## Utilização e Custo da Atenção à Saúde

•Em uma abordagem de modelagem global para avaliar o impacto econômico de quatro condições cardíacas no Brasil (hipertensão, insuficiência cardíaca, IAM e fibrilação atrial), o IAM representou o maior custo financeiro, com custo por caso para o sistema de saúde de US$ 48.118 e custo de produtividade de US$ 18.678.^132^

•De acordo com estudo publicado em 2008, o custo anualizado por indivíduo com DAC crônica foi estimado em R$ 2.733±2.307 no SUS, sendo que o custo para o paciente ambulatorial correspondeu a 54% do total. Para os planos de saúde privados, o custo foi estimado em R$ 6.788±7.842, sendo 69% referentes a custos com pacientes internados. Quanto ao custo dos pacientes ambulatoriais, os medicamentos foram responsáveis por R$ 1.154, representando, para os pagadores públicos e privados, 77% e 55% dos custos com os pacientes ambulatoriais e 42% e 17% do custo total, respectivamente.^133^

•Alexim *et al* . analisaram os dados de 1.088 jovens com SCA submetidos a revascularização coronariana em hospitais públicos em Brasília-DF entre 2013 e 2015. Os custos diretos para CRVM e ICP foram equivalentes (Int$ 3.141 [IIQ: 15.392]/ano e Int$ 3.348 [IIQ: 17.884]/ano), enquanto o custo por perda de produtividade laboral foi mais alto no grupo ICP (Int$ 4.511 [IIQ: 18.062]/ano e Int$ 3.578 [IIQ: 13.198]/ano, p = 0,049) em comparação ao grupo CRVM.^134^

•De acordo com dados administrativos do sistema público de saúde, o número de hospitalizações por DAC (aguda e crônica) permaneceu estável de 2015 a 2019, com mais pacientes admitidos por SCA do que por SCC. Em 2020 e 2021, houve redução de ~13% nas admissões clínicas. O número anual de internações para ICP aumentou continuamente de 38.635 em 2008 para 85.518 em 2019, diminuiu para uma média de 79.018 por ano em 2020-2021 e depois aumentou para 80.909 em 2022. O número anual de hospitalizações para CRVM manteve-se estável de 2008 (20.515) a 2019 (21.018), diminuiu para uma média de 16.243 por ano em 2020-2021 (redução de 26%) e depois aumentou para 19.565 em 2022.

•Houve significativas mudanças no reembolso e custo médio por procedimento para tratamento de DAC no SUS. Embora o número de ICPs realizadas nos hospitais públicos tenha mais do que dobrado de 2008 a 2022, o *ticket* médio por caso caiu à metade (valores ajustados: de R$ 12.916 em 2008 para R$ 6.443 em 2022) ( **Tabela 3-2** ). Tendência similar foi observada para ICP primária.^12^

**Tabela 3-2:** – Número e valores reembolsados ajustados das angioplastias coronarianas realizadas no SUS de 2008 a 2022

Ano		ANGIOPLASTIA CORONARIANA	
**Número de procedimentos**	**Valor total pago (ajustado para inflação)**	**Valor médio por procedimento**
2008	38.635	R$ 499.028.630,90	R$ 12.916,49
2009	45.648	R$ 596.852.199,60	R$ 13.075,10
2010	49.492	R$ 634.390.961,40	R$ 12.818,05
2011	55.931	R$ 684.756.412,70	R$ 12.242,88
2012	60.959	R$ 707.818.064,00	R$ 11.611,38
2013	63.838	R$ 699.061.695,70	R$ 10.950,56
2014	66.492	R$ 697.953.632,50	R$ 10.496,81
2015	66.550	R$ 657.219.710,30	R$ 9.875,58
2016	69.802	R$ 624.864.836,60	R$ 8.951,96
2017	73.971	R$ 632.773.406,80	R$ 8.554,34
2018	78.575	R$ 653.083.595,90	R$ 8.311,60
2019	85.518	R$ 693.261.774,50	R$ 8.106,62
2020	77.846	R$ 620.573.346,40	R$ 7.971,81
2021	80.190	R$ 613.985.563,10	R$ 7.656,64
2022	80.909	R$ 521.362.010,90	R$ 6.443,81

Fonte: Ministério da Saúde do Brasil – Sistema de Informações Hospitalares do Sistema Único de Saúde (SIH/SUS). ^49^

•O número de CRVM por DAC no SUS foi similar em 2008 (20.515) e 2022 (19.565) e o valor ajustado reembolsado por CRVM (ticket médio) diminuiu em ~23% (de R$ 20.339 em 2008 para R$ 15.723 em 2021).^49^

## Qualidade da Atenção à Saúde

•Várias publicações sobre qualidade do cuidado em SCA no Brasil são descritas em detalhe na versão 2021 da Estatística Cardiovascular - Brasil.^4^ Esses estudos destacam as oportunidades de melhoria para o cuidado em saúde, as diferenças regionais nos índices de desempenho da qualidade do cuidado e a heterogeneidade entre os serviços públicos e privados.^102 , 103 , 110 , 113 , 135 - 139^ Por exemplo, no registro ACCEPT, a taxa de total adesão às medicações recomendadas nas diretrizes foi de apenas 62% logo após admissão por SCA, sendo que 18% dos pacientes com IAMCSST não foram submetidos a fibrinólise nem a ICP primária.^102^ No estudo BRACE, a qualidade do cuidado na SCA foi pior nas regiões norte e nordeste do que no resto do país.^103^ No estudo VICTIM, o tempo médio entre o início dos sintomas e a admissão hospitalar foi maior e a taxa de ICP primária foi mais baixa nos serviços públicos do que nos privados.^110^ Importante notar que a baixa qualidade do cuidado na SCA foi associada a maior risco de desfechos duros e mortalidade hospitalar nos estudos ACCEPT e BRACE.^102 , 103^

•Algumas publicações, descritas em detalhe na versão 2021 da Estatística Cardiovascular - Brasil,^4^ relataram o sucesso de algumas estratégias, como incorporação de telemedicina, para melhorar a qualidade do cuidado na SCA (por exemplo, aumento na taxa de terapia de reperfusão e do uso de antiagregantes plaquetários e estatinas, além de redução da mortalidade hospitalar).^137 - 139^

•A implementação do SAMU, o serviço brasileiro de ambulância, reduziu levemente a taxa de mortalidade por IAM (OR = 0,967 [IC 95%, 0,936-0,998]) e a mortalidade hospitalar por IAM (OR = 0,914 [IC 95%, 0,845-0,986]) de 2008 a 2016 em Minas Gerais.^109^

•Tempos fundamentais na estratégia fármaco-invasiva para o IAMCSST foram relatados por Bianco *et al.* , que analisaram 2.290 pacientes admitidos em um hospital universitário na cidade de São Paulo. Encontraram os seguintes tempos:

▫dor-unidade de saúde: mediana, 120 (IIQ 60-220) minutos;

▫porta-agulha: mediana, 71 (IIQ 42-135) minutos;

▫dor-agulha: mediana, 222 (IIQ 140-345) minutos.^114^

•No registro B-CaRe:QCO, ~90% dos pacientes admitidos com IAMCSST foram submetidos a estratégia fármaco-invasiva. Carvalho *et al.* relataram os seguintes tempos para os pacientes com idade >55 anos:

▫dor-hospital primário: média, 165 (desvio-padrão, 142) minutos;

▫porta-agulha: mediana 70 (IIQ 43-120) minutos;

▫dor-agulha: mediana 225 (IIQ 150-335) minutos.

▫Os respectivos tempos para os pacientes com idade ≤55 anos foram: média, 154 (desvio-padrão, 131); mediana, 68 (IIQ 43-111); e mediana, 210 (IIQ 140-315) minutos.^118^

•No registro B-CaRe:QCO, terapia com estatina foi prescrita na alta para 83% dos pacientes com idade >55 anos e 82% daqueles com idade ≤55 anos. As respectivas taxas de prescrição de ácido acetilsalicílico na alta foram 91% e 89%.^118^

•Passaglia *et al.* relataram taxas de adesão a terapias baseadas em evidência em 763 pacientes com SCA admitidos no Hospital das Clínicas da Universidade Federal de Minas Gerais entre maio de 2016 e setembro de 2019. Os pacientes foram incluídos no programa Boas Práticas em Cardiologia da Sociedade Brasileira de Cardiologia. As taxas de adesão foram superiores a 85% para os seguintes indicadores de desempenho: uso precoce de ácido acetilsalicílico; ácido acetilsalicílico na alta; inibidor da enzima de conversão da angiotensina ou bloqueador do receptor de angiotensina na alta para pacientes com fração de ejeção ventricular esquerda <45%; betabloqueador na alta; terapia com estatina na alta; e controle da pressão arterial na alta. A adesão ao aconselhamento para cessação do tabagismo para fumantes ativos na alta foi de 81,5%.^115^

•Nos 542 pacientes com IAMCSST arrolados no *Brasilia Heart Study* , os de baixa renda menos frequentemente receberam dupla antiagregação plaquetária e estatinas na alta hospitalar em comparação aos de alta renda.^116^

•Entre 2.290 pacientes com IAMCSST submetidos a estratégia fármaco-invasiva e admitidos em hospital universitário na cidade de São Paulo, uma maior prevalência de sintomas atípicos na chegada, mais longos tempos entre início de sintomas e busca por cuidado e maiores atrasos para receber fibrinólise foram observados nas mulheres.^114^

•Em estudo retrospectivo transversal com coleta de dados por profissionais de saúde comunitária do programa de atenção primária Estratégia de Saúde da Família, entre 2016 e 2021, dos 35.103 indivíduos com infarto do miocárdio ou acidente vascular cerebral prévios, apenas 6,7% e 0,6%, respectivamente, relataram uso de estatina e de terapia com alta dose de estatina. O uso de estatina foi associado a idade acima de 60 anos, residir na região sul e presença de comorbidades (insuficiência cardíaca, diabetes *mellitus* ou doença renal crônica).^91^

## Impacto da Pandemia de COVID-19

•Um estudo ecológico de série temporal avaliou as admissões hospitalares (dados apenas do SUS) e as taxas de mortalidade por DCV no país durante a pandemia de COVID-19. Em comparação aos valores médios nos três anos anteriores (2017-2019), as admissões hospitalares por SCC e SCA caíram 12,8% (IC 95%, 12,5%-13,2%) e 13,6% (IC 95%, 13,3%-13,9%), respectivamente, durante a pandemia ( **Tabela 3-3** ). A mortalidade hospitalar relacionada a SCC e SCA aumentou e a proporção de pacientes com SCA admitidos em UTI foi mais alta durante a pandemia ( **Tabela 3-3** ), podendo refletir casos mais graves e/ou atraso nas admissões. As taxas de mortalidade por SCC e SCA padronizadas por idade por 100 mil habitantes diminuíram 23% (IC 95%, 21%-25%) e 6% (IC 95%, 5%-7%), respectivamente ( **Tabela 3-3** ), embora quase não tenha havido mudança quando SCA foi analisada não apenas como causa básica de óbito, mas também como causa associada (redução de 1% [IC 95%, 0%-2%]). Aumento na proporção de óbitos domiciliares foi relatado para tanto SCC quanto SCA ( **Tabela 3-3** ).^15^

**Tabela 3-3:** – Número de admissões hospitalares, porcentagem de mortalidade hospitalar, taxa de mortalidade (por 100 mil habitantes) e porcentagem de óbitos domiciliares por síndromes coronarianas crônica e aguda durante a pandemia de COVID-19

		Observado (IC 95%)	Esperado (IC 95%)	Razão de risco* (IC 95%)
Síndrome coronariana crônica	Admissões hospitalares (n)	115.250 (111.624;118.876)	132.173 (129.754;134.592)	0,872 (0,868;0,875)
	Mortalidade hospitalar (%)	3,3 (3,2;3,4)	3,0 (2,9;3,0)	1,116 (1,098;1,134)
	Taxa de mortalidade	7,15 (7,04;7,27)	9,29 (9,16;9,42)	0,77 (0,75;0,79)
	Óbitos domiciliares (%)	0,37 (0,36;0,38)	0,31 (0,31;0,32)	1,17 (1,16;1,19)
Síndrome coronariana aguda	Admissões hospitalares (n)	171.287 (166.244;176.330)	198.208 (196.571;199.845)	0,864 (0,861;0,867)
	Admissões em UTI (%)	26,5 (26,3;26,7)	24,8 (24,6;25,0)	1,068 (1,064;1,072)
	Mortalidade hospitalar (%)	10,8 (10,6;10,9)	9,9 (9,8;10,0)	1,085 (1,077;1,093)
	Taxa de mortalidade	44,58 (44,29;44,86)	47,3 (47,01;47,59)	0,94 (0,93;0,95)
	Óbitos domiciliares (%)	0,37 (0,36;0,37)	0,33 (0,33;0,34)	1,10 (1,10;1,11)

Semanas epidemiológicas: 10/2020-21/2021 para dados hospitalares, 10/2020-11/2021 para taxa de mortalidade na população. UTI: unidade de terapia intensiva. * Razão de risco é a razão entre os valores observado e esperado. Fonte: Brant et al. ^15^

•Brant *et al.* compararam mortes por DCV observadas e esperadas durante a fase inicial da pandemia de COVID-19 (17 de março a 22 de maio de 2020) nas seis cidades brasileiras com os mais altos números de mortes por COVID-19 no período (São Paulo, Rio de Janeiro, Fortaleza, Recife, Belém e Manaus). O número de mortes especificadas por SCA aumentou em Belém e Manaus, mas diminuiu nas outras cidades. O número de mortes cardiovasculares não especificadas aumentou em todas as cidades e foi correlacionado aos óbitos domiciliares. O efeito líquido foi um aumento no percentual de excesso de mortes cardiovasculares totais em todas as cidades (especialmente Belém e Manaus), exceto Rio de Janeiro e Recife.^8^

•Em um registro de pacientes de 16 hospitais em seis diferentes estados, o número de indivíduos admitidos no setor de emergência com suspeita de SCA (especialmente SCA sem supradesnível do segmento ST) nos primeiros três meses da pandemia de COVID-19 diminuiu 36,5% em comparação à média mensal nos 12 meses anteriores.^140^

•Em estudo de série temporal no contexto do SUS, em comparação ao período entre março e maio de 2019, as admissões por IAM caíram 4% de março a maio de 2020, enquanto as mortes hospitalares caíram 9% e a taxa de letalidade (porcentagem de mortes nas admissões) caiu 5%.^141^

•Em estudo ecológico com residentes de Belo Horizonte com idade ≥30 anos, não se observou excesso de mortalidade por SCA ao se comparar as taxas padronizadas por idade observadas nas semanas epidemiológicas 10-48 de 2020 com as taxas esperadas (média do período 2015-2019). Os autores relataram reduções nas taxas de admissão hospitalar (RR 0,79; IC 95%, 0,75-0,84) e mortes hospitalares (RR 0,76; IC 95%, 0,57-1,01) por SCA, além de aumento nos óbitos domiciliares (RR 1,38; IC 95%, 1,04-1,82).^66^

•Em estudo de série temporal em Belo Horizonte, o número de hospitalizações por SCA no período pandêmico de 2020 foi 21% menor do que o projetado (n=2.369 e 3.013, respectivamente).^142^

•As admissões por eventos coronarianos urgentes e eletivos entre 18 de março e 30 de setembro de 2020 foram comparadas às hospitalizações durante uma série histórica de dois anos (2018 e 2019) no contexto do plano de saúde UNIMED-BH, um sistema médico privado que cobre aproximadamente 1,31 milhão de indivíduos (22% da população) na área metropolitana de Belo Horizonte. Os números de admissões por DAC foram 2.789, 3.519 e 2.348 em 2018, 2019 e 2020, respectivamente, indicando uma redução de 26% (IC 95%, 22%-30%) em 2020 em comparação à série histórica. A taxa de mortalidade hospitalar foi significativamente mais alta em 2020 (5,4%; IC 95%, 4,5%-6,4%) do que em 2018-2019 (3,6%; IC 95%, 3,2%-4,1%).^143^

## Perspectiva

•Dados adicionais são necessários para a melhor compreensão da distribuição epidemiológica da DAC no Brasil, em particular:

1. Desenvolvimento de bases de dados em âmbito nacional para a coleta de informação precisa e em tempo real sobre a epidemiologia das distintas apresentações clínicas da DAC, incluindo medidas de prestação do cuidado, de desempenho e de desfecho;

2. Análises de dados desagregados para um entendimento profundo dos diversos aspectos da epidemiologia e do tratamento da DAC de acordo com sexo, idade, raça, educação, renda, acesso ao sistema de saúde, região e outras características do microambiente;

3. Revisões sistemáticas das taxas de prevalência e de mortalidade por SCA, angina estável, ICP e CRVM, incluindo amostras representativas de todas as regiões geográficas do país, estratificadas segundo o tipo de sistema de saúde;

4. Avaliação da efetividade dos programas nacionais estruturados para medida da qualidade e do desempenho dos diferentes provedores (público, sem e com fins lucrativos) para entender a atual situação, além de elaborar estratégias para reduzir a morbimortalidade por DCV;

5. Análises econômicas e de custo-efetividade adicionais do impacto da DAC e de suas intervenções diagnósticas e terapêuticas, a partir de nível macro e usando métodos de microcusteio para os sistemas de saúde público e privado;

6. Desenvolvimento de programas estruturados para avaliar a prevalência, a incidência, a clínica e o impacto econômico da DAC crônica no contexto ambulatorial;

7. Análises para avaliar o impacto de longo prazo da pandemia de COVID-19 na prevalência, incidência, mortalidade e carga da DAC, assim como na efetividade de seu tratamento.

## CAPÍTULO 4 – CARDIOMIOPATIA E INSUFICIÊNCIA CARDÍACA

### CID-10 I42; I50; B57.2.

#### Ver Figuras 4-1 a 4-6

**Table:** Abreviaturas Usadas no Capítulo 4

BREATHE	I Registro Brasileiro de Insuficiência Cardíaca
CID-10	Classificação Estatística Internacional de Doenças e Problemas Relacionados à Saúde, 10^a^ Revisão
CMCh	Cardiomiopatia da doença de Chagas
DALYs	Anos de vida perdidos ajustados por incapacidade (do inglês, * Disability-Adjusted Life-Years* )
DCh	Doença de Chagas
GBD	Global Burden of Disease
HR	Hazard Ratio
IC	Intervalo de Confiança
II	Intervalo de Incerteza
RR	Risco Relativo
SEADE	Fundação Sistema Estadual de Análise de Dados
SUS	Sistema Único de Saúde
T. cruzi	Trypanosoma cruzi
UF	Unidade Federativa
US	United States
YLDs	Anos vividos com incapacidade (do inglês, *Years Lived with Disability* )
YLLs	Anos potenciais de vida perdidos (do inglês, * Years of Life Lost)*

## Cardiomiopatia e Miocardite

### Prevalência e Incidência

•De acordo com as estimativas do Estudo GBD 2019, que são os últimos dados completos disponíveis, a prevalência de cardiomiopatia e miocardite padronizada por idade no Brasil diminuiu 4,7% (95% II, -9,5;0.8) de 1990 a 2019. Em números absolutos, as estimativas de prevalência de cardiomiopatia e miocardite no Brasil passaram de menos de 60 mil em 1990 para mais de 160 mil em 2019, principalmente devido ao crescimento e envelhecimento da população. Mais detalhes sobre os dados de cardiomiopatia e miocardite do Estudo GBD 2019 estão disponíveis na versão de 2021 da Estatística Cardiovascular - Brasil.^4^

### Mortalidade

•De acordo com as estimativas do GBD 2021, a taxa de mortalidade por miocardite no Brasil foi 0.2 (II 95%, 0.2-0.2), sendo as mais altas taxas observadas em Roraima (0,7; II 95%, 0,6-0,8) e Goiás (0,4; II 95%, 0,3-0,4). Além disso, de acordo com as estimativas do GBD 2021, a taxa de mortalidade por outras cardiomiopatias foi 7,0 (II 95%, 6,3-7,4). As UF com as mais baixas taxas de mortalidade foram Rio Grande do Norte (3,6; II 95%, 3,1-4), Rio Grande do Sul (3,6; II 95%, 3,1-4) e Amazonas (4,0; II 95%, 3,5-4,4), enquanto as de mais altas taxas foram Roraima (12,8; II 95%, 10,7-15,1), São Paulo (11,3; II 95%, 10-12,2) e Rio de Janeiro (9,7; II 95%, 8,7-11). Como os dados do GBD 2021 não representam uma série histórica, não podem ser comparados a dados anteriores. Os últimos dados completos do GBD disponíveis são de 2019.

•De acordo com as estimativas do Estudo GBD 2019, as taxas de mortalidade por cardiomiopatia e miocardite pareceram aumentar na década de 1990, mas diminuíram nas duas décadas seguintes. As mortes por insuficiência cardíaca que resulta de outras causas específicas são atribuídas à doença de base, i.e., mortes relacionadas a cardiomiopatia isquêmica são codificadas como devidas a doença isquêmica do coração. Além disso, para o projeto GBD, a insuficiência cardíaca não é considerada uma causa de morte primária e, portanto, todas as mortes codificadas como relacionadas a insuficiência cardíaca são recodificadas para a condição de base (ver adiante). Mais detalhes sobre mortalidade por cardiomiopatia e miocardite do Estudo GBD 2019 estão disponíveis na versão de 2021 da Estatística Cardiovascular - Brasil.^4^

•Em estudo relatando dados da Fundação SEADE, do estado de São Paulo, as cardiomiopatias foram responsáveis por um total de 3.571 óbitos, correspondendo a 23,3% das mortes relacionadas a insuficiência cardíaca em 2006, a saber: cardiomiopatia dilatada, responsável por 17,2% das mortes; cardiomiopatia alcoólica, por 0,45%; e cardiomiopatias restritivas, por 0,37%. A CMCh e a cardiomiopatia alcoólica foram responsáveis por 7,8% e 0,45% das mortes relacionadas a insuficiência cardíaca, respectivamente.^144^

•Dados sobre cardiomiopatias específicas são escassos. Em estudo de coorte de 214 pacientes com cardiomiopatia hipertrófica, acompanhados por 7 anos em hospital terciário de São Paulo, a idade média foi 37±16 anos, sendo 52% mulheres. Houve 22 mortes (10%), 15 diretamente relacionadas à cardiomiopatia hipertrófica (11 mortes súbitas). As taxas de sobrevida acumulada foram 94,5% em 5 anos, 91% em 10 anos e 87,9% em 15 anos, com taxa de mortalidade anual de 1%, que é baixa, considerando que o estudo foi realizado em um centro de referência.^145^

### Carga de Doença

•De acordo com as estimativas do GBD 2021, a taxa de DALYs por miocardite foi 8,7 (II 95%, 7,7-9,8), sendo as mais altas taxas observadas em Roraima (23; II 95%, 20,1-26,4), Goiás (12,6; II 95%, 10,7-14,8) e Paraná (12; II 95%, 10,4-13,8). Além disso, de acordo com as estimativas do GBD 2021, a taxa de DALYs por outras cardiomiopatias foi 184,2 (II 95%, 174,4-194,3). As UF com as mais baixas taxas de DALYs foram Rio Grande do Sul (86,1; II 95%, 77,3-94,5) e Rio Grande do Norte (96,8; II 95%, 87,8-106,2), enquanto as UF com as mais altas taxas de DALYs por outras cardiomiopatias foram São Paulo (303,1; II 95%, 275,2-332,4), Roraima (281; II 95%, 238,3-331,5) e Rio de Janeiro (279,1; II 95%, 254,1-310,6). Como os dados do GBD 2021 não representam uma série histórica, não podem ser comparados a dados anteriores. Os últimos dados completos do GBD disponíveis são de 2019 e foram discutidos na Estatística Cardiovascular – Brasil 2021.^4^ Em resumo, de acordo com as estimativas do GBD 2019, as tendências das taxas de DALYs por cardiomiopatia e miocardite padronizadas por idade foram similares àquelas de mortalidade, com um pequeno aumento na década de 1990 e diminuição nas décadas seguintes.

## Doença de Chagas Crônica e Cardiomiopatia da Doença de Chagas

### Prevalência e Incidência

•A prevalência de DCh no Brasil em 2010 foi estimada em 1.156.821 pela Organização Mundial da Saúde,^146^ sendo essa a última estimativa oficial disponível, publicada em 2015. De acordo com tal estimativa, o número de indivíduos com CMCh no Brasil era 231.364. Esses números revelam uma tendência significativa de diminuição de casos humanos de DCh no Brasil em relação às estimativas anteriores, sendo isso atribuído a vários fatores, mas principalmente à quase completa interrupção da transmissão vetorial e transfusional no Brasil. Essa redução é relatada na versão de 2021 da Estatística Cardiovascular - Brasil baseada nas estimativas do Estudo GBD 2019.^4^

•Uma revisão sistemática incluindo 42 artigos confirmou uma redução na prevalência de DCh.^147^ Entretanto, estima-se que cerca de 4,6 milhões (IC 95%, 2,9-7,2 milhões) de pessoas tenham sido infectadas pelo *T. cruzi* em 2010. Essas estimativas são bem maiores do que as da Organização Mundial da Saúde para 2010.^146^

•No estudo de coorte retrospectivo sobre DCh dos *National Institutes of Health* , REDS-II, doadores de sangue inicialmente saudáveis com uma doação-índice soropositiva para *T. cruzi* pareados por idade, sexo e período com doadores soronegativos foram acompanhados por 10 anos.^148^ A incidência diferencial de cardiomiopatia atribuível à infecção por *T. cruzi* foi 1,85 por 100 pessoas-ano. Quando acompanhados prospectivamente por mais uma década, de 2008–2010 a 2018–2019, a incidência de cardiomiopatia nos doadores soropositivos para *T. cruzi* foi 13,8 (IC 95%, 9,5–19,6) eventos/1.000 pessoas-ano (32/262, 12%) em comparação a 4,6 (IC 95%, 2,3–8,3) eventos/1.000 pessoas-ano (11/277, 4%) em controles soronegativos, com uma diferença absoluta na incidência associada à soropositividade para *T. cruzi* de 9,2 (IC 95%, 3,6–15,0) eventos/1.000 pessoas-ano.^149^

•No Estudo de Coorte de Idosos de Bambuí, a incidência de cardiomiopatia foi avaliada em residentes idosos de Bambuí infectados e não infectados sem maiores anormalidades no ECG na linha de base em 1997, que foram seguidos até 2008. No grupo com DCh (n=245), a incidência de cardiomiopatia foi 24,8 (IC 95%, 19,5–30,1) eventos/1.000 pessoas-ano e, no grupo controle (n=617), 15,5 (IC 95%, 12,7–18,5) eventos/1.000 pessoas-ano. A diferença absoluta na incidência associada à DCh em todo o período de 1997 a 2008 foi 9,25 (IC 95%, 3,2–15,2).^150^

•Em uma revisão sistemática e meta-análise de 32 estudos com pacientes com DCh (amostras variaram de 9 a 3.336 participantes), a estimativa da taxa anual *pooled* de cardiomiopatia foi 4,6% entre aqueles com DCh aguda e 1,9% entre aqueles com a forma crônica indeterminada de DCh.^151^

### Mortalidade

•No Estudo GBD 2019, tanto o número de mortes quanto a taxa de mortalidade padronizada por idade por DCh no Brasil diminuíram de 1990 a 2019. Mais detalhes sobre os dados de DCh do Estudo GBD 2019 estão disponíveis na versão de 2021 da Estatística Cardiovascular - Brasil.^4^

•Da mesma forma, vários estudos de base populacional mostraram uma redução na mortalidade por DCh no Brasil nas últimas décadas, como indicado na Estatística Cardiovascular – Brasil 2021, tendo todos relatado reduções na taxa de mortalidade com variações regionais.^4^

•Um recente estudo ecológico de base populacional no nordeste do Brasil, usando dados de 2016 a 2018, avaliou 801 pacientes com DCh crônica nas mesorregiões de Pernambuco. A taxa de ocorrência média da doença crônica foi 3,2/100 mil pessoas-ano.^152^Não foram relatados casos de doença aguda. No total, 350 mortes foram registradas, mostrando predominância masculina, idade ≥ 60 anos e doença crônica com envolvimento cardíaco como a principal causa de morte. Padrões similares foram anteriormente relatados, como discutido na Estatística Cardiovascular – Brasil 2021.^4^ A mortalidade proporcional média anual foi 1,6/100 mil pessoas (desvio-padrão, 2,4) ( **Figura 4-1** ).

**Figura 4-1:**
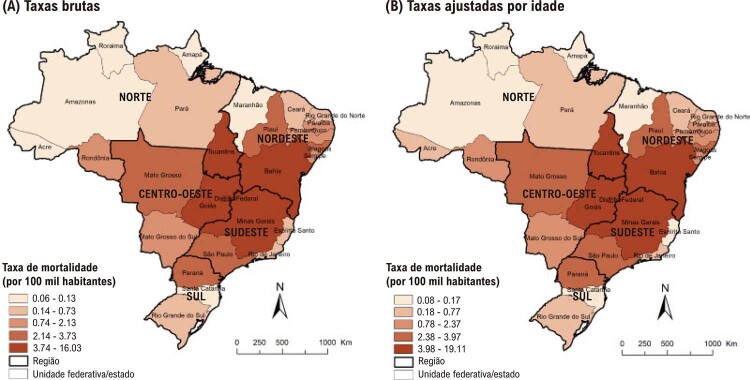
– Taxas padronizadas de mortalidade por doença de Chagas no Brasil de acordo com o grupo etário (em anos) e o ano de ocorrência, de 2000 a 2010. Fonte: Nóbrega et al. ^177^

•Em estudo nacional que analisou todas as declarações de óbito emitidas entre 2000 e 2019 no Brasil, DCh foi mencionada em 122.291 mortes (0,54% das declarações de óbito), 94.788 (77,5%) como causa básica e 27.503 (22,5%) como causa associada. A taxa de mortalidade média padronizada foi 3,22/100 mil habitantes/ano, com tendência de declínio no período.^153^ As mais altas taxas de mortalidade foram observadas nos homens, grupo etário ≥80 anos, raça/cor de pele negra, 1-3 anos de escolaridade e residentes na região Centro-Oeste ( **Figura 4-2** ).

**Figura 4-2:**
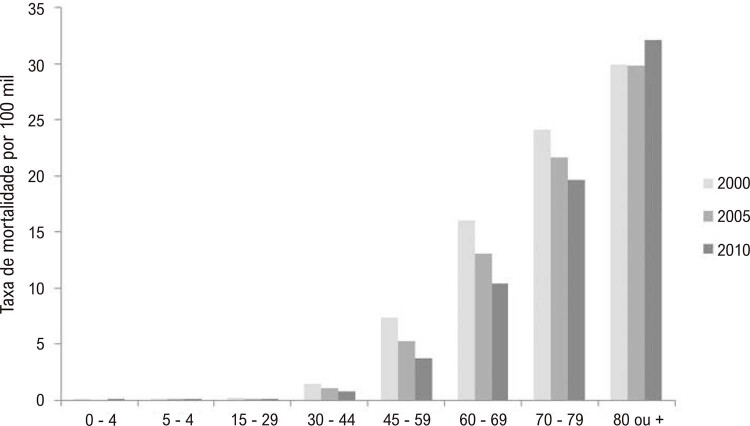
– Distribuição espacial das taxas de mortalidade relacionada à doença de Chagas, por 100 mil habitantes, de acordo com as unidades federativas no Brasil, 2000–2019. (A) Taxas brutas; (B) Taxas ajustadas por idade. Fonte: Martins-Melo et al. ^153^

•No já mencionado Estudo de Coorte de Idosos de Bambuí,^150^ o desenvolvimento de cardiomiopatia aumentou o risco de morte em comparação à manutenção de ECG normal [HR: 1,93 (IC 95%, 1,02–3,65)]. O estudo concluiu que a DCh está associada a maior risco de progressão para cardiomiopatia no idoso, que representa maior risco de morte em indivíduos com DCh.

•Como discutido na Estatística Cardiovascular – Brasil 2021, os estudos prévios relataram aumento do risco de morte de doadores de sangue soropositivos para DCh,^154^ além de subnotificação da DCh como causa de morte. Em pacientes com insuficiência cardíaca, as mortalidades por todas as causas e por insuficiência cardíaca foram significativamente mais elevadas naqueles com DCh em comparação àqueles sem DCh,^155^ com risco de morte aumentado naqueles com cardiomiopatia, maior ainda na presença de aneurisma ventricular esquerdo.^156^ Outra coorte mostrou reduções nas taxas de morte absolutas ao longo do tempo em pacientes com CMCh e cardiomiopatias não DCh, mas observaram-se aumentos na HR associada com DCh ao longo do tempo.^157^ Maior número de eventos cardiovasculares foram relatados nos municípios com menos médicos por 1.000 habitantes ou menor cobertura da Atenção Primária.^158^

•Em um estudo de coorte incluindo 1.551 pacientes com CMCh em Minas Gerais, foi desenvolvido um escore preditivo de mortalidade em 2 anos. O escore incluiu variáveis simples, como idade, classe funcional da *New York Heart Association* , frequência cardíaca, duração do QRS e níveis anormais de NT-proBNP ajustados para idade. As taxas de mortalidade observadas nos grupos de risco baixo, médio e alto foram 0%, 3,6% e 32,7%, respectivamente, na coorte de derivação e 3,2%, 8,7% e 19,1%, respectivamente, na coorte de validação, com estatística C de 0,82 e 0,71, respectivamente. Esse parece ser um escore útil e simples para ser usado em áreas remotas com limitados recursos tecnológicos.^159^

•Uma revisão sistemática mais recente de mortalidade na CMCh incluiu 52 estudos longitudinais (9.569 pacientes e 2.250 mortes).^160^ A taxa anual de mortalidade por todas as causas foi 7,9% [IC 95%, 6,3–10,1]. A estimativa da taxa anual *pooled* de morte cardiovascular foi 6,3% (IC 95%, 4,9–8,0). Meta‐regressão mostrou associação de fração de ejeção ventricular esquerda baixa (coeficiente = -0,04; IC 95%, -0,07;-0,02; *P* = 0,001) com risco de mortalidade aumentado, sendo a mortalidade maior nos estágios mais avançados de insuficiência cardíaca, indicando que o risco de mortalidade na CMCh crônica é substancial e primariamente atribuível a causas cardiovasculares.

### Carga de Doença

•Como relatado na Estatística Cardiovascular - Brasil 2021,^4^ de acordo com o Estudo GBD 2019, foram estimados 174.194 DALYs (II 95%, 109.039-302.974) devidos a DCh no Brasil, com uma redução relativa de 32,1% em comparação ao valor de 1990. As taxas de DALYs padronizadas por idade diminuíram em nível nacional (-70,5%) e em todas as UF brasileiras entre 1990 e 2019, mas com diferentes padrões regionais. A diminuição nas taxas de DALYs foi primariamente devida a uma redução consistente nas taxas de YLLs, o principal componente do total de DALYs por DCh. A maior carga fatal e não fatal por DCh foi observada entre os homens e os idosos e nas UF brasileiras com importantes áreas endêmicas de transmissão vetorial no passado, como Goiás, Tocantins, Minas Gerais, Bahia e Distrito Federal.

### Insuficiência cardíaca

•Como a insuficiência cardíaca não é considerada uma causa básica de morte (i.e., código *garbage* ) no Estudo GBD, todas as mortes atribuídas a insuficiência cardíaca nas declarações de óbito são reclassificadas e/ou redistribuídas para outras causas, de acordo com o método do GBD. Assim, não há dados do GBD sobre mortalidade por insuficiência cardíaca. A insuficiência cardíaca é classificada pelo GBD como um “comprometimento”, portanto, os únicos indicadores do GBD para insuficiência cardíaca são prevalência e YLDs, que é o componente de morbidade do DALYs.

### Prevalência e Incidência

•Os últimos dados completos do GBD disponíveis são de 2017, como discutido na Estatística Cardiovascular – Brasil 2021.^4^A prevalência padronizada por idade de insuficiência cardíaca no Brasil passou de 818 (II 95%, 718-923) em 1990 para 772 (II 95%, 680-875) em 2017, uma diminuição de 5% (95 UI, -7,1;-3) no período. Em números absolutos, as estimativas de prevalência de insuficiência cardíaca no Brasil subiram de 0,67 milhão em 1990 para quase 1,7 milhão em 2017, principalmente devido a crescimento e envelhecimento da população. As mais altas taxas foram observadas no Rio Grande do Norte e as mais baixas, no Acre. A prevalência de insuficiência cardíaca foi maior em mulheres (795; II 95%, 694-901) do que em homens (751; II 95%, 656-845) em 2017, e a redução na prevalência de 1990 a 2017 foi mais pronunciada nos homens, sendo a porcentagem de diminuição 7,5 (II 95%, -10,2;-4,8) para homens e 3,2 (II 95%, -6,5;-0,1) para mulheres. Quanto aos grupos etários, as taxas de incidência aumentaram 10 vezes do grupo de 15-49 anos ao de 50-69 anos, e 6 vezes do último grupo ao de 70+ anos, tendo esses aumentos sido similares para mulheres e homens. De 1990 a 2017, a prevalência aumentou apenas no grupo de 15-49 anos, enquanto diminuiu nos demais, provavelmente em associação com a elevação de eventos isquêmicos naquele grupo etário.

•Uma revisão sistemática, avaliando a carga de insuficiência cardíaca na América Latina, incluiu 143 artigos publicados entre janeiro de 1994 e junho de 2014, com pelo menos 50 participantes com idade ≥ 18 anos; a maioria dos estudos incluídos (64%) foi do Brasil.^161^ A idade média dos pacientes foi 60±9 anos, a fração de ejeção média, 36±9%, e a prevalência de insuficiência cardíaca, 1% (IC 95%, 0,1-2,7). Dos estudos incluídos, apenas um avaliou incidência, com 1.091 indivíduos identificados através de amostragem probabilística em múltiplas etapas na cidade de Porto Alegre. A idade média foi 42,8±16,9 anos e 55% eram mulheres. A incidência de insuficiência cardíaca em um estudo com apenas uma população fornecendo essa informação foi de 199 casos por 100 mil pessoas-ano.^162^

•Em estudo de base populacional em atenção primária de uma cidade brasileira de tamanho médio, 633 indivíduos com idade ≥45 anos foram selecionados aleatoriamente e registrados em um programa de atenção primária. A idade média foi 59,6±10,4 anos e 62% eram mulheres. A prevalência de insuficiência cardíaca sintomática (estágio C) foi 9,3% e a de insuficiência cardíaca estágio B (anormalidades estruturais) foi 42,7%. Dos pacientes com insuficiência cardíaca, 59% apresentavam fração de ejeção preservada e 41% apresentavam fração de ejeção reduzida.^163^

•Um estudo baseado na Pesquisa Nacional de Saúde de 2013, com dados de 59.655 adultos (≥ 18 anos), encontrou prevalência autorreferida de insuficiência cardíaca de 1,1%, representando cerca de 1,7 milhão de indivíduos. Naqueles acima de 60 anos, a prevalência foi de 3,3%.^164^

## Mortalidade

•Um estudo ecológico desenvolvido com dados secundários sobre mortalidade por insuficiência cardíaca no Brasil de 1996 a 2017 relatou 1.242.014 mortes por insuficiência cardíaca, como mostra a **Figura 4-3** .^165^ Os resultados da análise da tendência temporal mostraram redução significativa de 2,3% (IC 95%, -2,3;-2,7) na taxa de mortalidade em todas as UF brasileiras. Os municípios do norte mantiveram taxas de mortalidade baixas no período analisado, sugerindo subnotificação.

**Figura 4-3:**
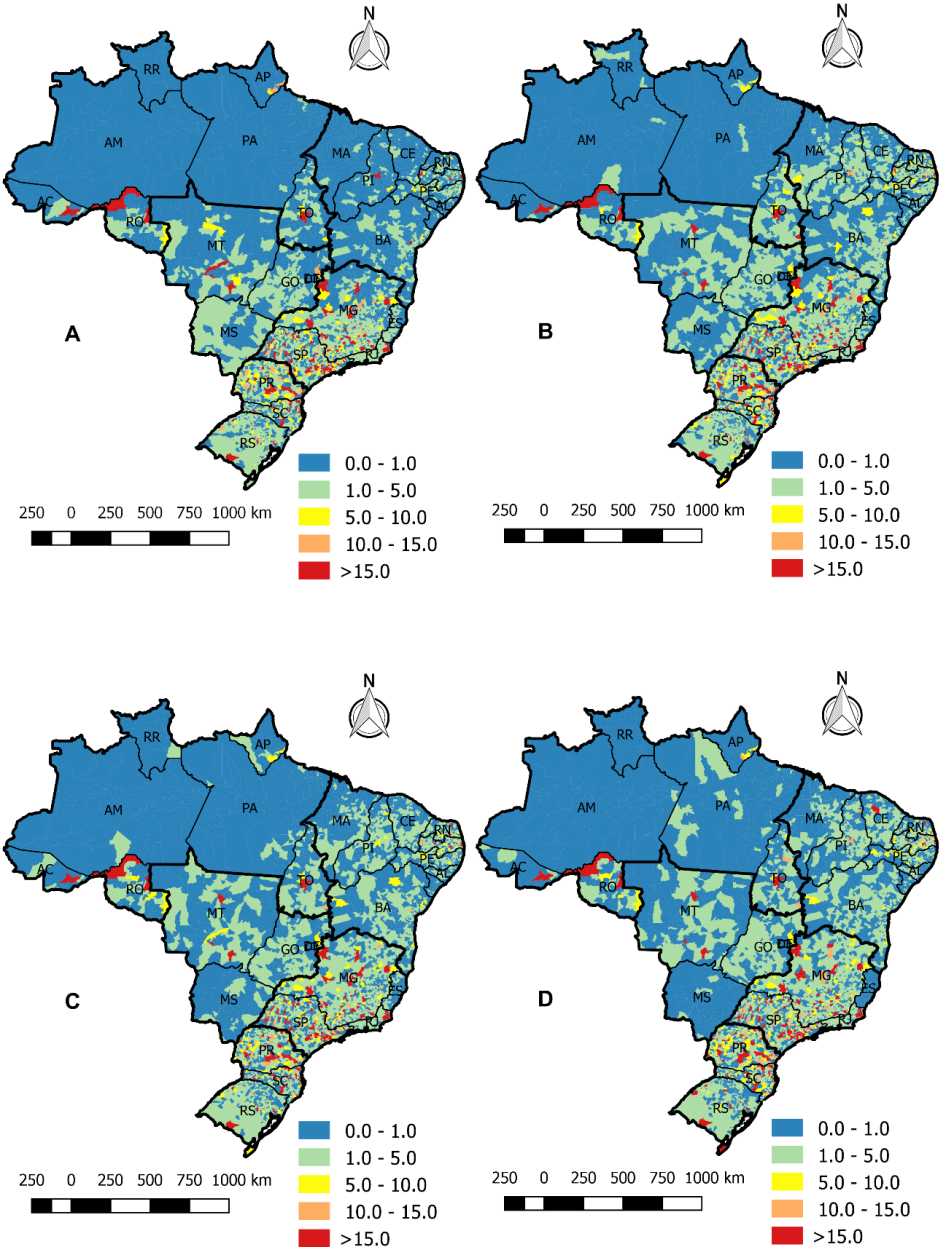
– Risco relativo de mortalidade por insuficiência cardíaca de todos os municípios brasileiros nos períodos: A) 1996-2001; B) 2002-2007; C) 2008-2012; e D) 2013-2017. Fonte: Cestari et al. ^165^

• Outro estudo ecológico analisando mortalidade por insuficiência cardíaca no Brasil para indivíduos com idade igual ou superior a 50 anos, entre 1998 e 2019, mostrou resultados similares.^166^A taxa média foi de 75,5 mortes por 100 mil habitantes e as mulheres corresponderam à maior proporção (n=299.093; 52,67%). A mortalidade por insuficiência cardíaca em brasileiros com idade superior a 50 anos apresentou tendência decrescente em 21 anos, sendo tal tendência observada em ambos os sexos e em 23 UF ( **Figura 4-4** ). Esse estudo mostrou que as taxa de mortalidade por insuficiência cardíaca aumentaram com o avançar da idade em todas as regiões brasileiras, com maior ocorrência em indivíduos a partir dos 80 anos de idade (n=257.277; 45,31%).^166 , 167^

**Figura 4-4:**
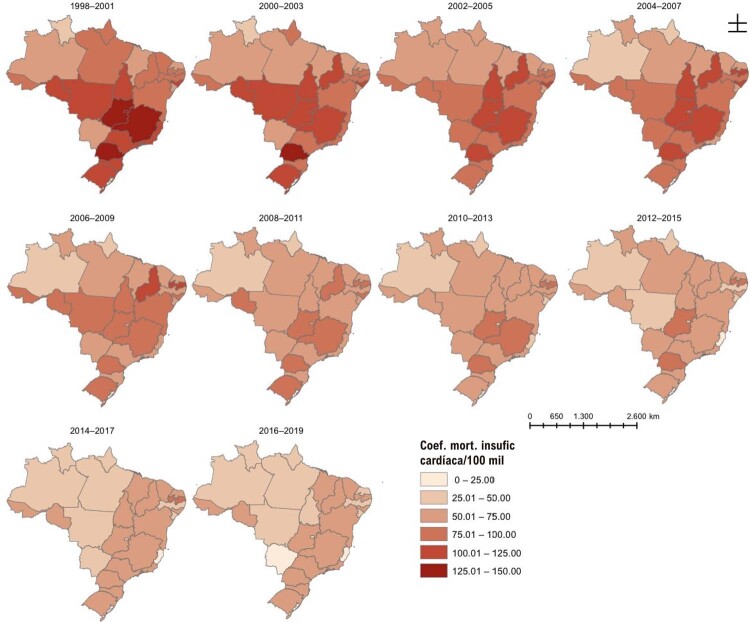
– Distribuição espacial das médias móveis das taxas de mortalidade por insuficiência cardíaca entre os brasileiros com idade superior a 50 anos, Brasil, 1998 a 2019. Fonte: Arruda et al. ^166^

•Um estudo foi realizado com todas as declarações de óbito emitidas no Brasil entre 2006 e 2016 para identificar insuficiência cardíaca como causa básica de morte ou como causa múltipla de morte. Foram incluídas 1.074.038 declarações de óbito emitidas entre 2006 e 2016, em cujas Partes I ou II constasse o código I50. A frequência com que insuficiência cardíaca apareceu como causa múltipla de morte nos dois sexos foi quase três vezes maior do que aquela com insuficiência cardíaca como causa básica de morte. Os códigos específicos da CID mais frequentemente listados como causa básica de morte nas declarações de óbito em que insuficiência cardíaca apareceu em qualquer parte foram cardiopatia hipertensiva (11,3%), infarto agudo do miocárdio (8,4%), diabetes mellitus (3,1%), cardiomiopatia dilatada (2,9%), doença pulmonar obstrutiva crônica (2,6%) e DCh (1,7%).^168^

•Como relatado na Estatística Cardiovascular - Brasil 2021,^4^ a porcentagem de mortes devidas a insuficiência cardíaca varia de 3% a 9%, a depender da região, idade e análise realizada. Ademais, a mortalidade durante o seguimento dos pacientes com insuficiência cardíaca varia nos estudos de coorte brasileiros, com mortalidade em 1 ano variando de 6,8% a 20%, dependendo dos fatores clínicos, e com sobrevida melhorando ao longo do tempo.

## Hospitalizações

•As hospitalizações são a principal consequência de insuficiência cardíaca descompensada, resultando em pior prognóstico e elevando os custos. O Estudo BREATHE avaliou uma amostra de pacientes admitidos por insuficiência cardíaca descompensada aguda. No total, 1.263 pacientes de 51 centros de diferentes regiões brasileiras foram incluídos em 2011 e 2012. A mortalidade hospitalar foi 12,6% e as metas dos indicadores de qualidade assistencial baseados nas recomendações de alta hospitalar foram alcançados em menos de 65% dos pacientes.^167^Como relatado na Estatística Cardiovascular – Brasil 2021^4^ e em avaliação recente de admissões hospitalares por insuficiência cardíaca, taxas de mortalidade hospitalar similares foram informadas, variando de 9% a 17%.^115^

•Várias análises temporais mostraram redução no número de hospitalizações e na mortalidade hospitalar ao longo do tempo, principalmente na última década,^168 , 169^ como discutido com detalhes na Estatística Cardiovascular – Brasil 2021.^4^

## Carga de Doença

•Como relatado na Estatística Cardiovascular – Brasil 2021,^4^ as taxas de YLDs padronizadas por idade por insuficiência cardíaca foram 112 (II 95%, 83-141) em 1990 e 109 (II 95%, 81-134) em 2017 por 100 mil habitantes, correspondendo a diminuição de 3% (II 95%, -6,7 a 0,3). Tais variações são similares às observadas nas taxas de prevalência de insuficiência cardíaca. A despeito dessa diminuição nas taxas de YLDs, a insuficiência cardíaca resultou em 88.114 (II 95%, 64.078-112.624) DALYs no Brasil em 1990 e em 234.169 (II 95%, 174.338-291.188) DALYs em 2017, devido ao crescimento e envelhecimento da população. As reduções foram mais pronunciadas nos homens durante aquele período. Como esperado, as mais altas taxas de YLDs foram observadas no grupo de 70+ anos, seguido pelo grupo de 50-69 anos. À semelhança das variações observadas na prevalência, de 1990 a 2017, os maiores aumentos de YLDs foram verificados no grupo de 15-49 anos.

## Impacto da Pandemia de COVID-19

•Os dados sobre hospitalização e mortalidade no Brasil entre 2020 e 2021 mostram redução na hospitalização por doença cardiovascular, com insuficiência cardíaca e cardiomiopatias apresentando a mais alta redução absoluta nas hospitalizações (-66.499; IC 95%, -58.863,4; -74.035,9). Houve ainda uma redução relativa nas hospitalizações no período (RRi 0,761; IC 95%, 0,759-0,763). Entretanto, houve um aumento na gravidade clínica dos pacientes hospitalizados, com aumento proporcional de 19,4% (RRi 1.194; IC 95%, 1.188-1.200) nas admissões em unidade de terapia intensiva e de 13,6% (RRi 1.136; IC 95%, 1.130-1.143) nas mortes hospitalares. Esses dados sugerem que os pacientes menos graves podem não ter sido hospitalizados e, por outro lado, os mais graves devem ter buscado o hospital mais tardiamente, agravando seus desfechos.^15^

•A maior redução na hospitalização ocorreu em municípios da região norte, naqueles com mais baixo índice de desenvolvimento humano e nos menores. Além disso, a redução foi maior entre idosos e mulheres, refletindo o maior impacto da pandemia nas admissões clínicas dos grupos mais vulneráveis. Quanto à mortalidade por insuficiência cardíaca e cardiomiopatias, houve redução na taxa de mortalidade padronizada e aumento nas mortes domiciliares.^15^

•Outro estudo com dados de janeiro de 2011 a junho de 2022 mostrou redução nas hospitalizações por insuficiência cardíaca desde o início da série, mas com queda mais significativa após o início da pandemia de COVID-19. Dados de 2020 a junho de 2022 mostram média mensal de hospitalização de 8.547 casos, o que representa uma queda de 16,10% em relação aos dados de 2019 e de 37,75% em relação aos de 2011. Isso corresponde a uma diminuição de 132,81 hospitalizações por 100 mil habitantes em 2011 para 78,58 hospitalizações por 100 mil habitantes em 2021 (redução de 40,83%, como mostra a **Figura 4-5** ). Por outro lado, houve elevação da letalidade da insuficiência cardíaca, que aumentou de 10,00% em 2011 para 12,63% em 2019 e para 13,47% em 2022 ( **Figura 4-6** ).^170^

**Figura 4-5:**
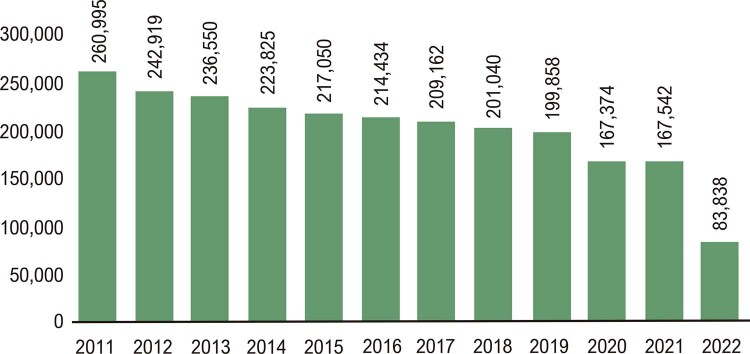
– Série histórica de hospitalizações por insuficiência cardíaca de janeiro de 2011 a junho de 2022. Fonte: Cruz et al. ^170^

**Figura 4-6:**
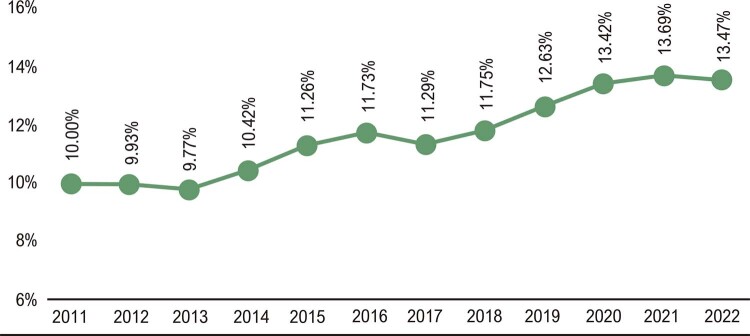
– Série histórica de letalidade da insuficiência cardíaca de 2011 a 2022. Fonte: Cruz et al. ^170^

•Um estudo de coorte usando dados do programa de Boas Práticas Clínicas em Cardiologia comparou as hospitalizações por insuficiência cardíaca durante o surto de COVID-19 de 12 de março a 31 de outubro de 2020 com as admissões nos mesmos hospitais durante as mesmas semanas epidemiológicas em 2019 e 2018. Esse estudo relatou uma redução de 20% no número de hospitalizações. Entretanto, houve significativo aumento na mortalidade dos pacientes hospitalizados. Após ajuste, o risco de morte em 30 dias foi aproximadamente duas vezes maior nos pacientes admitidos durante o surto de COVID-19 (HR = 1,89 [IC 95%, 1,19-3,03]; p=0,007) em comparação ao risco de morte nos dois anos anteriores. Má adesão às recomendações de tratamento e doença renal aguda foram mais comumente relatadas como fatores desencadeantes de descompensação de insuficiência cardíaca durante o surto de COVID-19. Além disso, os pacientes foram hospitalizados com maior gravidade (medida através do escore de risco ADHERE) durante o surto de COVID-19.^171^

•Houve preocupação quanto ao impacto da coinfecção por COVID-19 e DCh, devido tanto às diferentes formas de envolvimento ou complicações cardíacas que a COVID-19 pode causar quanto ao impacto que a COVID-19 aguda e a imunossupressão induzida pela doença e por seu tratamento podem ter na DCh.^172^ Entretanto, um estudo multicêntrico comparando pacientes hospitalizados com COVID-19 e DCh a outros sem DCh não observou diferença em mortalidade, tempo de permanência ou complicações, como insuficiência cardíaca aguda, entre os dois grupos.^173^ Um estudo usando dados de taxa de mortalidade para 2020 obtidos na base de dados do Sistema de Informações sobre Mortalidade do Brasil identificou 492 mortes relacionadas a coinfecção por DCh e COVID-19. As mais altas taxas de mortalidade foram observadas nos grupos etários mais idosos, indivíduos de etnia afrobrasileira e aqueles com baixos níveis educacionais.^174^

***•Utilização e Custo da Atenção à Saúde***

•De acordo com dados do SUS, houve 3.454.570 hospitalizações por insuficiência cardíaca de 2008 a 2021. Esse número representa mais de um terço do total de admissões clínicas relacionadas a condições cardiovasculares no período observado. Os custos totais ajustados foram R$ 8.438.025.775.

•Durante o período observado, houve redução no número de admissões clínicas por insuficiência cardíaca, que passou de 298.474 (157 por 100 mil) em 2008 para 181.441 (85 por 100 mil) em 2021, sendo tal redução uniforme ao longo dos anos. A despeito dessa diminuição no número de admissões, as estimativas das despesas não ajustadas com atenção à saúde a partir de pagamento direto ajustado para o cuidado de pacientes com insuficiência cardíaca aumentaram de 2008 para 2019 em quase 24%, passando de R$ 288.345.221 em 2008 para R$ 356.801.947 em 2021. O número reduzido de admissões e as despesas aumentadas representam maiores custos por admissão no período observado (de R$ 912 em 2008 para R$ 1.787 em 2021). A insuficiência cardíaca foi a principal responsável pelos custos relacionados às admissões clínicas entre as doenças cardiovasculares.

•Um estudo ecológico usando dados de 2018 a 2021 identificou projeções que indicam despesa total de mais de R$ 1 bilhão com hospitalizações, com um custo hospitalar médio de R$ 1.725,27 por pessoa. O custo médio por hospitalização excedeu R$ 2 bilhões. Aproximadamente 3% das despesas federais são reservadas para pagamento de benefícios previdenciários para insuficiência cardíaca. Do total de afastamentos do trabalho, 65% correspondem a homens e 35% a mulheres, com custos que poderiam alcançar R$ 6 bilhões em perdas por ano.^175 , 176^

•Como discutido na Estatística Cardiovascular – Brasil 2021,^4^ a carga econômica de insuficiência cardíaca no Brasil determina um custo financeiro de R$ 22,1 bilhões (US$ 6,8 bilhões), como avaliado em 2015, o segundo custo dentre as quatro principais condições cardíacas no Brasil: infarto do miocárdio, insuficiência cardíaca, hipertensão e fibrilação atrial.^132^ Foram também relatados aumentos no custo médio por paciente das hospitalizações relacionadas à insuficiência cardíaca.

### •Perspectivas

•Por ser a insuficiência cardíaca considerada um código *garbage* quando designada como causa básica de morte, são necessários estudos que investiguem o melhor método para reclassificar e redistribuir essa causa de modo a reduzir viés e propiciar melhor comparabilidade de dados para o aperfeiçoamento das políticas de saúde.

•Estudos brasileiros de coorte sobre cardiomiopatias são raros, tendo alguns estudos clínicos no Brasil informado dados de insuficiência cardíaca, havendo, no entanto, poucos estudos multicêntricos com dados da população brasileira. Vale ressaltar a importância de se poder contar com dados tanto de insuficiência cardíaca quanto de cardiomiopatia, assim como de pacientes ambulatoriais e hospitalizados, além de se compreender de maneira ampla a carga crescente da insuficiência cardíaca nas doenças cardiovasculares. São necessários mais estudos multicêntricos de larga escala para melhor descrever a carga, os desfechos e os custos da insuficiência cardíaca na população brasileira.

•Além disso, estudos que explorem a qualidade e os custos da assistência na insuficiência cardíaca auxiliariam no desenvolvimento de políticas de saúde para melhorar a conscientização, o acesso a intervenções que salvam vidas, a doação de órgãos, assim como o uso de recursos nessa doença tão complexa.

•Mais estudos sobre o impacto da pandemia de COVID-19, assim como sobre a era pós-pandêmica, ainda são necessários.

## CAPÍTULO 5 – DOENÇA VALVAR DO CORAÇÃO

### CID-9 424; CID-10 I34 a I38

#### Ver Tabelas 5-1 e 5-2 e Figuras 5-1 a 5-7

**Table:** Abreviaturas usadas no Capítulo 5

CID-10	Classificação Estatística Internacional de Doenças e Problemas Relacionados à Saúde, 10^a^ Revisão
DALYs	Anos de vida perdidos ajustados por incapacidade (do inglês, * Disability-Adjusted Life-Years* )
DCR	Doença Cardíaca Reumática
DVC	Doença Valvar do Coração
DVNR	Doença Valvar do Coração Não Reumática
EI	Endocardite Infecciosa
GBD	Global Burden of Disease
IC	Intervalo de Confiança
II	Intervalo de Incerteza
SUS	Sistema Único de Saúde
TAVI	Implante percutâneo de válvula aórtica
VMPB	Valvoplastia Mitral Percutânea por Balão

## Prevalência

### Doença Cardíaca Reumática

•A DCR é uma condição que pode ser prevenida. Tratamento imediato e adequado das infecções estreptocócicas com antibióticos pode evitar o desenvolvimento da DCR. Entretanto, em muitos países de renda média e baixa, o acesso a cuidados de saúde e antibióticos pode ser limitado, levando a taxas mais altas de DCR. De acordo com o *Global Atlas on Cardiovascular Disease Prevention and Control* , estima-se que atualmente a DCR afete 33,0-40,5 milhões de pessoas no mundo e seja responsável por 1% a 1,5% (306 mil) de todas as mortes cardiovasculares.^36 , 178^Até meados do século 20, a DCR foi a principal causa de DVC no mundo. Melhores condições de saúde, identificação precoce de infecções por *Streptococcus pyogenes* e uso de antibiótico reduziram significativamente a prevalência de DCR nos países de alta renda.

•A DCR é a causa primária de 2,5% das DVC nos Estados Unidos e Canadá, chegando a 22% na Europa.^179^ Taxas ainda mais altas foram relatadas no Brasil, onde a DCR chegou a ser a causa de 50% das cirurgias cardíacas valvares no SUS.^180 - 182^

•Em países de média e baixa renda, a prevalência de DCR permanece em torno de 444 por 100 mil habitantes.^36 , 183^ No Brasil, persiste como a principal etiologia de DVC, especialmente nos pacientes do SUS. Avaliações prévias mostraram prevalência de 360 por 100 mil no Brasil.^184^ Outras avaliações encontraram prevalência variando de 100 a 700 por 100 mil escolares.^185^ Mais recentemente (2022), um estudo da Bacia Amazônica Brasileira encontrou prevalência de 39 por 1.000, indicando a necessidade de programas de triagem em áreas remotas e de reconhecida alta prevalência.^186^ Critérios ecocardiográficos simplificados podem aumentar a triagem em áreas com suspeita de alta prevalência.^187^

•Conforme dados mais recentes do *Global Burden of Cardiovascular Diseases and Risks Collaboration* , de 1990 a 2021,^186^a prevalência padronizada por idade de DCR na América Latina tropical (que inclui Brasil e Paraguai, com a maioria das estimativas a partir de dados brasileiros) foi 1.266,6 (II 95%, 987,3-1.543,5) por 100 mil, resultando em aproximadamente 3.047.102 casos prevalentes ( **Tabela 5-1** ). Essas taxas pontuais estão acima das observadas no GBD 2019 no Brasil [918,5 (II 95%, 716-1.142,5) por 100 mil] e Paraguai, sugerindo que a prevalência de DCR possa ainda estar crescendo na região. No entanto, também pode significar que diagnóstico, detecção e estatística de saúde estejam melhorando.^186^

**Tabela 5-1:** – Doença cardiovascular regional (endocardite infecciosa, doenças valvares reumáticas e não reumáticas) em 2021 na América do Sul tropical (Brasil e Paraguai): números e taxas padronizadas por idade por 100 mil

Tipo de doença cardiovascular	Casos prevalentes (número)	Mortes (número)	Prevalência (taxa/100 mil)	Mortes (taxa/100 mil)	DALYs (taxa/100 mil)
Doença cardíaca reumática	3.047.102	2.823	1.266,6	1,1	93,5
Doença valvar aórtica calcífica não reumática	359.861	4.059	140,6	1,6	32,2
Doença valvar mitral degenerativa não reumática	112.951	1.761	44,0	0,7	17,2
Outras doenças valvares não reumáticas	474	75	0,2	<0,1	0,8
Endocardite infecciosa	15.053	2.775	6,7	1,1	34,6

Fonte: Adaptado de Lindstrom et al. ^186^

•A DCR ainda é a causa mais prevalente de doença valvar mitral no Brasil tanto estenose mitral (mais de 90%) quanto regurgitação mitral (cerca de 55-60%) são consideradas.^187^

•A estenose mitral é mais frequente em mulheres do que em homens, na razão de 3:2. É sequela comum de febre reumática aguda, alcançando mais de 85% dos casos mesmo nos países de alta renda, como os da Europa,^188^ com padrão similar ao observado no Brasil.^182 , 187^

### Doença Valvar do Coração Não Reumática

•De acordo com atualização recente do GBD 2021,^36 , 37 , 186^ a prevalência padronizada por idade de doença valvar mitral degenerativa não reumática na América Latina tropical em 2021 foi 44,0 (II 95%, 41,5-47,0) por 100 mil, resultando em 112.951 casos prevalentes ( **Tabela 5-1** ),^186^ mostrando um aumento contínuo se comparado às tendências de 1990-2019 para o total de DVNR, passando de 25,3 (II 95%, 22,4-27,8) por 100 mil em 1990 para 39 (II 95%, 33,9-44,6) por 100 mil em 2019.^36^Essa tendência passada, entretanto, resultou principalmente da doença valvar aórtica calcífica (201,8%), pois a doença valvar mitral degenerativa apesentou tendência à estabilidade, com discreta variação percentual: -2,3% (II 95%, -4 a -0,4).^34 , 36^

•A doença valvar aórtica calcífica não reumática apresentou taxa de prevalência padronizada por idade de 140,6 (II 95%, 112,9-169,7) por 100 mil na América Latina tropical, com média de 359.861 casos prevalentes, representando tendência a aumento de prevalência, em especial em idades mais avançadas, como anteriormente observado no Brasil (1990-2019) ( **Tabela 5-1** ).^36 , 186 , 189^

•Outras DVNR com consideráveis desvantagens relacionadas à qualidade dos dados primários e definição de caso apresentaram prevalência padronizada por idade de 0,20 (IC 95%, 0,16-0,24) por 100 mil indivíduos na América Latina tropical.^186^

•No GBD 2021, as estimativas de prevalência para DCR e DVNR não estão disponíveis a nível subnacional nem para o Brasil em separado, impedindo inferências sobre sua associação com acesso a cuidados de saúde e sua qualidade ou ainda com marcadores de desenvolvimento socioeconômico.^186^

•Diferentemente da doença valvar mitral, a doença valvar aórtica é predominantemente degenerativa ou calcífica. Estudos observacionais mostraram estenose aórtica em 4,5% da população com idade >75 anos nos países de alta renda, como os Estados Unidos.^190^ De acordo com estudos observacionais^187 , 191^ e dados do GBD 2021,^186^ no Brasil, assim como no resto do mundo, observou-se tendência a aumento da doença valvar aórtica degenerativa nas áreas em que a transição epidemiológica e as mudanças na composição etária evoluíram, em comparação à das demais etiologias, como DCR.^192^

## Mortalidade

•A DVC é uma das principais causas de morte cardiovascular no Brasil, em particular nas regiões economicamente desfavorecidas, sendo a DCR a etiologia com maior componente social.

### Doença Cardíaca Reumática

•Ainda que os dados em âmbito nacional sejam escassos, as taxas de mortalidade padronizadas por idade atribuíveis à DCR continuam a diminuir. A análise do último estudo GBD 2021 mostrou taxa de 1,0 (II 95%, 1,0-1,2) por 100 mil mortes na América Latina tropical, que inclui Brasil e Paraguai,^186^ em comparação a 1,2 (II 95%, 1,1-1,2) por 100 mil, de acordo com o estudo GBD 2019, e a 2,8 (II 95%, 2,7-3,0) de 20 anos antes, de acordo com a série temporal mais recente do GBD.^36^ Entretanto, as taxas de mortalidade brutas ( **Tabela 5-1** ) mostraram discreto aumento. O número total de mortes foi 2.715 (II 95%, 2.505-2.913) em 2019 e 2.823 em 2021, possivelmente devido a crescimento populacional ( **Tabela 5-1** ), embora tal tendência deva ser cuidadosamente avaliada, considerando-se que, para 2021, apenas estimativas pontuais – em oposição a séries temporais – estejam disponíveis até o momento. A taxa de mortalidade padronizada por idade por DCR no Brasil foi 1,1 (II 95%, 1,0-1,2) por 100 mil,^186^ superpondo-se àquela observada nas estimativas pontuais do GBD 2019, 1,2 (II 95%, 1,1-1,2) por 100 mil. Em 2023, Paraná, Minas Gerais, Bahia, Goiás, Sergipe e Pernambuco foram as unidades federativas com taxas de mortalidade padronizadas por idade por DCR (variando de 1,2 a 1,5 por 100 mil) no mais alto quartil, sendo que Goiás e Paraná apresentaram as mais altas taxas^37 , 186^( **Tabela 5-1 e Figura 5-1A** ).

**Figura 5-1A:**
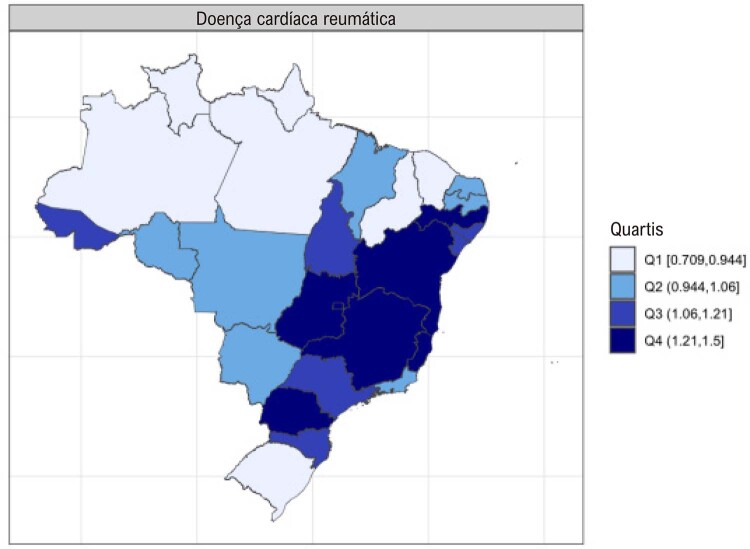
– Doença cardíaca reumática: quartis da taxa de mortalidade padronizada por idade por 100 mil, de acordo com as unidades federativas brasileiras. Dados derivados do Estudo Global Burden of Disease 2021. ^48^

•Uma vez mais, para tais estimativas, a falta de dados primários sistematizados pode ter levado a imprecisões, considerando que o Paraná tem um dos maiores índices sociodemográficos do Brasil. Por outro lado, três estados da região Nordeste, a mais pobre do país, também estão nessa lista.

### Doença Valvar do Coração Não Reumática

•De acordo com o estudo GBD 2021, as taxas de mortalidade padronizadas por idade atribuíveis

•a DVNR específicas da América Latina tropical foram: 0,7 (II 95%, 0,6-0,7) por 100 mil por doença valvar mitral degenerativa não reumática, 1,6 (II 95%, 1,4-1,7) por 100 mil por doença valvar aórtica calcífica não reumática e <0,1 [0,03 (II 95%, 0,026-0,034) por 100 mil] para outras DVNR ( **Tabela 5-1** ).^186^ Os números totais estimados de fatalidades em 2021 foram 1.761, 4.059 e 75 por doença valvar mitral degenerativa não reumática, doença valvar aórtica calcífica não reumática e outras DVNR, respectivamente ( **Tabela 5-1** ). Tais dados reforçam as tendências das estimativas recentes de maior mortalidade por doença valvar aórtica não reumática, como visto na análise do GBD 2019.^4 , 36^ Essa observação, além das tendências temporais prévias, está alinhada ao perfil demográfico do país, com envelhecimento populacional e crescente impacto dos fatores de risco cardiovascular.^189^

•Rio Grande do Sul, Santa Catarina, Paraná, São Paulo, Espírito Santo, Distrito Federal e Amapá foram as unidades federativas com taxas de mortalidade padronizadas por idade para doença valvar aórtica calcífica não reumática no mais alto quartil (variação de 1,65 a 2,42 por 100 mil) ( **Tabela 5-2 e Figura 5-2A** ).^186^

**Tabela 5-2:** – Métrica relacionada às doenças valvares do coração (endocardite infecciosa, doenças valvares reumáticas e não reumáticas)

	DALYs por 100 mil				Mortes por 100 mil	
Local	Estimativa	IC 95% Inferior	IC 95% Superior	Estimativa	IC 95% Inferior	IC 95% Superior
**Endocardite infecciosa**						
Acre	47,58	44,62	52,12	1,44	1,35	1,57
Alagoas	39,68	35,65	43,64	1,20	1,07	1,32
Amapá	40,17	35,89	45,96	1,25	1,12	1,43
Amazonas	27,73	25,52	29,70	0,87	0,78	0,94
Bahia	36,24	31,65	40,22	1,03	0,89	1,15
Brasil *	34,42	32,74	36,17	1,11	1,03	1,18
Ceará	29,70	26,16	33,78	0,93	0,80	1,07
Distrito Federal	27,51	24,92	30,41	1,05	0,94	1,15
Espírito Santo	35,11	31,16	38,36	1,10	0,97	1,20
Goiás	36,46	32,77	40,25	1,21	1,10	1,34
Maranhão	27,52	23,78	30,90	0,82	0,72	0,93
Mato Grosso	34,07	30,33	37,53	1,07	0,95	1,16
Mato Grosso do Sul	38,25	34,19	41,61	1,23	1,11	1,34
Minas Gerais	40,02	36,43	43,26	1,30	1,16	1,41
Pará	32,72	28,59	37,08	0,99	0,84	1,12
Paraíba	29,60	26,64	32,13	0,93	0,84	1,01
Paraná	29,20	26,74	31,85	0,97	0,89	1,06
Pernambuco	27,78	24,90	30,46	0,88	0,78	0,98
Piauí	30,78	26,89	34,85	0,93	0,81	1,05
Rio de Janeiro	39,18	35,82	43,74	1,24	1,12	1,39
Rio Grande do Norte	30,24	26,88	33,56	0,91	0,81	1,01
Rio Grande do Sul	31,25	28,39	34,37	1,06	0,96	1,17
Rondônia	31,64	28,11	36,34	0,99	0,88	1,14
Roraima	30,30	25,93	35,05	0,98	0,84	1,14
Santa Catarina	33,69	30,04	36,88	1,16	1,03	1,28
São Paulo	34,97	31,42	38,19	1,20	1,07	1,33
Sergipe	34,86	31,84	38,21	1,09	0,99	1,19
Tocantins	32,47	28,27	37,01	1,02	0,89	1,16
**Doença valvar aórtica calcífica não reumática**						
Acre	25,58	23,44	28,45	1,28	1,14	1,40
Alagoas	27,35	24,63	30,74	1,31	1,16	1,48
Amapá	33,36	29,12	38,11	1,66	1,46	1,86
Amazonas	31,56	28,41	34,89	1,51	1,34	1,65
Bahia	27,59	24,20	30,69	1,26	1,10	1,40
Brasil *	31,78	29,43	34,01	1,63	1,44	1,73
Ceará	24,40	20,83	27,98	1,20	1,01	1,38
Distrito Federal	31,07	27,90	34,17	1,91	1,69	2,08
Espírito Santo	32,92	28,97	36,35	1,70	1,50	1,87
Goiás	33,39	30,52	36,86	1,64	1,44	1,80
Maranhão	24,89	21,43	28,84	1,20	1,06	1,37
Mato Grosso	29,35	26,49	32,44	1,43	1,26	1,57
Mato Grosso do Sul	32,11	29,01	35,47	1,60	1,42	1,79
Minas Gerais	30,41	27,23	33,45	1,51	1,31	1,66
Pará	27,56	24,18	32,02	1,32	1,09	1,54
Paraíba	20,74	18,71	22,92	0,99	0,88	1,10
Paraná	37,98	34,80	41,87	1,97	1,76	2,19
Pernambuco	29,92	27,05	33,17	1,50	1,30	1,68
Piauí	20,76	18,08	23,72	0,98	0,87	1,12
Rio de Janeiro	28,92	25,88	32,36	1,48	1,26	1,65
Rio Grande do Norte	25,42	22,54	28,36	1,24	1,07	1,36
Rio Grande do Sul	39,05	35,00	42,63	2,15	1,92	2,37
Rondônia	28,14	24,75	31,95	1,38	1,20	1,57
Roraima	31,14	26,92	36,69	1,64	1,39	1,88
Santa Catarina	43,17	38,26	47,68	2,42	2,09	2,65
São Paulo	35,93	32,37	40,13	1,94	1,69	2,18
Sergipe	19,87	17,77	21,78	0,96	0,85	1,05
Tocantins	28,34	24,92	32,19	1,40	1,22	1,60
**Doença valvar mitral degenerativa não reumática**						
Acre	14,41	13,37	15,79	0,57	0,52	0,63
Alagoas	18,97	17,01	21,30	0,72	0,64	0,83
Amapá	18,71	16,30	20,87	0,75	0,65	0,83
Amazonas	13,18	11,76	14,67	0,52	0,46	0,58
Bahia	16,09	14,11	18,14	0,58	0,51	0,65
Brasil *	17,25	16,28	18,18	0,70	0,64	0,74
Ceará	16,71	14,17	19,17	0,67	0,57	0,77
Distrito Federal	15,56	14,05	17,24	0,75	0,68	0,84
Espírito Santo	21,74	19,27	24,21	0,89	0,78	1,00
Goiás	17,83	16,02	19,86	0,72	0,64	0,80
Maranhão	13,13	11,26	15,07	0,50	0,43	0,57
Mato Grosso	16,71	15,08	18,41	0,66	0,58	0,72
Mato Grosso do Sul	18,98	17,04	20,77	0,75	0,67	0,83
Minas Gerais	15,04	13,60	16,39	0,60	0,52	0,66
Pará	17,90	15,33	20,93	0,70	0,59	0,82
Paraíba	12,22	10,95	13,53	0,47	0,41	0,52
Paraná	19,80	17,75	21,84	0,83	0,74	0,92
Pernambuco	21,79	19,54	23,85	0,87	0,77	0,96
Piauí	14,16	12,40	16,08	0,54	0,47	0,61
Rio de Janeiro	15,24	13,65	17,04	0,60	0,53	0,67
Rio Grande do Norte	13,30	11,81	14,69	0,51	0,45	0,57
Rio Grande do Sul	17,66	16,09	19,36	0,76	0,66	0,83
Rondônia	14,39	12,58	16,53	0,58	0,50	0,66
Roraima	11,52	9,72	13,71	0,46	0,38	0,55
Santa Catarina	18,37	16,35	20,34	0,80	0,71	0,89
São Paulo	18,97	16,94	21,13	0,81	0,72	0,91
Sergipe	13,67	12,25	15,05	0,54	0,49	0,60
Tocantins	21,95	19,47	24,99	0,90	0,79	1,02
**Outras doenças valvares não reumáticas**						
Acre	1,07	0,88	1,27	0,04	0,04	0,05
Alagoas	0,60	0,47	0,72	0,02	0,02	0,03
Amapá	0,99	0,80	1,22	0,04	0,03	0,05
Amazonas	0,70	0,57	0,85	0,03	0,02	0,03
Bahia	0,67	0,58	0,81	0,02	0,02	0,03
Brasil *	0,79	0,71	0,88	0,03	0,03	0,03
Ceará	1,19	0,98	1,45	0,05	0,04	0,06
Distrito Federal	0,71	0,59	0,86	0,03	0,03	0,04
Espírito Santo	0,98	0,80	1,16	0,04	0,03	0,05
Goiás	0,87	0,74	1,06	0,03	0,03	0,04
Maranhão	1,14	0,96	1,35	0,04	0,04	0,05
Mato Grosso	0,66	0,55	0,84	0,03	0,02	0,03
Mato Grosso do Sul	0,32	0,26	0,38	0,01	0,01	0,02
Minas Gerais	0,69	0,57	0,85	0,03	0,02	0,03
Pará	1,81	1,45	2,18	0,07	0,05	0,09
Paraíba	0,90	0,76	1,05	0,03	0,03	0,04
Paraná	1,06	0,87	1,29	0,04	0,04	0,05
Pernambuco	0,84	0,69	1,01	0,03	0,03	0,04
Piauí	0,77	0,62	0,96	0,03	0,02	0,04
Rio de Janeiro	0,44	0,37	0,53	0,02	0,01	0,02
Rio Grande do Norte	1,14	0,95	1,35	0,04	0,04	0,05
Rio Grande do Sul	0,90	0,77	1,08	0,04	0,03	0,04
Rondônia	0,92	0,75	1,09	0,04	0,03	0,04
Roraima	1,89	1,55	2,25	0,07	0,06	0,09
Santa Catarina	0,50	0,40	0,59	0,02	0,02	0,03
São Paulo	0,60	0,51	0,76	0,02	0,02	0,03
Sergipe	0,65	0,55	0,77	0,03	0,02	0,03
Tocantins	1,56	1,32	1,81	0,06	0,05	0,07
**Doença cardíaca reumática**						
Acre	93,71	72,25	120,60	1,13	1,02	1,25
Alagoas	98,41	75,74	123,81	1,18	0,99	1,37
Amapá	83,57	61,54	110,32	0,81	0,71	0,92
Amazonas	79,17	57,75	106,01	0,71	0,54	0,80
Bahia	102,46	77,61	134,08	1,22	1,06	1,41
Brasil*	93,66	70,92	122,70	1,11	0,97	1,20
Ceará	85,65	62,72	115,53	0,82	0,71	0,95
Distrito Federal	97,24	74,63	129,24	1,50	1,22	1,71
Espírito Santo	96,43	74,58	125,54	1,23	1,01	1,42
Goiás	102,86	80,71	133,75	1,45	1,19	1,65
Maranhão	92,06	69,06	120,67	1,02	0,85	1,16
Mato Grosso	87,12	64,35	116,87	0,95	0,75	1,09
Mato Grosso do Sul	89,44	66,95	120,27	0,95	0,80	1,08
Minas Gerais	98,87	74,13	128,93	1,33	1,16	1,46
Pará	86,80	63,37	115,89	0,89	0,77	1,03
Paraíba	92,53	69,89	119,40	1,02	0,86	1,16
Paraná	99,95	76,58	127,95	1,36	1,21	1,56
Pernambuco	103,62	80,45	131,52	1,32	1,08	1,54
Piauí	84,69	60,53	112,98	0,76	0,65	0,86
Rio de Janeiro	91,72	68,77	121,04	1,03	0,83	1,20
Rio Grande do Norte	94,03	71,73	123,51	1,06	0,90	1,19
Rio Grande do Sul	84,38	61,76	112,28	0,83	0,73	0,92
Rondônia	88,51	66,25	117,17	1,00	0,86	1,15
Roraima	85,87	65,88	110,36	0,94	0,80	1,11
Santa Catarina	91,62	69,41	123,48	1,10	0,95	1,24
São Paulo	90,94	67,73	122,63	1,07	0,89	1,22
Sergipe	98,83	74,55	135,42	1,19	0,98	1,34
Tocantins	93,75	71,02	123,10	1,15	0,99	1,32

* Estimativas para o Brasil. Fonte: Dados derivados do Global Burden of Disease Collaborative Network. Global Burden of Disease (GBD) Cardiovascular Burden Estimates 1990 and 2021, Institute for Health Metrics and Evaluation, University of Washington.48

**Figura 5-2A:**
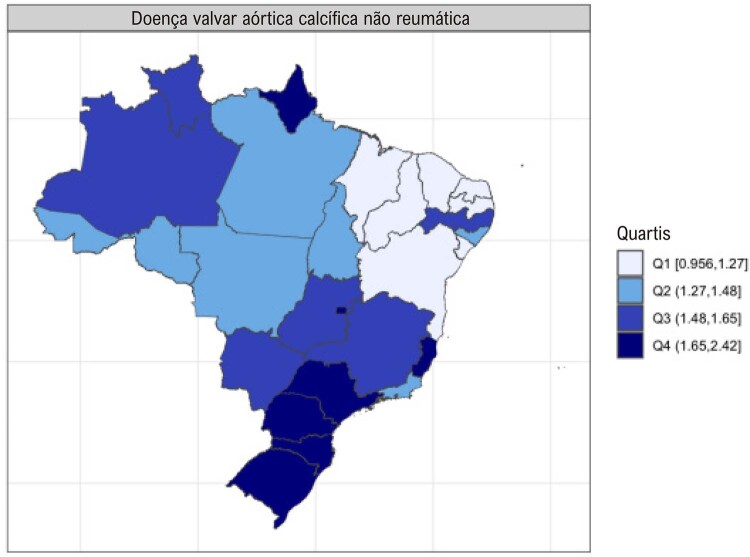
– Doença valvar aórtica calcífica não reumática: quartis da taxa de mortalidade padronizada por idade por 100 mil, de acordo com as unidades federativas brasileiras. Dados derivados do Estudo Global Burden of Disease 2021. ^48^

•Quanto à doença valvar mitral degenerativa não reumática, as taxas de mortalidade padronizadas por idade foram mais altas no Rio Grande do Sul, Santa Catarina, Paraná, São Paulo, Espírito Santo, Tocantins e Pernambuco (variação de 0,754 a 0,896 por 100 mil) ( **Tabela 5-2 e Figura 5-3A** ).^186^

**Figura 5-3A:**
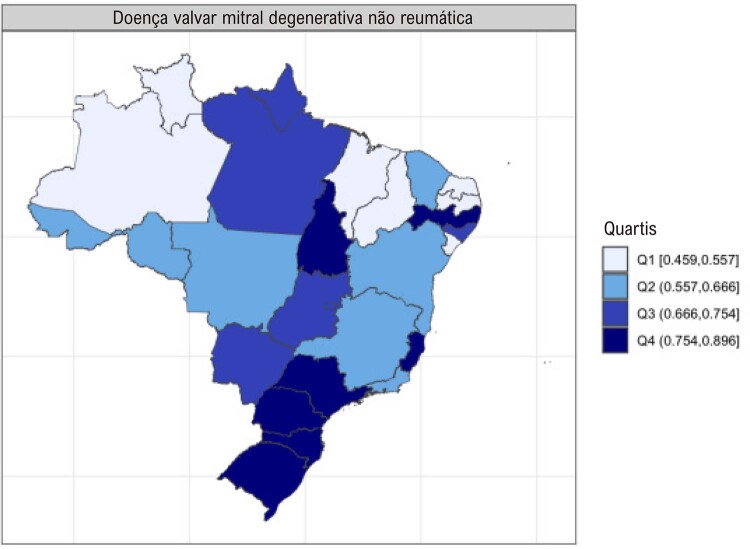
– Doença valvar mitral degenerativa não reumática: quartis da taxa de mortalidade padronizada por idade por 100 mil, de acordo com as unidades federativas brasileiras. Dados derivados do Estudo Global Burden of Disease 2021. ^48^

•Quanto às outras DVNR, Roraima, Pará, Maranhão, Tocantins, Ceará, Acre e Paraná foram as unidades federativas com as mais altas estimativas das taxas de mortalidade padronizadas por idade (variação de 0,04 a 0,07 por 100 mil) ( **Tabela 5-2 e Figura 5-4A** ).^186^

**Figura 5-4A:**
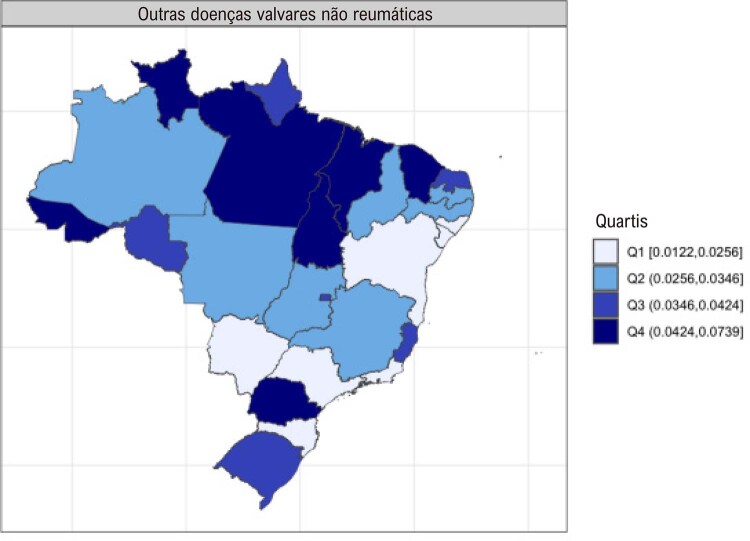
– Outras doenças valvares não reumáticas: quartis da taxa de mortalidade padronizada por idade por 100 mil, de acordo com as unidades federativas brasileiras. Dados derivados do Estudo Global Burden of Disease 2021. ^48^

•As crescentes taxas de mortalidade por DVNR nas idades avançadas contrastam com as tendências observadas para a DCR, possivelmente refletindo a maior prevalência e, consequentemente, mortalidade nos grupos etários >70 anos para as doenças aórtica e mitral.^34 , 36 , 186^ Além da necessidade de atenção específica, a partir da perspectiva da saúde pública, essas tendências indicam um significativo crescimento das despesas relacionadas às doenças valvares degenerativas, especialmente aquelas ligadas aos novos dispositivos, como TAVI e reparo da válvula mitral *edge-to-edge* , e ao cuidado continuado dos idosos portadores de DVC.^189 , 191^

## Carga de Doença

### Doença Cardíaca Reumática

•De acordo com dados do GBD 2021, a taxa de DALYs padronizada por idade atribuível a DCR aumentou significativamente para 93,7 (II 95%, 70,9-122,7) por 100 mil no Brasil ( **Tabela 5-2** ), em oposição à tendência de queda observada de 1990 a 2019, 144,6 (II 95%, 126,8-167,3) e 79,3 (II 95%, 61,6-102,6) por 100 mil, respectivamente.^37 , 186^ Mais uma vez, essa tendência requer interpretação cuidadosa, devido à ausência de séries temporais até as estimativas pontuais de 2021, além das recentes mudanças na modelagem de DCR, em especial com a incorporação de *inputs* adicionais, como a prevalência de doença latente, que pode impactar consideravelmente as estimativas em locais onde são realizados programas de triagem.^180 , 181^ Minas Gerais, Goiás, Paraná, Bahia, Sergipe, Alagoas e Pernambuco foram as unidades federativas com as mais altas taxas de DALYs padronizadas por idade no Brasil, ocupando o quartil superior (variação de 97,8 a 104,0 por 100 mil) ( **Figura 5-1B** ).^186^

**Figura 5-1B:**
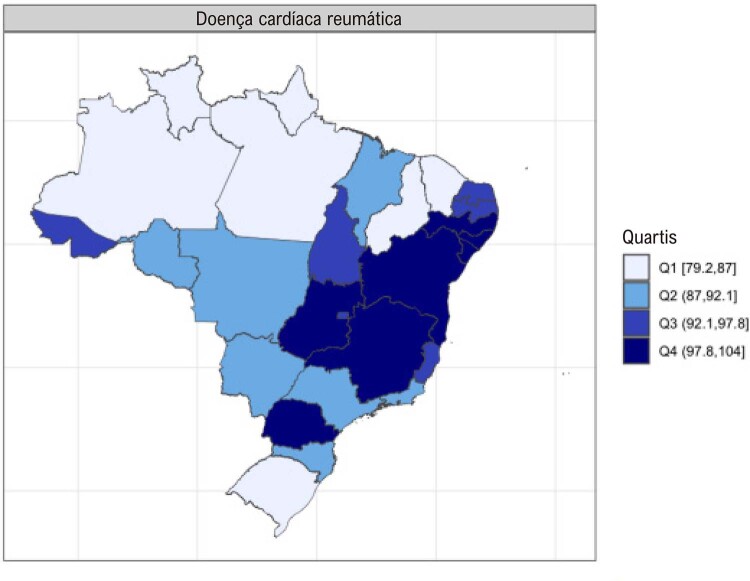
– Doença cardíaca reumática: quartis da taxa de DALYs padronizada por idade por 100 mil, de acordo com as unidades federativas brasileiras. Dados derivados do Estudo Global Burden of Disease 2021. ^48^

Doença Valvar do Coração Não Reumática

•De acordo com o estudo GBD, a taxa de DALYs padronizada por idade atribuível a doença valvar aórtica calcífica não reumática em 2021 foi 31,8 (II 95%, 29,4-34,0) por 100 mil, consideravelmente maior do que aquela observada para doença valvar mitral degenerativa não reumática, 17,2 (II 95%, 16,3-18,2) por 100 mil, e em especial do que a observada para outras DVNR, levando a uma carga bem mais baixa, 0,8 (II 95%, 0,7-0,9) por 100 mil.^186^ Entretanto, as taxas gerais de DALYs padronizadas por idade associadas com DVNR continuam a cair, mostrando uma tendência decrescente contínua quando comparada à última série publicada: 62,8 (II 95%, 60,3-65,2) por 100 mil em 1990 e 44 (II 95%, 40,7-47) por 100 mil em 2019 ( **Tabela 5-2** ).^4 , 36^

•Rio Grande do Sul, Santa Catarina, Paraná, São Paulo, Espírito Santo, Goiás e Amapá foram as unidades federativas com taxas de DALYs padronizadas por idade por doença valvar aórtica calcífica não reumática por 100 mil no mais alto quartil (variação de 32,5 a 43,2 por 100 mil) ( **Tabela 5-2 e Figura 5-2B** ). Importante notar que, seis dessas sete unidades federativas ocupam o mais alto estrato sociodemográfico brasileiro, podendo, pelo menos em parte, refletir as tendências na composição etária – em última análise, envelhecimento populacional – e, consequentemente, a carga dos fatores de risco.^189 , 191 , 193^

**Figura 5-2B:**
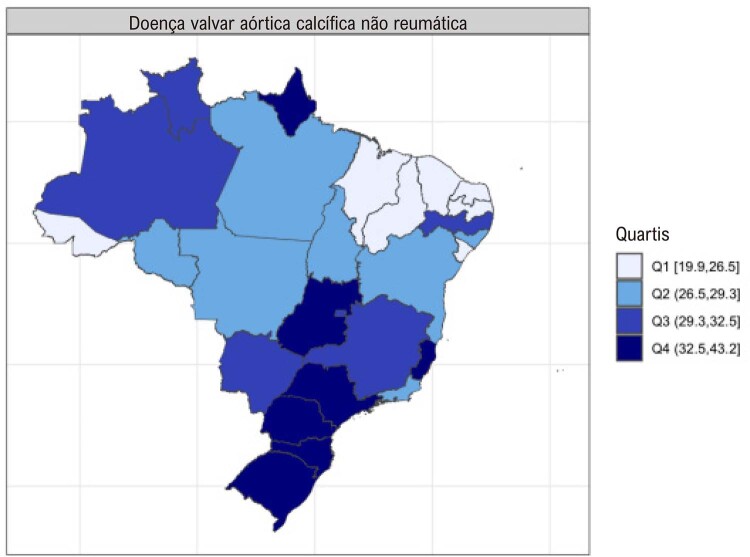
– Doença valvar aórtica calcífica não reumática: quartis da taxa de DALYs padronizada por idade por 100 mil, de acordo com as unidades federativas brasileiras. Dados derivados do Estudo Global Burden of Disease 2021. ^48^

•Quanto à doença valvar mitral degenerativa não reumática, as mais altas taxas de DALYs padronizadas por idade foram observadas nas unidades federativas do Paraná, São Paulo, Mato Grosso do Sul, Espírito Santo, Tocantins e Pernambuco (variação de 18,8 a 22,0 por 100 mil) ( **Tabela 5-2 e Figura 5-3B** ),^186^ uma tendência similar à da doença valvar aórtica não reumática, exceto em Pernambuco, que contrasta com as outras unidades federativas em termos de *status* sociodemográfico e acesso e utilização de cuidado de saúde ótimo.

**Figura 5-3B:**
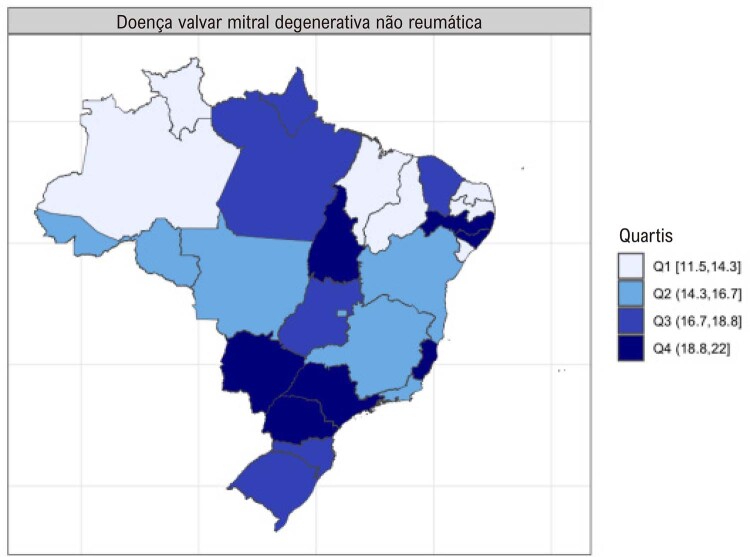
– Doença valvar mitral degenerativa não reumática: quartis da taxa de DALYs padronizada por idade por 100 mil, de acordo com as unidades federativas brasileiras. Dados derivados do Estudo Global Burden of Disease 2021. ^48^

•Quanto às outras DVNR, Roraima, Pará, Maranhão, Tocantins, Ceará e Rio Grande do Norte foram as unidades federativas com taxas de DALYs padronizadas por idade no maior quartil (variação de 1,06 a 1,89 por 100 mil) ( **Tabela 5-2 e Figura 5-4B** ).^37 , 186^

**Figura 5-4B:**
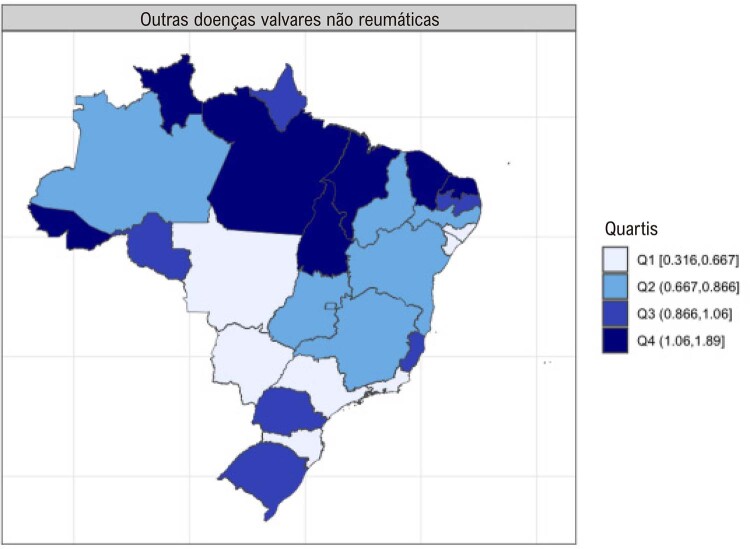
– Outras doenças valvares não reumáticas: quartis da taxa de DALYs padronizada por idade por 100 mil, de acordo com as unidades federativas brasileiras. Dados derivados do Estudo Global Burden of Disease 2021. ^48^

## Doenças Associadas

### Endocardite

•A taxa de mortalidade padronizada por idade associada com EI na América Latina tropical foi 1,1 (IC 95%, 1,0-1,2) por 100 mil em 2021, resultando em aproximadamente 2.775 fatalidades. De acordo com o GBD 2021, a EI apresentou o maior aumento percentual, desde 1990, nas taxas de mortalidade padronizadas por idade com doença cardiovascular como causa específica (aumento estimado de 35,4%). A taxa de mortalidade foi a mesma para o Brasil, assim como a tendência temporal ( **Tabela 5-1** ).^186^

•O número total estimado de casos prevalentes de EI na região foi 15.053 em 2021, resultando em uma taxa de prevalência de 6,7 (II 95%, 5,6-7,7) por 100 mil. Rio de Janeiro, Minas Gerais, Goiás, Mato Grosso do Sul, Amapá e Acre foram as unidades federativas brasileiras que apresentaram taxas de mortalidade padronizadas por idade por EI no mais alto quartil (variação de 1,2 a 1,4 por 100 mil) ( **Tabela 5-2 e Figura 5-5A** ),^186^ um padrão discretamente diferente se comparado às estimativas da DCR – uma conhecida causa de endocardite nos jovens.^194^

**Figura 5-5A:**
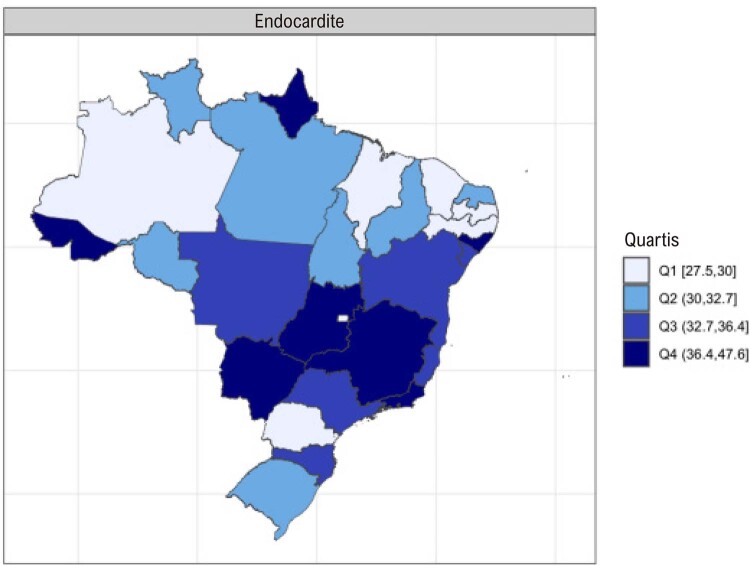
– Endocardite infecciosa: quartis da taxa de mortalidade padronizada por idade por 100 mil, de acordo com as unidades federativas brasileiras. Dados derivados do Estudo Global Burden of Disease 2021. ^48^

•A taxa de DALYs atribuíveis a EI padronizada por idade no Brasil foi 34,4 (II 95%, 32,7-36,2) por 100 mil em 2021. As mesmas unidades federativas do grupo com as mais altas mortalidades por EI, além de Alagoas, apresentavam taxas de DALYs no mais alto quartil no Brasil (variação de 36,4 a 47,6 por 100 mil) ( **Tabela 5-2 e Figura 5-5B** ).^186^

**Figura 5-5B:**
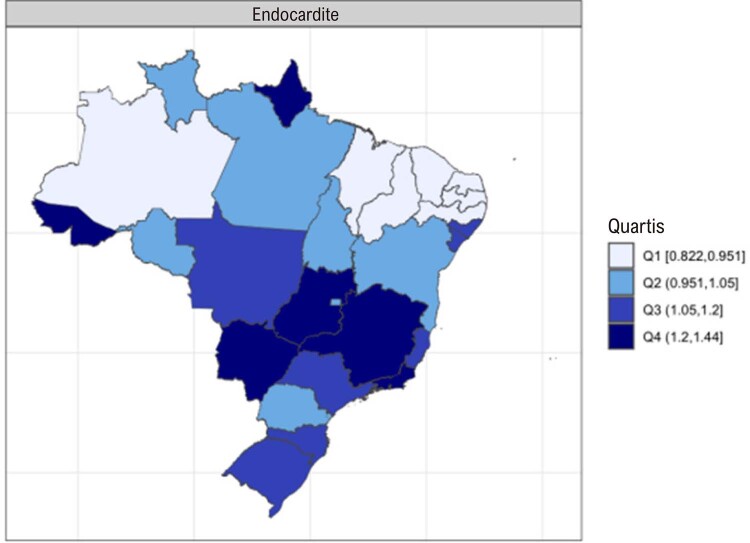
– Endocardite infecciosa: quartis da taxa de DALYs padronizada por idade por 100 mil, de acordo com as unidades federativas brasileiras. Dados derivados do Estudo Global Burden of Disease 2021. ^48^

•Um estudo recente no Brasil observou taxa de mortalidade de 22,3% em um grupo de 221 pacientes em Minas Gerais^195^ hospitalizados com EI, principalmente por infecção por *Staphylococcus sp* . Uma revisão sistemática de EI após TAVI por estenose aórtica não reumática observou mortalidade hospitalar estimada em 29,5% e mortalidade em seguimento de médio e longo prazo de 29,9%. A incidência de EI após TAVI foi 3,25%, mas associada com maior mortalidade e complicações potencialmente ameaçadoras à vida. Entretanto, em países de alta renda, há tendência de redução da mortalidade em pacientes com EI.^196^ Tal tendência não foi ainda observada no Brasil, embora estimativas primárias sólidas sejam escassas.^189^

## Utilização e Custo da Atenção à Saúde

•O número de hospitalizações clínicas associadas com DVC diminuiu após a pandemia de COVID-19, passando de 2.289 (1,09 por 100 mil) em 2019 para 1.536 (0,72 por 100 mil) em 2020. Uma discreta recuperação foi observada em 2021: 1.597 hospitalizações clínicas (0,75 por 100 mil).^49^

•De acordo com a base de dados administrativos do SUS, o total de despesas brutas (reembolso) com hospitalização para tratamento clínico de DVC no Brasil diminuiu de R$ 1.999.540 ($397.355) em 2019 para R$ 1.526.256 ($303.913) em 2020, com uma recuperação parcial para R$ 1.465.800 ($307.346) em 2021. Em comparação a uma série temporal de 3 anos (2017-2019), a redução nos anos de pandemia em dólares americanos foi de cerca de 19,1% ( **Tabelas 1-1 até 1-7 e Figuras 1-1 até 1-7** ).^49^

•Além disso, o número de cirurgias abertas por DVC diminuiu significativamente após a pandemia de COVID-19, passando de 12.771 (6,08 por 100 mil) em 2019 a 9.198 (4,34 por 100 mil) em 2020 e 8.759 (4,10 por 100 mil) em 2021.^49^

•Quanto a outros procedimentos de valvoplastia (não incluindo VMPB), o número de hospitalizações também caiu inicialmente, passando de 450 (0,21 por 100 mil) em 2019 para 399 (0,19 por 100 mil) em 2020 (-9,5%). Em 2021, houve retorno aos valores prévios de 470 hospitalizações anuais (0,22 por 100 mil), podendo refletir uma demanda reprimida por procedimentos adiados durante a pandemia.^49^

•Fenômeno similar foi observado quanto ao número de VMPB, que diminuiu de 195 (0,09 por 100 mil) em 2019 para 129 (0,06 por 100 mil) em 2020 e para 159 (0,07 por 100 mil) em 2021.^49^Esses números são impressionantes para o Brasil e merecem a consideração das autoridades de saúde, uma vez que o procedimento é principalmente realizado em instituições públicas de ensino, que requerem um orçamento complementar, considerando o atual reembolso por procedimento bem abaixo dos preços de mercado dos dispositivos necessários (em especial, o sistema de balão Inoue).

•Esses padrões mencionados estão associados a restrições para cirurgias e procedimentos eletivos, além da necessidade de maior disponibilidade de quartos hospitalares para pacientes com COVID-19, com reorganização dos serviços de saúde. No pico da pandemia, as restrições tenderam a ser ainda mais rígidas e associadas com a concorrência por leitos hospitalares para pacientes com COVID-19 muito graves. Como os procedimentos intervencionistas percutâneos resultam em mais curta permanência hospitalar, em 2021, houve discreto aumento desses procedimentos em comparação às cirurgias valvares abertas ( **Tabelas 1-1 até 1-7 e Figuras 1-1 até 1-7** ).^37 , 49 , 182^

•Assim, as despesas totais com hospitalização para cirurgia valvar cardíaca aberta caíram de R$ 187.382.032 ($42.835.735) em 2019 para R$ 137.383.131 ($30.108.266) em 2020 (-29,8%) e mantiveram a tendência de queda em 2021 [R$ 131.110.929 ($27 491 081)] ( Figura 5-6 ). Em 2021, entretanto, os impactos da pandemia ainda estavam presentes nos serviços de saúde, em especial nos cenários de alta complexidade. Além dos surtos das variantes do coronavírus, como a ômicron, os serviços de saúde ainda não estavam totalmente recuperados nem adequadamente estruturados para retomar as cirurgias eletivas ( **Tabelas 1-1 até 1-7 e Figuras 1-1 até 1-7** ).^49^

**Figura 5-6:**
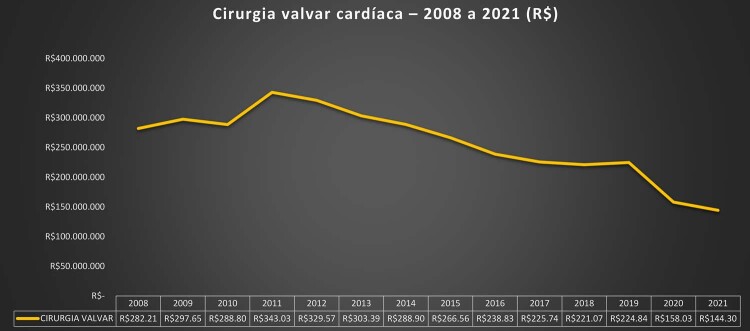
– Despesas ajustadas para a inflação para cirurgia valvar cardíaca no SUS, de 2008 a 2021 (Moeda: Reais brasileiros). Dados do Ministério da Saúde do Brasil – Sistema de Informações Hospitalares do Sistema Único de Saúde (SIH/SUS). ^49^

•Entretanto, as despesas públicas associadas com outros procedimentos de valvoplastia inicialmente caíram, passando de R$ 1.959.571 ($447.960) em 2019 para R$ 1.851.963 ($397.978) em 2020 (-11,2%), mas, em 2021, subiram para R$ 2.175.077 ($456.066.06), acima dos valores basais da série temporal pré-pandêmica ( **Figura 5-7** ).^49^

**Figura 5-7:**
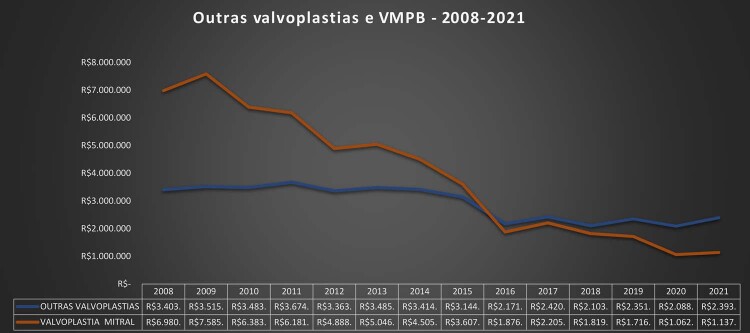
– Despesas ajustadas para a inflação para outras valvoplastias e valvoplastia mitral percutânea por balão (VMPB) no SUS, de 2008 a 2021 (Moeda: Reais brasileiros). Dados do Ministério da Saúde do Brasil – Sistema de Informações Hospitalares do Sistema Único de Saúde (SIH/SUS). ^49^

•O mesmo ocorreu com as despesas totais brutas com hospitalização para VMPB, que caíram de R$ 1.430.166 ($326.937) em 2019 para R$ 923.776 ($202.450) em 2020, tendo se recuperado parcialmente em 2021, R$ 1.033.888 ($216.783) ( **Figura 5-7** ).^49^ Além da retomada dos procedimentos cancelados devido à pandemia, isso reflete o aumento dos valores reembolsados para os dispositivos, que começou em meados de 2021. Isso é muito importante para a VMPB, pois vários hospitais públicos e privados não mais realizam tais procedimentos, devido ao reembolso insuficiente. Isso limita ainda mais o acesso de pacientes – em especial aqueles com DCR – ao cuidado ótimo, em especial nas localidades remotas sem hospitais universitários. Revisão das tabelas de reembolso para dispositivos cardiovasculares deve estar entre as prioridades para a elaboração de políticas na década de 2020.

## •Doença Valvar do Coração e Pandemia de COVID-19

•Pacientes com doença valvar avançada tiveram impacto desfavorável durante a epidemia de COVID-19. Isso foi observado principalmente naqueles com indicação de tratamento cirúrgico ou intervencionista, os mais idosos e aqueles com comprometimento das condições físicas.^37^

•Um estudo multicêntrico mostrou que pacientes com DVC grave e COVID-19 apresentavam piores desfechos clínicos de curto prazo, com mortalidade geral em 30 dias de 41,8%, muito mais alta do que a de outras séries publicadas.^197^ Nessa coorte, a idade média foi alta (80,0 ± 9,7 anos), e os tipos mais frequentes de DVC foram estenose aórtica (54,4%) e regurgitação mitral grave (20,6%), diferentemente da realidade brasileira, em especial nas clínicas do SUS.^4 , 197^ A maioria dos pacientes tinha sintomas pré-existentes relacionados com suas DVC, como dispneia (83,1%), dor torácica (19,1%) e síncope (8,1%) antes da infecção de COVID-19. Na admissão, dispneia (64,9%), tosse (57,5%) e febre (41,3%), entre outras manifestações respiratórias graves, foram sintomas relacionados à COVID-19.^197^ Mortalidade mais baixa (11,1%) foi observada nos pacientes com idade inferior a 80 anos e com suas DVC previamente tratadas com intervenções cirúrgicas. Ao contrário, pior prognóstico (mortalidade de 59,9%) foi observado naqueles com DVC grave, mais de 80 anos de idade e não tratados,^197^ indicando a necessidade de programas estruturados com foco na preparação e manejo de pacientes idosos para procedimentos valvares, fornecendo a provedores educação para a saúde em DCR e DVNR. Importante notar que regurgitação mitral grave (54,4%) e estenose aórtica grave (42,6%) – as doenças mais prevalentes – apresentaram mortalidade maior do as outras DVC (29,7%).^197^

•Esses dados mostrando o marcante impacto da COVID-19 na mortalidade por DVC cirúrgica em comparação a outras séries históricas e dados administrativos reforçam a necessidade de preparação específica dos serviços de saúde para futuros surtos.^37 , 197^ Mesmo com o fim da pandemia, após quase 7 milhões de mortes e mais de 760 milhões de casos confirmados, a COVID-19 ainda requer atenção devido a seus efeitos devastadores diretos e indiretos na saúde das pessoas, incluindo aquelas com doenças cardiovasculares.

## Perspectivas

•Como previamente ressaltado (2020/2021),^2 , 4^ reforçamos a importância de se melhorar a coleta de dados, a pesquisa e a infraestrutura de cuidado em saúde relacionadas à DVC no Brasil. Após a pandemia, enquetes em saúde, registros e triagem específica, assim como programas de diagnóstico precoce, devem ser retomados e reforçados. As iniciativas em pesquisa devem focar os padrões de DVC com mais alto impacto na saúde populacional, como as sequelas tardias de DCR e a crescente carga de doença valvar aórtica degenerativa.

•Considerando a falta de dados primários sobre a epidemiologia da DVC no Brasil, assim como o impacto da pandemia nas pesquisas em andamento, reunir dados precisos e atualizados sobre prevalência, incidência e tendências da DVC deve ser o foco dos setores público e privado. Dados atualizados são essenciais para o planejamento do cuidado em saúde, alocação de recursos e compreensão da carga de doença.

•A coleta de dados administrativos e a codificação específica para procedimentos cirúrgicos e intervencionistas relacionados a DVC são essenciais. Esses códigos específicos, evitando a necessidade de cruzamento com os códigos da CID, podem auxiliar os profissionais de saúde e elaboradores de políticas públicas a melhor identificar a prevalência e as características da doença, assim como o entendimento detalhado da carga econômica. A identificação de variáveis, como o tipo de envolvimento valvar, disfunção valvar, tipo de prótese, etiologia e associação com doenças sistêmicas, é importante para um cuidado customizado.

•O estabelecimento de registros nacionais para DVC e procedimentos é um passo significativo a se considerar. Os registros podem servir como recursos valiosos para pesquisadores, clínicos e gestores de cuidados em saúde. A coleta abrangente e padronizada de dados sobre as DVC e intervenções pode auxiliar a melhorar os desfechos dos pacientes e a qualidade do cuidado.

•Várias iniciativas foram implementadas no âmbito da pesquisa e da atenção à saúde para desenvolver inteligência artificial para diagnóstico automatizado e sinalização de anormalidades nos exames de imagem cardiovasculares. Algumas dessas estratégias combinadas foram desenvolvidas no Brasil em colaboração com grupos internacionais^198 - 200^e, com mais pesquisa, podem disponibilizar triagem e diagnóstico no ponto de atendimento em áreas com limitação de pessoal e recursos.

•Tais recomendações podem auxiliar a melhorar a infraestrutura de saúde, os dados de pesquisa e o cuidado ao paciente no sistema de saúde brasileiro. Ao abordar tais questões, o Brasil pode melhor responder ao desafio das DVC e aprimorar a saúde cardiovascular da sua população. A customização da prestação de cuidado de saúde com base em epidemiologia e impacto, priorizando a alocação de recursos, é estratégica para impulsionar a eficácia, especialmente no setor público.

## CAPÍTULO 6 – FIBRILAÇÃO ATRIAL E FLUTTER ATRIAL

### CID-10 I48

#### Ver Tabelas 6-1 e 6-2 e Figuras 6-1 e 6-2

**Table:** Abreviaturas Usadas no Capítulo 6

wsxq	Acidente Vascular Cerebral
BNP	Peptídeo Natriurético Cerebral
CID-10	Classificação Estatística Internacional de Doenças e Problemas Relacionados à Saúde, 10a Revisão
DALYs	Anos de vida perdidos ajustados por incapacidade (do inglês, Disability-Adjusted Life-Years)
DCh	Doença de Chagas
DOAC	Anticoagulante oral direto (do Inglês, Direct Oral Anticoagulant)
ECG	Eletrocardiograma
ELSA-Brasil	Estudo Longitudinal da Saúde do Adulto
FA	Fibrilação Atrial
GARFIELD-AF	The Global Anticoagulant Registry in the FIELD-AF
GBD	Global Burden of Disease
GIRAF	CoGnitive Impairment Related to Atrial Fibrillation
HR	Hazard Ratio
IC	Intervalo de Confiança
II	Intervalo de Incerteza
IMPACT-AF	Multifaceted Intervention to Improve Treatment With Oral Anticoagulants in Atrial Fibrillation
INR	Índice Internacional Normalizado (do inglês, International Normalized Ratio)
NYHA	New York Heart Association
OR	Odds Ratio
PPC	Paridade do Poder de Compra
RECALL	Registro Brasileiro de Fibrilação Atrial Crônica
SUS	Sistema Único de Saúde
TTR	Tempo na Faixa Terapêutica (do inglês, Time in Therapeutic Range)
UF	Unidade Federativa

## Prevalência e Incidência

•A prevalência de FA/ *flutter* atrial varia de acordo com as características do cenário do estudo (estudos de base populacional ou de cuidados de saúde), em particular porque a prevalência de FA está altamente associada com o avançar da idade.

•Dados de estudos de base populacional mostraram prevalência variando de 0,3% a 2,5%. No estudo de coorte ELSA-Brasil, que incluiu 14.424 adultos com ECG válidos (45,8% homens; idade média, 51 anos; faixa etária, 35-74 anos), a prevalência de FA e *flutter* atrial foi 0,3% (homens, 0,5%; mulheres, 0,2%), sendo maior na faixa etária de 65-74 anos (mulheres: OR, 17; IC 95%, 2,1-135,9; homens: OR, 52,3; IC 95%, 3,1-881,8).^201^ Quando autorrelato foi acrescentado à definição de FA no estudo ELSA-Brasil, a prevalência de FA subiu para 2,5%, mas observaram-se diferenças nas características dos pacientes entre FA diagnosticada ao ECG e FA autorrelatada ( **Tabela 6-1** ).^202^ Em estudo transversal com 1.524 idosos em São Paulo, a prevalência de FA ou *flutter* atrial foi 2,4% (homens, 3,9%; mulheres, 2,0%).^203^

**Tabela 6-1:** – Características dos participantes com fibrilação atrial ou flutter na linha de base do estudo ELSA-Brasil de acordo com o escore CHA2DS2 -VASc

FA ou *flutter*							
	Com registro ECG		Apenas autorrelato		Todos os casos de FA ou *flutter*		
	CHA_**2**_DS_**2**_-VASc < 2 (N=16)	CHA_**2**_DS_**2**_-VASc ≥ 2 (N=32)	CHA_**2**_DS_**2**_-VASc < 2 (N=130)	CHA_**2**_DS_**2**_-VASc ≥ 2 (N=153)	CHA_**2**_DS_**2**_-VASc < 2 (N=146)	CHA_**2**_DS_**2**_-VASc ≥ 2 (N=185)	Valor de p
Idade (anos; mediana [P25 - P75])	56,0 [49,5 – 61,2]	67,5 [58,0 – 71,2]	49,5 [45,0 – 56,0]	59,0 [53,0 – 66,0]	50,0 [45,0 – 57,0]	60,0 [53,0 – 68,0]	<0,001 ^**‡**^
Sexo feminino N (%))	3 (18,8%)	15 (46,9%)	60 (46,2%)	97 (63,4%)	63 (43,2%)	112 (60,5%)	0,002 ^**†**^
Doença arterial coronariana (N (%))	0 (0,0%)	7 (21,9%)	2 (1,5%)	64 (41,8%)	2 (1,4%)	71 (38,4%)	<0,001 ^**†**^
AVC (N (%))	0 (0,0%)	1 (3,1%)	0 (0,0%)	7 (4,6%)	0 (0,0%)	8 (4,3%)	0,010 ^**¥**^
Risco de ASCVD em 10 anos > 10% (N (%))	7 (43,8%)	16 (66,7%)	15 (11,7%)	26 (30,6%)	22 (15,3%)	42 (38,5%)	<0,001 ^**†**^
ASCVD prévia ou risco de ASCVD em 10 anos > 10%	7 (43,8%)	24 (75,0%)	17 (13,1%)	94 (61,4%)	24 (16,4%)	118 (63,8%)	<0,001 ^**†**^
Uso de anticoagulantes (N (%))	3 (18,8%)	16 (50,0%)	1 (0,8%)	4 (2,6%)	4 (2,7%)	20 (10,8%)	0,005 ^**¥**^
Uso de agentes antiagregantes (N (%))	2 (12,5%)	5 (15,6%)	2 (1,5%)	43 (28,1%)	4 (2,7%)	48 (25,9%)	<0,001 ^**†**^

Risco de ASCVD em 10 anos definido apenas em participantes sem doença arterial coronariana prévia ou AVC. FA: fibrilação atrial; ASCVD: doença cardiovascular aterosclerótica; AVC: acidente vascular cerebral. Valores de P apresentados para comparação entre os grupos de escore CHA_2_DS_2_-VASc < 2 (N=146) e CHA_2_DS_2_-VASc ≥ 2 (N=185) em todos os casos de AF ou flutter. Testes † qui-quadrado, ‡ Mann-Whitney U ou ¥ exato de Fisher. Fonte: Santos et al. ^202^

•Os centros de telessaúde no Brasil forneceram informação sobre a prevalência de FA e *flutter* atrial com base em ECG da atenção primária.^204 - 206^ No conjunto de dados sobre ECG do sistema de telessaúde de Minas Gerais, que incluiu 1.558.421 indivíduos (idade média, 51±18 anos; 40,2% homens) com ECG realizado entre 2010 e 2017, a prevalência de FA foi 1,33%, maior em homens (1,81% *vs.* 1,02%) e aumentando com a idade (OR 1,08; IC 95% 1,08-1,08), chegando a 7,0% nos octogenários (8,4% em homens *vs.* 5,9% em mulheres).^204^

•Dados de 676.621 ECG (idade média, 51±19 anos; 57,5% mulheres) foram analisados no serviço de telemedicina da Universidade Federal de São Paulo (2009-2016), revelando uma prevalência de FA em 7 anos de 2,2% e uma prevalência de FA projetada para 2025 no Brasil de 1,7%.^205^

•O registro de base hospitalar GARFIELD-AF incluiu pacientes ≥18 anos com FA diagnosticada nas 6 semanas anteriores e pelo menos um fator de risco adicional para AVC. No Brasil, 41 *sites* incluíram 1.065 pacientes com FA não valvar entre 2010 e 2014 (idade média, 68±13 anos; 55% homens). A prevalência dos tipos de FA foi a seguinte: primeiro episódio, 52%; paroxística, 25%; persistente, 14%; e permanente, 8%.^207^

## Mortalidade

•No Estudo GBD 2021, a taxa de mortalidade padronizada por idade estimada no Brasil foi 5,3/100 mil (II 95%, 4,5-5,8). A **Tabela 6-2** mostra as taxas de mortalidade e de DALYs padronizadas por idade (por 100 mil habitantes) por FA e *flutter* , em 2021, para ambos os sexos, no Brasil e suas UF. As UF com as mais altas taxas de mortalidade em 2021 foram Roraima, Amapá e Tocantins, enquanto as de mais baixas taxas foram Bahia, Maranhão e Rio Grande do Norte. Entretanto, devido a problemas de notificação, esses dados podem apresentar inconsistência e devem ser interpretados com cautela. Ainda não haviam sido publicadas outras estratificações dos dados do GBD 2021 para o Brasil até a data de finalização deste documento. Para dados do GBD 2019, consultar a Estatística Cardiovascular Brasil anterior.^4^

**Tabela 6-2:** – Taxas de DALYs e mortalidade padronizadas por idade (por 100 mil habitantes) por fibrilação e *flutter* atrial em 2021, no Brasil e suas unidades federativas

Local	DALYs	Mortalidade
Acre	126,2(106,6-152,7)	6,3(5,4-6,9)
Alagoas	122,2(100,3-147,1)	5,3(4,4-6)
Amapá	130,4(110,2-156,2)	6,7(5,6-7,5)
Amazonas	122,5(99,6-149,9)	5,6(4,8-6,1)
Bahia	116,9(93,8-145,1)	4,8(3,9-5,5)
Ceará	117,9(97-143,8)	5,3(4,5-6)
Distrito Federal	120,5(98,7-146,9)	6,5(5,6-7)
Espírito Santo	122,2(99,4-149,5)	5,5(4,5-6,1)
Goiás	118,6(96,7-145,4)	5,4(4,6-5,9)
Maranhão	109(85,9-131,6)	4,8(4-5,4)
Mato Grosso	122,7(101,7-150,3)	5,5(4,7-6)
Mato Grosso do Sul	121,1(99,5-146,1)	5,4(4,5-6)
Minas Gerais	119,1(98,7-146,2)	5,1(4,4-5,6)
Pará	118,5(96,2-146,4)	5,1(4-6)
Paraíba	119,6(97,7-148,1)	5,2(4,5-5,8)
Paraná	124,1(99,9-152,1)	5,6(4,8-6,2)
Pernambuco	113,5(93,8-138,8)	4,9(4,1-5,4)
Piauí	115,9(93,9-145,3)	5,1(4,2-5,8)
Rio de Janeiro	120,7(98,7-147,3)	5,3(4,5-5,9)
Rio Grande do Norte	114,2(91,4-141,2)	4,7(4-5,3)
Rio Grande do Sul	124,2(100,8-148,6)	5,6(4,8-6,2)
Rondônia	120,6(96,8-146,2)	5,4(4,6-6,2)
Roraima	135,6(112,1-162,4)	7,4(6,2-8,4)
Santa Catarina	123,3(101,6-152)	5,6(4,6-6,2)
São Paulo	125,2(100,6-152,9)	5,7(4,7-6,4)
Sergipe	122,8(100,9-150,6)	5,5(4,5-6,1)
Tocantins	123,1(102,3-148,3)	6(5-6,7)
Brasil	120,5(99,1-147,6)	5,3(4,5-5,8)

Fonte: Dados derivados do Global Burden of Disease Collaborative Network. Global Burden of Disease (GBD) Cardiovascular Burden Estimates 1990 and 2021, Institute for Health Metrics and Evaluation, University of Washington. ^48^

•A partir de dados de 1.558.421 ECG de pacientes da atenção primária do sistema de telessaúde de Minas Gerais, ligado ao Sistema de Informação sobre Mortalidade do Brasil, a taxa de mortalidade geral foi 3,34% em um seguimento médio de 3,68 anos. Após ajuste para idade e comorbidades, os portadores de FA apresentaram maior risco de morte geral (HR 2,10; IC 95%, 2,03-2,17) e cardiovascular (HR 2,06; IC 95%, 1,86-2,29), que foi ainda maior nas mulheres, que perderam sua vantagem de sobrevida em relação aos homens na presença de FA.^208 , 209^

•Em seguimento de 10 anos de 1.462 indivíduos com idade ≥60 anos (idade média, 69 anos; 61% mulheres) incluídos no estudo de coorte de Bambuí em 1997, FA ou *flutter* mostrou associação independente com um aumento na mortalidade por todas as causas (HR, 2,35; IC 95%, 1,53-3,62) entre pacientes com e sem DCh (HR,1,92; IC 95%, 1,05-3,51).^210^

•O registro RECALL foi um registro multicêntrico prospectivo que incluiu e acompanhou 4.585 pacientes com FA por 1 ano em 89 *sites* em todo o Brasil de abril de 2012 a agosto de 2019. Morte ocorreu em 8,8/100 pacientes/ano (IC 95%, 8,0-9,6) e, em modelos multivariados, foi associada com idade mais avançada, FA permanente, classe III/IV da NYHA, doença renal crônica, doença arterial periférica, AVC, doença pulmonar obstrutiva crônica e demência. O uso de anticoagulante foi associado a menor mortalidade. Pacientes com TTR <60% apresentaram maior mortalidade e mais eventos de sangramento maior em comparação aos pacientes com TTR ≥60%.^210^

## Carga de Doença

•De acordo com estimativas do GBD 2021, a taxa de DALYs por FA padronizada por idade no Brasil foi 120 (II 95%, 99-148) por 100 mil habitantes. Ainda não haviam sido publicadas outras estratificações dos dados do GBD 2021 para o Brasil até a data de finalização deste documento. Para dados do GBD 2019, consultar a Estatística Cardiovascular Brasil anterior.^209^

## Complicações

•A FA está relacionada a outros desfechos clínicos adversos além de morte, como AVC, hospitalizações, sangramento e demência.^211 - 215^

•Todos os 429 casos de AVC (87,2% dos quais isquêmicos) que ocorreram na cidade de Joinville em 2015 foram incluídos em um registro, sendo FA detectada em 11,4% deles e em 58% daqueles com AVC cardioembólico.^216^ Entre 2017 e 2020, dos 3.303 casos de AVC isquêmico na mesma cidade, 11% tinham FA. Desses pacientes com FA, 258 (71,6%) tinham diagnóstico prévio da doença e 102 (28,3%) foram diagnosticados após o AVC. Dos pacientes com FA diagnosticada previamente, 170 (47,2%) estavam em uso de anticoagulantes e 88 (24,4%) usavam outros medicamentos.^215^

•Em uma coorte de 1.121 pacientes com AVC isquêmico em seguimento de 12 anos, FA foi independentemente associada a aumento da mortalidade geral (HR 1,82; IC 95%, 1,43-2,31) e cardiovascular (HR 2,07; IC 95%, 1,36-3,14).^213^

•No registro prospectivo RECALL de pacientes com FA, a taxa de incidência de AVC foi 2,5/100 pacientes/ano (2,1-3,0), enquanto a taxa de incidência de embolia sistêmica foi 3,5/100 pacientes/ano (2,8-4,4). Sangramento maior ocorreu em 2,0/100 pacientes/ano (1,6-2,4) e hospitalização em 19,6/100 pacientes/ano (18,4-20,9). Hospitalização relacionou-se principalmente a causas cardiovasculares em comparação a sangramento (2,7/100 pacientes/ano, IC 95%, 2,2-3,2).^210^

•No ensaio clínico randomizado GIRAF, 301 pacientes idosos de seis centros em São Paulo foram randomizados para dabigatrana ou varfarina para avaliar se a incidência de demência diferia de acordo com o anticoagulante usado por 2 anos. Não houve diferença estatística com nível de significância de 5% em relação a qualquer desfecho cognitivo, após ajuste para múltiplas comparações, entre os grupos dabigatrana e varfarina.^214^

## Associação com Fatores de Risco para Fibrilação/Flutter Atrial

•Dados do sistema de telessaúde de Minas Gerais com ECG de 1.558.421 indivíduos (idade média, 51±18 anos; 40,2% homens) realizados entre 2010 e 2017 revelaram, em modelos multivariados ajustados por idade e sexo, a relação das seguintes comorbidades autorrelatadas com a presença de FA: DCh (OR 3,08; IC 95%, 2,91-3,25), infarto do miocárdio prévio (OR 1,74; 95% CI, 1,56-1,93), doença pulmonar obstrutiva crônica (OR 1,48; IC 95%, 1,33-1,66), hipertensão (OR 1,31; IC 95%, 1,27-1,34), dislipidemia (OR 1,09; IC 95%, 1,03-1,16). Tabagismo atual e diabetes não foram associados à prevalência de FA.^208^

•No registro prospectivo RECALL de pacientes com FA, os fatores de risco mais comuns foram hipertensão (77,9%), eventos cardiovasculares prévios (37,3%) e diabetes (30%).^210^

•Em uma análise transversal do estudo de coorte ELSA-Brasil, não foi identificada associação entre o escore de saúde cardiovascular proposto pela *American Heart Association* e o diagnóstico de FA na linha de base (80/13.141 ECG válidos, 0,8%).^216 , 217^

## Comorbidades Associadas

### Fibrilação atrial e outras doenças cardíacas

•A FA correlaciona-se a outras doenças cardiovasculares concomitantes. No ecocardiograma, FA foi associada com doença cardíaca (OR = 3,9; IC 95%, 2,1-7,2; p <0,001) em 1.518 pacientes (idade média, 58±16 anos; 66% mulheres) de uma lista de espera para ecocardiografia na atenção primária.^218^ Entre 300 idosos monitorados com marca-passo, a incidência de FA foi 22% em seguimento de 435 dias,^219^ tendo alcançado 85% dos pacientes com marca-passo e doença renal crônica em seguimento de 1 ano.^220^ Em outro estudo de 186 portadores de marca-passo de um único centro no sul do Brasil [52% mulheres; mediana de idade, 67 anos (IIQ 57-76)], a prevalência de FA foi 25,3%, com incidência de 5,64 casos/100 pessoas-ano.^221^ Entre os pacientes com doença cardiovascular que procuraram o setor de emergência, a prevalência de FA foi 40% daqueles com insuficiência cardíaca descompensada^222^ e 44% daqueles com doença valvar cardíaca.^187^

### Fibrilação atrial peroperatória e cirurgia cardiovascular

•No pós-operatório de cirurgia cardíaca, identificou-se FA em 12% a 33% dos pacientes.^223 - 226^ As cirurgias de substituição valvar foram associadas a maior ocorrência de FA (31%-33%) durante a hospitalização em comparação à cirurgia de revascularização miocárdica (12%-16%). Idade avançada, doença valvar mitral e não uso de betabloqueadores foram associados com FA no pós-operatório de cirurgia valvar.^227^ Entre aqueles submetidos a cirurgia de revascularização miocárdica, a incidência de FA no pós-operatório foi associada com átrio esquerdo >40,5 mm e idade >64,5 anos.^227^

•Em um estudo para avaliar o impacto de um programa de melhoria da qualidade na mortalidade hospitalar em um centro de cirurgia cardiovascular em São Paulo, com pacientes operados antes e depois da implementação do programa (858 em cada grupo), entre outros benefícios, houve redução na FA no pós-operatório de 4,4% para 1,5%, p<0,0001.^228^

### Fibrilação atrial e doença de Chagas

•A FA tem sido consistentemente associada a DCh e aumenta o risco de morte nos pacientes com DCh.^229^ Em uma revisão sistemática e meta-análise, a prevalência de FA foi significativamente maior em pacientes com DCh (OR: 2,11; IC 95%, 1,40-3,19).^231^

•Em uma grande amostra de 264.324 pacientes submetidos a tele-ECG em unidades de atenção primária à saúde, AF foi observada em 5,35% dos indivíduos com DCh e em 1,65% daqueles sem DCh (OR: 3,15; IC 95%, 2,83-3,51, com ajuste para idade, sexo e comorbidades autorrelatadas).^229^

•No estudo de coorte de Bambuí, 1.462 participantes com idade ≥60 anos (idade média, 69 anos; DCh n=557, 38,1%) e ECG na linha de base foram seguidos por 10 anos. Fibrilação atrial foi mais frequentemente observada nos indivíduos com DCh [6,1% vs. 3,4% (OR: 3,43; IC 95%, 1,87-6,32, com ajuste para idade, sexo e variáveis clínicas)], nos quais foi um fator de risco independente para morte (HR: 2,35; IC 95%, 1,53-3,62, com ajuste para idade, sexo, variáveis clínicas e níveis de BNP).^209^

### Fibrilação atrial e Pacientes Críticos

•Em um estudo observacional retrospectivo, com revisão de prontuários eletrônicos e inclusão de 895 pacientes com idade ≥80 anos, incluídos no protocolo de sepse de um hospital privado de alta complexidade na cidade de São Paulo, de janeiro de 2018 a dezembro de 2020, a incidência de FA na amostra foi 13%. A FA foi um fator de risco independente para mortalidade hospitalar.^233^

## Utilização e Custo da Atenção à Saúde

(Refer to Tabelas 1-1 até 1-7 e Figuras 1-11 até 1-1)

•De 2008 a 2021, houve 406.666 hospitalizações por FA, variando de 25.283 em 2021 a 32.753 em 2019, logo antes da pandemia de COVID-19, revelando uma redução nas hospitalizações por FA na pandemia ( Figura 6-1 ). De 2008 a 2021, os custos totais devidos às hospitalizações por FA foram R$ 310.739.362. Após ajuste para a inflação brasileira, os custos foram R$ 569.678.472 e, em dólares internacionais convertidos em US$ 2023 ajustados para PPC, $ 108.530.857. Interessante notar que, a despeito da redução no número anual de hospitalizações para FA em 2020 e 2021 no contexto de pandemia de COVID-19, os custos anuais ajustados para a inflação brasileira não diminuíram na mesma proporção.

**Figura 6-1:**
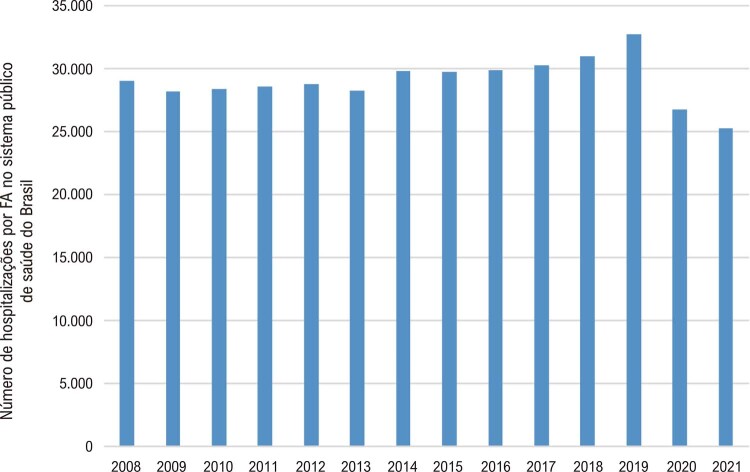
– Número de hospitalizações por fibrilação atrial no sistema público de saúde do Brasil, de 2008 a 2021. Dados do Ministério da Saúde do Brasil – Sistema de Informações Hospitalares do Sistema Único de Saúde (SIH/SUS). ^49^

•Com relação a ablação de FA e *flutter* atrial, 1.694 procedimentos foram realizados pelo SUS de 2008 a 2021, com uma redução em 2020 em comparação a 2019 (120 vs 163, respectivamente) e recuperação em 2021 (161 procedimentos). O custo não ajustado da ablação de FA de 2008 a 2021 foi R$ 9.614.010. Após ajuste para a inflação brasileira e conversão para US$ 2023 ajustado para PPC, foi $ 2.751.563.

### Conhecimento, Tratamento e Controle

•Um estudo com métodos mistos foi realizado para identificar as percepções sobre o cuidado de pacientes com FA, assim como barreiras e facilitadores para tal cuidado, em 11 unidades de atenção primária em São Paulo, a partir da perspectiva de 107 profissionais da saúde. O estudou mostrou que a falta de treinamento específico sobre FA para profissionais da saúde, de protocolos/diretrizes sobre condutas em FA e de programas de educação para o paciente, assim como o reduzido acesso a testes de INR em unidades de atenção primária e a limitada disponibilidade dos novos anticoagulantes orais são as principais barreiras para um cuidado ótimo.^234^

•Anticoagulação

•O uso de anticoagulação entre pacientes com FA variou muito, de 1,5% a 91%.^204 , 205 , 235 , 236^ Estudos com amostras da atenção primária apresentaram maior probabilidade de baixo uso de anticoagulação em comparação a amostras recrutadas de centros terciários ou de cardiologistas, como detalhado a seguir.

•Em centros de atenção primária de 658 municípios de Minas Gerais, o uso médio de anticoagulante relatado foi de 1,5%, enquanto, no estudo IMPACT-AF, 91% dos pacientes arrolados no Brasil (n=360), a maioria em acompanhamento com cardiologista em centros terciários, estavam em uso de anticoagulação oral na linha de base.^204 , 235^

•No estudo de coorte de base populacional ELSA-Brasil, de 185 participantes com FA e escore CHA_2_DS_2_-VASc ≥2, apenas 20 (10,8%) usavam anticoagulantes. A prevenção de AVC no grupo foi associada com idade mais avançada (1,8% *vs.* 17,7% naqueles ≤54 e ≥65 anos, respectivamente; p=0,013). Observou-se tendência a menor uso de anticoagulantes em mulheres (7,1% *vs.* 16,4% em mulheres e homens, respectivamente; p=0,055).^202^

•No registro prospectivo RECALL de pacientes com FA, o escore CHA_2_DS_2_-VASc médio (desvio-padrão) foi 3,2 (1,6); a mediana do escore HAS-BLED foi 2 (2, 3). Na linha de base, 78% dos pacientes estavam em uso de anticoagulantes (62,6% usavam antagonistas da vitamina K e 37,4% usavam DOAC). As principais razões do não uso dos anticoagulantes orais foram decisão do médico (24,6%) e dificuldade para controlar (14,7%) ou realizar (9,9%) INR ( **Figura 6-2** ).^210^

**Figura 6-2:**
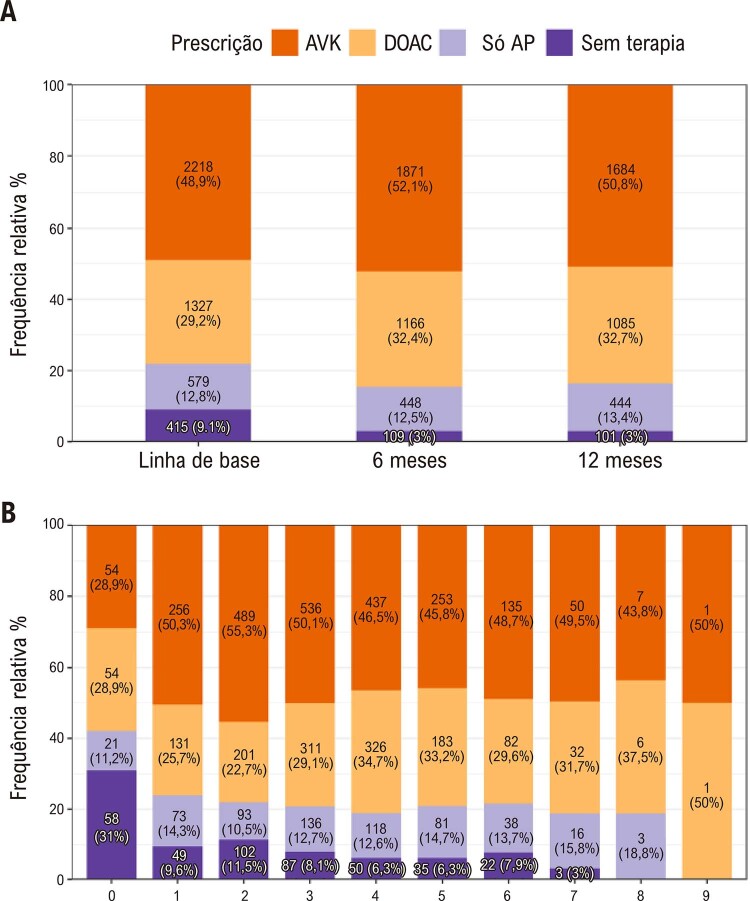
– Terapias antitrombóticas durante 1 ano de seguimento (A) e na linha de base (B) de acordo com o CHA *2* DS *2* -VASc. AVK: antagonista da vitamina K; DOAC: anticoagulante oral direto; AP: antiagregante plaquetário. Fonte: Lopes et al. ^210^

•Uso de anticoagulação foi associado com menor risco de morte no registro prospectivo RECALL de pacientes com FA e em uma coorte de 200 pacientes com AVC isquêmico e FA (efeito tempo-dependente do anticoagulante oral: HR multivariável, 0,47; IC 95%, 0,30-0,50) e mortalidade por AVC (efeito tempo-dependente do anticoagulante oral ≥ 6 meses: OR multivariável, 0,09; IC 95%, 0,01-0,65).^210 , 236^

•A qualidade da terapia com varfarina foi avaliada usando TTR como parâmetro em diferentes amostras no Brasil. O TTR da anticoagulação para FA variou de 31% a 67% nos estudos.^210 , 233 , 235 - 238^ No registro prospectivo RECALL de pacientes com FA, o TTR médio (desvio-padrão) para o período do estudo foi 49,5% (27,5). Durante o seguimento, o uso de anticoagulantes e o INR na faixa terapêutica aumentaram para 87,1% e 59,1%, respectivamente.^210^

•Em um estudo para avaliar os custos relacionados à terapia anticoagulante de pacientes com FA (n=90) tratados em hospital universitário público em 1 ano, os autores relataram custos totais mais altos naqueles usando DOAC em comparação àqueles usando varfarina. Entretanto, uma redução de 40% no preço de DOAC facilitaria a incorporação desses medicamentos no sistema brasileiro de saúde pública.^237^

### Controle de ritmo ou frequência (medicamento, cardioversão, ablação por cateter)

•Um estudo transversal com 167 pacientes com FA relatou que controle da frequência foi mais comum do que controle do ritmo como estratégia de tratamento (79% vs. 21%; p < 0,001).^239^

•Dados de 125 centros de atenção primária mostraram que, de 301 pacientes com FA, 91 (30,2%) não recebiam qualquer tipo de tratamento para controle de frequência ou ritmo. Dos demais pacientes com FA, 70% usavam apenas agentes para controle de frequência e 30% usavam pelo menos um agente antiarrítmico.^205^

•No registro prospectivo RECALL de pacientes com FA, 4,4% tinham história prévia de ablação de FA, 0,9% de ablação do nó atrioventricular com implantação de marca-passo e 25,2% de cardioversão. Quase 30% usaram um antiarrítmico para controle de ritmo, principalmente amiodarona. Para controle da frequência cardíaca, 69,5% usaram betabloqueadores, 25,7% receberam bloqueadores de canal de cálcio não diidropiridínicos, e 15,8% usavam digitálicos.^210^

## Impacto da Pandemia de COVID-19

•Em pacientes com COVID-19, FA está associada com maior risco para eventos adversos,^239^ embora um estudo retrospectivo unicêntrico de pacientes hospitalizados por COVID-19 no Brasil (n=128) não tenha observado associação de FA e mortalidade.^240^

•Houve redução em hospitalizações e procedimentos para FA na pandemia, em particular em 2020 ( **Figura 6-1** ). Essa redução relacionou-se provavelmente aos efeitos indiretos da pandemia, quando houve uma diminuição nas hospitalizações cardiovasculares em geral no Brasil e no mundo, pois procedimentos eletivos foram adiados, contato com provedores de serviços de saúde foi evitado e cuidado cardiovascular foi interrompido.^14 , 241^

## Perspectivas

•Estudos de coorte de base populacional sobre a incidência e determinantes de FA no Brasil estão em andamento.

•A triagem de FA em estudos de base populacional ou populações selecionadas através do uso de ECG ou dispositivos, incluindo dispositivos eletrônicos vestíveis e *patches* de longa duração, deve contribuir com informação sobre a relevância da inclusão dessa estratégia nos centros de atenção primária ou especializados.

•Estudos utilizando algoritmos de inteligência artificial para diagnosticar ou prever FA a partir de ECG foram desenvolvidos, inclusive a partir de dados brasileiros.^242^ Esses algoritmos de inteligência artificial podem ser uma ferramenta para melhorar o diagnóstico de FA e personalizar as estratégias de triagem conforme o risco.

•Estratégias para reduzir as lacunas no tratamento da FA devem ser desenvolvidas, incluindo a avaliação de custo-efetividade da incorporação de DOAC no sistema de saúde pública.

•A implementação de estratégias para melhorar o uso de anticoagulação em pacientes com FA deve ser encorajada, particularmente na atenção primária.

## CAPÍTULO 7 – HIPERTENSÃO

### CID-10 - I10

#### Ver Tabelas 7-1 a 7-5 e Figuras 7-1 a 7-3

**Table:** Abreviaturas Usadas no Capítulo 7

aPR	Razão de Prevalência Ajustada
CID-10	Classificação Estatística Internacional de Doenças e Problemas Relacionados à Saúde, 10^a^ Revisão
DALYs	Anos de vida perdidos ajustados por incapacidade (do inglês, * Disability-Adjusted Life-Year* )
ELSA-Brasil	Estudo Longitudinal de Saúde do Adulto
GBD	Global Burden of Disease
IC	Intervalo de Confiança
IMC	Índice de Massa Corporal
OR	Odds Ratio
PAD	Pressão Arterial Diastólica
PAS	Pressão Arterial Sistólica
SUS	Sistema Único de Saúde
UF	Unidade Federativa
VIGITEL	Sistema de Vigilância de Fatores de Risco e Proteção para Doenças Crônicas por Inquérito Telefônico

## Panorama

•Com o objetivo de padronização neste documento, hipertensão arterial foi caracterizada como níveis sustentados de PAS igual ou superior a 140 mmHg e/ou PAD igual ou superior a 90 mmHg.^243^

•Os valores de prevalência percentual serão apresentados seguidos por IC 95%, como disponibilizado nos estudos citados. Em estudos populacionais, hipertensão pode ser aferida ou autorreferida. No primeiro caso, o valor deriva da medida direta da pressão arterial através da utilização de técnicas padronizadas e é descrito em um documento, enquanto a hipertensão autorreferida se caracteriza por uma resposta positiva à pergunta sobre a presença desse diagnóstico médico ou o uso de anti-hipertensivos,^244^ dependendo, portanto, do acesso do paciente ao diagnóstico e do seu entendimento sobre essa informação, o que resulta em diferenças na prevalência de hipertensão conforme a coleta de dados.

•Quando o Estudo GBD é a fonte de dados, o risco é atribuído à PAS alta, como descrito em publicação anterior.^245^

## Incidência e Prevalência de Hipertensão

### Crianças e Adolescentes

•Uma revisão sistemática e meta-análise de Paiva *et al* ., usando 15 estudos e incluindo 43.227 adolescentes, investigou a prevalência de síndrome metabólica e seus componentes em adolescentes brasileiros. Os autores estimaram a prevalência de hipertensão em 10,3% (7,8-13,5) dos adolescentes brasileiros. Hipertensão foi o terceiro componente mais prevalente da síndrome metabólica nessa população, após níveis baixos de colesterol da lipoproteína de alta densidade [22,1% (12,5-36,2)] e obesidade abdominal [11,0% (8,1-14,9)].^246^

•Em estudo longitudinal avaliando 469 crianças e adolescentes do sul do Brasil, com idade de 7-17 anos (43,1% meninos), PAS e PAD, circunferência da cintura, IMC, percentual de gordura corporal, perfil lipídico, glicose, aptidão cardiorrespiratória e polimorfismo rs9939609 foram avaliados. A incidência acumulada de hipertensão foi calculada e realizou-se regressão logística multinomial. A medida da pressão arterial foi classificada de acordo com os percentis 90 e 95 para hipertensão limítrofe e hipertensão, respectivamente.^247^ A incidência de hipertensão foi 11,5% após três anos de seguimento. Indivíduos com sobrepeso ou obesos apresentaram maior probabilidade de se tornarem hipertensos limítrofes (sobrepeso: OR 3,22, IC 95%, 1,08-9,55; obesidade: OR 4,05, IC 95%, 1,68-9,75) e indivíduos obesos apresentaram maior probabilidade de se tornarem hipertensos (obesidade: OR 4,84, IC 95%, 1,57-14,95). Valores de circunferência da cintura e percentual de gordura corporal de alto risco foram associados ao desenvolvimento de hipertensão (OR 3,41, IC 95%, 1,26-9,19 e OR 2,49, IC 95%, 1,08-5,75, respectivamente).^248^

### Adultos

•Malta *et al* .,^248^ em estudo que analisou os dados da Pesquisa Nacional de Saúde 2019,^249^ encontraram prevalência de hipertensão autorreferida no Brasil de 23,93% (IC 95%, 23,42- 24,43), com 26,45% (IC 95%, 25,75-27,15) para mulheres e 21,06% (20,37-21,75) para homens ( **Tabelas 7-1** e **7-2** ). Quanto aos grupos etários, a mais alta prevalência foi observada em indivíduos com mais de 60 anos de idade e naqueles com baixo nível educacional, em ambos os sexos.^248^ Vale ressaltar que a prevalência de hipertensão em mulheres tende a ser mais alta do que em homens quando avaliada a partir de dados autorreferidos, fenômeno não observado nos estudos que utilizam a medida da pressão arterial.

•Em outro estudo, Macinko *et al* . avaliaram doenças crônicas não transmissíveis e sua relação com desigualdade de nível educacional, entre 2013 e 2019.^250^ Esse estudo usou dados da Pesquisa Nacional de Saúde de 2013^251^ e da de 2019^249^ no Brasil. Os dados não ajustados sobre prevalência de hipertensão em adultos com mais de 18 anos mostraram aumento, passando de 22% (IC 95%, 21,4-22,7) em 2013 para 25,9% (IC 95%, 25,4-26,4) em 2019 em ambos os sexos.^250^

•De acordo com dados do VIGITEL para 2021, a porcentagem de pacientes com idade a partir de 18 anos com diagnóstico autorreferido de hipertensão no Brasil foi 26,3% (IC 95%, 25,1-27,6), sendo 27,1% (IC 95%, 25,5-28,7) em mulheres e 25,4% (IC 95%, 23,4-27,4) em homens.^252^ Em relação às capitais, essa porcentagem variou de 19,3% (IC 95%, 15,7-23,0) na cidade de São Luís a 32% (IC 95%, 27,5-36,4) na cidade do Rio de Janeiro em ambos os sexos.^252^ Entre as mulheres, a mais alta porcentagem observada foi 32,2% (IC 95%, 27,5-36,9) na cidade de Belo Horizonte e a mais baixa, 19,3% (IC 95%, 16,0-22,6) na cidade de Macapá. Por outro lado, entre os homens, a mais alta porcentagem foi 32,2% (IC 95%, 24,7-39,7) na cidade do Rio de Janeiro e a mais baixa, 13,8% (IC 95%, 8,1-19,6) na cidade de São Luís.^252^ Quanto aos grupos etários, a mais alta prevalência de hipertensão no país, 61% (IC 95%, 59,0-63,0), foi observada em indivíduos com idade a partir de 65 anos, sendo que, nesse grupo etário, as mulheres apresentaram maior prevalência do que os homens, 63,7% (IC 95%, 61,6-65,8) e 57,1% (IC 95%, 53,4-60,7), respectivamente.^252^ A **Figura 7-1** mostra a evolução temporal das porcentagens de hipertensão no país entre 2007 e 2021 em ambos os sexos. A **Figura 7-2** mostra a prevalência de hipertensão no país por UF de acordo com os quartis em 2021, com base no estudo VIGITEL 2021.^252^ Novamente, usando dados autorreferidos, maior prevalência foi observada nas mulheres em comparação aos homens, diferentemente de quando dados de medidas da pressão arterial são usados.

**Figura 7-1:**
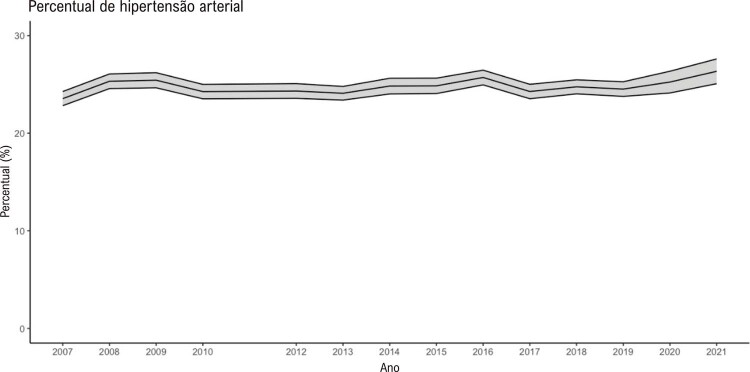
– Tendências da prevalência de hipertensão nas capitais brasileiras entre 2007 e 2021, para ambos os sexos. Dados do VIGITEL Brasil 2021. ^252^

**Figura 7-2:**
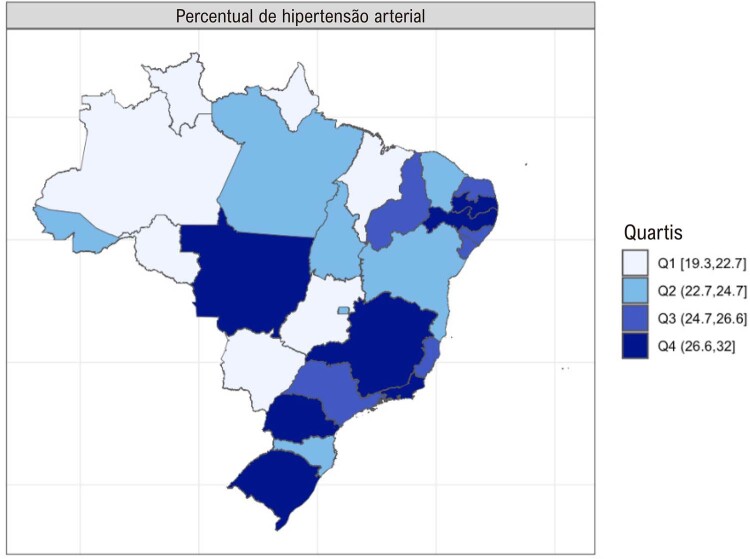
– Prevalência de hipertensão no Brasil por unidade federativa e de acordo com os quartis de porcentagem, em 2021, com base no estudo Vigitel 2021. Dados do Vigitel Brasil 2021. ^252^

•No estudo de coorte ELSA-Brasil, Scaranni *et al* . avaliaram a relação entre o consumo de alimentos ultraprocessados e a incidência de hipertensão em pacientes previamente normotensos. Foi incluído um total de 8.754 participantes com idade de 35-74 anos, tendo sido 1.312 casos de hipertensão observados ao final de seguimento de 4 anos.^253^ Além disso, os autores relataram que os indivíduos com alto consumo de alimentos ultraprocessados apresentaram maior risco de desenvolver hipertensão (OR 1,23, IC 95%, 1,06-1,44).^253^

•Ainda no estudo de coorte ELSA-Brasil, ao se avaliar a incidência de hipertensão de acordo com sexo e raça, observou-se mais alta incidência de hipertensão em homens negros (59,4/1000 pessoas-ano), enquanto a mais baixa incidência foi observada em mulheres brancas (30,5/1000 pessoas-ano).^254^ Após ajuste para idade e história familiar de hipertensão, a razão da taxa de incidência foi mais alta em homens negros (2,25; IC 95%, 1,65–3,08), homens pardos, mulheres negras, mulheres pardas e homens brancos em comparação a mulheres brancas.^254^

•O consumo de álcool foi associado a maior incidência de hipertensão apenas na população masculina no Brasil. De acordo com Coelho *et al* .,^255^ em análise do estudo de coorte ELSA-Brasil envolvendo 3.990 indivíduos com idade de 35-74 anos, os homens nos mais altos tercis de consumo total de álcool, de cerveja, de vinho e de bebidas alcoólicas apresentaram a mais alta OR para hipertensão em comparação àqueles nos tercis mais baixos (1,62 [IC 95%, 1,14-2,29], 1,51 [IC 95%, 1,07-12,13], 1,71 [IC 95%, 1,01-2,86] e 2,01 [IC 95%, 1,21-3,32], respectivamente).

## Mortalidade Atribuível a Hipertensão

•As estimativas do GBD para as taxas de mortalidade e DALYs e para o número absoluto de mortes atribuíveis a PAS elevada no Brasil e em suas 27 UF de 1990 a 2019 são apresentadas na versão anterior deste documento, pois as estimativas do GBD ainda não haviam sido atualizadas até o momento de finalização deste documento.^4^

## Morbidade e Carga de Doença Atribuíveis a Hipertensão

•As tendências de carga de doença atribuível à exposição a fatores de risco foram analisadas de acordo com sexo e grupos etários, usando as estimativas do GBD de 1990 a 2019. Para mortalidade, hipertensão foi o principal fator de risco responsável pela maioria das mortes (104,8 por 100 mil mortes). Para DALYs, no mesmo período, IMC alto, PAS elevada e glicemia de jejum alta ocuparam as três primeiras posições no *ranking* . Para DALYs, IMC alto foi o principal fator de risco para mulheres, enquanto consumo de álcool foi para os homens.^256^

•Pires *et al.* estudaram a prevalência do *cluster* hipertensão, obesidade e/ou diabetes e investigaram sua associação com fatores sociodemográficos e comportamentais. Os autores verificaram que a prevalência de hipertensão foi mais alta nos homens do que nas mulheres [17,8% (IC 95%, 17,0-18,6) vs. 11,4% (IC 95%, 10,8-12,0)]. A prevalência de multimorbidade foi 9,6%. As probabilidades de multimorbidade foram mais altas na idade mais avançada, entre os negros, mas mais baixas na região norte do país para os dois sexos. As probabilidades de multimorbidade das doenças não transmissíveis aumentaram entre as mulheres com níveis educacionais mais baixos e estilo de vida sedentário e entre os homens que viviam acompanhados e eram fisicamente inativos. Em comparação aos homens, as mulheres apresentaram mais alta prevalência de obesidade [15,9% (IC 95%, 15,2-16,6) vs. 9,4% (IC 95%, 8,8-10,0)], obesidade e diabetes mellitus [1,6% (IC 95%, 1,4-1,8) vs. 0,9% (IC 95%, 0,9-1,2)] e três doenças não transmissíveis [1,3% (IC 95%, 1,1-1,6) vs. 0,8% (IC 95%, 0,6-1,0)].^257^

•Entre os anos 1990 e 2019, as estimativas de mortes por doença renal crônica secundária a hipertensão aumentaram de 2,4 por 100 mil habitantes em 1990 para 5,38 por 100 mil habitantes em 2019, tendo a região sudeste as maiores taxas de mortalidade. Quanto ao sexo, as mais altas taxas foram observadas entre os homens; entretanto, com o passar dos anos, essa diferença diminuiu. O grupo etário ≥70 anos foi o mais afetado, apresentando as mais altas taxas de morte.^258 - 262^

## Impacto na Saúde Cardiovascular

•Malta *et al* ., usando dados da Pesquisa Nacional de Saúde de 2013 e de 2019, avaliaram 60.202 indivíduos e demonstraram que os indivíduos negros apresentaram mais hospitalizações por hipertensão ou suas complicações (PR=1,2; IC 95%, 1,05-1,38) além de limitação grave ou muito grave para as atividades da vida diária (RP=1,37; IC 95%, 1,06-1,76).^263^ Relataram ainda resultados conflitantes quanto à comparação da qualidade da assistência a pacientes hipertensos. A despeito do aumento no uso de medicação do programa federal, observou-se redução no acesso a especialistas, quando necessário.^261^

•Em 4.717 participantes do estudo de coorte ELSA-Brasil, sem diabetes e sem doença cardiovascular na linha de base (2008-2010), resistência a insulina avaliada pelo HOMA-IR foi associada à chance de desenvolver pré-hipertensão de 51% (IC 95%, 1,28-1,79) e hipertensão de 150% (IC 95%, 1,48-4,23). Mesmo em indivíduos não obesos (IMC<25 kg/m^2^), a resistência a insulina foi associada a incidência de pré-hipertensão (OR 1,41; IC 95%, 1,01-1,98) e hipertensão (OR 3,15; IC 95%, 1,27-7,81).^260^

•O papel da urbanização na saúde cardiometabólica foi elegantemente investigado por Kramer *et al* . Esses autores realizaram revisão sistemática e meta-análise de 46 estudos, incluindo um total de 20.574 adultos de pelo menos 33 etnias indígenas brasileiras, para entender a saúde metabólica e o papel da urbanização e do desmatamento (ambiental) no risco cardiovascular. Avaliaram a prevalência de obesidade e fatores de risco cardiometabólicos relacionados. Meta-análises sobre a prevalência de obesidade mostraram taxas de obesidade mais altas entre os indígenas vivendo nas regiões Centro-Oeste (23% [IC 95%, 17–29]) e Sul do Brasil (23% [13–34]) do que aqueles vivendo nas regiões menos urbanizadas (Norte do Brasil: 11% [8-15]). O mesmo padrão foi observado para hipertensão: a prevalência foi mais alta no Sul (30% [10–50]) e mais baixa na região Norte, a menos urbanizada (1% [1-2]). A prevalência de obesidade foi 3,5 vezes mais alta em indivíduos vivendo nos territórios indígenas urbanizados (28%) do que naqueles vivendo na floresta Amazônica nativa (8%). Os autores não observaram variação incremental na pressão arterial com o envelhecimento dos indígenas que viviam conforme seu estilo de vida tradicional, ao contrário daqueles vivendo nas regiões urbanizadas. Para os anos 1997 e 2019, a taxa de mortalidade cardiovascular entre residentes das áreas mais urbanizadas foi 2,5 vezes maior do que a observada no Norte. Entretanto, o aumento incremental na mortalidade cardiovascular nas duas últimas décadas entre os indígenas brasileiros foi observado apenas nas populações do Norte e Nordeste (aumento de 2,7 vezes).^260^

## Conhecimento, Tratamento e Controle da Hipertensão

•Tavares *et al* . investigaram o controle da saúde cardiovascular de acordo com o construto da *American Heart Association* de sete métricas cardiovasculares em 400 pacientes adultos seguidos na Estratégia de Saúde da Família em Sergipe.^262^ Apenas 32,5% tinham a saúde cardiovascular controlada (≥5 métricas idealmente controladas) e, quanto a hipertensão, apenas 35% da população estava idealmente controlada (pressão arterial < 120/80 mm Hg).^250^As características ‘ser mulher’, ‘jovem’ e ‘seguir a orientação de familiares e vizinhos’ influenciaram positivamente no controle da saúde cardiovascular.^262^

•A Pesquisa Nacional de Saúde estimou que de 88.531 indivíduos, 23,9% apresentavam hipertensão autorreferida. Desses, 57,8% relataram cuidados médicos nos seis meses anteriores: 61,1% no serviço público de saúde e 45,8% em unidades de atenção primária^262^ ( **Tabelas 7-3, 7-4, 7-5** e **Figura 7-3** ).

**Figura 7-3:**
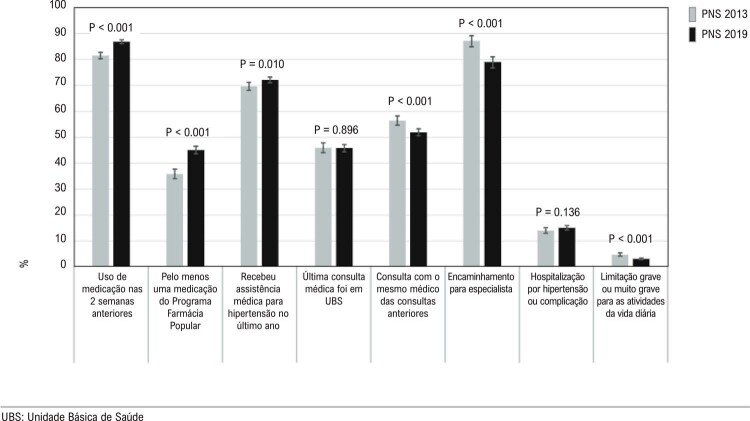
– Comparação dos indicadores de assistência de saúde de adultos com hipertensão arterial. Pesquisa Nacional de Saúde, Brasil, 2013 e 2019. Fonte: Malta et al. ^261^

•A proporção de portadores de diabetes e hipertensão obtendo medicação no Programa Brasileiro de Farmácia Popular foi estimada usando dados da Pesquisa Nacional de Saúde 2019: 45,1% (IC 95%, 43,7-46,5%) para hipertensão e 51,5% (IC 95%, 49,5-53,6%) para diabetes. A obtenção de medicação foi mais alta na região Sul e mais baixa nos indivíduos com níveis mais altos de educação e renda^264^( **Tabelas 7-3, 7-4, 7-5** e **Figura 7-3** ).

•Usando dados do estudo de coorte ELSA-Brasil, 3.897 participantes com hipertensão foram avaliados quanto a controle da pressão arterial, raça autorreferida e segregação econômica residencial em um estudo transversal. Após ajuste para idade, sexo, nível educacional e centro do estudo, a porção não explicada (disparidade residual) da raça na hipertensão não controlada foi 18,2% (IC 95%, 13,4%-22,9%) para participantes negros vs. brancos e 12,6% (8,2%-17,1%) para participantes pardos vs. brancos. Entretanto, a porção explicada (redução de disparidade) através de segregação econômica foi -2,1% (-5,1%; 1,3%) para participantes negros vs. brancos e 0,5% (-1,7%; 2,8%) para participantes pardos vs. brancos. Embora a hipertensão não controlada tenha sido maior para negros e pardos vs. brancos, as desigualdades raciais na hipertensão não controlada não foram explicadas por segregação econômica.^265^

## COVID-19 e Hipertensão

•Durante a pandemia, houve discreto aumento na prevalência de hipertensão nas capitais brasileiras de acordo com dados do VIGITEL em comparação à tendência de estabilidade de 2009 a 2019 ( **Figura 7-1** ).

•Malta *et al* . investigaram a associação entre diagnóstico autorreferido de doenças não transmissíveis, incluindo hipertensão, e a adesão ao distanciamento social e o uso dos serviços de saúde durante a pandemia de COVID-19 através de enquete telefônica em 2020. Os indivíduos com doenças não transmissíveis mostraram maior adesão ao distanciamento social intenso (aPR: 1,07; IC 95%, 1,03-1,11), buscaram os serviços de saúde com maior frequência (aPR: 1,24; IC 95%, 1,11-1,38) e tiveram maior dificuldade para agendar consultas médicas (aPR: 1,52; IC 95%, 1,35-1,71), para receber tratamento (aPR: 1,50; IC 95%, 1,22-1,84) e medicação (APR:2,17; IC 95%, 1,77-2,67) e para se submeter a exames (aPR: 1,78; IC 95%, 1,50-2,10) e a intervenções eletivas (aPR: 1,65; IC 95%, 1,16-2,34).^266^

•O controle da hipertensão durante a pandemia de COVID-19 no Brasil foi avaliado por Feitosa *et al* . Os autores relataram uma discreta redução, com questionável significado clínico, no monitoramento da pressão arterial no consultório e domiciliar nos primeiros meses seguintes ao surto de COVID-19 em 987 pacientes hipertensos tratados em comparação a 27.699 pacientes avaliados antes do surto de COVID-19. Entre os não tratados, não se observou diferença no controle da hipertensão.^267^ Duarte *et al* . investigaram 194 mortes relacionadas à COVID-19 no sul do Brasil. A maioria da população era do sexo masculino (63,4%), ≥60 anos de idade (82,5%) e branca (82,5%). Cerca de metade apresentava multimorbidade. As prevalências de hipertensão, diabetes e doença cardíaca foram 29,4% (23,3-36,2), 28,0% (22,1- 34,8) e 38,7 % (32,0-45,7), respectivamente.^268^

•Em estudo retrospectivo de 1.276 mortes por COVID-19 em Pernambuco, Santos *et al* . relataram que 26.48% dos pacientes apresentavam hipertensão como comorbidade prévia.^269^

## Perspectiva

•Quanto à diminuição da carga cardiovascular da hipertensão no Brasil, há uma lacuna referente ao conhecimento mais profundo e holístico acerca de como melhorar a prevenção, a conscientização, o tratamento e o controle da hipertensão, assim como referente à sua relação com outros comportamentos e fatores de risco cardiovasculares desfavoráveis, como proposto pela *American Heart Association* .^216^ O atual entendimento indica ser a hipertensão, em nível populacional, o principal determinante de morbimortalidade cardiovascular em âmbito nacional. Portanto, há necessidade urgente no Brasil de melhores dados para a medição de desfechos populacionais e do desempenho do sistema de saúde para embasar a implementação de estratégias científicas investigativas de como melhorar esses desfechos.^110 , 270 , 271^

•Além disso, temos que avançar de um modelo de política de saúde em que apenas se gera evidência para um modelo de ação a fim de corrigir a realidade subótima revelada pela ciência. Estratégias nacionais populacionais com campanhas efetivas para a promoção de hábitos saudáveis ( *i.e.* , redução do sal na dieta, taxação dos alimentos pouco saudáveis, aumento de atividade física) aliadas a identificação e tratamento mais efetivos dos indivíduos com maior risco cardiovascular, assim como a vigilância objetiva dos resultados, devem ser prioridade máxima no futuro próximo.^110 , 270 , 271^

•Aperfeiçoamento da pesquisa relacionada às disparidades de acesso, tempos e desfechos para os hipertensos usuários do SUS em comparação àqueles dos serviços privados de saúde, como *benchmarking* , poderia auxiliar na elaboração de novas políticas públicas de saúde para reduzir a carga de hipertensão na nossa sociedade.

•Considerando-se que aproximadamente 75% dos brasileiros são usuários do SUS, é imperativo que se meçam continuamente os desfechos dos programas de hipertensão implementados pelo SUS, como a Estratégia de Saúde da Família, e que se comparem àqueles obtidos no sistema privado de saúde.^261^

## CAPÍTULO 8 – DISLIPIDEMIA

### CID-10 E78 (E78.0 – E78.9); CID-10-CM E78 (E78.0 – E78.9)

#### Ver Tabelas 8-1 a 8-4

**Table:** Abreviaturas usadas no Capítulo 8

CT	Colesterol Total
DALYs	Anos de vida perdidos ajustados por incapacidade (do inglês, DisabilityAdjusted Life-Year)
DCV	Doenças Cardiovasculares
ELSA-Brasil	Estudo Longitudinal de Saúde do Adulto - Brasil
ERICA	Estudo de Riscos Cardiovasculares em Adolescentes
GBD	Carga Global de Doenças (do inglês, Global Burden of Disease)
HDLc	Colesterol da Lipoproteína de Alta Densidade (do inglês, High-Density Lipoprotein Cholesterol)
IAM	Infarto Agudo do Miocárdio
IC	Intervalo de Confiança
II	Intervalo de Incerteza
LDLc	Colesterol da Lipoproteína de Baixa Densidade (do inglês, Low-Density Lipoprotein Cholesterol)
OR	Odds Ratio
PNAUM	Pesquisa Nacional sobre Acesso, Utilização e Promoção do Uso Racional de Medicamentos no Brasil
PNS	Pesquisa Nacional de Saúde
RP	Razão de Prevalência
SDI	Índice Sociodemográfico (do inglês, Sociodemographic Index)
SUS	Sistema Único de Saúde
TG	Triglicerídeos
UF	Unidade Federativa
YLDs	Anos vividos com incapacidade (do inglês, Years Lived with Disability)
YLLs	Anos potenciais de vida perdidos (do inglês, Years of Life Lost)

## Introdução

•Define-se dislipidemia como níveis séricos anormais de lipídios, incluindo colesterol, suas frações e/ou TG. A dislipidemia é um conhecido fator de risco para as DCV.^273^ Mesmo no âmbito da prevenção primária, estudos randomizados já demonstraram que o tratamento da dislipidemia efetivamente reduz as DCV.^274^ No presente documento os dados laboratoriais da PNS coletados em 2014-2015 foram usadas como fonte principal de dados sobre os níveis médios de colesterol e a prevalência de dislipidemia em adultos, enquanto o Estudo ERICA foi utilizado para dados sobre adolescentes. Estudos menores (de base regional) foram utilizados, quando apropriado para descrições adicionais. Este documento traz referências da edição anterior de 2021 do Estatística Cardiovascular^4^ salvo, quando da disponibilidade de dados mais recentes.

•Este capítulo apresenta dados sobre CT, LDLc, HDLc e TG. As definições de dislipidemia variam historicamente e conforme as posições das sociedades de cardiologia locais. Com o objetivo de classificação e a menos que especificado de outra maneira, neste capítulo, usaremos o termo dislipidemia para os seguintes valores: 1) em adultos: CT ≥ 200 mg/dl, LDLc ≥ 130 mg/dl, HDLc < 40 mg/dl, ou TG ≥ 150 mg/dl;^275^ 2) em crianças e adolescentes: CT ≥ 170 mg/dl, LDLc ≥ 130 mg/dl, HDLc < 45 mg/dl, e TG ≥ 130 mg/dl.^276^

## Prevalência

### Jovens

•O Estudo ERICA, publicado em 2021, reporta dados de 38.069 estudantes (60% sexo feminino) com idade variando de 12 a 17 anos, nas capitais das 27 UF brasileiras, além de cinco conjuntos de municípios com mais de 100 mil habitantes, nas cinco regiões geográficas do país.^276^ Os seguintes valores médios foram encontrados: CT, 148 mg/dl (IC 95%, 147-149 mg/dl); LDLc, 85 mg/dl (IC 95%, 84-86 mg/dl); HDLc, 47 mg/dl (IC 95%, 47-48 mg/dl); e TG, 78 mg/dl (IC 95%, 76-79 mg/dl). Com relação à prevalência de valores anormais, 20,1% (IC 95%, 19-21,3%) mostraram aumento de CT, 3,5% (IC 95%, 3,2-4%) de LDLc e 7,8% (IC 95%, 7,1-8,6%) de TG. A prevalência de HDLc baixo foi de 47% (IC 95%, 45-49%). Os dados estratificados por idade e sexo são apresentados na **Tabela 8-1** .

**Tabela 8-1:** – Níveis médios de lipídios plasmáticos, prevalências de níveis borderline e altos, além de população estimada com níveis lipídicos anormais, de acordo com sexo e grupo etário. ERICA-Brasil, 2013-2014

Lipídios	Médio		Borderline		Alto		População estimada com anormalidade
	mg/dl	IC 95%	%	IC 95%	%	IC 95%	
Colesterol total							
População geral	148,1	147,1-149,1	24,2	22,7-25,8	20,1	19,0-21,3	2.940.705
Homens	143,6	142,4-144,8	22,7	20,4-25,2	15,3	13,9-16,9	1.256.102
Mulheres	152,6	151,4-153,9	25,7	24,5-27,0	24,9	23,4-26,5	1.684.602
12-14 anos	149,4	148,0-150,7	25,8	24,3-27,4	20,7	19,1-22,5	937.793
15-17 anos	147,1	145,8-148,3	22,8	20,8-24,9	19,6	18,0-21,2	2.002.911
LDLc							
População geral	85,3	84,5-86,1	19,5	18,5-20,5	3,5	3,2-4,0	1.526.733
Homens	83,4	82,2-84,5	17,4	16,0-18,9	2,9	2,3-3,6	669.805
Mulheres	87,2	86,3-88,1	21,5	20,2-22,9	4,3	3,7-4,9	856.928
12-14 anos	86,2	85,1-87,3	20,6	19,0-22,4	3,7	3,1-4,4	467.877
15-17 anos	84,5	83,5-85,5	18,4	17,2-19,7	3,4	2,9-4,1	1058.856
Triglicerídeos							
População geral	77,8	76,5-79,2	12,0	11,0-13,0	7,8	7,1-8,6	1.312.329
Homens	76,4	74,7-78,1	10,9	9,8-12,2	7,6	6,5-8,8	610.449
Mulheres	79,3	77,8-80,7	13,0	11,8-14,2	8,1	7,3-9,0	701.880
12-14 anos	78,9	76,7-81,0	12,7	11,0-14,6	8,3	7,2-9,5	434.638
15-17 anos	76,9	75,8-78,1	11,3	10,2-12,4	7,4	6,6-8,4	877.690
HDLc	Médio		Baixo				
População geral	47,3	46,7-47,9	46,8	44,8-48,9	-	-	3.104.161
Homens	44,9	44,4-45,5	55,9	53,7-58,2	-	-	1.256.003
Mulheres	49,6	48,9-50,3	37,8	35,4-40,2	-	-	1.848.158
12-14 anos	47,4	46,7-48,1	45,0	42,3-47,8	-	-	819.980
15-17 anos	47,2	46,4-48,0	48,4	45,9-50,8	-	-	2.284.181

a: alteração = níveis borderline + altos. b: estimativas populacionais foram obtidas do processamento de microdados do Censo Demográfico do IBGE 2000 e 2010. Modificado de Faria JR Neto et al. ^276^

•Para esta mesma faixa etária, 1200 adolescentes foram avaliados no Distrito Federal em 2022 (idade média 14,8 anos, 50,4% sexo feminino, 35,6% brancos, 53,5% pardos e 6% negros). A prevalência de CT ≥ 170 mg/dL foi de 30,6% (IC 95% 27,6-33,7), LDLc ≥110 mg/dL 21,3% (IC 95% 19,0-23,7), HDLc ≤ 45 mg/dL 41,8% (IC 95% 38,1-45,4) e TG≥ 90 mg/dL foi de 30,5% (IC 95% 27,4-33,8). Como no estudo ERICA, a alteração mais frequente foi a de baixos níveis de HDLc, com valores muito semelhantes.^277^

•Em Santa Catarina, 1.011 estudantes entre 6 a 14 anos (52,4% meninas) foram avaliados em 2013, quanto ao perfil lipídico, encontrando-se os seguintes níveis médios: CT, 172 (± 27) mg/dl em meninas e 170 (± 28) mg/dl em meninos; LDLc, 104 (± 24) mg/dl em meninas e 104 (± 27) mg/dl em meninos; HDLc, 49 (± 11) mg/dl em meninas e 49 (± 11) mg/dl em meninos; e TG, 80 (24-459) mg/dl em meninas e 77 (14-752) mg/dl em meninos.^278^

•Para uma faixa etária semelhante, 511 crianças (idade, 6 a 9 anos; 46,77% sexo masculino) foram avaliadas em 2016, em Vitória (ES) quanto a alterações do perfil lipídicos. Nesta população 32,7% tinham níveis altos de CT, 9,2% de LDLc, 4,1% de TG e 27% de baixos níveis de HDLc.^279^ Outro estudo conduzido em 2012 na cidade de Salvador, avaliando 1.131 crianças (idade, 7 a 15 anos; 50,1% sexo masculino), identificou dislipidemia (CT ≥ 170 mg/dl e/ou TG ≥ 130 mg/dl) em 25,5% (IC 95%, 22,7 – 28,3) delas. A dislipidemia estava associada com excesso de peso corporal (OR: 3,40; IC 95%, 2,07-5,58) e consumo moderado a elevado de alimentos de alto risco como laticínios integrais, fritura, gordura de origem animal e alimentos processados (OR: 1,49; IC 95%, 1,01-2,19).^279^

### Adultos

•Para a população brasileira adulta, os dados da PNS 2014-2015 em estudo de Malta *et al* .^275^ revela uma prevalência de níveis altos de CT de 32,7%, LDLc de 18,6%, e níveis baixos de HDLc, de 31,8%. Esse estudo identificou os seguintes níveis médios: CT, 185 mg/dl; HDLc, 46 mg/dl; e LDLc, 105 mg/dl. A prevalência de CT elevado foi maior nas mulheres, mas a prevalência de HDLc baixo foi maior nos homens. As **Tabelas 8-2 a 8-4** sintetizam esses achados e apresentam análise estratificada por sexo, para diferentes grupos etários, nível educacional, cor da pele e região do país. Em geral, níveis educacionais mais altos foram relacionados a menor prevalência de níveis elevados de CT e LDLc, assim como de níveis baixos de HDLc. Os grupos etários mais avançados apresentaram maior prevalência de níveis elevados de CT e LDLc. Residir nas regiões Sul e Sudeste do Brasil esteve relacionado a menor prevalência de níveis baixos de HDLc. Uma relação significativa entre cor de pele autorrelatada e perfil lipídico foi menos clara, mas mulheres negras apresentaram menor prevalência de níveis baixos de HDLc.^275 , 280^ Sobrepeso e obesidade também estiveram, nesta população, associados ao LDLc aumentado.^281^ Outros fatores associados com alterações relatadas no perfil lipídico da população brasileira incluem atividade física^282^ e variações sazonais.^283^

•A PNS de 2019 utilizou o diagnóstico autorreferido de colesterol alto e nos 88.531 adultos avaliados, identificou-se uma prevalência de 14,6% de colesterol alto. Os fatores mais fortemente associados a esta condição, medidos pela sua razão de prevalência (RP) foram: Sexo feminino (RP = 1,44; IC95% 1,40;1,52), idade ≥ 60 anos (RP = 3,80; IC95% 3,06;4,71), ter plano de saúde (RP = 1,33; IC95% 1,24;1,42), autoavaliação de saúde ruim ou muito ruim (RP = 1,75; IC95% 1,60;1,90), ter hipertensão (RP = 1,78; IC95% 1,68;1,89), ter diabetes (RP = 1,54; IC95% 1,45;1,65), ter insuficiência renal (RP = 1,33; IC95% 1,15;1,53), ter obesidade (RP = 1,27; IC95% 1,18;1,36), ser ex-fumante (RP = 1,13; IC95% 1,07;1,20), consumir álcool abusivamente (RP = 1,11; IC95% 1,01;1,21), ser ativo no lazer (RP = 1,22; IC95% 1,15;1,30).^284^

•O estudo ELSA-Brasil em 2016 apontou as seguintes frequências em mulheres e homens, respectivamente: hipertrigliceridemia, 23,2% e 40,7%; níveis baixos de HDLc, 20,7% e 14,7%; e níveis altos de LDLc, 57,6% e 58,8%. Além disso, o estudo ELSA-Brasil identificou pequenas diferenças no perfil lipídico de acordo com a cor da pele, cujo impacto clínico parece limitado.^285^

•Em estudo de 2023, Fonseca e colaboradores reportaram sobre fatores de risco para DCV no âmbito de atenção da rede primária de saúde em 7724 indivíduos sem DCV estabelecida de residentes em 32 municípios do estado de São Paulo. A prevalência de dislipidemia (definida por menção em prontuário) foi de 70,1% (68,6% em homens e 71,2% em mulheres).^286^

## Risco Atribuído

•Os dados mais recentes de risco de morte ou carga de doença (DALYs) atribuídos ao colesterol LDL elevado estimados pelo estudo GBD estão publicados na versão anterior deste documento.^4^ De forma resumida, para mortalidade, em âmbito nacional, entre 1990 e 2019, a mortalidade cardiovascular atribuída a altos níveis de LDLc no Brasil aumentou em números absolutos de 68.327 (IC 95%, 55.097-83.768) para 99.375 (II 95%, 78.039-126.143), mas a taxa padronizada por idade diminuiu em 51,3%, passando de 88,6 (II 95%, 67,8-114,8) para 43,1 (II 95%, 33,4-55,9) por 100.000, como resultado do envelhecimento da população.^4^

## Hipercolesterolemia Familiar

•A prevalência de hipercolesterolemia familiar pode ser estimada utilizando-se os critérios da Dutch Lipid Clinic Network. Com esta abordagem, no estudo ELSA-Brasil documentou-se prevalência de 1 em 263 indivíduos, sendo maior nos indivíduos de cor de pele negra (1 em 156) e parda (1 em 204) do que branca (1 em 417).^287^

•A despeito das controvérsias no uso de triagem em cascata para identificar parentes de indivíduos com hipercolesterolemia familiar, um estudo brasileiro demonstrou que 59% dos familiares de indivíduos com mutações eram portadores das mesmas mutações, sugerindo uma alta prevalência de hipercolesterolemia familiar no subgrupo selecionado.^288^ No entanto, em um estudo de Coutinho e colaboradores, envolvendo pacientes idosos hipercolesterolêmicos identificados por triagem em cascata, a presença de DCV prévia foi um preditor para DCV incidente enquanto a presença de uma variante genética associada a hipercolesterolemia familiar não foi, quando comparada a pacientes com idade e níveis de colesterol semelhantes.^289^

•Quanto à conscientização sobre hipercolesterolemia familiar e seu tratamento, Santos *et al* . relataram resultados de um banco de dados com 70.000 indivíduos submetidos a avaliação de saúde rotineira e obrigatória patrocinada pelo empregador em um hospital privado de São Paulo.^290^ Dos 70.000 indivíduos, 1.987 atendiam aos critérios estabelecidos para hipercolesterolemia familiar (LDLc ≥ 190 mg/dl ou LDLc ≥ 160 mg/dl em uso de estatina). Uma amostra de 200 foi selecionada para completar o questionário. Desses 200 indivíduos, o médico assistente suspeitou de hipercolesterolemia familiar em apenas 29 (14.5%), embora a maioria deles (97%) conhecesse seus altos níveis séricos de colesterol. Apenas 18% tinham a percepção de seu alto risco para DCV, 30% conheciam seus alvos de LDLc e 37% não utilizavam medicação hipolipemiante.

## Conhecimento, Tratamento e Controle

•Em análise de registros obtidos da “Estratégia de Saúde da Família “, dados de mundo real sobre o uso de estatinas na prevenção secundária em 2133900 indivíduos de todo território nacional foram reportados.^91^ Desses sujeitos, 35103 (idade média 66,2 anos, 49,5% sexo masculino) haviam tido um IAM prévio ou um acidente vascular encefálico. Nessa população o uso de estatinas foi da ordem de 6.7% somente. A taxa cai para 0,6% quando avaliado o uso de estatinas de alta potência. Ter tido um IAM prévio ou fazer uso de anti-hipertensivos, ter hipertensão esteve associado a maior chance de uso dos hipolipemiantes com OR 4.53(IC95%3.66–5.60) e OR5.47(IC95%4.60–6.47) respectivamente. Ser da região sudeste também foi um forte preditor de uso (OR4.53[IC95%3.66–5.60]).^91^

•Análise conduzida no estudo ELSA-Brasil, incluindo 15.096 adultos com idade de 35-74 anos, explorou a prevalência de níveis elevados de LDLc (de acordo com os critérios NCEP-ATP-III) e a proporção de participantes conhecedores de seu diagnóstico.^291^ A frequência de participantes com níveis elevados de LDLc foi 45,5%, dos quais, apenas 58,1% conheciam seu diagnóstico. Dos participantes com níveis elevados de LDLc, 42,3% se tratavam com medicações hipolipemiantes e 58,3% alcançaram o alvo definido pelo painel NCEP-ATP-III.

•Em uma análise baseada na PNAUM entre 2014 e 2015, avaliou-se o uso de estatinas na atenção primária do SUS nas cinco regiões brasileiras.^290^ Entre os 8.803 respondentes, a prevalência de uso de estatina foi 9,3%, sendo que 81,4% desses usuários relataram ter dislipidemia. Sinvastatina foi a mais usada (90,3%), seguida por atorvastatina (4,7%) e rosuvastatina (1,9%)

•Em relação a adequação do tratamento e das metas de controle em contexto de prevenção secundária, um estudo conduzido no sistema de saúde da cidade de Curitiba acompanhou 7066 sujeitos internados devido a IAM entre 2008 e 2015. Dentre eles, 1451 tinham pelo menos uma medida de LDLc e dados de seguimento ambulatorial. O LDLc médio encontrado foi 93,3mg/dl. Apenas 7,4% possuíam o LDLc < 50 mg/dL, 21,5% entre 50 e 70 mg/dL, 35,2% entre 70-100 mg/dL e 35,5% (grupo de maior prevalência) > 100 mg/dL. Dentre aqueles que possuíam medidas de LDLc antes e depois do infarto, notou-se uma redução dos níveis de LDLc mas ainda aquém do preconizado (de 128 para 92 mg/dl, com redução relativa de 24,3% dos níveis de colesterol, e apenas 19,3% dos indivíduos obtendo redução > 50%).^291^

•Em análise transversal do Estudo Brasileiro de Diabetes na cidade de Campinas, 1030 participantes (idade média 58 anos, 54% homens) foram avaliados quanto a adequação do perfil lipídico ao grupo de risco cardiovascular pertencente (muito alto, alto e moderado). Os níveis médios de LDLc foram 105 ± 38, 109 ± 39 e 110 ± 37 respectivamente com apenas 18,8% sendo considerados com níveis de LDLc adequados à categoria de risco pertencente.^292^

•No já mencionado estudo de Fonseca e colaboradores no estado de São Paulo, apenas 13,9% dos indivíduos considerados dislipidemicos estavam usando hipolipemiantes e conseguiram atingir níveis séricos de LDLc < 100mg/dl.^286^

## Dislipidemia e Aterosclerose Subclínica

•Aterosclerose subclínica, incluindo marcadores como escore de cálcio coronariano e espessura médio-intimal da carótida, foi utilizada como substituto para aterosclerose e, portanto, sua associação com perfil lipídico anormal pode ser de interesse epidemiológico.^293^

•Em um estudo com mais de 3.600 indivíduos, Generoso *et al* . demonstraram que HDLc estava associado com calcificação na artéria coronária mesmo após ajuste para os fatores de risco cardiovascular tradicionais. Entretanto, essa associação não permaneceu significativa após ajuste para TG.^294^ Esse estudo também avaliou as frações de HDLc e mostrou que elas não estavam associadas com calcificação na artéria coronária após ajuste para HDLc total. Além disso, o mesmo grupo demonstrou a associação entre HDLc e a espessura médio-intimal da carótida e ainda que tal associação é modificada pela presença de diabetes.^295^

•Laurinavicius *et al* . estudaram a associação entre níveis muito altos de HDLc (acima de 90 mg/dL) e espessura médio-intimal da carótida. Tais níveis muito altos de HDLc podem caracterizar hiperalfalipoproteinemia, uma condição disfuncional do HDLc. A despeito de evidência anterior, esses autores não mostraram associação entre esse perfil e a espessura médio-intimal da carótida.^296^

•Em uma análise das lipoproteínas ricas em TG no estudo ELSA-Brasil, Bittencourt *et al* . mostraram que essas partículas estão associadas com a presença de calcificação na artéria coronária mesmo após ajuste para fatores de risco significativos.^297^

•Em um estudo com octogenários brasileiros, os autores descobriram que a associação entre LDLc alto e calcificação na artéria coronária enfraquece com a idade, enquanto a associação com HDLc baixo não.^298^

•Em conjunto, esses estudos demonstram a robusta associação entre perfil lipídico desfavorável e aterosclerose subclínica, corroborando achados da associação entre dislipidemia e DCV.

## Custo em Saúde atribuído à dislipidemia

•A única publicação encontrada acerca de custos em saúde atribuída à dislipidemia refere-se aos custos estimados por hospitalizações por doença arterial coronariana atribuída à hipercolesterolemia familiar.^298^ Esta avaliação, utilizou dados do Sistema de Internação Hospitalar do SUS de 245981 internações/ano por doença arterial coronariana. Foi estimada a prevalência de hipercolesterolemia familiar a partir de dados internacionais (0,4% e 0,73% Estados Unidos e Dinamarca respectivamente). Neste cenário, o custo anual estimado atribuível à hipercolesterolemia familiar foi de $17.650972,00 (para prevalência de 0.4%) a $31.448466,00 (para prevalencia de 0.73%).^299^

## Direções Futuras

•Dados atuais sobre a epidemiologia da dislipidemia na população brasileira contemporânea são limitados. Estudos adicionais sobre a prevalência de dislipidemia na população geral, assim como em grupos específicos de alto risco, como aqueles de nível socioeconômico mais baixo, são necessários.

•A frequência de rastreio, tratamento e controle da dislipidemia no Brasil, de acordo com sexo e grupos etários, precisa ser investigada. Assim como o impacto da dislipidemia no sistema de saúde, incluindo custos, ainda não foram avaliados de maneira mais abrangente.

•Estudos contextualizando o papel dos marcadores de aterosclerose subclínica na prática médica e o efeito do uso desses marcadores substitutos de aterosclerose na modificação do tratamento clínico, assim como da custo-efetividade desta utilização são necessários.

## CAPÍTULO 9 – DIABETES MELLITUS

### CID-10 E10 a E14; CID-10-CM E8 a E13

**Table:** Abreviaturas usadas no Capítulo 9

BINDER	BrazIliaN Type 1 & 2 DiabetEs Disease Registry
CID-10	Classificação Internacional das Doenças, 10ª Revisão
COVID-19	Doença do novo coronavírus 2019
DALYs	Anos de vida perdidos ajustados por incapacidade (do inglês, Disability-Adjusted Life-Years)
DATASUS	Departamento de Informática do Sistema Único de Saúde
DCV	Doença Cardiovascular
ELSA-Brasil	Estudo Longitudinal de Saúde do Adulto - Brasil
ERICA	Estudo de Riscos Cardiovasculares em Adolescentes
GBD	Carga Global de Doenças (do inglês, Global Burden of Disease)
HbA1c	Hemoglobina glicada
HR	Hazard Ratio
IBGE	Instituto Brasileiro de Geografia e Estatística
IC	Intervalo de Confiança
IDF	Federação Internacional de Diabetes (do inglês, International Diabetes Federation)
II	Intervalo de Incerteza
IMC	Índice de Massa Corporal
LDL	Lipoproteína de Baixa Densidade (do inglês, Low-Density Lipoprotein)
OR	Odds Ratio
PNS	Pesquisa Nacional de Saúde
PR	Prevalence ratio
SIH	Sistema de Informações Hospitalares
SIVEP-Gripe	Sistema de Informação da Vigilância Epidemiológica da Gripe
SUS	Sistema Único de Saúde
VIGITEL	Sistema de Vigilância de Fatores de Risco e Proteção para Doenças Crônicas por Inquérito Telefônico

### Introdução

•Diabetes melito é uma doença caracterizada por hiperglicemia crônica devida à menor secreção e/ou resistência à ação da insulina, o que em longo prazo pode levar a complicações microvasculares (retinopatia, doença renal do diabetes, neuropatia) e macrovasculares (doença arterial coronariana, doença cerebrovascular e doença arterial obstrutiva periférica). Pré-diabetes (tolerância diminuída à glicose e glicemia de jejum alterada) é a condição em que há hiperglicemia sem atingir critérios diagnósticos para diabetes, o que determina maior risco de diabetes no futuro e aumento de risco de DCV.^300^

•Neste capítulo, o diabetes mellitus será abordado como fator de risco cardiovascular,^301^ assim como na versão anterior desse documento,^4^ já que sua presença, associada ao tabagismo, hipertensão arterial sistêmica e dislipidemia, aumenta em duas a três vezes o risco de DCV.^302^

### Prevalência

•Dados recentes do VIGITEL (2021) mostram que 9,1% (IC 95% 8,5-9,8%) dos adultos referem diagnóstico de diabetes, o que é semelhante entre homens e mulheres. Belo Horizonte é a capital com a maior prevalência (11,3%, IC 95% 9,0-13,5%) e Rio Branco a com menor prevalência (6,4%, IC 95% 4,5-8,3%). Considerando anos de educação, quanto menor a escolaridade, maior a prevalência de diabetes (< 8 anos: 17,7%, IC 95% 16-19,4%; >12 anos: 5,1%, IC 95% 4,3-5,9%). Quanto maior a faixa etária considerada, maior a prevalência de diabetes (>65 anos: 28,4%, IC 95% 26,5-30,2%).^252^

•De acordo com dados da IDF, o Brasil é o sexto país no mundo em quantidade de adultos com diabetes (15,73 milhões; IC 95% 14,04-17,43), com uma prevalência de 10,5% (IC 95% 9,4-11,6), dos quais 31,9% desconheciam ter a doença. A redução de casos desconhecidos observada em relação aos dados anteriores^4 , 303^ pode dever-se à elevada taxa de rastreamento, como observado na PNS do IBGE, pois, em 2013, 11,6% (IC 95% 11,1-12,1) dos adultos nunca tinham medido sua glicemia, o que foi reduzido para 6,2% em 2019 (IC 95% 5,9-6,5).^304^ Maior acesso ao diagnóstico foi observado em mulheres (PR=1,16; 1,15-1,17), idosos (PR=1,25; 1,22-1,28) e naqueles com maior nível educacional (PR=1,17; 1,15-1,18), obesidade (PR=1,06; 1,05-1,08) e hipertensão (PR=1,12; 1,11-1,13). Por outro lado, menor acesso ao diagnóstico foi observado em autodeclarados pretos (PR=0,97; 0,95-0,99), em indivíduos que residiam em áreas rurais (PR=0,89; 0,87-0,90) e naqueles sem plano de saúde privado (PR=0,85; 0,84-0,86).^305^

•A prevalência de pré-diabetes foi de 10,8% (IC 6,9-12,8) ou 17,77 milhões de pessoas (IC 11,46-21,04).^303^ Esses números são menores do que os descritos pela mesma fonte em 2019,^303^ o que é diferente do constante crescimento da prevalência de diabetes^306^ e pré-diabetes descrito previamente.^307^ Importante observar que os números dependem do método utilizado para diagnóstico, sendo menores quando o diagnóstico é autorreferido e maiores nos estudos utilizando confirmação com exame laboratorial, especialmente nos que empregaram teste oral de tolerância à glicose^306^ou HbA1c.^6^ Em qualquer um dos critérios, a prevalência foi maior em mulheres, em indivíduos acima de 30 anos e naqueles com sobrepeso ou obesidade. Maior nível educacional associou-se com menor prevalência de diabetes. A região brasileira com maior prevalência foi a Centro-Oeste.^6^

•Dados da PNS 2019 mostraram que a prevalência autorrelatada de diabetes foi maior nas pessoas com menor nível educacional, como segue: 12,9% (analfabetos/ensino básico incompleto), 6,3% (ensino básico completo/ensino médio incompleto), 4,6% (ensino médio completo/ensino superior incompleto) e 4,65% (ensino superior completo).^6^ Da mesma forma, pessoas sem plano de saúde privado tiveram maior prevalência de diabetes autorrelatado [PR=1,88; IC 95% 1,22-2,89).^308^

•O estudo PURE/América do Sul mostrou prevalência de diabetes (glicemia de jejum > 126 mg/dL ou história pessoal de diabetes) na linha de base do estudo de 9%. Dos 24.718 participantes, 5.661 eram do Brasil, da zona urbana (65,1%) e rural de São Paulo, mas dados específicos para o Brasil não foram apresentados.^309^

•Os dados do ERICA, grande estudo transversal desenvolvido em amostra representativa de estudantes brasileiros de 12 a 17 anos (n=37.854), ainda são os mais atuais quanto à prevalência de diabetes tipo 2 (3,3%, IC 95% 2,9-3,7) e pré-diabetes (22,0%, IC 95% 20,6-23,4).^310^ Dessa forma, o Brasil ocupa as primeiras posições em número de casos, juntamente com o México.^303^

•O Brasil é o terceiro no mundo em número de casos de diabetes tipo 1 considerando todas as idades (588.800 indivíduos) e a faixa etária até 20 anos de idade (112.240 indivíduos). A diferença de prevalência entre os países, especialmente entre faixas etárias, reflete populações mais jovens e maior mortalidade em países de baixa renda (Índia, Argélia, Marrocos e Turquia), o que reduz o número de adultos com diabetes tipo 1.^303^

### Incidência

•O estudo multicêntrico brasileiro ELSA-Brasil reportou incidência cumulativa de diabetes de 2,0/100 pessoas-ano (IC 95% 1,8-2,1), maior nos idosos [2,8%; IC 95% 2,3-3,4], naqueles com obesidade (3,8%; IC 95% 3,4-4,3) e com menor nível educacional (3,0%; IC 95% 2,6-3,6).^311^

•O número de crianças e adolescentes com diabetes tipo 1 tem crescido em todo o mundo, aproximadamente 3% ao ano, embora com importantes diferenças regionais.^303 , 312 - 314^ O Brasil segue sendo o terceiro no *ranking* mundial em número de novos casos de crianças e adolescentes de 0 a 19 anos com diabetes tipo 1 (8,9 casos/1000/ano).^303^ A incidência anual geral de diabetes tipo 1 na cidade de Bauru (São Paulo), de 1986 a 2015, em crianças ≤ 14 anos foi de 12,8 (IC 95% 11,2-14,4) por 100 mil, variando de 2,8 em 1987 a 25,6 em 2013, sem diferença entre sexos.^310^

### Mortalidade

#### Mortalidade geral atribuível ao diabetes

•Cardoso *et al.* , com base em dados do DATASUS do Ministério da Saúde, avaliaram a mortalidade precoce de adultos de 30-69 anos, entre 2010 e 2017. A taxa média anual de mortalidade causada por diabetes foi 28,1 (27,8-28,4) por 100 mil habitantes entre 2010 e 2012, enquanto, de 2015 a 2017, foi de 27,0 (26,7-27,3) por 100 mil habitantes. As maiores taxas foram observadas no Nordeste [34,4 (33,9-35) por 100 mil habitantes entre 2015 e 2017] e as menores, na região Sul [20,4 (20,0-20,9) por 100 mil habitantes entre 2015 e 2017].^315^

•A mortalidade precoce (30 a 69 anos) também foi avaliada por Cousin *et al.* de 1990 a 2019, utilizando estimativas calculadas pelo Estudo GBD. A causa de mortalidade precoce padronizada pela idade foi determinada pelo diabetes em 33,0 (32,0-34,2) por 100 mil habitantes em 1990, em 26,8 (25,9-27,6) por 100 mil habitantes em 2010, e em 24,8 (23,7-25,9) por 100 mil habitantes em 2019, o que representou redução de 1,0% entre 1990 e 2010 e de 0,9% entre 2010 e 2019.^316^

•Os demais dados do Estudo GBD mais recente ainda não estão disponíveis, sendo que os dados da última versão do Estudo GBD 2019 estão descritos na Estatística Cardiovascular 2021.^4^

•A 10ª edição do IDF Diabetes Atlas, publicada em 2021, descreve 214.175 mortes atribuíveis ao diabetes no Brasil em adultos de 20-79 anos, número esse ligeiramente mais elevado do que o apresentado no Estudo GBD 2019.^303^

•Arrais KR *et al.* analisaram o perfil das internações e da mortalidade hospitalar por diabetes mellitus no Piauí entre 2015 e 2019. Nesse estado, foram registradas 18.361 hospitalizações por diabetes mellitus, das quais 527 evoluíram a óbito, o que representa uma taxa de mortalidade hospitalar de 2,87 por cada 100 internações. Na caracterização das hospitalizações, assim como dos óbitos, ambos predominaram em mulheres, pardas e idosas.^317^

#### Mortalidade cardiovascular atribuível ao diabetes

•Os dados do Estudo GBD mais recentes ainda não estão disponíveis, sendo que os da última versão do Estudo GBD 2019 estão descritos na Estatística Cardiovascular 2021.^4^

## Carga de Doença

### Carga de doença atribuível ao diabetes

•Os dados do Estudo GBD 2021 estimaram para o Brasil em 2021 uma contagem de DALYs atribuíveis ao diabetes de 2.740.000 (2.370.000-3.160.000), com uma variação positiva de 159,7% (II 95% 149,2-172,1) entre 1990 e 2021. No entanto, a taxa de DALYs atribuíveis ao diabetes padronizada por idade por 100 mil habitantes no Brasil foi estimada em 1.075,2 (931,4-1.239,0), uma queda de -2,7% (-7,1 a 2,1) entre 1990 e 2021. Em conjunto, esses dados refletem o efeito do crescimento e envelhecimento populacional do Brasil na carga de doença atribuível ao diabetes no período.^318^

***Carga de doença cardiovascular atribuível ao diabetes***

•Os dados do Estudo GBD mais recentes ainda não estão disponíveis, sendo que os da última versão do Estudo GBD 2019 estão descritos na Estatística Cardiovascular 2021.^4^

### Impacto na saúde cardiovascular

•Estudos prévios brasileiros, detalhados na versão anterior deste documento, mostraram que o diabetes está relacionado a diferentes desfechos cardiovasculares adversos e marcadores subclínicos de aterosclerose.^4 , 319 , 320^

•Dynkoski *et al* ., usando dados do SIH do DATASUS e analisando internações de idosos por condições sensíveis à Atenção Primária no período de 2008 a 2014 no estado de Santa Catarina, mostraram que diabetes mellitus foi a 5ª causa de internação (6,75%).^319^

•Mosenzon *et al.,* no estudo CAPTURE, reportaram a prevalência de DCV estabelecida, avaliada por registro em prontuários de centros de atenção primária e terciária em adultos com diabetes tipo 2 em vários países (n=9.823), incluindo o Brasil. Dentre os 912 indivíduos avaliados no Brasil, 43,9% (IC 95% 40,9-46,8) tinham DCV, número maior do que o estimado para a amostra total dos 13 países avaliados [prevalência ponderada estimada de DCV de 34,8% (IC 95% 32,7-36,8)]. A insuficiência cardíaca estava reportada em 113 pacientes [12,4 (IC 95% 10,4-14,4)] e o Brasil teve a segunda maior prevalência entre os 13 países avaliados.^320^

## Conhecimento, tratamento e controle da condição

•O tratamento do diabetes baseia-se em três pilares: dieta, exercício físico e medicamentos (antidiabéticos orais e insulina). Adesão ao tratamento é importante determinante do controle metabólico. Gomes e Negrato relataram adesão mínima ao tratamento em 48,0% de pacientes, associando-se a HbA1c de 8,6% ± 1.9%.^321^ Outros estudos mostraram elevado acesso a medicamentos, porém baixa adesão ao tratamento [17,2% (IC 95% 14,6-20,1)].^322^Foram associados à melhor adesão: menor idade, menor IMC, presença de complicações macrovasculares e melhor desempenho ocupacional e no domínio emocional do questionário de qualidade de vida - SF-36.^323^

•Silva *et al.* em 2018 realizaram inquérito domiciliar em 63 municípios de Minas Gerais selecionados por conveniência, com o objetivo de analisar o perfil de utilização de medicamentos em pacientes com diabetes naquele estado. Mostraram que, entre as 2.619 pessoas com diabetes avaliadas, os medicamentos mais frequentes em uso foram metformina, losartan, glibenclamida e sinvastatina; 56,5% dos entrevistados estavam em polifarmácia (uso de cinco ou mais medicamentos).^324^ A prevalência de uso de antidiabéticos orais no Brasil passou de 77,4% (IC 95% 74,3-80,1) em 2012 para 85,2% (IC 95% 82,8-87,2) em 2018.^325^

•Uma coorte retrospectiva (n = 488 pacientes com diabetes) mostrou que apenas 7,3% dos pacientes com diabetes na atenção primária e 27,0% na atenção terciária tinham avaliados pelo menos 50% dos indicadores de qualidade de atendimento (avaliação anual de doença renal do diabetes, retinopatia e neuropatia diabéticas, perfil lipídico, avaliação nutricional e abordagem de cessação de fumo).^326^

•Em 2022, utilizando dados do SIH, DATASUS, Macedo Junior *et al.* realizaram estudo epidemiológico descritivo com análise temporal de internações de idosos no estado de Rondônia de 2015 a 2019. Das 23.844 internações (11% do total) no período, diabetes foi identificado como causa da internação em 267 (6,96%) em 2015 e em 349 (7,23%) em 2019, mantendo a segunda posição nos dois períodos, apenas atrás de pneumonia.^327^

•O Registro BINDER foi um estudo multicêntrico prospectivo em 43 cidades brasileiras que buscou avaliar o controle glicêmico de pacientes com diabetes em cenário de mundo real. Os 1142 participantes foram acompanhados de 2017 em diante por 2 anos. No basal, a HbA1c era 7,1% (4,1 – 15,0%); 396 (48,2%) pacientes tinham HbA1c ≤ 7,0%. Ser mais jovem (p = 0,014), ter menor nível educacional (p = 0,025) e ser atendido em serviço público (p = 0,0058) estavam associados à HbA1c elevada. No seguimento não houve diferença na HbA1c. Não houve diferença quanto ao uso dos diferentes antidiabéticos orais entre o basal e o seguimento, exceto para menor uso de metformina (p = 0,0044) e aumento do uso de inibidores de SGLT-2 (p < 0,001).^328^

•Uma análise transversal da população com diabetes do ELSA-Brasil reportou o controle metabólico e de fatores de risco dessa população. Dos 2.062 indivíduos com diabetes, 1.364 (66,1%) tinham HbA1c <7,0%, 1596 (77,4%) tinham adequado controle pressórico e 1.086 (52,7%) tinham LDL-colesterol no alvo (<100 mg/dL ou <70 mg/dL se alto risco cardiovascular). Mulheres (PR=1,13; 95% CI 1,07–1,20), pessoas acima de 74 anos de idade (PR=1,20; 95% CI 1,08–1,34) e aqueles com maior renda per capita (PR=1,26; 95% CI 1,10–1,45) tiveram maior probabilidade de melhor controle glicêmico. Indivíduos autodeclarados negros (PR=0,91; 95% CI 0,83–1,00) ou com maior duração do diabetes (PR=0,43; 95% CI 0,39–0,47) tiveram menor probabilidade. Mulheres (PR=1,05; 95% CI 1,00–1,11) e aqueles com seguro de saúde privado (PR=1,15; 95% CI 1,07–1,23) tinham mais probabilidade de alcançar duas ou mais das três metas; enquanto aqueles autodeclarados negros (PR=0,86; 95% CI 0,79–0,94) e com maior duração do diabetes (PR=0,68; 95% CI 0,63–0,73) tinham menor probabilidade.^329^

•Machline-Carrion *et al.* conduziram estudo transversal analisando dados rotineiramente registrados de atendimentos médicos de 2.133.900 indivíduos na atenção primária, idade média 66,2 ± 14,6 anos, 49,5% homens, 29,6% brancos, no Brasil entre 2016 e 2021. Desses, 6,7% (2346) tinham registro de prescrição de estatinas, o que foi mais provável naqueles com idade acima de 60 anos (OR 1,32 [95% CI 1,19–1,47), que viviam na região Sudeste (OR 4,53 [95% CI 3,66–5,60]), que tinham história de infarto agudo do miocárdio (OR 4,53 [95% CI 3,66–5,60]) e diabetes (OR 1,50 [95% CI 1,37–1,64]).^89^

•Malta D *et al.* , em 2022 avaliaram indicadores de cuidado de pessoas com diabetes a partir de dados da PNS de 2013 (n = 60.202), comparando-os com os obtidos da mesma base em 2019 (n = 88.531). Houve aumento no uso de medicações (80,2% para 88,8%) e pessoas que receberam tratamento médico (73,2% para 79,1%), mas manutenção do percentual de pessoas que realizaram rastreamento para retinopatia diabética (35,3% *vs.* 36,7%) e que tiveram avaliação dos pés (29,1% *vs.* 31,7%). Hospitalizações por complicações relacionadas ao diabetes foram menos frequentes em mulheres nesse período [13,1 (11,5-15,0) *vs.* 16,5 (14,3-19,1); PR 0,80 (IC 95% 0,65-0,97)]. Além disso, em 2019, os indicadores foram piores em homens, pessoas mais jovens, de cor preta e com menores níveis socioeconômicos e educacionais. Quanto às diferenças regionais, Sudeste, Sul e Centro-Oeste tiveram maior proporção de pessoas que obtinham medicamentos pela Farmácia Popular em relação à região Norte (56,4%, 59,1%, 56,4% vs. 35,6%, respectivamente). No Sudeste houve a maior proporção de pessoas que realizaram rastreamento da retinopatia diabética (40,8%) e menor número de hospitalizações (12,1%).^304^

## Fatores de risco e prevenção

•Dados de 151 estudos de prevalência de sobrepeso e obesidade em adolescentes brasileiros foram compilados em meta-análise mostrando aumento na prevalência de sobrepeso de 8,2% (IC 95% 7,7-8,7) até 2000, de 18,9% (IC 95% 14,7-23,2) de 2000 a 2009, e de 25,1% (IC 95% 23,4-26,8) de 2010 em diante, padrão que foi similar para a prevalência de obesidade. As regiões Sudeste e Sul tiveram maiores prevalências de sobrepeso e obesidade.^330^

•Em 37.892 adolescentes do ERICA, sobrepeso foi observado em 17,2%, obesidade em 5,6% e obesidade grave em 1,3%, com aumento da chance de desfechos cardiometabólicos adversos de acordo com o maior IMC, incluindo maior glicemia de jejum [RP 5,30 (IC 95% 1,94-14,50)] e HbA1c (2,04, IC 95% 1,29-3,25).^331^

•Na linha de base do ELSA-Brasil, a análise de 14.912 funcionários públicos brasileiros mostrou prevalência maior de diabetes entre pessoas com IMC de 25-29,9 kg/m^2^ (18,9%; IC 95% 18,0 - 19,9%) e acima de 30 kg/m^2^ (32,1%; IC 95% 30,6 - 33,6%) em comparação com aquelas com IMC ≤ 24,9 kg/m^2^ (11,7; IC 95% 10,9-12,6%).^281^ Considerando a atividade física durante o lazer, o ELSA-Brasil mostrou menor chance de ocorrência de diabetes em homens e mulheres ativos em relação aos inativos.^332^

•A partir de dados da segunda onda do ELSA-Brasil (2012-2014), foi realizada análise de 10.047 funcionários públicos brasileiros quanto à associação entre a distribuição da gordura corporal e a ocorrência de DCV e seus fatores de risco. Em análise multivariada, observou-se que a menor relação entre gordura nas extremidades/gordura no tronco foi protetora para a presença de diabetes em mulheres (0,04; IC 95% 0,01-0,19), mas não em homens (1,03; IC 95% 0,37-2,86).^333^

•Dados da PNS de 2019 com 86.678 adultos mostraram que assistir televisão por mais de 3 horas por dia aumentou a chance de desenvolver diabetes em comparação a menos de 3 horas por dia (3-6 horas/dia *vs.* referência OR 1,26; IC 95% 1,12-1,42; >6 horas/dia *vs.* referência OR 1,80; IC 95% 1,54-2,11; ajustes para sexo, faixa etária, etnia, nível educacional, consumo de álcool, tabagismo, consumo de refrigerantes e frutas, e atividade física de lazer). Por outro lado, não houve associação entre o uso de outras telas (computadores, *tablets* e telefones celulares) e a chance de desenvolver diabetes.^334^ A relação entre assistir televisão e a chance de desenvolver diabetes mostrou ser do tipo dose-resposta.^335^

•Teló j *et al* . mostraram, em estudo transversal com 37.854 adolescentes, maior chance de diabetes tipo 2 naqueles com obesidade (OR 1,59; IC 95% 1,20-2,11) e aumento da circunferência abdominal (OR 1,51; IC 95% 1,13-2,01), sem associação com inatividade física (< 60 min/dia).^310^

•Em amostra de 9909 participantes do estudo ELSA-Brasil, questionários de frequência alimentar foram utilizados para cálculo do índice inflamatório da dieta. Em regressão logística ajustada para fatores sociodemográficos e comportamentais e IMC, aquele índice foi preditor de maior ganho de peso (tercil 3 vs. 1: OR Z 1,30; 95% CI 1,08-1,55) e de incidência de diabetes (tercil 3 vs. 1: HR Z 1,26; 95% CI 1,04-1,52).^336^

## Custos

•Ferrari G *et al.* estimaram os custos diretos do cuidado à saúde relacionado às doenças crônicas não transmissíveis atribuíveis à obesidade no Brasil em 2019, mostrando que os custos totais do diabetes foram de US$ 44,2 milhões (de um total de US$ 2967,6 milhões). Considerando fração populacional atribuível de 63,1% (40,3 - 86,4), o custo atribuível ao diabetes seria de US$ 27,9 milhões (17,8-38,2).^337^

•Em 2022, Leal V *et al.* utilizaram dados do GBD 2019 e do DATASUS para estimar custos com o tratamento do diabetes e outras doenças crônicas não transmissíveis atribuíveis ao consumo de bebidas açucaradas no Brasil. Os resultados mostraram um total de US$14.116.240,55 em custos com hospitalizações por doenças crônicas não transmissíveis atribuíveis ao consumo de bebidas açucaradas, US$72.645,40 em mulheres e US$80.345,36 em homens com diabetes. Os custos ambulatoriais com diabetes atribuíveis ao consumo de bebidas açucaradas foram de US$4.645,79 em mulheres e US$4.641,06 em homens com diabetes. Dados por faixa etária podem ser encontrados na publicação original.^338^

•Em 2022, Pereda *et al.* utilizaram abordagem *cost-of-illness* para calcular a carga econômica total referente ao diabetes no Brasil em 2016, estimando até US$ 2,15 bilhões, dos quais US$1,52 bilhão (70,6%) eram custos indiretos relacionados a mortes prematuras, absenteísmo e aposentadoria precoce. Os custos indiretos relacionados às mortes prematuras foram de US$1,18 bilhão (77,9% dos custos indiretos). O total de custos diretos relacionados ao diabetes foi de US$633 milhões, US$232,8 milhões com hospitalizações e US$86 milhões com despesas ambulatoriais (95% cobertas pelo SUS). A farmácia popular representou US$304,2 milhões, enquanto as despesas *out-of-pocket* foram de US$10,0 milhões. O custo total por paciente diagnosticado no Brasil, considerando prevalência de 6,4% (dado de 2013), foi de US$223,54 (US$65,72 de custos diretos e US$157,81 de custos indiretos).^339^

•Os gastos relacionados ao diabetes em adultos (20-79 anos) foram de US$ 966 bilhões no mundo em 2019, sendo que o Brasil ocupa a terceira posição de maior gasto (US$ 42,9 bilhões).^303^

## Impacto da pandemia de COVID-19 sobre o diabetes mellitus

•Diabetes é fator de risco para piores desfechos relacionados à COVID-19. Pacientes com COVID-19 e diabetes têm maior risco de hospitalizações e morte em relação àqueles sem diabetes. Pior controle glicêmico, quando da ocorrência de COVID-19, determina piores desfechos.

•Niquini RP *et al.* , em 2020, utilizaram a base do SIVEP-Gripe para comparar os números de hospitalização por síndrome de angústia respiratória aguda, que foram 39.349 em 2019 (14,7% por influenza) e 94.807 em 2020 (33,7% por COVID-19). Desses, 16,2% e 24,7% tinham diabetes, respectivamente.^340^

•Nunes *et al* ., em 2021, utilizaram, além do SIVEP-Gripe, dados provenientes de *linkage* realizada pela Vigilância Epidemiológica Estadual da Bahia e Gerenciador de Ambiente Laboratorial (GAL), mostrando que, dos 7.286 óbitos por COVID-19 observados até novembro de 2020, a maioria foi em homens e 43,6% em pessoas com diabetes.^341^

•Um estudo transversal avaliou 21.942 brasileiros internados com COVID-19 até metade de 2020 também utilizando dados do SIVEP-gripe. Dentre os adultos (até 60 anos), obesidade associada ao diabetes e/ou DCV associou-se a maior prevalência de ventilação mecânica invasiva (PR 3,76; 95% CI 2,82-5,01) e não invasiva (PR 2,06; 1,58-2,69), admissão em unidade de terapia intensiva (PR 1,60; 1,40-1,83) e morte (PR 1,79; 1,45-2,21) *vs.* pacientes sem essas condições.^342^

•Prado *et al.* , em 2021, analisaram retrospectivamente todos os casos de COVID-19 notificados até setembro de 2020 (n = 57.700) e observaram mortalidade de 61,8/100 mil habitantes. Foram fatores de risco para o óbito: ser homem (HR=1,48; IC 95% 1,25-1,76), ser idoso (≥60 anos, HR=10,64; IC 95% 8,84-12,81) e apresentar multimorbidade (HR=2,23; IC 95% 1,77-2,81), o que incluiu diabetes mellitus.^343^

•Já Pietre *et al.* , em 2021, reportaram dados obtidos da mesma base até agosto de 2020 (n=181.964, 96.567 mortes), ressaltando maior risco de mortalidade por síndrome respiratória aguda grave em pacientes mais jovens com diabetes e autodeclarados negros (aOR 5,58, IC 95% 4,97-6,25; p<0,0001) e naqueles com obesidade ou doenças hematológicas (aOR 21,09, IC 95% 13,64-32,06) *vs.* seus controles.^344^

•Por fim, em estudo transversal (Garces T *et al.* 2022) realizado com dados de 397.600 indivíduos hospitalizados entre 2020 e 2021 (SIVEP-gripe), 32,0% deles evoluíram para óbito. A prevalência do óbito entre as pessoas com diabetes foi de 40,8% (n = 41.776), uma razão de prevalências ajustada da associação entre diabetes e óbito por COVID-19 de 1,15 (IC 95% 1,14-1,16), representando prevalência de óbito 15% mais elevada entre os brasileiros com diabetes hospitalizados por COVID-19.^345^

•Considerando a faixa etária menor de 20 anos, Oliveira *et al.* mostraram que, dentre 21.591 crianças e adolescentes hospitalizados por COVID-19, 379 (1,8%) tinham diabetes. Esses pacientes com diabetes tiveram mais internações em unidade de terapia intensiva (46,6% *vs* . 26,0%), mais necessidade de ventilação invasiva (16,9% vs. 10,3%) e maior mortalidade (15,0% vs. 7,6%; HR = 2,0, 95% CI 1,58-2,66) do que aqueles sem diabetes. Nesses indivíduos, morar em regiões mais pobres (Nordeste, HR, 2,17, 95% CI 1,18-4,01, Norte, HR 4,0, 95% CI 1,79-8,94), ter saturação de oxigênio < 95% na admissão (HR 2,97, 95% CI 1,64-5,36), ter doença renal (HR 3,39, 95% CI 1,42-8,09) e obesidade (HR 3,77, 95% CI 1,83-7,76) associaram-se a mortalidade.^346^

•Sardinha *et al.* , em 2021, realizaram estudo transversal em indígenas brasileiros, baseados em dados do DATASUS, mostrando que, até agosto de 2020, ocorreram 1.207 casos e 470 mortes por COVID-19 nessa população. Embora tenha sido uma das comorbidades mais prevalentes nessa população (18,97%), o diabetes não se associou a maior mortalidade.^347^

•Estudo transversal de Andrade CM *et al.* , 2022, a partir de análises de notificação de síndrome respiratória aguda grave por COVID-19 (DATASUS) de 2020 a 2021, comparou casos de pessoas hospitalizadas com (111.046) e sem (273.759) diabetes, mostrando que aqueles com diabetes tiveram maior probabilidade de serem admitidos em unidade de terapia intensiva (43,7% *vs.* 37,3%) e de mortalidade (44,6% *vs.* 35,7%).^348^

•Foppa *et al.* publicaram coorte de 289 pacientes com diabetes tipo 1, 40 ± 12 anos, 49,5% mulheres, avaliados em 2019 e 2020 quanto ao cuidado recebido. O número de consultas com time multiprofissional foi menor durante a pandemia, mesmo considerando consultas presenciais e teleconsultas (4,0; 3,0-5,0 *vs.* 2,0; 1,0-3,0), assim como o percentual de pacientes com HbA1c no alvo (26% *vs.* 1%) e de pacientes que realizaram rastreamento para retinopatia diabética (63,9% *vs.* 30,9%).^349^

•Um estudo de coorte de pacientes com COVID-19 hospitalizados entre março e setembro de 2020 em 25 hospitais brasileiros buscou avaliar fatores de risco para mortalidade intra-hospitalar. De 2.054 pacientes, 52,6% eram homens e a mortalidade intra-hospitalar foi de 22,0%, enquanto a mortalidade entre aqueles que precisaram de internação em unidade de terapia intensiva foi de 47,6% e entre aqueles com diabetes foi de 29,2%. No entanto, na análise multivariada, o diabetes não foi associado a maior mortalidade.^11^

•Dados do VIGITEL mostram que, entre 2019 e 2022, apesar da redução da prevalência de consumo de refrigerantes (15,1% IC 95% 14,3-15,9% em 2019; 14,0% IC 95% 12,9%-15,3% em 2021-2022), houve aumento do sedentarismo (62,7% IC 95% 61,8-63,6% em 2019; 66,0% IC 95% 64,6%-67,4% em 2021-2022) e redução da atividade física no tempo livre (39,0% IC 95% 38,1-39,9% em 2019; 36,7% IC 95% 35,3%-38,2% em 2021-2022). Isso pode ter influenciado o aumento da prevalência de obesidade e diabetes autorreferido (7,5% IC 95% 7,0-7,9% em 2019 e 9,1%, IC 95% 8,5-9,8% em 2021) no período.^252^

## Perspectivas

•Estudos de incidência de diabetes tipo 1 e tipo 2 com representatividade nacional, buscando determinantes sociais e comportamentais, são necessários.

•Considerando a abrangência do SUS e a possibilidade de atingir cobertura de boa parte de pacientes com diabetes tipo 1 e 2, estudos focados na avaliação da efetividade de cuidado a esses pacientes no Brasil são desejáveis.

•Considerando as várias publicações que mostram aumento de sobrepeso e obesidade na população brasileira de todas as faixas etárias, principalmente para classes sociais mais baixas, políticas públicas eficientes na prevenção da obesidade deveriam ser prioritárias na busca de redução de novos casos de diabetes e suas complicações. Nelas podem ser incluídas: 1. Taxação de alimentos com alto teor calórico; 2. Rotulagem obrigatória de produtos alimentícios; 3. Criação de programas de prevenção e tratamento da obesidade nas comunidades, resgatando pessoas predispostas ao diabetes através de ferramentas simples (questionários); 4. Capacitação de equipes multiprofissionais para que possam se envolver em programas de MEV para prevenção e tratamento do diabetes; 5. Integração de profissionais de educação física aos programas mencionados.

•Inteligência artificial é ferramenta que vem sendo utilizada em diversos aspectos do diabetes: identificação de grupos de pacientes com risco distinto de complicações crônicas a partir da avaliação do comportamento da variação da HbA1c; identificação de variáveis clínicas que predizem a resposta da HbA1c em curto e longo prazo após o início do tratamento; identificação do tratamento medicamentoso instituído conforme o estado de comorbidades do paciente e a associação com o desenvolvimento de complicações; identificação de grupos de pacientes com diabetes com diferentes graus de progressão da doença; e equações preditivas para internação hospitalar. Essas novas estratificações podem ajudar a direcionar o tratamento, representando assim um primeiro passo para a medicina de precisão no diabetes. Se bem aplicada, pode trazer importantes informações que auxiliem na prevenção e manejo da doença.

•Desigualdades no acesso e na aplicação de medidas preventivas, diagnósticas e terapêuticas são claras em alguns estudos dirigidos para a população com diabetes no Brasil, relacionadas a questões sociais, demográficas, econômicas, de gênero e cor. Dados existentes devem ser compilados e os faltantes ativamente buscados para que políticas públicas com vistas a diminuir essas diferenças possam ser construídas.

## CAPÍTULO 10 – TABAGISMO E USO DE TABACO

### Tabagismo e suas consequências para as doenças cardiovasculares, Brasil e Unidades Federativas, 1990 a 2022

#### CID-10: Z.72.0

**Ver Tabelas 10-1 a 10-6 e Figuras 10-1 a 10-2**

**Table:** Abreviaturas Usadas no Capítulo 10

AVC	Acidente Vascular Cerebral
COVITEL	Inquérito Telefônico de Fatores de Risco para Doenças Crônicas não Transmissíveis em Tempos de Pandemia
DCV	Doenças Cardiovasculares
GBD	Global Burden of Disease
IC	Intervalo de Confiança
MSG	Minorias Sexuais e de Gênero
OMS	Organização Mundial da Saúde
PeNSE	Pesquisa Nacional de Saúde do Escolar
PIB	Produto Interno Bruto
PNS	Pesquisa Nacional de Saúde
UF	Unidade Federativa
VIGITEL	Vigilância de Fatores de Risco e Proteção para Doenças Crônicas por Inquérito Telefônico

## Introdução

•O uso de tabaco é uma das principais causas de mortes evitáveis no Brasil,^3^ sendo o tabagismo um importante fator de risco para DCV.^350^ O impacto negativo do tabaco na saúde resulta tanto do consumo direto de várias formas de produtos derivados do tabaco (fumado, inalado ou mascado) quanto da exposição ao tabagismo passivo.^351^ Recentemente, houve algumas mudanças nos hábitos de consumo de tabaco: a despeito da diminuição no consumo de cigarros, outras formas de consumo de tabaco estão cada vez mais comuns, como cigarros eletrônicos e narguilé.^352^ Além disso, houve impacto da pandemia de COVID-19 nos hábitos relacionados ao tabaco e seu consumo. Esses dois aspectos mais recentes foram incluídos e/ou atualizados.

•Neste capítulo, o consumo de tabaco e suas consequências para as DCV são descritos. A prevalência de tabagismo será apresentada de acordo com as principais pesquisas populacionais no Brasil, tais como: estudos atualizados da PNS 2019 contendo estimativas para a população com idade a partir de 18 anos;^4^ PeNSE 2019 contendo estimativas para adolescentes com idade de 13–17 anos;^353^ dados do VIGITEL 2021, uma pesquisa transversal de base populacional conduzida pelo Ministério da Saúde do Brasil nas capitais dos estados brasileiros, comparados com sua série temporal; e o estudo COVITEL 2022, uma pesquisa nacional transversal por inquérito telefônico realizada no primeiro trimestre de 2022 no Brasil.^252 , 352^ Essas são as pesquisas em âmbito nacional mais recentes para o Brasil. As definições aqui usadas e a relação do tabaco com DCV estão detalhadas na versão anterior deste documento (Estatística Cardiovascular – Brasil 2021).^2 , 4^

•Os principais indicadores de tabagismo entre adultos (uso de tabaco, tabagismo passivo e cigarros eletrônicos) e adolescentes (experimentação de tabaco, uso no último mês, uso de outros produtos derivados do tabaco) serão apresentados e atualizados. As taxas de mortalidade e o número absoluto de mortes atribuíveis ao tabaco no Brasil e suas 27 UF de 1990 a 2019 estão detalhados na versão anterior deste documento (Estatística Cardiovascular – Brasil 2021),^2 , 4^ pois as estimativas mais recentes do GBD ainda não haviam sido disponibilizadas até a finalização deste documento.

## Prevalência do uso de tabaco entre adolescentes

•De acordo com a PeNSE 2019, entre adolescentes do nono ano da educação fundamental e com idade de 13-17 anos, em 2019, a prevalência de tabagismo atual ou ter fumado nos 30 dias que precederam a pesquisa foi 6,8% (IC 95%, 6,3-7,3%), sendo maior no sexo masculino, 7,1% (IC 95%, 6,6-7,6%), do que no feminino, 6,5% (IC 95%, 5,8-7,2%) **( Tabela 10-1 )** .^353^

**Tabela 10-1:** – Porcentagem de estudantes com idade de 13-17 anos que fumaram nos 30 dias que antecederam a pesquisa, por sexo e tipo de escola, no Brasil, suas principais regiões e unidades federativas

Principais regiões e unidades federativas	Total			Sexo						Tipo de escola					
				Masculino			Feminino			Pública			Privada		
	Total	IC 95%		Total	IC 95%		Total	IC 95%		Total	IC 95%		Total	IC 95%	
		Limite inferior	Limite superior		Limite inferior	Limite superior		Limite inferior	Limite superior		Limite inferior	Limite superior		Limite inferior	Limite superior
Brasil	6,8	6,3	7,3	7,1	6,6	7,6	6,5	5,8	7,2	7,2	6,6	7,8	4,4	4,1	4,8
Norte	7,2	6,2	8,1	8,3	7,0	9,6	6,2	5,1	7,2	7,4	6,5	8,4	3,4	2,7	4,0
Rondônia	6,1	5,1	7,2	6,2	5,1	7,4	6,0	4,4	7,7	6,4	5,2	7,5	2,7	1,9	3,4
Acre	10,9	9,0	12,8	13,3	10,3	16,2	8,6	6,8	10,3	11,2	9,2	13,2	3,5	1,6	5,5
Amazonas	7,7	6,1	9,3	8,9	6,6	11,3	6,5	4,8	8,3	8,0	6,3	9,6	3,1	1,3	4,9
Roraima	9,2	7,8	10,6	11,2	9,3	13,0	7,1	5,5	8,7	9,6	8,1	11,1	2,4	1,0	3,7
Pará	6,6	4,9	8,4	7,9	5,2	10,5	5,6	3,7	7,6	6,9	5,1	8,8	3,3	2,3	4,2
Amapá	7,1	5,9	8,3	6,9	5,7	8,2	7,3	5,6	9,0	7,4	6,1	8,7	4,2	3,3	5,1
Tocantins	6,8	5,1	8,5	7,7	5,1	10,3	6,0	4,2	7,7	6,9	5,1	8,8	5,2	3,9	6,5
Nordeste	4,7	4,3	5,2	5,3	4,7	5,8	4,2	3,7	4,8	5,0	4,5	5,5	2,7	2,4	3,0
Maranhão	6,0	4,7	7,4	7,5	5,7	9,2	4,5	2,6	6,4	6,3	4,8	7,7	2,9	2,3	3,6
Piauí	4,3	3,2	5,3	5,1	3,7	6,5	3,5	2,2	4,7	4,5	3,3	5,7	2,6	1,8	3,4
Ceará	5,7	4,3	7,1	6,0	4,0	8,1	5,4	4,1	6,7	6,2	4,5	7,9	2,5	1,9	3,2
Rio Grande do Norte	4,5	3,5	5,5	4,7	3,4	6,1	4,3	3,1	5,5	5,0	3,9	6,2	1,9	1,3	2,4
Paraíba	5,9	4,9	6,8	6,5	5,0	8,0	5,3	4,0	6,5	6,4	5,3	7,5	3,3	2,4	4,3
Pernambuco	4,4	3,5	5,4	4,3	2,9	5,7	4,5	3,1	5,9	4,6	3,5	5,7	3,4	2,6	4,2
Alagoas	5,0	3,8	6,2	6,4	4,4	8,4	3,5	2,4	4,7	5,4	4,0	6,9	2,4	1,7	3,2
Sergipe	3,2	2,4	4,1	3,6	2,5	4,8	2,9	1,8	4,0	3,5	2,4	4,5	2,3	1,7	2,9
Bahia	3,7	2,6	4,7	3,9	2,8	5,0	3,4	2,1	4,8	3,8	2,6	5,0	2,4	1,7	3,1
Sudeste	7,6	6,4	8,7	7,6	6,5	8,6	7,6	5,8	9,4	8,2	6,8	9,6	5,0	4,3	5,7
Minas Gerais	8,2	6,7	9,6	8,3	6,5	10,1	8,0	5,9	10,2	8,4	6,8	10,0	6,7	5,2	8,2
Espírito Santo	6,8	5,5	8,0	8,2	6,3	10,0	5,4	4,1	6,8	7,0	5,6	8,4	4,9	3,8	6,0
Rio de Janeiro	6,6	5,5	7,7	7,0	5,6	8,4	6,2	4,9	7,6	7,6	6,1	9,1	3,9	3,1	4,7
São Paulo	7,7	5,7	9,8	7,3	5,7	9,0	8,1	4,9	11,3	8,4	5,8	10,9	5,0	3,8	6,1
Sul	8,0	7,1	8,9	7,8	6,6	9,0	8,3	6,9	9,6	8,3	7,3	9,4	5,9	5,0	6,9
Paraná	8,9	7,3	10,6	8,2	6,0	10,3	9,8	7,6	11,9	9,4	7,5	11,3	6,3	4,8	7,7
Santa Catarina	8,5	6,8	10,2	9,0	6,7	11,3	8,1	5,9	10,2	9,0	7,1	10,9	5,1	3,3	6,8
Rio Grande do Sul	6,8	5,4	8,1	6,6	5,0	8,1	7,0	4,5	9,4	6,8	5,3	8,4	6,2	4,6	7,9
Centro-Oeste	7,7	6,9	8,5	8,6	7,6	9,7	6,8	6,0	7,7	8,1	7,2	9,0	5,3	4,7	6,0
Mato Grosso do Sul	9,7	8,5	10,9	10,3	8,6	12,1	9,1	7,8	10,3	10,1	8,8	11,5	6,2	4,8	7,6
Mato Grosso	8,4	6,7	10,1	9,8	7,5	12,1	7,0	4,8	9,2	8,7	6,8	10,7	5,0	3,7	6,2
Goiás	7,4	6,0	8,7	8,4	6,6	10,2	6,3	5,2	7,5	7,6	6,1	9,2	6,1	4,9	7,2
Distrito Federal	6,0	4,5	7,4	6,1	4,4	7,9	5,8	3,7	8,0	6,5	4,7	8,4	4,2	3,1	5,3

Fonte: PeNSE 2019. ^353^

•A prevalência do primeiro uso de cigarro antes dos 13 anos de idade foi 13,1% (IC 95%, 11,7-14,5%), com menores níveis no sexo masculino, 11,7% (IC 95%, 10,2-13,1%), do que no feminino, 14,4% (IC 95%, 12,6-16,2%).^353^

•Entre adolescentes com idade de 13-17 anos, 22,6% (IC 95%, 21,7-23,4%) haviam experimentado cigarro, variando de 16,6% na região Nordeste a 28,5% na Sul.^353^

•Entre adolescentes, houve aumento de 2% na prevalência de alunos do nono ano que já haviam experimentado cigarro, que passou de 19% (IC 95%, 18,1-19,9%) em 2015 para 21% (IC 95%, 19,5%-22,5%) em 2019.^353^

•Em 2019, a prevalência do uso de cigarro eletrônico em algum momento por adolescentes de 13-17 anos foi 16,8% (IC 95%, 16,2-17,4%), sendo maior no sexo masculino (19,1%; IC 95%, 18,3-19,9%) do que no feminino (14,6%; IC 95%, 13,9%-15,3%) **(Tabela 10-4)** .^353^

•O uso de narguilé em algum momento por adolescentes de 13-17 anos foi 29,6%, (IC 95%, 28,6-30,7%), sem diferença entre o sexo feminino (29,6%; IC 95%, 28,2-31%) e o masculino (29,7%; IC 95%, 28,6-30,8%).^353^

•A proporção de produtos derivados do tabaco usados por adolescentes de 13-17 anos nos 30 dias anteriores à pesquisa foi 7,8% (IC 95%, 7,3-8,3%) para narguilé e 2,8% (IC 95%, 2,6-3,0%) para cigarros eletrônicos.^353^

•Aumento do risco de uso regular de tabaco foi associado com consumo de álcool nos últimos 30 dias para ambos os sexos. Além disso, para indivíduos do sexo masculino, esse risco foi aumentado pelo envolvimento em brigas físicas nos 12 meses anteriores.^354^

## Prevalência do uso de tabaco entre adultos

•De acordo com o VIGITEL 2021, a prevalência de tabagismo entre adultos (≥ 18 anos) foi 9,1% (IC 95%, 8,2-9,9%), maior nos homens, 11,8% (IC 95%, 10,3-13,3%), do que nas mulheres, 6,7% (IC 95%, 5,8-7,7%). A prevalência foi menor entre adultos jovens (≤ 34 anos) e aqueles ≥ 65 anos **( Tabela 10-2** e **Figuras 10-1** e **10-2)** .^252^

**Tabela 10-2:** – Prevalência de fumantes atuais com idade ≥ 18 anos, por sexo, idade e anos de escolaridade

	Total			Sexo					
				Masculino			Feminino		
	Total	IC 95%		Total	IC 95%		Total	IC 95%	
		Limite inferior	Limite superior		Limite inferior	Limite superior		Limite inferior	Limite superior
**Total (%) **	9,1	8,2	9,9	11,8	10,3	13,3	6,7	5,8	7,7
**Idade (anos) **									
18-24	6,4	4,4	8,4	7,2	4,8	9,6	5,4	2,1	8,8
25-34	7,3	5,6	9,0	11,9	8,7	15,0	3,3	1,7	4,8
35-44	11,6	9,0	14,3	17,7	12,7	22,6	6,9	4,4	9,4
45-54	10,1	8,1	12,1	11,6	8,3	14,9	8,8	6,4	11,2
55-64	11,5	9,7	13,4	13,1	9,9	16,3	10,3	8,2	12,4
≥ 65	7,4	6,2	8,6	7,3	5,2	9,4	7,5	6,1	9,0
**Escolaridade (anos) **									
0-8	12,9	11,0	14,8	15,7	12,6	18,9	10,3	8,0	12,6
9-11	8,1	6,8	9,5	10,5	8,1	12,8	6,0	4,7	7,4
≥ 12	7,2	5,9	8,4	10,3	8,0	12,6	4,8	3,5	6,1

Fonte: VIGITEL Brasil 2021. ^252^

•A prevalência foi máxima no grupo etário 35-44 anos [11,6% (IC 95%, 9,0-14,3%)] em comparação aos seguintes grupos etários: 18-24 anos [6,4% (IC 95%, 4,4-8,4%)]; 25-34 anos [7,3% (IC 95%, 5,6-9,0%)]; e ≥ 65 anos [7,4% (IC 95% 6,2-8,6%)] **( Tabela 10-2 )** .^252^

•A prevalência foi menor nas populações com mais altos níveis educacionais, sendo 7,2% (IC 95%, 5,9-8,4%) entre aqueles com pelo menos 12 anos de escolaridade, 8,1% (IC 95%, 6,8-9,5%) entre aqueles com 9-11 anos de escolaridade e 12,9% (IC 95%, 11,0-14,8%) entre aqueles com 0-8 anos de escolaridade.^252^

•Entre as MSG, a prevalência do consumo de qualquer produto derivado do tabaco foi 44,7%. O uso de narguilés foi ∼8 vezes maior para as MSG do que para indivíduos não pertencentes a essas minorias. Os usuários de tabaco pertencentes às MSG tendem a ser mais jovens e mais escolarizados e a ter menor probabilidade de relacionamentos estáveis em comparação a usuários de tabaco não pertencentes às MSG ou usuários de tabaco em geral.^355 , 356^

•De 2013 a 2019, houve aumento de 52% na prevalência de ex-fumantes, de 17,5% (IC 95%, 16,9-18,0) para 26,6% (IC 95%, 26,1-27,2). Além disso, em 2019, 46,6% (IC 95%, 45,0-48,3) dos fumantes relataram tentativa de parar de fumar em contraste com 51,1% (IC 95%, 49,3-52,9) em 2013, o que representa uma redução de 8,8% no mesmo período.

## Tendência de prevalência

•De acordo com dados do VIGITEL 2021, houve uma redução de 43,8% na prevalência de tabagismo na população adulta (idade ≥ 18 anos) para os dois sexos de 2006 a 2021. A prevalência de tabagismo foi 16,2% em 2006, com queda progressiva para 9,1% em 2021.^252^

•De 2015 a 2019, dados do VIGITEL mostraram que o consumo de cigarros por adolescentes permaneceu estável. Em 2015, 6,6% (IC 95%, 5,8-7,3%) dos adolescentes relataram ter usado cigarros nos últimos 30 dias, enquanto, em 2019, essa prevalência foi 6,8% (IC 95%, 6,3-7,3%).^353^ O uso de outros produtos derivados do tabaco nos últimos 30 dias por adolescentes aumentou de 2015 (7,2%; IC 95%, 6,1-8,2%) para 2019 (12,4%; IC 95%, 11,8-12,9%).^353^

## Tabagismo passivo

•De acordo com dados do VIGITEL 2021, a prevalência de tabagismo passivo no domicílio foi 6,9% (IC 95%, 6,0-7,9%), maior entre os homens (7,6%; IC 95%, 6,1-9,2%) do que entre as mulheres (6,4%; IC 95%, 5,1-7,6%) **(Tabela 10-3)** . A prevalência de tabagismo passivo no domicílio nas capitais brasileiras variou de 2,9% (IC 95%, 0,9-4,9%) em Salvador a 9,6% (IC 95%, 6,8-12,3%) em Rio Branco. A maior prevalência entre os homens foi no Rio de Janeiro, 11,2% (IC 95%, 5,0-17,4%), enquanto, entre as mulheres, em Belo Horizonte, 10,8% (IC 95%, 7,0-14,6%). A prevalência de tabagismo passivo no trabalho foi 5,4% (IC 95%, 4,6-6,3%), maior entre os homens, 8,1% (IC 95%, 6,6-9,5%), do que entre as mulheres, 3,2% (IC 95%, 2,4-4,0%).

## Cigarros eletrônicos e narguilés

•Os dispositivos eletrônicos de fumar, mais conhecidos como cigarros eletrônicos, são aqueles operados por bateria que fornecem nicotina, sabores e outras substâncias químicas em aerossol ao usuário. O resultado é a geração de um aerossol finamente particulado.^357^ Os líquidos usados nesses dispositivos podem diferir quanto à sua composição química, concentração de nicotina e aditivos usados, tendo sido descritos mais de 8 mil sabores de cigarros eletrônicos. Discrepância entre a composição do produto declarada na embalagem e a verdadeira já foi mostrada.^358^

•Os cigarros eletrônicos entraram no mercado global por volta de 2006. O Brasil foi um dos primeiros países a banir o uso de dispositivos eletrônicos de fumar em 2009.^359 , 360^

•Em 2019, a prevalência de uso ativo de cigarros eletrônicos foi 0,64% (IC 95%, 0,51-0,76%) na população com idade ≥ 15 anos. Entre os adultos com idade < 40 anos, a prevalência foi estimada em 40 vezes aquela entre adultos ≥ 40 anos (0,06% *vs.* 2,38%). No grupo etário de 15-24 anos em uso atual de cigarros eletrônicos, 62,6% relataram nunca ter fumado cigarros industrializados. A prevalência do uso de cigarro eletrônico aumentou entre indivíduos com idade de 15-65 anos de 0,45% em 2015 para 0,72% em 2019 e, de forma especial, no grupo etário de 15-24 anos.^361^ No estudo COVITEL 2022, na população ≥ 18 anos, a prevalência de uso de cigarro eletrônico e narguilé em algum momento foi idêntica àquela relatada no VIGITEL (7,3%; IC 95%, 6,0-8,9), sendo a maior prevalência de experimentação observada nos adultos jovens (18-24 anos).^352^ No geral, 16,8% (IC 95%, 16,2-17,4%) dos adolescentes de 13-17 anos relataram uso de cigarro eletrônico em algum momento, sendo as taxas mais altas entre os homens (19,1%; IC 95%, 18,3-19,9%) do que entre as mulheres (14,6%; IC 95%, 13,9-15,3%).^353^

•A prevalência do uso ativo de narguilé em 2019 foi 0,47% (IC 95%, 0,36-0,59%) na população com idade ≥ 15 anos. Essa prevalência aumentou de 0,14% em 2013 para 0,43% em 2019 entre indivíduos com idade ≥ 18 anos. No grupo etário de 18-24 anos, o aumento foi de cerca de 300% no mesmo período.^362^

## Impacto Econômico do Uso do Tabaco

•Usando um modelo econômico (micro simulação probabilística de Markov) que considera a história natural e os custos médicos diretos e indiretos, a carga econômica total atribuível ao tabaco no Brasil em 2020 foi estimada em US$ 24,3 bilhões, representando 1,9% do PIB e 7,8% das despesas nacionais em saúde.^354^O custo médico direto representa 38,5% da carga econômica, enquanto 24,8% são representados pelo custo do cuidador e 36,7% pela perda de produtividade.^354^A DCV representou 24% dos custos médicos diretos (US$ 2,3 bilhões) atribuíveis ao tabaco, enquanto o AVC representou 4,8% (US$ 447 milhões) **( Tabela 10-5 )** .^354^

**Tabela 10-5:** – Carga econômica anual atribuível a tabaco de acordo com a causa, Brasil, 2020 (US$ milhões)

Carga econômica (US$ milhões)	Dados brasileiros para 2020
Custo médico direto (%)	9.347,4 (38,5)
Custo com cuidador (%	6.023,7 (24,8)
Custo de produtividade (%)	6.023,7 (24,8)
Carga econômica total	24.301,3 (100%
Como proporção do PIB	1,9%
Proporção recuperada através dos impostos	9,4%
**Custo médico direto (US$ milhões)**	
DCV	2.280,4 (24,4%)
AVC	447,0 (4,8%
DPOC	4.307,8 (46,1%)
Pneumonia	31,2 (0,3%)
Câncer de pulmão	453,2 (4,8%)
Outros cânceres	751,8 (8,0%)
Tabagismo passivo	1.076,0 (11,5%)
Custo médico direto total	9.347,4 (100%)
Proporção das despesas nacionais de saúde	7,8%
**Custo de produtividade (US$ milhões)**	
**Morte prematura**	
DCV	917,7 (26,4%)
AVC	324,8 (9,4%)
DPOC	469,8 (13,5%)
Pneumonia	161,7 (4,7%)
Câncer de pulmão	452,2 (13,0%)
Outros cânceres	739,8 (21,3%)
Tabagismo passivo	405,9 (11,7%)
Total de mortes prematuras	3.471,8 (100%)
**Incapacidade **	
DCV	1.368,4 (25,1%)
AVC	375,0 (6,9%)
DPOC	1.544,7 (28,3%)
Pneumonia	00,9 (0,0%)
Câncer de pulmão	547,4 (10,0%)
Outros cânceres	988,3 (18,1%
Tabagismo passivo	633,7 (11,6%)
Incapacidade total	5.458,4 (100%)
Morte Prematura	3.471,8 (38,9%)
Incapacidade	5.458,4 (61,1%)
Total de custo de produtividade	8.930,2 (100%)
**Custos com cuidadores (US$ milhões)**	
DCV	2.160,5 (35,9%)
AVC	530,0 (8,8%)
DPOC	1.918,1 (31,8%)
Pneumonia	39,0 (0,6%)
Câncer de pulmão	224,3 (3,7%)
Outros cânceres	457,9 (7,6%)
Tabagismo passivo	693,8 (11,5%)
Total de custo com cuidadores	6.023,7 (100%)

PIB: produto interno bruto; DCV: doença cardiovascular; AVC: acidente vascular cerebral; DPOC: doença pulmonar obstrutiva crônica. Fonte: Adaptado de Pichon-Riviere et al. ^354^

Impacto da COVID-19 no consumo de tabaco

•Durante a pandemia de COVID-19, a prevalência de uso de tabaco por adolescentes permaneceu estável (2,41%; IC 95%, 2,02-2,87). Os fatores associados com maior probabilidade de consumo de tabaco durante a pandemia de COVID-19 foram os seguintes: idade entre 16 anos e 17 anos; autoidentificação como negro; residência nas regiões Sul e Sudeste; relato de sensação de depressão e solidão; piora de distúrbios do sono; consumo de álcool; tabagismo passivo.^362^

•No estudo COVITEL 2022, entre a população com idade ≥ 18 anos, a prevalência de tabagismo permaneceu estável: 14,7% (IC 95%, 13,0-16,7%) antes da pandemia de COVID-19 e 12,2% (IC 95%, 10,4-14,1%) no primeiro trimestre de 2022 **( Tabela 10-6 )** .^363^

**Tabela 10-6:** – Prevalência de tabagismo antes e depois da pandemia de COVID-19, de acordo com sexo, principais regiões brasileiras, grupo etários e anos de escolaridade

	Antes da pandemia de COVID-19			Primeiro trimestre 2022		
	Total	IC 95%		Total	IC 95%	
		Limite inferior	Limite superior		Limite inferior	Limite superior
**Total**	14,7	13,0	16,7	12,2	10,4	14,1
**Sexo**						
Masculino	18,0	15,5	20,7	14,5	12,2	17,2
Feminino	11,7	9,9	13,9	9,9	8,1	12,2
**Principais regiões**						
Nordeste	10,1	7,2	13,9	7,9	5,8	10,7
Norte	12,1	10,5	13,9	8,0	6,1	10,4
Sul	18,9	15,0	23,5	15,5	11,9	19,9
Sudeste	16,3	14,3	18,5	14,3	12,0	16,8
Centro-Oeste	16,7	15,1	18,4	12,6	10,3	15,2
**Grupo etários (anos)**						
18 a 24	13,9	9,9	19,3	12,1	8,4	17,1
25 a 34	17,0	12,7	22,3	14,5	9,9	20,9
35 a 44	13,9	11,0	17,3	11,1	8,7	14,2
45 a 54	13,8	10,9	17,5	12,4	9,3	16,2
55 a 64	17,4	13,8	21,6	13,6	9,6	18,9
≥ 65	12,1	9,3	15,6	8,0	5,8	10,9
**Raça**						
Branca	13,2	11,2	15,4	10,8	9,0	13,1
Negra	15,0	12,7	17,5	12,4	10,2	15,0
Outras	21,7	15,4	29,6	17,7	11,8	25,7
**Escolaridade (anos)**						
0 a 8	17,6	15,2	20,4	14,7	12,4	17,3
9 a 11	14,1	11,5	17,2	11,6	9,0	14,7
≥ 12	8,3	6,8	10,1	6,5	5,4	7,8

Fonte: COVITEL 2022. ^352^

•De acordo com uma pesquisa *on-line* , a prevalência de fumantes durante a pandemia de COVID-19 foi 12% (95%CI, 11,1-12,9%) e 34% deles relataram aumento no consumo de cigarro. Esse aumento foi associado a pior qualidade do sono, sensação de isolamento dos familiares, de tristeza ou de ansiedade, problemas financeiros e saúde deficiente segundo autoavaliação.^364 , 365^

## Políticas públicas para controle do tabaco no Brasil

•O processo de controle do tabaco no Brasil começou em 1981 com a criação da Comissão para Estudos das Consequências do Fumo pelo Ministério da Saúde. Em 2006, o Brasil ratificou a Convenção-Quadro para o Controle do Tabaco da OMS. O país baniu o tabagismo em espaços fechados exceto em locais designados (cabines para fumantes) em 1996 e, em 2011, baniu o tabagismo completamente de todos os locais públicos e de trabalho, além dos transportes públicos. A reforma tributária de 2011 do Imposto sobre Produtos Industrializados contribuiu substancialmente para reduzir o tabagismo em meios às inúmeras leis antitabagismo e regulamentações postas em uso no Brasil nas últimas décadas. Essa reforma deu ao governo autoridade para estabelecer um preço mínimo pelo pacote de cigarros, que seria aumentado anualmente com base nas taxas de inflação estimadas.^366^ O aumento do imposto que elevou o preço do cigarro refletiu-se em redução das despesas médicas com doenças relacionadas ao tabagismo. Cada elevação de 10% no preço por aumento dos impostos referentes ao tabaco reduz seu consumo em 5%.^367^Por causa de todas essas medidas, houve um claro declínio no consumo de tabaco entre adultos desde 1989.^368^ A prevalência de tabagismo caiu drasticamente 63,2% nas últimas três décadas.^256^

•Em consequência, a carga de mortalidade atribuível ao tabagismo caiu 70,1% de 1990 a 2017, sendo a maior queda observada para AVC (-75,3%).^256^

•Com base nas estatísticas de 2019, a proporção de contrabando de cigarros no Brasil foi projetada em 38,6% (IC 95%, 35,8- 41,5%). Um quarto das marcas ilícitas de cigarros foi vendido pelo preço mínimo legal ou acima dele.^366^

## Perspectiva

•A redução progressiva na prevalência do tabagismo no Brasil pode ser atribuída à adoção no país de várias medidas de controle do fumo, como proibição de venda de tabaco para menores, inclusão de mensagens de advertência nas embalagens de cigarro, proibição de propaganda, promoção e patrocínio do fumo, elevação dos impostos sobre tabaco, criação de ambientes sem fumo e outras recomendações da Convenção-Quadro para o Controle do Tabaco.^369^ A política de progressiva elevação do imposto sobre o tabaco reduziria o consumo de cigarros e os custos médicos a ele associados. Consequentemente, vidas mais saudáveis e mais produtivas resultariam em mais renda para todos.^369^ Os formuladores de políticas precisam promover continuamente a implementação de uma estrutura para eliminar o comércio ilegal de derivados do tabaco, focando em particular nas populações com maior prevalência de consumo, como as MSG.^370^

•Há crescente preocupação com os novos caminhos que levam à adição à nicotina por meio de cigarros eletrônicos e narguilé. A indústria do tabaco visa principalmente os jovens, sendo “ *vaping* ” divulgado como seguro e inofensivo. Pesquisas futuras devem focar nos riscos à saúde associados ao consumo desses produtos, além do desenvolvimento de estratégias que visem ao consumo crescente de populações específicas, como adolescentes e adultos jovens. Ademais, a prevalência do uso de outros tipos de cigarros eletrônicos e narguilé, o tipo de dispositivo e a frequência de uso precisam ser mais bem estimadas.^352^

## CAPÍTULO 11 – OBESIDADE E SOBREPESO

### CID-10 E66

#### Ver Tabelas 11-1 a 11-4 e Figuras 11-1 a 11-4

**Table:** Abreviaturas Usada no Capítulo 11

CID	Classificação Estatística Internacional de Doenças e Problemas Relacionados à Saúde
DALYs	Anos de vida perdidos ajustados por incapacidade (do inglês, * Disability-Adjusted Life-Year* )
DCV	Doença Cardiovascular
ELSA-Brasil	Estudo Longitudinal de Saúde do Adulto
HR	Hazard Ratio
IC	Intervalo de Confiança
IMC	Índice de Massa Corporal
OMS	Organização Mundial da Saúde
OR	Odds Ratio
PNS	Pesquisa Nacional de Saúde
SIA/SUS	Sistema de Informações Ambulatoriais do SUS
SIH/SUS	Sistema de Informações Hospitalares do SUS
SUS	Sistema Único de Saúde
UF	Unidade Federativa
UTI	Unidade de Terapia Intensiva
VIGITEL	Vigilância de Fatores de Risco e Proteção para Doenças Crônicas por Inquérito Telefônico

## Panorama

•A obesidade é um fator de risco para DCV, além de doença crônica complexa. De acordo com a OMS, obesidade é definida como acúmulo anormal ou excessivo de gordura que representa um risco para a saúde. Um IMC ≥ 30 kg/m^2^ é considerado obesidade, enquanto um IMC maior que ou igual a 25 kg/m^2^é considerado excesso de peso ou sobrepeso.^371^ Vale notar que a obesidade é uma doença crônica que resulta de múltiplos fatores principalmente os relacionados a estilo de vida (sedentarismo, hábitos alimentares não saudáveis) e outros, como genéticos, hereditários, psicológicos, culturais e étnicos.

•Obesidade permanece uma ameaça à saúde pública. Gaspar *et al* .^372^ investigaram a associação de hiperglicemia, obesidade, dislipidemia e tabagismo no Brasil de 2005 a 2017, além das taxas de mortalidade e incidência das DCV, doença arterial coronariana e acidente vascular cerebral isquêmico, por 100 mil indivíduos. A exposição a fatores de risco foi calculada como uma medida da população exposta a um fator de risco que considera a extensão da exposição de acordo com o nível do risco e a intensidade de sua contribuição para a carga de doença. A mortalidade e a incidência de DCV padronizadas por idade diminuíram 21% e 8%, respectivamente, de 2005 a 2017. Entretanto, houve aumento naquela medida de 9,5% para hiperglicemia, de 31% para obesidade e de 5,2% para dislipidemia, enquanto que, para hipertensão, aquela medida manteve-se estável e, para tabagismo, diminuiu 33%. Isso ressalta a importância da obesidade em relação às DCV e a necessidade urgente de políticas para reduzir a obesidade na população brasileira, em um esforço para mitigar a carga da mortalidade por DCV.

## Prevalência

•No Brasil, em 2019, de acordo com dados antropométricos da PNS, as porcentagens de adultos (idade ≥18 anos) com excesso de peso e obesidade foram, respectivamente, 57,5% (IC 95%, 54,8 – 60,2) e 21,8 % (IC 95%, 19,2 – 24,7) para homens e 62,6% (IC 95%, 59,1 – 66,0) e 29,5% (IC 95%, 25,4 – 34,0) para mulheres.^373^

•A **Tabela 11-1** mostra a prevalência de excesso de peso entre indivíduos com 18 anos ou mais, ambos os sexos e todas as idades, nas capitais brasileiras, em 2021, de acordo com os grupos etários do VIGITEL 2021. Nas capitais brasileiras, a porcentagem de adultos (idade ≥18 anos) com excesso de peso em 2021 foi 57,2% (IC 95%, 55,7-58,8) no geral, sendo 59,9% (IC 95%, 57,6-62,2) para os homens e 55,0% (IC 95%, 53,0-57,0) para as mulheres. Aumento progressivo de excesso de peso foi observado com o aumento da idade, variando de 35,7% (IC 95%, 31,5-40,0) [homens: 39,3% (IC 95%, 33,6-45,1); mulher: 31,7% (IC 95%, 25,5-37,9)] no grupo etário de 18-24 anos a 64,4% (IC 95%, 61,5-67,2) [homens: 67,2% (IC 95%, 62,6-71,9); mulheres: 61,9% (IC 95%, 58,5-65,3)] no grupo etário de 45-54 anos. Para o grupo etário de 60+ anos, houve discreta redução na prevalência de excesso de peso, 60,7% (IC 95%, 58,8-62,6) [homens: 60,7% (IC 95%, 57,2-64,2); mulheres: 60,7% (IC 95%, 58,6-62,9)].

**Tabela 11-1:** – Porcentagem de adultos com sobrepeso (≥ 18 anos), por sexo, nas capitais brasileiras e no Distrito Federal, por grupo etário

Grupo etário	Homens		Mulheres		Total	
	%	IC 95%	%	IC 95%	%	IC 95%
18 - 24 anos	39,3	33,6 - 45,1	31,7	25,5 - 37,9	35,7	31,5 - 40,0
25 - 34 anos	63,4	57,7 - 69,1	46,6	41,1 - 52,0	54,4	50,3 - 58,5
35 - 44 anos	62,9	57,3 - 68,6	61,9	57,7 - 66,2	62,4	59,0 - 65,8
45 - 54 anos	67,2	62,6 - 71,9	61,9	58,5 - 65,3	64,4	61,5 - 67,2
55 - 64 anos	61,9	57,2 - 66,7	65,7	62,7 - 68,6	64,1	61,4 - 66,7
60 + anos	60,7	57,2 - 64,2	60,7	58,6 - 62,9	60,7	58,8 - 62,6

Fonte: VIGITEL Brazil 2021. ^252^

•A **Tabela 11-2** mostra a prevalência de obesidade entre indivíduos com idade de 18 anos ou mais, ambos os sexos e todas as idades, nas capitais brasileiras, em 2021, de acordo com os grupos etários do VIGITEL 2021. Nas capitais brasileiras, a porcentagem de adultos (idade ≥18 anos) com obesidade em 2021 foi 22,4% (IC 95%, 21,1-23,6) no geral, sendo 22,0% (IC 95%, 20,0-24,0) para homens e 22,6 (IC 95% 21,1-24,2) para mulheres. Aumento progressivo de obesidade foi observado com o aumento da idade, variando de 12,2% (IC 95%, 9,2-15,3%) [homens: 13,1% (IC 95%, 9,3-17,0); mulheres: 11,2% (IC 95%, 6,4-16,0)] no grupo etário de 18-24 anos a 26,2% (IC 95%, 23,7-28,8) [homens: 24,3% (IC 95%, 20,3-28,3); mulheres: 27,9% (IC 95%, 24,7-31,2)] no grupo etário de 45-54 anos. No grupo etário de 60+ anos, houve discreta redução na prevalência de obesidade, 21,8% (IC 95%, 20,2-23,4) [homens: 16,8% (IC 95%, 14,1-19,5); mulheres: 25,3% (IC 95%, 23,4-27,2)].

**Tabela 11-2:** – Porcentagem de adultos com obesidade (≥ 18 anos), por sexo, nas capitais brasileiras e no Distrito Federal, por grupo etário.

Grupo etário	Homens		Mulheres		Total	
	%	IC 95%	%	IC 95%	%	IC 95%
18 - 24 anos	13,1	9,3 - 17,0	11,2	6,4 - 16,0	12,2	9,2 - 15,3
25 - 34 anos	25,5	19,9 - 31,1	16,6	12,8 - 20,4	20,8	17,4 - 24,1
35 - 44 anos	25,1	20,7 - 29,6	25,7	21,9 - 29,5	25,5	22,5 - 28,4
45 - 54 anos	24,3	20,3 - 28,3	27,9	24,7 - 31,2	26,2	23,7 - 28,8
55 - 64 anos	22,1	18,3 - 26,0	29,3	26,2 - 32,5	26,2	23,8 - 28,7
60 + anos	16,8	14,1 - 19,5	25,3	23,4 - 27,2	21,8	20,2 - 23,4

Fonte: VIGITEL Brasil 2021. ^252^

•A **Tabela 11-3** mostra a porcentagem de sobrepeso em adultos, por sexo, nas capitais brasileiras e no Distrito Federal, de acordo com dados do VIGITEL 2021. A maioria das capitais mostrou porcentagens mais altas do que os valores nacionais para ambos os sexos, exceto Palmas, Salvador, São Luís, Teresina e Vitória. O mesmo ocorreu para homens nas capitais Belo Horizonte, Boa Vista, Cuiabá, Florianópolis, Maceió, Natal, Palmas, Recife, Salvador, São Luís, São Paulo, Vitória e Distrito Federal, que mostraram porcentagens mais baixas do que os valores nacionais. Vale notar que, para mulheres com excesso de peso, as capitais com valores abaixo da média nacional foram: Palmas, Salvador, São Luís, Teresina e Vitória. A **Figura 11-1** mostra a porcentagem de adultos com sobrepeso nas capitais brasileiras e no Distrito Federal de acordo com dados do VIGITEL 2021, estratificados por quartis. As capitais dos seguintes estados ocuparam o mais alto quartil de sobrepeso: Acre, Amazonas, Amapá, Pará e Rondônia.

**Tabela 11-3:** – Porcentagem de adultos com sobrepeso (≥ 18 anos), por sexo, de acordo com as capitais brasileiras e o Distrito Federal

Capitais	Homens		Mulheres		Total	
	%	IC 95%	%	IC 95%	%	IC 95%
Aracaju	60,9	52,0 - 69,9	54,4	48,8 - 60,1	57,3	52,3 - 62,4
Belém	61,6	54,3 - 68,9	61,0	55,6 - 66,4	61,3	56,8 - 65,7
Belo Horizonte	58,4	51,1 - 65,7	58,7	53,3 - 64,1	58,6	54,1 - 63,0
Boa Vista	58,7	51,9 - 65,5	54,2	49,3 - 59,2	56,4	52,2 - 60,5
Campo Grande	61,3	51,8 - 70,7	55,4	48,6 - 62,1	58,1	52,4 - 63,8
Cuiabá	58,5	50,2 - 66,8	55,7	49,2 - 62,2	57,0	51,8 - 62,3
Curitiba	62,8	55,7 - 69,9	48,9	42,7 - 55,0	55,3	50,6 - 60,1
Florianópolis	59,5	51,6 - 67,4	54,5	48,3 - 60,8	56,9	51,9 - 61,9
Fortaleza	61,2	53,4 - 68,9	57,5	51,5 - 63,6	59,2	54,4 - 64,0
Goiânia	62,1	54,4 - 69,9	51,2	45,2 - 57,2	56,3	51,4 - 61,2
João Pessoa	66,5	58,6 - 74,3	53,9	47,6 - 60,2	59,6	54,6 - 64,6
Macapá	62,3	55,9 - 68,7	55,5	50,1 - 60,9	58,7	54,6 - 62,9
Maceió	59,2	51,3 - 67,2	57,2	50,4 - 64,0	58,1	53,0 - 63,2
Manaus	65,2	57,3 - 73,1	61,8	56,3 - 67,4	63,5	58,7 - 68,2
Natal	60,9	52,7 - 69,1	57,5	51,7 - 63,3	59,1	54,2 - 64,0
Palmas	55,9	47,8 - 64,0	45,0	39,4 - 50,6	50,1	45,2 - 55,0
Porto Alegre	64,4	57,0 - 71,8	60,4	52,9 - 67,8	62,2	56,9 - 67,4
Porto Velho	67,5	60,3 - 74,7	61,0	55,5 - 66,6	64,4	59,8 - 69,0
Recife	58,1	49,1 - 67,1	55,6	49,5 - 61,7	56,7	51,5 - 61,9
Rio Branco	63,2	55,6 - 70,8	57,8	52,0 - 63,6	60,4	55,7 - 65,0
Rio de Janeiro	64,0	56,3 - 71,7	49,4	43,1 - 55,8	56,1	51,0 - 61,2
Salvador	50,8	43,0 - 58,7	55,1	49,5 - 60,8	53,2	48,5 - 57,9
São Luís	51,4	43,2 - 59,7	47,5	41,8 - 53,2	49,3	44,4 - 54,2
São Paulo	57,6	50,7 - 64,5	57,3	51,0 - 63,5	57,4	52,8 - 62,0
Teresina	60,0	52,1 - 67,9	46,4	40,4 - 52,3	52,5	47,5 - 57,5
Vitória	55,8	48,1 - 63,6	47,8	41,9 - 53,7	51,5	46,7 - 56,3
Distrito Federal	59,4	51,7 - 67,2	53,7	47,5 - 59,8	56,4	51,5 - 61,2

Fonte: VIGITEL Brasil 2021. ^252^

**Figura 11-1:**
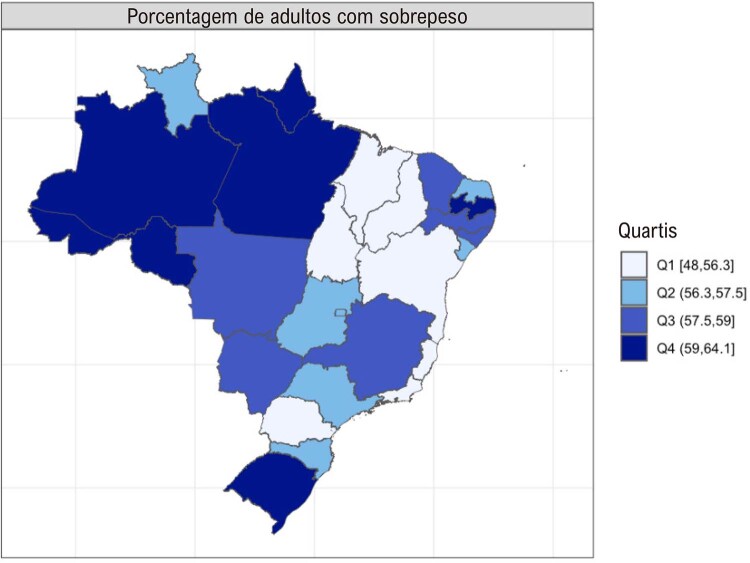
– Porcentagem de adultos com sobrepeso nas capitais brasileiras e no Distrito Federal, de acordo com dados do VIGITEL 2021, estratificados por quartis. Dados do VIGITEL Brasil 2021. ^252^

•A **Tabela 11-4** mostra a porcentagem de adultos obesos, por sexo, nas capitais brasileiras e no Distrito Federal, de acordo com dados do VIGITEL 2021. A maioria das capitais mostrou porcentagens mais altas do que os valores nacionais para ambos os sexos, exceto Belo Horizonte, Campo Grande, Florianópolis, Palmas, Rio de Janeiro, Salvador, São Luís, Teresina e Vitória. O mesmo ocorreu para homens nas capitais Belo Horizonte, Campo Grande, Florianópolis, Maceió, Recife, Salvador, São Luís, São Paulo e Vitória, que mostraram porcentagens mais baixas do que os valores nacionais. Vale notar que, para mulheres com obesidade, o número de capitais com porcentagens abaixo da média nacional foi maior do que para os homens: Belo Horizonte, Campo Grande, Florianópolis, Palmas, Rio de Janeiro, Salvador, São Luís, São Paulo, Teresina e Vitória. A **Figura 11-2** mostra a porcentagem de adultos obesos nas capitais brasileiras e no Distrito Federal de acordo com dados do VIGITEL 2021, estratificados por quartis de obesidade. As capitais dos seguintes estados ocuparam o mais alto quartil de obesidade: Acre, Amazonas, Amapá, Rondônia, Mato Grosso e Goiás.

**Tabela 11-4:** – Porcentagem de adultos com obesidade (≥ 18 anos), por sexo, de acordo com as capitais brasileiras e o Distrito Federal

Capitais	Homens		Mulheres		Total	
	%	IC 95%	%	IC 95%	%	IC 95%
Aracaju	27,9	19,2 - 36,5	22,6	18,4 - 26,9	25,0	20,4 - 29,5
Belém	23,8	17,1 - 30,4	24,2	19,2 - 29,1	24,0	19,9 - 28,0
Belo Horizonte	20,7	14,3 - 27,0	22,0	17,3 - 26,6	21,4	17,5 - 25,2
Boa Vista	25,0	19,1 - 30,8	22,3	18,3 - 26,3	23,6	20,1 - 27,1
Campo Grande	19,4	12,4 - 26,3	21,0	16,1 - 25,9	20,2	16,1 - 24,4
Cuiabá	22,6	15,3 - 29,9	25,0	19,8 - 30,1	23,8	19,4 - 28,2
Curitiba	24,3	17,6 - 30,9	21,1	16,3 - 26,0	22,6	18,6 - 26,6
Florianópolis	21,4	15,0 - 27,9	19,2	14,6 - 23,7	20,2	16,4 - 24,1
Fortaleza	23,2	16,5 - 30,0	24,1	19,1 - 29,1	23,7	19,6 - 27,8
Goiânia	26,7	19,4 - 34,0	20,3	15,7 - 24,8	23,3	19,0 - 27,5
João Pessoa	22,0	15,3 - 28,8	25,1	19,4 - 30,9	23,7	19,3 - 28,1
Macapá	23,7	18,4 - 29,0	23,7	19,2 - 28,2	23,7	20,3 - 27,1
Maceió	23,7	16,2 - 31,2	25,4	18,8 - 31,9	24,6	19,7 - 29,5
Manaus	23,3	16,7 - 29,9	26,6	21,4 - 31,7	25,0	20,8 - 29,1
Natal	23,0	16,3 - 29,8	23,1	17,7 - 28,4	23,0	18,8 - 27,3
Palmas	23,3	14,9 - 31,6	16,1	12,2 - 19,9	19,5	15,0 - 24,0
Porto Alegre	22,9	16,0 - 29,8	22,4	16,4 - 28,3	22,6	18,1 - 27,1
Porto Velho	26,6	19,8 - 33,4	26,2	21,1 - 31,3	26,4	22,1 - 30,7
Recife	17,7	11,5 - 23,9	26,5	20,7 - 32,4	22,6	18,3 - 26,9
Rio Branco	25,0	18,7 - 31,3	23,4	18,7 - 28,0	24,2	20,3 - 28,0
Rio de Janeiro	23,1	15,8 - 30,4	20,1	15,7 - 24,5	21,5	17,4 - 25,6
Salvador	18,6	12,8 - 24,3	22,0	17,7 - 26,3	20,5	17,0 - 23,9
São Luís	18,6	11,1 - 26,0	17,6	13,3 - 22,0	18,0	13,9 - 22,2
São Paulo	20,6	14,8 - 26,3	24,2	19,2 - 29,1	22,5	18,8 - 26,3
Teresina	24,1	16,9 - 31,3	17,2	12,9 - 21,5	20,3	16,3 - 24,3
Vitória	19,2	13,0 - 25,4	16,8	12,9 - 20,6	17,9	14,4 - 21,4
Distrito Federal	23,0	16,6 - 29,4	22,2	17,2 - 27,3	22,6	18,6 - 26,6

Fonte: VIGITEL Brazil 2021. ^252^

**Figura 11-2:**
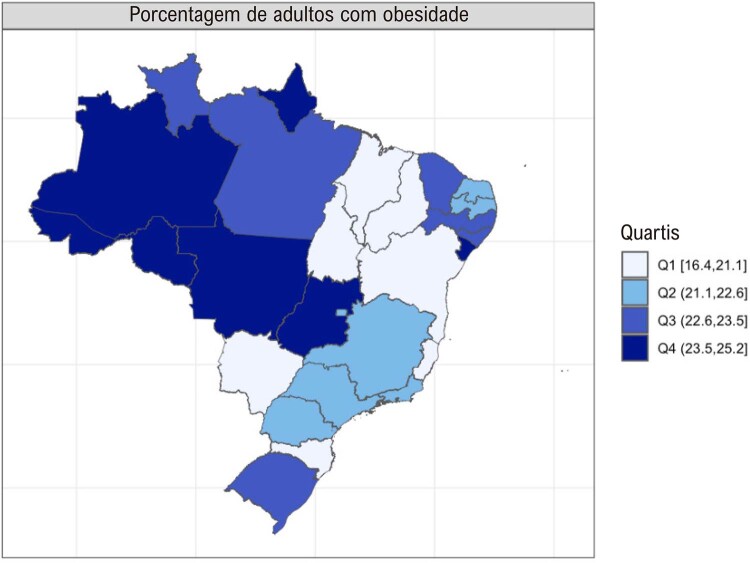
– Porcentagem de adultos com obesidade nas capitais brasileiras e no Distrito Federal, de acordo com dados do VIGITEL 2021, estratificados por quartis. Dados do VIGITEL Brasil 2021. ^252^

•As **Figuras 11-3** e **11-4** são séries temporais com as porcentagens de adultos com sobrepeso e obesos (≥ 18 anos), respectivamente, no Brasil, de 2006 a 2021, de acordo com dados do VIGITEL. Houve tendência de aumento nas porcentagens de sobrepeso e obesidade no Brasil de 2006 a 2021, de acordo com dados do VIGITEL.

### Crianças e adolescentes

•Um estudo transversal com base nos dados do Estudo sobre Risco Cardiovascular em Adolescentes avaliou 2.530 adolescentes de 12–17 anos arrolados em escolas públicas e privadas de Belo Horizonte. O estudo teve por objetivo associar fatores ambientais internos das escolas e suas vizinhanças com obesidade na adolescência naquela metrópole brasileira. A prevalência de obesidade foi 7,21%. Aspectos internos e externos relacionados à alimentação na escola, tais como número de bebedouros em funcionamento e comercialização de alimentos processados nas proximidades das escolas, foram associados com obesidade em adolescentes de uma metrópole brasileira.^374^

•Aumento na prevalência de obesidade também foi observado entre crianças e adolescentes brasileiros. Uma revisão sistemática e meta-análise de 53 estudos (n = 122.395) conduzidos de 1986 a 2015 mostrou prevalência de obesidade no período de 8,2% (IC 95%, 8,1–8,4%, I2 = 98,5%). Maior prevalência foi observada entre meninos (9,7% [9,4–9,9%], I2 = 97,4%) do que entre meninas (7,3% [7,1−7,5%], I2 = 96,1%). A prevalência aumentou de acordo com a década (1990: 6,5% [6,0–7,0%], I2 = 96,8%; 2000: 7,9% [7,7–8,0%], I2 = 98,8%; 2010: 12,0% [11,5–12,6%], I2 = 95,8%) e a região brasileira (Nordeste: 6,4% [6,2−6,7%], I2 = 98,1%; Norte: 6,7% [6,3−7,2%], I2 = 98,8%; Sudeste: 10,6% [10,2−11,0%], I2 = 98,2%; Sul: 10,1 [9,7−10,4%], I2 = 97,7%). Os autores concluíram que, para cada 100 crianças brasileiras, mais de 8 apresentaram obesidade nas três décadas e, nas estimativas mais recentes, 12 de cada 100 apresentaram obesidade infantil.^375^

## Incidência

•O estudo ELSA-Brasil incluiu 13.625 mulheres e homens de 35-74 anos (2008-2010) atendidos em consulta após acompanhamento médio de 3,8 anos. Os investigadores mediram altura e peso de todos os participantes durante as consultas presenciais. Relataram incidência global de 7,7% de sobrepeso e de 10,6% de obesidade, com mais altos níveis entre mulheres negras (28,5%), homens jovens (21,1%) e mulheres com baixo nível educacional (35,0%). As proporções de sobrepeso e obesidade aumentaram com a idade, mais comumente entre aqueles com os menores níveis de renda *per capita* e menos anos de escolaridade.^376^

## Mortalidade atribuída a obesidade

•Em 2021, 1,95 milhão (IC 95%, 1,12-2,91) de mortes cardiovasculares e 3,7 milhões (IC 95%, 1,97-5,49) de mortes em geral foram atribuídas a IMC elevado.^377^

•De 1990 a 2019, houve uma mudança negativa nas taxas de mortalidade por DCV atribuída a IMC alto em mulheres [-33,9 (-43,7;-16,7)], que foi maior do que a dos homens [-22,8 (-35,9;6,2)]. As maiores reduções na porcentagem de mortalidade ocorreram nas UF com mais altas rendas no Brasil.^4^

## Carga de Doença Atribuída a Obesidade

•Em 2021, o número de DALYs por todas as causas decorrentes de IMC elevado foi 1.560 (IC 95%, 711-2.380) por 100 mil.^377^

•A maioria das UF apresentou diminuição nas taxas de DALYs padronizadas por idade devidos a DCV atribuída a IMC elevado em mulheres de 1990 a 2019. Comportamento similar foi observado naquelas taxas em homens, com redução percentual de obesidade no período.^4^

## Impacto na Saúde Cardiovascular

•Obesidade e seus fatores de risco têm uma forte interação. No *EpiFloripa Aging Study* ,^378^ um estudo populacional longitudinal de base domiciliar realizado em Florianópolis, de 2009/2010 a 2013/2014, 559 indivíduos foram avaliados. Houve uma associação entre ter ou desenvolver sobrepeso (OR = 4,59; IC 95%, 3,05–6,89) e o diagnóstico de síndrome metabólica: esses indivíduos tiveram 4,71 vezes maior probabilidade de diagnóstico de síndrome metabólica do que aqueles que não tinham nem desenvolveram sobrepeso. No *Baependi Heart Study* ,^379^ estudo longitudinal para investigar fatores associados ao desenvolvimento de DCV nos cidadãos de Baependi, Minas Gerais, desde 2005, a incidência de hipertensão foi monitorada por 10 anos e foi 24,3%. O IMC foi o melhor preditor de hipertensão em um modelo que incluiu idade, sexo, colesterol da lipoproteína de alta densidade e pressão arterial sistólica.

•Importante notar que a associação entre obesidade e hipertensão é também observada nos jovens como descrito por Santiago *et al* .^380^ Em estudo transversal de 1.132 adolescentes (16,50 ± 1,14 anos) dos dois sexos, os do sexo masculino com excesso de adiposidade periférica, central e geral apresentaram maior probabilidade de pressão arterial sistólica alta.

•Por fim, dados de duas coortes do estudo transversal *Health Survey of São Paulo focusing on Nutrition* ( *ISA-Nutrition* ), uma sub-amostra do *Health Survey of São Paulo* ( *ISA-Capital* ), conduzido em 2008 e 2015, mostraram associação de excesso de peso corporal com maior chance de apresentar todos os outros fatores de risco analisados.^381^ A distribuição da gordura corporal é também um determinante de risco, sendo menor acúmulo de gordura corporal considerado protetor em oposição a obesidade abdominal. Oliveira *et al* .^333^ estimaram, entre 10.917 participantes do estudo ELSA-Brasil, as associações da relação ‘gordura de membros inferiores/tronco’ com o risco de DCV em 10 anos, de acordo com o escore de risco Framingham, estratificados por gênero e ajustados por idade, cor da pele, nível educacional, consumo de álcool, atividade física de lazer, uso de hipolipemiante, e *status* menopausal. Uma relação ‘gordura de membros inferiores/tronco’ mais alta foi relacionada a menor risco de DCV em 10 anos, assim como redução na pressão arterial sistólica, colesterol total e uso de anti-hipertensivos, especialmente em mulheres.

•Uma meta-análise com 46 estudos avaliou a associação entre urbanização (incluindo dados de desmatamento da floresta Amazônica) e fatores de risco cardiometabólicos e desfechos. A avaliação de 20.574 adultos de 33 etnias indígenas brasileiras mostrou taxas mais altas de obesidade (região Centro-Oeste: 23% [IC 95%, 17–29]; e região Sul: 23% [13–34]) nos povos nativos residentes em áreas urbanas no Brasil e menores taxas de obesidade (11% [IC 95%, 8–15]) naqueles residentes de áreas menos urbanizadas do Brasil (região Norte). A prevalência de obesidade foi 3,5 vezes mais alta entre os residentes de territórios indígenas urbanizados (28%) do que entre aqueles residindo em áreas com mais de 80% de floresta Amazônica nativa (8%). Além disso, entre 1997 e 2019, a taxa de mortalidade por DCV entre aqueles residindo na região Sudeste (a mais urbanizada) foi 2,5 vezes maior do que a observada na região Norte.^259^

## Utilização e Custo da Atenção à Saúde

•A obesidade está associada à elevação dos custos diretos (por exemplo, para o diagnóstico e tratamento da doença) e indiretos (relacionados à perda dos ganhos laborais e mortalidade prematura). Em estudo interessante, Ferrari *et al* .^337^ estimaram a carga econômica das doenças atribuídas a sobrepeso e obesidade no SUS. Os autores desenvolveram um modelo de macro simulação para estimar a carga econômica das doenças atribuídas a IMC elevado no Brasil. Dados autorrelatados sobre peso e altura da PNS brasileira conduzida em 2019 foram usados para calcular a distribuição do IMC e incluíram 85.715 adultos com idade ≥20 anos. A média e o desvio-padrão do IMC e a prevalência de sobrepeso e obesidade foram calculados por sexo e UF brasileira. Os custos diretos da assistência foram obtidos do SIA/SUS e do SIH/SUS em 2019. Os resultados mostraram que, no Brasil, em 2019, US$ 654 milhões em custos diretos na atenção à saúde relacionados a doenças não comunicáveis foram atribuídos a IMC elevado. Os custos atribuídos foram mais altos entre mulheres do que entre homens. A doença com os mais altos custos atribuídos foi a DCV, seguida por doenças respiratórias crônicas, neoplasias, doenças digestivas, distúrbios musculoesqueléticos, diabetes e doenças renais. Nas mulheres, as doenças com os mais altos custos atribuídos foram neoplasias, doenças digestivas, distúrbios musculoesqueléticos, diabetes e doenças renais, enquanto, nos homens, foram DCV e doenças respiratórias crônicas. Os mais altos custos atribuídos foram encontrados nas UF das regiões Sudeste e Sul, São Paulo, seguido de Minas Gerais e Paraná. Esses dados reforçam o impacto da obesidade na saúde e na economia brasileiras, mostrando a necessidade de políticas públicas para controlar essa doença, o que pode resultar em economia de custos além dos óbvios benefícios à saúde populacional.

## Obesidade e COVID-19

•A pandemia de COVID-19 levou a observações em todo o mundo da associação entre obesidade e desfechos adversos nos indivíduos infectados.^382 - 386^ Em estudo multicêntrico de base hospitalar com 8.183 pacientes admitidos em UTI que testaram positivo para SARS-CoV-2,^387^ ao estratificar por categorias de IMC e ajustando para idade, sexo e *status* de tabagismo, os pacientes com obesidade grave mostraram maior risco de mortalidade por COVID-19 (HR 1,21; IC 95%, 1,03–1,43) em comparação àqueles com ‘peso normal/sobrepeso’. Não houve diferença para as categorias ‘obesidade leve/moderada’ (HR 0,91; IC 95%, 0,83–1,00) e ‘peso abaixo do normal’ (HR 1,21; IC 95%, 0,80–1,81). Ademais, para os sobreviventes na categoria de mais alto IMC (≥ 40 kg/m^2^), a permanência na UTI foi 31% mais longa em comparação à daqueles na categoria ‘peso normal/sobrepeso’. Permanência mais longa na UTI foi também observada na categoria de ‘obesidade leve/moderada’ em comparação à de ‘peso normal/sobrepeso’. Não houve diferença para a categoria ‘abaixo do peso’. Entretanto, ao estratificar por idade, observou-se aumento do risco de mortalidade apenas para os pacientes mais jovens (< 60 anos) em comparação àqueles com ‘peso normal/sobrepeso’ (HR 1,27; IC 95%, 1,01–1,61). Observou-se risco aumentado de morte para pacientes abaixo do peso (HR 3,74; IC 95%, 1,39–10,07). Para os pacientes com idade ≥ 60 anos, ‘obesidade leve/moderada’ foi associada com risco reduzido de mortalidade (HR 0,87; IC 95%, 0,78–0,97).

•Por outro lado, estudo de coorte multicêntrico conduzido em oito estados da região Nordeste, GENSCoV-BR,^386^ analisou os dados demográficos, clínicos e antropométricos de indivíduos que testaram positivo para SARS-CoV-2, além dos desfechos que incluíram hospitalização, ventilação mecânica e morte, para avaliar os efeitos dos extremos de peso corporal nos desfechos clínicos. Dentre 1.308 indivíduos, excesso de peso corporal foi observado em 66,9%, baixo peso em 2,7% e obesidade em 32,4%, de acordo com a classificação da OMS. Hospitalização ocorreu em 75,2% e 22,2% morreram. Hospitalização foi mais frequentemente observada entre os indivíduos com baixo peso (3,2% *vs.* 1,2%) e sobrepeso (68,1% *vs.* 63,3%), embora, na análise multivariada, o peso corporal (baixo peso ou sobrepeso) não tenha sido associado a risco de hospitalização, ventilação mecânica ou morte. Os diferentes resultados podem refletir as diferentes características das populações estudadas e, mais importante, que os estudos foram focados em ou pacientes já hospitalizados e, portanto, mais graves ou pacientes ambulatoriais com COVID-19 menos grave.^387^

•As tendências temporais do VIGITEL mostram que, entre 2006 (início do sistema VIGITEL) e 2019 (antes da pandemia), houve aumento na prevalência de sobrepeso, que passou de 55,4% (IC 95%, 54,4-56,3) em 2019 para 57,2% (IC 95%, 55,7-58,8) em 2021/22. Ademais, a prevalência de obesidade aumentou de 20,3% (IC 95%, 19,5-21,0) em 2019 para 22,4% (IC 95%, 21,1-23,6) em 2021/22. A obesidade também aumentou nos homens, de 19,5% (IC 95%, 18,3-20,7) para 22,0% (IC 95%, 20,0-24,0).^388^

## Perspectivas

•Considerando o aumento da obesidade no mundo, inclusive no Brasil, e sua contribuição para DCV, esforços são necessários para controlá-la. Caso contrário, os resultados favoráveis observados nos anos anteriores, mostrando redução na mortalidade por DCV, podem logo se perder. Nas últimas décadas, programas exitosos de controle do tabagismo foram implementados no Brasil, indicando que intervenções em âmbito nacional direcionadas ao controle do peso podem ser instituídas. Políticas no âmbito nacional são necessárias para prevenir a obesidade e reduzir a prevalência da obesidade estabelecida. Intervenções de base comunitária, sustentáveis e multidisciplinares que visem o aumento da prática de atividade física, a redução do consumo de alimentos ultraprocessados, a promoção da ingestão de alimentos saudáveis e o reforço da importância das diretrizes dietéticas para a população brasileira são fundamentais.

•Por fim, várias discussões sobre o uso de novos medicamentos para redução de peso, incluindo sobre sua custo-efetividade, são necessárias, pois eles são uma realidade e parecem ter vindo para ficar.

## CAPÍTULO 12 – ATIVIDADE FÍSICA

### Ver Tabelas 12-1 a 12-5 e Figuras 12-1 a 12-5

**Table:** Abreviaturas usadas no Capítulo 12

COVID-19	Doença do Novo Coronavírus 2019
DALYS	Anos de vida perdidos ajustados por incapacidade (do inglês, *Disability-Adjusted Life-Years* )
DCV	Doenças Cardiovasculares
ELSA-Brasil	Estudo Longitudinal da Saúde do Adulto - Brasil
GBD	Carga Global de Doenças (do inglês, *Global Burden of Disease* )
IC	Intervalo de confiança
II	Intervalo de incerteza
MET	Equivalente metabólico
min	minuto
PENSE	Pesquisa Nacional de Saúde do Escolar
PNS	Pesquisa Nacional de Saúde
SDI	Índice sociodemográfico (do inglês, *sociodemographic index* )
SUS	Sistema Único de Saúde
TV	Televisão
VIGITEL	Sistema de Vigilância de Fatores de Risco e Proteção para Doenças Crônicas por Inquérito Telefônico
V̇O_2_	Consumo de oxigênio

### Introdução

•“Atividade física” e “exercício físico” têm significados diferentes. Conceitua-se atividade física como qualquer movimento corporal realizado com a contração dos músculos esqueléticos resultando em um aumento do gasto energético em relação ao estado de repouso.^389 , 390^ Atividade física pode ser traduzida como atividades recreacionais ou ocupacionais realizadas de forma espontânea. Alguns exemplos são: caminhadas até o trabalho, limpar a casa, cortar a grama, subir e descer escadas, etc. Exercício físico é um tipo de atividade física. Para que uma atividade física seja categorizada como exercício físico, duas premissas básicas devem ser respeitadas: (a) deve ser estruturada; e (b) deve ser realizada com o intuito de se atingir ou melhorar valências físicas, como desempenho, força, potência, resistência, equilíbrio, etc.^391 , 392^

•Já os termos “sedentarismo” e “inatividade física” apresentam controvérsia em relação às suas definições.^393^Sedentarismo pode ser definido como a realização de atividades com um gasto de energia muito baixo, de 1,5 MET ou menos.^394^ Exemplos típicos desse quadro incluem muitas horas em frente à TV ou ao *tablet* . Inatividade física refere-se à realização de um volume de atividade física semanal menor do que o recomendado pelas diretrizes atuais, ou seja, menos de 150 min/semana de atividade física de intensidade moderada ou menos de 75 min/semana de atividade física de alta intensidade, um volume correspondente a 450 MET-min/semana.^395^Outros termos para essa definição podem ser encontrados na literatura, como “prática insuficiente de atividade física” utilizado pelo VIGITEL Brasil.^252^

•Um MET equivale ao V̇̇O_2_ estimado, quando em repouso, de aproximadamente 3,5 mL.kg^- 1^.min^- 1^. Pode-se usar a seguinte classificação relacionada ao V̇O_2 _necessário para realizar determinada atividade física, ou seja, à sua intensidade: (a) ≤1,5 MET = comportamento sedentário; (b) >1,5 e <3 METs = atividade física de intensidade baixa/leve; (c) ≥3 e <6 METs = atividade física de intensidade média/moderada; (d) ≥6 METs = atividade física de intensidade alta/vigorosa. De forma ainda mais prática, quando se recomenda de 2,5 a 5 horas de atividade de intensidade moderada por semana, isso equivale a aproximadamente 7,5 a 29,5 MET-hora/semana ou 450 a 1770 MET-min/semana (equivalente a 150 a 300 min/semana de exercício de intensidade moderada) - sendo essa a recomendação padrão para a prática de atividade física em adultos.^395^

•Para crianças e adolescentes, a recomendação é uma média de 60 min/dia de atividade física aeróbica de intensidade moderada/alta. Para os idosos (>65 anos de idade), a recomendação é atividade física em intensidade leve/moderada em 3 ou mais dias por semana, com foco também em atividades que visem reduzir o risco de quedas e melhorar a função muscular.^395 , 396^ Ademais, tais diretrizes enfatizam que a prática de atividade física de intensidade moderada além dos 300 min/semana pode trazer benefícios adicionais para a saúde.^396^

•No Brasil, o primeiro Guia de Atividade Física para a População Brasileira, publicado pelo Ministério da Saúde em 2021,^397^ também traz recomendações semelhantes àquelas supracitadas ( **Tabela 12-1** ). Além disso, destaca-se a importância da inclusão de atividades que visem o fortalecimento muscular e ósseo em pelo menos 2 dias/semana. Crianças e adolescentes (5-17 anos de idade) devem se engajar em pelo menos 1 hora/dia de atividade física e pelo menos 3 dias/semana devem ser destinados ao fortalecimento muscular e ósseo. Em relação ao tempo de tela, a Academia Americana de Pediatria^398^ recomenda que crianças com menos de 18 meses não despendam qualquer tempo em frente à tela e que aquelas com idade entre 18 meses e 5 anos despendam no máximo 1 hora/dia de tela. Já para crianças com 6 anos ou mais, não há um tempo máximo definido, desde que o tempo de tela não interfira no sono ou nos níveis de atividade física.

**Tabela 12-1:** – Recomendações para a prática de atividade física de acordo com o Primeiro Guia de Atividade Física para a População Brasileira

Atividade Física	Jovens (6-17 anos de idade)	Adultos	Idosos
Se intensidade média/moderada	≥60 min/dia*	≥150 min/semana	≥150 min/semana
Se intensidade alta/vigorosa	NM	≥75 min/semana	≥75 min/semana
Fortalecimento muscular	Incluir nos 60 min/dia	≥2 dias/semana	2-3 vezes/semana
Equilíbrio	NM	NM	2-3 vezes/semana

*Podendo ser dividida em blocos; NM: Não mencionado. Fonte: VIGITEL Brasil 2021. ^252^

### Prevalência

•De acordo com os dados do VIGITEL 2021, aproximadamente 48% da população brasileira adulta não alcança o nível recomendado de atividade física, com percentuais mais elevados entre as mulheres (56%) em comparação com os homens (39%). Observou-se a maior frequência de adultos considerados inativos em Porto Alegre (51,8%) e a menor, em Goiânia (39,8%).^252^ O percentual de homens fisicamente inativos variou entre 30,3% em Goiânia e 46,1% em Campo Grande, enquanto, para as mulheres, esse percentual variou de 43,4% em Florianópolis a 63,1% no Rio de Janeiro ( **Figura 12-1 e Tabela 12-2** ).

**Figura 12-1:**
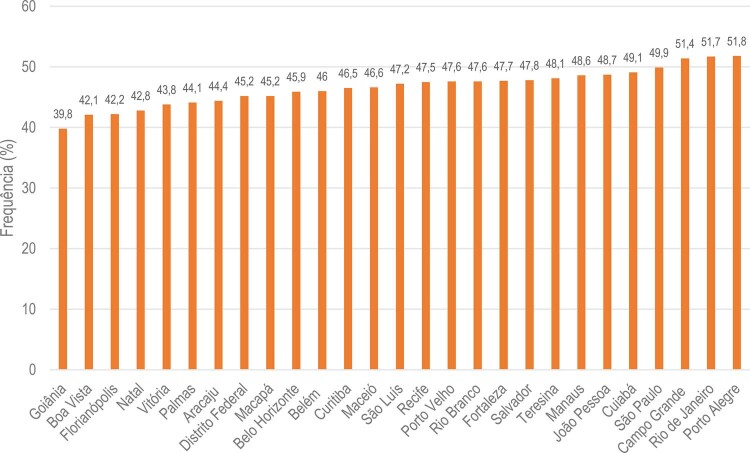
– Frequência de adultos com idade ≥18 anos fisicamente inativos nas capitais dos estados brasileiros e Distrito Federal de acordo com dados do VIGITEL 2021. Fonte: VIGITEL Brasil 2021. ^252^

**Tabela 12-2:** – Frequência de adultos (≥18 anos) fisicamente inativos, por sexo, segundo as capitais dos estados brasileiros e Distrito Federal de acordo com dados do VIGITEL 2021

Capitais	Homens		Mulheres	
	%	IC 95%	%	IC 95%
Aracaju	37,6	29,1 - 46,2	49,9	44,4 - 55,5
Belém	36,2	29,1 - 43,3	54,2	48,6 - 59,9
Belo Horizonte	40,1	33,0 - 47,3	50,8	45,2 - 56,3
Boa Vista	33,7	27,3 - 40,1	49,8	44,9 - 54,7
Campo Grande	46,1	36,7 - 55,6	56,1	49,3 - 62,9
Cuiabá	44,8	36,2 - 53,3	53,1	46,5 - 59,6
Curitiba	37,8	30,9 - 44,8	54,1	47,9 - 60,3
Florianópolis	40,9	33,1 - 48,7	43,4	37,3 - 49,4
Fortaleza	36,6	29,2 - 44,0	57,1	51,1 - 63,1
Goiânia	30,3	23,4 - 37,3	48,2	42,2 - 54,1
João Pessoa	43,5	35,1 - 51,9	53	46,7 - 59,4
Macapá	38	31,5 - 44,4	52	46,5 - 57,4
Maceió	40,1	32,2 - 48,0	52	44,9 - 59,1
Manaus	37,3	29,1 - 45,4	59,2	53,5 - 64,8
Natal	33,9	26,7 - 41,1	50,4	44,3 - 56,4
Palmas	38,1	29,5 - 46,6	49,4	43,8 - 55,0
Porto Alegre	42,3	34,9 - 49,7	59,6	52,0 - 67,3
Porto Velho	40,9	33,3 - 48,4	54,9	49,2 - 60,6
Recife	34,4	26,3 - 42,5	58,1	52,1 - 64,1
Rio Branco	39,8	32,7 - 46,8	54,7	48,8 - 60,6
Rio de Janeiro	38,2	30,3 - 46,0	63,1	57,1 - 69,0
Salvador	38,1	30,5 - 45,7	55,8	50,1 - 61,4
São Luís	36	28,4 - 43,7	56,3	50,6 - 61,9
São Paulo	43,2	36,4 - 50,1	55,6	49,3 - 61,8
Teresina	37	29,3 - 44,7	57,3	51,4 - 63,2
Vitória	38	30,6 - 45,3	48,8	42,8 - 54,8
Distrito Federal	38,1	30,4 - 45,7	51,4	45,2 - 57,7

IC: intervalo de confiança. Fonte: VIGITEL Brasil 2021. ^252^

•Para ambos os sexos, a idade apresentou uma associação direta com a frequência de inatividade física ( **Tabela 12-3** ). Na faixa etária entre 18 e 24 anos, a frequência de inatividade física foi de 35,6% (27,6% entre os homens e 44,5% entre as mulheres), enquanto, nos indivíduos com idade maior ou igual a 65 anos, esse percentual foi de 73% (65,6% entre os homens e 78,2% entre as mulheres).^252^

**Tabela 12-3:** – Frequência de adultos (≥ 18 anos) fisicamente inativos, por sexo e faixa etária, de acordo com dados do VIGITEL 2021

	Homens		Mulheres		Total	
Faixa de idade	%	IC 95%	%	IC 95%	%	IC 95%
18 a 24 anos	27,6	22,5 - 32,7	44,5	37,6 - 51,4	35,6	31,3 - 39,8
25 a 34 anos	33,9	28,0 - 39,7	50,2	44,7 - 55,6	42,6	38,5 - 46,7
35 a 44 anos	37,0	31,5 - 42,5	51,4	46,9 - 55,9	45,0	41,5 - 48,6
45 a 54 anos	37,8	33,1 - 42,4	53,8	50,3 - 57,4	46,3	43,3 - 49,2
55 a 64 anos	48,4	43,6 - 53,3	62,8	59,6 - 65,9	56,5	53,8 - 59,3
65+ anos	65,6	62,2 - 68,9	78,2	76,4 - 80,0	73,0	71,3 - 74,7

IC: intervalo de confiança. Fonte: VIGITEL Brasil 2021. ^252^

•Uma relação inversa foi observada entre escolaridade e inatividade física. Entre aqueles com 0 a 8 anos de escolaridade, a frequência de inatividade física observada foi de 58,4% (47,7% entre os homens e 67,9% entre as mulheres), enquanto, entre aqueles com 12 ou mais anos de escolaridade, essa frequência foi de 43,5% (39,7% entre os homens e 46,3% entre as mulheres).^252^

•A **Figura 12-1** ilustra a frequência de inatividade física nas 26 capitais dos estados brasileiros e Distrito Federal, segundo dados do VIGITEL 2021.^252^

•Em relação à adoção de comportamentos sedentários, em 2021, 66% dos adultos das 26 capitais dos estados brasileiros e Distrito Federal responderam despender ao menos 3 horas/dia do seu tempo livre vendo TV ou usando computador, *tablet* ou celular, sendo essa frequência semelhante em homens (66,7%) e mulheres (65,4%).^252^ Os menores percentuais foram observados em Recife (58,9%), entre os adultos com idade ≥65 anos (51%) e entre aqueles com 0 a 8 anos de escolaridade (49,2%). Enquanto isso, as maiores frequências foram encontradas na cidade do Rio de Janeiro (70,1%), na faixa etária de 18-24 anos (83,2%) e entre aqueles com 12 ou mais anos de escolaridade (73%).

•A **Figura 12-2** ilustra a frequência de adultos (≥18 anos) que despendem diariamente ≥3 horas do seu tempo livre assistindo à TV ou usando computador, *tablet* ou celular nas 26 capitais dos estados brasileiros e Distrito Federal, segundo dados do VIGITEL 2021.^252^

•Dados provenientes do estudo ELSA-Brasil entre 2008 e 2010, com 15.105 homens e mulheres com idade de 35-74 anos, revelaram que menos da metade dessa população praticava atividade física. Nesse estudo, observou-se uma prevalência de 44,1% de atividade física no tempo livre entre homens e 33,8% entre mulheres. Essa proporção foi maior entre indivíduos com idade igual ou superior a 60 anos, tanto homens (46,7%) quanto mulheres (39,7%).^399^

### Tendências

•Segundo a série histórica dos dados do VIGITEL,^400^ a frequência de adultos (≥18 anos) fisicamente inativos manteve-se estável entre 2013 e 2020, sendo de 49,4% e 47,2%, respectivamente, resultando em uma variação anual média aferida em pontos percentuais ao ano (pp/ano) de -0,48 (IC 95%, -1,15;0,19). Essa estabilidade também foi observada avaliando-se separadamente os sexos. Em 2013, 39,9% dos homens e 57,4% das mulheres eram fisicamente inativos e, em 2020, esses números foram de 37,3% e de 55,6%, respectivamente, correspondendo a uma variação anual média de -0,50 (IC 95%, -1,20;0,21) pp/ano entre homens e de -0,47 (IC 95%, -1,25;-0,30) pp/ano entre mulheres.

•No entanto, a variação entre 2013 e 2020 não foi semelhante comparando-se as diferentes faixas etárias e níveis educacionais. Enquanto nas faixas etárias de 18 a 24 anos, 25 a 34 anos e 55 a 64 anos houve estabilidade da frequência de adultos fisicamente inativos, observou-se redução desse número nas faixas etárias de 35 a 44 anos (-0,68 pp/ano; IC 95%, -1,34;-0,02), de 45 a 54 anos (-1,20 pp/ano; IC 95%, -1,59;-0,81) e de 65 anos ou mais (-0,55 pp/ano; IC 95%, -0,84;-0,26).^400^ Em relação ao nível educacional, entre os adultos com 0 a 8 anos e com 9 a 11 anos de escolaridade, houve estabilidade do percentual de indivíduos fisicamente inativos, mas, entre aqueles com 12 ou mais anos de escolaridade, houve redução desse percentual (-0,45 pp/ano; IC 95%, -0,86;-0,04).^400^

•A variação do percentual de adultos com idade ≥18 anos fisicamente inativos de acordo com dados brasileiros referentes aos 5 últimos anos (2017-2021) disponibilizados pelo VIGITEL pode ser vista na **Figura 12-3** .^400^

**Figura 12-3:**
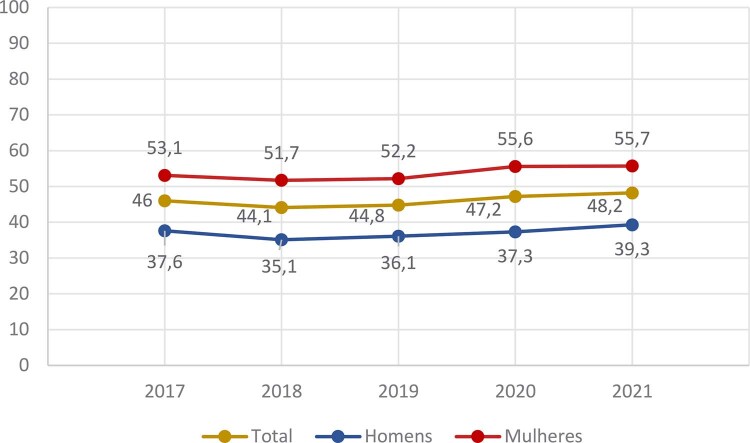
– Percentual de adultos com idade ≥18 anos fisicamente inativos* no Brasil, de acordo com sexo, nos últimos 5 anos disponíveis pela coleta do VIGITEL. *Realização de menos de 150 min/semana de atividades de intensidade moderada, ou menos de 75 min/semana de atividades de intensidade vigorosa Fonte: VIGITEL Brasil 2021. ^252^

•De forma semelhante, dados da PNS revelaram um aumento relativo de mais de 30% na proporção de adultos brasileiros que afirmaram se engajar em pelo menos 150 min/semana de atividade física moderada a vigorosa no período de 2013 a 2019, subindo de 22,7% em 2013 para 30,1% em 2019. Em ambos os anos, essa proporção foi mais alta entre homens (27,3% e 34,2%, respectivamente), jovens entre 18 e 24 anos (35,6% e 41,0%, respectivamente), brancos (23,9% e 31,6%, respectivamente) e indivíduos com alto nível de escolaridade (38,8% e 49,1%, respectivamente).^401^

•No entanto, de maneira oposta, a PENSE constatou uma redução na atividade física de estudantes de 13-17 anos de idade de escolas brasileiras entre 2015 e 2019. Segundo esse estudo, o percentual de atividade física diminuiu de 31,6% em 2015 para 28,1% em 2019.^402^

•Já em relação à adoção de comportamentos sedentários, entre 2010 e 2020, houve redução da frequência de adultos que despendem ao menos 3 horas/dia do tempo livre assistindo à TV, passando de 27,3% em 2010 para 26,5% em 2020 (-0,31 pp/ano; IC 95%, -0,61;-0,01).^400^ Após estratificação por sexo, idade e nível educacional, essa redução foi observada apenas nos homens (-0,54 pp/ano; IC 95%, -0,76;0,32), nas faixas etárias de 18 a 24 anos (-1,49 pp/ano; IC 95%, -1,83;-1,15) e de 25 a 34 anos (-0,57; IC 95%, -1,12;-0,02), e naqueles com escolaridade de 9 a 11 anos (-0,43 pp/ano; IC 95%, -0,69;-0,18) e 12 ou mais anos (-0,46 pp/ano; IC 95%, -0,79;-0,13). Em mulheres, adultos com idade acima de 34 anos e aqueles com 0 a 8 anos de escolaridade, houve estabilidade dessa frequência.

•Comparando com a situação global, estima-se que 27,5% dos adultos e mais de 81% dos adolescentes não atendam às recomendações internacionais para a prática de atividade física aeróbica.^395^Tais informações sugerem que mais de 1,4 bilhão de adultos estão em risco de desenvolver ou agravar doenças relacionadas à falta de atividade física.^403^

### Mortalidade por Doenças Cardiovasculares Atribuíveis à Baixa Atividade Física

•Segundo dados do GBD de 2019 referentes ao Brasil,^404^ na avaliação da taxa de mortalidade por DCV atribuíveis aos fatores de risco selecionados, o baixo nível de atividade física, definido como <3000 MET-min/semana, passou de oitavo lugar no *ranking* dos fatores de risco mais importantes em 1990 para o sétimo lugar em 2019. Apesar disso, houve uma redução da taxa de mortalidade padronizada por idade por DCV atribuíveis ao baixo nível de atividade física, que passou de 26,1 (II 95%, 12,6;41,4) por 100 mil habitantes em 1990 para 13,7 (II 95%, 7,6;20,8) por 100 mil habitantes em 2019 ( **Figura 12-4** ). Em termos percentuais, no Brasil, essa redução foi de 47,6% (II 95%, -53,6;-35,0), enquanto, nos diferentes estados brasileiros e Distrito Federal, foram observadas reduções que variaram de 57,1% (II 75%, -63,1;-46,3) em São Paulo a 3,6% (II 75%, -22,9;29,8) no Maranhão ( **Tabela 12-4 e Figura 12-5** ). Na comparação entre os sexos, a taxa de mortalidade padronizada por idade por DCV foi semelhante em homens [15,4 (II 95%, 7,9;24,5) por 100 mil habitantes] e mulheres [12,3 (II 95%, 7,4;17,9) por 100 mil habitantes], assim como a redução percentual entre os anos de 1990 e 2019 [-42,9% (II 95%, -50,3;-24,9) nos homens e -50,9% (II 95%, -56,7;-39,7) nas mulheres]. Já na comparação entre as faixas etárias, pode-se observar um aumento da taxa de mortalidade atribuível ao baixo nível de atividade física com o avançar da idade tanto em 1990 quanto em 2019, porém a redução percentual foi semelhante nas diferentes faixas etárias, sendo a maior redução observada entre 35 e 39 anos de idade [-55,0% (II 75%, -65,9;-39,6)] e a menor redução observada entre 75 e 79 anos de idade [-41,0% (II 75%, -50,0;-16,8)] ( **Tabela 12-5** ). A **Figura 12-5** mostra a variação percentual da taxa de mortalidade padronizada por idade entre 1990 e 2019 nas unidades federativas brasileiras.

**Figura 12-4:**
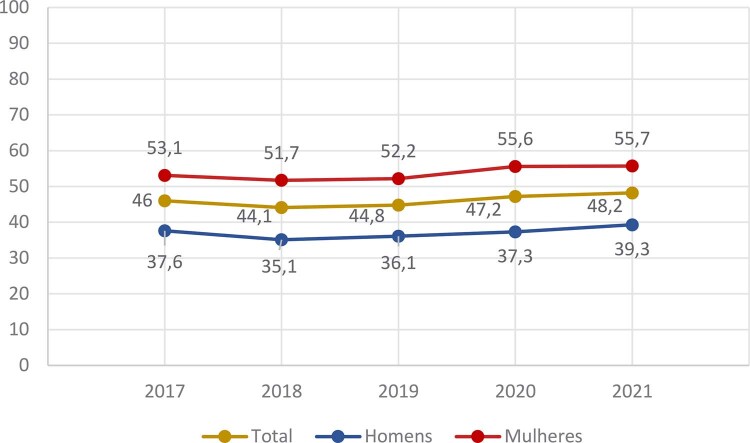
– Taxa de mortalidade padronizada por idade (por 100 mil habitantes) por doença cardiovascular atribuível ao baixo nível de atividade física no Brasil de 1990 a 2019. Fonte: Dados derivados do Global Burden of Disease Collaborative Network. Global Burden of Disease (GBD) Cardiovascular Burden Estimates 1990 and 2021, Institute for Health Metrics and Evaluation, University of Washington. ^48^

**Tabela 12-4:** – Número de mortes e taxas de mortalidade padronizadas por idade (por 100 mil habitantes) por doenças cardiovasculares atribuíveis ao baixo nível de atividade física e percentual de variação das taxas. Brasil e suas unidades federativas, 1990 e 2019

Localidade	1990		2019		Percentual de variação (II 95%)
	Número (II 95%)	Taxa (II 95%)	Número (II 95%)	Taxa (II 95%)	
Brasil	17147,7(7861,7;28644,3)	26,1(12,6;41,4)	30228,8(16769,4;46333,5)	13,7(7,6;20,8)	-47,6%(-53,6;-35)
Acre	21,8(9,7;36,4)	21,9(10,7;34,5)	68,2(38,2;104,4)	14,2(8,3;21,1)	-35,3%(-45,2;-14,9)
Alagoas	251,4(112,3;418,9)	24(11;38,8)	509,9(275,7;799,5)	17(9,1;26,5)	-29,2%(-42,3;-4)
Amapá	12,3(5,6;20,6)	18,6(8,9;29,6)	48,8(25,9;75,6)	12(6,7;18,1)	-35,5%(-44;-19,1)
Amazonas	113,1(55;183,9)	23,5(12;36,4)	297,4(171,5;447,1)	12,2(7,3;18)	-48,2%(-56,2;-31,1)
Bahia	1080,1(499,9;1771,1)	19,4(9,2;31,2)	2055,6(1108,1;3238,8)	12,6(6,8;19,9)	-35,1%(-46,4;-14,4)
Ceará	644,7(309,2;1029,1)	17,3(8,5;27,4)	1548,5(873,3;2352,3)	15,9(9;24,2)	-8%(-26,4;26,1)
Distrito Federal	87,6(37,3;151,9)	34,7(17;54,1)	237,8(126,2;359,2)	15,2(8,6;22,3)	-56,1%(-62,6;-44,1)
Espírito Santo	264(118,2;448,6)	26,9(12,6;43,5)	559,8(290,8;885,6)	14,2(7,5;22,1)	-47,1%(-56;-33,2)
Goiás	332,8(142,5;581,3)	25,1(11,8;40,9)	764,8(394,5;1253,4)	12,6(6,7;20,1)	-49,9%(-60,5;-33,2)
Maranhão	419,2(180,7;739,7)	20,6(9,7;34,8)	1245,5(676;1929,2)	19,9(10,8;30,7)	-3,6%(-22,9;29,8)
Mato Grosso	117,7(51,3;209,9)	23,4(10,9;38,8)	315,6(170,7;506,9)	11,4(6,3;17,8)	-51,4%(-59,5;-35,6)
Mato Grosso do Sul	148,2(64,4;261,4)	25,4(12;41,7)	347,4(185;539,9)	13,3(7,3;20,4)	-47,7%(-55,5;-34,1)
Minas Gerais	1832,8(822,4;3137,1)	27,2(12,7;43,8)	2810,5(1595,3;4299,8)	10,8(6,2;16,5)	-60,3%(-66,2;-49,2)
Pará	363,1(166,7;606)	26(12,7;41,7)	841(467,1;1318)	13,5(7,7;20,9)	-48,1%(-57,2;-31,6)
Paraíba	435,4(214,1;687,8)	20,7(10,4;32,4)	814,9(492,9;1200,5)	16,2(9,7;23,9)	-21,8%(-36,2;4,4)
Paraná	972,1(430,2;1665,5)	30,9(14,5;49,5)	1575(837,5;2538,4)	13,4(7,2;21,1)	-56,5%(-62,6;-45,7)
Pernambuco	923,7(430,7;1521,5)	26,1(12,7;41,7)	1639(877,2;2531,7)	17,6(9,4;27)	-32,4%(-42,9;-16,4)
Piauí	220,5(101;381,8)	22,7(10,6;37,3)	517,8(292;786,7)	13,2(7,4;20,1)	-41,6%(-52,2;-22,1)
Rio de Janeiro	2527,4(1210,1;4104,4)	35,7(17,7;55,1)	3428,7(1923,7;5200,1)	16(9;24)	-55,2%(-61,5;-44,6)
Rio Grande do Norte	249,4(117,3;415,7)	17,4(8,3;28,7)	475,4(262;737,3)	11,8(6,5;18,3)	-32,2%(-45,2;-12,1)
Rio Grande do Sul	1347,6(614,2;2263,3)	28,1(13,6;44,9)	2051,2(1105,1;3132,1)	13,6(7,3;20,6)	-51,7%(-58,2;-39,1)
Rondônia	51,7(20,4;91,5)	37,7(18,2;59,7)	203,7(115,8;310,3)	15,8(9,2;23,7)	-58%(-66,4;-41,1)
Roraima	7,5(3,2;13,3)	27,8(13,2;44,3)	34,4(18,5;54,3)	14,5(8,2;21,7)	-47,8%(-55,6;-30,4)
Santa Catarina	519,7(238,6;858,8)	30,2(14,8;47,1)	956,6(535,1;1478,4)	13,8(7,9;21)	-54,4%(-61,4;-41,4)
São Paulo	4025(1845,2;6915,6)	30,2(14,8;48,3)	6410,1(3530,9;9836,9)	13(7,3;19,7)	-57,1%(-63,1;-46,3)
Sergipe	125,7(56,4;211,7)	23,5(11,1;38,4)	280,1(146,8;447,1)	13,3(7,1;21)	-43,4%(-53,5;-29,3)
Tocantins	53,3(23,2;95,2)	25,8(12,6;41,6)	190,8(100,9;302)	14,9(8;23,1)	-42,2%(-52,4;-25,8)

II: intervalo de incerteza. Fonte: Dados derivados do Global Burden of Disease Collaborative Network. Global Burden of Disease (GBD) Cardiovascular Burden Estimates 1990 and 2021, Institute for Health Metrics and Evaluation, University of Washington. ^48^

**Figura 12-5:**
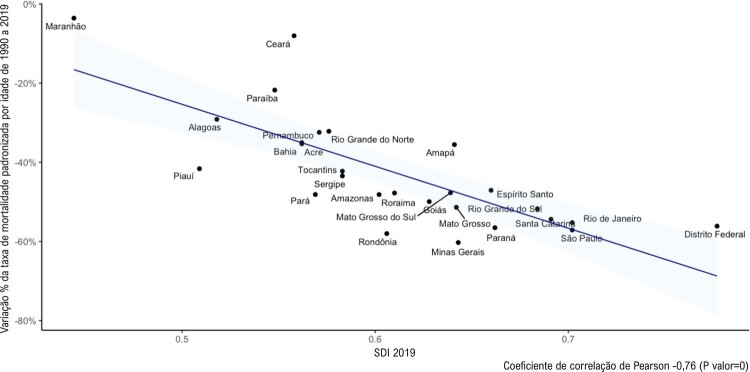
– Variação percentual da taxa de mortalidade padronizada por idade por doença cardiovascular atribuível aos baixos níveis de atividade física de 1990 a 2019, conforme o índice sociodemográfico (SDI) 2019 das unidades federativas brasileiras. Fonte: Dados derivados do Global Burden of Disease Collaborative Network. Global Burden of Disease (GBD) Cardiovascular Burden Estimates 1990 and 2021, Institute for Health Metrics and Evaluation, University of Washington. ^48^

**Tabela 12-5:** – Número de mortes e taxas de mortalidade específica padronizadas por idade por doenças cardiovasculares atribuíveis ao baixo nível de atividade física e percentual de variação das taxas por faixa etária. Brasil, 1990 e 2019

Localidade	1990		2019		Variação percentual (II 95%)
	Número (II 95%)	Taxa (II 95%)	Número (II 95%)	Taxa (II 95%)	
25-29 anos	70,1(15,4;160)	0,5(0,1;1,2)	49,6(9,1;114,2)	0,3(0,1;0,7)	-46,9%(-62,9;-32,5)
30-34 anos	144,2(29,7;331)	1,3(0,3;3)	106(18,8;243,1)	0,6(0,1;1,4)	-53,4%(-65,5;-36,5)
35-39 anos	255,9(53,8;581,2)	2,7(0,6;6,1)	213(39,8;479)	1,2(0,2;2,7)	-55%(-65,9;-39,6)
40-44 anos	363,9(81,8;823,1)	4,7(1,1;10,6)	340,6(68,9;754,6)	2,2(0,4;4,8)	-53,8%(-65,6;-40,3)
45-49 anos	572,8(147;1240,9)	9,3(2,4;20,2)	595,2(135,9;1311,9)	4,4(1;9,6)	-53,3%(-64,2;-39,8)
50-54 anos	794,9(214,2;1689,7)	15,4(4,1;32,7)	919,5(246,8;1976,8)	7,3(1,9;15,6)	-52,9%(-62,5;-38,1)
55-59 anos	960,9(285,4;1991,3)	22,5(6,7;46,6)	1269,9(367,5;2625,9)	11,3(3,3;23,4)	-49,7%(-59,1;-35,1)
60-64 anos	1380,4(441,6;2616,1)	38,6(12,4;73,2)	1871,2(624,5;3451,5)	20,2(6,8;37,3)	-47,6%(-56,7;-32,5)
65-69 anos	1633,8(586;3029,7)	61(21,9;113,2)	2468,2(965,3;4160,9)	34,3(13,4;57,8)	-43,8%(-52,4;-23,8)
70-74 anos	2083,2(884,8;3490,5)	110,1(46,8;184,5)	3348(1785;5150,2)	63,5(33,8;97,7)	-42,3%(-51;-19,9)
75-79 anos	2206,7(997,2;3495,6)	172,6(78;273,3)	3636,4(2179,2;5231,9)	101,9(61,1;146,6)	-41%(-50;-16,8)
80+ anos	6680,9(3508,8;10243,7)	630,9(331,4;967,4)	15411,3(9112,4;22314,1)	363(214,7;525,6)	-42,5%(-49,8;-26,4)

II: intervalo de incerteza. Estimativas do Estudo Global Burden of Disease 2021, Institute for Health Metrics and Evaluation da Universidade de Washington. ^48^

•A **Figura 12-5** mostra que, nas unidades federativas com SDI mais elevado (métrica do GBD que reflete as condições socioeconômicas de uma localidade e varia de 0 a 1, sendo 1 a melhor condição), houve maior redução da taxa de mortalidade padronizada por idade atribuível aos baixos níveis de atividade física de 1990 a 2019 (coeficiente de correlação de Pearson=0,76, p< 0,001).

•Ainda, em 1990, 6,4% do total de mortes por DCV foi atribuído ao baixo nível de atividade física, mas, em 2019, esse percentual aumentou para 7,6%.^404^

•Esse percentual é semelhante ao observado ao redor do mundo. Globalmente, estima-se que 7,6% das mortes cardiovasculares sejam atribuídas à inatividade física, sendo esse percentual maior nos países de alta renda (9,9%) do que nos de média (7,2%) e baixa (4,6%) renda.^405^

### Carga de Doença Atribuível

•De acordo com dados do GBD de 2019,^404^ tanto em 1990 quanto em 2019, o baixo nível de atividade física foi o nono fator de risco mais importante em relação à taxa de DALYs por DCV padronizada por idade, que foi de 434,5 (IC 95%, 193,2;747,3) por 100 mil habitantes em 1990 e de 233,1 (IC 95%, 118,2;375,7) por 100 mil habitantes em 2019, o que representa uma redução percentual de 46,4% (IC 95%, -84,6;-30,1) nessa taxa.

•Na comparação entre homens e mulheres, as taxas de DALYs padronizadas por idade foram semelhantes tanto em 1990 quanto em 2019.^404^ Entre os homens, a taxa de DALYs padronizada por idade diminuiu de 483,4 (IC 95%, 191,0;878,6) por 100 mil habitantes em 1990 para 275,7 (IC 95%, 127,9;478,4) por 100 mil habitantes em 2019, o que representa uma queda de 43,0% (IC 95%, -49,8;-27,6). Já entre as mulheres, essa redução foi de 49,1% (IC 95%, -54,9;-38,3), passando de 387,4 (IC 95%, 187,6;624,2) por 100 mil habitantes em 1990 para 197,2 (IC 95%, 110,7;303,3) por 100 mil habitantes em 2019.

•Globalmente, em 2019, as taxas de DALYs padronizadas por idade (/100 mil habitantes) por doença isquêmica do coração e por acidente vascular cerebral atribuídas à realização de <3000 MET-min/semana foram de 96,36 (IC 95%, 33,45;210,82) e 31,16 (IC 95%, 5,69;82,02), respectivamente.^406^

### Utilização e Custo da Atenção à Saúde

•A inatividade física tem um importante impacto nos sistemas de saúde do ponto de vista econômico. Bielemann *et al* .^407^ realizaram um estudo sobre o custo atribuível à inatividade física das internações no SUS por neoplasia maligna de mama e de cólon, doenças do aparelho circulatório, diabetes e osteoporose em 2013 de indivíduos com idade ≥40 anos. Do total de 974.641 internações devidas às doenças crônicas avaliadas, 15,0% foram atribuídas à inatividade física. Em relação à doença isquêmica do coração, a fração atribuída à inatividade física entre os homens variou de 11,3% na região Sul a 12,6% nas regiões Norte e Nordeste, enquanto, entre as mulheres, tal número variou de 16,1% na região Sul a 18,4% na região Norte. Já em relação à doença cerebrovascular, a fração atribuída a inatividade física variou, entre os homens, de 20,7% na região Sul a 22,8% nas regiões Norte e Nordeste e, entre as mulheres, de 7,6% na região Sul a 8,8% na região Norte.^407^

•Em relação ao custo para o SUS em 2013, do total de 1,8 bilhão de reais (quase 700 milhões de dólares), estimou-se que mais de 275 milhões de reais deveram-se às internações hospitalares atribuíveis à inatividade física pelas doenças citadas acima.^407^ Em todas as regiões analisadas e em ambos os sexos, a doença isquêmica do coração foi responsável pelo maior volume de custos totais e atribuíveis à inatividade física. Do custo total de 781 milhões de reais com internação hospitalar por doença isquêmica do coração e de quase 155 milhões de reais por doença cerebrovascular, 12% e 22%, respectivamente, foram atribuíveis à inatividade física em homens. Já em mulheres, do total de quase 500 milhões de reais com internação hospitalar por doença isquêmica do coração e de 161 milhões de reais por doença cerebrovascular, 17% e 8%, respectivamente, foram atribuíveis à inatividade física.^407^

•Um estudo retrospectivo realizado por Araújo *et al* .^408^ avaliou os custos diretos com saúde e os custos indiretos devidos à perda de produtividade de 278 residentes da cidade de Presidente Prudente (São Paulo) portadores de DCV e sua associação com o padrão habitual de prática de atividade física mensurada pelo questionário de Baecke. Indivíduos que reportaram absenteísmo no trabalho custaram ao sistema de saúde em um ano 187 dólares a mais do que indivíduos que não faltaram ao trabalho por razões relacionadas a saúde; além disso, estimou-se que 53 dólares poderiam ser economizados anualmente a cada aumento de uma unidade no escore de padrão de atividade física mensurado pelo questionário.

•Finalmente, de acordo com Santos *et al.* ,^409^ caso não haja redução na prevalência da inatividade física, estima-se um custo direto atribuível à inatividade física para o sistema de saúde brasileiro entre 2020 e 2023 de aproximadamente 126 milhões de dólares devido a doença arterial coronariana, de quase 4 bilhões de dólares devido a hipertensão arterial e mais de 470 milhões de dólares em decorrência de acidentes vasculares cerebrais.

### Impacto da pandemia da COVID-19 na atividade física

•A pandemia da COVID-19 teve um impacto significativo no aumento do tempo dedicado a comportamentos sedentários no Brasil, afetando consideravelmente a prática de atividade física em todo o país e em suas principais macrorregiões. Segundo o estudo de Silva *et al.* ^410^ realizado entre 24 de abril/2020 e 24 de maio/2020, foram constatadas elevadas prevalência e incidência de inatividade física e tempo excessivo de tela entre os brasileiros. Cerca de 40 mil adultos responderam a um questionário *online* , revelando uma prevalência de inatividade física de 70,4%. Essa prevalência foi ainda maior entre as mulheres (74,6%) e os mais jovens com idade entre 18 e 29 anos (78,2%).^411^

•Além disso, 31,4% dos participantes relataram passar 4 ou mais horas por dia em frente à TV, enquanto o uso de computador ou *tablet* por no mínimo 4 horas diárias teve uma prevalência de 37,9%.^410^ Novamente, a frequência de participantes que relataram assistir à TV por ≥4 horas/dia foi maior entre as mulheres (33,6%) e a frequência de participantes que relataram um número elevado de horas em frente ao computador/ *tablet* foi maior entre os jovens de 18 a 29 anos (58,6%).^411^

•Com base nos dados do VIGITEL de mais de 784 mil indivíduos, a tendência de aumento da prevalência de atividade física no lazer foi interrompida durante a pandemia. Em 2009, essa prevalência era de 29% e aumentou para 39% em 2019. No entanto, essa prevalência diminuiu para 36,8% em 2020 e para 36,7% em 2021. Entre os homens, houve uma queda de 3,6%, enquanto, entre as mulheres, a redução foi de 1,1% entre os anos de 2019 e 2021.^412^

•Segundo Schuch *et al.* ,^413^ brasileiros que estavam em autoisolamento durante a pandemia da COVID-19 reduziram o tempo dedicado à atividade física moderada/vigorosa em 65 min/dia em relação ao período pré-pandemia. Por outro lado, o tempo gasto em comportamentos sedentários aumentou em 42%, o que equivale a um acréscimo de 152 min/dia.

### Perspectivas

•A inatividade física é um dos principais fatores de risco modificáveis para doenças não transmissíveis, como acidente vascular cerebral, hipertensão arterial, diabetes tipo 2, doença coronariana, vários tipos de câncer, demência, depressão, assim como para mortalidade por todas as causas.^414^Um estilo de vida fisicamente ativo possui um papel inequívoco para que importantes benefícios cardiovasculares, tanto na prevenção primária quanto na secundária, sejam alcançados.^415^A relação curvilínea entre os volumes de atividade física e proteção cardiovascular^416^ permite que o simples fato de abandonar o comportamento sedentário e tornar-se fisicamente ativo promova reduções significativas no risco cardiovascular. Portanto, o comportamento sedentário deve ser desencorajado e a adoção de um estilo de vida fisicamente ativo deve ser estimulada.

•Com o objetivo de incentivar a prática de atividade física, ações nacionais foram implementadas, destacando-se o Programa Academia da Cidade e a Política Nacional de Promoção da Saúde, que contribuíram para o aumento da prática de atividade física dos seus usuários, impactando positivamente na sua saúde e incentivando, ainda, a melhoria dos espaços públicos destinados àquela prática.^417^Além disso, a inclusão da atividade física nos inquéritos populacionais brasileiros, como o VIGITEL, a PNS e a PENSE, permitiu um maior monitoramento da atividade física entre os brasileiros, auxiliando na revisão e elaboração de políticas públicas de saúde.

•No entanto, apesar da tendência à redução da inatividade física entre os brasileiros nos últimos anos, sua prevalência ainda permanece elevada, tendo, ainda, sido negativamente afetada pela pandemia da COVID-19. Dessa forma, faz-se necessário o incentivo contínuo a políticas nacionais que promovam hábitos de vida saudáveis, incluindo o aumento da prática regular de atividade física, visando amenizar as consequências socioeconômicas deletérias da inatividade física e do sedentarismo no Brasil.

**Tabela 7-1:** – Prevalência e intervalo de confiança de 95% (IC 95%) por hipertensão de acordo com características sociodemográficas. Pesquisa Nacional de Saúde, Brasil, 2019

Variáveis	Total		Feminino		Masculino	
	Prevalência (IC_**95%**_)	RP	Prevalência (IC_**95%**_)	RP	Prevalência (IC_**95%**_)	RP
**Total**	23,9 (23,5;24,4)		26,4 (25,7;27,2)		21,1 (20,4;21,8)	
**Grupo etário (anos)**						
18-24	2,3 (1,7;2,9)	1,0	2,1 (1,5;2,8)	1,0	2,5 (1,6;3,4)	1,0
25-39	7,3 (6,7;7,8)	3,2	7,2 (6,3;8,0)	3,5	7,4 (6,6;8,1)	3,0
40-59	27,2 (26,3;28,1)	11,5	29,5 (28,2;30,7)	13,3	24,6 (23,3;25,9)	9,9
≥ 60	55,0 (53,9;56,1)	22,1	59,4 (57,9;60,8)	24,7	49,3 (47,6;50,9)	19,7
**Escolaridade**						
Sem instrução/ fundamental incompleto	36,6 (35,7;37,5)	1,0	43,3 (42,0;44,6)	1,0	29,2 (28,0;30,3)	1,0
Fundamental completo/médio incompleto	20,4 (19,1;21,6)	1,0	24,7 (22,8;26,6)	0,9	16,2 (14,6;17,8)	1,0
Médio completo/ superior incompleto	15,4 (14,7;16,2)	0,8	15,7 (14,7;16,7)	0,8	15,1 (14,0;16,2)	1,0
Superior completo	18,2 (17,1;19,3)	0,7	16,3 (15,0;17,7)	0,6	20,7 (18,8;22,6)	1,0
**Raça/cor da pele autorreferida**						
Branca	24,4 (23,6;25,2)	1,0	26,0 (24,9;27,1)	1,0	22,5 (21,4;23,5)	1,0
Preta	25,8 (24,4;27,2)	1,2	30,2 (28,2;32,2)	1,2	20,9 (19,1;22,7)	1,1
Parda	22,9 (22,2;23,6)	1,1	25,7 (24,8;26,7)	1,1	19,7 (18,8;20,6)	1,0

RP: Razão de Prevalência. Fonte: Instituto Brasileiro de Geografia e Estatística – Pesquisa Nacional de Saúde 2019272 e Malta et al. ^263^

**Tabela 7-2:** – Distribuição percentual e intervalo de confiança de 95% (IC 95%) das características relacionadas à assistência de indivíduos com hipertensão autorreferida (n = 38.082), Pesquisa Nacional de Saúde, Brasil, 2019

Variáveis	%	IC 95%
**Último atendimento**		
Menos de 6 meses	57,8	56,6;59,0
6 meses a menos de 1 ano	14,4	13,6;15,2
1 ano a menos de 2 anos	9,4	8,8;10,1
2 anos a menos de 3 anos	2,7	2,3;3,2
3 anos ou mais	13,7	12,9;14,5
Nunca	1,9	1,6;2,3
**Local do último atendimento**		
Unidade básica de saúde	45,8	44,4;47,2
Consultório particular	28,8	27,5;30,1
Unidade de pronto-atendimento	9,6	8,9;10,5
Ambulatório de hospital público	7,1	6,5;7,8
Policlínica pública	3,6	3,1;4,2
Pronto-atendimento privado	1,7	1,4;2,0
Domicílio	1,4	1,2;1,6
Farmácia	0,9	0,7;1,2
Outro serviço	1,0	0,7;1,4
**Orientações**		
Práticas integrativas	7,4	6,8;8,1
Não beber em excesso	66,5	65,1;67,8
Não fumar	67,2	65,8;68,6
Praticar atividade física regular	81,7	80,7;82,7
Manter peso adequado	84,4	83,4;85,4
Fazer acompanhamento regular	85,2	84,2;86,2
Adotar alimentação saudável	87,2	86,3;88,2
Ingerir menos sal	87,8	86,7;88,8
Exames e encaminhamentos		
E **xame de sangue**	79,9	78,8;80,9
Exame de urina	69,9	68,5;71,2
Eletrocardiograma	64,5	63,2;65,8
Teste de esforço	33,6	32,2;34,9
Encaminhamento a especialista	25,0	23,7;26,2

Fonte: Instituto Brasileiro de Geografia e Estatística – Pesquisa Nacional de Saúde 2019 ^272^ e Malta et al. ^263^

**Tabela 7-3:** – Indicadores de assistência e acesso a serviços de saúde em adultos com hipertensão arterial de acordo com o sexo. Pesquisa Nacional de Saúde, Brasil, 2019

Indicadores	Total			Sexo				

	**%**	**IC 95%**	**Masculino (A)**		**Feminino (B)**		**RP ajustada (B/A)**	**IC 95%**
			**%**	**IC 95%**	**%**	**IC 95%**		
Medicação prescrita	**95,4**	**94,9; 5,8**	**938**	**92,9; 4,5**	96,5	95,9; 7,0	1,02	1,01; 1,03
Uso de medicação nas 2 semanas anteriores	**86,9**	**86,1; 7,7**	831	**81,7; 4,5**	89,6	88,6; 0,5	1,06	1,04; 1,08
Pelo menos uma medicação do Programa Farmácia Popular	**45,1**	**43,6; 6,5**	437	**41,7; 5,7**	46,0	44,3; 7,7	1,03	0,98; 1,08
Recebeu assistência médica para hipertensão no último ano	**72,2**	**71,1; 3,3**	68,9	**67,3; 0,5**	74,5	73,1; 5,8	1,07	1,04; 1,11
Última consulta médica foi em UBS	**45,8**	**44,4; 7,2**	41,8	**39,7; 3,9**	48,5	46,9; 0,2	1,11	1,05; 1,17
Consulta com o mesmo médico das consultas anteriores	**51,9**	**50,6; 3,3**	52,3	**50,3; 4,3**	51,7	49,8; 3,5	0,99	0,94; 1,05
Consulta com especialista	**49,0**	**47,6; 0,4**	49,5	47,4; 1,5	48,6	46,8; 0,5	1,01	0,96; 1,07
Encaminhamento para especialista	**79,1**	**76,8; 1,1**	78,6	75,0; 1,8	79,3	76,5; 2,0	1,01	0,96; 1,06
Hospitalização por hipertensão ou complicação	**15,0**	**14,2; 5,9**	13,6	12,4; 5,0	15,9	14,8; 7,1	1,14	1,01; 1,28
Limitação grave ou muito grave para as atividades da vida diária	**3,0**	**2,7; 3,4**	3,1	2,5; 3,7	3,0	2,6; 3,5	0,93	0,73; 1,17

IC: Intervalo de confiança; RP: Razão de Prevalência; UBS: Unidade Básica de Saúde. A categoria de referência usada foi ‘masculino’. Fonte: Malta et al. ^261^

**Tabela 7-4:** – Indicadores de assistência de saúde em adultos com hipertensão arterial de acordo com o grupo etário. Pesquisa Nacional de Saúde, Brasil, 2019

Indicadores					Grupo etário (anos)					
	18-29 (A)		30-59 (B)		≥ 60 (C)		RP ajustada B/A	IC95%	RP ajustada (C/A)	IC95%
	%	IC95%	%	IC95%	%	IC95%				
Medicação prescrita	68,4	61,6; 74,5	93,3	92,4; 94,1	98,8	98,4; 99,0	1,35	1,23; 1,49	1,43	1,30; 1,57
Uso de medicação nas 2 semanas anteriores	39,3	31,9; 47,2	81,9	80,6; 83,2	94,3	93,6; 94,9	2,05	1,69; 2,49	2,34	1,93; 2,85
Pelo menos uma medicação do Programa Farmácia Popular	21,1	14,0; 30,6	45,7	43,3; 48,0	45,1	43,5; 46,7	2,16	1,46; 3,20	2,08	1,41; 3,08
Recebeu assistência médica para hipertensão no último ano	62,9	54,9; 70,3	71,1	69,5; 72,8	73,7	72,3; 75,0	1,11	0,98; 1,25	1,13	1,00; 1,27
Última consulta médica foi em UBS	47,1	38,6; 55,7	45,5	43,6; 47,4	46,0	44,3; 47,8	0,95	0,80; 1,14	0,92	0,76; 1,10
Consulta com o mesmo médico das consultas anteriores	37,6	29,4; 46,6	49,6	47,6; 51,5	54,9	53,2; 56,7	1,29	1,02; 1,62	1,42	1,13; 1,79
Consulta com especialista	64,2	48,3; 77,8	75,4	71,7; 78,8	83,4	80,6; 85,9	1,17	0,93; 1,47	1,31	1,04; 1,64
Encaminhamento para especialista	35,8	28,0; 44,5	46,4	44,3; 48,5	52,1	50,4; 53,9	1,27	1,02; 1,58	1,47	1,18; 1,83
Hospitalização por hipertensão ou complicação	13,0	8,3; 19,9	14,1	12,9; 15,5	16,0	14,8; 17,2	1,02	0,65; 1,58	1,07	0,69; 1,68
Limitação grave ou muito grave para as atividades da vida diária	2,9	1,2; 6,8	3,1	2,6; 3,8	2,9	2,5; 3,4	0,92	0,37; 2,26	0,70	0,29; 1,72

IC: Intervalo de confiança; RP: Razão de Prevalência; UBS: Unidade Básica de Saúde. A categoria de referência usada foi ‘grupo etário de 18-29 anos’. Fonte: Malta et al. ^261^

**Table 7-5:** – Healthcare indicators of adults with arterial hypertension according to ethnicity. National Health Survey, Brazil, 2019

Indicator	Ethnicity									
	White (A)		Black (B)		Mixed-race (C)		Adjusted PR (B/A)	95%CI	Adjusted PR (C/A)	95%CI
	%	95%CI	%	95%CI	%	95%CI				
Prescribed medication	96.2	95.5; 96.8	95.0	93.5; 96.2	94.5	93.7; 95.2	1.00	0.98; 1.01	1.00	0.98; 1.01
Use of medication in the previous two weeks	88.5	87.2; 89.6	86.7	84.6; 88.6	85.3	84.1; 86.5	0.99	0.97; 1.01	1.00	0.98; 1.03
At least one medication from the “Popular Pharmacy Program”	44.8	42.9; 46.8	46.3	43.0; 49.7	44.8	42.8; 46.9	0.94	0.89; 1.00	0.97	0.89; 1.06
Received medical care for hypertension within the last year	71.5	69.9; 73.1	74.7	71.9; 77.3	72.1	70.5; 73.7	1.01	0.98; 1.04	1.05	1.01; 1.09
Had the last physician appointment at UBS	39.4	37.3; 41.6	51.9	48.6; 55.2	50.7	48.9; 52.5	1.01	0.96; 1.07	1.06	0.98; 1.14
Had an appointment with the same physician as in the previous appointments	56.7	54.6; 58.7	48.8	45.4; 52.2	48.2	46.3; 50.2	0.92	0.87; 0.97	0.93	0.85; 1.00
Appointments with a specialist	54.6	52.5; 56.6	47.9	44.6; 51.3	43.4	41.5; 45.3	0.96	0.91; 1.01	0.94	0.87; 1.02
Referral for a specialist	82.9	79.7; 85.7	75.4	68.9; 80.9	76.7	73.3; 79.7	0.92	0.88; 0.97	1.02	0.94; 1.09
Hospitalization for hypertension or a complication	13.2	11.9; 14.6	15.7	13.5; 18.2	16.8	15.4; 18.3	1.20	1.05; 1.38	1.14	0.95; 1.36
Intense or very intense degree of limitation of daily living activities	2.2	1.8; 2.7	3.5	2.7; 4.6	3.6	3.1; 4.3	1.37	1.06; 1.76	1.38	0.99; 1.94

CI: Confidence Interval; PR: Prevalence Ratio; UBS: Basic Healthcare Unit. The reference category used was ‘white’. Source: Malta et al. ^261^

**Tabela 8-2:** – Prevalência de colesterol total ≥ 200 mg/dl de acordo com sexo, grupo etário, nível educacional, cor da pele e região do Brasil, PNS 2014-2015

	Total			Homens			Mulheres		
	%	IC 95%	p	%	IC 95%	p	%	IC 95%	p
**Total**	**32,7**	**31,5 - 34,1**		**30,1**	**28,2 - 32,1**		**35,1**	**33,4 - 36,8**	**< 0,001**
**Grupo etário (anos)**									
18 - 29	17,9	15,7 - 20,4	< 0,001	13,9	11,2 - 17,4	< 0,001	21,9	18,7 - 25,5	< 0,001
30 - 44	31,0	28,7 - 33,4		34,9	31,2 - 38,8		27,6	24,9 - 30,5	
45 - 59	43,4	40,8 - 46,0		39,4	35,7 - 43,4		47,0	43,5 - 50,5	
≥ 60	41,9	39,1 - 44,8		33,5	29,5 - 37,9		48,4	44,7 - 52,2	
**Escolaridade (anos)**									
0 - 8	37,1	35,2 - 39,1	< 0,001	31,6	28,9 - 34,5	0,237	42,2	39,6 - 44,8	< 0,001
9 - 11	28,6	25,5 - 32,0		26,6	22,2 - 31,6		30,6	26,4 - 35,2	
≥ 12	30,4	28,4 - 32,5		30,0	26,9 - 33,3		30,8	28,3 - 33,4	
**Cor da pele**									
Branca	33,9	31,9 - 36,0	0,146	30,8	27,8 - 33,9	0,669	36,6	33,9 - 39,4	0,196
Negra	33,2	29,0 - 37,6		30,0	23,9 - 37,0		36,0	30,5 - 41,8	
Parda	31,5	29,8 - 33,3		29,5	26,9 - 32,4		33,4	31,1 - 35,7	
Outras	23,3	14,8 - 34,6		19,6	9,7 - 35,4		25,8	14,2 - 42,2	
**Região**									
Norte	32,5	30,4 - 34,6	0,195	31,0	27,9 - 34,3	0,376	33,9	31,2 - 36,7	0,291
Nordeste	34,0	32,3 - 35,8		30,2	27,7 - 33,0		37,4	35,1 - 39,8	
Sudeste	31,5	29,1 - 34,1		28,7	25,1 - 32,6		34,1	30,9 - 37,4	
Sul	34,7	31,7 - 37,8		33,4	28,9 - 38,3		35,8	32,0 - 39,8	
Centro-Oeste	31,7	28,7 - 34,8		30,1	25,7 - 34,9		33,0	29,1 - 37,2	

Fonte: Malta et al. 2019. ^275^

**Tabela 8-3:** – Prevalência de níveis baixos de HDL-colesterol (< 40 mg/dl) de acordo com sexo, grupo etário, nível educacional, cor da pele e região do Brasil, PNS 2014-2015

	Total			Homens			Mulheres		
	%	IC 95%	P	%	IC 95%	p	%	IC 95%	p
**Total**	31,8	30,5 - 33,1		42,8	40,6 - 45,0		22,0	20,6 - 23,5	< 0,001
**Grupo etário (anos)**									
18 - 29	29,1	26,2 - 32,2	0,070	39,7	34,9 - 44,7	0,159	18,7	15,9 - 21,9	0,046
30 - 44	31,8	29,4 - 34,2		41,8	37,9 - 45,7		23,0	20,4 - 25,9	
45 - 59	34,1	31,6 - 36,6		44,8	40,9 - 48,8		24,3	21,5 - 27,4	
≥ 60	32,4	29,8 - 35,2		46,5	42,1 - 51,1		21,5	18,7 - 24,6	
**Escolaridade (anos)**									
0 - 8	33,7	31,8 - 35,7	< 0,001	43,3	40,2 - 46,4	0,006	24,9	22,8 - 27,2	< 0,001
9 - 11	38,5	34,9 - 42,2		50,0	44,3 - 55,6		27,0	22,9 - 31,5	
≥ 12	27,8	25,9 - 29,9		39,6	36,2 - 43,2		18,1	16,1 - 20,3	
**Cor da pele**									
Branca	31,0	29,0 - 33,1	0,072	43,0	39,7 - 46,5	0,586	20,6	18,4 - 23,0	0,006
Negra	28,5	24,3 - 33,2		41,8	34,5 - 49,4		16,6	12,6 - 21,6	
Parda	33,5	31,7 - 35,4		43,0	40,0 - 46,1		24,8	22,8 - 27,0	
Outras	24,7	15,8 - 36,5		27,7	15,1 - 45,2		22,7	11,6 - 39,5	
**Região do país**									
Norte	36,6	34,4 - 38,8	< 0,001	47,2	43,7 - 50,7	0,036	26,7	24,2 - 29,4	< 0,001
Nordeste	34,8	33,0 - 36,6		44,3	41,4 - 47,2		26,4	24,3 - 28,6	
Sudeste	30,8	28,3 - 33,4		43,1	38,9 - 47,3		20,0	17,4 - 22,9	
Sul	26,1	23,3 - 29,0		36,3	31,6 - 41,2		16,8	14,1 - 20,0	
Centro-Oeste	34,3	31,1 - 37,6		45,0	39,8 - 50,3		24,7	21,2 - 28,6	

Fonte: Malta et al. 2019. ^275^

**Tabela 8-4:** – Prevalência de níveis altos de LDL-colesterol (> 130 mg/dl) de acordo com sexo, grupo etário, nível educacional, cor da pele e região do Brasil, PNS 2014-2015

	Total			Homens			Mulheres		
	%	IC 95%	p	%	IC 95%	p	%	IC 95%	p
**Total**	18,6	17,5 - 19,7		17,1	15,6 - 18,8		19,9	18,5 - 21,3	0,012
**Grupo etário (anos)**									
18 - 29	8,8	7,2 - 10,7	< 0,001	6,6	4,8 - 9,0	< 0,001	11,0	8,7 - 14,0	< 0,001
30 - 44	17,5	15,7 - 19,5		20,2	17,3 - 23,6		15,2	13,0 - 17,6	
45 - 59	25,6	23,3 - 27,9		23,2	20,0 - 26,7		27,7	24,7 - 30,9	
≥ 60	24,5	22,2 - 27,0		19,5	16,3 - 23,2		28,4	25,1 - 31,9	
**Escolaridade (anos)**									
0 - 8	21,5	20,0 - 23,2	< 0,001	17,8	15,7 - 20,1	0,525	24,9	22,8 - 27,2	< 0,001
9 - 11	16,8	14,3 - 19,7		15,2	11,8 - 19,3		18,5	15,0 - 22,6	
≥ 12	16,7	15,1 - 18,4		17,2	14,8 - 20,0		16,2	14,2 - 18,4	
**Cor da pele**									
Branca	20,1	18,5 - 21,9	0,009	18,8	16,4 - 21,4	0,131	21,3	19,1 - 23,8	0,095
Negra	16,6	13,6 - 20,2		15,2	10,9 - 20,8		17,9	13,9 - 22,7	
Parda	17,4	16,1 - 18,8		15,9	13,9 - 18,1		18,8	17,0 - 20,7	
Outras	10,1	6,0 - 16,6		8,6	3,6 - 19,1		11,2	5,7 - 20,7	
**Região do país**									
Norte	16,2	14,7 - 17,9	0,136	15,5	13,2 - 18,1	0,355	17,0	14,9 - 19,2	0,195
Nordeste	19,8	18,4 - 21,3		17,5	15,5 - 19,8		21,9	19,9 - 23,9	
Sudeste	17,9	16,0 - 19,9		16,1	13,4 - 19,3		19,4	16,8 - 22,2	
Sul	20,0	17,6 - 22,6		19,8	16,2 - 24,0		20,1	17,1 - 23,5	
Centro-Oeste	17,8	15,4 - 20,4		17,8	14,3 - 21,9		17,8	14,8 - 21,3	

Fonte: Malta et al. 2019. ^275^

**Table 10-3:** – Prevalence of second-hand smoke at work, by sex, according to age and years of schooling

	Total			Sexo					
				Masculino			Feminino		
	Total	IC 95%		Total	IC 95%		Total	IC 95%	
		Limite inferior	Limite superior		Limite inferior	Limite superior		Limite inferior	Limite superior
**Total (%) **	5,4	4,6	6,3	8,1	6,6	9,5	3,2	2,4	4,0
**Idade (anos) **									
18-24	4,6	2,9	6,4	5,3	2,7	8,0	3,9	1,5	6,2
25-34	7,6	5,0	10,2	10,0	5,6	14,5	5,5	2,6	8,3
35-44	5,4	3,8	6,9	9,2	6,0	12,5	2,3	1,4	3,2
45-54	6,4	4,9	8,0	9,9	7,1	12,8	3,3	2,0	4,6
55-64	4,1	3,0	5,2	6,6	4,3	9,0	2,1	1,3	2,9
≥ 65	1,9	1,2	2,6	3,6	2,0	5,1	0,7	0,2	1,1
**Escolaridade (anos) **									
0-8	5,9	4,2	7,7	11,0	7,5	14,5	1,5	0,9	2,0
9-11	6,3	5,0	7,7	8,6	6,3	10,9	4,2	2,9	5,6
≥ 12	4,0	2,7	5,3	4,7	2,9	6,6	3,4	1,6	5,2

Fonte: VIGITEL Brasil 2021. ^252^

**Tabela10-4:** – Porcentagem de estudantes com idade de 13-17 anos que já experimentaram cigarros eletrônicos, por sexo, tipo de escola, de acordo com grupo etário e principais regiões brasileiras

	Total			Sexo						Tipo de escola					
				Masculino			Feminino			Pública			Privada		
	Total	IC 95%		Total	IC 95%		Total	IC 95%		Total	IC 95%		Total	IC 95%	
		Limite inferior	Limite superior		Limite inferior	Limite superior		Limite inferior	Limite superior		Limite inferior	Limite superior		Limite inferior	Limite superior
**13 a 17 anos **															
Brasil	16,8	16,2	17,4	19,1	18,3	19,9	14,6	13,9	15,3	16,6	15,9	17,3	18,0	17,3	18,8
Norte	12,3	11,1	13,4	14,9	13,2	16,5	10,0	8,7	11,2	11,9	10,6	13,2	16,6	15,3	17,9
Nordeste	10,8	10,0	11,5	12,7	11,6	13,8	8,9	8,1	9,7	10,3	9,5	11,1	13,8	13,0	14,7
Sudeste	19,6	18,4	20,8	21,6	19,9	23,2	17,6	16,2	19,0	19,9	18,5	21,3	17,9	16,6	19,3
Sul	21,0	19,3	22,7	23,2	21,1	25,4	18,7	16,9	20,6	20,7	18,8	22,6	23,2	21,4	24,9
Centro-Oeste	23,7	22,6	24,9	27,4	25,8	28,9	20,2	18,8	21,7	23,6	22,3	25,0	24,3	22,7	25,9
**13 a 15 anos **															
Brasil	13,6	13,0	14,2	14,8	13,9	15,7	12,5	11,7	13,2	13,5	12,8	14,2	14,2	13,4	15,0
Norte	10,5	9,3	11,8	12,4	10,5	14,4	8,8	7,6	10,1	10,3	9,0	11,7	12,9	11,6	14,1
Nordeste	8,5	7,7	9,3	9,9	8,9	11,0	7,2	6,3	8,0	8,2	7,3	9,1	10,2	9,4	10,9
Sudeste	15,7	14,5	16,9	16,4	14,5	18,2	15,0	13,5	16,5	16,0	14,5	17,4	14,6	13,2	16,0
Sul	16,6	15,0	18,3	17,6	15,6	19,5	15,7	13,6	17,9	16,4	14,5	18,3	17,8	16,0	19,6
Centro-Oeste	20,4	19,0	21,7	22,1	20,3	23,9	18,8	17,1	20,4	20,5	18,9	22,0	19,9	18,2	21,6
**16 a 17 anos **															
Brasil	22,7	21,7	23,7	27,0	25,7	28,3	18,5	17,3	19,8	22,1	21,0	23,2	26,9	25,1	28,6
Norte	15,3	13,5	17,2	19,0	16,4	21,7	12,0	10,0	14,0	14,7	12,7	16,7	24,9	22,3	27,6
Nordeste	14,9	13,8	16,1	17,7	15,8	19,7	12,2	10,8	13,5	14,0	12,7	15,3	23,1	21,1	25,1
Sudeste	26,7	24,5	28,8	31,7	29,3	34,1	22,0	19,2	24,8	26,9	24,4	29,3	25,7	22,5	28,9
Sul	28,9	25,9	31,8	32,9	29,0	36,8	24,4	21,3	27,6	28,2	24,9	31,5	33,8	30,7	36,9
Centro-Oeste	30,1	28,1	32,1	37,1	34,4	39,8	23,1	20,8	25,4	29,5	27,3	31,7	34,3	31,3	37,3

Fonte: PeNSE 2019. ^353^

**Figura 10-1:**
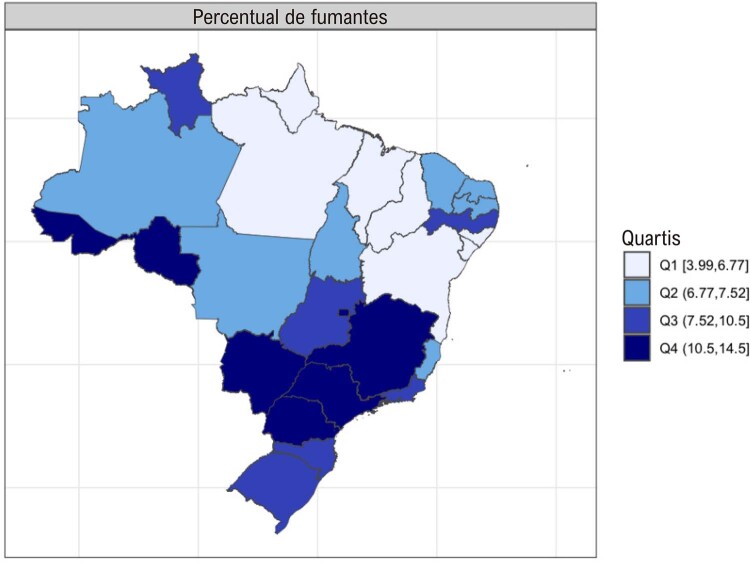
– Prevalência de adultos fumantes atuais (≥ 18 anos) de acordo com as unidades federativas brasileiras e os quartis de prevalência. Dados do VIGITEL Brasil 2021. ^252^

**Figura 10-2:**
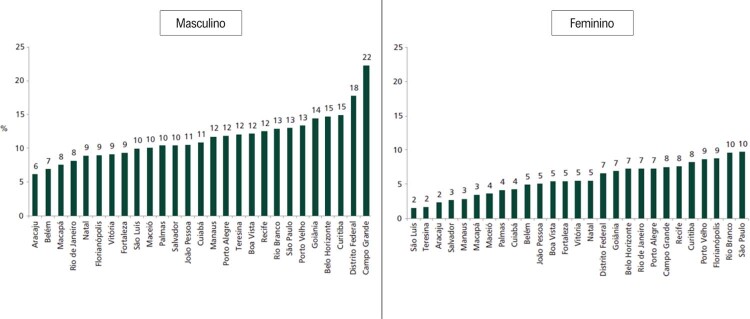
– Prevalência de adultos fumantes atualmente (≥ 18 anos) de acordo com as capitais dos estados brasileiros e Distrito Federal, por sexo. Dados do VIGITEL Brasil 2021. ^252^

**Figura 11-3:**
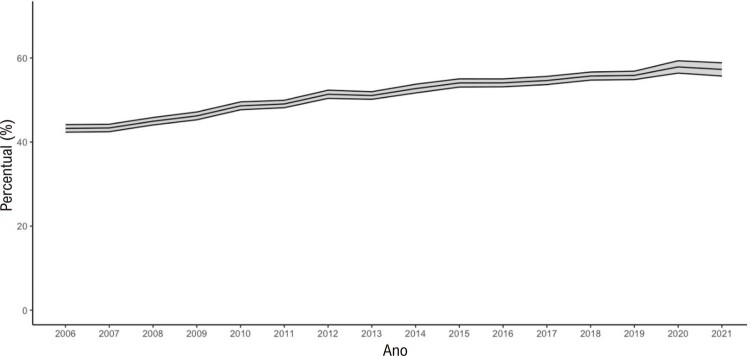
– Porcentagem de adultos com sobrepeso (≥ 18 anos), Brasil, 2006-2021. Dados do VIGITEL Brasil 2021. ^252^

**Figura 11-4:**
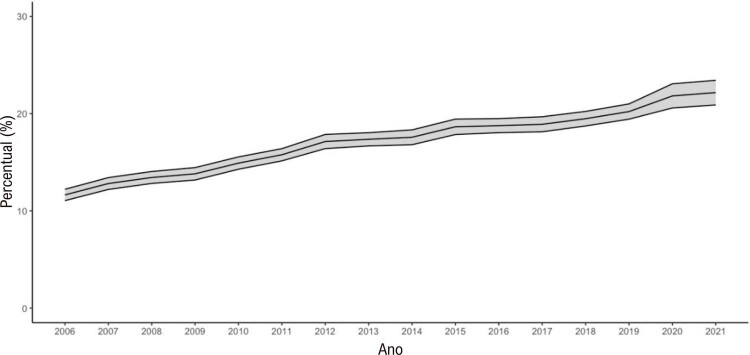
– Porcentagem de adultos com obesidade (≥ 18 anos), Brasil, 2006-2021. Dados do VIGITEL Brasil 2021. ^252^

**Figura 12-2:**
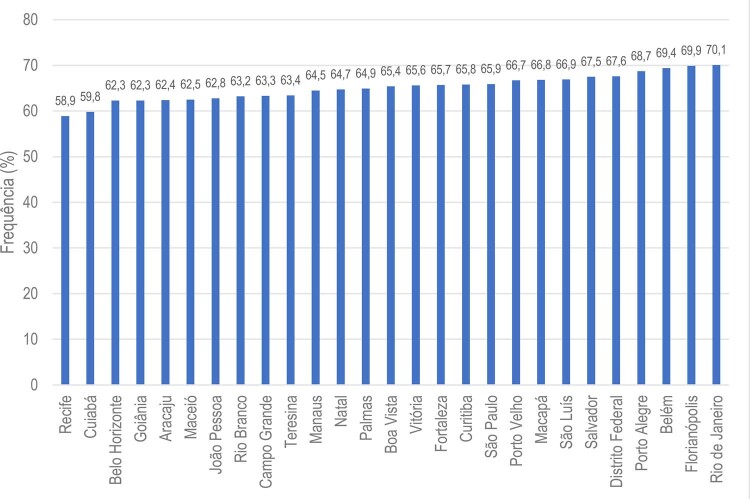
– Frequência de adultos com idade ≥18 anos que despendem diariamente ≥3 horas do tempo livre assistindo à televisão ou usando computador, tablet ou celular nas capitais dos estados brasileiros e Distrito Federal de acordo com dados do Vigitel 2021.2. ^252^

## References

[1] Malta DC, Passos VMA, Machado ÍE, Souza MFM, Ribeiro ALP (2020). The GBD Brazil Network: Better Information for Health Policy Decision-Making in Brazil. Popul Health Metr.

[2] Oliveira GMM, Brant LCC, Polanczyk CA, Biolo A, Nascimento BR, Malta DC, Souza MFM, Soares GP, Xavier GF, Machline-Carrion MJ, Bittencourt MS, Pontes OM, Silvestre OM, Teixeira RA, Sampaio RO, Gaziano TA, Roth GA, Ribeiro ALP (2020). Cardiovascular Statistics - Brazil 2020. Arq Bras Cardiol.

[3] Tsao CW, Aday AW, Almarzooq ZI, Anderson CAM, Arora P, Avery CL, Baker-Smith CM, Beaton AZ, Boehme AK, Buxton AE, Commodore-Mensah Y, Elkind MSV, Evenson KR, Eze-Nliam C, Fugar S, Generoso G, Heard DG, Hiremath S, Ho JE, Kalani R, Kazi DS, Ko D, Levine DA, Liu J, Ma J, Magnani JW, Michos ED, Mussolino ME, Navaneethan SD, Parikh NI, Poudel R, Rezk-Hanna M, Roth GA, Shah NS, St-Onge MP, Thacker EL, Virani SS, Voeks JH, Wang NY, Wong ND, Wong SS, Yaffe K, Martin SS, American Heart Association Council on Epidemiology and Prevention Statistics Committee and Stroke Statistics Subcommittee (2023). Heart Disease and Stroke Statistics-2023 Update: A Report from the American Heart Association. Circulation.

[4] Oliveira GMM, Brant LCC, Polanczyk CA, Malta DC, Biolo A, Nascimento BR, Souza MFM, Lorenzo AR, Fagundes AAP, Schaan BD, Castilho FM, Cesena FHY, Soares GP, Xavier GF, Barreto JAS, Passaglia LG, Pinto MM, Machline-Carrion MJ, Bittencourt MS, Pontes OM, Villela PB, Teixeira RA, Sampaio RO, Gaziano TA, Perel P, Roth GA, Ribeiro ALP (2022). Cardiovascular Statistics - Brazil 2021. Arq Bras Cardiol.

[5] Johns Hopkins University and Medicine (2023). Johns Hopkins Coronavirus Resource Center.

[6] Strabelli TMV, Uip DE (2020). COVID-19 and the Heart. Arq Bras Cardiol.

[7] Borba MGS, Val FFA, Sampaio VS, Alexandre MAA, Melo GC, Brito M, Mourão MPG, Brito-Sousa JD, Baía-da-Silva D, Guerra MVF, Hajjar LA, Pinto RC, Balieiro AAS, Pacheco AGF, Santos JDO, Naveca FG, Xavier MS, Siqueira AM, Schwarzbold A, Croda J, Nogueira ML, Romero GAS, Bassat Q, Fontes CJ, Albuquerque BC, Daniel-Ribeiro CT, Monteiro WM, Lacerda MVG, CloroCovid-19 Team (2020). Effect of High vs Low Doses of Chloroquine Diphosphate as Adjunctive Therapy for Patients Hospitalized with Severe Acute Respiratory Syndrome Coronavirus 2 (SARS-CoV-2) Infection: A Randomized Clinical Trial. JAMA Netw Open.

[8] Brant LCC, Nascimento BR, Teixeira RA, Lopes MACQ, Malta DC, Oliveira GMM, Ribeiro ALP (2020). Excess of Cardiovascular Deaths During the COVID-19 Pandemic in Brazilian Capital Cities. Heart.

[9] Azevedo RB, Botelho BG, Hollanda JVG, Ferreira LVL, Andrade LZJ, Oei SSML, Mello TS, Muxfeldt ES (2021). Covid-19 and the Cardiovascular System: A Comprehensive Review. J Hum Hypertens.

[10] Sliwa K, Singh K, Raspail L, Ojji D, Lam CSP, Thienemann F, Ge J, Banerjee A, Newby LK, Ribeiro ALP, Gidding S, Pinto F, Perel P, Prabhakaran D (2021). The World Heart Federation Global Study on COVID-19 and Cardiovascular Disease. Glob Heart.

[11] Marcolino MS, Ziegelmann PK, Souza-Silva MVR, Nascimento IJB, Oliveira LM, Monteiro LS, Sales TLS, Ruschel KB, Martins KPMP, Etges APBS, Molina I, Polanczyk CA, Brazilian COVID-19 Registry Investigators (2021). Clinical Characteristics and Outcomes of Patients Hospitalized with COVID-19 in Brazil: Results from the Brazilian COVID-19 Registry. Int J Infect Dis.

[12] Kim HW, Jenista ER, Wendell DC, Azevedo CF, Campbell MJ, Darty SN, Parker MA, Kim RJ (2021). Patients with Acute Myocarditis Following mRNA COVID-19 Vaccination. JAMA Cardiol.

[13] França EB, Ishitani LH, Abreu DMX, Teixeira RA, Corrêa PRL, Jesus EDS, Marinho MAD, Bahia TV, Bierrenbach AL, Setel P, Marinho F (2022). Measuring Misclassification of Covid-19 as Garbage Codes: Results of Investigating 1,365 Deaths and Implications for Vital Statistics in Brazil. PLOS Glob Public Health.

[14] Nadarajah R, Wu J, Hurdus B, Asma S, Bhatt DL, Biondi-Zoccai G, Mehta LS, Ram CVS, Ribeiro ALP, van Spall HGC, Deanfield JE, Lüscher TF, Mamas M, Gale CP (2022). The Collateral Damage of COVID-19 to Cardiovascular Services: A Meta-Analysis. Eur Heart J.

[15] Brant LCC, Pinheiro PC, Passaglia LG, Souza MFM, Malta DC, Banerjee A, Ribeiro ALP, Nascimento BR (2023). Cardiovascular Mortality in Brazil During the COVID-19 Pandemic: A Comparison between Underlying and Multiple Causes of Death. Public Health.

[16] COVID-19 Cumulative Infection Collaborators (2022). Estimating Global, Regional, and National Daily and Cumulative Infections with SARS-CoV-2 Through Nov 14, 2021: A Statistical Analysis. Lancet.

[17] COVID-19 Excess Mortality Collaborators (2022). Estimating Excess Mortality Due to the COVID-19 Pandemic: A Systematic Analysis of COVID-19-Related Mortality, 2020-21. Lancet.

[18] Castro MC, Massuda A, Almeida G, Menezes-Filho NA, Andrade MV, Noronha KVMS, Rocha R, Macinko J, Hone T, Tasca R, Giovanella L, Malik AM, Werneck H, Fachini LA, Atun R (2019). Brazil's Unified Health System: The First 30 Years and Prospects for the Future. Lancet.

[19] Ribeiro ALP, Duncan BB, Brant LC, Lotufo PA, Mill JG, Barreto SM (2016). Cardiovascular Health in Brazil: Trends and Perspectives. Circulation.

[20] Marinho MF, Torrens A, Teixeira R, Brant LCC, Malta DC, Nascimento BR, Ribeiro ALP, Delaney R, de Paula PDCB, Setel P, Sampaio JM, Nogales-Vasconcelos AM (2022). Racial Disparity in Excess Mortality in Brazil During COVID-19 Times. Eur J Public Health.

[21] Ribeiro ALP, Oliveira GMM (2019). Toward a Patient-Centered, Data-Driven Cardiology. Arq Bras Cardiol.

[22] Brasil, Ministério da Saúde, Fundação Nacional de Saúde (2011). Manual de Instruções para o Preenchimento da Declaração de Óbito.

[23] Jorge MH, Laurenti R, Gotlieb SL (2007). Quality Analysis of Brazilian Vital Statistics: The Experience of Implementing the SIM and SINASC Systems. Cien Saude Colet.

[24] França E, Abreu DX, Rao C, Lopez AD (2008). Evaluation of Cause-of-Death Statistics for Brazil, 2002-2004. Int J Epidemiol.

[25] Malta DC, Teixeira R, Oliveira GMM, Ribeiro ALP (2020). Cardiovascular Disease Mortality According to the Brazilian Information System on Mortality and the Global Burden of Disease Study Estimates in Brazil, 2000-2017. Arq Bras Cardiol.

[26] Escosteguy CC, Portela MC, Medronho RA, Vasconcellos MT (2002). The Brazilian Hospital Information System and the acute Myocardial Infarction Hospital Care. Rev Saude Publica.

[27] Rocha TAH, Silva NCD, Amaral PVM, Barbosa ACQ, Vissoci JRN, Thomaz EBAF, Queiroz RCS, Harris M, Facchini LA (2018). Geolocation of Hospitalizations Registered on the Brazilian National Health System's Hospital Information System: A Solution Based on the R Statistical Software. Epidemiol Serv Saude.

[28] Stopa SR, Szwarcwald CL, Oliveira MM, Gouvea ECDP, Vieira MLFP, Freitas MPS, Sardinha LMV, Macário EM (2020). National Health Survey 2019: History, Methods and Perspectives. Epidemiol Serv Saude.

[29] Malta DC, Stopa SR, Szwarcwald CL, Gomes NL, Silva JB, Reis AA (2015). Surveillance and Monitoring of Major Chronic Diseases in Brazil - National Health Survey, 2013. Rev Bras Epidemiol.

[30] França EB, Passos VMA, Malta DC, Duncan BB, Ribeiro ALP, Guimarães MDC, Abreu DMX, Vasconcelos AMN, Carneiro M, Teixeira R, Camargos P, Melo APS, Queiroz BL, Schmidt MI, Ishitani L, Ladeira RM, Morais-Neto OL, Bustamante-Teixeira MT, Guerra MR, Bensenor I, Lotufo P, Mooney M, Naghavi M (2017). Cause-Specific Mortality for 249 Causes in Brazil and States During 1990-2015: A Systematic Analysis for the Global Burden of Disease Study 2015. Popul Health Metr.

[31] GBD 2016 Brazil Collaborators (2018). Burden of Disease in Brazil, 1990-2016: A Systematic Subnational Analysis for the Global Burden of Disease Study 2016. Lancet.

[32] GBD 2019 Viewpoint Collaborators (2020). Five Insights from the Global Burden of Disease Study 2019. Lancet.

[33] GBD 2019 Demographics Collaborators (2020). Global Age-Sex-Specific Fertility, Mortality, Healthy Life Expectancy (HALE), and Population Estimates in 204 Countries and Territories, 1950-2019: A Comprehensive Demographic Analysis for the Global Burden of Disease Study 2019. Lancet.

[34] GBD 2019 Diseases and Injuries Collaborators (2020). Global Burden of 369 Diseases and Injuries in 204 Countries and Territories, 1990-2019: A Systematic Analysis for the Global Burden of Disease Study 2019. Lancet.

[35] GBD 2019 Risk Factors Collaborators (2020). Global Burden of 87 Risk Factors in 204 Countries and Territories, 1990-2019: A Systematic Analysis for the Global Burden of Disease Study 2019. Lancet.

[36] O’Gara PT, Kushner FG, Ascheim DD, Casey DE, Chung MK, Lemos JA (2013). 2013 ACCF/AHA Guideline for the Management of STElevation Myocardial Infarction: A Report of the American College of Cardiology Foundation/American Heart Association Task Force on Practice Guidelines. Circulation.

[37] Roth GA, Vaduganathan M, Mensah GA (2022). Impact of the COVID-19 Pandemic on Cardiovascular Health in 2020: JACC State-of-the-Art Review. J Am Coll Cardiol.

[38] Naghavi M, Makela S, Foreman K, O'Brien J, Pourmalek F, Lozano R (2010). Algorithms for Enhancing Public Health Utility of National Causes-Of-Death Data. Popul Health Metr.

[39] Shemilt I, Thomas J, Morciano M (2010). A Web-based Tool for Adjusting Costs to a Specific Target Currency and Price Year. Evidence and Policy is Evid Policy.

[40] World Health Organization (2013). Global Action Plan for the Prevention and Control of NCDs 2013-2020.

[41] Malta DC, Moura L, Prado RR, Escalante JC, Schmidt MI, Duncan BB (2014). Chronic Non-communicable Disease Mortality in Brazil and its Regions, 2000-2011. Epidemiol Serv Saúde.

[42] Duncan BB, Chor D, Aquino EM, Bensenor IM, Mill JG, Schmidt MI, Lotufo PA, Vigo A, Barreto SM (2012). Chronic Non-Communicable Diseases in Brazil: Priorities for Disease Management and Research. Rev Saude Publica.

[43] Malta DC, França E, Abreu DMX, Perillo RD, Salmen MC, Teixeira RA, Passos V, Souza MFM, Mooney M, Naghavi M (2017). Mortality Due to Noncommunicable Diseases in Brazil, 1990 to 2015, According to Estimates from the Global Burden of Disease study. Sao Paulo Med J.

[44] Nascimento BR, Brant LCC, Oliveira GMM, Malachias MVB, Reis GMA, Teixeira RA, Malta DC, França E, Souza MFM, Roth GA, Ribeiro ALP (2018). Cardiovascular Disease Epidemiology in Portuguese-Speaking Countries: Data from the Global Burden of Disease, 1990 to 2016. Arq Bras Cardiol.

[45] Malta DC, Bernal RT, Souza MF, Szwarcwald CL, Lima MG, Barros MB (2016). Social Inequalities in the Prevalence of Self-Reported Chronic Non-Communicable Diseases in Brazil: National Health Survey 2013. Int J Equity Health.

[46] Brant LCC, Nascimento BR, Passos VMA, Duncan BB, Bensenõr IJM, Malta DC, Souza MFM, Ishitani LH, França E, Oliveira MS, Mooney M, Naghavi M, Roth G, Ribeiro ALP (2017). Variations and Particularities in Cardiovascular Disease Mortality in Brazil and Brazilian States in 1990 and 2015: Estimates from the Global Burden of Disease. Rev Bras Epidemiol.

[47] Hasani WSR, Muhamad NA, Hanis TM, Maamor NH, Wee CX, Omar MA, Ganapathy SS, Abdul Karim Z, Musa KI (2023). The Burden of Premature Mortality from Cardiovascular Diseases: A Systematic Review of Years of Life Lost. PLoS One.

[48] Global Burden of Disease Collaborative Network (2022). Global Burden of Disease (GBD) Cardiovascular Burden Estimates 1990 and 2021.

[49] Brasil, Ministério da Saúde (2023). Sistema de Informações Hospitalares do Sistema Único de Saúde (SIH/SUS).

[50] Gonçalves RPF, Haikal DS, Freitas MIF, Machado ÍE, Malta DC (2019). Self-Reported Medical Diagnosis of Heart Disease and Associated Risk Factors: National Health Survey. Rev Bras Epidemiol.

[51] Schmidt MI, Duncan BB, Mill JG, Lotufo PA, Chor D, Barreto SM, Aquino EM, Passos VM, Matos SM, Molina MC, Carvalho MS, Bensenor IM (2015). Cohort Profile: Longitudinal Study of Adult Health (ELSA-Brasil). Int J Epidemiol.

[52] Massa KHC, Duarte YAO, Chiavegatto ADP (2019). Analysis of the Prevalence of Cardiovascular Diseases and Associated Factors among the Elderly, 2000-2010. Cien Saude Colet.

[53] Motta ACSV, Bousquet-Santos K, Motoki IHL, Andrade JML (2023). Prevalence of Ideal Cardiovascular Health in the Brazilian Adult Population - National Health Survey 2019. Epidemiol Serv Saude.

[54] Manderson L, Jewett S (2023). Risk, Lifestyle and Non-Communicable Diseases of Poverty. Global Health.

[55] Yeh EJ, Grigolon RB, Rodrigues SR, A Bueno AP (2023). Systematic Literature Review and Meta-Analysis of Cardiovascular Risk Factor Management in Selected Asian Countries. J Comp Eff Res.

[56] Mansur AP, Favarato D (2016). Mortality due to Cardiovascular Diseases in Women and Men in the Five Brazilian Regions, 1980-2012. Arq Bras Cardiol.

[57] Baptista E, Queiroz B, Rigotti J (2018). Decomposition of Mortality Rates from Cardiovascular Disease in the Adult Population: A Study for Brazilian Micro-Regions between 1996 and 2015. Rev Bras Estud Popul.

[58] Rasella D, Harhay MO, Pamponet ML, Aquino R, Barreto ML (2014). Impact of Primary Health Care on Mortality from Heart and Cerebrovascular Diseases in Brazil: A Nationwide Analysis of Longitudinal Data. BMJ.

[59] Lotufo PA (2019). Trends in Cardiovascular Diseases and Heart Disease Death Rates among Adults Aged 45-64: Brazil, 2000-2017. Sao Paulo Med J.

[60] Lotufo PA, Fernandes TG, Bando DH, Alencar AP, Benseñor IM (2013). Income and Heart Disease Mortality Trends in Sao Paulo, Brazil, 1996 to 2010. Int J Cardiol.

[61] Villela PB, Klein CH, de Oliveira GMM (2019). Socioeconomic Factors and Mortality Due to Cerebrovascular and Hypertensive Disease in Brazil. Rev Port Cardiol.

[62] Soares GP, Klein CH, Silva NA, Oliveira GM (2016). Progression of Mortality due to Diseases of the Circulatory System and Human Development Index in Rio de Janeiro Municipalities. Arq Bras Cardiol.

[63] Soares GP, Brum JD, Oliveira GM, Klein CH, Silva NAS (2013). Evolution of Socioeconomic Indicators and Cardiovascular Mortality in Three Brazilian States. Arq Bras Cardiol.

[64] Baptista E, Queiroz B (2019). The Relation between Cardiovascular Mortality and Development: Study for Small Areas in Brazil, 2001-2015. Demographic Res.

[65] Silveira IH, Oliveira BFA, Cortes TR, Junger WL (2019). The Effect of Ambient Temperature on Cardiovascular Mortality in 27 Brazilian Cities. Sci Total Environ.

[66] Brant LCC, Pinheiro PC, Ribeiro ALP, Machado IE, Correa PRL, Santos MR, de Souza MFM, Malta DC, Passos VMA (2022). Cardiovascular Mortality During the COVID-19 Pandemics in a Large Brazilian City: A Comprehensive Analysis. Glob Heart.

[67] Araújo JM, Rodrigues REA, Arruda ACP, Ferreira FELL, Lima RLFC, Vianna RPT, Moreira LVL, Silva JM, Moreira PVL (2022). The Direct and Indirect Costs of Cardiovascular Diseases in Brazil. PLoS One.

[68] World Health Organization (2013). WHO's Global Health Estimates.

[69] Abe IM, Lotufo PA, Goulart AC, Benseñor IM (2011). Stroke Prevalence in a Poor Neighbourhood of São Paulo, Brazil: Applying a Stroke Symptom Questionnaire. Int J Stroke.

[70] Goulart AC, Bustos IR, Abe IM, Pereira AC, Fedeli LM, Benseñor IM, Lotufo PA (2010). A Stepwise Approach to Stroke Surveillance in Brazil: the EMMA (Estudo de Mortalidade e Morbidade do Acidente Vascular Cerebral) Study. Int J Stroke.

[71] Fernandes TG, Benseñor IM, Goulart AC, Tavares BM, Alencar AP, Santos IS, Lotufo PA (2014). Stroke in the Rain Forest: Prevalence in a Ribeirinha Community and an Urban Population in the Brazilian Amazon. Neuroepidemiology.

[72] Bensenor IM, Goulart AC, Szwarcwald CL, Vieira ML, Malta DC, Lotufo PA (2015). Prevalence of Stroke and Associated Disability in Brazil: National Health Survey-2013. Arq Neuropsiquiatr.

[73] Minelli C, Cabral NL, Ujikawa LT, Borsetti FA, Langhi Chiozzini EM, Reis GC, Borin LA, Carvalho CC (2020). Trends in the Incidence and Mortality of Stroke in Matão, Brazil: The Matão Preventing Stroke (MAPS) Study. Neuroepidemiology.

[74] Fernandes TG, Bando DH, Alencar AP, Benseñor IM, Lotufo PA (2015). Income Inequalities and Stroke Mortality Trends in Sao Paulo, Brazil, 1996-2011. Int J Stroke.

[75] Guimarães RM, Andrade SS, Machado EL, Bahia CA, Oliveira MM, Jacques FV (2015). Regional Differences in Cardiovascular Mortality Transition in Brazil, 1980 to 2012. Rev Panam Salud Publica.

[76] Minelli C, Fen LF, Minelli DP (2007). Stroke Incidence, Prognosis, 30-Day, and 1-Year Case Fatality Rates in Matão, Brazil: A Population-Based Prospective Study. Stroke.

[77] Ducci RD, Tessaro CL, Fontes DP, Fraga GS, Cirino RHD, Lopes FDN, Zetola VHF, Lange MC (2022). Stroke-Related Mortality Analysis in Paraná, Brazil, Over 10 Years. Arq Neuropsiquiatr.

[78] Reis MF, Chaoubah A (2023). The Burden of Stroke in the Southeast Region of Brazil in 2019: An Estimate Based on Secondary Data from the Brazilian United Health System. Int J Cardiovasc Sci.

[79] Schmidt MHS, Selau CM, Soares PS, Franchi EF, Piber VD, Quatrin LB (2019). Stroke and Different Limitations: An Interdisciplinary Analysis. Arq Cienc Saude UNIPAR.

[80] Torres JL, Andrade FB, Lima-Costa MF, Nascimento LR (2022). Walking Speed and Home Adaptations are Associated with Independence after Stroke: A Population-Based Prevalence Study. Cien Saude Colet.

[81] Dantas LF, Marchesi JF, Peres IT, Hamacher S, Bozza FA, Neira RAQ (2019). Public Hospitalizations for Stroke in Brazil from 2009 to 2016. PLoS One.

[82] Santos EFS, Antunes JLF (2020). Factors Associated with Lack of Hospital Care in Deaths from Cerebrovascular Disease, São Paulo, Brazil: A Comparative Analysis of 1996-1998 and 2013-2015. Cad Saude Publica.

[83] Lange MC, Braga GP, Nóvak EM, Harger R, Felippe MJDB, Canever M, Dall'Asta I, Rauen J, Bazan R, Zetola V (2017). Key Performance Indicators for Stroke from the Ministry of Health of Brazil: Benchmarking and Indicator Parameters. Arq Neuropsiquiatr.

[84] Machline-Carrion MJ, Santucci EV, Damiani LP, Bahit MC, Málaga G, Pontes OM, Martins SCO, Zétola VF, Normilio-Silva K, Freitas GF, Gorgulho A, Salles A, Silva BGP, Santos JY, Jesuíno IA, Bueno PRT, Cavalcanti AB, Guimarães HP, Xian Y, Bettger JP, Lopes RD, Peterson ED, Berwanger O, BRIDGE-Stroke Investigators (2019). Effect of a Quality Improvement Intervention on Adherence to Therapies for Patients with Acute Ischemic Stroke and Transient Ischemic Attack: A Cluster Randomized Clinical Trial. JAMA Neurol.

[85] Martins SO, Mont'Alverne F, Rebello LC, Abud DG, Silva GS, Lima FO, Parente BSM, Nakiri GS, Faria MB, Frudit ME, Carvalho JJF, Waihrich E, Fiorot JA, Cardoso FB, Hidalgo RCT, Zétola VF, Carvalho FM, Souza AC, Dias FA, Bandeira D, Miranda Alves M, Wagner MB, Carbonera LA, Oliveira-Filho J, Bezerra DC, Liebeskind DS, Broderick J, Molina CA, Fogolin Passos JE, Saver JL, Pontes OM, Nogueira RG, RESILIENT Investigators (2020). Thrombectomy for Stroke in the Public Health Care System of Brazil. N Engl J Med.

[86] Araújo DV, Teich V, Passos RB, Martins SC (2010). Analysis of the Cost-Effectiveness of Thrombolysis with Alteplase in Stroke. Arq Bras Cardiol.

[87] Souza AC, Martins SO, Polanczyk CA, Araújo DV, Etges APB, Zanotto BS, Neyeloff JL, Carbonera LA, Chaves MLF, Carvalho JJF, Rebello LC, Abud DG, Cabral LS, Lima FO, Mont'Alverne F, Sc Magalhães P, Diegoli H, Safanelli J, Salvetti TAS, Parente BSM, Frudit ME, Silva GS, Pontes OM Neto, Nogueira RG (2021). Cost-Effectiveness of Mechanical Thrombectomy for Acute Ischemic Stroke in Brazil: Results from the RESILIENT Trial. Int J Stroke.

[88] Avezum A, Oliveira GBF, Lanas F, Lopez-Jaramillo P, Diaz R, Miranda JJ, Seron P, Camacho-Lopez PA, Orlandini A, Bernabe-Ortiz A, Cordeiro Mattos A, Islam S, Rangarajan S, Teo K, Yusuf S (2017). Secondary CV Prevention in South America in a Community Setting: The PURE Study. Glob Heart.

[89] Vinereanu D, Al-Khalidi HR, Rao MP, He W, Lopes RD, Bahit CM, Ciobanu AO, Fox KA, Pokorney SD, Xian Y, Jiang J, Kamath DY, Berwanger O, Tajer C, Huo Y, Xavier D, Granger CB (2017). Regional Differences in Presentation and Antithrombotic Treatment of Patients with Atrial Fibrillation: Baseline Characteristics from a Clustered Randomized Trial to IMProve Treatment with AntiCoagulanTs in Patients with Atrial Fibrillation (IMPACT-AF). Am Heart J.

[90] Silva DAS, Ribeiro ALP, Marinho F, Naghavi M, Malta DC (2022). Physical Activity to Prevent Stroke Mortality in Brazil (1990-2019). Rev Soc Bras Med Trop.

[91] Machline-Carrion MJ, Girotto AN, Nieri J, Pereira PM, Monfardini F, Forestiero F, Raupp P, Roveda F, Santo K, Berwanger O, Santos RD (2023). Assessing Statins Use in a Real-World Primary Care Digital Strategy: A Cross-Sectional Analysis of a Population-Wide Digital Health Approach. Lancet Reg Health Am.

[92] Martins SCO, Sacks C, Hacke W, Brainin M, Figueiredo FA, Pontes OM, Germain PML, Marinho MF, Wiegering AH, McGhie DV, Cruz-Flores S, Ameriso SF, Villareal WMC, Durán JC, Passos JEF, Nogueira RG, Carvalho JJF, Silva GS, Moro CHC, Oliveira-Filho J, Gagliardi R, Sousa EDG, Soares FF, Campos KP, Teixeira PFP, Gonçalves IP, Carquin IRS, Collazos MM, Romero GEP, Figueredo JIM, Barboza MA, López MÁC, Góngora-Rivera F, Cantú-Brito C, Novarro-Escudero N, Blanco MÁV, Morvil CAAO, Bareiro ABO, Rojas GM, Flores A, Hancco-Saavedra JA, Jimenez VP, Argomedo CA, Kadota LR, Crosa R, Cuervo DLM, Souza AC, Carbonera LA, Guzmán TFA, Maldonado N, Cabral NL, Anderson C, Lindsay P, Hennis A, Feigin VL (2019). Priorities to Reduce the Burden of Stroke in Latin American Countries. Lancet Neurol.

[93] Pontes OM, Silva GS, Feitosa MR, Figueiredo NL, Fiorot JA, Rocha TN, Massaro AR, Leite JP (2008). Stroke Awareness in Brazil: Alarming Results in a Community-Based Study. Stroke.

[94] Falavigna A, Teles AR, Vedana VM, Kleber FD, Mosena G, Velho MC, Mazzocchin T, Silva RC, Lucena LF, Santin JT, Roth F (2009). Awareness of Stroke Risk Factors and Warning Signs in Southern Brazil. Arq Neuropsiquiatr.

[95] Rissardo JP, Caprara ALF, Prado ALC (2018). Stroke Literacy in a South Brazilian City: A Community Based Survey. J Stroke Cerebrovasc Dis.

[96] Calderaro M, Salles IC, Gouvêa GB, Monteiro VS, Mansur AP, Shinohara HNI, Aikawa P, Umeda IIK, Semeraro F, Carmona MJC, Böttiger BW, Nakagawa NK (2022). The Lack of Knowledge on Acute Stroke in Brazil: A Cross-Sectional Study with Children, Adolescents, and Adults from Public Schools. Clinics.

[97] Rodrigues MS, Santana LFE, Castro APF, Coelho KKA, Guimarães MP, Gomes OV, Schwingel PA, Cerqueira RB, Guimarães MD, Moura JC (2022). Awareness Towards Stroke among High School Students in Brazil: A Cross-Sectional Study. Sao Paulo Med J.

[98] Berwanger O, Machline-Carrion MJ (2022). Digital Health-Enabled Clinical Trials in Stroke: Ready for Prime Time?. Stroke.

[99] Valêncio RFZ, Souza JT, Winckler FC, Modolo GP, Ferreira NC, Bazan SGZ, Lange MC, Freitas CCM, Paiva SAR, Oliveira RC, Luvizutto GJ, Bazan R (2022). Semi-Automated Data Collection from Electronic Health Records in a Stroke Unit in Brazil. Arq Neuropsiquiatr.

[100] Birck MG, Goulart AC, Lotufo PA, Benseñor IM (2019). Secondary Prevention of Coronary Heart Disease: A Cross-Sectional Analysis on the Brazilian Longitudinal Study of Adult Health (ELSA-Brasil). Sao Paulo Med J.

[101] Malta DC, Pinheiro PC, Vasconcelos NM, Stopa SR, Vieira MLFP, Lotufo PA (2021). Prevalence of Angina Pectoris and Associated Factors in the Adult Population of Brazil: National Survey of Health, 2019. Rev Bras Epidemiol.

[102] Silva PGMBE, Berwanger O, Santos ESD, Sousa ACS, Cavalcante MA, Andrade PB, Neuenschwander FC, Vargas H, Guimarães JI, Andrade J, Paola AAV, Malachias MVB, Mattos LAPE, Precoma DB, Bacal F, Dutra OP (2020). One Year Follow-Up Assessment of Patients Included in the Brazilian Registry of Acute Coronary Syndromes (ACCEPT). Arq Bras Cardiol.

[103] Franken M, Giugliano RP, Goodman SG, Baracioli LM, Godoy LC, Furtado RHM, Lima FG, Nicolau JC (2020). Performance of Acute Coronary Syndrome Approaches in Brazil: A Report from the BRACE (Brazilian Registry in Acute Coronary SyndromEs). Eur Heart J Qual Care Clin Outcomes.

[104] Nicolau JC, Franken M, Lotufo PA, Carvalho AC, Marin JÁ, Lima FG, Dutra O, Knobel E, Oliveira CC, Timerman S, Stefanini E (2012). Use of Demonstrably Effective Therapies in the Treatment of Acute Coronary Syndromes: Comparison between Different Brazilian Regions. Analysis of the Brazilian Registry on Acute Coronary Syndromes (BRACE). Arq Bras Cardiol.

[105] Berwanger O, Guimarães HP, Laranjeira LN, Cavalcanti AB, Kodama AA, Zazula AD, Santucci EV, Victor E, Tenuta M, Carvalho V, Mira VL, Pieper KS, Weber B, Mota LH, Peterson ED, Lopes RD, Bridge-Acs Investigators (2012). Effect of a Multifaceted Intervention on Use of Evidence-Based Therapies in Patients with Acute Coronary Syndromes in Brazil: The BRIDGE-ACS Randomized Trial. JAMA.

[106] Pereira AC, Gomez LM, Bittencourt MS, Staniak HL, Sharovsky R, Foppa M, Blaha MJ, Bensenor IM, Lotufo PA (2016). Age, Gender, and Race-Based Coronary Artery Calcium Score Percentiles in the Brazilian Longitudinal Study of Adult Health (ELSA-Brasil). Clin Cardiol.

[107] Ferreira LCM, Nogueira MC, Carvalho MS, Teixeira MTB (2020). Mortality Due to Acute Myocardial Infarction in Brazil from 1996 to 2016: 21 Years of Disparities in Brazilian Regions. Arq Bras Cardiol.

[108] Moreira PVL, Arruda ADCP, Ferreira SS, Ferreira FELL, Lima RLFC, Toledo Vianna RP, Araújo JM, Rodrigues REA, Silva JM, O'Flaherty M (2021). Coronary Heart Disease and Stroke Mortality Trends in Brazil 2000-2018. PLoS One.

[109] Vieira RCP, Marcolino MS, Silva LGSE, Pereira DN, Nascimento BR, Jorge AO, Ribeiro ALP (2022). Assessment of the Impact of the Implementation of a Pre-Hospital Ambulance System on Acute Myocardial Infarction Mortality in a Developing Country. Arq Bras Cardiol.

[110] Oliveira JC, Almeida-Santos MA, Cunha-Oliveira J, Oliveira LCS, Barreto IDC, Lima TCRM, Arcelino LAM, Prado LFA, Silveira FS, Nascimento TAS, Ferreira EJP, Barreto RV, Moraes EV, Mendonça JT, Sousa ACS, Barreto-Filho JA, VICTIM Register Investigators (2019). Disparities in Access and Mortality of Patients With ST-Segment-Elevation Myocardial Infarction Using the Brazilian Public Healthcare System: VICTIM Register. J Am Heart Assoc.

[111] Piegas LS, Avezum A, Guimarães HP, Muniz AJ, Reis HJ, Santos ES, Knobel M, Souza R (2013). Acute Coronary Syndrome Behavior: Results of a Brazilian Registry. Arq Bras Cardiol.

[112] Soeiro AM, Silva PGMBE, Roque EAC, Bossa AS, Biselli B, Leal TCAT, Soeiro MCFA, Pitta FG, Serrano CV, Oliveira MT (2018). Prognostic Differences between Men and Women with Acute Coronary Syndrome. Data from a Brazilian Registry. Arq Bras Cardiol.

[113] Marino BC, Marcolino MS, Reis RS, França AL, Passos PF, Lemos TR, Antunes IO, Ferreira CG, Antunes AP, Ribeiro ALP (2016). Epidemiological Profile and Quality Indicators in Patients with Acute Coronary Syndrome in Northern Minas Gerais - Minas Telecardio 2 Project. Arq Bras Cardiol.

[114] Bianco HT, Povoa R, Izar MC, Alves CMR, Barbosa AHP, Bombig MTN, Gonçalves I, Luna B, Aguirre AC, Moraes PIM, Almeida D, Moreira FT, Povoa FF, Stefanini E, Caixeta AM, Bacchin AS, Moisés VA, Fonseca FAH (2022). Pharmaco-invasive Strategy in Myocardial Infarction: Descriptive Analysis, Presentation of Ischemic Symptoms and Mortality Predictors. Arq Bras Cardiol.

[115] Passaglia LG, Cerqueira MLR, Pires MM, Chagas LV, Érika CTC, Rodrigues ENO, Diniz FMM, Ferreira DF, Nogueira MR, Braga GT, Taniguchi FP, Ribeiro ALP (2023). Cardiovascular Statistics from the Good Practices in Cardiology Program - Data from a Brazilian Tertiary Public Hospital. Arq Bras Cardiol.

[116] Barreto J, Matos LCV, Quinaglia JC, Sposito AC, Carvalho LS (2021). The Impact of Low Income on Long-Term Mortality of Myocardial Infarction Patients: Results from the Brazilian Heart Study. Curr Med Res Opin.

[117] Bruno TC, Bittencourt MS, Quidim AVL, Santos I, Lotufo P, Bensenor I, Goulart A (2021). The Prognosis of Coronary Artery Disease in a Brazilian Community Hospital: Findings from the ERICO Study. Arq Bras Cardiol.

[118] Carvalho LSF, Alexim G, Nogueira ACC, Fernandez MD, Rezende TB, Avila S, Reis RTB, Soares AAM, Sposito AC (2023). The Framing of Time-Dependent Machine Learning Models Improves Risk Estimation among Young Individuals with Acute Coronary Syndromes. Sci Rep.

[119] Hueb W, Rezende PC, Gersh BJ, Soares PR, Favarato D, Lima EG, Garzillo CL, Jatene FB, Ramires JAF, Kalil R (2019). Ten-Year Follow-Up of Off-Pump and On-Pump Multivessel Coronary Artery Bypass Grafting: MASS III. Angiology.

[120] Scudeler TL, Farkouh ME, Hueb W, Rezende PC, Campolina AG, Martins EB, Godoy LC, Soares PR, Ramires JAF, Kalil R (2022). Coronary Atherosclerotic Burden Assessed by SYNTAX Scores and Outcomes in Surgical, Percutaneous or Medical Strategies: A Retrospective Cohort Study. BMJ Open.

[121] Carvalho FPC, Hueb W, Lima EG, Rezende PC, Linhares JPP, Garcia RMR, Soares PR, Ramires JAF, Kalil R (2023). Cardiovascular Events in Patients with Coronary Artery Disease with and without Myocardial Ischemia: Long-Term Follow-Up. Am Heart J.

[122] Lodi-Junqueira L, Silva JL, Ferreira LR, Gonçalves HL, Athayde GR, Gomes TO, Borges JC, Nascimento BR, Lemos PA, Ribeiro ALP (2015). In-Hospital Mortality Risk Prediction after Percutaneous Coronary Interventions: Validating and Updating the Toronto Score in Brazil. Catheter Cardiovasc Interv.

[123] Paula LJC, Lemos PA, Medeiros CR, Marin-Neto JA, Figueiredo GL, Polanczyk CA, Wainstein MV, Ribeiro ALP, Lodi-Junqueira L, Oliveira FRA, Sarmento-Leite R, Mattos LA, Cantarelli MJC, Brito FS, Carvalho ACC, Barbosa MR (2010). Construction and validation of an Integrated Percutaneous Coronary Intervention Data System in Brazil (ICP-BR Registry): Clinical Profile of the First 1,249 Patients Included. Rev Bras Cardiol. Invasiva.

[124] Paez RP, Hossne NA, Santo JADE, Berwanger O, Santos RHN, Kalil RAK, Jatene FB, Cavalcanti AB, Zilli AC, Bettiati LC, Figueira FAMDS, D'Azevedo SSP, Soares MJF, Fernandes MP, Ardito RV, Bogdan RAB, Campagnucci VP, Nakasako D, Rodrigues CG, Rodrigues AB, Cascudo MM, Atik FA, Lima EB, Nina VJDS, Heluy RA, Azeredo LG, Henrique OS, Mendonça JT, Silva KKOG, Pandolfo M, Lima JD, Faria RM, Santos JGD, Coelho GHB, Pereira SN, Senger R, Buffolo E, Caputi GM, Oliveira JAB, Gomes WJ, BYPASS Registry Study Group (2019). Coronary Artery Bypass Surgery in Brazil: Analysis of the National Reality Through the BYPASS Registry. Braz J Cardiovasc Surg.

[125] Gonzales-Tamayo L, Campos CM, Lisboa LAF, Dallan LAO, Jatene FB, Mejia OAV (2019). Choosing the Best Mortality Predictor for Isolated CABG in Complex Coronary Artery Disease Patients: Performance Comparison of Sts, Euroscore II, and Syntax Score II. J Am Coll Cardiol.

[126] Paredes RAM, Borgomoni GB, Micalay AKP, Camacho JCA, Dallan LRP, Lisboa LAF, Dallan LAO, Mejia OAV, Grupo de Estudos REPLICCAR (2023). Immediate Results after Multiple Arterial Grafts in Coronary Artery Bypass Graft Surgery in the São Paulo State: Cross Cohort Study. Arq Bras Cardiol.

[127] Rösler Á, Constantin G, Nectoux P, Holz BS, Letti E, Sales M, Lucchese-Lobato F, Lucchese F (2022). Thirty-day Outcomes of On-Pump and Off-Pump Coronary Artery Bypass Grafting: An Analysis of a Brazilian Sample by Propensity Score Matching. Braz J Cardiovasc Surg.

[128] Buffolo E, Branco JN, Gerola LR, Aguiar LF, Teles CA, Palma JH, Catani R (2006). Off-pump Myocardial Revascularization: Critical Analysis of 23 Years' Experience in 3,866 Patients. Ann Thorac Surg.

[129] Lisboa LA, Moreira LF, Mejia OV, Dallan LA, Pomerantzeff PM, Costa R, Puig LB, Jatene FB, Marcial MB, Stolf NA (2010). Evolution of Cardiovascular Surgery at the Instituto do Coração: Analysis of 71,305 Surgeries. Arq Bras Cardiol.

[130] Santos CA, Oliveira MA, Brandi AC, Botelho PH, Brandi JC, Santos MA, Godoy MF, Braile DM (2014). Risk Factors for Mortality of Patients Undergoing Coronary Artery Bypass Graft Surgery. Rev Bras Cir Cardiovasc.

[131] Lobato PHM, Vieira FM, Nunes MBG, Galucio VAQL, Barreto EL (2019). Clinical Course of Patients Undergoing Myocardial Revascularization Surgery in a Public Cardiology Referral Hospital in Pará, Brazil. Int J Cardiovasc Sci.

[132] Stevens B, Pezzullo L, Verdian L, Tomlinson J, George A, Bacal F (2018). The Economic Burden of Heart Conditions in Brazil. Arq Bras Cardiol.

[133] Azambuja MI, Foppa M, Maranhão MF, Achutti AC (2008). Economic Burden of Severe Cardiovascular Diseases in Brazil: An Estimate Based on Secondary Data. Arq Bras Cardiol.

[134] Alexim GA, Rocha LF, Dobri GP, Rosa ADS, Reis RTB, Nogueira ACC, Soares AASM, Sposito AC, Paula AP, Carvalho LSF (2022). Clinical and Economic Impact of Coronary Artery Bypass Graft and Percutaneous Coronary Intervention in Young Individuals with Acute Coronary Syndromes and Multivessel Disease: A Real-World Comparison in a Middle-Income Country. Front Cardiovasc Med.

[135] Santos IS, Goulart AC, Brandão RM, Santos RC, Bittencourt MS, Sitnik D, Pereira AC, Pastore CA, Samesima N, Lotufo PA, Bensenor IM (2015). One-year Mortality after an Acute Coronary Event and its Clinical Predictors: The ERICO Study. Arq Bras Cardiol.

[136] Lana MLL, Beaton AZ, Brant LCC, Bozzi ICRS, Magalhães O, Castro LRA, Silva FCT, Silva JLP, Ribeiro ALP, Nascimento BR (2017). Factors Associated with Compliance to AHA/ACC Performance Measures in a Myocardial Infarction System of Care in Brazil. Int J Qual Health Care.

[137] Marino BCA, Ribeiro ALP, Alkmim MB, Antunes AP, Boersma E, Marcolino MS (2016). Coordinated Regional Care of Myocardial Infarction in a Rural Area in Brazil: Minas Telecardio Project 2. Eur Heart J Qual Care Clin Outcomes.

[138] Marcolino MS, Brant LCC, Araujo JG, Nascimento BR, Castro LR, Martins P, Lodi-Junqueira L, Ribeiro ALP (2013). Implementation of the Myocardial Infarction System of Care in City of Belo Horizonte, Brazil. Arq Bras Cardiol.

[139] Filgueiras NM, Feitosa GS, Solla DJF, Argôlo FC, Guimarães PO, Paiva IM, Carvalho LGM, Teixeira LS, Rios MNO, Câmara SF, Novais VO, Barbosa LS, Ballalai CS, Lúcia CV, Granger CB, Newby LK, Lopes RD (2018). Implementation of a Regional Network for ST-Segment-Elevation Myocardial Infarction (STEMI) Care and 30-Day Mortality in a Low- to Middle-Income City in Brazil: Findings From Salvador's STEMI Registry (RESISST). J Am Heart Assoc.

[140] Silva PGMBE, Dutra AAF, Manfredi AB, Sampaio PPN, Correa CM, Griz HB, Setta D, Furlan V (2021). Reduction in the Number of Patients with Suspected and Confirmed Acute Coronary Syndrome During the Early Months of the COVID-19 Pandemic: Analysis of a Brazilian Network. Arq Bras Cardiol.

[141] Normando PG, Araujo-Filho JA, Fonseca GA, Rodrigues REF, Oliveira VA, Hajjar LA, Almeida ALC, Bocchi EA, Salemi VMC, Melo M (2021). Reduction in Hospitalization and Increase in Mortality Due to Cardiovascular Diseases during the COVID-19 Pandemic in Brazil. Arq Bras Cardiol.

[142] Ribeiro EG, Pinheiro PC, Nascimento BR, Cacique JPP, Teixeira RA, Nascimento JS, Franco TB, Brant LCC, Malta DC (2022). Impact of the COVID-19 Pandemic on Hospital Admissions for Cardiovascular Diseases in a Large Brazilian Urban Center. Rev Soc Bras Med Trop.

[143] Nascimento BR, Brant LCC, Castro ACT, Froes LEV, Ribeiro ALP, Teixeira RA, Cruz LV, Araújo CBM, Souza CF, Froes ET, Souza SD (2021). Reduction in Hospital Admissions Associated with Coronary Events during the COVID-19 Pandemic in the Brazilian Private Health System: Data from the UNIMED-BH System. Rev Soc Bras Med Trop.

[144] Bocchi EA (2013). Heart Failure in South America. Curr Cardiol Rev.

[145] Arteaga E, Ianni BM, Fernandes F, Mady C (2005). Benign Outcome in a Long-Term Follow-Up of Patients with Hypertrophic Cardiomyopathy in Brazil. Am Heart J.

[146] World Health Organization (2015). Chagas Disease in Latin America: An Epidemiological Update Based on 2010 Estimates. Wkly Epidemiol Rec.

[147] Martins-Melo FR, Ramos AN, Alencar CH, Heukelbach J (2014). Prevalence of Chagas Disease in Brazil: A Systematic Review and Meta-Analysis. Acta Trop.

[148] Sabino EC, Ribeiro ALP, Salemi VM, Oliveira CL, Antunes AP, Menezes MM, Ianni BM, Nastari L, Fernandes F, Patavino GM, Sachdev V, Capuani L, Almeida-Neto C, Carrick DM, Wright D, Kavounis K, Goncalez TT, Carneiro-Proietti AB, Custer B, Busch MP, Murphy EL, National Heart, Lung, and Blood Institute Retrovirus Epidemiology Donor Study-II (REDS-II), International Component (2013). Ten-Year Incidence of Chagas Cardiomyopathy among Asymptomatic Trypanosoma Cruzi-Seropositive Former Blood Donors. Circulation.

[149] Nunes MCP, Buss LF, Silva JLP, Martins LNA, Oliveira CDL, Cardoso CS, Brito BOF, Ferreira AM, Oliveira LC, Bierrenbach AL, Fernandes F, Busch MP, Hotta VT, Martinelli LMB, Soeiro MCFA, Brentegani A, Salemi VMC, Menezes MM, Ribeiro ALP, Sabino EC (2021). Incidence and Predictors of Progression to Chagas Cardiomyopathy: Long-Term Follow-Up of Trypanosoma cruzi-Seropositive Individuals. Circulation.

[150] Brito BOF, Lima EM, Soliman EZ, Silva EF, Lima-Costa MF, Ribeiro ALP (2023). The Evolution of Electrocardiographic Abnormalities in the Elderly with Chagas Disease During 14 Years of Follow-Up: The Bambui Cohort Study of Aging. PLoS Negl Trop Dis.

[151] Chadalawada S, Sillau S, Archuleta S, Mundo W, Bandali M, Parra-Henao G, Rodriguez-Morales AJ, Villamil-Gomez WE, Suárez JA, Shapiro L, Hotez PJ, Woc-Colburn L, De Santo K, Rassi A, Franco-Paredes C, Henao-Martínez AF (2020). Risk of Chronic Cardiomyopathy among Patients with the Acute Phase or Indeterminate Form of Chagas Disease: A Systematic Review and Meta-analysis. JAMA Netw Open.

[152] Medeiros CA, Silva MBA, Oliveira ALS, Alves SMM, Barros MDNDDS, Cavalcanti MDGAM, Oliveira GMA, Carrazzone CFV, Oliveira WA, Medeiros ZM (2022). Mapping the Morbidity and Mortality of Chagas Disease in an Endemic Area in Brazil. Rev Inst Med Trop Sao Paulo.

[153] Martins-Melo FR, Castro MC, Werneck GL (2021). Levels and Trends in Chagas Disease-Related Mortality in Brazil, 2000-2019. Acta Trop.

[154] Capuani L, Bierrenbach AL, Alencar AP, Mendrone A, Ferreira JE, Custer B, Ribeiro ALP, Sabino EC (2017). Mortality among Blood Donors Seropositive and Seronegative for Chagas Disease (1996-2000) in São Paulo, Brazil: A Death Certificate Linkage Study. PLoS Negl Trop Dis.

[155] Ayub-Ferreira SM, Mangini S, Issa VS, Cruz FD, Bacal F, Guimarães GV, Chizzola PR, Conceição-Souza GE, Marcondes-Braga FG, Bocchi EA (2013). Mode of Death on Chagas Heart Disease: Comparison with Other Etiologies. A Subanalysis of the REMADHE Prospective Trial. PLoS Negl Trop Dis.

[156] Borges-Pereira J, Coura JR, Zauza PL, Pirmez C, Xavier SS (2020). Chagas Disease in Virgem da Lapa, Minas Gerais, Brazil: Left Ventricle Aneurysm and the Risk of Death in the 24-Year Interval. Mem Inst Oswaldo Cruz.

[157] Nadruz W, Gioli-Pereira L, Bernardez-Pereira S, Marcondes-Braga FG, Fernandes-Silva MM, Silvestre OM, Sposito AC, Ribeiro ALP, Bacal F, Fernandes F, Krieger JE, Mansur AJ, Pereira AC (2018). Temporal Trends in the Contribution of Chagas Cardiomyopathy to Mortality among Patients with Heart Failure. Heart.

[158] Ferreira AM, Sabino ÉC, Oliveira LC, Oliveira CDL, Cardoso CS, Ribeiro ALP, Damasceno RF, Nunes MDCP, Haikal DSA (2020). Impact of the Social Context on the Prognosis of Chagas Disease Patients: Multilevel Analysis of a Brazilian Cohort. PLoS Negl Trop Dis.

[159] Oliveira CL, Nunes MCP, Colosimo EA, Lima EM, Cardoso CS, Ferreira AM, Oliveira LC, Moreira CHV, Bierrenbach AL, Haikal DSA, Peixoto SV, Lima-Costa MF, Sabino EC, Ribeiro ALP (2020). Risk Score for Predicting 2-Year Mortality in Patients With Chagas Cardiomyopathy From Endemic Areas: SaMi-Trop Cohort Study. J Am Heart Assoc.

[160] Chadalawada S, Rassi A, Samara O, Monzon A, Gudapati D, Barahona LV, Hyson P, Sillau S, Mestroni L, Taylor M, Moreira MCV, Santo K, Agudelo Higuita NI, Franco-Paredes C, Henao-Martínez AF (2021). Mortality Risk in Chronic Chagas Cardiomyopathy: A Systematic Review and Meta-Analysis. ESC Heart Fail.

[161] Ciapponi A, Alcaraz A, Calderón M, Matta MG, Chaparro M, Soto N, Bardach A (2016). Burden of Heart Failure in Latin America: A Systematic Review and Meta-Analysis. Rev Esp Cardiol.

[162] Moraes RS, Fuchs FD, Moreira LB, Wiehe M, Pereira GM, Fuchs SC (2003). Risk Factors for Cardiovascular Disease in a Brazilian Population-Based Cohort Study. Int J Cardiol.

[163] Jorge AL, Rosa ML, Martins WA, Correia DM, Fernandes LC, Costa JA, Moscavitch SD, Jorge BA, Mesquita ET (2016). The Prevalence of Stages of Heart Failure in Primary Care: A Population-Based Study. J Card Fail.

[164] Nogueira IDB, Nogueira PAMS, Fonseca AMC, Santos, TZM, Souza DE, Ferreira GMH (2019). Prevalência de Insuficiência Cardíaca e Associação com Saúde Autorreferida no Brasil: Pesquisa Nacional de Saúde - 2013. Acta Fisiátrica.

[165] Cestari VRF, Garces TS, Sousa GJB, Maranhão TA, Souza JD, Pereira MLD, Pessoa VLMP, Sales JTL, Florêncio RS, Souza LC, Vasconcelos GG, Sobral MGV, Damasceno LLV, Moreira TMM (2022). Spatial Distribution of Mortality for Heart Failure in Brazil, 1996 - 2017. Arq Bras Cardiol.

[166] Arruda VL, Machado LMG, Lima JC, Silva PRS (2022). Trends in Mortality from Heart Failure in Brazil: 1998 to 2019. Rev Bras Epidemiol.

[167] Albuquerque DC, David J, Bacal F, Rohde LE, Bernardez-Pereira S, Berwanger O, Almeida DR, Investigadores Estudo BREATHE (2015). I Brazilian Registry of Heart Failure - Clinical Aspects, Care Quality and Hospitalization Outcomes. Arq Bras Cardiol.

[168] Nicolao CZ, Ferreira JB, Paz AA, Linch GFC, Rover M, Souza EN (2019). Heart Failure: An Overview of Morbidity and Mortality in Rio Grande do Sul. Int J Cardiovac Sci.

[169] Fernandes ADF, Fernandes GC, Mazza MR, Knijnik LM, Fernandes GS, Vilela AT, Badiye A, Chaparro SV (2020). A 10-Year Trend Analysis of Heart Failure in the Less Developed Brazil. Arq Bras Cardiol.

[170] Cruz JAW, Buso GM, Moura LAZ, Moraes TP, Cunha MAVC, Zequinão T, Gasparetto J, Tuon FF, Marques S (2022). Brazilian Public Health System: History and Profile of Heart Failure Care and the Impacts of COVID-19. J Bras Econ Saude.

[171] Fernandes-Silva MM, Adam EL, Bernardez-Pereira S, Silva SA, Passaglia LG, Pereira KRP, Guedes MAV, Souza JD, Paola ÂAV, Rivera MAM, Resende ES, Albuquerque DC, Bacal F, Ribeiro ALP, Morgan L, Smith SC, Taniguchi FP (2022). Heart Failure Mortality during COVID-19 Pandemic: Insights from a Cohort of Public Hospitals in Brazil. Arq Bras Cardiol.

[172] Zaidel EJ, Forsyth CJ, Novick G, Marcus R, Ribeiro ALP, Pinazo MJ, Morillo CA, Echeverría LE, Shikanai-Yasuda MA, Buekens P, Perel P, Meymandi SK, Ralston K, Pinto F, Sosa-Estani S (2020). COVID-19: Implications for People with Chagas Disease. Glob Heart.

[173] Molina I, Marcolino MS, Pires MC, Ramos LEF, Silva RT, Guimarães MH, Oliveira IJR, Carvalho RLR, Nunes AGS, Barros ALRM, Scotton ALBA, Madureira AAC, Farace BL, Carvalho CA, Rodrigues FD, Anschau F, Botoni FA, Nascimento GF, Duani H, Guimarães HC, Alvarenga JC, Moreira LB, Zandoná LB, Almeida LF, Oliveira LM, Kopittke L, Castro LC, Santos LEA, Cabral MAS, Ferreira MAP, Sampaio NCS, Oliveira NR, Assaf PL, Lopes SJTS, Fereguetti TO, Santos VB, Carvalho VEB, Ramires YC, Ribeiro ALP, Moscoso FAB, Moura R, Polanczyk CA, Nunes MCP (2021). Chagas Disease and SARS-CoV-2 Coinfection Does Not Lead to Worse In-Hospital Outcomes. Sci Rep.

[174] Martins-Melo FR, Castro MC, Ribeiro ALP, Heukelbach J, Werneck GL (2022). Deaths Related to Chagas Disease and COVID-19 Co-Infection, Brazil, March-December 2020. Emerg Infect Dis.

[175] Souza MV, Nascimento LF, Kozlowsky I, Farjun B, França K, Kuriyama SN, Fidalgo A (2022). Impacts of Heart Failure on the Brazilian Health and Pension System: What is the Cost of the Disease?. J Bras Econ Saude.

[176] Villela PB, Santos SC, Oliveira GMM (2021). Heart Failure Quantified by Underlying Cause and Multiple Cause of Death in Brazil between 2006 and 2016. BMC Public Health.

[177] Nóbrega AA (2014). Carga de Doença Associada à Cardiomiopatia Chagásica no Brasil.

[178] Thomas H, Diamond J, Vieco A, Chaudhuri S, Shinnar E, Cromer S, Perel P, Mensah GA, Narula J, Johnson CO, Roth GA, Moran AE (2018). Global Atlas of Cardiovascular Disease 2000-2016: The Path to Prevention and Control. Glob Heart.

[179] Iung B, Vahanian A (2014). Epidemiology of Acquired Valvular Heart Disease. Can J Cardiol.

[180] Nascimento BR, Beaton AZ, Nunes MC, Diamantino AC, Carmo GA, Oliveira KK, Oliveira CM, Meira ZM, Castilho SR, Lopes EL, Castro IM, Rezende VM, Chequer G, Landay T, Tompsett A, Ribeiro ALP, Sable C, PROVAR (Programa de RastreamentO da VAlvopatia Reumática) Investigators (2016). Echocardiographic Prevalence of Rheumatic Heart Disease in Brazilian Schoolchildren: Data from the PROVAR Study. Int J Cardiol.

[181] Santos JPAD, Carmo GALD, Beaton AZ, Lourenço TV, Diamantino AC, Nunes MDCP, Sable C, Nascimento BR (2017). Challenges for the Implementation of the First Large-Scale Rheumatic Heart Disease Screening Program in Brazil: The PROVAR Study Experience. Arq Bras Cardiol.

[182] Ribeiro GS, Tartof SY, Oliveira DW, Guedes AC, Reis MG, Riley LW, Ko AI (2012). Surgery for Valvular Heart Disease: A Population-Based Study in a Brazilian Urban Center. PLoS One.

[183] Watkins DA, Johnson CO, Colquhoun SM, Karthikeyan G, Beaton A, Bukhman G, Forouzanfar MH, Longenecker CT, Mayosi BM, Mensah GA, Nascimento BR, Ribeiro ALP, Sable CA, Steer AC, Naghavi M, Mokdad AH, Murray CJL, Vos T, Carapetis JR, Roth GA (2017). Global, Regional, and National Burden of Rheumatic Heart Disease, 1990-2015. N Engl J Med.

[184] Meira ZMA, Castilho SR, Barros MVL, Vitarelli AM, Capanema FD, Moreira NS, Camargos PAM, Mota CCC (1995). Prevalence of Rheumatic Fever in Children from a Public High School in Belo Horizonte. Arq Bras Cardiol.

[185] Berent R, Auer J, von Duvillard SP, Sinzinger H, Schmid P. (2010). Komplikationen bei der Ergometrie. Herz.

[186] Lindstrom M, De Cleene N, Dorsey H, Fuster V, Johnson CO, Le Grand KE, Mensah GA, Razo C, Stark B, Varieur Turco J, Roth GA (2022). Global Burden of Cardiovascular Diseases and Risks Collaboration, 1990-2021. J Am Coll Cardiol.

[187] Moraes RC, Katz M, Tarasoutchi F (2014). Clinical and Epidemiological Profile of Patients with Valvular Heart Disease Admitted to the Emergency Department. Einstein.

[188] Iung B, Baron G, Butchart EG, Delahaye F, Gohlke-Bärwolf C, Levang OW, Tornos P, Vanoverschelde JL, Vermeer F, Boersma E, Ravaud P, Vahanian A (2003). A Prospective Survey of Patients with Valvular Heart Disease in Europe: The Euro Heart Survey on Valvular Heart Disease. Eur Heart J.

[189] Lopes MACQ, Nascimento BR, Oliveira GMM (2020). Treatment of Aortic Stenosis in Elderly Individuals in Brazil: How Long Can We Wait?. Arq Bras Cardiol.

[190] Freeman RV, Otto CM (2005). Spectrum of Calcific Aortic Valve Disease: Pathogenesis, Disease Progression, and Treatment Strategies. Circulation.

[191] Grinberg M, Accorsi TA (2009). Aortic Stenosis in the Elderly: A Brazilian Perspective. Arq Bras Cardiol.

[192] Thaden JJ, Nkomo VT, Enriquez-Sarano M (2014). The Global Burden of Aortic Stenosis. Prog Cardiovasc Dis.

[193] Tarasoutchi F, Montera MW, Ramos AIO, Sampaio RO, Rosa VEE, Accorsi TAD, Santis A, Fernandes JRC, Pires LJT, Spina GS, Vieira MLC, Lavitola PL, Ávila WS, Paixão MR, Bignoto T, Togna DJD, Mesquita ET, Esteves WAM, Atik F, Colafranceschi AS, Moises VA, Kiyose AT, Pomerantzeff PMA, Lemos PA, Brito FS, Weksler C, Brandão CMA, Poffo R, Simões R, Rassi S, Leães PE, Mourilhe-Rocha R, Pena JLB, Jatene FB, Barbosa MM, Abizaid A, Ribeiro HB, Bacal F, Rochitte CE, Fonseca JHAPD, Ghorayeb SKN, Lopes MACQ, Spina SV, Pignatelli RH, Saraiva JFK (2020). Update of the Brazilian Guidelines for Valvular Heart Disease - 2020. Arq Bras Cardiol.

[194] Nunes MC, Gelape CL, Ferrari TC (2010). Profile of Infective Endocarditis at a Tertiary Care Center in Brazil During a Seven-Year Period: Prognostic Factors and In-Hospital Outcome. Int J Infect Dis.

[195] Bezerra RL, Salgado LS, Silva YM, Figueiredo GGR, Bezerra RM, Machado ELG, Gomes IC, Cunha AGJ (2022). Epidemiological Profile of Patients with Infective Endocarditis at three Tertiary Centers in Brazil from 2003 to 2017. Int J Cardiovasc Sciences.

[196] Khan A, Aslam A, Satti KN, Ashiq S (2020). Infective Endocarditis Post-Transcatheter Aortic Valve Implantation (TAVI), Microbiological Profile and Clinical Outcomes: A Systematic Review. PLoS One.

[197] Dvir D, Simonato M, Amat-Santos I, Latib A, Kargoli F, Nombela-Franco L, Agrifoglio M, Giannini F, Regazzoli D, Reimers B, Villa E, M Becerra-Muñoz V, Mennuni M, Rognoni A, Modine T, Leroux L, Estévez-Loureiro R, Nerla R, Castriota F, Cerillo A, Søndergaard L, Iadanza A, Duncan A, Vincent F, Mancone M, Birtolo L, Maestrini V, Testa L, Wojakowski W, Salizzoni S, Esteves V, Mangione F, Zukowski C, Amabile N, Shuvy M, Stone GW (2021). Severe Valvular Heart Disease and COVID-19: Results from the Multicenter International Valve Disease Registry. Struct Heart.

[198] Martins JFBS, Nascimento ER, Nascimento BR, Sable CA, Beaton AZ, Ribeiro ALP, Meira W, Pappa GL (2021). Towards Automatic Diagnosis of Rheumatic Heart Disease on Echocardiographic Exams Through Video-Based Deep Learning. J Am Med Inform Assoc.

[199] Nascimento BR, Martins JFBS, Nascimento ER, Pappa GL, Sable CA, Beaton AZ, Gomes PR, Nunes MCP, Oliveira KKB, Franco J, Pereira AFC, Rezende B, Mata MDO, Ribeiro ALP, Meira W (2020). Deep Learning for Automatic Identification of Rheumatic Heart Disease in Echocardiographic Screening Images - Data from the Atmosphere-PROVAR study. J Am Coll Cardiol.

[200] Edwards LA, Feng F, Iqbal M, Fu Y, Sanyahumbi A, Hao S, McElhinney DB, Ling XB, Sable C, Luo J (2023). Machine Learning for Pediatric Echocardiographic Mitral Regurgitation Detection. J Am Soc Echocardiogr.

[201] Pinto MM, Brant LCC, Foppa M, Garcia-Silva KB, Oliveira RAM, Fonseca MJM, Alvim S, Lotufo PA, Mill JG, Barreto SM, Macfarlane PW, Ribeiro ALP (2017). Major Electrocardiographic Abnormalities According to the Minnesota Coding System among Brazilian Adults (from the ELSA-Brasil Cohort Study). Am J Cardiol.

[202] Santos IS, Lotufo PA, Brant L, Pinto MM, Pereira ADC, Barreto SM, Ribeiro ALP, Thomas GN, Lip GYH, Bensenor IM (2021). Atrial Fibrillation Diagnosis Using ECG Records and Self-Report in the Community: Cross-Sectional Analysis from ELSA-Brasil. Arq Bras Cardiol.

[203] Kawabata-Yoshihara LA, Benseñor IM, Kawabata VS, Menezes PR, Scazufca M, Lotufo PA (2009). Prevalence of Electrocardiographic Findings in Elderly Individuals: The Sao Paulo Aging & Health Study. Arq Bras Cardiol.

[204] Marcolino MS, Palhares DM, Benjamin EJ, Ribeiro ALP (2015). Atrial Fibrillation: Prevalence in a Large Database of Primary Care Patients in Brazil. Europace.

[205] Moraes ERFL, Cirenza C, Lopes RD, Carvalho AC, Guimaraes PO, Rodrigues AAE, Paola AAV (2019). Prevalence of Atrial Fibrillation and Stroke Risk Assessment Based on Telemedicine Screening Tools in a Primary Healthcare Setting. Eur J Intern Med.

[206] Marcolino MS, Santos TMM, Stefanelli FC, Oliveira JAQ, Silva MVRS, Andrade DF, Silva GKME, Ribeiro ALP (2017). Cardiovascular Emergencies in Primary Care: An Observational Retrospective Study of a Large-Scale Telecardiology Service. Sao Paulo Med J.

[207] Jerjes-Sanchez C, Corbalan R, Barretto ACP, Luciardi HL, Allu J, Illingworth L, Pieper KS, Kayani G, GARFIELD-AF Investigators (2019). Stroke Prevention in Patients from Latin American Countries with Non-Valvular Atrial Fibrillation: Insights from the GARFIELD-AF Registry. Clin Cardiol.

[208] Paixão GMM, Silva LGS, Gomes PR, Lima EM, Ferreira MPF, Oliveira DM, Ribeiro MH, Ribeiro AH, Nascimento JS, Canazart JA, Ribeiro LB, Benjamin EJ, Macfarlane PW, Marcolino MS, Ribeiro ALP (2020). Evaluation of Mortality in Atrial Fibrillation: Clinical Outcomes in Digital Electrocardiography (CODE) Study. Glob Heart.

[209] Ribeiro ALP, Marcolino MS, Prineas RJ, Lima-Costa MF (2014). Electrocardiographic Abnormalities in Elderly Chagas Disease Patients: 10-Year Follow-Up of the Bambui Cohort Study of Aging. J Am Heart Assoc.

[210] Lopes RD, Silva PGMB, Hoffman CR, Cavalvante MA, Miranda CM, Esper RB, Lima GG, Ritt LEF, Silva RMFL, Nakazone MA, Almeida AP, Pavanello R, Lima CEB, Backes LM, Oliveira LH, Souza OF, Lorga AM, God EMG, Jorge JCM, Luiz AA, Martins SFPP, Dantas RC, Vieira RDO, Zimerman LI, Alves ÁR, Figueiredo MJO, Gomes SPC, Lima LM, Damiani LP, Teixeira RA, Fagundes AA, Saad EB, RECALL Investigators (2023). The First Brazilian Cardiovascular Registry of Atrial Fibrillation: Primary Results of the RECALL Study. Am Heart J.

[211] Amaral CHD, Amaral AR, Nagel V, Venancio V, Garcia AC, Magalhaes PS, Longo AL, Moro CH, Reis FI, D'Avila A, Cabral NL (2017). Incidence and Functional Outcome of Atrial Fibrillation and Non-Atrial Fibrillation- Related Cardioembolic Stroke in Joinville, Brazil: A Population-Based Study. Arq Neuropsiquiatr.

[212] Lange MC, Ribas G, Scavasine V, Ducci RD, Mendes DC, Zétola VHF, Cabral N, Rundek T (2018). Stroke Recurrence in the Different Subtypes of Ischemic Stroke. The Importance of the Intracranial Disease. Arq Neuropsiquiatr.

[213] Figueiredo MM, Rodrigues AC, Alves MB, Cendoroglo MC, Silva GS (2014). Score for Atrial Fibrillation Detection in Acute Stroke and Transient Ischemic Attack Patients in a Brazilian Population: The Acute Stroke Atrial Fibrillation Scoring System. Clinics.

[214] Caramelli B, Yu PC, Cardozo FAM, Magalhães IR, Spera RR, Amado DK, Escalante-Rojas MC, Gualandro DM, Calderaro D, Tavares CAM, Borges FA, Pastana AF, Matheus MG, Brucki SMD, Rodrigues ACO, Nitrini R, Caramelli P (2022). Effects of Dabigatran Versus Warfarin on 2-Year Cognitive Outcomes in Old Patients with Atrial Fibrillation: Results from the GIRAF Randomized Clinical Trial. BMC Med.

[215] Diegolli H, Oliveira RENDN, Silva CFD, Silva GFD, Souza FF, Machado FRA, Lacerda MP (2023). Incidence of Cardioembolic Stroke Related to Atrial Fibrillation in Joinville, Brazil. Arq Neuropsiquiatr.

[216] Lloyd-Jones DM, Hong Y, Labarthe D, Mozaffarian D, Appel LJ, Van Horn L, Greenlund K, Daniels S, Nichol G, Tomaselli GF, Arnett DK, Fonarow GC, Ho PM, Lauer MS, Masoudi FA, Robertson RM, Roger V, Schwamm LH, Sorlie P, Yancy CW, Rosamond WD, American Heart Association Strategic Planning Task Force and Statistics Committee (2010). Defining and Setting National Goals for Cardiovascular Health Promotion and Disease Reduction: The American Heart Association's Strategic Impact Goal Through 2020 and Beyond. Circulation.

[217] Santos IS, Lotufo PA, Goulart AC, Brant LCC, Pinto MM, Pereira AC, Barreto SM, Ribeiro ALP, Thomas GN, Lip GYH, Bensenor IM, Arasalingam A, Beane A, Bensenor IM, Brocklehurst P, Cheng KK, El-Bouri W, Feng M, Goulart AC, Greenfield S, Guo Y, Guruparan M, Gusso G, Gooden TE, Haniffa R, Humphreys L, Jolly K, Jowett S, Kumarendran B, Lancashire E, Lane DA, Li X, Lip Co-Pi GYH, Li YG, Lobban T, Lotufo PA, Manseki-Holland S, Moore DJ, Nirantharakumar K, Olmos RD, Paschoal E, Pirasanth P, Powsiga U, Romagnolli C, Santos IS, Shantsila A, Sheron VA, Shribavan K, Szmigin I, Subaschandren K, Surenthirakumaran R, Tai M, Neil Thomas Co-Pi G, Varella AC, Wang H, Wang J, Zhang H, Zhong J, NIHR Global Health Research Group on Atrial Fibrillation Management (2022). Cardiovascular Health and Atrial Fibrillation or Flutter: A Cross-Sectional Study from ELSA-Brasil. Arq Bras Cardiol.

[218] Diamantino AC, Nascimento BR, Beaton AZ, Nunes MCP, Oliveira KKB, Rabelo LC, Barbosa MM, Tompsett AR, Olivieri L, Mata MD, Costa WAA, Pereira AF, Diamantino LC, Ribeiro ALP, Sable C, Brant LCC (2020). Atrial Fibrillation Detection with a Portable Device During Cardiovascular Screening in Primary Care. Heart.

[219] Lima C, Martinelli M, Peixoto GL, Siqueira SF, Wajngarten M, Silva RT, Costa R, Roberto R, Ramires JA (2016). Silent Atrial Fibrillation in Elderly Pacemaker Users: A Randomized Trial Using Home Monitoring. Ann Noninvasive Electrocardiol.

[220] Kiuchi MG, Chen S, Silva GR, Paz LMR, SoutoG LL (2016). Register of Arrhythmias in Patients with Pacemakers and Mild to Moderate Chronic Kidney Disease (RYCKE): Results from an Observational Cohort Study. Relampa.

[221] Costa MAC, Santos JFLP, Schafranski MD (2022). Prevalence of Atrial Fibrillation in Pacemaker Patients. Int J Cardiovasc Sci.

[222] Mendes FS, Atié J, Garcia MI, Gripp EA, Sousa AS, Feijó LA, Xavier SS (2014). Atrial Fibrillation in Decompensated Heart Failure: Associated Factors and In-Hospital Outcome. Arq Bras Cardiol.

[223] Barbieri LR, Sobral ML, Gerônimo GM, Santos GG, Sbaraíni E, Dorfman FK, Stolf NA (2013). Incidence of Stroke and Acute Renal Failure in Patients of Postoperative Atrial Fibrillation after Myocardial Revascularization. Rev Bras Cir Cardiovasc.

[224] Bohatch MS, Matkovski PD, Di Giovanni FJ, Fenili R, Varella EL, Dietrich A (2015). Incidence of Postoperative Atrial Fibrillation in Patients Undergoing On-Pump and Off-Pump Coronary Artery Bypass Grafting. Rev Bras Cir Cardiovasc.

[225] Pivatto F, Teixeira GF, Sant'anna JR, Py PM, Prates PR, Nesralla IA, Kalil RA (2014). Advanced age and Incidence of Atrial Fibrillation in the Postoperative Period of Aortic Valve Replacement. Rev Bras Cir Cardiovasc.

[226] Sá MP, Sá MV, Albuquerque AC, Silva BB, Siqueira JW, Brito PR, Ferraz PE, Lima RC (2012). Predicting Risk of Atrial Fibrillation after Heart Valve Surgery: Evaluation of a Brazilian Risk Score. Rev Bras Cir Cardiovasc.

[227] Folla CO, Melo CC, Silva RC (2016). Predictive Factors of Atrial Fibrillation after Coronary Artery Bypass Grafting. Einstein.

[228] Mejia OAV, Borgomoni GB, Dallan LRP, Mioto BM, Accorsi TAD, Lima EG, Soeiro AM, Lima FG, Brandão CMA, Pomerantzeff PMA, Dallan LAO, Lisboa LAF, Jatene FB (2022). Quality Improvement Program in Latin America Decreases Mortality after Cardiac Surgery: A before-after Intervention Study. Int J Surg.

[229] Marcolino MS, Palhares DM, Ferreira LR, Ribeiro ALP (2015). Electrocardiogram and Chagas Disease: A Large Population Database of Primary care Patients. Glob Heart.

[230] Cardoso CS, Sabino EC, Oliveira CD, Oliveira LC, Ferreira AM, Cunha-Neto E, Bierrenbach AL, Ferreira JE, Haikal DS, Reingold AL, Ribeiro ALP (2016). Longitudinal Study of Patients with Chronic Chagas Cardiomyopathy in Brazil (SaMi-Trop project): A Cohort Profile. BMJ Open.

[231] Rojas LZ, Glisic M, Pletsch-Borba L, Echeverría LE, Bramer WM, Bano A, Stringa N, Zaciragic A, Kraja B, Asllanaj E, Chowdhury R, Morillo CA, Rueda-Ochoa OL, Franco OH, Muka T (2018). Electrocardiographic Abnormalities in Chagas Disease in the General Population: A Systematic Review and Meta-Analysis. PLoS Negl Trop Dis.

[232] Rassi A, Rassi A, Little WC, Xavier SS, Rassi SG, Rassi AG, Rassi GG, Hasslocher-Moreno A, Sousa AS, Scanavacca MI (2006). Development and Validation of a Risk Score for Predicting Death in Chagas' Heart Disease. N Engl J Med.

[233] Honorato MO, Sousa JT, Honorato LFB, Watanabe N, Goulart GM, Prado RRD (2023). Atrial Fibrillation and Sepsis in Elderly Patients and Their Associaton with In-Hospital Mortality. Arq Bras Cardiol.

[234] Paschoal E, Gooden TE, Olmos RD, Lotufo PA, Benseñor IM, Manaseki-Holland S, Lip GYH, Thomas GN, Jolly K, Lancashire E, Lane DA, Greenfield S, Goulart AC, NIHR Global Health Research Group on Atrial Fibrillation Management (2022). Health Care Professionals' Perceptions About Atrial Fibrillation Care in the Brazilian Public Primary Care System: A Mixed-Methods Study. BMC Cardiovasc Disord.

[235] Vinereanu D, Lopes RD, Bahit MC, Xavier D, Jiang J, Al-Khalidi HR, He W, Xian Y, Ciobanu AO, Kamath DY, Fox KA, Rao MP, Pokorney SD, Berwanger O, Tajer C, Silva PGMB, Roettig ML, Huo Y, Granger CB, IMPACT-AF investigators (2017). A Multifaceted Intervention to Improve Treatment with Oral Anticoagulants in Atrial Fibrillation (IMPACT-AF): An International, Cluster-Randomised Trial. Lancet.

[236] Goulart AC, Olmos RD, Santos IS, Tunes G, Alencar AP, Thomas N, Lip GY, Lotufo PA, Benseñor IM (2022). The Impact of Atrial Fibrillation and Long-Term Oral Anticoagulant Use on All-Cause and Cardiovascular Mortality: A 12-Year Evaluation of the Prospective Brazilian Study of Stroke Mortality and Morbidity. Int J Stroke.

[237] Guerrero AZA, Coutinho EL, Ferraz MB, Cirenza C, Santos MCED, Ferraro JR, Paola AAV (2022). Economy- and Social-Based Strategies for Anticoagulation of Patients with Atrial Fibrillation. Arq Bras Cardiol.

[238] Oliveira LH, Mallmann FB, Botelho FN, Paul LC, Gianotto M, Abt RB, Silva NJ, Luize CM, Nogueira FL, Carvalho RS, Paola AA, Cirenza C (2012). Cross-Sectional Study of Treatment Strategies on Atrial Fibrillation. Arq Bras Cardiol.

[239] Mountantonakis SE, Saleh M, Fishbein J, Gandomi A, Lesser M, Chelico J, Gabriels J, Qiu M, Epstein LM, Northwell COVID-19 Research Consortium (2021). Atrial Fibrillation is an Independent Predictor for In-Hospital Mortality in Patients Admitted with SARS-CoV-2 Infection. Heart Rhythm.

[240] Paulino MR, Moreira JAS, Correia MG, Santos LRA, Duarte IP, Sabioni LR, Mucillo FB, Garrido RQ, Pacheco SL, Lorenzo A, Lamas CDC (2021). COVID-19 in Patients with Cardiac Disease: Impact and Variables Associated with Mortality in a Cardiology Center in Brazil. Am Heart J Plus.

[241] Brant LCC, Pinheiro PC, Machado IE, Correa PRL, Santos MR, Ribeiro ALP, Tupinambás U, Santiago CF, Souza MFM, Malta DC, Passos VMA (2021). The Impact of COVID-19 Pandemic Course in the Number and Severity of Hospitalizations for Other Natural Causes in a Large Urban Center in Brazil. PLOS Glob Public Health.

[242] Biton S, Gendelman S, Ribeiro AH, Miana G, Moreira C, Ribeiro ALP, Behar JA (2021). Atrial Fibrillation Risk Prediction from the 12-Lead Electrocardiogram Using Digital Biomarkers and Deep Representation Learning. Eur Heart J Digit Health.

[243] Müller J, Heck PB, Ewert P, Hager A. (2017). Noninvasive Screening for Pulmonary Hypertension by Exercise Testing in Congenital Heart Disease. Ann Thorac Surg.

[244] Malta DC, Gonçalves RPF, Machado ÍE, Freitas MIF, Azeredo C, Szwarcwald CL (2018). Prevalence of Arterial Hypertension According to Different Diagnostic Criteria, National Health Survey. Rev Bras Epidemiol.

[245] Brant LCC, Nascimento BR, Veloso GA, Gomes CS, Polanczyk C, Oliveira GMM, Flor LS, Gakidou E, Ribeiro ALP, Malta DC (2022). Burden of Cardiovascular Diseases Attributable to Risk Factors in Brazil: Data from the "Global Burden of Disease 2019" Study. Rev Soc Bras Med Trop.

[246] Paiva MHP, Miranda VA, Oliveira ARS, Cruz KJC, Araújo RMS, Oliveira KA (2022). Prevalence of Metabolic Syndrome and its Components in Brazilian Adolescents: A Systematic Review and Meta-Analysis. Rev Paul Pediatr.

[247] Welser L, Pfeiffer KA, Silveira JFC, Valim ARM, Renner JDP, Reuter CP (2023). Incidence of Arterial Hypertension is Associated with Adiposity in Children and Adolescents. Arq Bras Cardiol.

[248] Malta DC, Bernal RTI, Ribeiro EG, Moreira AD, Felisbino-Mendes MS, Velásquez-Meléndez JG (2022). Hipertensão Arterial e Fatores Associados: Pesquisa Nacional de Saúde, 2019. Rev Saude Publica.

[249] Malta DC, Silva AGD, Gomes CS, Stopa SR, Oliveira MM, Sardinha LMV, Caixeta RB, Pereira CA, Rios-Neto ELG (2022). Monitoring the Goals of the Plans for Coping with Chronic Non-Communicable Diseases: Results of the National Health Survey, Brazil, 2013 and 2019. Epidemiol Serv Saude.

[250] Macinko J, Mullachery PH (2022). Education-Related Health Inequities in Noncommunicable Diseases: An Analysis of the Brazilian National Health Survey, 2013 and 2019. Cad Saude Publica.

[251] Malta DC, Santos NB, Perillo RD, Szwarcwald CL (2016). Prevalence of High Blood Pressure Measured in the Brazilian Population, National Health Survey, 2013. Sao Paulo Med J.

[252] Brasil, Ministério da Saúde, Secretaria de Vigilância em Saúde, Departamento de Análise em Saúde e Vigilância de Doenças Não Transmissíveis (2021). VIGITEL Brasil 2021: Vigilância de Fatores de Risco e Proteção Proteção para Doenças Crônicas por Inquérito Telefônico: Estimativas sobre Frequência e Distribuição Sociodemográfica de Fatores de Risco e Proteção para Doenças Crônicas nas Capitais dos 26 Estados Brasileiros e no Distrito Federal em 2021.

[253] Scaranni PODS, Cardoso LO, Chor D, Melo ECP, Matos SMA, Giatti L, Barreto SM, Fonseca MJM (2021). Ultra-Processed Foods, Changes in Blood Pressure and Incidence of Hypertension: The Brazilian Longitudinal Study of Adult Health (ELSA-Brasil). Public Health Nutr.

[254] Silva EKP, Barreto SM, Brant LCC, Camelo LV, Araújo EM, Griep RH, Fonseca MJMD, Pereira ADC, Giatti L (2023). Gender, Race/Skin Colour and Incidence of Hypertension in ELSA-Brasil: An Intersectional Approach. Ethn Health.

[255] Coelho JS, Martinez OGE, Siqueira JH, Campos GC, Viana MC, Griep RH, Alvim RO, Mill JG, Molina MCB (2021). Alcoholic Beverage Consumption, Changes in Blood Pressure, and Incidence of Hypertension in the Longitudinal Adult Health Study (ELSA-Brasil). Nutrition.

[256] Malta DC, Felisbino-Mendes MS, Machado ÍE, Veloso GA, Gomes CS, Brant LCC, Ribeiro ALP, Oliveira PPV, Flor LS, Gakidou E (2022). Burden of Disease Attributable to Risk Factors in Brazil: An Analysis of National and Subnational Estimates from the 2019 Global Burden of Disease study. Rev Soc Bras Med Trop.

[257] Pires MP, Moreira CC, Andrade ACS, Rodrigues PMR, Murano AP, Luz VG, Moreira NF (2023). Multimorbidity of Chronic Noncommunicable Diseases: Data from the Brazilian National Health Survey. Rev Chil Nutr.

[258] Silva MVB, Lima CA, Bernardino AO, Gouveia VA (2022). Mortality from Chronic Kidney Disease Secondary to Hypertension in Brazil: A Study of the "Global Burden of Disease". Rev Epidemiol Controle Infec.

[259] Kramer CK, Leitão CB, Viana LV (2022). The Impact of Urbanisation on the Cardiometabolic Health of Indigenous Brazilian Peoples: A Systematic Review and Meta-Analysis, and Data from the Brazilian Health Registry. Lancet.

[260] Castro L, Brant L, Diniz MF, Lotufo P, Bensenor IJ, Chor D, Griep R, Barreto SM, Ribeiro ALP (2023). Association of Hypertension and Insulin Resistance in Individuals Free of Diabetes in the ELSA-Brasil Cohort. Sci Rep.

[261] Malta DC, Gomes CS, Stopa SR, Andrade FMD, Prates EJS, Oliveira PPV, Ferreira SAM, Pereira CA (2022). Inequalities in Health Care and Access to Health Services among Adults with Self-Reported Arterial Hypertension: Brazilian National Health Survey. Cad Saude Publica.

[262] Tavares GA, Ribeiro JB, Almeida-Santos MA, Sousa ACS, Barreto-Filho JAS (2022). Cardiovascular Health Control in the Family Health Strategy. Front Cardiovasc Med.

[263] Malta DC, Bernal RTI, Prates EJS, Vasconcelos NM, Gomes CS, Stopa SR, Sardinha LMV, Pereira CA (2022). Self-Reported Arterial Hypertension, Use of Health Services and Guidelines for Care in Brazilian Population: National Health Survey, 2019. Epidemiol Serv Saude.

[264] Costa KS, Tavares NUL, Tierling VL, Leitão VBG, Stopa SR, Malta DC (2022). National Health Survey 2019: Medication Obtainment Through the Brazilian Popular Pharmacy Program by Adults Being Treated for Hypertension and Diabetes. Epidemiol Serv Saude.

[265] Guimarães JMN, Jackson JW, Barber S, Griep RH, Fonseca MJM, Camelo LV, Barreto SM, Schmidt MI, Duncan BB, Cardoso LO, Pereira AC, Chor D (2023). Racial Inequities in the Control of Hypertension and the Explanatory Role of Residential Segregation: A Decomposition Analysis in the Brazilian Longitudinal Study of Adult Health (ELSA-Brasil). J Racial Ethn Health Disparities.

[266] Malta DC, Gomes CS, Silva AGD, Cardoso LSM, Barros MBA, Lima MG, Souza PRB, Szwarcwald CL (2021). Use of Health Services and Adherence to Social Distancing by Adults with Noncommunicable Diseases during the COVID-19 Pandemic, Brazil, 2020. Cien Saude Colet.

[267] Feitosa FGAM, Feitosa ADM, Paiva AMG, Mota-Gomes MA, Barroso WS, Miranda RD, Barbosa ECD, Brandão AA, Lima-Filho JL, Sposito AC, Coca A, Nadruz W (2022). Impact of the COVID-19 Pandemic on Blood Pressure Control: A Nationwide Home Blood Pressure Monitoring Study. Hypertens Res.

[268] Duarte V, Trevisan MG, Menetrier JV, Costa KD, Cavalheiri JC, Teixeira GT (2022). Perfil Epidemiológico de Óbitos Decorrentes da COVID-19 em um Município do Sudoeste do Paraná. Arq Ciências Saúde UNIPAR.

[269] Santos LG, Baggio JAO, Leal TC, Costa FA, Fernandes TRMO, Silva RVD, Armstrong A, Carmo RF, Souza CDF (2021). Prevalence of Systemic Arterial Hypertension and Diabetes Mellitus in Individuals with COVID-19: A Retrospective Study of Deaths in Pernambuco, Brazil. Arq Bras Cardiol.

[270] Oliveira JC, Barreto-Filho JA (2015). Public Health Policy Based on "Made-in-Brazil" Science: A Challenge for the Arquivos Brasileiros de Cardiologia. Arq Bras Cardiol.

[271] Krumholz HM (2008). Outcomes Research: Generating Evidence for Best Practice and Policies. Circulation.

[272] Instituto Brasileiro de Geografia e Estatística (2020). Pesquisa Nacional de Saúde 2019: Percepção do Estado de Saúde, Estilos de Vida, Doenças Crônicas e Saúde Bucal: Brasil e Grandes Regiões.

[273] Borén J, Chapman MJ, Krauss RM, Packard CJ, Bentzon JF, Binder CJ, Daemen MJ, Demer LL, Hegele RA, Nicholls SJ, Nordestgaard BG, Watts GF, Bruckert E, Fazio S, Ference BA, Graham I, Horton JD, Landmesser U, Laufs U, Masana L, Pasterkamp G, Raal FJ, Ray KK, Schunkert H, Taskinen MR, van de Sluis B, Wiklund O, Tokgozoglu L, Catapano AL, Ginsberg HN (2020). Low-Density Lipoproteins Cause Atherosclerotic Cardiovascular Disease: Pathophysiological, Genetic, and Therapeutic Insights: A Consensus Statement from the European Atherosclerosis Society Consensus Panel. Eur Heart J.

[274] Arnett DK, Blumenthal RS, Albert MA, Buroker AB, Goldberger ZD, Hahn EJ, Himmelfarb CD, Khera A, Lloyd-Jones D, McEvoy JW, Michos ED, Miedema MD, Muñoz D, Smith SC, Virani SS, Williams KA, Yeboah J, Ziaeian B (2019). 2019 ACC/AHA Guideline on the Primary Prevention of Cardiovascular Disease: A Report of the American College of Cardiology/American Heart Association Task Force on Clinical Practice Guidelines. Circulation.

[275] Malta DC, Szwarcwald CL, Machado ÍE, Pereira CA, Figueiredo AW, Sá ACMGN, Velasquez-Melendez G, Santos FMD, Souza PB, Stopa SR, Rosenfeld LG (2019). Prevalence of Altered Total Cholesterol and Fractions in the Brazilian Adult Population: National Health Survey. Rev Bras Epidemiol.

[276] Faria JR, Bento VF, Baena CP, Olandoski M, Gonçalves LG, Abreu GA, Kuschnir MC, Bloch KV (2016). ERICA: Prevalence of Dyslipidemia in Brazilian Adolescents. Rev Saude Publica.

[277] Lima LR, Okamura AB, Carvalho KMB, Dutra ES, Gonçalves VSS (2022). Hypertension and Associated Lipid, Glucose, and Adiposity Parameters in School-Aged Adolescents in the Federal District, Brazil. Arq Bras Cardiol.

[278] Rosini N, Machado MJ, Webster IZ, Moura SA, Cavalcante LS, Silva EL (2013). Simultaneous Prediction of Hyperglycemia and Dyslipidemia in School Children in Santa Catarina State, Brazil Based on Waist Circumference Measurement. Clin Biochem.

[279] Almeida PCD, Silva JP, Pinasco GC, Hegner CC, Mattos DC, Potratz MO, Bravin LS, Silva VR, Lamounier JA (2016). Lipid Profile in Schoolchildren in Vitória - Brazil. J Hum Growth Dev.

[280] Alcântara OD, Silva RC, Assis AM, Pinto EJ (2012). Factors Associated with Dyslipidemia in Children and Adolescents Enrolled in Public Schools of Salvador, Bahia. Rev Bras Epidemiol.

[281] Sá ACMGN, Machado ÍE, Bernal RTI, Malta DC (2021). Factors Associated with High LDL-Cholesterol in the Brazilian Adult Population: National Health Survey. Cien Saude Colet.

[282] Silva RC, Diniz MF, Alvim S, Vidigal PG, Fedeli LM, Barreto SM (2016). Physical Activity and Lipid Profile in the ELSA- Brasil Study. Arq Bras Cardiol.

[283] Moura FA, Dutra-Rodrigues MS, Cassol AS, Parra ES, Zago VH, Panzoldo NB, Alexandre F, Vieira IC, Vendrame F, Virginio VW, Castanho VS, Danelon MR, Nunes VS, Leança CC, Saraiva FK, Coelho OR, Nakandakare E, Quintão EC, Faria EC, Sposito AC (2013). Impact of Seasonality on the Prevalence of Dyslipidemia: A Large Population Study. Chronobiol Int.

[284] Sá ACMGN, Gomes CS, Moreira AD, Velasquez-Melendez G, Malta DC (2022). Prevalence and Factors Associated with Self-Reported Diagnosis of High Cholesterol in the Brazilian Adult Population: National Health Survey 2019. Epidemiol Serv Saude.

[285] Santos RD, Bensenor IM, Pereira AC, Lotufo PA (2016). Dyslipidemia According to Gender and Race: The Brazilian Longitudinal Study of Adult Health (ELSA-Brasil). J Clin Lipidol.

[286] Fonseca HAR, Izar MCO, Drager LF, Pinto IM, Saraiva JFK, Ferreira JFM, Avezum Á, Fonseca FA, Berwanger O (2023). Primary Prevention of Cardiovascular Disease at Community Clinics in the State of São Paulo, Brazil: Results from the Epidemiological Information Study of Communities. Glob Heart.

[287] Harada PH, Miname MH, Benseñor IM, Santos RD, Lotufo PA (2018). Familial Hypercholesterolemia Prevalence in an Admixed Racial Society: Sex and Race Matter. The ELSA-Brasil. Atherosclerosis.

[288] Jannes CE, Santos RD, Souza Silva PR, Turolla L, Gagliardi AC, Marsiglia JD, Chacra AP, Miname MH, Rocha VZ, Salgado WS, Krieger JE, Pereira AC (2015). Familial Hypercholesterolemia in Brazil: Cascade Screening Program, Clinical and Genetic Aspects. Atherosclerosis.

[289] Coutinho ER, Miname MH, Rocha VZ, Bittencourt MS, Jannes CE, Krieger JE, Pereira AC, Santos RD (2023). Cardiovascular Disease Onset in Old People with Severe Hypercholesterolemia. Atherosclerosis.

[290] Santos RD, Pereira C, Cesena F, Laurinavicius AG, Tabone V, Bittencourt MS (2021). Cardiovascular Risk Misperception and Low Awareness of Familial Hypercholesterolemia in Individuals with Severe Hypercholesterolemia. Arq Bras Cardiol.

[291] Lotufo PA, Santos RD, Figueiredo RM, Pereira AC, Mill JG, Alvim SM, Fonseca MJ, Almeida MC, Molina MC, Chor D, Schmidt MI, Ribeiro ALP, Duncan BB, Bensenor IM (2016). Prevalence, Awareness, Treatment, and Control of High Low-Density Lipoprotein Cholesterol in Brazil: Baseline of the Brazilian Longitudinal Study of Adult Health (ELSA-Brasil). J Clin Lipidol.

[292] Bernardi A, Olandoski M, Erbano LO, Guarita-Souza LC, Baena CP, Faria-Neto JR (2022). Achievement of LDL-Cholesterol Goals after Acute Myocardial Infarction: Real-World Data from the City of Curitiba Public Health System. Arq Bras Cardiol.

[293] Barreto J, Luchiari B, Wolf VLW, Bonilha I, Bovi TG, Assato BS, Breder I, Kimura-Medorima ST, Munhoz DB, Quinaglia T, Coelho OR Filho, Carvalho LSF, Nadruz W, Sposito AC (2022). Compliance with Cardiovascular Prevention Guidelines in Type 2 Diabetes Individuals in a Middle-Income Region: A Cross-Sectional Analysis. Diagnostics.

[294] Generoso G, Bensenor IM, Santos RD, Staniak HL, Sharovsky R, Santos IS, Goulart AC, Jones SR, Kulkarni KR, Blaha MJ, Toth PP, Lotufo PA, Bittencourt MS (2019). High-Density Lipoprotein-Cholesterol Subfractions and Coronary Artery Calcium: The ELSA-Brasil Study. Arch Med Res.

[295] Generoso G, Bensenor IM, Santos IS, Santos RD, Goulart AC, Jones SR, Kulkarni KR, Blaha MJ, Toth PP, Lotufo PA, Bittencourt MS (2018). Diabetes Alters the Association between High-Density Lipoprotein Subfractions and Carotid Intima-Media Thickness: The Brazilian Longitudinal Study of Adult Health (ELSA-Brasil). Diab Vasc Dis Res.

[296] Laurinavicius AG, Santos IS, Santos RD, Bensenor IM, Conceição RD, Lotufo PA (2016). Extremely Elevated HDL-Cholesterol Levels are Not Associated with Increased Carotid Intima-Media Thickness: Data from ELSA Brasil. J Clin Lipidol.

[297] Bittencourt MS, Santos RD, Staniak H, Sharovsky R, Kondapally R, Vallejo-Vaz AJ, Ray KK, Bensenor I, Lotufo P (2017). Relation of Fasting Triglyceride-Rich Lipoprotein Cholesterol to Coronary Artery Calcium Score (from the ELSA-Brasil Study). Am J Cardiol.

[298] Freitas WM, Quaglia LA, Santos SN, Paula RC, Santos RD, Blaha M, Rivera JJ, Cury R, Blumenthal R, Nadruz W, Agatston A, Figueiredo VN, Nasir K, Sposito AC, Brazilian Study on Healthy Aging (2015). Low HDL Cholesterol but not High LDL Cholesterol is Independently Associated with Subclinical Coronary Atherosclerosis in Healthy Octogenarians. Aging Clin Exp Res.

[299] Bahia LR, Rosa RS, Santos RD, Araujo DV (2018). Estimated Costs of Hospitalization Due to Coronary Artery Disease Attributable to Familial Hypercholesterolemia in the Brazilian Public Health System. Arch Endocrinol Metab.

[300] Kahn CR, Ferris HA, O'NEILL BT, Melmed S, Koenig R, Rosen C, Auchus R, Goldfine A (2019). Williams Textbook of Endocrinology.

[301] Kannel WB, McGee DL (1979). Diabetes and Cardiovascular Disease. The Framingham Study. JAMA.

[302] Stamler J, Vaccaro O, Neaton JD, Wentworth D (1993). Diabetes, Other Risk Factors, and 12-yr Cardiovascular Mortality for Men Screened in the Multiple Risk Factor Intervention Trial. Diabetes Care.

[303] International Diabetes Federation (2022). IDF Diabetes Atlas.

[304] Malta DC, Ribeiro EG, Gomes CS, Alves FTA, Stopa SR, Sardinha LMV, Pereira CA, Duncan BB, Schimidt MI (2022). Indicators of the Line of Care for People with Diabetes in Brazil: National Health Survey 2013 and 2019. Epidemiol Serv Saude.

[305] Santos KBM, Reis RCP, Duncan BB, D'Avila OP, Schmidt MI (2023). Access to Diabetes Diagnosis in Brazil Based on Recent Testing and Consultation: The Brazilian National Health Survey, 2013 and 2019. Front Endocrinol.

[306] Telo GH, Cureau FV, Souza MS, Andrade TS, Copês F, Schaan BD (2016). Prevalence of Diabetes in Brazil Over Time: A Systematic Review with Meta-Analysis. Diabetol Metab Syndr.

[307] Iser BPM, Pinheiro PC, Malta DC, Duncan BB, Schmidt MI (2021). Prediabetes and Intermediate Hyperglycemia Prevalence in Adults and Associated Factors, Health National Survey. Cien Saude Colet.

[308] Malta DC, Bernal RTI, Sá ACMGN, Silva TMRD, Iser BPM, Duncan BB, Schimdt MI (2022). Self-Reported Diabetes and Factors Associated with it in the Brazilian Adult Population: National Health Survey, 2019. Cien Saude Colet.

[309] Lopez-Jaramillo P, Joseph P, Lopez-Lopez JP, Lanas F, Avezum A, Diaz R, Camacho PA, Seron P, Oliveira G, Orlandini A, Rangarajan S, Islam S, Yusuf S (2022). Risk Factors, Cardiovascular Disease, and Mortality in South America: A PURE Substudy. Eur Heart J.

[310] Teló GH, Cureau FV, Szklo M, Bloch KV, Schaan BD (2019). Prevalence of Type 2 Diabetes among Adolescents in Brazil: Findings from Study of Cardiovascular Risk in Adolescents (ERICA). Pediatr Diabetes.

[311] Schmidt MI, Bracco PA, Yudkin JS, Bensenor IM, Griep RH, Barreto SM, Castilhos CD, Duncan BB (2019). Intermediate Hyperglycaemia to Predict Progression to Type 2 Diabetes (ELSA-Brasil): An Occupational Cohort Study in Brazil. Lancet Diabetes Endocrinol.

[312] Diamond Project Group (2006). Incidence and Trends of Childhood Type 1 Diabetes Worldwide 1990-1999. Diabet Med.

[313] Patterson CC, Harjutsalo V, Rosenbauer J, Neu A, Cinek O, Skrivarhaug T, Rami-Merhar B, Soltesz G, Svensson J, Parslow RC, Castell C, Schoenle EJ, Bingley PJ, Dahlquist G, Jarosz-Chobot PK, Marciulionyte D, Roche EF, Rothe U, Bratina N, Ionescu-Tirgoviste C, Weets I, Kocova M, Cherubini V, Rojnic Putarek N, Beaufort CE, Samardzic M, Green A (2019). Trends and Cyclical Variation in the Incidence of Childhood Type 1 Diabetes in 26 European Centres in the 25 Year Period 1989-2013: A Multicentre Prospective Registration Study. Diabetologia.

[314] Negrato CA, Lauris JRP, Saggioro IB, Corradini MCM, Borges PR, Crês MC, Leal A, Guedes MFS, Gomes MB (2017). Increasing Incidence of Type 1 Diabetes between 1986 and 2015 in Bauru, Brazil. Diabetes Res Clin Pract.

[315] Cardoso LSM, Teixeira RA, Ribeiro ALP, Malta DC (2021). Premature Mortality Due to Non-Communicable Diseases in Brazilian Municipalities Estimated for the Three-Year Periods of 2010 to 2012 and 2015 to 2017. Rev Bras Epidemiol.

[316] Cousin E, Schmidt MI, Stein C, Aquino ÉC, Gouvea ECDP, Malta DC, Naghavi M, Duncan BB (2022). Premature Mortality Due to Four Main Non-Communicable Diseases and Suicide in Brazil and its States from 1990 to 2019: A Global Burden of Disease Study. Rev Soc Bras Med Trop.

[317] Arrais KR, Máximo LWM, Rodrigues ASA, Silva MSG, Sousa SS, Araujo ACA (2022). Hospitalizations and Deaths by Diabetes Mellitus. Rev Pesqui.

[318] GBD 2021 Diabetes Collaborators (2023). Global, Regional, and National Burden of Diabetes from 1990 to 2021, with Projections of Prevalence to 2050: A Systematic Analysis for the Global Burden of Disease Study 2021. Lancet.

[319] Rodrigues MR, Alvarez AM, Pereira KCR (2022). Hospitalizations of the Elderly Due to Conditions Sensitive to Primary Care in the State of Santa Catarina. Ob Clin Res.

[320] Mosenzon O, Alguwaihes A, Leon JLA, Bayram F, Darmon P, Davis TME, Dieuzeide G, Eriksen KT, Hong T, Kaltoft MS, Lengyel C, Rhee NA, Russo GT, Shirabe S, Urbancova K, Vencio S, CAPTURE Study Investigators (2021). CAPTURE: A Multinational, Cross-Sectional Study of Cardiovascular Disease Prevalence in Adults with Type 2 Diabetes Across 13 Countries. Cardiovasc Diabetol.

[321] Gomes MB, Negrato CA (2016). Adherence to Insulin Therapeutic Regimens in Patients with Type 1 Diabetes. A Nationwide Survey in Brazil. Diabetes Res Clin Pract.

[322] Meiners MMMA, Tavares NUL, Guimarães LSP, Bertoldi AD, Pizzol TDSD, Luiza VL, Mengue SS, Merchan-Hamann E (2017). Access and Adherence to Medication among People with Diabetes in Brazil: Evidences from PNAUM. Rev Bras Epidemiol.

[323] Marinho FS, Moram CBM, Rodrigues PC, Leite NC, Salles GF, Cardoso CRL (2018). Treatment Adherence and Its Associated Factors in Patients with Type 2 Diabetes: Results from the Rio de Janeiro Type 2 Diabetes Cohort Study. J Diabetes Res.

[324] Silva MRRD, Diniz LM, Santos JBRD, Reis EA, Mata ARD, Araújo VE, Álvares J, Acurcio FA (2018). Drug Utilization and Factors Associated with Polypharmacy in Individuals with Diabetes Mellitus in Minas Gerais, Brazil. Cien Saude Colet.

[325] Leitão VBG, Francisco PMSB, Malta DC, Costa KS (2021). Tendency of use and Sources for Obtaining Oral Antidiabetic Drugs for Treatment of Diabetes in Brazil from 2012 to 2018: Analysis of the Vigitel Survey. Rev Bras Epidemiol.

[326] Schneiders J, Telo GH, Bottino LG, Pasinato B, Neyeloff JL, Schaan BD (2019). Quality Indicators in type 2 Diabetes Patient Care: Analysis Per Care-Complexity Level. Diabetol Metab Syndr.

[327] Macedo AM, Macedo JPS, Cardoso TIA, Moraga LMVM, Lima GM, Nogami ASA (2022). Analysis of the Reasons for Hospitalization of Elderly People in the Extreme North of Brazil. Medicina.

[328] Moreira RO, Vianna AGD, Ferreira GC, Paula MA (2022). Determinants of Glycemic Control in Type 2 Diabetes Mellitus in Brazil: A Sub-Analysis of the Longitudinal Data from the BrazIian Type 1 & 2 Diabetes Disease Registry (BINDER). Prim Care Diabetes.

[329] Chwal BC, Dos Reis RCP, Schmidt MI, Duncan BB, Barreto SM, Griep RH (2023). Levels and Correlates of Risk Factor Control in Diabetes Mellitus -ELSA-Brasil. Diabetol Metab Syndr.

[330] Sbaraini M, Cureau FV, Ritter JDA, Schuh DS, Madalosso MM, Zanin G, Goulart MR, Pellanda LC, Schaan BD (2021). Prevalence of Overweight and Obesity among Brazilian Adolescents Over Time: A Systematic Review and Meta-Analysis. Public Health Nutr.

[331] Sbaraini M, Cureau FV, Sparrenberger K, Teló GH, Kuschnir MCC, Oliveira JS, Leal VS, Bloch KV, Schaan BD (2020). Severity of Obesity is Associated with Worse Cardiometabolic Risk Profile in Adolescents: Findings from a Brazilian National Study (ERICA). Nutrition.

[332] Lin X, Alvim SM, Simoes EJ, Bensenor IM, Barreto SM, Schmidt MI, Ribeiro ALP, Pitanga F, Almeida MC, Liu S, Lotufo PA (2016). Leisure Time Physical Activity and Cardio-Metabolic Health: Results from the Brazilian Longitudinal Study of Adult Health (ELSA-Brasil). J Am Heart Assoc.

[333] Oliveira BBR, Coelho CG, Barreto SM, Giatti L, Araújo LF (2022). Body Fat Distribution and its Risk for Cardiovascular Events in 10 Years: Brazilian Longitudinal Study of Adult Health (ELSA-Brasil). Cad Saude Publica.

[334] Barboza LLS, Werneck AO, Araujo RHO, Porto LGG, Silva DR (2022). Multimorbidity is Associated with TV-Viewing, but Not With Other Types of Screen-Based Behaviors in Brazilian Adults. BMC Public Health.

[335] Araújo RHO, Werneck AO, Barboza LL, Silva ECM, Silva DR (2022). The Moderating Effect of Physical Activity on the Association between Screen-Based Behaviors and Chronic Diseases. Sci Rep.

[336] Riboldi BP, Luft VC, Bracco PA, Oliveira Cardoso L, Molina MDC, Alvim S, Giatti L, Schmidt MI, Duncan BB (2022). The Inflammatory Food Index and its Association with Weight Gain and Incidence of Diabetes: Longitudinal Study of Adult Health (ELSA-Brasil). Nutr Metab Cardiovasc Dis.

[337] Ferrari G, Giannichi B, Resende B, Paiva L, Rocha R, Falbel F, Rache B, Adami F, Rezende LFM (2022). The Economic Burden of Overweight and Obesity in Brazil: Perspectives for the Brazilian Unified Health System. Public Health.

[338] Leal JSV, Fogal AS, Meireles AL, Cardoso LO, Machado ÍE, Menezes MC (2022). Health Economic Impacts Associated with the Consumption of Sugar-Sweetened Beverages in Brazil. Front Nutr.

[339] Pereda P, Boarati V, Guidetti B, Duran AC (2022). Direct and Indirect Costs of Diabetes in Brazil in 2016. Ann Glob Health.

[340] Niquini RP, Lana RM, Pacheco AG, Cruz OG, Coelho FC, Carvalho LM, Villela DAM, Gomes MFDC, Bastos LS (2020). Description and Comparison of Demographic Characteristics and Comorbidities in SARI from COVID-19, SARI from Influenza, and the Brazilian General Population. Cad Saude Publica.

[341] Nunes AFC, Rezende EP, Lima JO, Presta MCF, Brandão MC, Purificação SMO (2021). As Doenças Crônicas Não Transmissíveis no Contexto da Pandemia da Covid-19 no Estado da Bahia. Rev Baiana Saúde Pública.

[342] Silva NJ, Ribeiro-Silva RC, Ferreira AJF, Teixeira CSS, Rocha AS, Alves FJO, Falcão IR, Pinto EJ, Santos CAST, Fiaccone RL, Ichihara MYT, Paixão ES, Barreto ML (2021). Combined Association of Obesity and Other Cardiometabolic Diseases with Severe COVID-19 Outcomes: A Nationwide Cross-Sectional Study of 21.773 Brazilian Adult and Elderly Inpatients. BMJ Open.

[343] Prado PRD, Gimenes FRE, Lima MVM, Prado VBD, Soares CP, Amaral TLM (2021). Risk Factors for Death Due to COVID-19, in the State of Acre, Brazil, 2020: A Retrospective Cohort Study. Epidemiol Serv Saude.

[344] Pietre E, Amorim GP, Bittecount MFF, Ribeiro-Alves M, Acquarone M (2021). Prevalent Comorbidities among Young and Underprivileged: Death Portrait of COVID-19 among 235 555 Hospitalized Patients in Brazil. medRxiv.

[345] Garces TS, Sousa GJB, Cestari VRF, Florêncio RS, Damasceno LLV, Pereira MLD, Moreira TMM (2022). Diabetes as a Factor Associated with Hospital Deaths Due to COVID-19 in Brazil, 2020. Epidemiol Serv Saude.

[346] Oliveira EA, Mak RH, Colosimo EA, Mendonça ACQ, Vasconcelos MA, Martelli H, Silva LR, Oliveira MCL, Pinhati CC, Silva ACS (2022). Risk Factors for COVID-19-Related Mortality in Hospitalized Children and Adolescents with Diabetes Mellitus: An Observational Retrospective Cohort Study. Pediatr Diabetes.

[347] Sardinha DM, Lima KVB, Ferreira ALS, Garcez JCD, Ueno TMRL, Rodrigues YC, Santos ALS, Loiola RSP, Guimarães RJPS, Lima LNGC (2021). Clinical and Spatial Characteristics of Severe Acute Respiratory Syndrome by COVID-19 in Indigenous of Brazil. Emerg Infect Dis.

[348] Andrade CM, Geumaro EA, Borges FA, Ângelo CFJ (2022). Desfecho Clínico de Pessoas com Diabetes Infectadas pelo SARS-CoV-2 que Desenvolveram Síndrome Respiratória Aguda Grave, Brasil. Medicina.

[349] Foppa L, Alessi J, Nemetz B, Matos R, Telo GH, Schaan BD (2022). Quality of Care in Patients with Type 1 Diabetes During the COVID-19 Pandemic: A Cohort Study from Southern Brazil. Diabetol Metab Syndr.

[350] Khan SS, Ning H, Sinha A, Wilkins J, Allen NB, Vu THT, Berry JD, Lloyd-Jones DM, Sweis R (2021). Cigarette Smoking and Competing Risks for Fatal and Nonfatal Cardiovascular Disease Subtypes Across the Life Course. J Am Heart Assoc.

[351] Centers for Disease Control and Prevention (2019). Electronic Nicotine delivery Systems Key Facts.

[352] Menezes AMB, Wehrmeister FC, Sardinha LMV, Paula PDCB, Costa TA, Crespo PA, Hallal PC (2023). Use of Electronic Cigarettes and Hookah in Brazil: A New and Emerging Landscape. The Covitel study, 2022. J Bras Pneumol.

[353] Instituto Brasileiro de Geografia e Estatística (2022). Pesquisa Nacional de Saúde do Escolar: Análise de Indicadores Comparáveis dos Escolares do 9º ano do Ensino Fundamental: Municípios das Capitais: 2009/2019.

[354] Pichon-Riviere A, Bardach A, Rodríguez Cairoli F, Casarini A, Espinola N, Perelli L, Reynales-Shigematsu LM, Llorente B, Pinto M, Juárez BSM, Villacres T, Peña Torres E, Amador N, Loza C, Castillo-Riquelme M, Roberti J, Augustovski F, Alcaraz A, Palacios A (2023). Health, Economic and Social Burden of Tobacco in Latin America and the Expected Gains of Fully Implementing Taxes, Plain Packaging, Advertising Bans and Smoke-Free Environments Control Measures: A Modelling Study. Tob Control.

[355] Cunha SB, Araújo RC, Oliveira JVB, Mola R, Pitangui ACR (2020). Factors Associated with Current Tobacco Use among Adolescents and Young Students. J Pediatr.

[356] Carvalho AM, Bertoni N, Coutinho C, Bastos FI, Fonseca VM (2023). Tobacco Use by Sexual and Gender Minorities: Findings from a Brazilian National Survey. BMJ Open.

[357] Feeney S, Rossetti V, Terrien J (2022). E-Cigarettes-a Review of the Evidence-Harm versus Harm Reduction. Tob Use Insights.

[358] Choi H, Lin Y, Race E, Macmurdo MG (2021). Electronic Cigarettes and Alternative Methods of Vaping. Ann Am Thorac Soc.

[359] Caponnetto P (2021). Well-Being and Harm Reduction, the Consolidated Reality of Electronic Cigarettes Ten Years Later from this Emerging Phenomenon: A Narrative Review. Health Psychol Res.

[360] Agência Nacional de Vigilância Sanitária (2009). Resolução nº 46, de 28 de agosto de 2009. Proíbe a Comercialização, a Importação e a Propaganda de Quaisquer Dispositivos Eletrônicos para Fumar, Conhecidos como Cigarro Eletrônico.

[361] Bertoni N, Cavalcante TM, Souza MC, Szklo AS (2021). Prevalence of Electronic Nicotine Delivery Systems and Waterpipe Use in Brazil: Where are we Going?. Rev Bras Epidemiol.

[362] Malta DC, Gomes CS, Vasconcelos NM, Alves FTA, Ferreira APS, Barros MBA, Lima MG, Szwarcwald CL (2023). Smoking among Brazilian Adolescents During the COVID-19 Pandemic: A Cross-Sectional Study. Sao Paulo Med J.

[363] Vital Strategies Brasil (2022). Inquérito Telefônico de Fatores de Risco para Doenças Crônicas em Tempos de Pandemia - Covitel.

[364] Malta DC, Gomes CS, Souza PRB, Szwarcwald CL, Barros MBA, Machado ÍE, Romero DE, Lima MG, Silva AGD, Prates EJS, Cardoso LSM, Damacena GN, Werneck AO, Silva DRPD, Azevedo LO (2021). Factors Associated with Increased Cigarette Consumption in the Brazilian Population During the COVID-19 Pandemic. Cad Saude Publica.

[365] Malta DC, Gomes CS, Barros MBA, Lima MG, Silva AGD, Cardoso LSM, Werneck AO, Silva DRPD, Ferreira APS, Romero DE, Freitas MIF, Machado ÍE, Souza PRB, Damacena GN, Azevedo LO, Almeida WDS, Szwarcwald CL (2021). The COVID-19 Pandemic and Changes in the Lifestyles of Brazilian Adolescents. Rev Bras Epidemiol.

[366] Szklo AS, Drope J (2023). The Cigarette Market in Brazil: New Evidence on Illicit Practices from the 2019 National Health Survey. Tob Control.

[367] Divino JA, Ehrl P, Candido O, Valadao MAP (2022). Extended Cost-Benefit Analysis of Tobacco Taxation in Brazil. Tob Control.

[368] World Health Organization (2021). WHO Health Emergency Dashboard.

[369] Souza LE, Rasella D, Barros R, Lisboa E, Malta D, Mckee M (2021). Smoking Prevalence and Economic Crisis in Brazil. Rev Saude Publica.

[370] Szklo AS, Iglesias RM (2020). Decrease in the Proportion of Illicit Cigarette use in Brazil: What does it Really Mean?. Tob Control.

[371] World Health Organization (2022). Obesity.

[372] Gaspar RS, Rezende LFM, Laurindo FRM (2022). Analysing the Impact of Modifiable Risk Factors on Cardiovascular Disease Mortality in Brazil. PLoS One.

[373] Brasil, Ministério da Saúde (2020). Pesquisa Nacional de Saúde: 2019: Percepção do Estado de Saúde, Estilos de Vida, Doenças Crônicas e Saúde Bucal - Brasil e Grandes Regiões.

[374] Assis MM, Gratão LHA, Silva TPR, Cordeiro NG, Carmo AS, Freitas Cunha C, Oliveira TRPR, Rocha LL, Mendes LL (2022). School Environment and Obesity in Adolescents from a Brazilian Metropolis: Cross-Sectional Study. BMC Public Health.

[375] Ferreira CM, Reis NDD, Castro AO, Höfelmann DA, Kodaira K, Silva MT, Galvao TF (2021). Prevalence of Childhood Obesity in Brazil: Systematic Review and Meta-Analysis. J Pediatr.

[376] Matos SMA, Duncan BB, Bensenor IM, Mill JG, Giatti L, Molina MDCB, Fonseca MJM, Schmidt MI, Lotufo PA, Griep RH, Barreto SM, Almeida MDCC (2022). Incidence of Excess Body Weight and Annual Weight Gain in Women and Men: Results from the ELSA-Brasil Cohort. Am J Hum Biol.

[377] Vaduganathan M, Mensah GA, Turco JV, Fuster V, Roth GA (2022). The Global Burden of Cardiovascular Diseases and Risk: A Compass for Future Health. J Am Coll Cardiol.

[378] Garcia KC, Confortin SC, Meneghini V, d'Orsi E, Barbosa AR (2022). Metabolic Syndrome and its Association with Changes in Modifiable Risk Factors: Epifloripa Aging Study. J Diabetes Metab Disord.

[379] Oliveira CM, Rosa FF, Alvim RO, Mourão CA, Bacells M, Liu C, Pavani J, Capasso R, Lavezzo Dias FA, Krieger JE, Pereira AC (2022). Body Mass Index is Superior to Other Body Adiposity Indexes in Predicting Incident Hypertension in a Highly Admixed Sample after 10-Year Follow-Up: The Baependi Heart Study. J Clin Hypertens.

[380] Santiago LS, Martins PC, Silva DA (2022). Association between Excess Peripheral, Central and General Adiposity with High Blood Pressure in Adolescents in Southern Brazil. J Hum Growth Dev.

[381] Pereira JL, Castro MA, Leite JMRS, Rogero MM, Sarti FM, César CLG, Goldbaum M, Fisberg RM (2021). Overview of Cardiovascular Disease Risk Factors in Adults in São Paulo, Brazil: Prevalence and Associated Factors in 2008 and 2015. Int J Cardiovasc Sci.

[382] Zheng Z, Peng F, Xu B, Zhao J, Liu H, Peng J, Li Q, Jiang C, Zhou Y, Liu S, Ye C, Zhang P, Xing Y, Guo H, Tang W (2020). Risk Factors of Critical & Mortal COVID-19 Cases: A Systematic Literature Review and Meta-Analysis. J Infect.

[383] Yang J, Zheng Y, Gou X, Pu K, Chen Z, Guo Q, Ji R, Wang H, Wang Y, Zhou Y (2020). Prevalence of Comorbidities and its Effects in Patients Infected with SARS-CoV-2: A Systematic Review and Meta-Analysis. Int J Infect Dis.

[384] Noor FM, Islam MM (2020). Prevalence and Associated Risk Factors of Mortality among COVID-19 Patients: A Meta-Analysis. J Community Health.

[385] Paravidino VB, Leite TH, Mediano MFF, Sichieri R, Azevedo E Silva G, Cravo V, Balduino A, Salgueiro E, Besen BAMP, Moreira RC, Brandão CE, Gomes DCK, Assemany CAG, Cougo P (2022). Association between Obesity and COVID-19 Mortality and Length of Stay in Intensive Care Unit Patients in Brazil: A Retrospective Cohort Study. Sci Rep.

[386] Barros-Neto JA, Mello CS, Vasconcelos SML, Bádue GS, Ferreira RC, Andrade MIS, Nascimento CQD, Macena ML, Silva JAD, Clemente HA, Petribu MMV, Dourado KF, Pinho CPS, Vieira RAL, Mello LB, Neves MBD, Jesus CA, Santos TMPD, Soares BLM, Medeiros LB, França AP, Sales ALCC, Furtado EVH, Oliveira AC, Farias FO, Freitas MC, Bueno NB (2022). Association between Being Underweight and Excess Body Weight Before SARS Coronavirus Type 2 Infection and Clinical Outcomes of Coronavirus Disease 2019: Multicenter Study. Nutrition.

[387] Vera-Zertuche JM, Mancilla-Galindo J, Tlalpa-Prisco M, Aguilar-Alonso P, Aguirre-García MM, Segura-Badilla O, Lazcano-Hernández M, Rocha-González HI, Navarro-Cruz AR, Kammar-García A, Vidal-Mayo JJ (2021). Obesity is a Strong Risk Factor for Short-Term Mortality and Adverse Outcomes in Mexican Patients with COVID-19: A National Observational Study. Epidemiol Infect.

[388] Malta DC, Gomes CS, Prates EJS, Bernal RTI (2023). Changes in Chronic Diseases and Risk and Protective Factors Before and after the Third Wave of COVID-19 in Brazil. Cien Saude Colet.

[389] Caspersen CJ, Powell KE, Christenson GM (1985). Physical Activity, Exercise, and Physical Fitness: Definitions and Distinctions for Health-Related Research. Public Health Rep.

[390] Thivel D, Tremblay A, Genin PM, Panahi S, Rivière D, Duclos M (2018). Physical Activity, Inactivity, and Sedentary Behaviors: Definitions and Implications in Occupational Health. Front Public Health.

[391] Dasso NA (2019). How is Exercise Different from Physical Activity? A Concept Analysis. Nurs Forum.

[392] Yang YJ (2019). An Overview of Current Physical Activity Recommendations in Primary Care. Korean J Fam Med.

[393] González K, Fuentes J, Márquez JL (2017). Physical Inactivity, Sedentary Behavior and Chronic Diseases. Korean J Fam Med.

[394] Park JH, Moon JH, Kim HJ, Kong MH, Oh YH (2020). Sedentary Lifestyle: Overview of Updated Evidence of Potential Health Risks. Korean J Fam Med.

[395] Bull FC, Al-Ansari SS, Biddle S, Borodulin K, Buman MP, Cardon G, Carty C, Chaput JP, Chastin S, Chou R, Dempsey PC, DiPietro L, Ekelund U, Firth J, Friedenreich CM, Garcia L, Gichu M, Jago R, Katzmarzyk PT, Lambert E, Leitzmann M, Milton K, Ortega FB, Ranasinghe C, Stamatakis E, Tiedemann A, Troiano RP, van der Ploeg HP, Wari V, Willumsen JF (2020). World Health Organization 2020 Guidelines on Physical Activity and Sedentary Behaviour. Br J Sports Med.

[396] Piercy KL, Troiano RP, Ballard RM, Carlson SA, Fulton JE, Galuska DA, George SM, Olson RD (2018). The Physical Activity Guidelines for Americans. JAMA.

[397] Brasil, Ministério da Saúde (2023). Guia de Atividade Física para a População Brasileira.

[398] Sainani A (2022). Commentary: Screening of Screen Time in Children. Indian J Ophthalmol.

[399] Pitanga FJG, Almeida MCC, Queiroz CO, Aquino EML, Matos SMA (2017). Physical Activity in Brazil: Lessons from ELSA-Brasil. Narrative Review. Sao Paulo Med J.

[400] Brasil, Ministério da Saúde (2022). VIGITEL Brasil 2006-2020. Vigilância de Fatores de Risco e Proteção para Doenças Crônicas por Inquérito Telefônico.

[401] Mielke GI, Stopa SR, Gomes CS, Silva AGD, Alves FTA, Vieira MLFP, Malta DC (2021). Leisure Time Physical Activity among Brazilian Adults: National Health Survey 2013 and 2019. Rev Bras Epidemiol.

[402] Ferreira ACM, Silva AG, Sá ACMG, Oliveira PPV, Felisbino-Mendes MS, Pereira CA, Malta DM (2022). Risk and Protection Factors for Chronic Non-Communicable Diseases among Brazilian Students: 2015 and 2019 National School Health Survey. Rev Min Enferm.

[403] Guthold R, Stevens GA, Riley LM, Bull FC (2018). Worldwide Trends in Insufficient Physical Activity from 2001 to 2016: A Pooled Analysis of 358 Population-Based Surveys with 1·9 Million Participants. Lancet Glob Health.

[404] Nascimento BR, Brant LCC, Naback ADN, Veloso GA, Polanczyk CA, Ribeiro ALP, Malta DC, Ferreira AVL, Oliveira GMM (2022). Burden of Cardiovascular Diseases Attributable to Risk Factors in Portuguese-Speaking Countries: Data from the "Global Burden of Disease 2019" Study. Arq Bras Cardiol.

[405] Katzmarzyk PT, Friedenreich C, Shiroma EJ, Lee IM (2022). Physical Inactivity and Non-Communicable Disease Burden in Low-Income, Middle-Income and High-Income Countries. Br J Sports Med.

[406] Xu YY, Xie J, Yin H, Yang FF, Ma CM, Yang BY, Wan R, Guo B, Chen LD, Li SL (2022). The Global Burden of Disease Attributable to Low Physical Activity and its Trends from 1990 to 2019: An Analysis of the Global Burden of Disease Study. Front Public Health.

[407] Bielemann RM, Silva BG, Coll CV, Xavier MO, Silva SG (2015). Burden of Physical Inactivity and Hospitalization Costs Due to Chronic Diseases. Rev Saude Publica.

[408] Araújo MYC, Barros MVG, Ricardo SJ, Mantovani AM, Turi-Lynch BC, Codogno JS (2022). Productivity Loss, Healthcare Costs, and Habitual Physical Activity among Adults with Cardiovascular Diseases. J Occup Environ Med.

[409] Santos AC, Willumsen J, Meheus F, Ilbawi A, Bull FC (2023). The Cost of Inaction on Physical Inactivity to Public Health-Care Systems: A Population-Attributable Fraction Analysis. Lancet Glob Health.

[410] Silva DRPD, Werneck AO, Malta DC, Souza PRB, Azevedo LO, Barros MBA, Szwarcwald CL (2021). Changes in the Prevalence of Physical Inactivity and Sedentary Behavior During COVID-19 Pandemic: A Survey with 39,693 Brazilian Adults. Cad Saude Publica.

[411] Silva DR, Werneck AO, Malta DC, Souza PRB, Azevedo LO, Barros MBA, Szwarcwald CL (2021). Incidence of Physical Inactivity and Excessive Screen Time During the First Wave of the COVID-19 Pandemic in Brazil: What are the Most Affected Population Groups?. Ann Epidemiol.

[412] Faria TMTR, Silva AG, Claro RM, Malta DC (2023). Time Trends and COVID-19 Post-Pandemic Changes in Physical Activity and Sedentary Behavior Prevalence among Brazilian Adults between 2006 and 2021. Rev Bras Epidemiol.

[413] Schuch FB, Bulzing RA, Meyer J, López-Sánchez GF, Grabovac I, Willeit P, Vancampfort D, Caperchione CM, Sadarangani KP, Werneck AO, Ward PB, Tully M, Smith L (2022). Moderate to Vigorous Physical Activity and Sedentary Behavior Changes in Self-Isolating Adults During the COVID-19 Pandemic in Brazil: A Cross-Sectional Survey Exploring Correlates. Sport Sci Health.

[414] Lee IM, Shiroma EJ, Lobelo F, Puska P, Blair SN, Katzmarzyk PT, Lancet Physical Activity Series Working Group (2012). Effect of Physical Inactivity on Major Non-Communicable Diseases Worldwide: An Analysis of Burden of Disease and Life Expectancy. Lancet.

[415] World Health Organization (2022). Global Status Report on Physical Activity 2022: Let's get moving!.

[416] Jeong SW, Kim SH, Kang SH, Kim HJ, Yoon CH, Youn TJ, Chae IH (2019). Mortality Reduction with Physical Activity in Patients with and without Cardiovascular Disease. Eur Heart J.

[417] Silva AGD, Prates EJS, Malta DC (2021). Evaluation of Community Physical Activity Programs in Brazil: A Scoping Review. Cad Saude Publica.

