# Supplementary material for: Estatística Cardiovascular – Brasil 2023
Source: Arq Bras Cardiol. 2024 Feb 15;121(2):e20240079. [Article in Portuguese] doi: 10.36660/abc.20240079 (PMC11185831; doi:10.36660/abc.20240079)
Supplement: Translation

# Translation: Cardiovascular Statistics – Brazil 2023

**Authors:** Gláucia Maria Moraes de Oliveira, Luisa Campos Caldeira Brant, Carisi Anne Polanczyk, Deborah Carvalho Malta, Andreia Biolo, Bruno Ramos Nascimento, Maria de Fatima Marinho de Souza, Andrea Rocha De Lorenzo, Antonio Aurélio de Paiva Fagundes, Beatriz D. Schaan, Christina Grüne de Souza e Silva, Fábio Morato de Castilho, Fernando Henpin Yue Cesena, Gabriel Porto Soares, Gesner Francisco Xavier, Jose Augusto Soares Barreto, Luiz Guilherme Passaglia, Marcelo Martins Pinto, M. Julia Machline-Carrion, Marcio Sommer Bittencourt, Octavio M. Pontes, Paolo Blanco Villela, Renato Azeredo Teixeira, Ricardo Stein, Roney Orismar Sampaio, Thomaz A. Gaziano, Pablo Perel, Gregory A. Roth, Antonio Luiz Pinho Ribeiro

**Affiliations:** 1 Rio de Janeiro RJ Brasil Instituto do Coração Edson Saad da Universidade Federal do Rio de Janeiro (UFRJ), Rio de Janeiro, RJ – Brasil; 2 Rio de Janeiro RJ Brasil Universidade Federal do Rio de Janeiro (UFRJ), Rio de Janeiro, RJ – Brasil; 3 Belo Horizonte MG Brasil Universidade Federal de Minas Gerais (UFMG), Belo Horizonte, MG – Brasil; 4 Belo Horizonte MG Brasil Hospital das Clínicas da Universidade Federal de Minas Gerais (UFMG), Belo Horizonte, MG – Brasil; 5 Porto Alegre RS Brasil Universidade Federal do Rio Grande do Sul (UFRGS), Porto Alegre, RS – Brasil; 6 Porto Alegre RS Brasil Hospital Moinhos de Vento, Porto Alegre, RS – Brasil; 7 Porto Alegre RS Brasil Hospital de Clínicas de Porto Alegre (HCPA), Porto Alegre, RS – Brasil; 8 Belo Horizonte MG Brasil Hospital Madre Teresa, Belo Horizonte, MG – Brasil; 9 New York EUA Vital Strategies,New York – EUA; 10 Rio de Janeiro RJ Brasil Instituto Nacional de Cardiologia, Rio de Janeiro, RJ – Brasil; 11 Brasília Brasil Instituto D’Or de Pesquisa e Ensino (IDOR), Brasília – Brasil; 12 Brasília Brasil Universidade de Brasília (UNB), Brasília – Brasil; 13 Brasília Brasil Hospital DFStar, Rede DO´r, Brasília – Brasil; 14 São Paulo SP Brasil Instituto Dante Pazzanese de Cardiologia, São Paulo, SP – Brasil; 15 Vassouras RJ Brasil Curso de Medicina da Universidade de Vassouras, Vassouras, RJ – Brasil; 16 Aracaju SE Brasil Universidade Federal de Sergipe, Aracaju, SE – Brasil; 17 Aracaju SE Brasil Hospital São Lucas Rede São Luiz D’Or, Aracaju, SE – Brasil; 18 Londres Reino Unido epHealth UK, Londres – Reino Unido; 19 São Paulo SP Brasil Instituto epHealth, São Paulo, SP – Brasil; 20 Pittsburgh EUA Department of Medicine and Radiology University of Pittsburgh, Pittsburgh – EUA; 21 São Paulo SP Brasil Faculdade de Medicina de Ribeirão Preto da Universidade de São Paulo (USP), São Paulo, SP – Brasil; 22 São Paulo SP Brasil Faculdade de Medicina da Universidade de São Paulo (USP), São Paulo, SP – Brasil; 23 São Paulo SP Brasil Instituto do Coração (Incor) do Hospital das Clínicas da Faculdade de Medicina da Universidade de São Paulo (HCFMUSP), São Paulo, SP – Brasil; 24 Boston EUA Division of Cardiovascular Medicine, Brigham and Women’s Hospital, Boston – EUA; 25 Boston EUA Department of Health Policy and Management, Harvard T.H. Chan School of Public Health, Boston – EUA; 26 Geneva Suíça World Heart Federation, Geneva – Suíça; 27 Londres Inglaterra Centre for Global Chronic Conditions, London School of Hygiene & Tropical Medicine, Londres – Inglaterra; 28 Washington EUA Division of Cardiology, Department of Medicine, University of Washington, Washington – EUA

**Keywords:** Cardiovascular Diseases, Cerebrovascular Disorders, Coronary Disease, Cardiomyopathies, Heart Valve Diseases, Heart Failure, Atrial Fibrillation, Atrial Flutter, Hypertension, Dyslipidemias, Diabetes Mellitus, Tobacco Use Disorder, Obesity, Overweight, Exercise, Statistics, Brazil

## ABOUT THESE STATISTICS

**Table:** Abbreviations Used in this Introduction

ACS	Acute Coronary Syndrome
AF	Atrial Fibrillation
CABG	Coronary Artery Bypass Grafting
CCS	Chronic Coronary Syndrome
ChD	Chagas Disease
CHD	Coronary Heart Disease
CI	Confidence Interval
CINAHL	Cumulative Index to Nursing and Allied Health Literature
COVID-19	New coronavirus disease 2019
CV	Cardiovascular
CVD	Cardiovascular Disease
DALY	Disability-Adjusted Life Year
DATASUS	Brazilian Unified Health System Database
DeCS	Descritores em Ciências da Saúde
ECG	Electrocardiogram
ELSA-Brasil	The Brazilian Longitudinal Study of Adult Health
FU	Federative Unit
GBD	Global Burden of Disease
GDP	Gross Domestic Product
IBGE	Brazilian Institute of Geography and Statistics (in Portuguese, Instituto Brasileiro de Geografia e Estatística)
ICD	International Statistical Classification of Diseases and Related Health Problems
IHME	Institute for Health Metrics and Evaluation
Int$	International dollars
IPCA	Brazilian Consumer Price Index (in Portuguese, Índice de Preços ao Consumidor Amplo)
LILACS	Literatura Latino-Americana e do Caribe em Ciências da Saúde
MEDLINE	Medical Literature Analysis and Retrievel System Online
MeSH	Medical Subject Headings
NCD	Non-Communicable Disease
NYHA	New York Heart Association
PCI	Percutaneous Coronary Intervention
PNS	National Health Survey (in Portuguese, Pesquisa Nacional de Saúde)
PPP	Purchasing Power Parity
R$	Reais, Brazilian currency
RECALL	The Brazilian Registry of Chronic Atrial Fibrillation
RHD	Rheumatic Heart Disease
SIH	Brazilian Hospital Information System (in Portuguese, Sistema de Informações Hospitalares)
SIM	Brazilian Mortality Information System (in Portuguese, Sistema de Informações sobre Mortalidade)
SUS	Brazilian Unified Health System (in Portuguese, Sistema Único de Saúde)
TTR	Time in Therapeutic Range
UI	Uncertainty Interval
US$	US dollars
VHD	Valvular heart disease
YLD	Year Lived with Disability
YLL	Year of Life Lost

The publication ***Cardiovascular Statistics – Brazil*** aims to provide an annual compilation of data and research on the epidemiology of CVDs in Brazil. The report integrates official statistics from the Brazilian Ministry of Health and other governmental entities alongside data from the GBD project, coordinated by the IHME at the University of Washington. Additionally, it incorporates data derived from various sources and scientific studies, including cohorts and registries, that relate to CVDs and their associated risk factors. This publication is intended for a wide range of individuals, including researchers, clinicians, patients, healthcare policymakers, media professionals, the general public, and other interested parties seeking extensive national data about heart disease and stroke. Volunteer researchers from various Brazilian universities and research institutions carry out the project. The group is led by a five-member steering committee (ALPR, CAP, DCM, GMMO, and LCCB). The Brazilian Society of Cardiology fully supports this initiative, and the project receives collaborative support from the GBD Brazil Network^1^and an International Committee (GAR, PP, and TAG) from both the IHME/University of Washington (GAR) and the World Heart Federation (PP and TAG).

The first ***Cardiovascular Statistics – Brazil*** was released in 2020^2^and included chapters on total CVD and five specific conditions: cerebrovascular disease, coronary heart disease, cardiomyopathy and heart failure, and atrial fibrillation. The publication follows the methodology used by the American Heart Association to produce the annual ***Heart Disease & Stroke Statistics Update*** ,^3^which also emphasizes epidemiological and public health data. The ***Cardiovascular Statistics – Brazil 2021*** version^4^included the most recent data on those diseases and all-new chapters on CV risk behaviors and factors, specifically hypertension, diabetes, dyslipidemia, obesity, and smoking and tobacco use. A companion website lets users get graphs and maps based on the available data. These two first versions of ***Cardiovascular Statistics – Brazil*** ^2 , 4^soon became a standard in the field and a natural source of information for stakeholders, health professionals, and researchers, being accessed more than 26 thousand times (only through the ABC Cardiol page) until November 2023.

The advent of the COVID-19 pandemic, which hit Brazil hard from February 2020 on and dominated the scene for the next two years, changed our lives and practices in a profound way. The number of deaths in Brazil reached the unbelievable toll of 700 000, and nearly 37 million persons were infected.^5^Health resources and research capacity in Brazil and everywhere were heavily directed to assist COVID-19 patients and better understand the disease and how to prevent and treat it. Since the beginning, it was clear that there was an interaction between COVID-19 and CVDs, because of not only the competition for health resources and mitigation policies that, although necessary, indirectly affected CV care, but also the possible interaction of the virus with the CV system, its prevention by vaccines, and some putative treatments.^6^However, we could not forecast that, as detailed in this version of the ***Cardiovascular Statistics - Brazil*** , COVID-19 would become the most important cause of death in Brazil in 2021, surpassing acute coronary syndrome and stroke. In this document, each chapter will cover the Brazilian data on the interaction of COVID-19 and CVDs and risk factors, highlighting the importance of the Brazilian research community in this field.

In addition to all these aspects widely discussed in scientific publications, conferences, and general media, the COVID-19 pandemic affected the timing and the way health data were collected and reported, causing delays and uncertainties in the statistics on mortality and disease burden in general. The co-existence and the higher lethality of COVID-19 in patients with chronic diseases, such as CVD, and the higher frequency of deaths occurring outside the hospital reduced the accuracy of the information on the cause of death, making the interpretation of the findings complex and challenging.^8 , 13^Epidemiologic studies were suspended or postponed, and the release of definite datasets from health authorities was delayed due to the strenuous work to give rapid information on the pandemic and the vaccination process. The IHME, which produces one of the main sources of the ***Cardiovascular Statistics – Brazil*** , has canceled the release of the GBD 2020, expected to be released in mid-2022, and has dedicated itself to estimating and forecasting data related to the pandemic.^16 , 17^Until now, we have not had a publicly available update on the latest version of the GBD 2019, and the publication of the GBD 2021 is estimated for early 2024.

The ***Cardiovascular Statistics – Brazil*** uses data from four different sources: (a) the Brazilian mortality and health information systems, provided by the government; (b) the most recent GBD estimates; (c) a systematic review of the literature with emphasis of what was published in the last 10 years; (d) healthcare utilization costs, based on the reimbursement Tables from the Public Health System. The absence of updated GBD estimates profoundly impacted our work: we had to cancel the release of the ***Cardiovascular Statistics – Brazil 2022*** . We decided to publish the ***Cardiovascular Statistics – Brazil 2023*** with the available data from the GBD study and other sources, adding (a) a new chapter on physical activity as a health habit related to CVDs, (b) sections on COVID-19 in specific CVDs and risk factors, and (c) a more detailed cost analysis. The ***Cardiovascular Statistics – Brazil 2024*** , to be released by the end of 2024, will show the full dataset regarding the GBD 2021, to be published in the first semester of 2024.

In addition, this ***Cardiovascular Statistics – Brazil 2023*** stresses that CVD is still responsible for nearly one-third of deaths in Brazil, disproportionally affecting the poorest *stratum* of its population, who has marked difficulties in access to high-quality health care.^18 , 19^COVID-19 exposed these inequalities, and the excess mortality was higher in the black/brown population^20^and excess CV mortality was greater in the less developed cities, possibly associated with healthcare collapse.^8^Having representative, reliable, and extensive national data on CVDs, risk behaviors, and factors is an obligatory step towards overcoming these inequalities and providing the best possible CV care to all Brazilians. This study gathers this information, which is essential to individual care and to plan the next steps of health policy in Brazil,^21^but also points out gaps in the knowledge to be filled with further studies. We all aspire for people to live longer and better, and knowing more about CV statistics to help confront CVDs is a good start to this goal.

### Brazilian Mortality and Health Information Systems

The main sources of Brazilian data for ***Cardiovascular Statistics – Brazil*** are morbidity and mortality information systems (see below), periodic health surveys, such as the National Health Survey, and official population estimates:

A. Brazilian Mortality Information System (SIM): The SIM is responsible for collecting, storing, managing, and disseminating national mortality data. The Brazilian Ministry of Health uses a Standard Certificate of Death model, a document for collecting information on death that uses the ICD to code the causes of death; in addition, a flow of collection, processing, and distribution of death information has been implemented in all 5570 municipalities across the country.^22 - 27^The quality of statistics on causes of death in Brazil significantly improved in the last two decades, but data from the beginning of the 2000 decade is still of low quality, specifically in some parts of the country.^28^Knowing the heterogeneity of these indicators in Brazil, the **Cardiovascular Statistics** - **Brazil** report treated data to estimate information closer to real, with the correction for underreporting and redistribution of ill-defined causes of death.^25^

B. Brazilian Hospital Information System (SIH): The SIH database registers all SUS-funded hospitalizations at the municipal level through the ‘Hospital Admission Authorization’, which has information about the diseases that led to hospitalization (using ICD-10), length of stay, procedures, and costs.^26^The SIH-SUS information permits the development of methodologies and the definition of indicators to identify geographical disparities related to hospital resources.^27^

C. National Health Survey (PNS): The PNS is a household-based epidemiological survey, representative of Brazil, its large regions, FUs, metropolitan regions, capitals, and other municipalities in each FU. The first version of the PNS was conducted in 2013 with a sample of 64 348 households. In 2019, a second survey was conducted with more than 94 114 households.^28^Most health topics were included, such as NCDs, renal function, elderly, women, children, use of health services, health inequalities, and physical and laboratory measurements, as well as blood pressure and anthropometric measurements.^29^

D. For population estimates, the most updated population estimates generated by the IBGE ( www.ibge.gov.br ) were used in the denominator. For the hospitalizations and cost analyses, we used the resident population estimated for the National Audit Office yearly from 2008 to 2021.

### Global Burden of Disease Study

The GBD Study ( http://www.healthdata.org/gbd ) is the most comprehensive worldwide observational epidemiological study to date. It describes mortality and morbidity from major diseases, injuries, and risk factors at global, national, and regional levels. Examining trends from 1990 to the present and comparing populations enable an understanding of the changing health challenges faced by people worldwide in the 21st century. The GBD Brazil network^1^has been collaborating with the IHME, from the University of Washington, that leads the project in the world in the identification and provision of datasets, revision of models and estimates, validation and publication of the results for Brazil.^30 , 31^Details on how the estimates are calculated can be obtained on the IHME website ( http://www.healthdata.org/acting-data/what-we-measure-and-why ). The GBD 2019 is the latest dataset publicly available.^32^In 2022, an update for CVDs with limited data updated to 2021 was released – these estimates were also used in our report.^37^We summarize below the main estimates used in this document:

A. Estimates of deaths and causes of deaths. The main source of information is the SIM, a database from the Brazilian Ministry of Health, adjusted to other national and international sources. The IHME used methods for correcting for underreporting of deaths and “garbage code” deaths according to previously published algorithms,^38^updated in the newer versions of the study ( http://www.healthdata.org/acting-data/determining-causes-death-how-we-reclassify-miscoded-deaths ).

B. YLLs are years lost due to premature mortality. The YLLs are calculated by subtracting the age at death from the longest possible life expectancy for a person at that age. For example, if the longest life expectancy for men in a given country is 75 years, but a man dies of cancer at 65 years, this would be 10 years of life lost due to cancer.

C. YLDs, years lived with disability, can also be described as years lived in less-than-ideal health. This includes conditions such as influenza, which may last for only a few days, or epilepsy, which can last a lifetime. It is measured by taking the prevalence of the condition multiplied by the disability weight for that condition. Disability weights reflect the severity of different conditions and are developed through surveys of the general public.

D. DALY is the abbreviation for disability-adjusted life year. It is a universal metric that allows researchers and policymakers to compare very different populations and health conditions across time. The DALYs equal the sum of YLLs and YLDs. One DALY equals one lost year of healthy life. DALYs allow us to estimate the total number of years lost due to specific causes and risk factors at the country, regional, and global levels.

### Systematic Review of the Literature

Descriptors for elaborating search strategies were selected in MeSH and DeCS, and controlled vocabularies were selected from MEDLINE and LILACS, respectively. Embase’s plan was designed with Emtree descriptors associated with MeSH. Free terms were also used, that is, significant keywords and their synonyms, spelling variations, and acronyms that are essential for searching in the searched domain, but which are not controlled descriptors (or are not in the synonym list of these descriptors). Importantly, the same descriptors were used in all search strategies to maintain search uniformity. However, search strategies were customized according to the specifics of each database. It is also noteworthy that the terms related to ‘Brazil’ were generally searched in all research fields (subject, author, title, institutional affiliation, journal name, etc.).

The selected bases for research were MEDLINE via PubMed, Embase, LILACS, CINAHL, Cochrane Library Scopus, and Web of Science. The following bibliographic research filters and limits were used: period of publication (2004-2022); languages: Portuguese, English, and Spanish; type of study/publication: Review, Meta-Analysis, Clinical Trial, Randomized Controlled Trial, Comparative Study, Practice Guideline, Guideline, Systematic Review, Evaluation Studies, Government Publications, and Multicenter Study. All references were managed using *EndNote Web* . From the search, articles were included if the studies were population- or community-based; nation- or state-wide studies were preferred. Moreover, articles set at health services or hospitals were included if the study was multicenter and had an adequate sample size (> 200 participants was the suggested cut-off), preferably. In addition to the articles identified by the systematic search, authors could include other studies found in the references of the searched articles or other articles they were aware of in their area of expertise, if the studies fulfilled the criteria above mentioned. Finally, which studies should be described in each chapter was mostly a decision of the experts commissioned to the specific theme.

### Health Care Utilization

Healthcare costing studies have expressive methodologic variability and, thus, need to be carefully interpreted. In the present document, most of the cost data were gathered from the Public Health System reimbursement databases from 2008 to 2021. During this period, payment adjustment for inflation was neither performed regularly nor was homogeneous across CVD groups or procedures. The Brazilian inflation rate (based on the IPCA) from 2008 to 2018 was 76.3%, and the mean inflation for CV procedures was 43.5%. For some procedure codes, the adjustment was minimal; for coronary stenting, for example, it was 8.7%. Other procedures, however, were adjusted above the inflation rate, such as the treatment of arrhythmias (83.4%).

To minimize biases in reporting and interpreting cost data, a systematic approach was applied to all chapters. Overall costing studies were described in original currency (R$ or US$ in a specific year) and Int$. International dollars were converted to PPP adjusted for US$ 2021 (Int$ 2021) using the Campbell and Cochrane Economics Methods Group Evidence for Policy and Practice Information and Coordination Centre cost methodology. A two-stage approach is applied in this method. First, it adjusts the original estimate of cost from the original price-year to a target price-year, using a GDP deflator index, which, for this report, was the IPCA (Brazilian inflation rate). Second, it converts the price-year-adjusted cost estimate from the original currency to a target currency, using conversion rates based on the PPP for GDP (PPP values).^39^For original economic studies, when the base year of the currency was not reported or could not be inferred from the manuscript (e.g., last year of data collection), the recommendation was to assume the year before the paper’s publication.

## HIGHLIGHTS

### Chapter 1 – Total Cardiovascular Diseases

•Cardiovascular diseases used to be the number 1 cause of death in Brazil, where, of all CVDs, CHD was the leading cause of death, followed by stroke, in 1990 and 2019. According to the SIM, in 2021, COVID-19 became the leading cause of death among men and women.

•The early years of the COVID-19 pandemic, 2020 and 2021, showed a significant reduction in hospital admissions for all types of CVDs. Some factors should be considered, such as the increased number of cardiovascular deaths outside the hospital, reduced search for medical care, and hospital admission with concurrent CVD and COVID-19 being recorded as the primary diagnosis.

•More than R$ 1 billion is spent annually on cardiovascular surgical procedures in Brazil by the SUS.

### Chapter 2 – Stroke (Cerebrovascular Diseases)

•Data from the GBD have shown that the age-standardized mortality rate per 100 000 from ischemic stroke in 2021 was 31.7 (28 to 33.9). The highest mortality was observed in the Maranhão state, 49.8 (42.8 to 56), and the lowest, in the Rio Grande do Norte state, 25 (21.1 to 27.9).

•Data from the GBD have shown that the age-standardized DALY rate per 100 000 due to ischemic stroke in 2021 was 511.4 (470.6 to 542.5). The highest DALY rate was observed in the Maranhão state, 759.8 (674.3 to 858), and the lowest, in the Rio Grande do Norte state, 407.1 (359.5 to 449.7).

•The costs related to stroke hospital admissions and adjusted for inflation from 2008 to 2021 sum up to US$ 1 195 975 877.98, varying from US$ 28 661 321.88, in 2008, to US$ 66 843 953.39, in 2021.

•In recent years, there have been several initiatives to improve awareness of stroke in the Brazil population, mainly around the World Stroke Day (October 29th) annual campaigns led by the World Stroke Organization. Despite these efforts, several studies have shown an alarming lack of knowledge about stroke risk factors and treatment, and recognition of stroke symptoms as a medical emergency.

### Chapter 3 – Acute and Chronic Coronary Heart Disease

•According to the GBD estimates for the year 2021, the age-standardized mortality rate due to CHD was 67.1 (95% UI, 60.9-71.0) per 100 000 inhabitants in Brazil.

•The COVID-19 pandemic substantially impacted CHD-related statistics in Brazil. Data from the SUS have shown that hospital admissions due to CCS and ACS declined 12.8% (95% CI, 12.5%-13.2%) and 13.6% (95% CI, 13.3%-13.9%), respectively, during the pandemic in relation to average values in the preceding years (2017-2019). In-hospital mortality and the proportion of home deaths related to both CCS and ACS increased during the pandemic.

•The number of PCIs performed in Brazilian public hospitals more than doubled from 2008 to 2022, although the average ticket dropped by half (adjusted values: from R$ 12 916 in 2008 to R$ 6443 in 2022). The adjusted amount reimbursed per CABG (mean ticket) decreased by ~23% (from R$ 20 339 in 2008 to R$ 15 723 in 2021).

### Chapter 4 – Cardiomyopathy and Heart Failure

•A study of DATASUS databases showed a reduction in hospitalization due to HF and myocardiopathy in Brazil during the COVID-19 pandemic, associated with an increase in the clinical severity of hospitalized patients as well as in the in-hospital deaths.

•Despite concerns about the impact of the COVID-19 and ChD coinfection, available data do not show an increased risk of death in these patients.

•Recent studies have confirmed a reduction in the prevalence of ChD and its mortality in Brazil in the last decades.

### Chapter 5 – Valvular Heart Disease

•While the prevalence of RHD in Brazil remains relatively stable, with increases explained by better access to diagnosis and incorporation of latent disease into the models, the associated mortality tends to decrease.

•There is growing awareness about the disease in the region, reinforced by local research initiatives and programs.

•Changes in the epidemiological profile and age composition in Brazil are leading to a growing burden of non-rheumatic VHD, especially degenerative aortic and mitral valve disease, that requires specific actions and policies, notably by the public sector.

•There was a notable and multifactorial impact of the COVID-19 pandemic on hospital admissions associated with clinical and surgical management of VHD, and only in late 2021 and 2022 the numbers tended to resume to baseline levels. A similar pattern was observed for the associated costs.

•Considering budgetary constraints and the increasing costs of surgical and percutaneous procedures and devices, investments are required to treat VHD in Brazil, especially to address the specific needs that emerge from administrative and scientific data in the past years.

### Chapter 6 – Atrial Fibrillation and Atrial Flutter

•In the ELSA-Brasil cohort study, which included 14 424 adults with valid ECG (45.8% men; mean age 51 years, age range 35-74 years), the AF and atrial flutter prevalence ascertained by ECG or self-report was 2.5%.

•In the RECALL multicenter prospective registry that included and followed 4585 patients with AF up for 1 year at 89 sites across Brazil from April 2012 to August 2019, death occurred in 8.8/100 patients/year (95% CI, 8.0-9.6) and was associated in multivariable models with older age, permanent AF, NYHA class III/IV, chronic kidney disease, peripheral arterial disease, stroke, chronic obstructive pulmonary disease, and dementia. The use of anticoagulants was associated with lower mortality. Patients with TTR <60% had higher mortality and more major bleeding events compared to patients with TTR ≥60%.

•There was a reduction in hospitalizations and procedures for AF during the COVID-19 pandemic period, particularly in 2020. This reduction was probably related to the indirect effects of the pandemic, during which there was a decrease in cardiovascular hospitalizations in general in Brazil and worldwide.

### Chapter 7 – Hypertension

•The percentage of patients aged 18 years or older with a self-reported diagnosis of hypertension in the Brazilian capitals (Vigitel) was 26.3% in 2021.

•The association of hypertension and hypertension control with social determinants of health, such as urbanization, sex, and race, has been repeatedly shown in Brazil in recent years.

•During the COVID-19 pandemic, there was a change in the hypertension prevalence from a stable trend (from 2007 to 2019) to a slight rise in 2020 and 2021 in the Brazilian capitals, according to the Vigitel data.

### Chapter 8 – Dyslipidemia

•The PNS 2019 used the self-reported diagnosis of high cholesterol levels, and, among the 88 531 adults assessed, a 14.6% prevalence of high cholesterol was identified. The factors most strongly associated with that condition, measured with their PR, were as follows: female sex; age ≥ 60 years; healthcare insurance coverage; poor or very poor health self-assessment; hypertension; diabetes; renal failure; obesity; former tobacco use; alcohol abuse; active leisure time.

•Studies on the role of subclinical atherosclerosis markers in medical practice and on the effect of their use on the clinical treatment modification, as well as on the cost-effectiveness of their use, are required.

•The frequency of dyslipidemia screening, treatment, and control in Brazil, according to sex and age groups, needs to be assessed. In addition, the impact of dyslipidemia on the health system, including costs, has yet to be assessed comprehensively.

### Chapter 9 – Diabetes Mellitus

•There has been a reduction in the number of cases of unknown diabetes in Brazil, which might have resulted from the higher diabetes screening rate and wider access to diagnosis. However, inequalities still characterize access to diagnosis, which is higher among women, self-reported white individuals, the elderly, and those with higher educational level, and lower among self-reported black individuals, those living in rural areas, and those without private health insurance coverage.

•The healthcare indicators of individuals with diabetes have been monitored, showing an increase in the number of individuals receiving medical treatment. However, the screening percentage for chronic complications has worsened in men, younger individuals, black individuals, and those with lower socioeconomic and educational levels.

•Similarly to data from other countries, Brazilian data have shown that severe acute respiratory syndrome due to COVID-19 has increased the likelihood of intensive care unit admission and mortality of individuals with diabetes as compared to those without diabetes.

### Chapter 10 – Smoking and Tobacco Use

•The prevalence of smoking in the adult population points to a total decrease (0.7%) from 2019 to 2021 for both sexes.

•The prevalence of use of other tobacco products in the last 30 days among adolescents increased from 2015 (7.2%; 95% CI 6.1-8.2%) to 2019 (12.4%; 95% CI, 11.8-12.9%).

•The prevalence of the use of e-cigarettes among individuals aged 15 to 65 years increased from 0.45% in 2015 to 0.72% in 2019.

•The total economic burden attributable to tobacco in Brazil in 2020 was US$ 24.3 billion, representing 1.9% of the GDP and 7.8% of the national health expenditures.

### Chapter 11 – Obesity and Overweight

•There was an upward trend in the percentage of overweight and obesity in Brazilian capital cities, from 2006 to 2021, according to VIGITEL data. Most Brazilian capitals showed higher percentages of obesity than the national values, for both sexes, except for Belo Horizonte, Campo Grande, Florianópolis, Palmas, Rio de Janeiro, Salvador, São Luis, Teresina, and Vitória.

•In Brazilian capital cities, the percentage of all adults (age ≥18 years) with obesity in 2021 was 22.4% (22.0% for men and 22.6% for women). A progressive increase of obesity was observed with age increase, ranging from 12.2% in the age group of 18-24 years to 26.2% in the age group of 45 - 54 years. For the age group of 60+ years, there was a slight reduction in the obesity prevalence, 21.8%.

•An increase in obesity prevalence has also been observed among Brazilian children and adolescents.

### Chapter 12 – Physical Activity

•Despite the increasing knowledge about the cardiovascular benefits of regular physical activity and the trend towards a reduction in physical inactivity among Brazilians in recent years, almost half of the Brazilian population does not meet the recommended minimum level of physical activity, the worse figures being found among women, elderly individuals, and those with lower educational levels.

•That scenario has been compounded by the COVID-19 pandemic, with an increase in the time dedicated to sedentary behaviors in the entire country, mainly among women and young adults aged 18-29 years.

•Data from the GBD 2019 have shown that despite the 47.6% reduction in the mortality rate from CVD attributable to low levels of physical activity between 1990 and 2019, 7.6% of all deaths due to CVD in Brazil are still attributed to that risk factor.

## CHAPTER 1 – TOTAL CARDIOVASCULAR DISEASES

### ICD-9 390 to 459; ICD-10 I00 to I99.

**See Tables 1-1 through 1-7 and Charts 1-1 through 1-7**

**Table:** Abbreviations used in this chapter

CABG	Coronary Artery Bypass Grafting
CHD	Coronary Heart Disease
CI	Confidence Interval
COVID-19	Coronavirus Disease 2019
CVD	Cardiovascular Diseases
DALYs	Disability-Adjusted Life Years
DATASUS	Brazilian Unified Health System Database
ELSA-Brasil	The Brazilian Longitudinal Study of Adult Health (in Portuguese *, Estudo Longitudinal de Saúde do Adulto* )
FU	Federative Unit
GBD	Global Burden of Disease
GDP	Gross Domestic Product
HDI	Human Development Index
HDIm	Municipal Human Development Index
HF	Heart Failure
IBGE	Brazilian Institute of Geography and Statistics
ICD	International Statistical Classification of Diseases and Related Health Problems
NCD	Non-Communicable Disease
NCDP	Non-Communicable Disease of Poverty
OR	Odds Ratio
SIDRA	IBGE Automated Retrieval System (in Portuguese, *Sistema IBGE de Recuperação Automática)*
SIM	Brazilian Mortality Information System (in Portuguese, *Sistema de Informações sobre Mortalidade* )
SUS	Brazilian Unified Health System (in Portuguese, *Sistema Único de Saúde* )
UI	Uncertainty Interval
WHO	World Health Organization

### Overview

•Noncommunicable diseases comprise the world’s leading group of causes of death, responsible for premature deaths, loss of quality of life, and adverse economic and social impacts. The NCDs are responsible for approximately 70% of global deaths, equivalent to more than 38 million deaths annually, significantly exceeding deaths from external causes and infectious diseases.^40 - 43^Of all deaths due to NCD worldwide, nearly 33%, over 18 million, result from CVD. A similar distribution is observed in Brazil, where 72% of deaths are due to NCD, of which 30% are due to CVD and 16% to neoplasms ( **Chart 1-1** ).^31 , 44 , 45^

**Chart 1-1:** – Proportional mortality due to infectious diseases (ICD-10 Chap, I) and cardiovascular diseases (Chap, IX) in relation to total deaths, Brazil, 2021. CVD: cardiovascular diseases, Ministry of Health of Brazil – Hospital Information System of the Unified Health System (SIH/SUS). ^49^

•The definition of CVD may vary according to the study, from including all diseases listed in ICD-10 Chapter IX to just grouping together the three leading conditions (CHD, stroke, and other heart diseases). For the GBD, the definition of total CVD comprises ten conditions: rheumatic heart disease, CHD, cerebrovascular disease, hypertensive heart disease, cardiomyopathy, myocarditis, atrial fibrillation and flutter, aortic aneurysm, peripheral vascular disease, and endocarditis.^46^

•Cardiovascular disease is the leading cause of premature death worldwide, responsible for approximately one-third of all deaths. This number is expected to increase in the future.^47^

•Cardiovascular diseases used to be the number 1 cause of death in Brazil. According to the GBD Study 2019 estimates, CHD was the leading cause of death in the country, followed by stroke in both 1990 and 2019. In fact, in 2019, CHD was the leading cause of death in all Brazilian FUs, except for the state of Amazonas, in the Northern region. Three states in that region, Acre, Amapá, and Pará, showed no significant difference regarding mortality rates due to CHD and stroke.^4^

•In 2021, due to the COVID-19 pandemic, CVDs were no longer the leading cause of death, giving way to infectious and contagious diseases in most Brazilian FUs, according to data from the SIM ( **Chart 1-1** ).^11^

•In 2021, COVID-19 became the leading cause of death among men and women. Among men, the second leading cause of death was CHD, followed by cerebrovascular diseases. Among women, this order reversed, with cerebrovascular diseases being the second leading cause and CHD, the third ( **Table 1-1** ).^12^

**Table 1-1:** – COVID-19 was the first of 10 leading causes of death in Brazil in 2021

Male Rank	Cause	%	Female Rank	Cause	%
1	COVID-19	24.3	1	COVID-19	24.0
2	Ischemic heart disease	6.9	2	Cerebrovascular disease	6.4
3	Cerebrovascular disease	5.3	3	Ischemic heart disease	6.0
4	Homicide	3.9	4	Diabetes mellitus	5.2
5	Lower respiratory infections	3.8	5	Lower respiratory infections	4.4
6	Diabetes mellitus	3.6	6	Alzheimer and other dementias	2.7
7	Road traffic accidents	2.6	7	Breast cancer	2.3
8	Chronic obstructive pulmonary disease	2	8	Chronic obstructive pulmonary disease	2.0
9	Cirrhosis of the liver	1.7	9	Hypertensive disease	1.8
10	Prostate cancer	1.6	10	Tracheal, bronchial and lung cancers	1.6

*Source: Ministry of Health of Brazil Hospital Information System of the Unified Health System (SIH/SUS).^*49*^*

### Prevalence

•The prevalence of CVD increases as people age, regardless of gender. However, the difference in prevalence between males and females is more significant among children under 5 years of age. From 5 years to 44 years, women have a higher prevalence, but, after 44 years, the prevalence among men increases and the difference between genders is highest in the age group of 60-69 years, as shown in **Chart 1-2** .^48^

**Chart 1-2:** – Prevalence of cardiovascular diseases among males and females by age group and prevalence ratio, Brazil, 2021. Data from the Global Burden of Disease Collaborative Network, Global Burden of Disease (GBD) Cardiovascular Burden Estimates 1990 and 2021, Institute for Health Metrics and Evaluation, University of Washington. ^48^

•According to a recent update of the GBD data, the prevalence rate of CVD in Brazil in 2021 was 6.9% (6.4-7.4) for both sexes, with a higher rate among males 7.6% (7.0-8.1) than females 6.3% (6.0-6.9).^48 , 49^

•Gonçalves *et al* . published in 2019 a cross-sectional study from the Brazilian National Health Survey conducted in 2013 on a sample of 60 202 adults over 18 years of age, stratified by sex and age groups, using a hierarchical binary logistic regression model. The self-reported diagnosis of heart disease in Brazil was 4.2% (95% CI, 4.0 - 4.3), being associated with the female sex (OR = 1.1; 95% CI, 1.1 - 1.1), individuals 65 years and older (OR = 4.7; 95% CI, 3.3 ‒ 5), hypertension (OR = 2.4; 95% CI, 2.2 ‒ 2.7), elevated cholesterol (OR = 1.6; 95% CI, 1.5 ‒ 1.8), overweight (OR = 1.5; 95% CI, 1.4 ‒ 1.8) or obesity (OR = 2.0; 95% CI, 1.7 ‒ 2.2), sedentary behavior (OR=1.5; 95% CI, 1.02 ‒ 2.1), and tobacco use (OR = 1.2; 95% CI, 1.03 ‒ 1.3).^50^

•In the ELSA-Brasil, a cohort study that included 15 105 civil servants from six academic institutions (54% women, 35-74 years, with baseline assessment between 2008 and 2010), the self-reported prevalence of CVD was as follows: CHD, 4.7% (men=5.7%, women=4.0%); HF, 1.5% (men=1.9%, women=1.5%); stroke, 1.3% for both sexes; rheumatic fever, 2.9% (men=2.2%, women=3.4%); and Chagas disease, 0.4%, for both sexes.^51^

•According to the 2013 Brazilian National Health Survey, the percentage of people in Brazil with ideal cardiovascular health was less than 1%. This percentage was even lower among men, individuals over 60 years of age, and those living in the Brazilian Southern region. International studies have found similar outcomes, indicating that the prevalence of ideal cardiovascular health in the Southern Cone countries (Argentina, Chile, and Uruguay) and the United States is less than 1% and null, respectively.^52^

•According to the Brazilian National Health Survey, in 2019, there were 12 946 932 individuals with CVD in Brazil, and 51% of them were men. The prevalence rate of CVD decreased among the elderly but increased among males and females aged 15-49 years.^53^

### Incidence

•According to the GBD Study 2019, the age-standardized incidence rate of CVD in Brazil in 2019 was 475 (95% UI, 447-507) per 100 000 inhabitants. From 1990 to 2019, that rate decreased -20% (-22 to -18).^4^The latest GBD estimates for CVD are from 2019 and have been discussed in the previous version of the Cardiovascular Statistics – Brazil (2021).^4^Updated GBD estimates had not been made available until the completion of this document.

•Globally, there is an increasing incidence of non-communicable diseases of poverty (NCDP), including CVDs (mainly heart disease and stroke), diabetes, osteoarthritis, and several types of cancers (breast, prostate, liver, kidney, and colon). Despite the well-established epidemiological transition from infectious diseases to NCD, high levels of various infectious diseases continue to occur concomitantly with NCD, the former often associated with poorly maintained or limited infrastructure, inadequate housing, crowding, and unsanitary conditions. While this is particularly so in poor countries, it is also true among poor communities in high-income settings.^53 , 54^

### Mortality

•Data from the GBD Study 2021 reveal that, although mortality rates from CVD in Brazil significantly decreased over the past few years, the total number of deaths due to CVD increased because of population growth and aging ( **Chart 1-4** ). The age-standardized mortality rate per 100 000 inhabitants was 348.5 (325;359.5) in 1990 and 162.2 (145.8;171.5) in 2021, decreasing by -53.5%. The age-standardized mortality rates were higher for men throughout the whole period, and the percent decrease was higher for women (-56%) than for men (-50.5%).^48^

**Chart 1-4:** – Trends of cardiovascular diseases crude death rates in Brazil, male and female, 2017-2021. Data from the Ministry of Health of Brazil Hospital Information System of the Unified Health System (SIH/SUS). ^49^

•A variation has been observed among the Brazilian states, with the five highest rates observed in Maranhão, Roraima, Amapá, Alagoas, Tocantins, Pernambuco, and Rio de Janeiro. The lowest five rates were observed in Amazonas, Rio Grande do Sul, Bahia, Rio Grande do Norte, Minas Gerais, Mato Grosso, and Santa Catarina ( **Chart 1-3 and Table 1-2** ).^48^

**Chart 1-3:** – Standardized death rate due to cardiovascular diseases, both sex, Brazil and Federative Units, 2021. Data from the Global Burden of Disease Collaborative Network, Global Burden of Disease (GBD) Cardiovascular Burden Estimates 1990 and 2021, Institute for Health Metrics and Evaluation, University of Washington. ^48^

**Table 1-2:** – Age standardized mortality rates due to cardiovascular diseases, Brazil and Federative Units, 2021

Federative Units		Mortality
Acre		168.8(153.6-181.3)
Alagoas		203.8(178.4-228.3)
Amapá		176(154.9-198.3)
Amazonas		145.2(130.7-157.6)
Bahia		148(130.6-164.3)
Ceará		164(141.1-185.4)
Distrito Federal		172(153.8-188.1)
Espírito Santo		165.9(145.4-180.8)
Goiás		159.2(142.4-174.1)
Maranhão		218.1(196.1-241.4)
Mato Grosso		151.7(133.6-165.8)
Mato Grosso do Sul		170.9(153.5-188.6)
Minas Gerais		135.6(120.4-147.7)
Pará		168.2(143.5-196.1)
Paraíba		169.5(154.2-185.3)
Paraná		158.7(144.9-172.8)
Pernambuco		181.2(158.2-197.7)
Piaui		174(149.3-194.7)
Rio de Janeiro		182.1(160.3-200.7)
Rio Grande do Norte		148.5(130.7-164)
Rio Grande do Sul		147.3(133.5-159.8)
Rondônia		156.2(135.4-176)
Roraima		211.6(182.7-243)
Santa Catarina		150.9(132.4-164.3)
São Paulo		168.9(148.5-186.4)
Sergipe		151.9(135-165.7)
Tocantins		190.2(168.6-214.6)
Brazil		162.2(145.8-171.5)

*Source: Data derived from Global Burden of Disease Collaborative Network. Global Burden of Disease (GBD) Cardiovascular Burden Estimates 1990 and 2021, Institute for Health Metrics and Evaluation, University of Washington.^*48*^*

•In 2021, Brazil experienced a significant shift in its mortality profile due to the pandemic. For the first time, infectious diseases became the primary cause of death, surpassing CVDs by over 100 000 deaths. Infectious diseases accounted for 27% of all deaths, with 486 667 deaths, while CVD caused 382 507 deaths, representing 21% of all deaths. Among infectious diseases, COVID-19 was the leading cause of death, with 424 461 deaths in 2021.^48^

•The mortality rate from CVD is higher in males than females in nearly all age groups. As shown in **Chart 1-5** , the ‘male to female’ ratio peaks in the age group ‘50-69 years’ and declines up to the age group ‘90-94 years. However, after the age of 95 years, females have a higher mortality rate than males.^48^

**Chart 1-5:** – Male to female cardiovascular disease mortality rate ratio by age group, Brazil, 2021. Data from the Global Burden of Disease Collaborative Network, Global Burden of Disease (GBD) Cardiovascular Burden Estimates 1990 and 2021, Institute for Health Metrics and Evaluation, University of Washington. ^48^

•Before the COVID-19 pandemic, CVD was the leading cause of death globally and, in 2019, 58% of the deaths in Asia were due to CVD. The WHO has established a global action plan for the year 2025, aiming to reduce by 25% the number of premature deaths from NCDs, including CVD.^55^

•In Brazil, Mansur *et al* . have shown that the age-standardized CVD mortality rate has declined significantly in recent decades. A 2016 study analyzed CVD mortality rates from the age of 30 years and older, by sex, per 100 000 inhabitants. The annual variations in cardiovascular mortality for the periods 1980-2006 and 2007-2012 were, respectively: for both sexes: -1.5% and -0.8%; men: -1.4% and -0.6%; women: -1.7% and -1.0%.^56^

•Baptista *et al* . have investigated how age composition and age-specific mortality rates are related to the observed difference in deaths from CVD in the adult population by sex, in Brazilian microregions from 1996 to 2015. After correcting for underreporting of death counts, they suggested that there was a decline in the rates of deaths from CVD in the period studied. However, the main driver of the change in mortality rates was heterogeneous across Brazilian microregions. In general, in the most socioeconomically developed areas, the age structure was more importantly related to the mortality rates, with older populations dying from CVD. Interestingly, there were differences in the main drivers of CVD mortality even within the Brazilian regions and FUs.^57^

•Malta *et al.* have compared the historical series of CVD mortality rates in Brazil using the SIM database with and without correction and the GBD 2017 estimates between 2000 and 2017. The authors pointed out that the increase in mortality rates observed in 2017 in some Northern and Northeastern FUs was due to the improvement in death registration and better classification of underlying causes of death in recent years.^25^

•Brant *et al.* , analyzing GBD 2015 data, have observed a decrease in age-standardized CVD mortality rate from 429.5 (1990) to 256.0 (2015) per 100 000 inhabitants (-40.4%), with marked differences across the FUs. That decrease was more pronounced in the FUs of the Southeastern and Southern regions and the Distrito Federal, regions that concentrate the largest populations and income, and more modest in most Northern and Northeastern states.^46^

•The Family Health Program coverage was associated with a reduction in hospitalizations and mortality from CVD that were included in Brazil’s national ambulatory care-sensitive list, and its effect increased according to the duration of the Family Health Program implementation in the municipality. Rasella *et al* . have observed reductions in the mortalities from cerebrovascular disease and heart disease of 0.82 (95% CI, 0.79 - 0.86) and 0.79 (95% CI, 0.75 - 0.80), respectively, reaching 0.69 (0.66 - 0.73) and 0.64 (0.59 - 0.68), respectively, when the Family Health Program coverage was consolidated during all 8 years studied.^58^

•Research has shown that there is a strong association between low socioeconomic *status* and a higher burden of CVD. As the socioeconomic *status* increases, CVD rates tend to decrease. This suggests that improvements in the local socioeconomic conditions have led to a reduction in CVD mortality, a finding consistent with that of other studies.^59^

•Lotufo *et al* . have compared three different household income levels (high, middle, and low) with mortality rates due to CVD in the city of São Paulo from 1996 to 2010. The annual percent change and 95% CI for men living in the high-, middle-, and low-income areas were -4.1 (-4.5 to -3.8), -3.0 (-3.5 to -2.6), and -2.5 (-2.8 to -2.1), respectively. The trend rates of women living in the high-income areas were -4.4 (-4.8 to -3.9) in 1996-2005 and -2.6 (-3.8 to -1.4) in 2005-2010. The reduction in deaths due to CVD was more significant for men and women living in the wealthiest neighborhoods, with a greater declining risk of death gradient for those living in the wealthiest areas compared to the most deprived neighborhoods.^60^

•An inverse association of the HDIm and the supplementary health coverage with mortality due to CVD was observed, suggesting a relationship between socioeconomic factors and CVD. The HDIm increased between 2000 and 2010 in all FUs, in half of which it was 0.7 or higher. Supplementary health coverage increased in the country during the study period and was inversely associated with mortality due to CVD between 2004 and 2013.^61^

•Soares *et al* . have observed a decrease in CVD mortality in the states of Rio de Janeiro, São Paulo, and Rio Grande do Sul that preceded improvement in the socioeconomic index. The GDP per capita evolution, the child mortality decline, a higher educational level (represented by the schooling years of individuals over 25 years), and the HDIm showed a high correlation with the reduction in the CVD mortality rate. A reduction in the mortality rates due to DCV, stroke, and CHD in the state of Rio de Janeiro in past decades was preceded by an increase in the HDI. An increment of 0.1 in the HDI correlated with the following reductions in the number of deaths per 100 000 inhabitants: 53.5 for DCV; 30.2 for stroke; and 10.0 for CHD.^62 , 63^

•Baptista *et al* . have investigated the relationship between CVD mortality rate and economic development over time and space, measured by GDP per capita, in Brazilian microregions from 2001 to 2015. Using nationwide databases (SIM, DATASUS and SIDRA, IBGE) the authors observed a rapid decline in CVD mortality in the Southern and Southeastern microregions and a slower decline in the West-Central region. On the other hand, the Northern and Northeastern regions had an increased CVD mortality over time, maybe due to lower access to healthcare and socioeconomic factors.^64^

•Silveira *et al* ., studying the effect of ambient temperature on cardiovascular mortality in 27 Brazilian cities, have observed a higher number of cardiovascular deaths associated with low and high temperatures in most Brazilian cities and the West-Central, Northern, Southern, and Southeastern regions. The overall relative risk for Brazil was 1.26 (95% CI, 1.17–1.35) for the 1^st^percentile of temperature and 1.07 (95% CI, 1.01–1.13) for the 99^th^percentile of temperature *versus* the 79^th^percentile (27.7 °C), whose relative risk was the lowest.^65^

### Burden of Disease

•CVDs not only impact population mortality or reduce life expectancy but also generate disability, thereby extending the period of life during which an individual, once affected, becomes unproductive or dependent on others for performing daily activities. Additionally, individuals may lose their economically productive capacity. **Table 1-3** presents the DALYs for 2021, categorized by age group and further adjusted by age for men and women in Brazil.^48^

**Table 1-3:** – Rates of DALYs due to cardiovascular diseases by age, sex, and year, in Brazil, 2021

Age group (years)	Female	Male	Both
Under 5	327.8(260.1;394.8)	383.9(308;472.4)	356.5(286.1;432.5)
5 to 19	198.1(167.6;235.1)	231.2(200.4;269.7)	214.9(185.7;251.6)
20 to 24	439.6(384.6;494.9)	608.4(552.8;683.7)	524.6(472.5;586.1)
25 to 29	546.4(480.6;613.8)	793.1(739.1;863.7)	668.4(613.3;733.6)
30 to 34	775.9(698.5;861.4)	1190.5(1111.4;1272.3)	978.9(904.8;1055.6)
35 to 39	1198.1(1096.1;1296.4)	1842.9(1739.1;1970.9)	1513.7(1439.4;1602.8)
40 to 44	1955.9(1814.5;2100.1)	3061.7(2905.8;3293.4)	2493(2381.7;2634.4)
45 to 49	2957.4(2776.7;3140.4)	4816(4573.4;5128)	3857.8(3675.1;4030.6)
50 to 54	4034.7(3829.6;4251.5)	7509.3(7126.1;7954.9)	5699.7(5478.4;5943.6)
55 to 59	5396.1(5096.8;5655.6)	10411.1(9888.5;11071)	7761.3(7446.2;8148.5)
60 to 64	7582.7(7157.8;8013.5)	14377.2(13743.6;15250.1)	10724.5(10227.3;11241.4)
65 to 69	10440.7(9551.5;10999.4)	18468.3(17560.5;19567.3)	14100.1(13252.9;14825.6)
70 to 74	13847.1(12713.4;14787.4)	22435.5(21179.5;23838.4)	17700.4(16549.4;18565)
75 to 79	18640.7(16866.6;19781.3)	27676.6(25780;29487)	22551.3(20604.1;23670.6)
80 to 84	24129(20672.6;26360.9)	32268.2(28825.4;34642.2)	27434.4(24030;29537.3)
85 to 89	30302.1(24656.6;33410)	37590.2(32959.5;40444.5)	33002.3(27682.6;35861)
90 to 94	36517.8(28368.6;41236)	39737.4(33252.6;43645.7)	37646(30075.8;42020.3)
95 plus	40754.9(30729.3;46726.8)	36987.3(29033.6;41740.1)	39576.6(30225.9;45134.3)
All Ages	2810.8(2594.7;2989.8)	4462.4(4215.9;4722.8)	3568.9(3343.4;3729.7)
Age-standardized	3389.8(3126.3;3610.1)	4713.5(4462.7;4992.9)	4035.5(3789.2;4219.2)


*Source: Data derived from Global Burden of Disease Collaborative Network. Global Burden of Disease (GBD) Cardiovascular Burden Estimates 1990 and 2021, Institute for Health Metrics and Evaluation, University of Washington.^*48*^*

•The distribution of NCD mortality is highly unequal, as 86% of premature deaths caused by NCDs before age 70 years are found in low- and middle-income countries. While there has been a global decrease in the incidence rates, CVDs remain the leading cause of NCD mortality. Additionally, diabetes-related deaths have increased worldwide in all regions over the past 30 years. Annual changes in the rates of DALYs and mortality per 100 000 vary among regions with diverse development profiles. This highlights the difference in the impact of various factors on health outcomes.^54^

### Health Care Utilization and Cost

•In Brazil, from 2008 to 2021, hospital admissions due to cardiovascular diseases increased until 2019 and dropped in 2020 and 2021, corresponding to 89,000 fewer hospital admissions 2020 than in 2019, a 12% reduction. Hospitalizations for clinical conditions showed a trend to decrease in the last decades, while those related to procedures or surgical interventions increased during this period ( **Tables 1-4 and 1-5** ).^49^Ischemic heart diseases, cerebrovascular diseases, cardiac failure, and arrhythmia accounted for 68% of all cardiovascular disease hospital admissions ( **Table 1-4** ).^49^

**Table 1-4:** – Total number of hospitalizations for clinical procedures for cardiovascular diseases in the SUS by competence year, Brazil, 2008 to 2021

	2008	2009	2010	2011	2012	2013	2014	2015	2016	2017	2018	2019	2020	2021	Total
Ischemic Heart disease	12,393	9,743	9,300	8,497	8,000	7,197	7,581	6,403	6,317	6,171	6,292	6,703	5,099	4,559	104,255
Cerebrovascular disease	159,545	176,047	181,035	184,751	182,065	183,043	187,110	191,678	195,787	198,068	203,066	211,149	199,126	206,518	2,658,988
Valvular disease	3,237	4,156	3,526	3,637	3,285	2,996	2,753	2,400	2,244	2,231	2,330	2,289	1,536	1,597	38,217
Atrial Fibrillation	29,034	28,174	28,382	28,583	28,760	28,268	29,799	29,754	29,889	30,265	30,958	32,753	26,764	25,283	406,666
Acute MI	47,358	50,987	55,513	58,194	59,562	58,552	62,809	66,647	70,441	71,835	74,569	80,614	76,444	81,143	914,668
Heart Failure	298,474	297,763	289,110	284,844	264,469	254,285	243,913	240,832	236,358	230,297	222,394	222,620	187,770	181,441	3,454,570
Cardiomyopathies	2,092	2,363	2,459	2,302	2,357	2,293	2,370	2,230	2,250	1,997	2,251	2,390	1,899	1,868	31,121
Acute coronary Syndrome	63,300	68,833	72,912	71,523	75,734	73,432	76,945	72,686	70,430	70,713	68,413	70,204	56,583	52,827	964,535
Total	615,433	638,066	642,237	642,331	624,232	610,066	613,280	612,630	613,716	611,577	610,273	628,722	555,221	555,236	8,573,020

*Source: Ministry of Health of Brazil – Hospital Information System of the Unified Health System (SIH/SUS).^*49*^*

•The number of hospital admissions for CVDs after the year 2020 showed a significant reduction in all Brazilian regions, as depicted in **Chart 1-6** . The Southeastern Region had the highest number of CVD hospital admissions, with a drop of 77 000 admissions in 2020, a reduction of 15%. All regions showed reduced CVD hospital admissions in 2020 and 2021, **Chart 1-6 .** ^49^

**Chart 1-6:** – Hospital admissions for cardiovascular disease according to the Brazilian regions, 2010-2022. Data from Ministry of Health of Brazil – Hospital Information System of the Unified Health System (SIH/SUS). ^49^

•The early years of the COVID-19 pandemic, 2020 and 2021, showed a significant reduction in hospital admissions for all types of diseases of the circulatory system ( **Chart 1-6** ).^49^Some factors should be considered. Hypotheses, such as increased number of cardiovascular deaths outside the hospital, reduced search for medical care, and hospital admission with concurrent CVD and COVID-19 being recorded as the primary diagnosis, could explain these data.

•The number of hospital admissions for acute myocardial infarction increased by 50% from 2010 to 2021. There were 61 000 hospital admissions in 2010 and 93 000 in 2021. Acute myocardial infarction slightly decreased in 2020 and increased in 2021 ( **Table 1-4** ).^49^Primary angioplasties increase significantly in the last 10 years in the SUS in both absolute numbers and adjusted for population ( **Table 1-5** ).^49^

**Table 1-5:** – Total number of hospitalizations for surgical procedures for cardiovascular diseases in the SUS by competence year, Brazil, 2008 to 2021

	2008	2009	2010	2011	2012	2013	2014	2015	2016	2017	2018	2019	2020	2021	Total
Atrial Fibrillation Ablation	68	72	90	85	123	139	143	161	124	120	125	163	120	161	1,694
Coronary angioplasty	38,635	45,648	49,492	55,931	60,959	63,838	66,492	66,550	69,802	73,971	78,575	85,518	77,846	80,190	913,447
CABG	20,515	22,077	21,225	23,187	23,900	23,249	22,997	22,559	22,248	21,474	20,674	21,018	16,554	15,932	297,609
Valvar surgery	12,201	12,664	12,169	13,181	13,435	13,067	12,993	12,624	12,432	12,277	12,088	12,771	9,198	8,759	169,859
Primary angioplasty	7,648	6,362	6,262	6,033	5,865	6,055	7,135	8,524	10,195	10,774	10,811	11,099	11,253	11,795	119,811
Cardiomyopathy	15	43	13	21	28	23	20	18	32	29	26	24	18	14	324
Other valvar diseases	451	477	445	486	456	527	515	513	399	427	391	450	399	470	6,406
Mitral valvuloplasty	477	551	478	473	403	431	408	341	206	236	200	195	129	159	4,687
Total	80,010	87,894	90,174	99,397	105,169	107,329	110,703	111,290	115,438	119,308	122,890	131,238	115,517	117,480	1,513,837

Source: Ministry of Health of Brazil – Hospital Information System of the Unified Health System (SIH/SUS). ^49^

•Over the last 14 years, in Brazil, there has been a significant reduction in hospitalizations for HF, an increase in annual hospitalizations for acute myocardial infarction and cerebrovascular diseases, and flat trends in other groups of clinical procedures ( **Table 1-4** ).^49^

•Regarding the surgical approaches in the same years, there was a great increase in the annual number of coronary angioplasties and a trend towards decreases of surgical revascularization, and stability in the number of other surgical procedures ( **Chart 1-7** ).^49^

**Chart 1-7:** – Total amount paid and number of surgical procedures per year in the SUS, Brazil, from 2008 to 2021. CABG: coronary artery bypass grafting; IPCA: Brazilian consumer price index, Data from Ministry of Health of Brazil – Hospital Information System of the Unified Health System (SIH/SUS). *49*

•From 2008 to 2010, the SUS paid for clinical admissions an average of R$1,4 billion per year; from 2017 to 2019, an average of R$1,1 billion, and from 2020 to 2021, it was 993 million per year ( **Table 1-6** ). Surgical procedures were paid $1,4 billion, $1,3 billion, and $1,1 billion, respectively. Although the number of admissions due to cardiovascular conditions more than doubled during this last decade, there was a significant reduction in the reimbursement for cardiovascular diseases ( **Chart 1-7 and Table 1-7 )** .^49^

**Table 1-6:** – Adjusted amount paid (in Reais) of clinical hospitalizations for cardiovascular diseases by competence year, Brazil, 2008 to 2021

	2008	2009	2010	2011	2012	2013	2014	2015	2016	2017	2018	2019	2020	2021
Ischemic Heart disease	17,473,695	14,515,852	13,375,022	11,971,544	10,232,200	8,917,122	9,968,291	7,750,106	7,256,573	7,090,575	7,061,618	7,770,301	6,019,912	5,239,722
Cerebrovascular disease	318,306,994	398,722,244	403,265,605	393,470,650	393,157,770	387,626,894	389,295,216	380,584,520	359,325,511	348,788,550	356,413,503	364,584,254	348,288,096	350,863,859
Valvular disease	2,357,047	3,362,521	2,919,687	3,077,011	2,714,235	2,565,230	2,541,501	2,521,358	2,282,181	2,151,729	2,543,861	2,399,303	1,755,725	1,613,260
Atrial Fibrillation	30,900,437	36,806,646	37,599,982	36,117,396	36,633,135	33,928,480	36,315,140	35,172,431	32,597,899	33,400,513	33,577,340	34,490,032	29,215,902	27,237,974
Acute MI	145,683,996	178,378,823	188,575,764	186,393,207	188,636,874	180,519,838	191,841,885	194,066,706	183,785,925	174,865,678	178,459,141	181,336,606	168,242,806	167,576,265
Heart Failure	610,079,105	683,083,024	665,130,816	632,953,807	571,113,086	546,610,902	523,214,027	508,987,795	470,752,097	444,528,797	434,269,861	431,135,829	369,639,100	356,801,947
Cardiomyopathies	2,885,134	4,023,341	4,347,884	3,638,104	3,795,299	3,910,733	4,325,566	4,043,157	4,175,497	3,276,496	3,883,812	3,808,453	3,102,123	3,172,783
Acute coronary Syndrome	100,179,911	122,550,378	131,057,092	125,609,896	135,250,270	126,783,127	134,127,142	123,767,713	109,233,624	105,187,837	99,639,789	97,394,487	76,776,561	69,697,477
Total	1,227,866,320	1,441,442,829	1,446,271,852	1,393,231,616	1,341,532,868	1,290,862,326	1,291,628,769	1,256,893,786	1,169,409,306	1,119,290,174	1,115,848,924	1,122,919,263	1,003,040,225	982,203,288

Source: Ministry of Health of Brazil – Hospital Information System of the Unified Health System (SIH/SUS). ^49^

**Table 1-7:** – Adjusted amount paid (in Reais) of clinical procedures for cardiovascular diseases by competence year, Brazil, 2008 to 2021

	2008	2009	2010	2011	2012	2013	2014	2015	2016	2017	2018	2019	2020	2021
Atrial Fibrillation Ablation	807,686	797,310	955,644	875,098	1,240,832	1,339,634	1,236,360	1,366,392	963,242	885,222	911,289	1,154,269	863,980	1,045,998
Coronary angioplasty	471,716,260	564,185,839	599,670,065	647,278,961	669,078,423	660,801,300	659,753,883	621,249,372	590,665,315	598,141,041	617,339,631	655,318,815	586,608,702	579,077,077
CABG	394,422,557	441,323,860	435,052,405	551,289,001	535,610,518	493,649,155	473,022,182	436,663,808	389,825,203	361,649,521	342,491,428	334,232,757	258,310,185	235,521,656
Valvar surgery	282,216,914	297,658,304	288,805,944	343,030,523	329,575,746	303,391,954	288,907,966	266,567,822	238,830,404	225,744,421	221,078,457	224,844,776	158,038,290	144,300,688
Primary angioplasty	101,427,047	80,162,895	75,278,257	68,136,360	63,921,172	63,354,779	73,608,840	84,578,572	90,611,405	91,797,091	91,413,951	89,883,845	88,171,102	89,814,989
Cardiomyopathy	378,650	1,152,515	390,162	624,198	784,238	601,155	490,184	449,706	717,795	579,036	530,431	485,825	359,755	249,798
Other valvar diseases	3,403,160	3,515,486	3,483,819	3,674,623	3,363,929	3,485,710	3,414,332	3,144,844	2,171,741	2,420,709	2,103,416	2,351,343	2,088,988	2,393,891
Mitral valvuloplasty	6,980,121	7,585,874	6,383,913	6,181,863	4,888,469	5,046,819	4,505,648	3,607,236	1,876,617	2,205,110	1,819,664	1,716,096	1,062,664	1,137,898
Total	1,261,352,395	1,396,384,093	1,410,020,209	1,621,090,628	1,608,463,326	1,531,670,505	1,504,939,395	1,417,627,751	1,315,661,722	1,283,422,152	1,277,688,268	1,309,987,725	1,095,503,666	1,053,541,996

Source: Ministry of Health of Brazil – Hospital Information System of the Unified Health System (SIH/SUS). ^49^

•Although the number of hospitalizations and interventional procedures increase in the last decade, payments for cardiovascular surgical procedures by the SUS in the last years of the series have decreased, especially in more complex procedures, such as CABG and valve replacement surgeries, as shown in **Chart 1-7** .^49^

•Brant *et al* ., analyzing CVD mortality during the pandemic years in Belo Horizonte city, have found the greater occurrence of CVD deaths at home, in parallel with lower hospitalization rates. Those authors concluded that “CVD care was disrupted during the COVID-19 pandemic, which more adversely affected older and more socially vulnerable individuals, exacerbating health inequities in Belo Horizonte city”.^66^

•Public institutions assist more than 70% of Brazilians, but the supplementary health systems account for almost the same share of healthcare expenditures. Unfortunately, there are scarce published studies or open databases to calculate the additional economic burden of cardiovascular from this segment. Overall estimates based on cardiovascular disease prevalence suggest that the direct and indirect cost of CVD is greater than 4 to 5 times the direct cost for SUS, approximately 10 billion dollars.^67^

### Future Directions

•The SIM, introduced in 1975, is a crucial tool for tracking mortality statistics in Brazil, because the registration of all deaths is mandatory in the FUs, with 98% coverage of the entire country in 2017. However, coverage is lower in the Northern region compared to the Southern region. The WHO usability index of the SIM is high, making the SIM information useful for analysis, although some data corrections may be required in the time series.^68^

•The Northeastern region has the poorest coverage of the SIM, still under 95%.^54^Although SIM has improved through specific Ministry of Health projects, some problems persist, such as ill-defined causes (around 6%), severe garbage codes around 15%, and misclassification of causes of death, which generate biases that may disrupt the metrics presented.^49^

•Garbage code issues are due to the poor quality of some death certificates and issues associated with limited access to health services, impaired access to diagnosis, or simply the quality of medical records, making it difficult to retrieve information.^49^In this regard, the best way to ensure the accuracy of the underlying cause of death is through the training of physicians throughout the country regarding death certification.

•It is worth mentioning that there is a lack of primary incidence data (cohorts) in Brazil, which requires research that allows us to understand how to face CVD in states and populations with low socioeconomic indices.

•Because of the reduction in the declining trend of age-standardized CVD mortality in the last 5 years, novel strategies to tackle CVD mortality must be studied. Understanding the drivers of this change is essential to implement effective policies, particularly facing population aging, which will increase the number of individuals with CVD in the country.

•The impact of COVID-19 and long-term COVID-19 on CVD deaths and burden has been incompletely studied, and there are many aspects to be uncovered. The disruption in the health system and the health statistics has been documented, but uncertainties on future trends are significant. Regarding CV health, it is essential to understand the long-term effect of the COVID-19 infection on those with CV risk factors and diseases.

## CHAPTER 2 – STROKE (CEREBROVASCULAR DISEASES)

### ICD-9 430 to 438; ICD-10 I60 to I69.

**See Table 2-1 and Charts 2-1 and 2-2**

**Table 2-1:** – Age-standardized mortality and DALY rates due to stroke per 100 000, in Brazil and Federative Units in 2021

	DALY	Mortality
Acre	560.4(513.6-606)	36.5(32.4-39.7)
Alagoas	753.8(667.3-828.1)	47(40.3-51.6)
Amapá	650.8(584-731.2)	42.1(35.8-47.8)
Amazonas	542.9(488.9-595.4)	35(31-38.2)
Bahia	523(454.7-580.2)	32.1(27.6-36)
Brazil	511.4(470.6-542.5)	31.7(28-33.9)
Ceará	513.5(445.6-580.7)	33.6(28.4-38.2)
Distrito Federal	573.3(521.3-630.5)	44.5(39.9-48.7)
Espírito Santo	553.3(494.4-602.7)	34.2(29.3-37.4)
Goiás	481.4(442.4-532.4)	30.4(26.7-33.5)
Maranhão	759.8(674.3-858)	49.8(42.8-56)
Mato Grosso	514.2(468.1-566.2)	30.8(27.2-33.7)
Mato Grosso do Sul	505(455.8-558.7)	31.1(26.9-34.8)
Minas Gerais	435.4(389.6-474.2)	26.4(23.3-28.9)
Pará	670.8(580.7-786.5)	41(34-47.8)
Paraíba	464.1(421.5-510.6)	29.1(25.8-31.8)
Paraná	586(532.3-638)	36.9(33.6-40.5)
Pernambuco	505.3(453.8-556.3)	31.8(27.5-34.9)
Piaui	602.4(523.5-672.7)	38.7(31.6-43.7)
Rio de Janeiro	497.1(444.2-551.7)	29.8(26-33)
Rio Grande do Norte	407.1(359.5-449.7)	25(21.1-27.9)
Rio Grande do Sul	566.6(515.1-614.4)	34.5(30.6-37.6)
Rondônia	514.3(451.9-584.8)	32.5(27.6-36.9)
Roraima	716(609.1-849.4)	48.3(41.2-55.7)
Santa Catarina	491.6(434.5-534.6)	32.1(27.7-35.3)
São Paulo	461.3(410.5-505.7)	28.6(24.3-31.6)
Sergipe	555.7(505.6-603.1)	35.1(30.8-38.7)
Tocantins	622.8(563-700.3)	39.5(34.3-44.9)

Source: Data derived from Global Burden of Disease Collaborative Network. Global Burden of Disease (GBD) Cardiovascular Burden Estimates 1990 and 2021, Institute for Health Metrics and Evaluation, University of Washington. ^48^

**Table:** Abbreviations Used in Chapter 2

ACEI/ARB	Angiotensin Converting Enzyme/Angiotensin Receptor Blocker
BRIDGE-Stroke	Brazilian Intervention to Increase Evidence Usage - Stroke
CI	Confidence Interval
CVD	Cardiovascular Diseases
DALY	Disability-Adjusted Life Years
ICD-10	International Statistical Classification of Diseases and Related Health Problems, 10^th^Revision
ICU	Intensive Care Unit
IMPACT-AF	Improve Treatment with Anticoagulants in Patients with Atrial Fibrillation
IRR	Incidence Rate Ratio
MAPS	Matão Preventing Stroke Study
OR	Odds Ratio
PURE	Prospective Urban Rural Epidemiology
RR	Relative Risk
SIM	Brazilian Mortality Information System (in Portuguese, *Sistema de Informações sobre Mortalidade* )
YLD	Years Lost for Disease
YLL	Years of Life Lost

## Introduction

•Stroke represents one of the main morbidity and mortality causes in Brazil. In recent years a growing representation of Brazilian generated evidence has been contributing to a better understanding of the stroke burden. Moreover, tackling stroke statistics represents a key step towards the development of evidence-based care pathways.

## Prevalence

•Stroke prevalence estimates may differ slightly between studies because each study selects and recruits a sample of participants to represent the target study population (e.g., state, region, or country). In community-based studies in the city of São Paulo, the age-adjusted prevalence rate for men was 4.6% (95% CI: 3.5-5.7) and for women 6.5% (95% 5.5-7/5)^69^in 2011, while, in another study in the same city, out of 3577 subjects, 244 cases (6.82%) of stroke survivors were identified.^70^In the town of Coari, authors have found a crude prevalence of stroke of 6.3% in rural areas and of 3.7% in urban areas.^71^From a nationally representative community-based epidemiological survey (National Health Survey - 2013), Bensenor *et al.* have estimated 2 231 000 stroke cases, representing prevalence of 1.6% and 1.4% in men and women, respectively.^72^

## Incidence

•In the Matão stroke registry study between 2003–2004 and 2015–2016, the age-adjusted incidence decreased by 39% (IRR 0.61; 95% CI, 0.46-0.79) and mortality, by 50% (IRR 0.50; 95% CI, 0.31-0.94), while 7% had a recurrent stroke.^73^

## Mortality

•Data from the GBD Brazil Group have shown that the age-standardized mortality rate from ischemic stroke per 100 000 in 2021 was 31.7 (28 to 33.9) ( **Table 2-1 and Chart 2-1** ). The highest mortality was observed in the Maranhão state, 49.8 (42.8 to 56), and the lowest, in the Rio Grande do Norte state, 25 (21.1 to 27.9).

**Chart 2-1:** – Age-standardized mortality rate due to ischemic stroke (A), intracerebral hemorrhage (B), and subarachnoid hemorrhage (C) per 100 000 inhabitants, 2021. Data from the Global Burden of Disease 2021 Estimates. ^48^

•Trends of mortality rates and DALYs for cerebrovascular disease (ICD-10: I-60-69), as per the GBD Brazil 2015, have shown that the annual reduction in age-adjusted mortality rates, for both sexes, slowed between 2005 and 2015 as compared to the previous period of 1990 to 2005.

•In the city of São Paulo, from 1996 to 2011, age-adjusted mortality rates for cerebrovascular diseases decreased by 46.6% in men and by 47.8% in women; for both sexes and age group of 35–74 years, the decline in age-adjusted rates was more pronounced among those residing in the wealthiest areas as compared to those living in the poorest ones.^74^A study using data from the SIM, from 1990 to 2012, has shown a –48.05% variation in the mortality coefficient.^75^In the MAPS study, between 2003–2004 and 2015–2016, mortality increased by 50% (IRR 0.50; 95% CI, 0.31–0.94). The 1-year case fatality was 26% and approximately 56% of the patients were functionally independent,^73 , 76^while 7% had a recurrent stroke.^73^

•In a study conducted in the Paraná state, the age- and sex-specific mortality rates related to stroke between 2007 and 2016 increased from 138 to 163 per 100 000 inhabitants, and individuals aged over 79 years had the highest increase. However, the mortality rates decreased for the age groups of 34-44 years and 44-54 years.^77^

### Global Burden of Cerebrovascular Diseases

#### YLL

•A study conducted in the Southeastern Region of Brazil in 2019 using secondary data from the Brazilian Unified Health System and the Brazilian Institute of Geography and Statistics showed a total of 713 132 estimated DALYs, with 80% attributed to YLDs and 20% to YLLs. When adjusted for population size, the highest impact was observed in the male sex in the Minas Gerais state, particularly in the age group of 70-79 years.^78^

#### DALY

•Data from the GBD Brazil Group have shown that the age-standardized DALY rate per 100 000 due to ischemic stroke in 2021 was 511.4 (470.6 to 542.5).^11^The highest DALY rate was observed in the Maranhão state, 759.8 (674.3 to 858), and the lowest, in the Rio Grande do Norte state, 407.1 (359.5 to 449.7) ( **Table 2-1 and Chart 2-2** ).

**Chart 2-2:** – Age-standardized DALY rate due to ischemic stroke (A), intracerebral hemorrhage (B), and subarachnoid hemorrhage (C) per 100 000 inhabitants, 2021. Data from the Global Burden of Disease 2021 Estimates. ^48^

## Complications and Associated Diseases

•Assessments from the community-based epidemiological survey (National Health Survey-2013) have shown a prevalence of post-stroke disabilities of 29.5% for men and of 21.5% for women, with only 0.27% of the individuals undergoing physical therapy for stroke.^79^

•A cross-sectional, population-based study has been conducted in Brazil using data from the Brazilian Longitudinal Study of Aging 2015-2016. It included 536 individuals aged 50 years and older who had experienced a stroke.^80^The prevalence of stroke was found to be 5.3% among individuals aged 50 years and older, increasing to 8.0% among those aged 75 years and older, with differences between sexes. Factors associated with independence in daily activities included walking speed, physical activity, and use of walking devices. A significant interaction was found between walking speed, home adaptations, and performance of daily living activities. The probability of independence was highest among fast walkers (> 0.8 m/s), who also had home adaptations. Faster walking speed combined with home adaptations emerged as the primary factor associated with long-term independence after a stroke.

## Quality of Care

•Regarding hospitalizations due to stroke, the study by Brant *et al* . has observed a decrease of 5.1% (RRi 0.949; 95% CI, 0.947-0.952) during the COVID-19 pandemic period (epidemiological week 10 to 21), the lowest relative decrease in the number of hospitalizations in the CVD group. Thus, although stroke is the second cause of hospitalization in absolute numbers, the reduction in the number of hospitalizations was lower than that for acute coronary syndrome. In parallel with this finding, ICU admissions for stroke showed a higher total (RRi 1154; 95% CI, 1145-1163) and proportional (RRi 1216; 95% CI, 1210-1222) increase than those for CVD together. However, although an increase in proportional mortality was observed, especially in the third phase of the pandemic, that was lower than for CVD alone (RRi 1053; 95% CI, 1048-1058) and did not recover until the end of the period evaluated. This progression of increase in numbers and proportion of hospital deaths throughout the pandemic period deserves to be highlighted, mainly because the proportion of ICU use remained higher than expected throughout the period evaluated.^15^

•Dantas *et al* . have performed a study to assess the stroke-related hospitalization at the Brazilian Unified Health System from 2009 to 2016, when the number of admissions increased from 131 122 to 146 950 and the absolute number of in-hospital deaths increased from 28 731 to 31 937. Younger age and male sex were significantly associated with patient survival. The annual age-adjusted hospitalization and in-hospital mortality rates decreased by 11.8% and 12.6%, respectively, but the case fatality rate increased for patients older than 70 years.^81^

•A study assessing sociodemographic factors related to lack of hospital care for cerebrovascular disease deaths in the state of São Paulo during the periods of 1996-1998 and 2013-2015 has shown that, of the 127 319 individuals who died due to stroke during the mentioned periods, 19 362 (15.2%) had no hospital care. In the latter period, a higher risk for death without care was identified for individuals of yellow races (RR = 1.48; 95% CI, 1.25-1.77), and a lower risk for black individuals (RR = 0.86; 95% CI, 0.76-0.95), married individuals (RR = 0.70; 95% CI, 0.64-0.75), and for those living in the city of São Paulo (RR = 0.92; 95% CI, 0.86-0.98).^82^

•An analysis of the stroke units in two centers from the cities of Curitiba and Botucatu for the key performance indicators required by the Brazilian Ministry of Health has shown that both centers admitted over 80% of the patients in their stroke units.^83^

•A cluster randomized trial assessing the effect of a multifaceted quality improvement intervention on adherence to evidence-based therapies for care of patients with acute ischemic stroke and transient ischemic attack (BRIDGE-Stroke) has shown that 402 of 817 patients (49.2%) at intervention hospitals received all the treatments they were eligible for as compared to 203 of 807 patients (25.2%) in control hospitals (OR, 2.59; 95% CI, 1.22-5.53; P= .01).^84^

•The RESILIENT study conducted in the Brazilian public healthcare system to assess the safety and efficacy of thrombectomy within that system has shown that this procedure, in conjunction with standard care, resulted in better functional outcomes at 90 days compared to standard care alone.^85^The standard OR for a better distribution of scores on the modified Rankin scale was 2.28, favoring thrombectomy. The percentage of patients with no or minor neurologic deficit was significantly higher in the thrombectomy group.

## Cost

•The costs related to hospital admissions for stroke and adjusted for inflation from 2008 to 2021 sum up to US$ 1 195 975 877.98, varying from US$ 28 661 321.88, in 2008, to US$ 66 843 953.39, in 2021.

•A cost-effectiveness study, assessing thrombolytic drugs in Brazil, has observed that, for a one-year result, for men, the cost of treatment with rt-PA is higher than the cost of the conservative treatment. The cost of the medication mainly directs this result. Part of this additional cost is compensated by the lower cost of rehabilitation and less productivity losses as early as in the first two years, as the patients treated with rt-PA (alteplase) presented fewer sequelae than those who received conservative treatment. After the second post-stroke year, for both sexes, treatment with rt-PA, considering direct and indirect costs, started to present a lower cost when compared to conservative treatment. From this time horizon onward, the additional cost of the medication begins to be more than compensated by the higher number of minor productivity losses and lower costs of social security and patient rehabilitation.^86^

•The cost-utility analysis from the RESILIENT trial comparing mechanical thrombectomy plus standard medical care with standard medical care alone has shown that the incremental costs and quality-adjusted life years gained were estimated at Int$ 7440 and 1.04, respectively, resulting in an incremental cost-effectiveness ratio of Int$ 7153 per quality-adjusted life year. Despite the initially higher costs, the long-term benefits of mechanical thrombectomy justified its incorporation into the stroke centers of the Brazilian public healthcare system, because it is likely to be cost-effective.^87^

## Risk Factors and Prevention

•Data from the PURE study, examining rates and predictors of use of evidence-based secondary prevention medications, have shown that fewer stroke patients received antiplatelets (24.3%), ACEI/ARB (37.6%), and statins (9.8%) as compared to chronic heart disease patients (30.1%, 36.0%, and 18.0%, respectively). Moreover, the use of no medication was observed in 30% of Brazilian stroke patients.^88^

•In the IMPACT-AF, a clustered randomized trial to IMProve treatment with AntiCoagulanTs in patients with Atrial Fibrillation, 91% of the patients from Brazil were on oral anticoagulation at baseline, and 27% on novel oral anticoagulants. Of all patients taking vitamin K antagonists in Brazil, 40.3% had international normalized ratio values between 2 and 3 prior to the baseline visit.^89^

•Using data from the GBD Study, Silva *et al* . have assessed the impact of low levels of physical activity on stroke mortality in Brazil from 1990 to 2019.^24^There was a reduction of 44.0% (males) and 52.0% (females) in age-standardized mortality rates due to stroke attributable to low levels of physical activity. The study suggests that higher levels of physical activity might have avoided approximately 6.1% (males) and 7.3% (females) of stroke deaths in 2019.^90^

•In a cross-sectional study analyzing anonymized data routinely collected by community health workers in Brazil between May 2016 and September 2021, from a population of 2 133 900 individuals in the database, 11 628 had had a previous myocardial infarction (33.1%) or stroke (n = 25 925; 73.9%). Only 6.7% (2346) and 0.6% (212) reported statins and high-dose statins use, respectively. Age over 60 years, living in the Southern region, previously diagnose of myocardial infarction, heart failure, diabetes, dyslipidemia, chronic kidney disease, and use of anti-hypertensives were associated with statin use.^91^

## Awareness and Treatment

•In recent years, there have been several initiatives to improve awareness of stroke in the Brazilian population, mainly around the World Stroke Day (October 29th) annual campaigns led by the World Stroke Organization.^92^Despite these efforts, several studies have shown an alarming lack of knowledge about stroke risk factors and treatment, and recognition of stroke symptoms as a medical emergency. Pontes-Neto *et al* . have shown that 32% of the interviewed subjects recognized no warning sign of stroke, only 34.6% of the subjects answered the correct nationwide emergency telephone number in Brazil (#192), and only 51.4% of the interviewees reported they would call an ambulance for a relative with symptoms of stroke.^93^

•In a study conducted in the city of Caxias do Sul, lower income and lower educational level were independent predictors of inability to recognize that stroke affects the brain.^94^While, in another study from the city of Santa Maria, Rio Grande do Sul, where 33% correctly identified the acronym “AVC”, meaning stroke in Portuguese, approximately 30% of the subjects incorrectly localized stroke in the heart.^95^

•In a study conducted in São Paulo, investigators surveyed students from different educational levels, including 1187 elementary-school students (mean age: 13 years, prior experience: 14%, 51% women), 806 high-school students (mean age: 17 years, prior experience: 13%, 47% women), and 1961 university students (mean age: 22 years, prior experience: 9%, 66% women). Stroke awareness and knowledge about risk factors and warning signs ranged between 42% and 66% of the students. Less than 52% of the students associated stroke with risk factors, such as hypercholesterolemia, smoking, diabetes, and hypertension. When prompted, 62% to 65% of the students recognized arm weakness, facial drooping, and speech difficulty as stroke warning signs, but only 43% identified acute headache. Notably, 67% of the students were aware of the emergency medical service number. Higher education, prior experience, and being female were associated with higher scores in identifying stroke risk factors (OR = 1.28; 95% CI, 1.10‒1.48; OR = 2.12; 95% CI, 1.87‒2.40; OR = 1.46; 95% CI, 1.16‒1.83; respectively) and warning signs/symptoms (OR = 2.22; 95% CI, 1.89‒2.60; OR = 3.30; 95% CI, 2.81‒3.87; OR = 2.04; 95% CI, 1.58‒2.63; respectively).^96^

•In a cross-sectional study conducted in high schools in Northeastern Brazil, Rodrigues *et al* . have aimed to assess stroke knowledge among high-school students.^97^Those authors have found that 80% of the students did not have the minimum knowledge on how to act in a stroke situation, while only 10% had the ideal knowledge on how to act. Males had lower levels of knowledge on risk factors and signs and symptoms of stroke. Students with more than 10 years of schooling and those aged 18 years demonstrated greater knowledge in certain aspects. That study highlights a knowledge deficit among high-school students regarding stroke recognition and activation of emergency medical services.

## Impact of the COVID-19 Pandemic

•Regarding hospitalizations for stroke, there was a 5.1% decrease during the period, representing the lowest relative decrease in the number of hospitalizations in the CVD group. Thus, although stroke is the second cause of hospitalization in absolute numbers, the reduction in the absolute number of hospitalizations was smaller than that for acute coronary syndrome. In parallel with this finding, there was a greater absolute (15.4% vs. 9.0%) and proportional (21.6% vs. 8.6%) increase in admissions to the ICU for stroke as compared to that for CVD combined. However, although an increase in proportional mortality was observed, especially in the third phase of the pandemic, it was lower than that for CVD alone (5.3% vs. 14.4%) and did not recover until the end of the period evaluated. This progression of increase in both numbers and proportion of in-hospital deaths throughout the pandemic period deserves to be highlighted, mainly because the proportion of ICU use remained higher than expected throughout the period evaluated. Taken together, these findings suggest that: a) possibly, due to the more striking and exuberant clinical symptoms in the acute phase of stroke, a higher proportion of patients maintained the tendency to seek urgent and emergency units, compared to those with acute coronary syndrome – a condition often with more nonspecific symptoms, especially in subgroups such as females, elderly, and individuals with diabetes. This may have contributed to the lower reduction in hospitalizations during the pandemic; and b) individuals with stroke were hospitalized with greater clinical severity or had worse results from treatment, possibly due to the compromise of the lines of health care.^15^

•Regarding stroke mortality, that same study has shown that stroke was the specific cause with the lowest reduction in mortality in the country, and there was no variation in the Northern and West-Central regions. These findings align with the relatively low decrease in hospitalizations, suggesting a lower real impact of the pandemic on cerebrovascular diseases. The clinical symptoms being different from those of COVID-19 might have allowed for a more accurate diagnosis. In addition, the lower direct relationship between the time to start treatment and mortality due to stroke allows for a longer time for the patient to recognize the symptoms and arrive at a hospital, differently from acute coronary syndrome, in which most deaths occur in the first hours after the event.^15^

## Future Directions

•The Brazilian research portfolio in vascular neurology has evolved largely in recent years as illustrated by the foundation of the Brazilian Stroke Research Network and the growing evidence generation directed to a variety of subjects and conducted by different groups. Still, there are several opportunities for further development.

•The most expressive community studies on stroke prevalence and incidence derive mostly from two cities. While both examples represent a major achievement in stroke epidemiology, there is still need for a broader assessment comprehending a representation of all geographical regions, diverse cultures, and income levels. As stroke continues to represent a major healthcare problem in Brazil, advancing evidence generation of new effective and safe stroke interventions is key. Thus, innovative models, such as virtual and decentralized patient-centric research, and the use of real-world data are proposed valuable strategies in the pursuit of a more effective learning-doing cycle.^98 , 99^

## CHAPTER 3 – ACUTE AND CHRONIC CORONARY HEART DISEASE

### ICD-9-CM 410 to 414; ICD-10 I10 to I25

**See Tables 3-1 through 3-3**

**Table:** Abbreviations Used in Chapter 3

ACCEPT	Brazilian Registry of Clinical Practice in Acute Coronary Syndromes
ACS	Acute Coronary Syndrome
AMI	Acute Myocardial Infarction
B-CaRe:QCO	Brasilia Cardiovascular Registry for Quality of Care and Outcomes
BRACE	Brazilian Registry in Acute Coronary Syndromes
BYPASS	Brazilian Registry of Adult Patients Undergoing Cardiovascular Surgery
CABG	Coronary Artery Bypass Grafting
CAC	Coronary Artery Calcification
CCS	Chronic Coronary Syndrome
CHD	Coronary Heart Disease
CI	Confidence Interval
CVD	Cardiovascular Disease
DALY	Disability-Adjusted Life Years
DATASUS	Brazilian Unified Health System Database
ERICO	Strategy of Registry of Acute Coronary Syndrome
GBD	Global Burden of Disease
IHD	Ischemic Heart Disease
IQR	Interquartile Range
MASS	Medicine, Angioplasty, or Surgery Study
OR	Odds Ratio
PCI	Percutaneous Coronary Intervention
PNS	National Health Survey (in Portuguese, *Pesquisa Nacional de Saúde* )
REPLICCAR-I	São Paulo's Cardiovascular Surgery Registry (in Portuguese, *Registro Paulista de Cirurgia Cardiovascular* )
REPLICCAR-II	São Paulo's Cardiovascular Surgery Registry II (in Portuguese: *Registro Paulista de Cirurgia Cardiovascular II* )
SAMU	Urgent Medical Care Service (in Portuguese: *Serviço de Atendimento Médico de Urgência* )
SIH	Brazilian Hospital Information System (in Portuguese, *Sistema de Informações Hospitalares* )
SIM	Brazilian Mortality Information System (in Portuguese, *Sistema de Informação sobre Mortalidade* )
STEMI	ST-Elevation Myocardial Infarction
SUS	Brazilian Unified Health System (in Portuguese, *Sistema Único de Saúde* )
UI	Uncertainty Interval
VICTIM	*Via Crucis* for the Treatment of Myocardial Infarction
YLD	Years Lived with Disability
YLL	Years of Life Lost

### Overview, Prevalence, and Incidence

•Coronary heart disease, also known as IHD, comprises a spectrum of symptomatic and asymptomatic clinical conditions typically related to a blood flow reduction in the heart muscle. The most common cause is atherosclerotic disease in the coronary arteries, a chronic condition with variable presentations, progressing from a long asymptomatic phase to stable angina, unstable angina, and AMI. Coronary heart disease is a common cause of heart failure, with reduced or preserved left ventricular ejection fraction, ventricular arrhythmias, and sudden cardiac arrest.

•Coronary heart disease was the leading cause of death in Brazil in the last decade, for men and women. Because of its wide range of clinical presentations, the reported CHD prevalence, incidence, and mortality vary widely, depending on the population and healthcare setting studied.

•Estimates of CHD prevalence and incidence in 2019 and historical series from 1990 to 2019, according to the GBD study, are available in the 2021 version of the Cardiovascular Statistics - Brazil.^4^

•The self-reported prevalence of CHD was 2.7% in the baseline assessment of the ELSA-Brasil cohort in 2008-2010. This cohort was composed of more than 15 000 civil servants aged 35-74 years from six cities (Salvador, Vitória, Belo Horizonte, Rio de Janeiro, São Paulo, and Porto Alegre). The prevalence was higher in males and individuals in the lower socioeconomic level.^100^

•According to the PNS 2019, an epidemiological nationwide home-based survey, overall prevalence rates of mild (class I) and moderate/severe (class II) angina among adults were 8.1% (95% CI, 7.8%-8.4%) and 4.5% (95% CI, 4.2%-4.7%). The prevalence of self-reported angina was higher in females and inversely proportional to formal education years. Compared to individuals who declared themselves white, mild angina was more prevalent in those self-reported black, while moderate/severe angina was more frequent in those self-reported brown.^101^It is important to note that the prevalence rates of angina observed in surveys are higher than the CHD prevalence obtained from epidemiological studies or national statistics. Self-reported assessments of angina are very sensitive but not specific to CHD because they require neither confirmatory exams nor health reports. In addition, national statistics might underrepresent the true epidemiology of CHD, considering its asymptomatic nature.

•In registries and one cluster-randomized study involving patients with ACS recruited from 2007 to 2014, the percentages of individuals with unstable angina, non-STEMI, and STEMI were 15-30%, 31-36%, and 36-54%, respectively.^102 - 105^

•Coronary artery calcification has been evaluated in 3616 individuals without CVD or diabetes mellitus from the ELSA-Brasil multiracial cohort. As expected, CAC increased with age and was more prevalent in males than females. No identifiable CAC was observed in 64% of males, 83% of females, 93% of younger individuals (35-44 years), and 34% of older participants (65-74 years). Conversely, CAC >100 ranged from 1.2% of the individuals aged 35-44 years to 28% of those aged 65-74 years. CAC >400 was not detected among participants aged 35-44 years but was seen in 12% of those aged 65-74 years. Significant differences in CAC distribution according to race were noted. For example, among participants aged 65-74 years, the point estimate for the 50^th^CAC percentile was 14 in black, 17 in brown, and 32 in white persons.^106^

### Mortality

•Estimates of the number of deaths and crude mortality rate attributable to CHD for the year 2019 and historical series from 1990 to 2019, according to the GBD study, are available in the 2021 version of the Cardiovascular Statistics - Brazil.^4^

•According to the GBD estimates for the year 2021, the age-standardized mortality rate due to CHD was 67.1 (95% UI, 60.9-71.0) per 100 000 inhabitants in Brazil ( **Table 3-1** ). The lowest estimated rates were observed in the states of Minas Gerais (50.7 [95% UI, 44.6-55.2]), Amazonas (54.1 [95% UI, 48.3-59.4]), and Bahia (56.0 [95% UI, 49.3-63.0]), whereas the highest rates were estimated in the states of Rio de Janeiro (81.3 [95% UI, 71.6-89.9]), Pernambuco (81.8 [95% UI, 72.4-89.0]), and Maranhão (89.0 [95% UI, 78.7-99.2]) per 100 000 inhabitants ( **Table 3-1** ).^48^

**Table 3-1:** – Estimates of age-standardized mortality and DALY rates (per 100 000) due to coronary heart disease in Brazil and its Federative Units in 2021

	Mortality	DALY
Brazil	67.1 (60.9;71.0)	1432.5 (1345.3;1511.2)
Acre	67.3 (61.8;72.6)	1344.1 (1256.4;1451.6)
Alagoas	80.1 (71.0;90.7)	1731.7 (1547.7;1969.4)
Amapá	68.4 (59.8;77.5)	1381.4 (1223.7;1554.6)
Amazonas	54.1 (48.3;59.4)	1117.4 (998.0;1210.4)
Bahia	56.0 (49.3;63.0)	1216.2 (1066.7;1363.9)
Ceará	70.1 (60.2;79.7)	1434.2 (1220.1;1636.2)
Distrito Federal	61.4 (54.2;67.1)	1049.1 (939.5;1153.0)
Espírito Santo	65.6 (57.4;71.5)	1369.2 (1227.2;1499.2)
Goiás	65.3 (58.3;71.6)	1403.7 (1291.0;1548.4)
Maranhão	89.0 (78.7;99.2)	1850.9 (1620.1;2091.6)
Mato Grosso	60.3 (52.9;66.0)	1281.8 (1146.9;1410.7)
Mato Grosso do Sul	75.5 (68.5;84.1)	1610.5 (1470.1;1780.6)
Minas Gerais	50.7 (44.6;55.2)	1111.3 (983.7;1218.0)
Pará	67.9 (57.9;79.8)	1468.3 (1285.2;1728.1)
Paraíba	77.4 (69.9;83.6)	1658.3 (1508.4;1813.6)
Paraná	61.3 (55.7;66.5)	1277.9 (1181.0;1392.8)
Pernambuco	81.8 (72.4;89.0)	1752.5 (1582.0;1921.1)
Piauí	67.3 (58.6;76.5)	1442.9 (1267.8;1654.2)
Rio de Janeiro	81.3 (71.6;89.9)	1762.7 (1583.4;1935.2)
Rio Grande do Norte	71.6 (63.3;79.4)	1548.9 (1392.2;1715.0)
Rio Grande do Sul	58.6 (53.1;64.0)	1195.2 (1082.3;1308.8)
Rondônia	64.3 (56.1;72.0)	1309.8 (1151.0;1474.5)
Roraima	77.0 (66.2;89.1)	1503.6 (1277.2;1777.9)
Santa Catarina	61.2 (54.0;66.7)	1233.4 (1105.8;1349.6)
São Paulo	72.8 (64.4;80.2)	1568.9 (1431.1;1730.3)
Sergipe	57.3 (51.0;62.9)	1208.0 (1105.0;1315.9)
Tocantins	72.6 (63.3;82.5)	1531.7 (1339.5;1735.3)

Source: Data derived from Global Burden of Disease Collaborative Network. Global Burden of Disease (GBD) Cardiovascular Burden Estimates 1990 and 2021, Institute for Health Metrics and Evaluation, University of Washington. ^48^

•According to a time series study, the age-standardized mortality rate from AMI declined 44% in the country from 1996 to 2016, with substantial regional differences (percent changes: +5% in the North, +11% in the Northeast, -35% in the West-Central, -68% in the Southeast, and -85% in the South). Death rates were corrected for ill-defined causes of death, garbage codes, and underreporting. Temporal changes were more pronounced in females than in males, and in capital cities than in other municipalities.^107^

•Using data from the DATASUS, one study has reported a decrease in crude mortality rates from CHD in Brazil in both sexes and all age groups from 2000 to 2018, except for a 1.78% increase in males over 85 years of age. In this period, age-adjusted mortality rates from CHD decreased in both sexes. From 2000-2008 to 2016-2018, the average annual rate decreased from 97.09 to 78.75 per 100 000 considering both sexes, from 115.89 to 97.23 per 100 000 males, and from 79.82 to 61.91 per 100 000 females.^108^

•Using data from the DATASUS/SIM, Vieira *et al.* have reported a decrease in age-adjusted mortality rate due to AMI in the state of Minas Gerais from 35.7 (95% CI, 35.3-36.1) per 100 000 inhabitants in 2008 to 30.4 (95% CI, 30.1-30.6) per 100 000 in 2016. The mortality rates due to AMI were higher during the winter and lower during the summer.^109^

#### Mortality after acute coronary syndrome

•Several Brazilian ACS registries have reported outcomes of individuals admitted with ACS. In general, the mortality rate in registries is lower than that reported in the SIH/SUS. Several studies have highlighted regional differences in treatment practices and mortality, as well as poorer outcomes in patients admitted to public services as compared to those admitted to private hospitals.^102 - 104 , 110^

•Registries and studies involving patients with ACS from 2003 to 2017 have reported in-hospital mortality rates ranging from 3.3% to 10.9%.^103 , 104 , 110 - 113^These studies are described in detail in the 2021 version of the Cardiovascular Statistics – Brazil.^4^More recently, an in-hospital mortality rate of 5.6% has been reported among 2290 patients with STEMI who underwent a pharmaco-invasive strategy and were hospitalized in a university hospital in the city of São Paulo.^114^Moreover, among 763 patients with ACS (66.5% STEMI, 20.6% non-STEMI, 12.4% unstable angina) admitted to the Hospital das Clínicas of the Federal University of Minas Gerais between May 2016 and September 2019, the in-hospital mortality rate was 2.9%.^115^

•Using data from the DATASUS/SIM/SIH, Vieira *et al.* have estimated a decrease in age-adjusted in-hospital mortality among patients admitted with AMI from 13.81% in 2008 to 11.43% in 2016 in the state of Minas Gerais.^109^

•Recent studies have reported long-term outcomes after ACS. In the ACCEPT registry that included 4782 patients with ACS in 53 hospitals from the five Brazilian geopolitical regions from 2010 to 2014, the rate of major cardiovascular events was 13.6% after an 1-year follow-up.^102^Among 542 patients with STEMI enrolled in the Brasilia Heart Study, the mortality rate was 13.2% after a median 1.7-year follow-up. Low income was associated with higher all-cause mortality.^116^In the ERICO study, among 800 patients with ACS admitted to a university hospital in the city of São Paulo, who underwent invasive angiography from 2009 to 2013, the overall mortality rate was 17.5% after a median 4-year follow-up.^117^

•The B-CaRe:QCO registry included consecutive individuals with ACS admitted to public hospitals in Brasília-DF from January 2011 to February 2020. The patients (n=4099 aged >55 years, and n=2242 aged ≤55 years) underwent invasive coronary angiography within 48 hours from hospital admission. In-hospital deaths occurred in 5.7% and 3.6% of those >55 years and ≤55 years, respectively. Post-discharge death rates were 15.06 and 5.97 per 1000 patient-years among those aged >55 years and ≤55 years, respectively, after a median follow-up of 6.67 years.^118^

#### Mortality in chronic coronary syndrome

•In the single-center MASS III clinical trial, among patients with multivessel CHD, stable angina, and preserved left ventricular function who underwent CABG between 2001 and 2006, rates of death, AMI, revascularization, or stroke at 10 years were 30% for ‘on-pump’ surgery and 36% for ‘off-pump’ surgery.^119^

•In a retrospective analysis of the MASS database, among 1719 patients with stable multivessel CHD and preserved left ventricular function who underwent CABG, PCI, or medical treatment between 2002 and 2015, the 5-year all-cause mortality rates were 7.5%, 7.5%, and 12.3% among those with low, intermediate, and high SYNTAX score, respectively.^120^

•In a retrospective analysis of 1001 patients with multivessel CHD included in the MASS database between 1995 and 2018, overall mortality rates were 23% and 22% among those with or without myocardial ischemia, respectively, after a median follow-up of 8.7 years.^121^

#### Mortality related to percutaneous coronary intervention

•In registries and studies involving patients undergoing PCI from 2005 to 2016, mostly from public institutions, the in-hospital mortality rate went from 2.3% to 2.6%.^122 , 123^In the ICP-BR Registry, the in-hospital mortality rate ranged from 0.2% for patients with stable angina to 6.1% for those with STEMI.^123^

#### Mortality related to coronary artery bypass graft surgery

•In the BYPASS registry, among 2292 patients from all Brazilian regions who had undergone isolated or combined CABG until November 2018, the in-hospital mortality rate was 2.8%.^124^

•In the REPLICCAR-I registry, among 2961 patients who had undergone isolated CABG in 10 hospitals between 2013 and 2016, the all-cause mortality rate was 3.4% at 30 days and 5.3% at 4 years of follow-up.^125^

•In the prospective REPLICCAR II registry, among 3122 patients undergoing isolated CABG in 5 hospitals in the state of São Paulo between July 2017 and June 2019, a propensity score matching technique was used to match 1062 patients with single or multiple arterial graft treatment, adjusted for the mortality risk. Operative mortality was 1.88% in both groups.^126^

•In a single-center study in Southern Brazil with 1767 patients undergoing isolated CABG between 2013 and 2018, propensity score matching was used to create groups of patients undergoing ‘off-pump’ (n=397) and ‘on-pump’ (n=1370) CABG. The one-month mortality rates were 1.5% and 2.4% in the ‘off-pump’ and ‘on-pump’ groups, respectively (p=0.401).^127^

•Several other single-center experiences, with both retrospective and prospective analyses, have described short-term mortality rates ranging from 1.9% to 11.7% for patients who had undergone CABG.^128 - 131^

## Burden of Disease

•GBD estimates of the YLD, YLL, and DALY rates attributable to CHD for the year 2019 and historical series from 1990 to 2019 are available in the 2021 version of the Cardiovascular Statistics - Brazil.^4^

•The GBD estimated an age-standardized DALY rate due to CHD of 1432.5 (95% UI, 1345.3-1511.2) per 100 000 inhabitants for the year 2021 in Brazil. The lowest rates were estimated in Distrito Federal (1049.1 [95% UI, 939.5-1153.0]), Minas Gerais (1111.3 [95% UI, 983.7-1218.0]), and Amazonas (1117.4 [95% UI, 998.0-1210.4]), whereas the highest rates were observed in Pernambuco (1752.5 [95% UI, 1582.0-1921.1]), Rio de Janeiro (1762.7 [95% UI, 1583.4-1935.2]), and Maranhão (1850.9 [95% UI, 1620.1-2091.6] per 100 000 inhabitants) ( **Table 3-1** ).^48^

## Healthcare Utilization and Cost

•In a global modeling approach to assess the economic impact of four heart conditions in Brazil (hypertension, heart failure, AMI, and atrial fibrillation), AMI posed the greatest financial cost, with a health system cost per case of US$ 48 118 and a productivity cost of US$ 18 678.^132^

•According to a study published in 2008, the annualized cost for an individual with chronic CHD was estimated to be R$ 2733 ± 2307 by the SUS, with the outpatient cost being responsible for 54% of the total. For private insurance plans, the cost was estimated to be R$ 6788 ± 7842, 69% of which related to inpatient costs. For outpatient costs, medications were responsible for R$ 1154, representing, for public and private payers, 77% and 55% of the outpatient costs and 42% and 17% of the total cost, respectively.^133^

•Alexim *et al* . have analyzed data from 1088 young ACS patients who underwent coronary revascularization in public hospitals in Brasília-DF between 2013 and 2015. Direct costs for CABG and PCI were equivalent (Int$ 3141 [IQR: 15 392]/year and Int$ 3348 [IQR: 17 884]/year), while the cost due to labor productivity loss was higher in the PCI group (Int$ 4 511 [IQR: 18 062]/year *vs* . Int$ 3578 [IQR: 13 198]/year, p = 0.049) as compared to that in the CABG group.^134^

•According to administrative data from the public health system, the number of hospitalizations for CHD (acute and chronic) remained stable from 2015 to 2019, with more patients being admitted for ACS than for CCS. During 2020 and 2021, there was a ~13% reduction in clinical admissions. The annual number of admissions for PCI continuously increased from 38 635 in 2008 to 85 518 in 2019, decreased to an average of 79 018 per year in 2020-2021, and then increased to 80 909 in 2022. The annual number of hospitalizations for CABGs remained stable from 2008 (20 515) to 2019 (21 018), decreased to an average of 16 243 per year in 2020-2021 (a 26% decrease), and then increased to 19 565 in 2022.

•Several significant changes have occurred regarding reimbursement and mean cost per procedure for the management of CHD in the SUS. Although the number of PCIs performed in public hospitals more than doubled from 2008 to 2022, the average ticket dropped by half (adjusted values: from R$ 12 916 in 2008 to R$ 6 443 in 2022) ( **Table 3-2** ). A similar trend was observed for primary PCIs.^12^

**Table 3-2:** – Number and adjusted reimbursement values of coronary angioplasties performed in the SUS from 2008 to 2022

Year		Coronary Angioplasty	
**Number of procedures**	**Total amount paid (adjusted for inflation)**	**Mean value per procedure**
2008	38 635	R$ 499 028 630.90	R$ 12 916.49
2009	45 648	R$ 596 852 199.60	R$ 13 075.10
2010	49 492	R$ 634 390 961.40	R$ 12 818.05
2011	55 931	R$ 684 756 412.70	R$ 12 242.88
2012	60 959	R$ 707 818 064.00	R$ 11 611.38
2013	63 838	R$ 699 061 695.70	R$ 10 950.56
2014	66 492	R$ 697 953 632.50	R$ 10 496.81
2015	66 550	R$ 657 219 710.30	R$ 9 875.58
2016	69 802	R$ 624 864 836.60	R$ 8 951.96
2017	73 971	R$ 632 773 406.80	R$ 8 554.34
2018	78 575	R$ 653 083 595.90	R$ 8 311.60
2019	85 518	R$ 693 261 774.50	R$ 8 106.62
2020	77 846	R$ 620 573 346.40	R$ 7 971.81
2021	80 190	R$ 613 985 563.10	R$ 7 656.64
2022	80 909	R$ 521 362 010.90	R$ 6 443.81

Source: Ministry of Health of Brazil – Hospital Information System of the Unified Health System (SIH/SUS). ^49^

•The number of CABG for CHD in the SUS was similar in 2008 (20 515) and 2022 (19 565), and the adjusted amount reimbursed per CABG (mean ticket) decreased by ~23% (from R$ 20 339 in 2008 to R$ 15 723 in 2021).^49^

## Quality of Care

•Several publications addressing the quality of care in ACS in Brazil are described in detail in the 2021 version of the Cardiovascular Statistics – Brazil.^4^Those studies highlight opportunities for healthcare improvement, regional differences in quality care performance indices, and heterogeneity between public and private services.^102 , 103 , 110 , 113 , 135 - 139^For instance, in the ACCEPT registry, the rate of full adherence to guideline-recommended medications was only 62% soon after admission for ACS, and 18% of patients with STEMI were treated with neither fibrinolysis nor primary PCI.^102^In the BRACE study, the quality of care in ACS was poorer in the Northern and Northeastern regions than in the rest of the country.^103^In the VICTIM study, the mean time between symptom onset and hospital admission was longer and the rate of primary PCI was lower in public services as compared to private hospitals.^110^Importantly, low quality of care in ACS was associated with a higher risk of hard outcomes and in-hospital mortality in the ACCEPT and BRACE studies.^102 , 103^

•Some publications, described in detail in the 2021 version of the Cardiovascular Statistics – Brazil,^4^have reported the success of some strategies (e.g., incorporation of telemedicine) in improving the quality of care in ACS (e.g., increasing the rate of reperfusion therapy and the use of antiplatelet agents and statins, and reducing in-hospital mortality).^137 - 139^

•The implementation of SAMU, the Brazilian nationwide ambulance service, mildly decreased AMI mortality rate (OR = 0.967 [95% CI, 0.936-0.998]) and AMI in-hospital mortality (OR = 0.914 [95% CI, 0.845-0.986]) from 2008 to 2016 in the state of Minas Gerais.^109^

•Pivot times in the setting of a pharmaco-invasive strategy for STEMI have been reported by Bianco *et al.* who analyzed 2290 patients hospitalized in a university hospital in the city of São Paulo. The following times were found:

▫pain-health unit: median 120 (IQR 60-220) minutes;

▫door-needle: median 71 (IQR 42-135) minutes;

▫pain-needle: median 222 (IQR 140-345) minutes.^114^

•In the B-CaRe:QCO registry, ~90% of patients admitted with STEMI underwent a pharmaco-invasive strategy. Carvalho *et al.* have reported the following times for patients aged >55 years:

▫pain-primary hospital: mean 165 (standard deviation, 142) minutes;

▫door-needle: median 70 (IQR 43-120) minutes;

▫pain-needle: median 225 (IQR 150-335) minutes.

The respective times for patients aged ≤55 years were: mean 154 (standard deviation, 131), median 68 (IQR 43-111), and median 210 (IQR 140-315) minutes.11^8^

•In the B-CaRe:QCO registry, statin therapy at discharge was prescribed to 83% of those aged >55 years and 82% of those aged ≤55 years. The respective rates of acetylsalicylic acid prescription at discharge were 91% and 89%.^118^

•Passaglia *et al.* have reported adherence rates to evidence-based therapies among 763 patients with ACS admitted to the Hospital das Clínicas of the Federal University of Minas Gerais between May 2016 and September 2019. The patients were included in the ‘Good Practices in Cardiology’ program supported by the Brazilian Society of Cardiology. Adherence rates were above 85% for the following performance indicators: early use of acetylsalicylic acid; acetylsalicylic acid at discharge; angiotensin-converting-enzyme inhibitor or angiotensin receptor blocker at discharge for patients with left ventricular ejection fraction <45%; beta-blocker at discharge; statin therapy at discharge; and blood pressure control at discharge. Adherence to tobacco cessation counseling for active smokers at discharge was 81.5%.^115^

•Among 542 patients with STEMI enrolled in the Brasilia Heart Study, low-income individuals less frequently received dual antiplatelet therapy and statins at hospital discharge as compared to high-income patients.^116^

•Among 2290 patients with STEMI who underwent a pharmaco-invasive strategy and were admitted to a university hospital in the city of São Paulo, a higher prevalence of atypical symptoms at presentation, longer time between symptom onset and seeking care, and longer delay in receiving fibrinolysis were observed among females.^114^

•In a retrospective cross-sectional study with data collected by community health workers in the context of the primary care program, Family Health Strategy, between 2016 and 2021, of 35 103 individuals with prior myocardial infarction or stroke, only 6.7% and 0.6% reported use of statin and high-dose statin therapy, respectively. Statin use was associated with age over 60 years, living in the Southern region, and the presence of comorbidities (e.g., heart failure, diabetes mellitus, or chronic kidney disease).^91^

## Impact of the COVID-19 Pandemic

•An ecological time-series study has evaluated hospital admissions (SUS-only data) and mortality rates due to CVD during the COVID-19 pandemic in Brazil. Compared to average values in the three preceding years (2017-2019), hospital admissions due to CCS and ACS declined 12.8% (95% CI, 12.5%-13.2%) and 13.6% (95% CI, 13.3%-13.9%), respectively ( **Table 3-3** ). The in-hospital mortality related to both CCS and ACS increased, and the proportion of ACS patients admitted to ICU was higher during the pandemic period ( **Table 3-3** ), possibly reflecting more severe cases and/or delayed admission. Age-standardized mortality rates due to CCS and ACS per 100 000 inhabitants decreased 23% (95% CI, 21%-25%) and 6% (95% CI, 5%-7%), respectively ( **Table 3-3** ), although almost no change was observed when ACS was analyzed not only as the underlying, but also as an associated cause of death (1% [95% CI, 0%-2%] decrease). An increase in the proportion of home deaths was reported for both CCS and ACS ( **Table 3-3** ).^15^

**Table 3-3:** – Number of hospital admissions, percentage of in-hospital mortality, mortality rate (per 100 000 inhabitants), and percentage of home deaths due to chronic and acute coronary syndromes during the COVID-19 pandemic

		Observed (95% CI)	Expected (95% CI)	Risk ratio* (95% CI)
Chronic coronary syndrome	Hospital admissions (n)	115 250 (111 624;118 876)	132 173 (129 754;134 592)	0.872 (0.868;0.875)
	In-hospital mortality (%)	3.3 (3.2;3.4)	3.0 (2.9;3.0)	1.116 (1.098;1.134)
	Mortality rate	7.15 (7.04;7.27)	9.29 (9.16;9.42)	0.77 (0.75;0.79)
	Home deaths (%)	0,37 (0,36;0,38)	0,31 (0,31;0,32)	1.17 (1.16;1.19)
Acute coronary syndrome	Hospital admissions (n)	171 287 (166 244;176 330)	198 208 (196 571;199 845)	0.864 (0.861;0.867)
	Admissions to ICU (%)	26.5 (26.3;26.7)	24.8 (24.6;25.0)	1.068 (1.064;1.072)
	In-hospital mortality (%)	10.8 (10.6;10.9)	9.9 (9.8;10.0)	1.085 (1.077;1.093)
	Mortality rate	44.58 (44.29;44.86)	47.3 (47.01;47.59)	0.94 (0.93;0.95)
	Home deaths (%)	0,37 (0,36;0,37)	0,33 (0,33;0,34)	1.10 (1.10;1.11)


Epidemiological weeks: 10/2020-21/2021 for hospital data, 10/2020-11/2021 for mortality rate in the population. ICU: intensive care unit. * Risk ratio is the ratio between observed and expected values. Source: Brant et al. ^15^

•Brant *et al.* have compared observed and expected CVD deaths during the initial phase of the COVID-19 pandemic (March 17 to May 22, 2020) in the six Brazilian cities with the greatest numbers of COVID-19 deaths at that time (São Paulo, Rio de Janeiro, Fortaleza, Recife, Belém, and Manaus). Specified deaths due to ACS increased in Belém and Manaus but decreased in the other cities. Unspecified cardiovascular deaths increased in all cities and were correlated with home deaths. The net effect was an increase in the excess percentage of total cardiovascular deaths in all cities (especially Belém and Manaus), except Rio de Janeiro and Recife.^8^

•In a registry of patients from 16 hospitals in 6 different states, the number of individuals admitted to the emergency department with suspected ACS (especially non-ST elevation ACS) in the first three months of the COVID-19 pandemic decreased by 36.5% as compared to the monthly average in the 12 previous months.^140^

•In a time-series study in the setting of SUS, as compared to the period between March and May 2019, admissions due to AMI decreased by 4% from March to May 2020, whereas in-hospital deaths dropped 9% and fatality rate (percentage of deaths among the admissions) decreased by 5%.^141^

•In an ecological study of residents in the city of Belo Horizonte aged ≥30 years, no overall excess mortality due to ACS was found when comparing the observed age-standardized rate during epidemiological weeks 10–48, 2020, with the expected rates (mean of the period from 2015 to 2019). The authors reported reductions in the rates of hospital admission (RR 0.79; 95% CI, 0.75-0.84) and in-hospital deaths (RR 0.76; 95% CI, 0.57-1.01) due to ACS, as well as an increase in deaths at home (RR 1.38; 95% CI, 1.04-1.82).^66^

•In a time-series study in the city of Belo Horizonte, the number of hospitalizations due to ACS in the 2020 pandemic period was 21% lower than projected (n=2369 and 3013, respectively).^142^

•Admissions due to urgent and elective coronary events between March 18 and September 30, 2020, were compared to hospitalizations during a 2-year historical series (2018 and 2019) in the setting of the UNIMED-BH health plan, a private medical system covering approximately 1.31 million individuals (22% of the population) in the metropolitan area of Belo Horizonte. The numbers of admissions due to CHD were 2789, 3519, and 2348 in 2018, 2019, and 2020, respectively, indicating a 26% (95% CI, 22%-30%) reduction in 2020 as compared to the historical series. The in-hospital mortality rate was significantly higher in 2020 (5.4%; 95% CI, 4.5%-6.4%) as compared to 2018-2019 (3.6%; 95% CI, 3.2%-4.1%).^143^

## Future Directions

•Additional data are needed for further understanding of the epidemiological distribution of CHD in Brazil, in particular:

1. Development of nationwide databases aiming at gathering accurate and real-time information on the epidemiology of the distinct clinical presentations of CHD, including delivery of care, performance, and outcome measurements;

2. Analyses of disaggregated data to deeply understand diverse aspects of the CHD epidemiology and treatment according to sex, age, race, education, income, health system access, region, and other characteristics of the microenvironment;

3. Systematic reviews of prevalence and mortality rates of ACS, stable angina, PCI, and CABG, including representative samples of all geographical regions of the country, stratified by system delivery aspects;

4. Assessment of the effectiveness of structured nationwide programs for quality and performance measurement of different providers (public, not-for-profit, and for-profit) to understand the current situation, as well as to design strategies aimed at reducing CVD morbidity and mortality;

5. Additional economic and cost-effectiveness analyses of the impact of CHD and its diagnostic and therapeutic interventions, from a macro level and using micro-costing methods, for both public and private healthcare systems;

6. Development of structured programs to assess the prevalence, incidence, clinical, and economic impact of chronic CHD on the outpatient setting;

7. Analyses to evaluate the long-term impact of the COVID-19 pandemic on the CHD prevalence, incidence, mortality, burden, and treatment effectiveness.

## CHAPTER 4 – CARDIOMYOPATHY AND HEART FAILURE

### ICD-10 I42; I50; B57.2.

**See Charts 4.1 through 4.6**

**Table:** Abbreviations Used in Chapter 4

BREATHE	Brazilian Registry of Heart Failure
ChCM	Chagas Cardiomyopathy
ChD	Chagas Disease
CI	Confidence Interval
DALY	Disability-Adjusted Life Years
FU	Federative Unit
GBD	Global Burden of Disease
HF	Heart Failure
HR	Hazard Ratio
ICD-10	International Statistical Classification of Diseases and Related Health Problems, 10^th^Revision
RR	Relative Risk
SEADE	Data Analysis State System Foundation (in Portuguese, *Fundação Sistema Estadual de Análise de Dados* )
SUS	Brazilian Unified Health System (in Portuguese, *Sistema Único de Saúde* )
T. cruzi	Trypanosoma cruzi
UI	Uncertainty Interval
US	United States
YLDs	Years Lived with Disability
YLLs	Years of Life Lost

### Cardiomyopathy and Myocarditis

#### Prevalence and Incidence

•According to the GBD Study 2019 estimates, which are the last complete data available, cardiomyopathy and myocarditis age-standardized prevalence decreased in Brazil by 4.7% (95% UI, -9.5;0.8) from 1990 to 2019. In absolute numbers, estimates for the prevalence of cardiomyopathy and myocarditis in Brazil increased from less than 60 000 in 1990 to over 160 000 in 2019, mainly due to population growth and aging. More details on cardiomyopathy and myocarditis data from the GBD Study 2019 are available in the 2021 version of the Cardiovascular Statistics - Brazil.^4^

#### Mortality

•According to the GBD 2021 estimates, the mortality rate due to myocarditis in Brazil was 0.2 (95% UI, 0.2-0.2), and the highest rates were observed in Roraima (0.7; 95% UI, 0.6-0.8) and Goiás (0.4; 95% UI, 0.3-0.4). Furthermore, according to the GBD 2021 estimates, the mortality rate due to other cardiomyopathies was 7.0 (95% UI, 6.3-7.4). The FUs with the lowest mortality rates were Rio Grande do Norte (3.6; 95% UI, 3.1-4), Rio Grande do Sul (3.6; 95% UI, 3.1-4), and Amazonas (4.0; 95% UI, 3.5-4.4), while the FUs with the highest rates were Roraima (12.8; 95% UI, 10.7-15.1), São Paulo (11.3; 95% UI, 10-12.2), and Rio de Janeiro (9.7; 95% UI, 8.7-11). As the GBD 2021 data do not represent a historical series, they cannot be compared with previous data. The latest available full GBD data are from 2019.

•According to the GBD Study 2019 estimates, the mortality rates due to cardiomyopathy and myocarditis seemed to increase in the 90s but decreased in the two following decades. Death due to HF related to other specific causes are attributed to the underlying disease, i.e., deaths related to ischemic cardiomyopathy are coded as due to ischemic heart disease. Moreover, for the GBD project, HF is not considered a primary cause of death, thus, all deaths coded as related to HF should be recoded to the baseline condition (see below). More details on cardiomyopathy and myocarditis mortality from the GBD Study 2019 are available in the 2021 version of the Cardiovascular Statistics - Brazil.^4^

•In a study reporting data from the SEADE, from the state of São Paulo, cardiomyopathies were responsible for a total of 3571 deaths, representing 23.3% of HF-related deaths in 2006: dilated cardiomyopathy accounted for 17.2% of the deaths; alcoholic cardiomyopathy, for 0.45%; and restrictive cardiomyopathies, for 0.37%. Chagas disease and alcoholic cardiomyopathy were responsible for 7.8% and 0.45% of the HF-related deaths, respectively.^144^

•For specific cardiomyopathies, data are scarce. A cohort study of 214 patients with hypertrophic cardiomyopathy has reported data from a 7-year follow-up in a tertiary hospital in São Paulo, Brazil. The mean age was 37±16 years, and 52% were women. There were 22 deaths (10%), 15 directly related to hypertrophic cardiomyopathy (11 sudden deaths). The cumulative survival rates were 94.5% at 5 years, 91% at 10 years, and 87.9% at 15 years, with an annual mortality rate of 1%, which is low, considering those were patients in a referral center.^145^

#### Burden of Disease

•According to the GBD 2021 estimates, the DALY rate due to myocarditis was 8.7 (95% UI, 7.7-9.8), and the highest rates were observed in Roraima (23; 95% UI, 20.1-26.4), Goiás (12.6; 95% UI, 10.7-14.8), and Paraná (12; 95% UI, 10.4-13.8). Furthermore, according to the GBD 2021 estimates, the DALY rate due to other cardiomyopathies was 184.2 (95% UI, 174.4-194.3). The FUs with the lowest DALY rates were Rio Grande do Sul (86.1; 95% UI, 77.3-94.5) and Rio Grande do Norte (96.8; 95% UI, 87.8-106.2), while the FUs with the highest DALY rates due to other cardiomyopathies were São Paulo (303.1; 95% UI, 275.2-332.4), Roraima (281; 95% UI, 238.3-331.5), and Rio de Janeiro (279.1; 95% UI, 254.1-310.6). As the GBD 2021 data do not represent a historical series, they cannot be compared with previous data. The latest available full GBD data are from 2019 and were discussed in the 2021 Cardiovascular Statistics – Brazil.^4^In brief, according to the GBD 2019 estimates, the trends of age-standardized DALY rates due to cardiomyopathy and myocarditis were similar to those of mortality, with a small increase in the 90s and a decrease during the following decades.

## Chronic Chagas Disease and Chagas Cardiomyopathy

### Prevalence and Incidence

•The 2010 prevalence of ChD in Brazil was estimated at 1 156 821 by the World Health Organization^146^in the last official available estimate published in 2015. According to that statement, the estimated number of individuals with ChCM in Brazil is 231 364 subjects. These numbers revealed a significant decreasing trend of ChD human cases in Brazil in relation to previous estimates, which was attributed to various factors, but specially to the almost complete interruption of the vectorial and transfusion-related transmission in Brazil. That decrease is reported in the 2021 version of the Cardiovascular Statistics - Brazil based on the GBD 2019 estimates.^4^

•A systematic review including 42 articles has confirmed a reduction in the prevalence of ChD.^147^However, about 4.6 million (95% CI, 2.9-7.2 million) people were estimated to be infected with *T. cruzi* in 2010. These estimates are much higher than those from the World Health Organization for 2010.^146^

•In the NIH REDS-II Chagas retrospective cohort study, initially healthy blood donors with an index *T. cruzi* -seropositive donation and age, sex, and period-matched seronegative donors were followed up for 10 years.^148^The differential incidence of cardiomyopathy was 1.85 per 100 person-years attributable to *T. cruzi* infection. When prospectively followed up for a further decade, from 2008–2010 to 2018–2019, the incidence of cardiomyopathy in *T. cruzi* -seropositive donors was 13.8 (95% CI, 9.5–19.6) events/1000 person-years (32/262, 12%) as compared to 4.6 (95% CI, 2.3–8.3) events/1000 person-years (11/277, 4%) in seronegative controls, with an absolute incidence difference associated with *T. cruzi* -seropositivity of 9.2 (95% CI, 3.6–15.0) events/1000 person-years.^149^

•In the Bambui Cohort Study of Aging, the incidence of cardiomyopathy has been evaluated in infected community-dwelling elderly and non-infected subjects with no major ECG abnormalities at the baseline in 1997, followed up until 2008. In the group with ChD (n=245), the incidence of cardiomyopathy was 24.8 (95% CI, 19.5–30.1) events/1000 person-years and, in the control group (n=617), 15.5 (95% CI, 12.7–18.5) events/1000 person-years. The absolute incidence difference associated with ChD in the entire period from 1997 to 2008 was 9.25 (95% CI, 3.2–15.2).^150^

•In a systematic review and meta-analysis of 32 studies of patients with ChD (samples ranged from 9 to 3336 participants), the pooled estimated annual rate of cardiomyopathy was 4.6% among patients with acute Chagas infection and 1.9% among those with indeterminate chronic ChD.^151^

### Mortality

•In the GBD Study 2019, both the number of deaths due to ChD in Brazil and age-standardized mortality rate decreased from 1990 to 2019. More details about ChD data from the GBD Study 2019 are available in the 2021 Cardiovascular Statistics – Brazil.^4^

•Similarly, several population-based studies have shown a reduction in mortality due to ChD in Brazil in the last decades, as reviewed in the 2021 Cardiovascular Statistics - Brazil, and they all have reported mortality rate decreases with regional variations.^4^

•A recent population-based ecological study in Northeastern Brazil, using data from 2016 to 2018, has evaluated 801 patients with chronic ChD in the Pernambuco state mesoregions. The average chronic disease occurrence rate was 3.2/100 000 person-year.^152^No cases of acute disease were reported. In total, 350 deaths were recorded, showing male predominance, age ≥ 60 years, and chronic disease with cardiac involvement as the main mortality cause. Similar patterns were previously reported, as discussed in the 2021 Cardiovascular Statistics - Brazil report.^4^The annual average mortality proportion was 1.6/100 000 people (standard deviation 2.4) ( **Chart 4-1** ).

**Chart 4-1:** – Standardized mortality rate due to Chagas disease in Brazil according to the year of occurrence and age range (in years), from 2000 to 2010. Source: Nóbrega et al. ^177^

•In a nationwide study that analyzed all death certificates of individuals who died between 2000 and 2019 in Brazil, ChD was mentioned in 122 291 deaths (0.54% of death certificates), 94 788 (77.5%) as an underlying cause and 27 503 (22.5%) as an associated cause. The mean standardized mortality rate was 3.22/100 000 inhabitants/year, with an observed declining trend in the period.^153^The highest mortality rates were observed in males, age group ≥80 years, black race/skin color, 1-3 years of schooling, and residents in the West-Central region, as shown in **Chart 4-2** .

**Chart 4-2:** – Spatial distribution of Chagas disease-related mortality rates (per 100 000 inhabitants) according to the Federative Unit in Brazil, 2000–2019. (A) Crude rates; (B) Age-adjusted rates. Source: Martins-Melo et al. ^153^

•In the above mentioned Bambui Cohort Study of Aging,^150^cardiomyopathy development increases the risk of death as compared to maintenance of a normal ECG [HR: 1.93 (95% CI, 1.02–3.65)]. The study concludes that ChD is associated with a higher risk of progression to cardiomyopathy in the elderly, which is associated with a higher risk of death in individuals with ChD.

•As discussed in the 2021 Cardiovascular Statistics -Brazil, previous studies have reported an increased risk of death of blood donors who were seropositive for ChD,^154^in addition to ChD as an underreported cause of death. In patients with HF, both all-cause and HF mortalities were significantly higher in patients with ChD as compared to those without ChD,^155^with an increased risk of death in patients with cardiomyopathy, higher in those with left ventricular aneurysms.^156^Another cohort has shown absolute death rates decreased over time in patients with ChCM and non-Chagas cardiomyopathies, but increases in the HR associated with ChD were observed over time.^157^More cardiovascular events have been reported in municipalities with fewer physicians per 1000 inhabitants or lower Primary Health Care coverage.^158^

•In a cohort study including 1551 patients with ChCM from Minas Gerais State, Brazil, a score to predict 2-year mortality was developed. The score included simple variables, such as age, New York Heart Association functional class, heart rate, QRS duration, and abnormal NT-proBNP adjusted by age. The observed mortality rates in the low, intermediate, and high-risk groups were 0%, 3.6%, and 32.7%, respectively, in the derivation cohort and 3.2%, 8.7%, and 19.1%, respectively, in the validation cohort, with C statistics of 0.82 and 0.71, respectively. It seems to be a helpful and simple score that could be used in remote areas with limited technological resources.^159^

•A more recent systematic review of mortality in ChCM included 52 longitudinal studies (9569 patients and 2250 deaths).^160^The annual all-cause mortality rate was 7.9% [95% CI, 6.3–10.1]. The pooled estimated annual cardiovascular death rate was 6.3% (95% CI, 4.9–8.0). Meta-regression showed that low left ventricular ejection fraction (coefficient = -0.04; 95% CI, -0.07;-0.02; P = 0.001) was associated with an increased mortality risk, and mortality was higher in more advanced HF stages, showing that mortality risk in chronic ChCM is substantial and primarily attributable to cardiovascular causes.

### Burden of Disease

•As reported in the 2021 Cardiovascular Statistics – Brazil,^4^in the GBD Study 2019, 174 194 DALYs (95% UI, 109 039-302 974) due to ChD were estimated in Brazil, with a relative reduction of 32.1% as compared to those of 1990. Age-standardized DALY rates declined at the national level (-70.5%) and in all Brazilian FUs between 1990 and 2019, but with different regional patterns. The decrease in the DALY rates was driven primarily by a consistent reduction in the YLL rates, the main component of total DALYs for ChD. The highest fatal and non-fatal burden due to ChD was observed among males, the elderly, and in the Brazilian FUs encompassing important endemic areas of vectorial transmission in the past, such as Goiás, Tocantins, Minas Gerais, Bahia, and Distrito Federal.

### Heart Failure

•Because HF is not considered an underlying cause of death (i.e., garbage code) in the GBD study, all deaths attributed to HF in death certificates are reclassified and/or redistributed to other causes, according to the GBD method. As such, there is no data from GBD on mortality from HF. Because HF is classified by GBD as an “impairment”, the only indicators we have for HF from the GBD are prevalence and YLDs, which is the morbidity component of DALYs.

### Prevalence and Incidence

•The latest available full GBD data are from 2017, as discussed in the 2021 Cardiovascular Statistics – Brazil.^4^There was a decrease of 5% (95 UI, -7.1;-3) in HF age-standardized prevalence, in Brazil, from 818 (95% UI, 718-923) in 1990 to 772 (95% UI, 680-875) in 2017. In absolute numbers, estimates for the prevalence of HF in Brazil rose from 0.67 million in 1990 to almost 1.7 million in 2017, mainly due to population growth and aging. The highest rates were observed in Rio Grande do Norte state, and the lowest in Acre. The HF prevalence was higher in women (795; 95% UI, 694-901) than in men (751; 95% UI, 656-845) in 2017, and the reduction in prevalence from 1990 to 2017 was more pronounced in men [the percentage of decrease was 7.5 (95% UI, -10.2;-4.8) for men and 3.2 (95% UI, -6.5;-0.1) for women]. Regarding age groups, there is a 10-fold increase in the incidence rates from the ‘15-49 years’ group to the ‘50-69 years’ group, as well as a 6-fold increase from the latter to the ‘70+ years’ group, and these increases are similar for women and men. From 1990 to 2017, the prevalence increased only in the ‘15-49 years’ group, while decreased in the others, probably associated with increased ischemic events in that age group.

•A systematic review has evaluated the burden of HF in Latin America and included 143 articles published between January 1994 and June 2014, with at least 50 participants aged ≥ 18 years; most studies included (64%) were from Brazil.^161^The patients’ mean age was 60±9 years, and the mean ejection fraction was 36±9%. The prevalence of HF was 1% (95% CI, 0.1-2.7). Of the studies included, only one assessed incidence, with 1091 individuals identified through multistage probability sampling in the city of Porto Alegre, Brazil. The mean age was 42.8±16.9 years, and 55% were women. The incidence of HF in the single population study providing this information was 199 cases per 100 000 person-years.^162^

•In a population-based study in primary care of a medium-sized city in Brazil, 633 individuals aged ≥45 years were randomly selected and registered in the primary care program. The mean age was 59.6±10.4 years, and 62% were females; the prevalence of symptomatic HF (stage C) was 9.3%, and the prevalence of stage B HF (structural abnormalities) was 42.7%. Of the patients with HF, 59% presented with HF with preserved ejection fraction and 41%, with HF with reduced ejection fraction.^163^

•A study from the Brazilian National Health Survey held in 2013, with data on 59 655 adults (≥ 18 years), found a prevalence of self-rated HF of 1.1%, which would represent about 1.7 million people. In those aged over 60 years, the prevalence was 3.3%.^164^

### Mortality

•An ecological study developed with secondary data on HF mortality in Brazil from 1996 to 2017 has reported 1 242 014 deaths from HF, as shown in **Chart 4-3** .^165^The temporal trend analysis results showed a significant decrease of 2.3% (95% CI, -2.3;-2.7) in the mortality rate across all Brazilian FUs. Northern municipalities maintained low mortality rates during the analyzed period, suggesting underreporting.

**Chart 4-3:** – Relative risk for heart failure mortality of all Brazilian municipalities in the periods: A) 1996-2001; B) 2002-2007; C) 2008-2012; and D) 2013-2017. Source: Cestari et al. ^165^

•Another ecological study that analyzed mortality from HF in Brazil for individuals aged 50 years or older, between 1998 and 2019, has found similar results.^166^The average rate was 75.5 deaths per 100 000 inhabitants, and women accounted for the highest proportion (n=299 093; 52.67%). The trend of mortality from HF in Brazilians older than 50 years was downward over 21 years, and this trend was also observed in both sexes and in 23 FUs ( **Chart 4-4** ). That study showed that the mortality rates from HF increased with advancing age in all Brazilian regions, with a higher occurrence in individuals aged 80 years or older (n=257 277; 45.31%).^166 , 167^

**Chart 4-4:** – Spatial distribution of moving averages of mortality rates due to heart failure among Brazilians over 50 years of age, Brazil, 1998 to 2019. Source: Arruda et al. ^166^

•A study was conducted on all death certificates related to deaths in Brazil between 2006 and 2016 to identify HF as the underlying cause of death or as a multiple cause of death. It included 1 074 038 death certificates issued between 2006 and 2016 with the code I50 in their Parts I or II. The frequency HF appeared as the multiple cause of death in both sexes was nearly three times higher than that of HF as an underlying cause of death. Specific ICD codes more frequently listed as the underlying cause of death in death certificates with HF present anywhere were hypertensive heart disease (11.3%), acute myocardial infarction (8.4%), diabetes mellitus (3.1%), dilated cardiomyopathy (2.9%), chronic obstructive pulmonary disease (2.6%), and ChD (1.7%).^168^

•As reported in the 2021 Cardiovascular Statistics – Brazil,^4^the percentage of death due to HF varies from 3% to 9% depending on the region, age, and performed analysis. In addition, mortality during the follow-up of patients with HF varies in Brazilian cohort studies, with 1-year mortality ranging from 6.8% to 20% depending on clinical factors and with survival improving over time.

### Hospitalizations

•Hospital admissions are the main consequences of decompensated HF, resulting in worse prognosis and increasing costs. The BREATHE Study evaluated a sample of patients admitted due to acute decompensated HF. A total of 1263 patients were included from 51 centers from different Brazilian regions in 2011 and 2012. In-hospital mortality was 12.6%, and care quality indicators based on hospital discharge recommendations were reached in less than 65% of the patients.^167^As reported in the 2021 Cardiovascular Statistics -Brazil,^4^and in recent evaluation of hospital admissions for HF, similar rates of in-hospital mortality have been reported, ranging from 9% to 17%.^115^

•Several temporal analyses have shown a decrease in both number of hospitalizations and in-hospital mortality over time, mostly in the last decade,^168 , 169^as discussed in detail in the 2021 Cardiovascular Statistics - Brazil.^4^

### Burden of Disease

•As reported in the 2021 Cardiovascular Statistics - Brazil,^4^the age-standardized YLD rates due to HF were 112 (95% UI, 83-141) in 1990 and 109 (95% UI, 81-134) in 2017, per 100 000 inhabitants, corresponding to a decrease of 3% (95% UI, -6.7 to 0.3). These changes are similar to those observed in the HF prevalence rates. Despite this decrease in YLD rates, HF resulted in 88 114 (95% UI, 64 078-112 624) DALYs in Brazil in 1990 and in 234 169 (95% UI, 174 338-291 188) in 2017, due to population growth and aging. The reductions were more pronounced in men during that period. As expected, the highest YLD rate was observed in the ‘70+ years’ group, followed by the ‘50-69 years’ group. Similar to the changes observed in prevalence, from 1990 to 2017, the greatest increases in YLD were observed in the ‘15-49 years’ group.

### Impact of the COVID-19 Pandemic

•Hospitalization and mortality data for Brazil between 2020 and 2021 have shown a reduction in hospitalization due to cardiovascular disease, with the highest absolute reduction in hospitalizations seen in HF and myocardiopathies (-66 499; 95% CI, -58 863.4; -74 035.9). There was also a relative reduction in hospitalizations in the period (RRi 0.761; 95% CI, 0.759-0.763). However, there was an increase in the clinical severity of hospitalized patients, with proportional increase of 19.4% (RRi 1194; 95% CI, 1188-1200) in intensive care unit admissions and 13.6% (RRi 1136; 95% CI, 1130-1143) in in-hospital deaths. These data suggest that less severe patients have probably not been hospitalized and, on the other hand, the more severe ones have probably sought the hospital later, worsening their outcomes.^15^

•The greatest reduction in hospitalization occurred in municipalities in the North region, in municipalities with lower human development index and in smaller municipalities. In addition, the reduction was greater in the elderly and women, reflecting the greater impact of the pandemic on clinical admissions of the most vulnerable groups. Regarding mortality from HF and cardiomyopathies, there has been a reduction in the standardized mortality rate and an increase in deaths at home.^15^

•Another study with data from January 2011 to June 2022 has shown a decrease in hospitalizations for HF since the beginning of the series, but with a most significant drop after the beginning of the COVID-19 pandemic. Data from 2020 to June 2022 show average monthly hospitalization of 8547 cases, which represents a 16.10% drop in relation to 2019 data and a 37.75% drop in relation to 2011 data. This represents a decrease from 132.81 hospitalizations per 100 000 inhabitants in 2011 to 78.58 hospitalizations per 100 000 inhabitants in 2021 (40.83% reduction, as reported in **Chart 4-5** ). On the other hand, there has been an increase in the lethality of HF, which increased from 10.00% in 2011 to 12.63% in 2019 and to 13.47% in 2022 ( **Chart 4-6** ).^170^

**Chart 4-5:** – Historical series of hospitalizations for HF from January 2011 to June 2022. Source: Cruz et al. ^170^

**Chart 4-6:** – Historical series of heart failure lethality from 2011 to 2022. Source: Cruz et al. ^170^

•A cohort study using data from the Best Practice in Cardiology program has compared the hospitalizations for HF during the COVID-19 outbreak from March 12^th^to October 31^st^, 2020, with admissions to the same hospitals during the same epidemiological weeks in 2019 and 2018. In that study, a 20% reduction in the number of hospitalizations was found. However, there was a significant increase in the mortality of hospitalized patients. After adjusting, the risk of death in 30 days was approximately two times higher in patients admitted during the COVID-19 outbreak (HR = 1.89 [95% CI, 1.19-3.03]; p=0.007) as compared to that of the two previous years. Poor compliance to treatment recommendations and acute kidney disease were more commonly reported as factors triggering HF decompensation during the COVID-19 outbreak. In addition, patients were admitted to the hospital with greater severity (measured through the ADHERE risk score) during the COVID-19 outbreak.^171^

•There has been concern about the impact of the COVID-19 and ChD coinfection, because of both the different forms of cardiac involvement or complications that COVID-19 can cause and the impact that acute COVID-19 and the immunosuppression induced by the disease and its treatment could have on ChD.^172^However, a multicenter study comparing hospitalized patients with COVID-19 and ChD with those without ChD has found no difference in mortality, length of stay or complications, such as acute HF, between the two groups.^173^A study using mortality rate data for 2020 obtained from the Brazilian Mortality Information System database has found 492 deaths related to the ChD and COVID-19 coinfection. The highest mortality rates were found for older age groups, individuals of Afro-Brazilian ethnicity, and those with low educational levels.^174^

### Health Care Utilization and Cost

•According to data from the SUS, there were 3 454 570 hospitalizations for HF from 2008 to 2021. This number represents more than one-third of the total clinical admissions related to cardiovascular conditions in the observed period. Adjusted total costs were R$ 8 438 025 775.

•During the observed period, there was a reduction in the number of clinical admissions for HF from 298 474 (157 per 100 000) in 2008 to 181 441 (85 per 100 000) in 2021, with an even reduction over the years. Despite that reduction in the number of admissions, unadjusted healthcare expenditure estimates from the adjusted direct payment for the care of patients with HF increased from 2008 to 2019 by almost 24%, from R$ 288 345 221 in 2008 to R$ 356 801 947 in 2021. A decreased number of admissions and increased expenditure represent higher costs *per* admission throughout the observed period (R$ 912 in 2008 to R$ 1787 in 2021). Heart failure was the main responsible for the costs related to clinical admissions among cardiovascular diseases.

•An ecological study using data from 2018 to 2021 has found projections that indicate a total expenditure of more than R$ 1 billion with hospitalizations, with an average hospital cost of R$ 1725.27 per person. The average cost per hospitalization exceeded R$ 2 billion. Approximately 3% of federal expenditures are earmarked for HF benefit payments. Of the total number of absences, 65% corresponded to men and 35% to women, with costs that could reach R$ 6 billion lost per year.^175 , 176^

•As discussed in the 2021 Cardiovascular Statistics - Brazil,^4^the economic burden of HF in Brazil imposes a financial cost of R$ 22.1 billion (US$ 6.8 billion), as assessed in 2015, the second out of four main heart conditions in Brazil: myocardial infarction, HF, hypertension, and atrial fibrillation,^132^and increases in the mean per-patient cost of HF-related hospitalizations have been reported.

### Future Directions

•Because HF is considered a garbage code when assigned as the underlying cause of death, studies investigating the better method to reclassify and redistribute this cause are needed to reduce bias and promote better data comparability to enhance health policies.

•Brazilian cohort studies for cardiomyopathies are scarce and some clinical studies in Brazil have reported HF data, but there are few multicenter studies with the Brazilian population data. It is important to have data for both HF and cardiomyopathy of both outpatients and hospitalizations, and to fully understand the increasing burden of HF on cardiovascular diseases. More multicenter large-scale studies are needed to better describe the burden, outcomes, and costs of HF in the Brazilian population.

•In addition, studies exploring quality of care and costs in HF would help develop health policies to improve awareness, access to life-saving interventions, organ donation, and the better use of resources in this complex and demanding disease.

•More studies on the impact of the COVID-19 pandemic, as well as on the post-pandemic era, are still needed.

## CHAPTER 5 – VALVULAR HEART DISEASE

### ICD-9 424; ICD-10 I34 to I38

**See Tables 5-1 and 5-2 and Charts 5-1 through 5-7**

**Table:** Abbreviations Used in Chapter 5

CI	Confidence Interval
DALY	Disability-Adjusted Life Years
ICD-10	International Statistical Classification of Diseases and Related Health Problems, 10^th^Revision
IE	Infective Endocarditis
GBD	Global Burden of Disease
HIC	High-Income Countries
LMIC	Low to Middle-Income Countries
NRVD	Non-Rheumatic Valvular Disease
RHD	Rheumatic Heart Disease
SUS	Brazilian Unified Health System (in Portuguese, *Sistema Único de Saúde* )
PBMVC	Percutaneous Balloon Mitral Valve Commissurotomy
VHD	Valvular heart disease
TAVI	Transcatheter Aortic Valve Implantation
UI	Uncertainty Interval

### Prevalence

#### Rheumatic Heart Disease

•RHD is a preventable condition. Prompt and appropriate treatment of streptococcal infections with antibiotics may prevent the development of RHD. However, in many LMIC, access to healthcare and antibiotics can be limited, leading to higher rates of RHD. According to the *Global Atlas on Cardiovascular Disease Prevention and Control* , it is estimated that RHD currently affects 33.0-40.5 million people worldwide, accounting for 1% to 1.5% (306 000 deaths) of all cardiovascular deaths.^36 , 178^Until the middle of the 20^th^century, RHD was the leading cause of VHD in the world. Improved health conditions, early identification of *Streptococcus pyogenes* infections and antibiotic use decreased significantly RHD prevalence in HIC.

•RHD is the primary cause of 2.5% of VHDs in the United States and Canada, reaching up to 22% in Europe,^179^and even higher rates have been reported in Brazil, reaching around 50% of the heart valve surgeries in SUS.^180 - 182^

•In LMIC the prevalence of RHD remains around 444 per 100 000 inhabitants.^36 , 183^In Brazil, it persists as the main etiology of VHDs, especially in patients from the SUS. Previous evaluations have shown a prevalence of 360 per 100 000 in our country.^184^Other evaluations have found a prevalence ranging from 100 to 700 per 100 000 schoolchildren.^185^More recently (2022), a study from the Brazilian Amazon Basin has found a prevalence of 39 per 1000, indicating the need for screening programs in both remote and known high-prevalence areas.^186^Simplified echocardiographic criteria may increase the screening in suspected high-prevalence areas.^187^

•According to more recent data from *Global Burden of Cardiovascular Diseases and Risks Collaboration* , from 1990 to 2021,^186^the age-standardized prevalence of RHD in Tropical Latin America (which includes Brazil and Paraguay, with estimates mostly driven by Brazilian data) was 1266.6 (95% UI, 987.3-1543.5) per 100 000, resulting in approximately 3 047 102 prevalent cases ( **Table 5-1** ). These point rates are above those observed in GBD 2019 in Brazil [918.5 (95% UI, 716-1142.5) per 100 000] and Paraguay, suggesting that the RHD prevalence may still be growing in the region. Nonetheless, it may also mean that diagnosis, detection, and health statistics are improving.^186^

**Table 5-1:** – Regional cardiovascular disease (infective endocarditis, rheumatic and non-rheumatic valve diseases) in 2021 in Tropical South America (Brazil and Paraguay): counts and age-standardized rates per 100 000

Cardiovascular disease type	Prevalent cases (count)	Deaths (count)	Prevalence (rate/100 000)	Deaths (rate/100 000)	DALYs (rate/100 000)
Rheumatic heart disease	3 047 102	2823	1266.6	1.1	93.5
Non-rheumatic calcific aortic valve disease	359 861	4059	140.6	1.6	32.2
Non-rheumatic degenerative mitral valve disease	112 951	1761	44.0	0.7	17.2
Other non-rheumatic valve diseases	474	75	0.2	<0.1	0.8
Infective endocarditis	15 053	2775	6.7	1.1	34.6

Source: Adapted from Lindstrom et al. ^186^

•RHD is still the most prevalent cause of mitral valve disease in Brazil when both mitral stenosis (over 90%) and regurgitation (around 55-60%) are considered.^187^

•Mitral stenosis occurs more frequently in women than in men, at a ratio of 3:2. It is a frequent sequela of acute rheumatic fever, reaching more than 85% of cases even in HIC, such as those in Europe,^188^with a similar pattern observed in Brazil.^182 , 187^

#### Non-Rheumatic Valvular Heart Disease

•According to a recent GBD 2021 update,^36 , 37 , 186^the age-standardized prevalence of non-rheumatic degenerative mitral valve disease in Tropical Latin America in 2021 was 44.0 (95% UI, 41.5-47.0) per 100 000, resulting in 112 951 prevalent cases ( **Table 5-1** ),^186^depicting a continuous increase if compared to the 1990-2019 trends for total NRVDs, which increased from 25.3 (95% UI, 22.4-27.8) per 100 000 in 1990 to 39 (95% UI, 33.9-44.6) per 100 000 in 2019.^36^This past trend, however, was mostly driven by calcific aortic valve disease (201.8%), as mitral degenerative valve disease showed a stable trend, with slight percent changes: -2.3% (95% UI, -4 to -0.4).^34 , 36^

•Non-rheumatic calcific aortic valve disease had an age-standardized prevalence rate of 140.6 (95% UI, 112.9-169.7) per 100 000 in Tropical Latin America, with an average of 359 861 prevalent cases, representing a trend towards increasing prevalence, especially in older ages, as previously observed in Brazil (1990-2019) ( **Table 5-1** ).^36 , 186 , 189^

•Other NRVDs, with considerable drawbacks related to the quality of primary data and case definition, had an age-standardized prevalence of 0.20 (95% CI, 0.16-0.24) per 100 000 individuals in Tropical Latin America.^186^

•Prevalence estimates in GBD 2021 for RHD and NRVD are available neither at the subnational level nor for Brazil separately, precluding deeper inferences about their association with healthcare access and quality or with markers of socioeconomic development.^186^

•Unlike mitral valve disease, aortic valve disease is predominantly degenerative or calcific. Observational studies have shown aortic stenosis in 4.5% of the population aged >75 years in HIC, such as the United States.^190^According to observational studies^187 , 191^and the GBD 2021 data,^186^in Brazil, as well as in the rest of the world, an increasing trend towards degenerative aortic valve disease has been observed in areas where the epidemiological transition and changes in age composition have evolved, as compared to other etiologies, such as RHD.^192^

## Mortality

•Valvular heart disease is one of the leading causes of cardiovascular death in Brazil, particularly in economically underserved regions, RHD being the most socially driven etiology.

### Rheumatic Heart Disease

•Although nationwide data are still scarce, age-standardized mortality rates attributable to RHD continue to decrease. The last GBD 2021 analysis observed a rate of 1.0 (95% UI, 1.0-1.2) per 100 000 deaths in the Tropical Latin America area, which includes Brazil and Paraguay,^186^compared to 1.2 (95% UI, 1.1-1.2) per 100 000, according to the GBD 2019 study, and to 2.8 (95% UI, 2.7-3.0) 20 years before, according to the most recent GBD temporal series.^36^However, for crude mortality rates ( **Table 5-1** ), there was a slight increase. The total number of deaths in 2019 was 2715 (95% UI, 2505-2913), compared to 2823 in 2021, possibly because of population growth ( **Table 5-1** ), although such trends must be carefully evaluated, considering that, for 2021, only point estimates – as opposed to temporal series – are available to date. The age-standardized mortality rate due to RHD in Brazil was 1.1 (95% UI, 1.0-1.2) per 100 000,^186^superimposable to that observed in the point estimates of the GBD 2019, 1.2 (95% UI, 1.1-1.2) per 100 000. In 2023, Paraná, Minas Gerais, Bahia, Goiás, Sergipe, and Pernambuco were the Federative Units with the RHD age-standardized mortality rates in the highest quartile (ranging from 1.2 to 1.5 per 100 000), the highest rates being observed in Goiás and Paraná^37 , 186^( **Table 5-1 and Chart 5-1A** ).

**Chart 5-1A:** – Rheumatic heart disease: age-standardized per 100 000 mortality rate quartiles according to the Brazilian Federative Units. Data from the Global Burden of Disease 2021 Estimates. ^48^

•Again, for those estimates, the lack of systematized primary data may have led to inaccuracies, considering that Paraná has one of the highest sociodemographic indexes in Brazil. On the contrary, three states of the Northeastern region, the poorest in the country, are also included in that list.

### Non-Rheumatic Valvular Heart Disease

•According to the GBD 2021 study, the age-standardized mortality rates attributable to specific NRVDs in Tropical Latin America were: 0.7 (95% UI, 0.6-0.7) per 100 000 for non-rheumatic degenerative mitral valve disease, 1.6 (95% UI, 1.4-1.7) per 100 000 for non-rheumatic calcific aortic valve disease, and <0.1 [0.03 (95% UI, 0.026-0.034) per 100 000] for other NRVDs ( **Table 5-1** ).^186^The total numbers of estimated fatalities in 2021 were 1761, 4059, and 75 for non-rheumatic degenerative mitral valve disease, non-rheumatic calcific aortic valve disease, and other NRVDs, respectively ( **Table 5-1** ). These data reinforce the trends from recent estimates of higher mortality for non-rheumatic aortic valve disease, as seen in the GBD 2019 analysis.^4 , 36^This observation, in addition to the previous temporal trends, is also aligned with the demographic profile of the country, with population aging and the increasing impact of cardiovascular risk factors.^189^

•Rio Grande do Sul, Santa Catarina, Paraná, São Paulo, Espírito Santo, Distrito Federal, and Amapá were the Federative Units with age-standardized mortality rates due to non-rheumatic calcific aortic valve disease in the highest quartile (ranging from 1.65 to 2.42 per 100 000) ( **Table 5-2 and Chart 5-2A** ).^186^

**Table 5-2:** – Metrics related to valvular heart diseases (infective endocarditis, rheumatic and non-rheumatic valvular diseases)

Location	DALYs per 100 000				Deaths per 100 000	
	Estimate	95% CI Lower	95% CI Upper	Estimate	95% CI Lower	95% CI Upper
**Infective endocarditis**						
Acre	47.58	44.62	52.12	1.44	1.35	1.57
Alagoas	39.68	35.65	43.64	1.20	1.07	1.32
Amapá	40.17	35.89	45.96	1.25	1.12	1.43
Amazonas	27.73	25.52	29.70	0.87	0.78	0.94
Bahia	36.24	31.65	40.22	1.03	0.89	1.15
Brazil *	34.42	32.74	36.17	1.11	1.03	1.18
Ceará	29.70	26.16	33.78	0.93	0.80	1.07
Distrito Federal	27.51	24.92	30.41	1.05	0.94	1.15
Espírito Santo	35.11	31.16	38.36	1.10	0.97	1.20
Goiás	36.46	32.77	40.25	1.21	1.10	1.34
Maranhão	27.52	23.78	30.90	0.82	0.72	0.93
Mato Grosso	34.07	30.33	37.53	1.07	0.95	1.16
Mato Grosso do Sul	38.25	34.19	41.61	1.23	1.11	1.34
Minas Gerais	40.02	36.43	43.26	1.30	1.16	1.41
Pará	32.72	28.59	37.08	0.99	0.84	1.12
Paraíba	29.60	26.64	32.13	0.93	0.84	1.01
Paraná	29.20	26.74	31.85	0.97	0.89	1.06
Pernambuco	27.78	24.90	30.46	0.88	0.78	0.98
Piaui	30.78	26.89	34.85	0.93	0.81	1.05
Rio de Janeiro	39.18	35.82	43.74	1.24	1.12	1.39
Rio Grande do Norte	30.24	26.88	33.56	0.91	0.81	1.01
Rio Grande do Sul	31.25	28.39	34.37	1.06	0.96	1.17
Rondônia	31.64	28.11	36.34	0.99	0.88	1.14
Roraima	30.30	25.93	35.05	0.98	0.84	1.14
Santa Catarina	33.69	30.04	36.88	1.16	1.03	1.28
São Paulo	34.97	31.42	38.19	1.20	1.07	1.33
Sergipe	34.86	31.84	38.21	1.09	0.99	1.19
Tocantins	32.47	28.27	37.01	1.02	0.89	1.16
**Non-rheumatic calcific aortic valve disease**						
Acre	25.58	23.44	28.45	1.28	1.14	1.40
Alagoas	27.35	24.63	30.74	1.31	1.16	1.48
Amapá	33.36	29.12	38.11	1.66	1.46	1.86
Amazonas	31.56	28.41	34.89	1.51	1.34	1.65
Bahia	27.59	24.20	30.69	1.26	1.10	1.40
Brazil *	31.78	29.43	34.01	1.63	1.44	1.73
Ceará	24.40	20.83	27.98	1.20	1.01	1.38
Distrito Federal	31.07	27.90	34.17	1.91	1.69	2.08
Espírito Santo	32.92	28.97	36.35	1.70	1.50	1.87
Goiás	33.39	30.52	36.86	1.64	1.44	1.80
Maranhão	24.89	21.43	28.84	1.20	1.06	1.37
Mato Grosso	29.35	26.49	32.44	1.43	1.26	1.57
Mato Grosso do Sul	32.11	29.01	35.47	1.60	1.42	1.79
Minas Gerais	30.41	27.23	33.45	1.51	1.31	1.66
Pará	27.56	24.18	32.02	1.32	1.09	1.54
Paraíba	20.74	18.71	22.92	0.99	0.88	1.10
Paraná	37.98	34.80	41.87	1.97	1.76	2.19
Pernambuco	29.92	27.05	33.17	1.50	1.30	1.68
Piaui	20.76	18.08	23.72	0.98	0.87	1.12
Rio de Janeiro	28.92	25.88	32.36	1.48	1.26	1.65
Rio Grande do Norte	25.42	22.54	28.36	1.24	1.07	1.36
Rio Grande do Sul	39.05	35.00	42.63	2.15	1.92	2.37
Rondônia	28.14	24.75	31.95	1.38	1.20	1.57
Roraima	31.14	26.92	36.69	1.64	1.39	1.88
Santa Catarina	43.17	38.26	47.68	2.42	2.09	2.65
São Paulo	35.93	32.37	40.13	1.94	1.69	2.18
Sergipe	19.87	17.77	21.78	0.96	0.85	1.05
Tocantins	28.34	24.92	32.19	1.40	1.22	1.60
**Non-rheumatic degenerative mitral valve disease**						
Acre	14.41	13.37	15.79	0.57	0.52	0.63
Alagoas	18.97	17.01	21.30	0.72	0.64	0.83
Amapá	18.71	16.30	20.87	0.75	0.65	0.83
Amazonas	13.18	11.76	14.67	0.52	0.46	0.58
Bahia	16.09	14.11	18.14	0.58	0.51	0.65
Brazil *	17.25	16.28	18.18	0.70	0.64	0.74
Ceará	16.71	14.17	19.17	0.67	0.57	0.77
Distrito Federal	15.56	14.05	17.24	0.75	0.68	0.84
Espírito Santo	21.74	19.27	24.21	0.89	0.78	1.00
Goiás	17.83	16.02	19.86	0.72	0.64	0.80
Maranhão	13.13	11.26	15.07	0.50	0.43	0.57
Mato Grosso	16.71	15.08	18.41	0.66	0.58	0.72
Mato Grosso do Sul	18.98	17.04	20.77	0.75	0.67	0.83
Minas Gerais	15.04	13.60	16.39	0.60	0.52	0.66
Pará	17.90	15.33	20.93	0.70	0.59	0.82
Paraíba	12.22	10.95	13.53	0.47	0.41	0.52
Paraná	19.80	17.75	21.84	0.83	0.74	0.92
Pernambuco	21.79	19.54	23.85	0.87	0.77	0.96
Piaui	14.16	12.40	16.08	0.54	0.47	0.61
Rio de Janeiro	15.24	13.65	17.04	0.60	0.53	0.67
Rio Grande do Norte	13.30	11.81	14.69	0.51	0.45	0.57
Rio Grande do Sul	17.66	16.09	19.36	0.76	0.66	0.83
Rondônia	14.39	12.58	16.53	0.58	0.50	0.66
Roraima	11.52	9.72	13.71	0.46	0.38	0.55
Santa Catarina	18.37	16.35	20.34	0.80	0.71	0.89
São Paulo	18.97	16.94	21.13	0.81	0.72	0.91
Sergipe	13.67	12.25	15.05	0.54	0.49	0.60
Tocantins	21.95	19.47	24.99	0.90	0.79	1.02
**Other non-rheumatic valve diseases**						
Acre	1.07	0.88	1.27	0.04	0.04	0.05
Alagoas	0.60	0.47	0.72	0.02	0.02	0.03
Amapá	0.99	0.80	1.22	0.04	0.03	0.05
Amazonas	0.70	0.57	0.85	0.03	0.02	0.03
Bahia	0.67	0.58	0.81	0.02	0.02	0.03
Brazil *	0.79	0.71	0.88	0.03	0.03	0.03
Ceará	1.19	0.98	1.45	0.05	0.04	0.06
Distrito Federal	0.71	0.59	0.86	0.03	0.03	0.04
Espírito Santo	0.98	0.80	1.16	0.04	0.03	0.05
Goiás	0.87	0.74	1.06	0.03	0.03	0.04
Maranhão	1.14	0.96	1.35	0.04	0.04	0.05
Mato Grosso	0.66	0.55	0.84	0.03	0.02	0.03
Mato Grosso do Sul	0.32	0.26	0.38	0.01	0.01	0.02
Minas Gerais	0.69	0.57	0.85	0.03	0.02	0.03
Pará	1.81	1.45	2.18	0.07	0.05	0.09
Paraíba	0.90	0.76	1.05	0.03	0.03	0.04
Paraná	1.06	0.87	1.29	0.04	0.04	0.05
Pernambuco	0.84	0.69	1.01	0.03	0.03	0.04
Piaui	0.77	0.62	0.96	0.03	0.02	0.04
Rio de Janeiro	0.44	0.37	0.53	0.02	0.01	0.02
Rio Grande do Norte	1.14	0.95	1.35	0.04	0.04	0.05
Rio Grande do Sul	0.90	0.77	1.08	0.04	0.03	0.04
Rondônia	0.92	0.75	1.09	0.04	0.03	0.04
Roraima	1.89	1.55	2.25	0.07	0.06	0.09
Santa Catarina	0.50	0.40	0.59	0.02	0.02	0.03
São Paulo	0.60	0.51	0.76	0.02	0.02	0.03
Sergipe	0.65	0.55	0.77	0.03	0.02	0.03
Tocantins	1.56	1.32	1.81	0.06	0.05	0.07
**Rheumatic heart disease**						
Acre	93.71	72.25	120.60	1.13	1.02	1.25
Alagoas	98.41	75.74	123.81	1.18	0.99	1.37
Amapá	83.57	61.54	110.32	0.81	0.71	0.92
Amazonas	79.17	57.75	106.01	0.71	0.54	0.80
Bahia	102.46	77.61	134.08	1.22	1.06	1.41
Brazil*	93.66	70.92	122.70	1.11	0.97	1.20
Ceará	85.65	62.72	115.53	0.82	0.71	0.95
Distrito Federal	97.24	74.63	129.24	1.50	1.22	1.71
Espírito Santo	96.43	74.58	125.54	1.23	1.01	1.42
Goiás	102.86	80.71	133.75	1.45	1.19	1.65
Maranhão	92.06	69.06	120.67	1.02	0.85	1.16
Mato Grosso	87.12	64.35	116.87	0.95	0.75	1.09
Mato Grosso do Sul	89.44	66.95	120.27	0.95	0.80	1.08
Minas Gerais	98.87	74.13	128.93	1.33	1.16	1.46
Pará	86.80	63.37	115.89	0.89	0.77	1.03
Paraíba	92.53	69.89	119.40	1.02	0.86	1.16
Paraná	99.95	76.58	127.95	1.36	1.21	1.56
Pernambuco	103.62	80.45	131.52	1.32	1.08	1.54
Piaui	84.69	60.53	112.98	0.76	0.65	0.86
Rio de Janeiro	91.72	68.77	121.04	1.03	0.83	1.20
Rio Grande do Norte	94.03	71.73	123.51	1.06	0.90	1.19
Rio Grande do Sul	84.38	61.76	112.28	0.83	0.73	0.92
Rondônia	88.51	66.25	117.17	1.00	0.86	1.15
Roraima	85.87	65.88	110.36	0.94	0.80	1.11
Santa Catarina	91.62	69.41	123.48	1.10	0.95	1.24
São Paulo	90.94	67.73	122.63	1.07	0.89	1.22
Sergipe	98.83	74.55	135.42	1.19	0.98	1.34
Tocantins	93.75	71.02	123.10	1.15	0.99	1.32

* Estimates for Brazil. Source: Data derived from Global Burden of Disease Collaborative Network. Global Burden of Disease (GBD) Cardiovascular Burden Estimates 1990 and 2021, Institute for Health Metrics and Evaluation, University of Washington. ^48^

**Chart 5-2A:** Data from the Global Burden of Disease 2021 Estimates.^*48*^

•Regarding non-rheumatic degenerative mitral valve disease, the age-standardized mortality rates observed were higher in Rio Grande do Sul, Santa Catarina, Paraná, São Paulo, Espírito Santo, Tocantins, and Pernambuco (ranging from 0.754 to 0.896 per 100 000) ( **Table 5-2 and Chart 5-3A** ).^186^

**Chart 5-3A:** Data from the Global Burden of Disease 2021 Estimates.^*48*^

•Concerning other NRVDs, Roraima, Pará, Maranhão, Tocantins, Ceará, Acre, and Paraná were the Federative Units with the highest estimated age-standardized mortality rates (ranging from 0.04 to 0.07 per 100 000) ( **Table 5-2 and Chart 5-4A** ).^186^

**Chart 5-4A:** 

•The increasing mortality rates in older ages due to NRVDs contrast with the trends observed for RHD, possibly reflecting a higher prevalence and, consequently, mortality in the age groups >70 years, for both aortic and mitral diseases.^34 , 36 , 186^In addition to the need for specific attention from the public health perspective, these trends point towards a significant growth in the expenses related to degenerative valve diseases, especially related to new devices, such as TAVI and edge-to-edge mitral valve repair, and continuing care for the elderly living with VHD.^189 , 191^

## Burden of Disease

### Rheumatic Heart Disease

•According to GBD 2021 data, the age-standardized rate of DALYs attributable to RHD significantly increased to 93.7 (95% UI, 70.9-122.7) per 100 000 in Brazil ( **Table 5-2** ), counteracting the downward trend observed from 1990 to 2019, 144.6 (95% UI, 126.8-167.3) and 79.3 (95% UI, 61.6-102.6) per 100 000, respectively.^37 , 186^Again, these trends require a careful interpretation, in the absence of a temporal series until the point estimates of 2021, and also considering the recent changes in the RHD modeling, especially with the incorporation of additional inputs, such as the prevalence of latent disease, which may considerably impact estimates in places where screening programs are held.^180 , 181^The Federative Units of Minas Gerais, Goiás, Paraná, Bahia, Sergipe, Alagoas, and Pernambuco had the highest age-standardized DALYs rates in the country, which were positioned in the upper quartile (range from 97.8 to 104.0 per 100 000) ( **Chart 5-1B** ).^186^

**Chart 5-1B:** – Rheumatic heart disease: age-standardized per 100 000 DALY rate quartiles according to the Brazilian Federative Units. Data from the Global Burden of Disease 2021 Estimates. ^48^

### Non-Rheumatic Valvular Heart Disease

•According to the GBD, the age-standardized rate of DALYs attributable to non-rheumatic calcific aortic valve disease in 2021 was 31.8 (95% UI, 29.4-34.0) per 100 000, considerably higher than that observed for non-rheumatic degenerative mitral valve disease, 17.2 (95% UI, 16.3-18.2) per 100 000, and especially for other NRVDs, which yield a much lower burden, 0.8 (95% UI, 0.7-0.9) per 100 000.^186^However, the overall age-standardized DALY rates associated with NRVDs continue to decrease, showing a continuing downward trend when compared to the last published time series: 62.8 (95% UI, 60.3-65.2) per 100 000 in 1990 and 44 (95% UI, 40.7-47) per 100 000 in 2019 ( **Table 5-2** ).^4 , 36^

•Rio Grande do Sul, Santa Catarina, Paraná, São Paulo, Espírito Santo, Goiás, and Amapá were the Federative Units with non-rheumatic calcific aortic valve disease age-standardized DALY rates per 100 000 within the highest quartile (range 32.5 to 43.2 per 100 000) ( **Table 5-2 and Chart 5-2B** ). Of note, 6 out of these 7 Federative Units are in the highest Brazilian sociodemographic strata, which may at least partially reflect the trends in age composition – ultimately population aging – and, consequently, the burden of risk factors.^189 , 191 , 193^

**Chart 5-2B:** 

•Regarding non-rheumatic degenerative mitral valve disease, the highest age-standardized DALY rates were observed in the Federative Units of Paraná, São Paulo, Mato Grosso do Sul, Espírito Santo, Tocantins, and Pernambuco (ranging from 18.8 to 22.0 per 100 000) ( **Table 5-2 and Chart 5-3B** ),^186^in a similar trend compared to non-rheumatic aortic valve disease, except for the Northeastern state of Pernambuco, which contrasts with those other Federative Units in terms of sociodemographic status and optimal healthcare access and utilization.

**Chart 5-3B:** Data from the Global Burden of Disease 2021 Estimates.^*48*^

•Regarding the other NRVDs, Roraima, Pará, Maranhão, Tocantins, Ceará, and Rio Grande do Norte were the Federative Units with the age-standardized DALY rates in the highest quartile (ranging from 1.06 to 1.89 per 100 000) ( **Table 5-2 and Chart 5-4B** ).^37 , 186^

**Chart 5-4B:** Data from the Global Burden of Disease 2021 Estimates.^*48*^

## Associated Diseases

### Endocarditis

•The age-standardized mortality rate associated with IE in Tropical Latin America was 1.1 (95% CI, 1.0-1.2) per 100 000 in 2021, resulting in approximately 2775 fatalities. According to the GBD 2021, IE had the largest percent increase in cardiovascular disease cause-specific age-standardized mortality rates since 1990 (an estimated increase of 35.4%). The mortality rate, as well as the temporal trends, was the same for Brazil ( **Table 5-1** ).^186^

•The estimated total number of prevalent cases of IE in the region was 15 053 in 2021, resulting in a prevalence rate of 6.7 (95% UI, 5.6-7.7) per 100 000. Rio de Janeiro, Minas Gerais, Goiás, Mato Grosso do Sul, Amapá, and Acre were the Federative Units with the IE age-standardized mortality rates in the highest quartile (range from 1.2 to 1.4 per 100 000) ( **Table 5-2 and Chart 5-5A** ),^186^a slightly different pattern when compared to RHD estimates – one known cause of endocarditis in the young.^194^

**Chart 5-5A:** 

•The age-standardized DALY rate attributable to IE in Brazil was 34.4 (95% UI, 32.7-36.2) per 100 000 in 2021. The same Federative Units included in the highest IE mortality group, in addition to the Northeastern state of Alagoas, had the rates of DALYs in the highest quartile in the country (range 36.4 to 47.6 per 100 000) ( **Table 5-2 and Chart 5-5B** ).^186^

**Chart 5-5B:** 

•A recent study in Brazil has observed a 22.3% mortality rate in a group of 221 patients from Minas Gerais^195^hospitalized with IE, mainly due to *Staphylococcus sp* infection. A systematic review of IE following TAVI for non-rheumatic aortic stenosis has observed an estimated 29.5% in-hospital mortality and a 29.9% mortality at mid- and long-term follow-up. The incidence of IE following TAVI was 3.25%, but it was associated with higher mortality and life-threatening complications. However, in HIC, there is a trend towards reduced mortality in IE patients.^196^Such a trend has not yet been observed in Brazil, although robust primary estimates are still scarce.^189^

## Healthcare Utilization and Cost

•The number of clinical hospitalizations associated with VHDs reduced after the COVID-19 pandemic, from 2289 (1.09 per 100 000) in 2019 to 1536 (0.72 per 100 000) in 2020. A slight recovery was observed in 2021: 1597 clinical hospitalizations (0.75 per 100 000).^49^

•According to the SUS administrative database, total crude expenses (reimbursement) with hospital admissions for clinical treatment of VHD in Brazil decreased from R$ 1 999 540 ($397 355) in 2019 to R$ 1 526 256 ($303 913) in 2020, with a partial recovery to R$ 1 465 800 ($307 346) in 2021. In comparison to a 3-year time series (2017-2019), the decrease in the pandemic years in US dollars was around 19.1% ( **Tables 1-1 through 1-7 and Charts 1-1 and 1-7** ).^49^

•In addition, the number of open VHD surgeries reduced significantly after the COVID-19 pandemic, ranging from 12 771 (6.08 per 100 000) in 2019 to 9198 (4.34 per 100 000) in 2020 and 8759 (4.10 per 100 000) in 2021.^49^

•Regarding other valvuloplasty procedures (not including PBMVC), the number of hospital admissions also initially reduced from 450 (0.21 per 100 000) in 2019 to 399 (0.19 per 100 000) in 2020 (-9.5%). In 2021, there was a return to previous values of 470 yearly hospitalizations (0.22 per 100 000), possibly reflecting a pent-up demand for procedures deferred during the pandemic.^49^

•A similar phenomenon was observed for the number of PBMVC, which reduced from 195 (0.09 per 100 000) in 2019 to 129 (0.06 per 100 000) in 2020 and to 159 (0.07 per 100 000) in 2021.^49^These numbers are especially remarkable for Brazil and require a deep consideration by health authorities, as the procedure is mainly performed at public teaching institutions, which demand a complementary budget, considering the current per-procedure reimbursement, far below the market prices of the required devices (especially the Inoue balloon system).

• These aforementioned patterns are certainly associated with restrictions for elective surgeries and procedures, and also with the need for more hospital rooms available for COVID-19 patients, with reorganization of health services. In the peak of the pandemic, the restrictions tended to be even stricter and associated with competition for hospital beds for severely ill COVID-19 patients. Since interventional percutaneous procedures result in shorter in-hospital stays, in 2021, there was a slight increase in these procedures compared to open heart valve surgeries ( **Tables 1-1 through 1-7 and Charts 1-1 and 1-7** ).^37 , 49 , 182^

•Therefore, total expenses with hospitalizations for open heart valve surgeries dropped from R$ 187 382 032 ($42 835 735) in 2019 to R$ 137 383 131 ($30 108 266) in 2020 (-29.8%) and maintained the downward trend in 2021 [R$ 131 110 929 ($27 491 081)] ( **Chart 5-6** ). In 2021, however, the impacts of the pandemic were still present in health services, especially in high-complexity settings. In addition to the outbreak of coronavirus variants, such as Omicron, the health services were still not fully recovered and adequately structured for resuming elective surgeries ( **Tables 1-1 through 1-7 and Charts 1-1 and 1-7** ).^49^

**Chart 5-6:** 

•On the other hand, public expenses associated with other valvuloplasty procedures initially dropped from R$ 1 959 571 ($447 960) in 2019 to R$ 1 851 963 ($397 978) in 2020 (-11.2%), but recovered to R$ 2 175 077 ($456 066 06), above the baseline values of the pre-pandemic time series, in 2021 ( **Chart 5-7** ).^49^

**Chart 5-7:** 

•The same happened to total crude expenses with hospital admissions for PBMVC, which reduced from R$ 1 430 166 ($326 937) in 2019 to R$ 923 776 ($202 450) in 2020, and then partially recovered to R$ 1 033 888 ($216 783) in 2021 ( **Chart 5-7** ).^49^In addition to resuming procedures deferred due to the pandemic, this reflects the increase in reimbursed values for devices which started in mid-2021. This is remarkably important for PBMVC, as several public and private hospitals are no longer performing these procedures, given the insufficient reimbursement. This further limits the access of patients – especially those with RHD – to optimal care, mainly in remote locations without university hospitals. A revision of the reimbursement tables for cardiovascular devices must be among the priority topics for policy-making in the 2020’s.

## Valvular Heart Disease and COVID-19 Pandemic

•Patients with advanced valve disease had an unfavorable impact during the COVID-19 epidemic. This was particularly observed for those with an indication for surgical or interventional treatment, for the elderly, and those with impaired physical conditions.^37^

•A multicenter study showed that patients with severe VHD and COVID-19 had poor short-term clinical outcomes, with 30-day overall mortality of 41.8%, much higher than that of other published series.^197^In that cohort, mean age was high (80.0 ± 9.7 years), and the most frequent types of VHD were aortic stenosis (54.4%) and severe mitral regurgitation (20.6%), different from the Brazilian reality, especially in the SUS clinics.^4 , 197^Most patients had preexisting symptoms related to their VHD, such as dyspnea (83.1%), chest pain (19.1%), and syncope (8.1%) before the COVID-19 infection. Upon admission, shortness of breath (64.9%), cough (57.5%), and fever (41.3%), among other severe respiratory manifestations, were symptoms related to COVID-19.^197^Lower mortality (11.1%) was observed in those patients under 80 years of age and with their VHD previously treated with surgical interventions. Conversely, the worst prognosis (mortality of 59.9%) was seen in those untreated patients with severe VHD and over 80 years of age,^197^what points towards the need for structured programs focused on preparing and managing elderly patients for valvular procedures and providing health education to providers related to RHD and NRVD. Notably, severe mitral regurgitation (54.4%) and severe aortic stenosis (42.6%) – the most prevalent diseases – had higher mortality than other VHDs (29.7%).^197^

•These compelling data showing the remarkable impact of COVID-19 on surgical VHD mortality compared to other historical series and administrative data urge specific preparation of health services for future outbreaks.^37 , 197^Even with the end of the pandemic, after almost 7 million deaths and more than 760 million confirmed cases, COVID-19 still needs attention due to its devastating direct and indirect effects on people’s health, including people with cardiovascular diseases.

## Future Directions

•As previously highlighted (2020/2021),^2 , 4^we reinforce the importance of improving data collection, research, and healthcare infrastructure related to VHD in Brazil. Following the pandemic, health surveys, registries and specific screening and early diagnosis programs must be resumed and reinforced. Research initiatives should focus on patterns of VHD with the highest impact on population health, such as RHD late sequelae and the growing burden of degenerative aortic valve disease.

•Given the lack of primary data regarding the epidemiology of VHD in Brazil, as well as the impact of the pandemic on ongoing research, gathering accurate and up-to-date data on the prevalence, incidence, and trends of VHD should be a focus for the public and private sectors. Up-to-date data are warranted for healthcare planning, resource allocation, and understanding the burden of the disease.

•Administrative data collection and specific coding for surgical and interventional procedures related to VHD are essential. These specific codes, avoiding the need for cross-linkage with ICD codes, can help healthcare professionals and policymakers better track the disease’s prevalence and characteristics, as well as more detailed insights on the economic burden. Discriminating variables, such as the type of valve involvement, valvular dysfunction, prosthesis type, etiology, and associations with systemic diseases, is important for tailored patient care.

•Establishing nationwide registries for VHD and procedures is also a significant step to be considered. Registries can serve as valuable resources for researchers, clinicians, and healthcare managers. Collecting comprehensive and standardized data on valve diseases and interventions can help improve patient outcomes and quality of care.

•Several initiatives have been implemented at the research and healthcare levels to develop artificial intelligence for automated diagnosis or flagging of abnormalities in cardiovascular imaging. Some of these combined strategies have been developed in Brazil in collaboration with international groups^198 - 200^and, with more research, can make screening and point-of-care diagnosis available in areas with limited personnel and resources.

•These recommendations may help enhance healthcare infrastructure, research data, and patient care in the Brazilian healthcare system. By addressing these issues, Brazil can better respond to the challenges presented by VHD and improve the overall cardiovascular health of its population in a more accurate way. Customization of health care delivery based on epidemiology and impact, leading prioritization of resource allocation, is strategic to boost efficacy, especially in the public sector.

## CHAPTER 6 – ATRIAL FIBRILLATION AND ATRIAL FLUTTER

### ICD-10 I48

**See Tables 6-1 and 6-2 and Charts 6-1 and 6-2**

**Table:** Abbreviations Used in Chapter 6

AF	Atrial Fibrillation
BNP	B-type Natriuretic Peptide
ChD	Chagas Disease
CI	Confidence Interval
DALYs	Disability-Adjusted Life Years
DOAC	Direct Oral Anticoagulants
ECG	Electrocardiogram
ELSA-Brasil	The Brazilian Longitudinal Study of Adult Health
FU	Federative Unit
GARFIELD-AF	The Global Anticoagulant Registry in the FIELD-AF
GBD	Global Burden of Disease
GIRAF	CoGnitive Impairment Related to Atrial Fibrillation
HR	Hazard Ratio
ICD-10	International Statistical Classification of Diseases and Related Health Problems, 10^th^Revision
IMPACT-AF	Multifaceted Intervention to Improve Treatment With Oral Anticoagulants in Atrial Fibrillation
INR	International Normalized Ratio
NYHA	New York Heart Association
OR	Odds Ratio
PPP	Purchasing Power Parity
RECALL	The Brazilian Registry of Chronic Atrial Fibrillation
SUS	Brazilian Unified Health System (in Portuguese, *Sistema Único de Saúde* )
TTR	Time in Therapeutic Range
UI	Uncertainty Interval

### Prevalence and Incidence

•The AF/atrial flutter prevalence varies according to the setting (population- vs healthcare-based studies) characteristics of the population included in the studies, particularly because AF prevalence is highly associated with increasing age.

•Population-based studies have shown AF prevalence varying from 0.3% to 2.5%. In the ELSA-Brasil cohort study, which included 14 424 adults with valid ECG (45.8% men; mean age, 51 years; age range, 35-74 years), the AF and atrial flutter prevalence was 0.3% (men, 0.5%; women, 0.2%), with the highest prevalence in the age range of 65-74 years (women: OR, 17; 95% CI, 2.1-135.9; men: OR, 52.3; 95% CI, 3.1-881.8).^201^When self-report was added to the definition of AF in the ELSA-Brasil, the AF prevalence rose to 2.5%, but differences in patients’ characteristics were observed between ECG-diagnosed AF and self-reported AF ( **Table 6-1** ).^202^In a cross-sectional study with 1524 elderly in São Paulo, the prevalence of AF or atrial flutter was 2.4% (men, 3.9%; women, 2.0%).^203^

**Table 6-1:** – Characteristics of participants with atrial fibrillation or flutter in the ELSA-Brasil baseline according to CHA **2** DS **2** -VASc scores

Atrial fibrillation or flutter							
	By ECG recording		By self-report only		All cases of AF or flutter		
	CHA_**2**_DS_**2**_-VASc score < 2 (N=16)	CHA_**2**_DS_**2**_-VASc score ≥ 2 (N=32)	CHA_**2**_DS_**2**_-VASc score < 2 (N=130)	CHA_**2**_DS_**2**_-VASc score ≥ 2 (N=153)	CHA_**2**_DS_**2**_-VASc score < 2 (N=146)	CHA_**2**_DS_**2**_-VASc score ≥ 2 (N=185)	p-value
Age (years; median [P25 - P75])	56.0 [49.5 – 61.2]	67.5 [58.0 – 71.2]	49.5 [45.0 – 56.0]	59.0 [53.0 – 66.0]	50.0 [45.0 – 57.0]	60.0 [53.0 – 68.0]	<0.001^**‡**^
Female sex (N (%))	3 (18.8%)	15 (46.9%)	60 (46.2%)	97 (63.4%)	63 (43.2%)	112 (60.5%)	0.002^**†**^
Coronary artery disease (N (%))	0 (0.0%)	7 (21.9%)	2 (1.5%)	64 (41.8%)	2 (1.4%)	71 (38.4%)	<0.001^**†**^
Stroke (N (%))	0 (0.0%)	1 (3.1%)	0 (0.0%)	7 (4.6%)	0 (0.0%)	8 (4.3%)	0.010^**¥**^
10-year ASCVD risk > 10% (N (%))	7 (43.8%)	16 (66.7%)	15 (11.7%)	26 (30.6%)	22 (15.3%)	42 (38.5%)	<0.001^**†**^
Prior ASCVD or 10-year ASCVD risk > 10%	7 (43.8%)	24 (75.0%)	17 (13.1%)	94 (61.4%)	24 (16.4%)	118 (63.8%)	<0.001^**†**^
Use of anticoagulants (N (%))	3 (18.8%)	16 (50.0%)	1 (0.8%)	4 (2.6%)	4 (2.7%)	20 (10.8%)	0.005^**¥**^
Use of antiplatelet agents (N (%))	2 (12.5%)	5 (15.6%)	2 (1.5%)	43 (28.1%)	4 (2.7%)	48 (25.9%)	<0.001^**†**^

*The 10-year ASCVD risk is defined only in participants without prior coronary artery disease or stroke. AF: Atrial fibrillation; ASCVD: Atherosclerotic cardiovascular disease. P-values are presented for the comparison between the CHA_*2*_DS_*2*_-VASc score < 2 (N=146) and the CHA_*2*_DS_*2*_-VASc score ≥ 2 (N=185) groups among all cases of AF or flutter using.† Chi-squared, ‡ Mann-Whitney U or ¥ Fisher’s exact tests. Source: Santos et al.^*202*^*

•Telehealth centers across Brazil have provided information on the prevalence of AF and atrial flutter based on ECG from primary care.^204 - 206^In the Telehealth Network of Minas Gerais including 1 558 421 individuals (mean age, 51±18 years; 40.2% men) with ECG performed between 2010 and 2017, the AF prevalence was 1.33%, higher in men (1.81% *vs.* 1.02%), and increased with age (OR 1.08; 95% CI, 1.08-1.08), reaching 7.0% in octogenarians (8.4% in men *vs.* 5.9% in women).^204^

•In 676 621 ECG (mean age, 51±19 years; 57.5% women) from the Federal University of São Paulo telemedicine center (2009-2016), a 7-year AF prevalence of 2.2% and a countrywide 2025-projected AF prevalence of 1.7% were observed.^205^

•The GARFIELD-AF is a hospital-based registry that included patients (≥18 years) with AF diagnosed within the previous 6 weeks and at least one additional risk factor for stroke. In Brazil, 41 sites included 1065 patients with non-valvular AF between 2010 and 2014 (mean age, 68±13 years; 55% males). New-onset AF diagnosed within the previous 6 weeks was recorded in 52% of the patients, paroxysmal in 25%, persistent in 14%, and permanent in 8%.^207^

### Mortality

•In the GBD Study 2021, age-standardized mortality rate in Brazil was estimated at 5.3/100 000 (95% UI, 4.5-5.8). **Table 6-2** shows the age-standardized mortality and DALY rates (per 100 000 inhabitants) due to AF and flutter, in 2021, for both sexes, in Brazil and its FUs. The FUs with the highest mortality rates in 2021 were Roraima, Amapa, and Tocantins, while those with the lowest were Bahia, Maranhão, and Rio Grande do Norte. However, due to reporting issues, these data can be inconsistent and should be interpreted with caution. No further stratification of 2021 GBD data for Brazil had been released until the completion of this document. For data on 2019 GBD, please refer to the previous Cardiovascular Statistics Brazil.^4^

**Table 6-2:** – Age-standardized DALY and mortality rates (per 100 000 inhabitants) due to atrial fibrillation and flutter in 2021, in Brazil and its Federative Units

Location	DALY rates	Deaths rates
Acre	126.2(106.6-152.7)	6.3(5.4-6.9)
Alagoas	122.2(100.3-147.1)	5.3(4.4-6)
Amapá	130.4(110.2-156.2)	6.7(5.6-7.5)
Amazonas	122.5(99.6-149.9)	5.6(4.8-6.1)
Bahia	116.9(93.8-145.1)	4.8(3.9-5.5)
Ceará	117.9(97-143.8)	5.3(4.5-6)
Distrito Federal	120.5(98.7-146.9)	6.5(5.6-7)
Espírito Santo	122.2(99.4-149.5)	5.5(4.5-6.1)
Goiás	118.6(96.7-145.4)	5.4(4.6-5.9)
Maranhão	109(85.9-131.6)	4.8(4-5.4)
Mato Grosso	122.7(101.7-150.3)	5.5(4.7-6)
Mato Grosso do Sul	121.1(99.5-146.1)	5.4(4.5-6)
Minas Gerais	119.1(98.7-146.2)	5.1(4.4-5.6)
Pará	118.5(96.2-146.4)	5.1(4-6)
Paraíba	119.6(97.7-148.1)	5.2(4.5-5.8)
Paraná	124.1(99.9-152.1)	5.6(4.8-6.2)
Pernambuco	113.5(93.8-138.8)	4.9(4.1-5.4)
Piaui	115.9(93.9-145.3)	5.1(4.2-5.8)
Rio de Janeiro	120.7(98.7-147.3)	5.3(4.5-5.9)
Rio Grande do Norte	114.2(91.4-141.2)	4.7(4-5.3)
Rio Grande do Sul	124.2(100.8-148.6)	5.6(4.8-6.2)
Rondônia	120.6(96.8-146.2)	5.4(4.6-6.2)
Roraima	135.6(112.1-162.4)	7.4(6.2-8.4)
Santa Catarina	123.3(101.6-152)	5.6(4.6-6.2)
São Paulo	125.2(100.6-152.9)	5.7(4.7-6.4)
Sergipe	122.8(100.9-150.6)	5.5(4.5-6.1)
Tocantins	123.1(102.3-148.3)	6(5-6.7)
Brazil	120.5(99.1-147.6)	5.3(4.5-5.8)

Source: Data derived from Global Burden of Disease Collaborative Network. Global Burden of Disease (GBD) Cardiovascular Burden Estimates 1990 and 2021, Institute for Health Metrics and Evaluation, University of Washington. ^48^

•From a dataset of 1 558 421 ECGs from primary care patients (Telehealth Network of Minas Gerais) linked to the Brazilian Mortality Information System, the overall mortality rate was 3.34% in a mean follow-up of 3.68 years. After adjusting for age and comorbidities, patients living with AF had a higher risk of overall (HR 2.10; 95% CI, 2.03–2.17) and cardiovascular (HR 2.06; 95% CI, 1.86–2.29) death, with increased risk in women, who lost their survival advantage over men when AF was present.^208 , 209^

•In a 10-year follow-up of 1462 individuals aged ≥60 years (mean age, 69 years; 61% women) included in 1997 in the Bambuí Cohort Study, AF or flutter was independently associated with an increase in all-cause mortality (HR, 2.35; 95% CI, 1.53-3.62) among patients with and without ChD (HR,1.92; 95% CI, 1.05-3.51).^210^

•The RECALL registry was a multicenter prospective registry that included and followed 4585 patients with AF up for 1 year at 89 sites across Brazil from April 2012 to August 2019. Death occurred in 8.8/100 patients/year (95% CI 8.0-9.6) and was associated in multivariable models with older age, permanent AF, NYHA class III/IV, chronic kidney disease, peripheral arterial disease, stroke, chronic obstructive pulmonary disease, and dementia. The use of anticoagulant was associated with lower mortality. Patients with TTR <60% had higher mortality and more major bleeding events compared to patients with TTR ≥60%.^210^

### Burden of Disease

•According to GBD 2021 estimates, the age-standardized DALY rate due to AF in Brazil was 120 (95% UI, 99-148) per 100 000 inhabitants. No further stratification of 2021 GBD data for Brazil had been released until the completion of this document. For data on GBD 2019, please refer to the previous Cardiovascular Statistics Brazil.^209^

### Complications

•Atrial fibrillation is related to adverse clinical outcomes other than death, such as stroke, hospitalizations, bleeding, and dementia.^211 - 215^

•Of all 429 cases of stroke (87.2% ischemic strokes) that occurred in the city of Joinville in 2015 and were included in a registry, AF was detected in 11.4% of all patients and in 58% of the cardioembolic strokes.^216^Between 2017 and 2020, of the 3303 cases of ischemic stroke in the same city, 11% had AF. Of these patients with AF, 258 (71.6%) had a previous diagnosis of the disease, and 102 (28.3%) were newly diagnosed after the stroke. Of the patients with a previously diagnosed AF, 170 (47.2%) were on anticoagulants and 88 (24.4%) were on other medications.^215^

•In a cohort of 1121 ischemic stroke patients in a 12-year follow-up, AF was independently associated with increased overall (HR 1.82; 95% CI, 1.43-2.31) and cardiovascular (HR 2.07; 95% CI, 1.36-3.14) mortality.^213^

•In the RECALL prospective registry of AF patients, the incidence rate of stroke was 2.5/100 patients/year (2.1-3.0), while that of systemic embolism was 3.5/100 patients/year (2.8-4.4). Major bleeding occurred in 2.0/100 patients/year (1.6-2.4) and hospitalization in 19.6/100 patients/year (18.4-20.9). Hospitalization was mainly related to cardiovascular causes as compared to bleeding (2.7/100 patients/year, 95% CI, 2.2-3.2).^210^

•In the GIRAF randomized clinical trial, 301 elderly patients from six centers in São Paulo, Brazil, were randomized to dabigatran or warfarin to evaluate whether the incidence of dementia differed according to the anticoagulant used after 2 years. There was no statistical difference at 5% significance level regarding any of the cognitive outcomes, after adjusting for multiple comparisons, between the dabigatran and warfarin groups.^214^

### Association of Risk Factors for Atrial Fibrillation/Flutter

•Data from the Telehealth Network of Minas Gerais with ECGs of 1 558 421 individuals (mean age, 51±18 years; 40.2% men) performed between 2010 and 2017 revealed in multivariable models adjusted for age and sex that the following self-reported comorbidities related to the presence of AF: ChD (OR 3.08; 95% CI, 2.91-3.25), previous myocardial infarction (OR 1.74; 95% CI, 1.56-1.93), chronic obstructive pulmonary disease (OR 1.48; 95% CI, 1.33-1.66), hypertension (OR 1.31; 95% CI, 1.27-1.34), dyslipidemia (OR 1.09; 95% CI, 1.03-1.16). Current smoking and diabetes were not associated with prevalent AF.^208^

•In the RECALL prospective registry of AF patients, the most common risk factors were hypertension (77.9%), previous cardiovascular events (37.3%), and diabetes (30%).^210^

•In a cross-sectional analysis of the ELSA-Brasil cohort study, the cardiovascular health score proposed by the American Heart Association was not associated with prevalent AF at baseline (80/13 141 valid ECGs, 0.8%).^216 , 217^

### Associated Comorbidities

#### Atrial fibrillation and other heart diseases

•AF correlates with other concomitant cardiovascular diseases. On echocardiogram, AF was associated with heart disease (OR = 3.9; 95% CI, 2.1 - 7.2, p <0.001) in 1518 patients (mean age, 58±16 years; 66% female) from a waiting list for echocardiogram in primary care.^218^Among 300 elderly monitored with pacemakers, the incidence of AF was 22% in a 435-day follow-up^219^and reached 85% of the patients with pacemakers and chronic kidney disease in a 1-year follow-up.^220^In another study of 186 patients with pacemakers from a single center in Southern Brazil [52% women; median age, 67 years (IQR 57-76)], the prevalence of AF was 25.3%, with an incidence of 5.64 cases/100 persons-year.^221^Among patients with cardiovascular disease visiting the emergency department, the prevalence of AF was 40% of those with decompensated heart failure^222^and 44% of those with valvular heart disease.^187^

#### Perioperative atrial fibrillation and cardiovascular surgery

•Of patients undergoing cardiac surgery, 12% to 33% had AF in the postoperative period.^223 - 226^Surgeries for valve replacement were associated with a higher occurrence of AF (31-33%) during hospitalization as compared to coronary artery bypass grafting (12-16%). Advanced age, mitral valve disease, and no beta-blocker use were associated with postoperative AF in valvular surgery.^227^Among those who underwent coronary artery bypass grafting, the postoperative AF incidence was associated with left atrial >40.5mm and age >64.5 years.^227^

•In a study to assess the impact of a quality improvement program on in-hospital mortality in a cardiovascular surgery center in São Paulo, with patients operated before and after the implementation of that program (858 patients in each group), among other benefits, there was a reduction in postoperative AF from 4.4% to 1.5%, p<0.0001.^228^

#### Atrial fibrillation and Chagas disease

•Atrial fibrillation has been consistently associated with ChD and increases the risk of death in patients with ChD.^229^In a systematic review and meta-analysis, the AF prevalence was significantly higher in patients with ChD (OR: 2.11; 95% CI, 1.40-3.19).^231^

•In a large sample of 264 324 patients undergoing tele-ECG in primary health care units, AF was observed in 5.35% of the ChD subjects and in 1.65% of the non-ChD ones (OR: 3.15; 95% CI, 2.83-3.51, adjusted for age, sex, and self-reported comorbidities).^229^

•In the Bambuí Cohort Study, 1462 participants aged ≥60 years (mean age, 69 years; ChD n=557, 38.1%), with baseline ECG, were followed up for 10 years. Atrial fibrillation was more frequently observed in ChD subjects [6.1% vs 3.4% (OR: 3.43; 95% CI, 1.87-6.32, adjusted for age, sex, and clinical variables)], in whom it was an independent risk factor for death (HR: 2.35; 95% CI, 1.53-3.62 adjusted for age, sex, clinical variables and BNP levels).^209^

#### Atrial fibrillation and Critically-Ill Patients

•In a retrospective observational study, with a review of electronic medical records and inclusion of 895 patients aged ≥80 years, included in the sepsis protocol of a high-complexity private hospital in the city of São Paulo, from January 2018 to December 2020, the incidence of AF in the sample was 13%. Atrial fibrillation was an independent risk factor for in-hospital mortality.^233^

## Health Care Utilization and Cost

## (Refer to Tables 1-1 through 1-7 and Charts 1-1 and 1-7)

•From 2008 to 2021, there were 406 666 hospitalizations for AF, varying from 25 283 in 2021 to 32 753 in 2019, just before the COVID-19 pandemic, revealing the reduction in hospitalizations due to AF in the pandemic period ( **Chart 6-1** ). From 2008 to 2021, the total costs due to hospitalizations for AF were R$ 310 739 362. After adjusting for Brazilian inflation, the costs were R$ 569 678 472, and, in international dollars converted to PPP-adjusted 2023-US$, $ 108 530 857. Interestingly, although there was a reduction in the annual hospitalizations for AF in 2020 and 2021 in the context of the COVID-19 pandemic, the annual costs adjusted for Brazilian inflation did not reduce in the same proportion.

**Chart 6-1:** – Number of hospitalizations due to atrial fibrillation in the Brazilian public health system from 2008 to 2021. Data from the Ministry of Health of Brazil – Hospital Information System of the Brazilian Unified Health System (SIH/SUS). ^49^

•Regarding ablation procedures for AF and atrial flutter, 1694 procedures were performed by the SUS from 2008 to 2021, with a reduction in 2020 as compared to 2019 (120 vs 163, respectively), which then recovered in 2021 (161 procedures). The unadjusted cost of AF ablation from 2008 to 2021 was R$ 9 614 010. After adjusting for Brazilian inflation and converting to PPP-adjusted US$ 2023, it was $ 2 751 563.

### Awareness, Treatment, and Control

•A mixed-methods study to capture perceptions of and barriers and facilitators to AF care in 11 São Paulo primary care units, from the perspective of 107 healthcare professionals, has found that the lack of AF-specific training for healthcare professionals, protocols/guidelines on AF management, educational programs for patients, as well as access to INR tests in primary care units, and availability of novel oral anticoagulants are key barriers to optimal AF care.^234^

#### Anticoagulation

•There was a high variation in the use of anticoagulation in patients with AF, from 1.5% to 91%.^204 , 205 , 235 , 236^Studies with samples from primary care were more likely to have low anticoagulation use as compared to samples recruited from tertiary centers or cardiologists.

•In primary care centers of 658 municipalities of the Minas Gerais state, mean anticoagulant use was reported by 1.5%, while, in the IMPACT-AF trial, 91% of patients enrolled in Brazil (n=360), most of them on cardiologist care at tertiary centers, were on oral anticoagulation at baseline.^204 , 235^

•In the population-based ELSA-Brasil cohort study, from 185 participants with prevalent AF and a CHA_2_DS_2_-VASc score ≥2, only 20 (10.8%) used anticoagulants. Stroke prevention in that group was associated with an older age (1.8% *vs* . 17.7% in those aged ≤ 54 and ≥ 65 years, respectively; p=0.013). A trend towards a reduced anticoagulant use was observed in women (7.1% *vs.* 16.4% in women and men, respectively; p=0.055).^202^

•In the RECALL prospective registry of AF patients, the mean (standard deviation) CHA_2_DS_2_-VASc score was 3.2 (1.6); median HAS-BLED score was 2 (2, 3). At baseline, 78% of the patients were on anticoagulants (62.6% taking vitamin K antagonists and 37.4% were taking DOACs). The primary reasons for not using an oral anticoagulant were physician judgment (24.6%) and difficulty in controlling (14.7%) or performing (9.9%) INR ( **Chart 6-2** ).^210^

**Chart 6-2:** – Antithrombotic therapies during one year of follow-up (A) and baseline antithrombotic therapies (B) according to CHA *2* DS *2* -VASc. VKA: vitamin K antagonist; DOAC: direct oral anticoagulant; AP: antiplatelet. Source: Lopes et al. ^210^

•Anticoagulation use is associated with lower risk of death in the RECALL prospective registry of AF patients and in a cohort of 200 ischemic stroke patients with AF (oral anticoagulant time-dependent effect: multivariable HR, 0.47; 95% CI, 0.30–0.50) and stroke mortality (oral anticoagulant time-dependent effect ≥ 6 months: multivariable OR, 0.09; 95% CI, 0.01–0.65).^210 , 236^

•The quality of warfarin therapy has been evaluated using the parameter TTR in different samples in Brazil. The TTR of anticoagulation for AF has ranged from 31% to 67% in the studies.^210 , 233 , 235 - 238^In the RECALL prospective registry of AF patients, mean (standard deviation) TTR for the study period was 49.5% (27.5). During follow-up, the use of anticoagulants and INR in the therapeutic range increased to 87.1% and 59.1%, respectively.^210^

•In a study that aimed to evaluate the costs related to anticoagulant therapy in one year of AF patients (n=90) treated in a public university hospital, the authors have found that total costs were higher in the group taking DOACs than in that taking warfarin. However, a 40% reduction in the price of DOACs could make it feasible to incorporate these drugs into the Brazilian public health system.^237^

#### Rhythm or rate control (medication, cardioversion, catheter ablation)

•A cross-sectional study with 167 AF patients has found that rate control was more common than rhythm control as treatment strategy (79% vs. 21%; p<0.001).^239^

•Data from 125 primary care centers showed that, of 301 patients with AF, 91 (30.2%) were receiving neither rate nor rhythm control therapy. From the remaining patients with AF, 70% used only rate control agents and 30% used at least one antiarrhythmic drug.^205^

•In the RECALL prospective registry of AF patients, 4.4% had a history of previous AF ablation, 0.9% atrioventricular node ablation with pacemaker implantation, and 25.2% had a previous cardioversion. Nearly 30% used an antiarrhythmic drug for rhythm control, led by amiodarone. For heart rate control, 69.5% used beta-blockers, 25.7% were receiving non-dihydropyridine calcium channel blockers, and 15.8% were on digitalis.^210^

## Impact of the COVID-19 Pandemic

•Atrial fibrillation is associated with a greater risk for adverse events for patients with COVID-19,^239^although a retrospective single-center study of hospitalized patients due to COVID-19 in Brazil (n=128) has not observed the association of AF and mortality.^240^

•There was a reduction in hospitalizations and procedures for AF during the pandemic period, particularly in 2020 ( **Chart 6-1** ). This reduction was probably related to indirect effects of the pandemic, when there was a decrease in cardiovascular hospitalizations in general in Brazil and worldwide, because elective procedures were postponed, contact with healthcare providers was avoided, and cardiovascular care was disrupted.^14 , 241^

### Future Directions

•Population-based cohort studies results regarding AF incidence and determinants in Brazil are underway.

•Screening of AF in population-based studies or selected populations using ECG or devices, including wearables and longer duration patches, should bring information on the relevance of including this strategy in primary care or specialized centers.

•Studies using artificial intelligence algorithms to diagnose or predict AF from ECGs have been developed, including derived from Brazilian data.^242^These artificial intelligence algorithms may be a tool to improve AF diagnosis and personalize screening strategies according to risk.

•Strategies to reduce gaps in AF treatment must be developed, including evaluating the cost-effectiveness of incorporating DOACs in the public health system.

•Implementation strategies to enhance anticoagulation use among AF patients should be encouraged, particularly in primary care settings.

## CHAPTER 7 – HYPERTENSION

### ICD-10 - I10

**See Tables 7-1 through 7-5 and Charts 7-1 through 7-3**

**Table:** Abbreviations Used in Chapter 7

aPR	Adjusted Prevalence Ratio
BMI	Body Mass Index
CI	Confidence Interval
DALYs	Disability-Adjusted Life Years
DBP	Diastolic Blood Pressure
ELSA-Brasil	Brazilian Longitudinal Study of Adult Health (in Portuguese, *Estudo Longitudinal de Saúde do Adulto* )
FU	Federative Unit
GBD	Global Burden of Disease
ICD-10	International Statistical Classification of Diseases and Related Health Problems, 10thRevision
OR	Odds Ratio
SBP	Systolic Blood Pressure
SUS	Brazilian Unified Health System (in Portuguese, *Sistema Único de Saúde* )
VIGITEL	Surveillance System of Risk and Protection Factors for Chronic Diseases by Telephone Survey (in Portuguese, *Sistema de Vigilância de Fatores de Risco e Proteção para Doenças Crônicas por Inquérito Telefônico* )

### Overview

•For standardization purposes, in this document, hypertension was characterized as sustained systemic blood pressure levels equal to or greater than 140 mm Hg for SBP and/or equal to or greater than 90 mm Hg for DBP.^243^

•The percentage prevalence values will be presented followed by 95% CI, as available in the cited studies. In population studies, hypertension can be measured or self-reported. The former derives from direct blood pressure measurement using standardized techniques and is described in each document, while self-reported hypertension is characterized by a positive answer to a question about the presence of this medical diagnosis or by a positive answer regarding the use of antihypertensive medications,^244^depending, therefore, on the patient’s access to the diagnosis and understanding of this information, resulting in differences in hypertension prevalence according to how data are collected.

•When the GBD study is the data source, the risk is attributable to high SBP, as described in a previous publication.^245^

### Incidence and Prevalence of Hypertension

#### Children and Adolescents

•A systematic review and meta-analysis by Paiva *et al* ., using 15 studies and including 43 227 adolescents, has investigated the prevalence of metabolic syndrome and its components in Brazilian adolescents. The authors have estimated a hypertension prevalence of 10.3% (7.8-13.5) for Brazilian adolescents. Hypertension was the third most prevalent component of metabolic syndrome in that population, following low levels of high-density lipoprotein-cholesterol [22.1% (12.5-36.2)] and abdominal obesity [11.0% (8.1-14.9)].^246^

•In a longitudinal study evaluating 469 children and adolescents from Southern Brazil, aged 7-17 years (43.1% boys), SBP and DBP, waist circumference, BMI, body fat percentage, lipid profile, glucose, cardiorespiratory fitness, and rs9939609 polymorphism have been assessed. Cumulative incidence of hypertension was calculated, and multinomial logistic regression was conducted. Blood pressure measurement was classified according to 90 and 95 percentiles for borderline hypertension and hypertension, respectively.^247^The incidence of hypertension was 11.5% after three years of follow-up. Overweight or obese individuals were more likely to become borderline hypertensive (overweight: OR 3.22, 95% CI, 1.08-9.55; obesity: OR 4.05, 95% CI, 1.68-9.75), and obese individuals were more likely to become hypertensive (obesity: OR 4.84, 95% CI, 1.57-14.95). High-risk waist circumference and body fat percentage values were associated with hypertension development (OR 3.41, 95% CI, 1.26-9.19; and OR 2.49, 95% CI, 1.08-5.75, respectively).^248^

#### Adults

•Malta *et al* .,^248^in a study that analyzed data from the 2019 National Health Survey,^249^have found that the prevalence of self-reported hypertension in the country was 23.93% (95% CI, 23.42- 24.43), with 26.45% (95% CI, 25.75-27.15) being female and 21.06% (20.37-21.75) being male ( **Tables 7-1 and 7-2** ). Regarding age groups, a higher prevalence was observed in those over 60 years of age and those with low educational level, in both sexes.^248^It is noteworthy that the prevalence of hypertension in females tends to be higher than in males when assessed through self-reported data, a phenomenon not observed in studies using measured blood pressure.

•In another study, Macinko *et al* . have evaluated noncommunicable chronic diseases and their relationship with educational level inequalities between 2013 and 2019.^250^This study used data from the Brazilian National Health Surveys carried out in 2013^251^and 2019,^249^and the unadjusted data on the hypertension prevalence in adults aged over 18 years showed an increase from 22% (95% CI, 21.4-22.7) in 2013 to 25.9% (95% CI, 25.4-26.4) in 2019, in both sexes.^250^

•According to VIGITEL data for 2021, the percentage of patients aged 18 years or older who self-reported a diagnosis of hypertension in Brazil was 26.3% (95% CI, 25.1-27.6), being 27.1% (95% CI, 25.5-28.7) among females and 25.4% (95% CI, 23.4-27.4) among males.^252^In relation to the capitals, this percentage varied from 19.3% (95% CI, 15.7-23.0) in the city of São Luís to 32% (95% CI, 27.5-36.4) in the city of Rio de Janeiro in both sexes.^252^Among females, the highest percentage observed was 32.2% (95% CI, 27.5-36.9) in the city of Belo Horizonte, and the lowest was 19.3% (95% CI, 16.0-22.6) in the city of Macapá. On the other hand, among males, the highest percentage found was 32.2% (95% CI, 24.7-39.7) in the city of Rio de Janeiro, and the lowest was 13.8% (95% CI, 8.1-19.6) in the city of São Luís.^252^Regarding age groups, individuals aged 65 years or older had the highest hypertension prevalence in the country, 61% (95% CI, 59.0-63.0), and in that age group, women had a higher prevalence as compared to men, 63.7% (95% CI, 61.6-65.8) and 57.1% (95% CI, 53.4-60.7), respectively.^252^ **Chart 7-1** shows the temporal evolution of the percentage values of hypertension in the country, between 2007 and 2021, in both sexes. **Chart 7-2** shows the hypertension prevalence in the country by FU according to the percentage quartiles in 2021, based on the VIGITEL study 2021.^252^Again, using self-reported data, a higher prevalence was observed in females as compared to males, differently from data using measured blood pressure.

**Chart 7-1:** – Tends in hypertension prevalence in Brazilian capitals, between 2007 and 2021, for both sexes. Data from the VIGITEL Brazil 2021. ^252^

**Chart 7-2:** – Prevalence of hypertension in Brazil by Federative Unit according to the percentage quartiles, in 2021, based on the VIGITEL study 2021. Data from the VIGITEL Brazil 2021. ^252^

•In the ELSA-Brasil cohort study, Scaranni *et al* . have assessed the relationship between the consumption of ultra-processed foods and the incidence of hypertension in previously normotensive patients. A total of 8754 participants aged 35-74 years were included, and 1312 hypertension cases were observed at the end of a four-year follow-up period.^253^Furthermore, those with a high consumption of ultra-processed foods were found to have a greater risk of developing hypertension, according to the authors (OR 1.23, 95% CI, 1.06-1.44).^253^

•In addition, in the ELSA-Brasil cohort study, when the hypertension incidence was evaluated according to sex and race, the highest hypertension incidence was observed in Black men (59.4/1000 person-years), while the lowest incidence was observed in White women (30.5/1000 person-years).^254^After adjusting for age and family history of hypertension, the incidence rate ratio was higher in Black men (2.25; 95% CI, 1.65–3.08), Brown men, Black women, Brown women, and White men compared to White women.^254^

•Alcohol consumption was found to be associated with a higher incidence of hypertension only in the male population in Brazil. According to Coelho *et al* .,^255^in an analysis of the ELSA-Brasil cohort study involving 3990 individuals aged 35-74 years, men in the highest tertiles of total alcohol consumption, beer consumption, wine consumption, and spirits consumption had a higher OR for hypertension compared to those in the lowest tertiles (1.62 [95% CI, 1.14-2.29], 1.51 [95% CI, 1.07-12.13], 1,71 [95% CI, 1.01-2.86], and 2.01 [95% CI, 1.21-3.32], respectively).

## Mortality Attributable to Hypertension

•The GBD estimates for mortality and DALY rates and absolute number of deaths attributable to high SBP in Brazil and its 27 FUs from 1990 to 2019 were shown in the previous version of this document, as the estimates from GBD have not been updated until the completion of this document.^4^

## Morbidity and Burden of Disease Attributable to Hypertension

•The trends of the burden of diseases attributable to exposure to risk factors were analyzed according to sex and age groups, using GBD estimates from 1990 to 2019. For mortality, hypertension was the leading risk factor responsible for most deaths (104.8 per 100 000 deaths). For DALYs, during this period, high BMI, high SBP, and high fasting plasma glucose moved up to the top three positions in the ranking. For DALYs, high BMI was the leading risk factor for women and alcohol consumption for men.^256^

•Pires *et al.* have studied the prevalence of hypertension, obesity, and/or diabetes cluster and investigated its association with sociodemographic and behavioral factors. The authors verified that the hypertension prevalence was higher in men than in women [17.8% (95% CI, 17.0-18.6) vs. 11.4% (95% CI, 10.8-12.0)]. The prevalence of multimorbidity was 9.6%. The odds of multimorbidity were higher with increasing age among Blacks, but lower in the Northern region of the country for both sexes. The odds of multimorbidity of noncommunicable diseases increased among women with lower educational levels and a sedentary lifestyle, and among men who lived with a partner and were physically inactive. Women as compared to men had a higher prevalence of obesity [15.9% (95% CI, 15.2-16.6) vs. 9.4% (95% CI, 8.8-10.0)], obesity and diabetes mellitus [1.6% (95% CI, 1.4-1.8) vs. 0.9% (95% CI, 0.9-1.2)], and three noncommunicable diseases [1.3% (95% CI, 1.1-1.6) vs. 0.8% (95% CI, 0.6-1.0)].^257^

•Between the years 1990 and 2019, the estimated deaths from chronic kidney disease secondary to hypertension increased from 2.4 per 100 000 inhabitants in 1990 to 5.38 per 100 000 inhabitants in 2019, with the Southeastern region having the highest mortality rates. Regarding sex, higher rates were observed among males; however, over the years, this difference has been reduced. The age group of ≥70 years was the most affected, standing out with the highest death rates.^258 - 262^

## Impact on Cardiovascular Health

•Malta *et al.,* using data from the National Health Survey 2013 and 2019, have evaluated 60 202 individuals and demonstrated that Black individuals had more hospitalization for hypertension or some complication (PR=1.2; 95% CI, 1.05-1.38) and intense or very intense degree of limitation of daily living activities (PR=1.37; 95% CI, 1.06-1.76).^263^Moreover, they reported conflicting results regarding the comparison of quality of care for hypertensive patients. Although there was an increase in the use of medication from the federal program, a reduction in the access to a specialist, when necessary, was also observed.^261^

•In 4717 individuals participating in the ELSA-Brasil cohort study, free of diabetes and cardiovascular disease at baseline (2008-2010), insulin resistance evaluated by HOMA-IR was associated with a chance of developing pre-hypertension by 51% (95% CI, 1.28-1.79) and hypertension by 150% (95% CI, 1.48-4.23). Even in individuals without obesity (BMI<25 kg/m^2^), insulin resistance was associated with the incidence of pre-hypertension (OR 1.41; 95% CI, 1.01-1.98) and hypertension (OR 3.15; 95% CI, 1.27-7.81).^260^

•The role of urbanization on cardiometabolic health has been elegantly investigated by Kramer *et al* . They have performed a systematic review and meta-analysis from 46 studies, including a total of 20 574 adults from at least 33 Indigenous Brazilian ethnicities, to understand the metabolic health and the role of urbanization and of deforestation (environmental) on cardiovascular risk. They assessed the prevalence of obesity and related cardiometabolic risk factors. Meta-analyses of the prevalence of obesity showed higher rates of obesity among Indigenous individuals living in West-Central Brazil (23% [95% CI, 17–29]) and Southern Brazil (23% [13–34]) than among those living in less urbanized regions (Northern Brazil: 11% [8-15]). The same pattern was verified for hypertension: the prevalence was highest in Southern Brazil (30% [10–50]) and lowest in populations living in the Northern region, the least urbanized (1% [1-2]). The prevalence of obesity was 3.5 times higher in individuals living in urbanized Indigenous territories (28%) than in those living in the native Amazon rainforest (8%). The authors have not found incremental change in blood pressure with ageing in Indigenous individuals who lived in accordance with their traditional lifestyle, in contrast to those living in urbanized regions. For the years 1997 and 2019, the cardiovascular mortality rate in individuals living in the most urbanized places was 2.5 times greater than that observed in the North. On the other hand, the incremental rise in cardiovascular mortality in the past two decades among Indigenous Brazilians was observed only in the Northern and Northeastern populations (2.7-time increase).^260^

## Awareness, Treatment, and Control

•Tavares *et al* . have investigated the control of cardiovascular health according to the American Heart Association construct of seven cardiovascular metrics among 400 adult patients followed-up in the Family Health Strategy in Sergipe, Brazil.^262^Only 32.5% had controlled cardiovascular health (≥5 metrics ideally controlled) and, regarding hypertension, only 35% of that population was ideally controlled (blood pressure < 120/80 mm Hg).^250^Being a woman, young, and following health advice from family members and neighbors have a positive influence in controlling cardiovascular health.^262^

•The National Health Survey has estimated that of 88 531 individuals, 23.9% had self-reported hypertension. Among those, 57.8% reported medical care in the past six months: 61.1% in public health services and 45.8% in primary care units^262^( **Tables 7-3, 7-4, and 7-5, and Chart 7-3** ).

**Chart 7-3:** – Comparison of healthcare indicators of adults with arterial hypertension. National Health Survey (PNS), Brazil, 2013 and 2019. Source: Malta et al. ^261^

•The proportion of individuals living with diabetes and hypertension obtaining medication from the Brazilian Popular Pharmacy Program was estimated using data from the 2019 Brazilian National Health Survey: 45.1% (95% CI, 43.7-46.5%) for hypertension and 51.5% (95% CI, 49.5-53.6%) for diabetes. Medication obtainment was higher in the Southern region and lower in the higher levels of education and income^264^( **Tables 7-3, 7-4, and 7-5, and Chart 7-3** ).

•Using data from the ELSA-Brasil cohort study, 3897 participants with hypertension were investigated regarding blood pressure control, self-reported race, and neighborhood economic segregation in a cross-sectional study. After adjusting for age, sex, education, and study center, the unexplained portion (disparity residual) of race on uncontrolled hypertension was 18.2% (95% CI, 13.4%-22.9%) for Black vs. White participants, and 12.6% (8.2%-17.1%) for Brown vs. White participants. However, the explained portion (disparity reduction) through economic segregation was − 2.1% (− 5.1%; 1.3%) for Black vs. White and 0.5% (− 1.7%; 2.8%) for Brown vs. White participants. Although uncontrolled hypertension was greater for Black and Brown vs. White individuals, racial inequities in uncontrolled hypertension were not explained by economic segregation.^265^

## COVID-19 and Hypertension

•During the pandemic, there was a slight rise in hypertension prevalence in the Brazilian capitals according to the VIGITEL data, when comparing to the stable trend from 2009 to 2019 ( **Chart 7-1** ).

•Malta *et al* . investigated the association between self-reported diagnosis of noncommunicable diseases, including hypertension, and the adherence to social distancing and the use of health services during the COVID-19 pandemic through a telephone survey in 2020. Individuals with noncommunicable diseases showed a greater adherence to intense social distancing (aPR: 1.07; 95% CI, 1.03-1.11), sought out health services more often (aPR: 1.24; 95% CI, 1.11-1.38), and found greater difficulty in scheduling doctor’s appointments (aPR: 1.52; 95% CI, 1.35-1.71), receiving healthcare treatment (aPR: 1.50; 95% CI, 1.22-1.84) and medication (aPR: 2.17; 95% CI, 1.77-2.67), and undergoing tests (aPR: 1.78; 95% CI, 1.50-2.10) and scheduled interventions (aPR: 1.65; 95% CI, 1.16-2.34).^266^

•Hypertension control during COVID-19 pandemic has been evaluated by Feitosa *et al* . in Brazil. The authors found a slight reduction in office and home blood pressure monitoring in the early months following the COVID-19 outbreak among 987 treated hypertensive patients with questionable clinical significance, compared to 27 699 patients evaluated before the COVID-19 outbreak. Among untreated patients, no difference in hypertension control was observed.^267^Duarte *et al* . have investigated 194 COVID-19-related deaths in Southern Brazil. Most of the population was male (63.4%), ≥60 years of age (82.5%), and white (82.5%). Approximately half of the patients had multimorbidity. The prevalences of hypertension, diabetes, and cardiac disease were 29.4% (23.3-36.2), 28.0% (22.1- 34.8), and 38.7 % (32.0-45.7), respectively.^268^

•In a retrospective study of 1276 deaths due to COVID-19 in the state of Pernambuco, Santos *et al* . have reported that 26.48% of the patients had hypertension as a previous comorbidity.^269^

## Future Directions

•Regarding the decrease in the cardiovascular burden of hypertension in Brazil, there is a gap regarding a deeper and more holistic knowledge about how to improve hypertension prevention, awareness, treatment, and control, and its relationship with other cardiovascular unfavorable behaviors and risk factors, such as proposed by the American Heart Association.^216^The current understanding points out to hypertension, at the population level, as the main determinant of cardiovascular morbidity and mortality nationwide. Therefore, better data to measure population outcomes and the healthcare system performance are urgently needed in Brazil to implement scientific investigative strategies of how to improve those outcomes.^110 , 270 , 271^

•Moreover, we must move on from only generating evidence to an action model of health policies to correct the suboptimal reality already disclosed by science. National population strategies with effective campaigns to promote healthy habits (i.e., salt decrease in diet, taxation of unhealthy foods, increase in physical activity, and improvement of psychological health and sleep pattern), allied to more effective identification and treatment of individuals at greater cardiovascular risk, and objective surveillance of outcomes must be top priority for the near future.^110 , 270 , 271^

•Better research related to disparities regarding access, timelines, and outcomes for hypertensive patients using the SUS as compared to patients using private healthcare service, as the benchmarking, could provide new public health policies to decrease hypertension burden in our society.

•Considering that approximately 75% of Brazilians are SUS users, it is imperative to continuously measure the outcomes of hypertension programs implemented by the SUS, such as the Family Health Strategy, and to compare them to those reached by the private healthcare system.^261^

## CHAPTER 8 – DYSLIPIDEMIA

### ICD-10 E78 (E78.0 to E78.9); ICD-10-CM E78 (E78.0 to E78.9)

**See Tables 8-1 through 8-4**

**Table:** Abbreviations Used in Chapter 8

AMI	Acute Myocardial Infarction
CI	Confidence Interval
CVD	Cardiovascular Diseases
DALYs	Disability-Adjusted Life Years
ELSA-Brasil	The Brazilian Longitudinal Study of Adult Health
ERICA	Brazilian Study of Cardiovascular Risk in Adolescents (in Portuguese, *Estudo dos Riscos Cardiovasculares em Adolescentes* )
GBD	Global Burden of Disease
HDLc	High-Density-Lipoprotein Cholesterol
LDLc	Low-Density-Lipoprotein Cholesterol
OR	Odds Ratio
PNAUM	Study on the Access to, Use, and Promotion of Rational Use of Medicines in Brazil (in Portuguese, *Pesquisa Nacional sobre Acesso, Utilização e Promoção do Uso Racional de Medicamentos no Brasil* )
PNS	National Health Survey (in Portuguese, *Pesquisa Nacional de Saúde* )
PR	Prevalence Ratio
SUS	Brazilian Unified Health System (in Portuguese, *Sistema Único de Saúde* )
TC	Total Cholesterol
TG	Triglycerides
UI	Uncertainty Interval

### Introduction

•Dyslipidemia is defined as abnormal serum levels of lipids, including cholesterol, its fractions, and/or TG. Dyslipidemia is a well-known risk factor for CVD.^273^Even in the primary prevention setting, randomized studies have already shown that the treatment of dyslipidemia effectively reduces CVD.^274^Data on mean cholesterol levels and prevalence of dyslipidemia in adults were obtained primarily from the PNS laboratory data collected in 2014-2015, while, in adolescents, from the ERICA Study. Smaller studies of regional basis were used, when appropriate, for additional descriptions. This document was based on data from the 2021 Cardiovascular Statistics,^4^except when more recent data were available.

•This chapter provides data on TC, LDLc, HDLc, and TG. The definitions of dyslipidemia vary historically and according to the positions of the local societies of cardiology. In this chapter, for the purpose of classification and unless stated otherwise, the term dyslipidemia is used as follows: 1) for adults: TC ≥ 200 mg/dL, LDLc ≥ 130 mg/dL, HDLc < 40 mg/dL, or TG ≥ 150 mg/dL;^275^2) and for children and adolescents: TC ≥ 170 mg/dL, LDLc ≥ 130 mg/dL, HDLc < 45 mg/dL, or TG ≥ 130 mg/dL.^276^

### Prevalence

#### Young individuals

•The ERICA Study, published in 2021, reported data of 38 069 schoolchildren (female sex, 60%) aged 12-17 years, from the capitals of the 27 Brazilian Federative Units, in addition to five sets of municipalities with more than 100 000 inhabitants, in all five Brazilian geographic regions.^276^The following mean values were found: TC, 148 mg/dL (95% CI, 147-149 mg/dL); LDLc, 85 mg/dL (95% CI, 84-86 mg/dL); HDLc, 47 mg/dL (95% CI, 47-48 mg/dL); and TG, 78 mg/dL (95% CI, 76-79 mg/dL). The prevalence of abnormal lipid levels (increases) in the general population were as follows: of TC, 20.1% (95% CI, 19-21.3%); of LDLc, 3.5% (95% CI, 3.2-4%), and of TG, 7.8% (95% CI, 7.1-8.6%). The prevalence of low HDLc was 47% (95% CI, 45-49%). Such data stratified by age and sex can be seen in **Table 8-1** .

**Table 8-1:** – Mean plasma lipid levels, prevalence of borderline and high lipid levels, and estimated population with abnormal lipid levels, by sex and age group. ERICA-Brasil, 2013-2014

Lipids	Mean		Borderline		High		Estimated population with abnormality
	mg/dL	95% CI	%	95% CI	%	95% CI	
Total cholesterol							
General population	148.1	147.1-149.1	24.2	22.7-25.8	20.1	19.0-21.3	2 940 705
Men	143.6	142.4-144.8	22.7	20.4-25.2	15.3	13.9-16.9	1 256 102
Women	152.6	151.4-153.9	25.7	24.5-27.0	24.9	23.4-26.5	1 684 602
12-14 years	149.4	148.0-150.7	25.8	24.3-27.4	20.7	19.1-22.5	937 793
15-17 years	147.1	145.8-148.3	22.8	20.8-24.9	19.6	18.0-21.2	2 002 911
LDLc							
General population	85.3	84.5-86.1	19.5	18.5-20.5	3.5	3.2-4.0	1 526 733
Men	83.4	82.2-84.5	17.4	16.0-18.9	2.9	2.3-3.6	669 805
Women	87.2	86.3-88.1	21.5	20.2-22.9	4.3	3.7-4.9	856 928
12-14 years	86.2	85.1-87.3	20.6	19.0-22.4	3.7	3.1-4.4	467 877
15-17 years	84.5	83.5-85.5	18.4	17.2-19.7	3.4	2.9-4.1	1058 856
Triglycerides							
General population	77.8	76.5-79.2	12.0	11.0-13.0	7.8	7.1-8.6	1 312 329
Men	76.4	74.7-78.1	10.9	9.8-12.2	7.6	6.5-8.8	610 449
Women	79.3	77.8-80.7	13.0	11.8-14.2	8.1	7.3-9.0	701 880
12-14 years	78.9	76.7-81.0	12.7	11.0-14.6	8.3	7.2-9.5	434 638
15-17 years	76.9	75.8-78.1	11.3	10.2-12.4	7.4	6.6-8.4	877 690
HDLc	Mean		Low				
General population	47.3	46.7-47.9	46.8	44.8-48.9	-	-	3 104 161
Men	44.9	44.4-45.5	55.9	53.7-58.2	-	-	1 256 003
Women	49.6	48.9-50.3	37.8	35.4-40.2	-	-	1 848 158
12-14 years	47.4	46.7-48.1	45.0	42.3-47.8	-	-	819 980
15-17 years	47.2	46.4-48.0	48.4	45.9-50.8	-	-	2 284 181

LDLc: low-density-lipoprotein cholesterol; HDLc: high-density-lipoprotein cholesterol a: change = borderline + high levels. b: Population estimates for the domains were obtained by processing the microdata from the 2000 and 2010 Demographic Census from the Brazilian Institute of Geography and Statistics. Modified from Faria Neto JR et al. ^276^

•For that same age group, 1200 adolescents in the Distrito Federal were assessed in 2022 (mean age, 14.8 years; female sex, 50.4%; white, 35.6%; mixed heritage, 53.5%; and black, 6%). The prevalence of TC ≥170 mg/dL was 30.6% (95% CI, 27.6-33.7), of LDLc ≥110 mg/dL, 21.3% (95% CI, 19.0-23.7), of HDLc ≤45 mg/dL, 41.8% (95% CI, 38.1-45.4), and of TG ≥90 mg/dL, 30.5% (95% CI, 27.4-33.8). Similarly to the findings of the ERICA Study, the most frequent change was low levels of HDLc, with very similar values.^277^

•In Santa Catarina, the lipid profile of 1011 schoolchildren aged 6-14 years (girls, 52.4%) was assessed in 2013, and the following mean levels were found: TC, 172±27 mg/dL in girls and 170±28 mg/dL in boys; LDLc, 104±24 mg/dL in girls and 104±27 mg/dL in boys; HDLc, 49±11 mg/dL in girls and 49±11 mg/dL in boys; and TG, 80 (24-459) mg/dL in girls and 77 (14-752) mg/dL in boys.^278^

•In 2016, 511 children from a similar age group (6-9 years; male sex, 46.77%) were assessed in the city of Vitória, Espírito Santo, regarding their lipid profile changes. Of that population, high levels of TC were observed in 32.7%, of LDLc in 9.2%, and of TG in 4.1%, while low levels of HDLc were observed in 27%.^279^Another study conducted in 2012 in the city of Salvador, assessing 1131 children (age, 7-15 years; male sex, 50.1%), identified that 25.5% (95% CI, 22.7-28.3) of them had dyslipidemia (TC ≥ 170 mg/dL and/or TG ≥ 130 mg/dL). Dyslipidemia was associated with excess body weight (OR: 3.40; 95% CI, 2.07-5.58) and moderate to high consumption of high-risk food, such as whole dairy products, fried food, fat from animal origin, and ultra-processed food (OR: 1.49; 95% CI, 1.01-2.19).^279^

#### Adults

•For the adult Brazilian population, the study by Malta *et al* .^275^using data from the PNS 2014-2015 has shown prevalence of high levels of TC and of LDLc (32.7% and 18.6%, respectively) and of low levels of HDLc (31.8%). That study identified the following mean levels: TC, 185 mg/dL; HDLc, 46 mg/dL; and LDLc, 105 mg/dL. The prevalence of high TC was higher among women, while the prevalence of low HDLc was higher in men. **Tables 8-2 to 8-4** synthesize those findings and show a sex-stratified analysis according to different age groups, educational level, skin color, and Brazilian geographic region. In general, higher educational levels were related to lower prevalence of high TC and LDLc levels, as well as of low HDLc levels. Older age groups showed a higher prevalence of elevated levels of TC and LDLc. Living in the Southern and Southeastern regions were related to a lower prevalence of low HDLc levels. A statistically significant relationship between self-reported skin color and lipid profile was less clear, but black women had a lower prevalence of low HDLc levels.^275 , 280^In addition, in that population, overweight and obesity were associated with increased LDLc levels.^281^Other factors associated with lipid profile changes that have been reported in the Brazilian population include physical activity^282^and seasonal variations.^283^

•The PNS 2019 used the self-reported diagnosis of high cholesterol levels, and, among the 88 531 adults assessed, a 14.6% prevalence of high cholesterol was identified. The factors most strongly associated with that condition, measured with their PR, were as follows: female sex (PR = 1.44; 95% CI, 1.40-1.52); age ≥ 60 years (PR = 3.80; 95% CI, 3.06-4.71); healthcare insurance coverage (PR = 1.33; 95% CI, 1.24-1.42); poor or very poor health self-assessment (PR = 1.75; 95% CI, 1.60-1.90); hypertension (PR = 1.78; 95% CI, 1.68-1.89); diabetes (PR = 1.54; 95% CI, 1.45-1.65); renal failure (PR = 1.33; 95% CI, 1.15-1.53); obesity (RP = 1.27; 95% CI, 1.18-1.36); former tobacco use (PR = 1.13; 95% CI, 1.07-1.20); alcohol abuse (PR = 1.11; 95% CI, 1.01-1.21); active leisure time (PR = 1.22; 95% CI, 1.15-1.30).^284^

•In 2016, the ELSA-Brasil study found the following percentages in women and men, respectively: hypertriglyceridemia, 23.2% and 40.7%; low HDLc levels, 20.7% and 14.7%; and high LDLc levels, 57.6% and 58.8%. In addition, the ELSA-Brasil study reported small differences in the lipid profile according to skin color, but with apparently limited clinical impact.^285^

•In 2023, Fonseca *et al* . conducted a study on CVD risk factors in the primary healthcare setting with 7724 individuals without established CVD, living in 32 municipalities of São Paulo State. The prevalence of dyslipidemia (reported in the medical record) was 70.1% (68.6% in men, and 71.2% in women).^286^

## Attributable Risk

•The most recent data on risk of death or burden of disease (DALYs) attributed to elevated LDLc levels estimated in the GBD Study were published in the previous version of this document.^4^Briefly, between 1990 and 2019, deaths from CVD attributable to high LDLc levels in Brazil increased in absolute numbers from 68 327 (95% UI, 55 097-83 768) to 99.375 (95% UI, 78 039-126 143), but the age-standardized mortality rate decreased by 51.3%, from 88.6 (95% UI, 67.8-114.8) to 43.1 (95% UI, 33.4-55.9) per 100 000, as the result from population aging.^4^

## Familial Hypercholesterolemia

•The prevalence of familial hypercholesterolemia can be estimated by using the *Dutch Lipid Clinic Network* criteria. According to those criteria, the ELSA-Brasil Study has reported a prevalence of familial hypercholesterolemia of 1 in 263 individuals, and higher in those of black skin (1 in 156) and of mixed heritage (1 in 204) than in white ones (1 in 417).^287^

•Despite controversies on the use of cascade screening to identify relatives of individuals with familial hypercholesterolemia, a Brazilian study has shown that 59% of the relatives of individuals with mutations carried such mutations, suggesting a high prevalence of familial hypercholesterolemia in that selected subgroup.^288^However, in a study by Coutinho *et al* ., involving elderly patients with hypercholesterolemia identified using cascade screening, the presence of previous CVD was a predictor of incident CVD, while the presence of a genetic variant associated with familial hypercholesterolemia was not, when comparing patients of similar ages and with similar cholesterol levels.^289^

•Regarding raising awareness on familial hypercholesterolemia and its treatment, Santos *et al* . have reported the results from a study of a database with 70 000 individuals submitted to routine and mandatory health assessment sponsored by their employer at a private hospital of São Paulo.^290^Of the 70 000 mil individuals, 1987 met the established criteria for familial hypercholesterolemia (LDLc ≥ 190 mg/dL or LDLc ≥ 160 mg/dL on statin use). A sample of 200 individuals was selected to fill in a questionnaire. Of those 200 individuals, familial hypercholesterolemia was suspected by the attending physician in only 29 (14.5%), although most of them (97%) knew their high serum levels of cholesterol. Only 18% were aware of their high risk for CVD, 30% knew their recommended LDLc levels, and 37% used no lipid-lowering drug.

## Knowledge, Treatment, and Control

•Analyzing records obtained from the Brazilian ‘Family Health Strategy’, real-world data on the use of statins for secondary prevention of 2 133 900 individuals from the entire country have been reported.^91^Of those individuals, 35 103 (mean age, 66.2 years; male sex, 49.5%) had had a previous AMI or stroke. In that population, only 6.7% were on statins. That rate dropped to 0.6% when assessing the use of high-potency statins. Previous AMI or use of anti-hypertensive drugs was associated with a higher chance of using lipid-lowering drugs [OR 4.53 (95% CI, 3.66-5.60) and OR 5.47 (95% CI, 4.60-6.47), respectively]. In addition, living in the Southern region was a strong predictor of statin use [OR 4.53 (95% CI, 3.66-5.60)].^91^

•An analysis conducted in the ELSA-Brasil Study, including 15 096 adults aged 35-74 years, has explored the prevalence of high LDLc levels (according to the NCEP-ATP-III criteria) and the proportion of participants aware of their diagnosis.^291^The frequency of participants with elevated LDLc levels was 45.5%, of whom only 58.1% were aware of the diagnosis. Among those participants with elevated LDLc levels, 42.3% were on some lipid-lowering medications as treatment and 58.3% reached the target defined by the NCEP-ATP-III panel.

•In an analysis based on the PNAUM between 2014 and 2015, the use of statins in SUS primary care in the five Brazilian regions was assessed.^290^Among the 8803 respondents, the prevalence of statin use was 9.3%, and 81.4% of those users reported having dyslipidemia. Simvastatin was the most widely used statin (90.3%), followed by atorvastatin (4.7%) and rosuvastatin (1.9%).

•Regarding proper treatment and the recommended control targets in the secondary prevention setting, a study conducted in the Curitiba city healthcare system followed up 7066 individuals hospitalized due to AMI from 2008 to 2015. Of those, 1451 had at least one LDLc level measurement and outpatient follow-up data. The mean LDLc level found was 93.3mg/dL. Only 7.4% had LDLc < 50 mg/dL; 21.5% had LDLc between 50 and 70 mg/dL; 35.2% had LDLc between 70 and 100 mg/dL; and 35.5% (most prevalent group) had LDLc > 100 mg/dL. Among those with pre- and post-infarction LDLc measurements, a reduction in LDLc levels was observed, although smaller than the one recommended (from 128 to 92 mg/dL, with a 24.3% relative reduction in cholesterol levels), and only 19.3% of the individuals had a reduction > 50%.^291^

•In a cross-sectional analysis of the *Estudo Brasileiro de Diabetes* in the city of Campinas, 1030 participants (mean age, 58 years; men, 54%) had their lipid profiles assessed regarding the recommended targets met according to their cardiovascular risk groups (very high, high, and moderate). Mean LDLc levels were 105 ± 38, 109 ± 39, and 110 ± 37, respectively, and only 18.8% of the participants had LDLc levels considered adequate for their risk groups.^292^

•In the already mentioned study by Fonseca *et al.* in São Paulo state, only13.9% of the individuals considered as having dyslipidemia were on lipid-lowering drugs and met the LDLc serum levels < 100mg/dL.^286^

## Dyslipidemia and Subclinical Atherosclerosis

•Subclinical atherosclerosis, including markers such as coronary artery calcium score and carotid intima-media thickness, has been used as surrogate for atherosclerosis. Thus, its association with abnormal lipid profiles can be of epidemiological interest.^293^

•In a study with more than 3600 individuals, Generoso *et al* . have shown that HDLc was associated with coronary artery calcium even after adjustment for traditional cardiovascular risk factors. However, that association was no longer significant after adjustment for TG.^294^In addition, that study has evaluated HDLc subtypes and demonstrated they were not associated with coronary artery calcification once adjusted for total HDLc. Furthermore, the same group has shown the association between HDLc and carotid intima-media thickness, as well as the modification of that association by the presence of diabetes.^295^

•Laurinavicius *et al* . have studied the association between very high HDLc levels (over 90 mg/dL) and carotid intima-media thickness. Very high HDLc can characterize hyperalphalipoproteinemia, a dysfunctional HDLc condition. Despite prior evidence, those authors have not shown an association between such profile and carotid intima-media thickness.^296^

•In an analysis of TG-rich lipoproteins in the ELSA-Brasil study, Bittencourt *et al* . have demonstrated that those particles are associated with coronary artery calcification even after adjusting for significant risk factors.^297^

•In a study of octogenarian Brazilians, the authors have found that the association of high LDLc levels with coronary artery calcification weakens with age, whereas the association of low HDLc does not.^298^

•Collectively, those studies show a robust association of unfavorable lipid profile with subclinical atherosclerosis, corroborating findings of the association between dyslipidemia and CVD.

## Health Costs Attributable to Dyslipidemia

•The only publication on health costs attributable to dyslipidemia refers to estimated costs of hospitalizations due to coronary artery disease attributable to familial hypercholesterolemia.^298^That assessment used data from the Brazilian Hospital Information System of the SUS with 245 981 hospitalizations/year due to coronary artery disease. The prevalence of familial hypercholesterolemia was estimated based on international data at 0.4% and 0.73% in the United States and Denmark, respectively. In that scenario, the annual costs attributable to familial hypercholesterolemia were estimated at US$17 650 972.00 (for 0.4% prevalence) and US$31 448 466.00 (for 0.73% prevalence).^299^

## Future Directions

•Current data on the epidemiology of dyslipidemia in the contemporary Brazilian population are limited. Additional studies on its prevalence in the broad population as well as in specific high-risk groups, such as those with a lower socioeconomic status, are needed.

•The frequency of dyslipidemia screening, treatment, and control in Brazil, according to sex and age groups, needs to be assessed. In addition, the impact of dyslipidemia on the health system, including costs, has yet to be assessed comprehensively.

•Studies on the role of subclinical atherosclerosis markers in medical practice and on the effect of their use on the clinical treatment modification, as well as on the cost-effectiveness of their use, are required.

## CHAPTER 9 – DIABETES MELLITUS

### ICD-10 E10 to E14; ICD-10-CM E8 to E13

**Table:** Abbreviations used in Chapter 9

BINDER	BrazIliaN Type 1 & 2 DiabetEs Disease Registry
BMI	Body Mass Index
CI	Confidence Interval
CVD	Cardiovascular Diseases
COVID-19	Coronavirus disease 2019
DALYs	Disability-Adjusted Life Years
DATASUS	Brazilian Unified Health System Database
ELSA-Brasil	Longitudinal Study of Adult Health - Brazil
ERICA	Brazilian Study of Cardiovascular Risk in Adolescents (in Portuguese, *Estudo dos Riscos Cardiovasculares em Adolescentes* )
GBD	Global Burden of Disease
HbA1c	Glycosylated Hemoglobin
HR	Hazard Ratio
IBGE	Brazilian Institute of Geography and Statistics (in Portuguese, *Instituto Brasileiro de Geografia e Estatística* )
ICD-10	International Statistical Classification of Diseases and Related Health Problems, 10^th^Revision
IDF	International Diabetes Federation
LDL	Low-Density Lipoprotein
OR	Odds Ratio
PNS	National Health Survey (in Portuguese, *Pesquisa Nacional de Saúde* )
PR	Prevalence Ratio
SIH	Brazilian Hospital Information System (in Portuguese, *Sistema de Informações Hospitalares* )
SIVEP-Gripe	Brazilian Information System on Epidemiological Surveillance for Influenza (in Portuguese, *Sistema de Informação da Vigilância Epidemiológica da Gripe* )
SUS	Brazilian Unified Health System (in Portuguese, *Sistema Único de Saúde* )
UI	Uncertainty Interval
VIGITEL	Surveillance System of Risk and Protection Factors for Chronic Diseases by Telephone Survey (in Portuguese, *Sistema de Vigilância de Fatores de Risco e Proteção para Doenças Crônicas por Inquérito Telefônico* )

### Introduction

•Diabetes mellitus is a chronic and progressive disease, characterized by chronic hyperglycemia resulting from lower secretion of insulin and/or resistance to insulin, which in the long run can lead to microvascular (diabetic retinopathy, nephropathy, neuropathy) and macrovascular (coronary artery disease, cerebrovascular disease, and peripheral artery occlusive disease) complications. Prediabetes (reduced glucose tolerance and impaired fasting plasma glucose) is the condition in which there is hyperglycemia and, although the diagnostic criteria for diabetes are not met, the risks for future diabetes and CVD are increased.^300^

•In this chapter, diabetes mellitus is approached as a cardiovascular risk factor,^301^as it was in the previous version of this document,^4^because its presence, in association with smoking, systemic arterial hypertension, and dyslipidemia, increases by two to three times the risk of CVD.^302^

### Prevalence

•Recent data from the VIGITEL (2021) have shown that 9.1% (95% CI, 8.5-9.8%) of the adults report diagnosis of diabetes, and that figure is similar in women and men. The city of Belo Horizonte is the Brazilian capital with the highest prevalence of diabetes (11.3%, 95% CI, 9.0-13.5%) and the city of Rio Branco, the one with the lowest prevalence (6.4%, 95% CI, 4.5-8,3%). Considering educational level, the lower the schooling, the higher the prevalence of diabetes (< 8 years: 17.7%, 95% CI, 16-19.4%; >12 years: 5.1%, 95% CI, 4.3-5.9%). The higher the age, the higher the prevalence of diabetes (> 65 years: 28.4%, 95% CI, 26.5-30.2%).^252^

•According to data from the IDF, Brazil ranks sixth worldwide regarding the number of adults with diabetes (15.73 million, 95% CI, 14.04-17.43), with a prevalence of 10.5% (95% CI, 9.4-11.6), and 31.9% of them are unaware of their disease. The reduction observed in the number of individuals unaware of their condition as compared to previous data^4 , 303^may have resulted from the high rate of screening, as observed in the PNS from the IBGE, because, in 2013, 11.6% (95% CI, 11.1-12.1) of the adults had never had their blood glucose levels measured, and that percentage was reduced to 6.2% in 2019 (95% CI, 5.9-6.5).^304^Higher access to diagnosis has been observed in women (PR=1.16; 1.15-1.17), elderly individuals (PR=1.25; 1.22-1.28), and those with higher educational level (PR=1.17; 1.15-1.18), obesity (PR=1.06; 1.05-1.08), and hypertension (PR=1.12; 1.11-1.13). However, lower access to diagnosis has been observed in self-reported Black individuals (PR=0.97; 0.95-0.99), in individuals living in rural areas (PR=0.89; 0.87-0.90), and in those without private health insurance coverage (PR=0.85; 0.84-0.86).^305^

•The prevalence of prediabetes was 10.8% (CI, 6.9-12.8) or 17.77 million individuals (CI, 11.46-21.04).^303^These numbers are lower than those reported by the same source in 2019,^303^which differs from the previously reported constant increase in the prevalence of diabetes^306^and prediabetes.^307^It is worth noting that those numbers depend on the diagnostic method used, and they are smaller when the diagnosis is self-reported and higher in studies using confirmation with laboratory tests, especially those using oral glucose tolerance test^306^or HbA1c.^6^With any of those criteria, the prevalence was higher in women, in individuals over the age of 30 years, and in those with overweight or obesity. Higher educational level was associated with lower prevalence of diabetes. The Brazilian West-Central region had the highest prevalence of diabetes.^6^

•Data from the PNS 2019 have shown that the self-reported prevalence of diabetes was higher among individuals with lower educational level, as follows: 12.9% (illiteracy/ incomplete elementary education), 6.3% (complete elementary education/incomplete secondary education), 4.6% (complete secondary education/incomplete higher education), and 4.65% (complete higher education).^6^Similarly, individuals without private healthcare insurance had a higher prevalence of self-reported diabetes [PR=1.88; 95% CI, 1.22-2.89).^308^

•The PURE/South America Study has shown a 9% prevalence of diabetes (fasting plasma glucose > 126 mg/dL or personal history of diabetes) at the study’s baseline. Of 24 718 participants, 5661 were from Brazil, urban (65.1%) and rural areas of the São Paulo State; however, specific data from Brazil have not been reported.^309^

•Data from the ERICA Study, a large cross-sectional study conducted in a representative sample of Brazilian students aged 12-17 years (n=37 854), remain the most updated information on the prevalence of type 2 diabetes (3.3%, 95% CI, 2.9-3.7) and prediabetes (22.0%, 95% CI, 20.6-23.4).^310^Brazil ranks first regarding the number of cases, along with México.^303^

•Brazil ranks third worldwide regarding the number of cases of type 1 diabetes considering all ages (588 800 individuals) and the age range of up to 20 years (112 240 individuals). The difference in prevalence among countries, especially when considering age ranges, reflects younger populations and higher mortality in low-income countries (India, Algeria, Morocco, and Turkey), which reduces the number of adults with type 1 diabetes.^303^

### Incidence

•The Brazilian multicenter ELSA-Brazil study has reported a cumulative incidence of diabetes of 2.0/100 person-year (95% CI, 1.8-2.1), higher among the elderly [2.8%; 95% CI, 2.3-3.4], individuals with obesity (3.8%; 95% CI, 3.4-4.3), and those with lower educational levels (3.0%; 95% CI, 2.6-3.6).^311^

•The number of children and adolescents with type 1 diabetes has increased worldwide, approximately 3% per year, although with significant regional differences.^303 , 312 - 314^Brazil continues to rank third worldwide regarding the number of new cases of children and adolescents aged 0 to 19 years with type 1 diabetes (8.9 cases/1000/year).^303^The overall annual incidence of type 1 diabetes in the city of Bauru, São Paulo State, from 1986 to 2015, among children aged ≤ 14 years was 12.8 (95% CI, 11.2-14.4) per 100 000, ranging from 2.8 in 1987 to 25.6 in 2013, with no difference between sexes.^310^

### Mortality

#### Overall mortality attributable to diabetes

•Cardoso *et al.* , based on data from DATASUS from the Brazilian Health Ministry, have assessed early mortality of adults aged 30-69 years, from 2010 to 2017. The mean annual diabetes mortality rate was 28.1 (27.8-28.4) per 100 000 inhabitants from 2010 to 2012, and 27.0 (26.7-27.3) per 100 000 inhabitants from 2015 to 2017. The highest rates were observed in the Northeastern region [34.4 (33.9-35) per 100 000 inhabitants from 2015 to 2017], while the lowest, in the Southern region [20.4 (20.0-20.9) per 100 000 inhabitants from 2015 to 2017].^315^

•In addition, early mortality (30 to 69 years of age) has been assessed by Cousin *et al.* from 1990 to 2019 using estimates calculated by the GBD Study. Diabetes was the cause of early age-standardized mortality in 33.0 (32.0-34.2) per 100 000 inhabitants in 1990, in 26.8 (25.9-27.6) per 100 000 inhabitants in 2010, and in 24.8 (23.7-25.9) per 100 000 inhabitants in 2019, which represented a reduction of 1.0% from 1990 to 2010 and of 0.9% from 2010 to 2019.^316^

•The most recent data from the GBD Study are not available, and those of the last GBD Study version, 2019, have been reported in the Cardiovascular Statistics 2021.^4^

•The 10^th^edition of the IDF Diabetes Atlas, published in 2021, reported 214 175 deaths attributable to diabetes in Brazil of adults aged 20-79 years, a number slightly greater than that reported in the GBD Study 2019.^303^

•Arrais KR *et al.* have assessed the profile of hospitalizations and in-hospital mortality due to diabetes mellitus in the State of Piauí from 2015 to 2019. In that state, 18 361 hospitalizations due to diabetes mellitus were reported, 527 of which resulted in death, representing an in-hospital mortality rate of 2.87 per 100 admissions. The hospitalizations and deaths were more frequent among women, of mixed heritage, and older.^317^

#### Cardiovascular mortality attributable to diabetes

•The most recent data from the GBD Study are not available, and those of the last GBD Study version, 2019, have been reported in the Cardiovascular Statistics 2021.^4^

## Burden of disease

### Burden of disease attributable to diabetes

•Based on data from the GBD Study 2021, 2 740 000 (2 370 000-3 160 000) DALYs attributable to diabetes were estimated for Brazil in 2021, with a positive variation of 159.7% (95% UI, 149.2-172.1) from 1990 to 2021. However, the age-standardized DALY rate attributable to diabetes per 100 000 inhabitants in Brazil was estimated at 1075.2 (931.4-1239.0), a decrease of -2.7% (-7.1 to 2.1) from 1990 to 2021. These data reflect the effect of the Brazilian population increase and aging on the burden of disease attributable to diabetes in that period.^318^

### Burden of cardiovascular disease attributable to diabetes

•The most recent data from the GBD Study are not available, and those of the last GBD Study version, 2019, have been reported in the Cardiovascular Statistics 2021.^4^

## Impact on cardiovascular health

•Previous Brazilian studies, detailed in the previous version of this document, have shown that diabetes is related to different adverse cardiovascular outcomes and subclinical markers of atherosclerosis.^4 , 319 , 320^

•Dynkoski *et al.* , using data from the SIH of the DATASUS and analyzing admissions of elderly individuals due to conditions cared for in primary healthcare settings from 2008 to 2014 in Santa Catarina State, have shown that diabetes mellitus was the fifth cause of hospitalization (6.75%).^319^

•Mosenzon *et al.,* in the CAPTURE study, have estimated the prevalence of established CVD, assessed by use of medical records from primary and tertiary healthcare centers, in adults with type 2 diabetes from several countries (n = 9823), including Brazil. Of the 912 individuals assessed in Brazil, 43.9% (95% CI, 40.9-46.8) had CVD, a percentage higher than that estimated for the total sample of the 13 countries assessed [overall weighted CVD prevalence estimated at 34.8% (95% CI, 32.7-36.8)]. Heart failure had been reported in 113 patients [12.4 (95% CI, 10.4-14.4)], and Brazil had the second highest prevalence of all 13 countries assessed.^320^

## Knowledge, treatment, and control of diabetes

•The treatment of diabetes is based on three pillars: diet, physical exercise, and drugs (oral antidiabetic drugs and insulin). Adherence to treatment is an important determinant of metabolic control. Gomes and Negrato have reported minimal adherence to treatment in 48.0% of the patients, associated with a HbA1c level of 8.6% ± 1.9%.^321^Other studies have shown high access to medications, but low adherence to treatment [17.2% (95% CI, 14.6-20.1)].^322^Better adherence to treatment was associated with the following: younger age, lower BMI, presence of macrovascular complications, better occupational performance, and higher scores in the emotional domain of the short form health-related quality-of-life questionnaire - SF-36.^323^

•In 2018, Silva *et al.* conducted a household survey in 63 municipalities of Minas Gerais State, selected by convenience, aimed at assessing the profile of the use of medications in patients with diabetes in that state. Those authors showed that, among the 2619 individuals with diabetes assessed, the most frequently used drugs were metformin, losartan, glibenclamide, and simvastatin. Polypharmacy (use of five or more drugs) was identified in 56.5% of the respondents.^324^The prevalence of the use of oral antidiabetic drugs in Brazil increased from 77.4% (95% CI, 74.3-80.1) in 2012 to 85.2% (95% CI, 82.8-87.2) in 2018.^325^

•A retrospective cohort (n = 488 patients with diabetes) has shown that only 7.3% of the patients with diabetes in primary healthcare and 27.0% of those in tertiary healthcare had undergone assessment of at least 50% of the healthcare quality indicators (annual assessment of diabetic nephropathy, retinopathy, and neuropathy, lipid profile, nutritional assessment, and inquiry about tobacco use).^326^

•In 2022, using data from the SIH, DATASUS, Macedo Jr *et al* . conducted a descriptive epidemiological study with temporal analysis of the admissions of elderly individuals in Rondônia State from 2015 to 2019. Of the 23 844 admissions (11% of the total) in the period, diabetes was identified as the cause of admission in 267 (6.96%) in 2015 and in 349 (7.23%) in 2019, ranking second in both periods, after pneumonia.^327^

•The BINDER Registry, a prospective multicenter study conducted in 43 Brazilian cities, has aimed to assess the control of blood glucose of patients with diabetes in the real-world scenario. The 1142 participants were followed up for 2 years, starting in 2017. At baseline, the HbA1c was 7.1% (4.1-15.0%), and 396 (48.2%) patients had HbA1c ≤ 7.0%. Younger age (p = 0.014), lower educational level (p = 0.025), and public healthcare provision (p = 0.0058) were associated with elevated HbA1c. In the follow-up, there was no difference in HbA1c. In addition, the use of different oral antidiabetic drugs did not differ, except for a reduction in metformin use (p = 0.0044) and an increase in SGLT-2 inhibitors use (p < 0.001).^328^

•A cross-sectional analysis of the population with diabetes from the ELSA-Brasil has reported on the metabolic control and risk factors of that population. Of the 2062 individuals with diabetes, 1364 (66.1%) had HbA1c < 7.0%, 1596 (77.4%) had proper blood pressure control, and 1086 (52.7%) had target LDL-cholesterol levels (<100 mg/dL or <70 mg/dL if high cardiovascular risk). Women (PR=1.13; 95% CI, 1.07-1.20), individuals over the age of 74 years (PR=1.20; 95% CI, 1.08-1.34), and those with a higher per capita income (PR=1.26; 95% CI, 1.10-1.45) were more likely to have better glycemic control. Self-reported Black individuals (PR=0.91; 95% CI, 0.83-1.00) and those with longer-lasting diabetes (PR=0.43; 95% CI, 0.39-0.47) were less likely. Women (PR=1.05; 95% CI, 1.00-1.11) and individuals with private healthcare insurance (PR=1.15; 95% CI, 1.07-1.23) were more likely to reach two or more goals, while self-reported Black individuals (PR=0.86; 95% CI, 0.79-0.94) and those with longer-lasting diabetes (PR=0.68; 95% CI, 0.63-0.73) were less likely.^329^

•In 2023, Machline-Carrion *et al.* conducted a cross-sectional study analyzing routinely recorded medical data from 2 133 900 individuals in primary healthcare, mean age of 66.2 ± 14.6 years, 49.5% men, 29.6% White, in Brazil, from 2016 to 2021. Of that sample, 6.7% (2346) had a record of statin prescription, which was more frequent among those over the age of 60 years (OR 1.32 [95% CI, 1.19-1.47), living in the Southeastern region (OR 4.53 [95% CI, 3.66-5.60]), with history of acute myocardial infarction (OR 4.53 [95% CI, 3.66-5.60]) and diabetes (OR 1.50 [95% CI, 1.37-1.64]).^89^

•In 2022, Malta *et al.* assessed healthcare indicators of individuals with diabetes from the 2013 PNS (n = 60 202) and compared them to those obtained from the same database in 2019 (n = 88 531). There was an increase in the use of medications (from 80.2% to 88.8%) and in the number of individuals on medical treatment (from 73.2% to 79.1%), although the percentages of individuals undergoing screening for diabetic retinopathy (35.3% *vs.* 36.7%) and foot assessment (29.1% *vs.* 31.7%) remained stable. Hospitalizations due to diabetes-related complications were less frequent in women in the period [13.1 (11.5-15.0) *vs.* 16.5 (14.3-19.1); PR 0.80 (95% CI, 0.65-0.97)]. In addition, in 2019, the indicators were worse for men, younger individuals, Black individuals, and those with lower socioeconomic and educational levels. Regarding regional differences, the Southeastern, Southern, and West-Central regions had a higher proportion of individuals receiving medication from the Popular Drugstore as compared to the Northern region (56.4%, 59.1%, 56.4% vs. 35.6%, respectively). The Southeastern region had the highest proportion of individuals undergoing diabetic retinopathy screening (40.8%) and the lowest number of hospitalizations (12.1%).^304^

## Risk factors and prevention

•A meta-analysis from 151 studies on the prevalence of overweight and obesity among Brazilian adolescents has shown an increase in the prevalence of overweight of 8.2% (95% CI, 7.7-8.7) up to 2000, of 18.9% (95% CI, 14.7-23.2) from 2000 to 2009, and of 25.1% (95% CI, 23.4-26.8) from 2010 onwards, a pattern similar to that of the prevalence of obesity. The Southeastern and Southern regions had higher prevalence of overweight and obesity.^330^

•Of the 37 892 adolescents of the ERICA Study, overweight was observed in 17.2%, obesity in 5.6%, and severe obesity in 1.3%, with an increased chance of adverse cardiometabolic outcomes according to higher BMI, such as higher fasting blood glucose [RP 5.30 (95% CI, 1.94-14.50)] and HbA1c (2.04; 95% CI, 1.29-3.25).^331^

•At the ELSA-Brasil baseline, the analysis of 14 912 Brazilian civil servants has shown higher prevalence of diabetes among individuals with BMI of 25-29.9 kg/m^2^(18.9%; 95% CI, 18.0-19.9%) and above 30 kg/m^2^(32.1%; 95% CI, 30.6-33.6%) as compared to those with BMI ≤ 24.9 kg/m^2^(11.7; 95% CI, 10.9-12.6%).^281^Considering leisure-time physical activity, the ELSA-Brasil has shown a smaller likelihood of diabetes in active women and men as compared to inactive ones.^332^

•Based on data from the second wave of the ELSA-Brasil (2012-2014), 10 047 Brazilian civil servants were assessed regarding the association between body fat distribution and the occurrence of CVD and its risk factors. The multivariate analysis showed that the smaller ratio between arm and leg fat/trunk fat protected against the presence of diabetes in women (0.04, 95% CI, 0.01-0.19), but not in men (1.03; 95% CI, 0.37-2.86).^333^

•Data from the PNS 2019 with 86 678 adults have shown that television watching for more than 3 hours per day increased the likelihood of developing diabetes as compared to less than 3 hours per day (3-6 hours/day *vs.* reference OR 1.26; 95% CI, 1.12-1.42; >6 hours/day *vs.* reference OR 1.80; 95% CI, 1.54-2.11; adjustments to sex, age range, ethnicity, educational level, alcohol consumption, smoking, soda and fruit consumption, and leisure-time physical activity). However, there was no association between the use of other screens (computers, tablets, and cell phones) and the likelihood of developing diabetes.^334^There was a dose-response relationship between television watching and the chance of developing diabetes.^335^

•Teló G *et al* ., in a cross-sectional study with 37 854 adolescents, have shown a higher likelihood of type 2 diabetes in those with obesity (OR 1.59; 95% CI, 1.20-2.11) and increased waist circumference (OR 1.51; 95% CI, 1.13-2.01), with no association with physical inactivity (< 60 min/day).^310^

•In a sample of 9909 participants of the ELSA-Brasil Study, food frequency questionnaires were used to calculate the dietary inflammatory index. In a logistic regression adjusted to sociodemographic and behavioral factors and BMI, that index was a predictor of greater weight gain (third tertile vs. 1: OR Z 1.30; 95% CI, 1.08-1.55) and incidence of diabetes (third tertile vs. 1: HR Z 1.26; 95% CI, 1.04-1.52).^336^

## Costs

•Ferrari G *et al.* have estimated the direct costs of healthcare related to noncommunicable chronic diseases attributable to obesity in Brazil in 2019 and have shown a total cost of diabetes of US$ 44.2 million (from a total of US$ 2967.6 million). Considering the attributable population fraction of 63.1% (40.3-86.4), the cost attributable to diabetes would be US$ 27.9 million (17.8-38.2).^337^

•In 2022, Leal *et al.* used data from the GBD Study 2019 and DATASUS to estimate the costs with the treatment of diabetes and other noncommunicable chronic diseases attributable to the consumption of sweetened sugar beverages in Brazil. The results showed a total cost of US$ 14 116 240.55 with hospitalizations from noncommunicable chronic diseases attributable to the consumption of sweetened sugar beverages, US$ 72 645.40 for women and US$ 80 345.36 for men with diabetes. The costs of outpatient care for diabetes attributable to the consumption of sweetened sugar beverages were US$ 4645.79 for women and US$ 4641.06 for men with diabetes. Data according to age range can be found in the original publication.^338^

•In 2022, Pereda *et al.* used the cost-of-illness approach to calculate the total economic burden related to diabetes in Brazil in 2016, which was estimated at up to US$ 2.15 billion, of which US$ 1.52 billion (70.6%) were indirect costs related to premature deaths, absenteeism, and early retirement. The indirect costs related to premature deaths were US$ 1.18 billion (77.9% of indirect costs). The total direct costs related to diabetes were US$ 633 million, US$ 232.8 million with hospitalizations and US$ 86 million with outpatient expenses (95% covered by the SUS). The Popular Drugstore represented US$ 304.2 million, while the out-of-pocket expenses were US$ 10.0 million. The total cost per patient diagnosed in Brazil, considering the prevalence of 6.4% (data from 2013), was US$ 223.54 (US$ 65.72 of direct costs and US$ 157.81 of indirect costs).^339^

•In 2019, the expenditures related to diabetes in adults (20-79 years) worldwide were US$ 966 billion, and Brazil ranks third with expenditures of US$ 42.9 billion.^303^

## Impact of the COVID-19 pandemic on diabetes mellitus

•Diabetes is a risk factor for worse outcomes related to COVID-19. Patients with COVID-19 and diabetes are at a higher risk for hospitalizations and death than those without diabetes. Poor glycemic control in the presence of COVID-19 determines worse outcomes.

•Niquini *et al.* , in 2020, used the database of the SIVEP-Gripe to compare the numbers of hospitalization due to acute respiratory distress syndrome, which were 39 349 in 2019 (14.7% due to influenza) and 94 807 in 2020 (33.7% due to COVID-19). Of those, 16.2% and 24.7% had diabetes, respectively.^340^

•In 2021, Nunes *et al.* used, in addition to the SIVEP-Gripe, data from the linkage performed by the Bahia State Epidemiological Surveillance and the Laboratory Environment Management System (GAL) and showed that most of the 7286 deaths from COVID-19 observed up to November 2020 occurred in men and 43.6% in individuals with diabetes.^341^

•A cross-sectional study has assessed 21 942 Brazilians who had been hospitalized due to COVID-19 by mid-2020 using data from the SIVEP-Gripe. In adults (age ≤ 60 years), obesity in combination with diabetes and/or CVD was associated with a higher prevalence of invasive (PR 3.76; 95% CI, 2.82-5.01) and noninvasive (PR 2.06; 1.58-2.69) mechanical ventilation, intensive care unit admission (PR 1.60; 1.40-1.83), and death (PR 1.79; 1.45-2.21) as compared to patients without those conditions.^342^

•Prado *et al.,* in 2021, assessed retrospectively all cases of COVID-19 reported up to September 2020 (n = 57 700) and found mortality of 61.8/100 000 inhabitants. The following risk factors for death were identified: male sex (HR=1.48; 95% CI, 1.25-1.76), elderly (≥60 years, HR=10,64; 95% CI, 8.84-12.81), and multiple comorbidities (HR=2.23; 95% CI, 1.77-2.81), which included diabetes mellitus.^343^

•In 2021, Pietre *et al.* reported on data obtained from that same database up to August 2020 (n=181 964, 96 567 deaths), emphasizing the higher risk of mortality from severe acute respiratory syndrome among younger patients with diabetes and self-reported Black individuals (aOR 5.58; 95% CI, 4.97-6.25; p<0.0001) and among those with obesity or hematological diseases (aOR 21.09; 95% CI, 13.64-32.06) as compared to their controls.^344^

•In the cross-sectional study by Garces *et al.* , 2022, performed with data from 397 600 individuals hospitalized between 2020 and 2021 (SIVEP-Gripe), 32.0% of them died. The prevalence of death among individuals with diabetes was 40.8% (n = 41 776), with an adjusted prevalence ratio of the association between diabetes and death from COVID-19 of 1.15 (IC 95% 1.14-1.16), representing a death prevalence 15% higher among Brazilians with diabetes hospitalized from COVID-19.^345^

•Considering the age range under 20 years, Oliveira *et al.* have shown that, of 21 591 children and adolescents hospitalized due to COVID-19, 379 (1.8%) had diabetes, among whom the following was more frequent as compared to those without diabetes: admission to the intensive care unit (46.6% *vs* . 26.0%), invasive ventilation (16.9% vs. 10.3%), and higher mortality (15.0% vs. 7.6%; HR = 2.0, 95% CI, 1.58-2.66). Regarding these individuals, living in poorer regions (Northeastern region: HR, 2.17, 95% CI, 1.18-4.01; Northern region: HR 4.0, 95% CI, 1.79-8.94), oxygen saturation < 95% on admission (HR 2.97, 95% CI, 1.64-5.36), kidney disease (HR 3.39, 95% CI, 1.42-8.09), and obesity (HR 3.77, 95% CI, 1.83-7.76) were associated with mortality.^346^

•In 2021, Sardinha *et al.* performed a cross-sectional study with native Brazilians, using data from DATASUS and showed that, up to August 2020, there were 1207 cases and 470 deaths due to COVID-19 in that population. Although diabetes was one of the most prevalent comorbidities in that population (18.97%), it did not associate with higher mortality.^347^

•In 2022, the cross-sectional study by Andrade *et al.* , based on the analysis of reports on severe acute respiratory syndrome due to COVID-19 (DATASUS) from 2020 to 2021, compared individuals hospitalized with (111 046) and without (273 759) diabetes. Those authors showed that individuals with diabetes were more likely to be admitted to the intensive care unit (43.7% *vs.* 37.3%) and to have higher mortality (44.6% *vs.* 35.7%).^348^

•Foppa *et al.* have published a cohort of 289 patients with type 1 diabetes, 40 ± 12 years, 49.5% women, assessed in 2019 and 2020 when receiving care. The number of consultations with a multiprofessional team was smaller during the pandemic, even considering the in-person and tele-consultations (4.0; 3.0-5.0 *vs.* 2.0; 1.0-3.0), as were the percentage of patients with target HbA1c level (26% *vs.* 1%) and the number of patients undergoing diabetic retinopathy screening (63.9% *vs.* 30.9%).^349^

•A cohort study of patients with COVID-19 admitted to 25 Brazilian hospitals from March to September 2020 has aimed to assess the risk factors for in-hospital mortality. Of 2054 patients, 52.6% were of the male sex and in-hospital mortality was 22.0%, while mortality for those admitted to the intensive care unit was 47.6% and for those with diabetes, 29.2%. On multivariate analysis, however, diabetes did not associate with higher mortality.^11^

•Data from the VIGITEL have shown that, from 2019 to 2022, despite the reduction in the prevalence of soda consumption (15.1%, 95% CI, 14.3-15.9% in 2019; 14.0%, 95% CI, 12.9%-15.3% in 2021-2022), there was an increase in sedentary lifestyle (62.7%, 95% CI, 61.8-63.6% in 2019; 66.0%, 95% CI, 64.6%-67.4% in 2021-2022), and a reduction in leisure-time physical activity (39.0%, 95% CI, 38.1-39.9% in 2019; 36.7%, 95% CI, 35.3%-38.2% in 2021-2022). This might have influenced the increase in the prevalence of obesity and self-reported diabetes (7.5%, 95% CI, 7.0-7.9% in 2019, and 9.1%, 95% CI, 8.5-9.8% in 2021) in the period.^252^

## Future Directions

•Studies on the incidence of type 1 and type 2 diabetes with national representativity, aimed at social and behavioral determinants are required.

•Considering the SUS coverage and the possibility of reaching many patients with type 1 and type 2 diabetes, studies focused on assessing the efficacy and effectiveness of the care provided to these patients in Brazil are required.

•Considering the several publications on the increase of the overweight and obesity incidence in the Brazilian population in all age ranges, mostly in the lower socioeconomic levels, efficient public policies to prevent obesity should be prioritized to reduce new cases of diabetes and its complications. Some examples are: 1. Taxation of high caloric foods; 2. Mandatory labeling of food products; 3. Creation of programs to prevent and treat obesity in communities, rescuing individuals predisposed to diabetes by using simple tools (questionnaires); 4. Training of multiprofessional teams to engage in lifestyle-change programs to prevent and treat diabetes; 5. Integration of physical education professionals into those programs.

•Artificial intelligence has been used in several aspects of diabetes: identification of groups of patients at risk for chronic complications based on the assessment of the behavior of the HbA1c variation; identification of several clinical variables that predict the HbA1c response in the short and long run after beginning treatment; identification of the medicamentous treatment to be instituted according to the patient’s comorbidities and the association with the development of complications; identification of groups of patients with diabetes at different stages of disease progression; and predictive equations for hospital admission. These new stratifications can help guide the treatment, representing a first step to precision medicine in diabetes. If properly used, it may provide important information to help disease prevention and management.

•Inequalities in access and in use of preventive, diagnostic, and therapeutic measures related to social, demographic, economic, gender, and skin-color questions have been made clear in studies with the Brazilian population with diabetes. Data should be compiled and those missing should be actively sought so that public policies aimed at reducing those differences can be built.

## CHAPTER 10 – SMOKING AND TOBACCO USE

### Smoking and the consequences for cardiovascular diseases, Brazil and Federative Units, 1990 to 2022

### ICD-10: Z.72.0

**See Tables 10-1 through 10-6 and Charts 10-1 through 10-2**

Abbreviations Used in Chapter 10

**Table:** 

CI	Confidence Interval
Covitel	Telephone Survey for Surveillance of Non-Communicable Chronic Diseases During the Pandemic (in Portuguese, *Inquérito Telefônico de Fatores de Risco para Doenças Crônicas não Transmissíveis em Tempos de Pandemia* )
CVD	Cardiovascular Diseases
e-cigarette	Electronic cigarette
FU	Federative Unit
GBD	Global Burden of Disease
GDP	Gross Domestic Product
PeNSE	National Survey of Schoolchildren Health (in Portuguese: *Pesquisa Nacional de Saúde do Escolar* )
PNS	Brazilian National Health Survey (in Portuguese, *Pesquisa Nacional de Saúde* )
SGM	Sexual and Gender Minorities
VIGITEL	Telephone Survey for Surveillance of Non-Communicable Chronic Diseases (in Portuguese, *Vigilância de Fatores de Risco e Proteção para Doenças Crônicas por Inquérito Telefônico* )
WHO	World Health Organization

### Introduction

•Tobacco use is one of Brazil’s leading causes of preventable death.^3^Smoking is a major risk factor for CVD.^350^The negative impact of tobacco on health results from both the direct consumption of various forms of tobacco products (smoked, inhaled or chewed) and the exposure to second-hand smoke.^351^Recently there have been some changes in the tobacco consumption habits: despite a decline in commercial use of cigarette smoking, other forms of tobacco use are becoming increasingly common, such as e-cigarettes and hookah.^352^Furthermore, there was an impact of the COVID-19 pandemic on smoking habits and consumption. These two more recent aspects were included and/or updated.

•In this chapter, tobacco use and its consequences for CVD are described. The prevalence of smoking will be addressed according to the main population surveys in Brazil, such as the following: the updated studies for the PNS 2019, containing estimates for the population aged 18 years and over, as previously described;^4^the PeNSE 2019, containing estimates for adolescents aged 13–17 years;^353^data from the VIGITEL 2021, a population-based cross-sectional survey conducted by the Brazilian Ministry of Health in Brazilian state capitals, and compared to its time series; and the Covitel study 2022, a countrywide cross-sectional telephone-based survey carried out during the first trimester of 2022 in Brazil.^252 , 352^These are the most recent nationwide surveys for Brazil. For definitions herein used and the relation of tobacco to CVD, refer to the previous version of this document (Cardiovascular Statistics – Brazil 2021).^2 , 4^

•The main indicators of smoking among adults (use of tobacco, passive smoking, and e-cigarettes) and adolescents (experimentation with tobacco, use in the last month, use of other tobacco products) will be presented and updated. For mortality rates and absolute number of deaths attributable to tobacco in Brazil and its 27 FUs from 1990 to 2019 refer to the previous version of this document (Cardiovascular Statistics – Brazil 2021),^2 , 4^as the most recent estimates of the GBD had not been updated until the completion of this document.

### Prevalence of tobacco use among adolescents

•According to PeNSE 2019, among adolescents in the ninth grade of school aged 13-17 years, in 2019, the prevalence of current smoking or having smoked in the 30 days before the survey was 6.8% (95% CI, 6.3-7.3%), with a higher prevalence among males, 7.1% (95% CI, 6.6-7.6%), than females, 6.5% (95% CI, 5.8-7.2%) **( Table 10-1 )** .^353^

**Table 10-1:** – Percentage of students aged 13-17 years who smoked in the 30 days before the survey, by sex and type of school, in Brazil, its major regions and Federative Units

Major regions and Federative Units	Total			Sex						Type of school					
				Male			Female			Public			Private		
	Total	95% CI		Total	95% CI		Total	95% CI		Total	95% CI		Total	95% CI	
		Lower limit	Upper limit		Lower limit	Upper limit		Lower limit	Upper limit		Lower limit	Upper limit		Lower limit	Upper limit
Brazil	6.8	6.3	7.3	7.1	6.6	7.6	6.5	5.8	7.2	7.2	6.6	7.8	4.4	4.1	4.8
North	7.2	6.2	8.1	8.3	7.0	9.6	6.2	5.1	7.2	7.4	6.5	8.4	3.4	2.7	4.0
Rondônia	6.1	5.1	7.2	6.2	5.1	7.4	6.0	4.4	7.7	6.4	5.2	7.5	2.7	1.9	3.4
Acre	10.9	9.0	12.8	13.3	10.3	16.2	8.6	6.8	10.3	11.2	9.2	13.2	3.5	1.6	5.5
Amazonas	7.7	6.1	9.3	8.9	6.6	11.3	6.5	4.8	8.3	8.0	6.3	9.6	3.1	1.3	4.9
Roraima	9.2	7.8	10.6	11.2	9.3	13.0	7.1	5.5	8.7	9.6	8.1	11.1	2.4	1.0	3.7
Pará	6.6	4.9	8.4	7.9	5.2	10.5	5.6	3.7	7.6	6.9	5.1	8.8	3.3	2.3	4.2
Amapá	7.1	5.9	8.3	6.9	5.7	8.2	7.3	5.6	9.0	7.4	6.1	8.7	4.2	3.3	5.1
Tocantins	6.8	5.1	8.5	7.7	5.1	10.3	6.0	4.2	7.7	6.9	5.1	8.8	5.2	3.9	6.5
Northeast	4.7	4.3	5.2	5.3	4.7	5.8	4.2	3.7	4.8	5.0	4.5	5.5	2.7	2.4	3.0
Maranhão	6.0	4.7	7.4	7.5	5.7	9.2	4.5	2.6	6.4	6.3	4.8	7.7	2.9	2.3	3.6
Piauí	4.3	3.2	5.3	5.1	3.7	6.5	3.5	2.2	4.7	4.5	3.3	5.7	2.6	1.8	3.4
Ceará	5.7	4.3	7.1	6.0	4.0	8.1	5.4	4.1	6.7	6.2	4.5	7.9	2.5	1.9	3.2
Rio Grande do Norte	4.5	3.5	5.5	4.7	3.4	6.1	4.3	3.1	5.5	5.0	3.9	6.2	1.9	1.3	2.4
Paraíba	5.9	4.9	6.8	6.5	5.0	8.0	5.3	4.0	6.5	6.4	5.3	7.5	3.3	2.4	4.3
Pernambuco	4.4	3.5	5.4	4.3	2.9	5.7	4.5	3.1	5.9	4.6	3.5	5.7	3.4	2.6	4.2
Alagoas	5.0	3.8	6.2	6.4	4.4	8.4	3.5	2.4	4.7	5.4	4.0	6.9	2.4	1.7	3.2
Sergipe	3.2	2.4	4.1	3.6	2.5	4.8	2.9	1.8	4.0	3.5	2.4	4.5	2.3	1.7	2.9
Bahia	3.7	2.6	4.7	3.9	2.8	5.0	3.4	2.1	4.8	3.8	2.6	5.0	2.4	1.7	3.1
Southeast	7.6	6.4	8.7	7.6	6.5	8.6	7.6	5.8	9.4	8.2	6.8	9.6	5.0	4.3	5.7
Minas Gerais	8.2	6.7	9.6	8.3	6.5	10.1	8.0	5.9	10.2	8.4	6.8	10.0	6.7	5.2	8.2
Espírito Santo	6.8	5.5	8.0	8.2	6.3	10.0	5.4	4.1	6.8	7.0	5.6	8.4	4.9	3.8	6.0
Rio de Janeiro	6.6	5.5	7.7	7.0	5.6	8.4	6.2	4.9	7.6	7.6	6.1	9.1	3.9	3.1	4.7
São Paulo	7.7	5.7	9.8	7.3	5.7	9.0	8.1	4.9	11.3	8.4	5.8	10.9	5.0	3.8	6.1
South	8.0	7.1	8.9	7.8	6.6	9.0	8.3	6.9	9.6	8.3	7.3	9.4	5.9	5.0	6.9
Paraná	8.9	7.3	10.6	8.2	6.0	10.3	9.8	7.6	11.9	9.4	7.5	11.3	6.3	4.8	7.7
Santa Catarina	8.5	6.8	10.2	9.0	6.7	11.3	8.1	5.9	10.2	9.0	7.1	10.9	5.1	3.3	6.8
Rio Grande do Sul	6.8	5.4	8.1	6.6	5.0	8.1	7.0	4.5	9.4	6.8	5.3	8.4	6.2	4.6	7.9
West-Central	7.7	6.9	8.5	8.6	7.6	9.7	6.8	6.0	7.7	8.1	7.2	9.0	5.3	4.7	6.0
Mato Grosso do Sul	9.7	8.5	10.9	10.3	8.6	12.1	9.1	7.8	10.3	10.1	8.8	11.5	6.2	4.8	7.6
Mato Grosso	8.4	6.7	10.1	9.8	7.5	12.1	7.0	4.8	9.2	8.7	6.8	10.7	5.0	3.7	6.2
Goiás	7.4	6.0	8.7	8.4	6.6	10.2	6.3	5.2	7.5	7.6	6.1	9.2	6.1	4.9	7.2
Distrito Federal	6.0	4.5	7.4	6.1	4.4	7.9	5.8	3.7	8.0	6.5	4.7	8.4	4.2	3.1	5.3

Source: PeNSE 2019. ^353^

•The prevalence of the first use of cigarettes before the age of 13 years was 13.1% (95% CI, 11.7-14.5%), with lower levels among males, 11.7% (95% CI, 10.2-13.1%), than females, 14.4% (95% CI, 12.6-16.2%).^353^

•Among adolescents aged 13-17 years, 22.6% (95% CI, 21.7-23.4%) had tried cigarettes in their lifetime. Experimentation ranged from 16.6% in the Northeastern region to 28.5% in the Southern region.^353^

•Among adolescents, there was a 2% increase in the prevalence of ninth graders who had ever tried cigarettes, from 19% (95% CI, 18.1-19.9%) in 2015 to 21% (95% CI, 19.5%-22.5%) in 2019.^353^

•In 2019, the prevalence of e-cigarette use any time in life for adolescents aged 13-17 years was 16.8% (95% CI, 16.2-17.4%) with a higher proportion of males (19.1%; 95% CI, 18.3-19.9%) than females (14.6%; 95% CI, 13.9%-15.3%) **( Table 10-4 )** .^353^

**Table 10-4:** – Percentage of students aged 13-17 years who had ever tried e-cigarettes, by sex and type of school, according to age groups and Brazilian major regions

	Total			Sex						Type of school					
				Male			Female			Public			Private		
	Total	95% CI		Total	95% CI		Total	95% CI		Total	95% CI		Total	95% CI	
		Lower limit	Upper limit		Lower limit	Upper limit		Lower limit	Upper limit		Lower limit	Upper limit		Lower limit	Upper limit
**13 to 17 years**															
Brazil	16.8	16.2	17.4	19.1	18.3	19.9	14.6	13.9	15.3	16.6	15.9	17.3	18.0	17.3	18.8
North	12.3	11.1	13.4	14.9	13.2	16.5	10.0	8.7	11.2	11.9	10.6	13.2	16.6	15.3	17.9
Northeast	10.8	10.0	11.5	12.7	11.6	13.8	8.9	8.1	9.7	10.3	9.5	11.1	13.8	13.0	14.7
Southeast	19.6	18.4	20.8	21.6	19.9	23.2	17.6	16.2	19.0	19.9	18.5	21.3	17.9	16.6	19.3
South	21.0	19.3	22.7	23.2	21.1	25.4	18.7	16.9	20.6	20.7	18.8	22.6	23.2	21.4	24.9
West-Central	23.7	22.6	24.9	27.4	25.8	28.9	20.2	18.8	21.7	23.6	22.3	25.0	24.3	22.7	25.9
**13 to 15 years**															
Brazil	13.6	13.0	14.2	14.8	13.9	15.7	12.5	11.7	13.2	13.5	12.8	14.2	14.2	13.4	15.0
North	10.5	9.3	11.8	12.4	10.5	14.4	8.8	7.6	10.1	10.3	9.0	11.7	12.9	11.6	14.1
Northeast	8.5	7.7	9.3	9.9	8.9	11.0	7.2	6.3	8.0	8.2	7.3	9.1	10.2	9.4	10.9
Southeast	15.7	14.5	16.9	16.4	14.5	18.2	15.0	13.5	16.5	16.0	14.5	17.4	14.6	13.2	16.0
South	16.6	15.0	18.3	17.6	15.6	19.5	15.7	13.6	17.9	16.4	14.5	18.3	17.8	16.0	19.6
West-Central	20.4	19.0	21.7	22.1	20.3	23.9	18.8	17.1	20.4	20.5	18.9	22.0	19.9	18.2	21.6
**16 to 17 years**															
Brazil	22.7	21.7	23.7	27.0	25.7	28.3	18.5	17.3	19.8	22.1	21.0	23.2	26.9	25.1	28.6
North	15.3	13.5	17.2	19.0	16.4	21.7	12.0	10.0	14.0	14.7	12.7	16.7	24.9	22.3	27.6
Northeast	14.9	13.8	16.1	17.7	15.8	19.7	12.2	10.8	13.5	14.0	12.7	15.3	23.1	21.1	25.1
Southeast	26.7	24.5	28.8	31.7	29.3	34.1	22.0	19.2	24.8	26.9	24.4	29.3	25.7	22.5	28.9
South	28.9	25.9	31.8	32.9	29.0	36.8	24.4	21.3	27.6	28.2	24.9	31.5	33.8	30.7	36.9
West-Central	30.1	28.1	32.1	37.1	34.4	39.8	23.1	20.8	25.4	29.5	27.3	31.7	34.3	31.3	37.3

Source: PeNSE 2019. ^353^

•Hookah use any time in life by adolescents aged 13-17 years was 29.6%, (95% CI, 28.6-30.7%), with no difference between females (29.6%; 95% CI, 28.2-31%) and males (29.7%; 95% CI, 28.6-30.8%).^353^

•The proportion of tobacco products used 30 days before the survey by adolescents aged 13-17 years was 7.8% (95% CI, 7.3-8.3%) for hookah and 2.8% (95% CI, 2.6-3.0%) for e-cigarettes.^353^

•An increased risk of becoming regular tobacco user was associated with alcohol consumption in the last 30 days for both sexes; in addition, for males, that risk was also increased by involvement in physical fighting in the last 12 months.^354^

### Prevalence of tobacco use among adults

•According to the VIGITEL 2021, the prevalence of smoking among adults (≥ 18 years) was 9.1% (95% CI, 8.2-9.9%), higher in males, 11.8% (95% CI, 10.3-13.3%), than in females, 6.7% (95% CI, 5.8-7.7%). The prevalence was lower among young adults (≤ 34 years) and those ≥ 65 years **( Table 10-2 and Charts 10-1 and 10-2)** .^252^

**Table 10-2:** – Prevalence of current smokers aged ≥ 18 years, by sex, according to age and years of schooling

	Total			Sex					
				Male			Female		
	Total	95% CI		Total	95% CI		Total	95% CI	
		Lower limit	Upper limit		Lower limit	Upper limit		Lower limit	Upper limit
**Total (%)**	9.1	8.2	9.9	11.8	10.3	13.3	6.7	5.8	7.7
**Age (years)**									
18-24	6.4	4.4	8.4	7.2	4.8	9.6	5.4	2.1	8.8
25-34	7.3	5.6	9.0	11.9	8.7	15.0	3.3	1.7	4.8
35-44	11.6	9.0	14.3	17.7	12.7	22.6	6.9	4.4	9.4
45-54	10.1	8.1	12.1	11.6	8.3	14.9	8.8	6.4	11.2
55-64	11.5	9.7	13.4	13.1	9.9	16.3	10.3	8.2	12.4
≥ 65	7.4	6.2	8.6	7.3	5.2	9.4	7.5	6.1	9.0
**Years of schooling**									
0-8	12.9	11.0	14.8	15.7	12.6	18.9	10.3	8.0	12.6
9-11	8.1	6.8	9.5	10.5	8.1	12.8	6.0	4.7	7.4
≥ 12	7.2	5.9	8.4	10.3	8.0	12.6	4.8	3.5	6.1

Source: VIGITEL Brazil 2021. ^252^

•The prevalence was highest in the age group 35-44 years [11.6% (95% CI, 9.0-14.3%)] as compared to the following age groups: 18-24 years [6.4% (95% CI, 4.4-8.4%)]; 25-34 years [7.3% (95% CI, 5.6-9.0%)]; and ≥65 years [7.4% (95% CI 6.2-8.6%)] **( Table 10-2 )** .^252^

•The prevalence was lower in populations with higher educational levels, being 7.2% (95% CI, 5.9-8.4%) among those with 12 years or more of schooling, 8.1% (95% CI, 6.8-9.5%) among those with 9-11 years of schooling, and 12.9% (95% CI, 11.0-14.8%) among those with 0-8 years of schooling.^252^

•Among SGM, the reported prevalence of any tobacco product use was 44.7%. Hookahs use was ∼8 times higher for SGM than for non-SGM. SGM tobacco users tend to be younger, more educated, and less likely to have stable relationships than non-SGM tobacco users or tobacco users overall.^355 , 356^

•From 2013 to 2019, there was a 52% increase in the prevalence of former smokers, from 17.5% (95% CI, 16.9-18.0) to 26.6% (95% CI, 26.1-27.2). However, in 2019, 46.6% (95% CI, 45.0-48.3) of smokers reported attempting to quit smoking, contrasted with 51.1% (95% CI, 49.3-52.9) in 2013, which represents an 8.8% decrease over the same period.

### Prevalence trend

•According to VIGITEL 2021 data, there was a 43.8% decrease in the prevalence of smoking in the adult population (age ≥18 years) for both sexes, from 2006 to 2021. The prevalence of smoking was 16.2% in 2006, with a progressive decline to 9.1% in 2021.^252^

•From 2015 to 2019, VIGITEL data show that the use of cigarettes among adolescents remained stable. In 2015, 6.6% (95% CI, 5.8-7.3%) of the adolescents reported having used cigarettes in the last 30 days, whereas, in 2019, this prevalence was 6.8% (95% CI, 6.3-7.3%).^353^The use of other tobacco products in the last 30 days among adolescents increased from 2015 (7.2%; 95% CI, 6.1-8.2%) to 2019 (12.4%; 95% CI, 11.8-12.9%).^353^

### Second-hand smoke

•According to VIGITEL 2021 data, the prevalence of second-hand smoke at home was 6.9% (95% CI, 6.0-7.9%), higher among males (7.6%; 95% CI, 6.1-9.2%) than among females (6.4%; 95% CI, 5.1-7.6%) **( Table 10-3 )** . The prevalence of second-hand smoke at home in the Brazilian capitals varied from 2.9% (95% CI, 0.9-4.9%) in Salvador to 9.6% (95% CI, 6.8-12.3%) in Rio Branco. Among males, the highest prevalence was in Rio de Janeiro, 11.2% (95% CI, 5.0-17.4%), whereas for females, the highest prevalence was in Belo Horizonte, 10.8% (95% CI, 7.0-14.6%). The prevalence of second-hand smoke at work was 5.4% (95% CI, 4.6-6.3%), higher among males, 8.1% (95% CI, 6.6-9.5%), than among females, 3.2% (95% CI, 2.4-4.0%).

**Table 10-3:** – Prevalence of second-hand smoke at work, by sex, according to age and years of schooling

	Total			Sex					
				Male			Female		
	Total	95% CI		Total	95% CI		Total	95% CI	
		Lower limit	Upper limit		Lower limit	Upper limit		Lower limit	Upper limit
**Total (%)**	5.4	4.6	6.3	8.1	6.6	9.5	3.2	2.4	4.0
**Age (years)**									
18-24	4.6	2.9	6.4	5.3	2.7	8.0	3.9	1.5	6.2
25-34	7.6	5.0	10.2	10.0	5.6	14.5	5.5	2.6	8.3
35-44	5.4	3.8	6.9	9.2	6.0	12.5	2.3	1.4	3.2
45-54	6.4	4.9	8.0	9.9	7.1	12.8	3.3	2.0	4.6
55-64	4.1	3.0	5.2	6.6	4.3	9.0	2.1	1.3	2.9
≥ 65	1.9	1.2	2.6	3.6	2.0	5.1	0.7	0.2	1.1
**Years of schooling**									
0-8	5.9	4.2	7.7	11.0	7.5	14.5	1.5	0.9	2.0
9-11	6.3	5.0	7.7	8.6	6.3	10.9	4.2	2.9	5.6
≥ 12	4.0	2.7	5.3	4.7	2.9	6.6	3.4	1.6	5.2

Source: VIGITEL Brazil 2021. ^252^

### E-cigarettes and hookahs

•Electronic smoking devices, more commonly called e-cigarettes, are battery-operated devices that provide nicotine, flavors, and other chemicals to the user in an aerosol. The result is an aerosol production with fine particulates.^357^The liquids used in the devices can be quite different in terms of chemical composition, concentration of nicotine and additives used, with more than 8000 flavors of e-cigarettes being described. In addition, the data reveal a discrepancy between the composition declared on the packaging and the actual composition of the product.^358^

•E-cigarettes entered the global marketplace around 2006. Brazil was one of the first countries in the world to ban the use of electronic smoking devices, in 2009.^359 , 360^

•In 2019, the prevalence of e-cigarette active use was 0.64% (95% CI, 0.51-0.76%) for the population aged ≥ 15 years. Among adults aged < 40 years, the prevalence was estimated to be 40 times that among adults ≥ 40 years (0.06% *vs.* 2.38%). Among the group aged 15-24 years who currently use e-cigarettes, 62.6% reported having never smoked industrialized cigarettes. The prevalence of e-cigarette use among individuals aged 15-65 years increased from 0.45% in 2015 to 0.72% in 2019. The prevalence increased especially among the age group of 15-24 years.^361^In the Covitel 2022 study, among the population ≥ 18 years, the prevalence of e-cigarette and hookah use any time in life was identical to that of the VIGITEL (7.3%; 95% CI, 6.0-8.9), with the highest prevalence of experimentation among young (18-24 years) adults.^352^Overall, 16.8% (95% CI, 16.2-17.4%) of the adolescents aged 13-17 years reported e-cigarette use any time in life with higher rates in males (19.1%; 95% CI, 18.3-19.9%) than in females (14.6%; 95% CI, 13.9-15.3%).^353^

•The prevalence of active use of hookah in 2019 was 0.47% (95% CI, 0.36-0.59%) in the population aged ≥ 15 years. That prevalence increased from 0.14% in 2013 to 0.43% in 2019 among individuals aged ≥ 18 years. For the age group of 18-24 years, the increase was around 300% in the same period.^362^

### Economic impact of tobacco use

•Using an economic model (Markov likelihood microsimulation) of individuals that considers the natural history, direct medical costs and indirect costs, the total economic burden attributable to tobacco in Brazil in 2020 was estimated in US$ 24.3 billion, representing 1.9% of the GDP and 7.8% of the national health expenditure.^354^The direct medical cost represents 38.5% of the economic burden, whereas 24.8% are represented by caregiver costs and 36.7% by loss of productivity.^354^Cardiovascular disease represented 24% of the direct medical costs (US$ 2.3 billion) attributable to tobacco, whereas stroke represented 4.8% (US$ 447 million) **( Table 10-5 )** .^354^

**Table 10-5:** – Annual economic burden attributable to tobacco, by cause, for Brazil, 2020 (US$ millions)

Economic burden (US$ millions)	Brazilian data for 2020
Direct medical cost (%)	9347.4 (38.5)
Caregiver cost (%	6023.7 (24.8)
Productivity cost (%)	6023.7 (24.8)
Total economic burden	24 301.3 (100%
As a proportion of GDP	1.9%
Proportion recovered through taxes	9.4%
**Direct medical cost (US$ millions)**	
CVD	2280.4 (24.4%)
Stroke	447.0 (4.8%
COPD	4307.8 (46.1%)
Pneumonia	31.2 (0.3%)
Lung cancer	453.2 (4.8%)
Other cancers	751.8 (8.0%)
Second-hand smoke	1076.0 (11.5%)
Total direct medical cost	9347.4 (100%)
Proportion of national health expenditure	7.8%
**Productivity cost (US$ millions)**	
**Premature death**	
CVD	917.7 (26.4%)
Stroke	324.8 (9.4%)
COPD	469.8 (13.5%)
Pneumonia	161.7 (4.7%)
Lung cancer	452.2 (13.0%)
Other cancers	739.8 (21.3%)
Second-hand smoke	405.9 (11.7%)
Total premature deaths	3471.8 (100%)
**Disability**	
CVD	1368.4 (25.1%)
Stroke	375.0 (6.9%)
COPD	1544.7 (28.3%)
Pneumonia	00.9 (0.0%)
Lung cancer	547.4 (10.0%)
Other cancers	988.3 (18.1%
Second-hand smoke	633.7 (11.6%)
Total disability	5458.4 (100%)
Premature death	3471.8 (38.9%)
Disability	5458.4 (61.1%)
Total productivity cost	8930.2 (100%)
**Costs of caregivers (US$ millions)**	
CVD	2160.5 (35.9%)
Stroke	530.0 (8.8%)
COPD	1918.1 (31.8%)
Pneumonia	39.0 (0.6%)
Lung cancer	224.3 (3.7%)
Other cancers	457.9 (7.6%)
Second-hand smoke	693.8 (11.5%)
Total cost of caregivers	6023.7 (100%)

CVD: cardiovascular disease; COPD: chronic obstructive pulmonary disease; GDP: gross domestic product. Source: Adapted from Pichon-Riviere et al. ^354^

### Impact of COVID-19 in tobacco consumption

•During the COVID-19 pandemic, the prevalence of adolescent tobacco use remained stable (2.41%; 95% CI, 2.02-2.87). Factors associated with a higher likelihood of using tobacco during the COVID-19 pandemic were as follows: age between 16 and 17 years; self-identification as black; residence in the Southern and Southeastern regions; reported feeling of depression and loneliness; sleeping issues that got worse; alcohol use; passive smoking.^362^

•In the Covitel 2022 study, among the population aged ≥ 18 years, there was stability of smoking prevalence: 14.7% (95% CI, 13.0-16.7%) before the COVID-19 pandemic and 12.2% (95% CI, 10.4-14.1%) in the first trimester of 2022 **( Table 10-6 )** .^363^

**Table 10-6:** – Prevalence of smoking before and after the COVID-19 pandemic, according to sex, Brazilian major regions, age groups, and years of schooling

	Before COVID-19 pandemic			2022 first trimester		
	Total	95% CI		Total	95% CI	
		Lower limit	Upper limit		Lower limit	Upper limit
**Total**	14.7	13.0	16.7	12.2	10.4	14.1
**Sex**						
Male	18.0	15.5	20.7	14.5	12.2	17.2
Female	11.7	9.9	13.9	9.9	8.1	12.2
**Major Regions**						
Northeast	10.1	7.2	13.9	7.9	5.8	10.7
North	12.1	10.5	13.9	8.0	6.1	10.4
South	18.9	15.0	23,5	15.5	11.9	19.9
Southeast	16.3	14.3	18.5	14.3	12.0	16.8
West-Central	16.7	15.1	18.4	12.6	10.3	15.2
**Age groups (years)**						
18 to 24	13.9	9.9	19.3	12.1	8.4	17.1
25 to 34	17.0	12.7	22.3	14.5	9.9	20.9
35 to 44	13.9	11.0	17.3	11.1	8.7	14.2
45 to 54	13.8	10.9	17.5	12.4	9.3	16.2
55 to 64	17.4	13.8	21.6	13.6	9.6	18.9
≥ 65	12.1	9.3	15.6	8.0	5.8	10.9
**Race**						
White	13.2	11.2	15.4	10.8	9.0	13.1
Black	15.0	12.7	17.5	12.4	10.2	15.0
Others	21.7	15.4	29.6	17.7	11.8	25.7
**Years of schooling**						
0 to 8	17.6	15.2	20.4	14.7	12.4	17.3
9 to 11	14.1	11.5	17.2	11.6	9.0	14.7
≥ 12	8.3	6.8	10.1	6.5	5.4	7.8

Source: COVITEL 2022. ^352^

•According to an on-line survey, the prevalence of smokers during the COVID-19 pandemic was 12% (95% CI, 11.1-12.9%), and 34% of smokers reported an increase in cigarette consumption. The increased cigarette use was associated with worse quality of sleep, feeling isolated from family members, feeling sad or anxious, financial problems, and having lower self-rated health.^364 , 365^

### Public policies for tobacco control in Brazil

•The tobacco control process in Brazil began in 1981 with the creation of the Commission for the Study of the Consequences of Tobacco Use by the Ministry of Health. In 2006, Brazil ratified the WHO’s Framework Convention on Tobacco Control. The country banned smoking in closed spaces except for designated spaces (smoking cabins) in 1996 and, in 2011, completely banned smoking in all public and workspaces, in addition to public transport. The 2011 tax reform made a substantial contribution to lowering tobacco use among the numerous anti-smoking laws and regulations put in place in Brazil over the past few decades. The reform gave the govern authority to set a minimum price for a pack of cigarettes, which would be increased every year over anticipated inflation rates.^366^The tax increase that rises cigarette prices has been shown to reduce medical expenses on tobacco-related diseases. Each 10%-price increase due to higher tobacco taxes reduces tobacco consumption by 5%.^367^Due to all these measures, there has been a clear decline in tobacco consumption among adults since 1989.^368^Smoking prevalence has declined sharply by 63.2% over the past three decades.^256^

•Consequently, the mortality burden attributable to smoking decreased 70.1% from 1990 to 2017, with the greatest reduction observed for stroke (-75.3%).^256^

•Based on statistics from 2019, the proportion of smuggled illicit cigarettes in Brazil was projected at 38.6% (95% CI, 35.8- 41.5%). A quarter of illicit brand cigarettes was sold at or above the minimum legal price.^366^

### Future Directions

•The progressive reduction in smoking prevalence in Brazil can be attributed to the country’s adoption of various tobacco control measures, such as prohibiting the sale of tobacco to minors, including warning labels on cigarette packs, banning tobacco advertising, promotion, and sponsorship, raising tobacco taxes, creating smoke-free environments, and other recommendations made by the Framework Convention on Tobacco Control.^369^A progressive tobacco tax increase policy would reduce cigarette consumption and medical costs. Consequently, healthier, and more productive lives result in higher incomes for everyone.^369^Policymakers need to continuously promote the framework implementation to eliminate illicit trade in tobacco products, particularly focusing populations with higher prevalences, such as SGM.^370^

•There is an increasing concern about the new routes to nicotine addiction by e-cigarettes and hookah. Tobacco industry mainly targets young people, and vaping is promoted as being safe and harmless. Future research should focus on both health risks associated with these products and the development of strategies to target their increasing use in specific populations, such as adolescents and young adults. Furthermore, the prevalence of the use of other types of e-cigarettes and hookah, the type of device, and the frequency of use need to be better estimated.^352^

## CHAPTER 11 – OBESITY AND OVERWEIGHT

### ICD-10 E66

**See Tables 11-1 through 11-4 and Charts 11-1 through 11-4**

**Table:** Abbreviations Used in Chapter 11

BMI	Body Mass Index
CI	Confidence Interval
CVD	Cardiovascular Disease
DALYs	Disability-Adjusted Life-Years
ELSA-Brasil	The Brazilian Longitudinal Study of Adult Health (in Portuguese *, Estudo Longitudinal de Saúde do Adulto* )
FU	Federative Unit
HR	Hazard Ratio
ICD	International Statistical Classification of Diseases and Related Health Problems
ICU	Intensive Care Unit
OR	Odds Ratio
PNS	Brazilian National Health Survey (in Portuguese, *Pesquisa Nacional de Saúde* )
SIA/SUS	Outpatient Information System from the SUS (in Portuguese, *Sistema de Informação Ambulatorial do SUS* )
SIH/SUS	Inpatient Information System (in Portuguese, *Sistema de Informação Hospitalar do SUS* )
SUS	Brazilian Unified Health System (in Portuguese, *Sistema Único de Saúde* )
VIGITEL	Telephone Survey for Surveillance of Non-Communicable Chronic Diseases (in Portuguese, *Vigilância de Fatores de Risco e Proteção para Doenças Crônicas por Inquérito Telefônico* )
WHO	World Health Organization

### Overview

•Obesity is a risk factor for CVD, as well as a complex chronic disease. According to the WHO, obesity is defined as abnormal or excessive accumulation of fat that presents a risk to health. A BMI ≥ 30 kg/m^2^is considered obesity, while a BMI greater than or equal to 25 kg/m^2^is considered excess weight or overweight.^371^It is worth noting that obesity is a chronic disease resulting from multifactorial causes mainly related to lifestyle (sedentary lifestyle, inadequate eating habits) and to other conditions, such as genetic, hereditary, psychological, cultural, and ethnic factors.

•Obesity remains a public health threat. Gaspar *et al* .^372^have investigated the association of hyperglycemia, obesity, dyslipidemia, and smoking from 2005 to 2017 in Brazil, as well as the mortality or incidence rates of CVD, ischemic heart disease or ischemic stroke, per 100 000 individuals. The exposure to risk factors was calculated as the summary exposure value, a measure of the population exposed to a risk factor that considers the extent of exposure by risk level and the severity of that risk contribution to disease burden. In that time range, the age-standardized mortality and incidence of CVDs decreased by 21% and 8%, respectively, from 2005 to 2017. However, there was a 9.5% increase in the summary exposure value for hyperglycemia, a 31% increase for obesity, and a 5.2% increase for dyslipidemia, while the summary exposure value for hypertension was stable, and that of smoking displayed a 33% reduction. This underscores the importance of obesity in relation to CVD and the urgent need for policies to decrease obesity in the Brazilian population, in an effort to mitigate the burden of CVD mortality.

### Prevalence

•In Brazil, in 2019, according to anthropometric data from the PNS, the percentages of adults (age ≥18 years) with excess weight and obesity were, respectively, 57.5% (95% CI, 54.8 – 60.2) and 21.8 % (95% CI, 19.2 – 24.7) for men, and 62.6% (95% CI, 59.1 – 66.0) and 29.5% (95% CI, 25.4 – 34.0) for women.^373^

**•Table 11-1** shows the prevalence of excess weight among individuals aged 18 years and over, both sexes and all ages, in Brazilian capitals, in 2021, according to the age groups from the VIGITEL 2021. In Brazilian capitals, the percentages of adults (age ≥18 years) with excess weight in 2021 were 57.2% (95% CI, 55.7-58.8) overall, 59.9% (95% CI, 57.6-62.2) for men, and 55.0% (95% CI, 53.0-57.0) for women. Progressive increase of excess weight was observed with age increase, ranging from 35.7% (95% CI, 31.5-40.0) [male: 39.3% (95% CI, 33.6-45.1); female: 31.7% (95% CI, 25.5-37.9)] in the age group of 18-24 years to 64.4% (95% CI, 61.5-67.2) [male: 67.2% (95% CI, 62.6-71.9); female: 61.9% (95% CI, 58.5-65.3)] in the age group of 45-54 years. For the age group of 60+ years, there was a slight reduction in the excess weight prevalence, 60.7% (95% CI, 58.8-62.6) [male: 60.7% (95% CI, 57.2-64.2); female: 60.7% (95% CI, 58.6-62.9)].

**•Table 11-2** shows the prevalence of obesity among individuals aged 18 years and over, of both sexes and all ages, in Brazilian capitals, in 2021, according to the age groups from the VIGITEL 2021. In Brazilian capitals, the percentages of adults (age ≥18 years) with obesity in 2021 were 22.4% (95% CI, 21.1-23.6) overall, 22.0% (95% CI, 20.0-24.0) for men, and 22.6 (95% CI 21.1-24.2) for women. Progressive increase of obesity was observed with age increase, ranging from 12.2% (95% CI, 9.2-15.3%) [male: 13.1% (95% CI, 9.3-17.0); female: 11.2% (95% CI, 6.4-16.0)] in the age group of 18-24 years to 26.2% (95% CI, 23.7-28.8) [male: 24.3% (95% CI, 20.3-28.3); female: 27.9% (95% CI, 24.7-31.2)] in the age group of 45-54 years. For the age group of 60+ years, there was a slight reduction in the obesity prevalence, 21.8% (95% CI, 20.2-23.4) [male: 16.8% (95% CI, 14.1-19.5); female: 25.3% (95% CI, 23.4-27.2)].

**•Table 11-3** shows the percentage of overweight adults, by sex, in the Brazilian capitals and the Distrito Federal, according to VIGITEL 2021 data. Most capitals showed higher percentages than the national values for both sexes, except for Palmas, Salvador, São Luís, Teresina, and Vitória. The same occurred for men in the capitals Belo Horizonte, Boa Vista, Cuiabá, Florianópolis, Maceió, Natal, Palmas, Recife, Salvador, São Luís, São Paulo, Vitória, and Distrito Federal, which showed smaller percentages than the national values. It is worth noting that, for female excess weight, the capitals below the national mean were: Palmas, Salvador, São Luís, Teresina, and Vitória. **Chart 11-1** shows the percentage of overweight adults in the Brazilian capitals and the Distrito Federal according to VIGITEL 2021 data, stratified by quartiles. The capitals in the highest quartile were in the states of Acre, Amazonas, Amapá, Pará, and Rondônia.

**Chart 11-1:** – Percentage of overweight adults in the Brazilian capitals and Distrito Federal, according to VIGITEL 2021 data, stratified by quartiles. Data from the VIGITEL Brazil 2021. ^252^

**•Table 11-4** shows the percentage of obese adults, by sex, in the Brazilian capitals and the Distrito Federal, according to VIGITEL 2021 data. Most capitals showed higher percentages than the national values for both sexes, except for Belo Horizonte, Campo Grande, Florianópolis, Palmas, Rio de Janeiro, Salvador, São Luís, Teresina, and Vitória. The same occurred for men in the capitals Belo Horizonte, Campo Grande, Florianópolis, Maceió, Recife, Salvador, São Luís, São Paulo, and Vitória, which showed lower percentages than the national values. It is worth noting that, for females with obesity, the number of capitals below the national mean was greater than for men: Belo Horizonte, Campo Grande, Florianópolis, Palmas, Rio de Janeiro, Salvador, São Luís, São Paulo, Teresina, and Vitória. **Chart 11-2** shows the percentage of obese adults in the Brazilian capitals and the Distrito Federal according to VIGITEL 2021 data, stratified by quartiles. The capitals in the highest quartile were in the states of Acre, Amazonas, Amapá, Rondônia, Mato Grosso, and Goiás.

**Chart 11-2:** – Percentage of adults with obesity in the Brazilian capitals and Distrito Federal, according to VIGITEL 2021 data, stratified by quartiles. Data from the VIGITEL Brazil 2021. ^252^

**•Charts 11-3** and **11-4** are time series showing the percentage of overweight and obese adults (≥ 18 years), respectively, in Brazil, from 2006 to 2021, according to VIGITEL data. There was an upward trend in the percentage of overweight and obesity in Brazil from 2006 to 2021, according to VIGITEL data.

#### Children and adolescents

•A cross-sectional study based on data from the Study on Cardiovascular Risk in Adolescents has assessed 2530 adolescents aged 12–17 years enrolled in public and private schools in Belo Horizonte city. The study aimed to associate schools’ internal environment factors and its surroundings with adolescent obesity in a Brazilian metropolis. The prevalence of obesity was 7.21%. Internal and external school food environment aspects, such as the number of operational drinking fountains and availability of stores selling ready-to-eat food around schools, were associated with obesity in adolescents from a Brazilian metropolis.^374^

•The increase in obesity prevalence has also been observed among Brazilian children and adolescents. A systematic review and meta-analysis of 53 studies (n = 122 395) held from 1986 to 2015 has shown a prevalence of obesity in the period of 8.2% (95% CI, 8.1–8.4%, I2 = 98.5%). Higher prevalence was observed among boys (9.7% [9.4–9.9%], I2 = 97.4%) than among girls (7.3% [7.1−7.5%], I2 = 96.1%). The prevalence increased according to the decade (1990: 6.5% [6.0–7.0%], I2 = 96.8%; 2000: 7.9% [7.7–8.0%], I2 = 98.8%; 2010: 12.0% [11.5–12.6%], I2 = 95.8%) and Brazilian region (Northeast: 6.4% [6.2−6.7%], I2 = 98.1%; North: 6.7% [6.3−7.2%], I2 = 98.8%; Southeast: 10.6% [10.2−11.0%], I2 = 98.2%; South: 10.1 [9.7−10.4%], I2 = 97.7%). The authors concluded that for every 100 Brazilian children, more than 8 had obesity in the three-decade period and, in more recent estimates, 12 for every 100 had childhood obesity.^375^

## Incidence

•The ELSA-Brasil study has included 13 625 women and men aged 35-74 years (2008-2010) who attended a medical visit after a mean 3.8-year follow-up. The investigators measured the height and weight of all participants during in-person visits. They reported a global incidence of 7.7% of overweight and of 10.6% of obesity, with higher levels among black women (28.5%), young men (21.1%), and women with low educational levels (35.0%). The proportions of overweight and obesity increased with age, more commonly among those with the lowest *per capita* income levels and fewer years of schooling.^376^

## Mortality attributable to obesity

•In 2021, 1.95 million (95% CI, 1.12-2.91) cardiovascular deaths and 3.7 million (95% CI, 1.97-5.49) deaths overall were attributable to elevated BMI.^377^

•From 1990 to 2019, there was a negative change in the mortality rates from CVD attributable to high BMI for women [-33.9 (-43.7;-16.7)], which was higher than that for men [-22.8 (-35.9;6.2)]. The highest decreases in the percentage of mortality occurred in the FUs with higher income in Brazil.^4^

## Burden of Disease Attributable to Obesity

•In 2021, the all-cause DALYs due to high BMI were 1560 (95% CI, 711-2380) per 100 000.^377^

•Most FUs had a decrease in the age-standardized rates of DALYs due to CVD attributed to high BMI for women from 1990 to 2019. Similar behavior was observed in those rates for men, with a percent decrease of obesity in the period.^4^

## Impact on Cardiovascular Health

•Obesity and its accompanying risk factors have a strong interplay. In the *EpiFloripa Aging* study,^378^a longitudinal, population, home-based study carried out in Florianópolis, in Southern Brazil, from 2009/2010 to 2013/2014, 559 individuals were evaluated. There was an association between those who were or became overweight (OR = 4.59; 95% CI, 3.05–6.89) and the metabolic syndrome diagnosis: they were 4.71 times more likely to have metabolic syndrome than those who neither were nor became overweight. In the *Baependi Heart Study* ,^379^a longitudinal study designed to investigate factors associated with the development of CVD in the citizens of Baependi, Minas Gerais state, since 2005, incident hypertension was monitored for 10 years and was found to be 24.3%. The BMI was the best predictor of hypertension in a model also including age, sex, high-density-lipoprotein cholesterol, and systolic blood pressure.

•Of note, the association between obesity and hypertension is also found in the young, as described by Santiago *et al* .^380^In a cross-sectional study of 1132 adolescents (16.50 ± 1.14 years) of both genders, male adolescents with excess peripheral, central, and general adiposity were more likely to have high systolic blood pressure.

•Finally, data from two cohorts of the cross-sectional *Health Survey of São Paulo focusing on Nutrition (ISA-Nutrition study* ), a sub-sample of the *Health Survey of São Paulo (ISA-Capital* ), carried out in 2008 and 2015, showed that excess body weight was associated with higher odds of presenting all the other risk factors analyzed.^381^The distribution of body fat is also a determinant of risk, with lower body fat accumulation being considered protective as opposed to abdominal obesity. Oliveira *et al* .^333^have estimated, among 10 917 participants from the *ELSA-Brasil* study, the associations between lower limb/trunk fat ratio and the 10-year CVD risk, according to the Framingham Risk Score, stratified by gender and adjusted by age, skin color, educational level, alcohol consumption, leisure physical activity, hypolipidemic drug use, and menopausal status. A higher lower limb/trunk fat ratio was related to lower 10-year CVD risk, as well as a reduction in systolic blood pressure, total cholesterol, and antihypertensive drug use, especially in women.

•A meta-analysis with 46 studies has evaluated the association between urbanization (including data from Amazon rainforest deforestation) and cardiometabolic risk factors and outcomes. The assessment of 20 574 adults from 33 Indigenous Brazilian ethnicities has shown higher rates of obesity (West-Central region: 23% [95% CI, 17–29]; and Southern region: 23% [13–34]) in Indigenous peoples living in urban areas in Brazil and lower rates of obesity (11% [95% CI, 8–15]) in those in less urbanized regions of Brazil (Northern region). The prevalence of obesity was 3.5 times higher in participants living in urbanized Indigenous territories (28%) than in those living in lands with >80% native Amazon rainforest (8%). In addition, between 1997 and 2019, the CVD mortality rate in individuals residing in the Southeastern region (the most urbanized) was 2.5 times greater than that observed in the Northern areas.^259^

## Health Care Utilization and Cost

•Obesity is associated with increased direct costs (for example, for the diagnosis and treatment of the disease) and indirect costs (related to lost labor earnings and premature mortality). Ferrari *et al* .,^337^in a very interesting study, provided an estimate of the economic burden of diseases attributable to overweight and obesity in the SUS. They developed a macrosimulation model to estimate the economic burden of diseases attributable to high BMI in Brazil. Self-reported data on weight and height from the Brazilian PNS conducted in 2019 were used to calculate the BMI distribution and included 85 715 adults aged ≥20 years. The mean and standard deviation of BMI and prevalence of overweight and obesity were calculated by sex and Brazilian FU. Direct healthcare costs of diseases were obtained from the Outpatient Information System (SIA/SUS) and the Inpatient Information System (SIH/SUS) in 2019. Their results showed that, in Brazil, in 2019, US$ 654 million in direct healthcare costs related to non-communicable diseases were attributable to high BMI. Attributable costs were higher among women than among men. The disease with the highest attributable costs was CVD, followed by chronic respiratory diseases, neoplasms, digestive diseases, musculoskeletal disorders, diabetes, and kidney diseases. Comparing women and men, the diseases with the highest attributable costs in women were neoplasms, digestive diseases, musculoskeletal disorders, diabetes, and kidney diseases, while the diseases with the highest attributable costs in men were CVDs and chronic respiratory diseases. The highest attributable costs were found in the FUs of the Southeastern and Southern regions, São Paulo, followed by Minas Gerais and Paraná. These data underscore the impact of obesity on the Brazilian health and economics, showing the need for public policies to control obesity, which may result in cost savings additionally to the more obvious populational health benefits.

## Obesity and COVID-19

•The COVID-19 pandemic led to worldwide observations of the association between obesity and adverse outcomes of infected individuals.^382 - 386^In a hospital-based, multicenter study of 8183 ICU hospitalized patients who tested positive for SARS-CoV-2,^387^when stratifying by BMI categories and adjusting for age, sex, and smoking status, the patients with severe obesity showed an increased risk for COVID-19 mortality (HR 1.21; 95% CI, 1.03–1.43) compared to those with normal/overweight. No difference was observed for the mild/moderate obese (HR 0.91; 95% CI, 0.83–1.00) and underweight (HR 1.21; 95% CI, 0.80–1.81) categories. In addition, for the survivors in the highest BMI category (≥ 40 kg/m^2^), the length of stay in ICU was 31% higher compared to those in the normal/overweight category. An increased ICU length of stay was also observed for mild/moderate obesity in comparison to normal/overweight. No difference was detected for the underweight group. However, when stratified by age, an increased risk of mortality was only observed for younger patients (< 60 years) compared to those with normal/overweight (HR 1.27; 95% CI, 1.01–1.61). An increased risk for death was also observed for underweight patients (HR 3.74; 95% CI, 1.39–10.07). For patients aged ≥ 60 years, mild/moderate obesity was associated with a reduced mortality risk (HR 0.87; 95% CI, 0.78–0.97).

•On the other hand, a multicenter cohort study conducted in eight different states in Northeastern Brazil, GENSCoV-BR,^386^has analyzed demographic, clinical, and anthropometric data of individuals who tested positive for SARS-CoV-2, as well as outcomes including hospitalization, mechanical ventilation, and death, to assess the effects of extremes in body weight on clinical outcomes. Among 1308 individuals, excess body weight was observed in 66.9%, underweight in 2.7%, and obesity in 32.4%, according to the WHO classification. Hospitalization occurred in 75.2%, and 22.2% died. Hospitalization was more often observed among underweight (3.2% *vs.* 1.2%) and overweight (68.1% *vs.* 63.3%) individuals, although, on multivariable analysis, body weight status (underweight or overweight) was not associated with the risk for hospitalization, mechanical ventilation, or death. The different results might reflect the different characteristics of the populations studied and, most importantly, that the studies were focused on either patients who were already hospitalized (and, thus, more severely ill) or outpatients with lower-severity COVID-19.^387^

•VIGITEL’s temporal trends have shown that, between 2006 (the beginning of the VIGITEL system) and 2019 (before the pandemic), there was an increase in the prevalence of overweight, from 55.4% (95% CI, 54.4-56.3) in 2019 to 57.2% (95% CI, 55.7-58.8) in 2021/22. In addition, the prevalence of obesity increased from 20.3% (95% CI, 19.5-21.0) in 2019 to 22.4% (95% CI, 21.1-23.6) in 2021/22. Obesity also increased in men, from 19.5% (95% CI, 18.3-20.7) to 22.0% (95% CI, 20.0-24.0).^388^

## Future Directions

•As obesity builds up worldwide, including in Brazil, and considering the contribution of obesity to CVD, increased efforts are needed to control this issue. Otherwise, the favorable results observed in previous years, showing a reduction in CVD mortality, might soon be lost. In past decades, successful tobacco control programs have been implemented in Brazil, demonstrating that nationwide interventions directed at weight control can be built. National-level policies are needed to 1) prevent obesity, and 2) reduce the prevalence of established obesity. Community-based, sustainable, multidisciplinary interventions aimed at increasing physical activity, reducing the consumption of ultra-processed foods while promoting healthy food ingestion and reinforcing the importance of the Dietary Guidelines for the Brazilian population are key in this process.

•Finally, several discussions regarding the use of the new weight-reducing medications, including their cost-effectiveness, might be necessary, because they are a reality that seems to have come to stay.

## CHAPTER 12 – PHYSICAL ACTIVITY

### See Tables 12-1 to 12-5 and Charts 12-1 to 12-5

**Table:** Abbreviations used in Chapter 12

CI	Confidence Interval
COVID-19	Coronavirus Disease 2019
CVD	Cardiovascular Disease
DALYs	Disability-Adjusted Life-Years
ELSA-Brasil	Longitudinal Study of Adult Health - Brazil
GBD	Global Burden of Disease
MET	Metabolic Equivalent of Task
min	minute
PENSE	National Survey on the Health of Schoolchildren (in Portuguese, *Pesquisa Nacional de Saúde do Escolar* )
PNS	National Health Survey (in Portuguese, *Pesquisa Nacional de Saúde* )
SDI	Sociodemographic Index
SUS	Brazilian Unified Health System (in Portuguese, *Sistema Único de Saúde* )
TV	Television
UI	Uncertainty Interval
VIGITEL	Surveillance System of Risk and Protection Factors for Chronic Diseases by Telephone Survey (in Portuguese, *Sistema de Vigilância de Fatores de Risco e Proteção para Doenças Crônicas por Inquérito Telefônico* )
V̇O_2_	Oxygen consumption

### Introduction

•“Physical activity” and “physical exercise” have different meanings. Physical activity is defined as any bodily movement produced by skeletal muscle contraction resulting in an increase in energy expenditure as compared to the state of rest.^389 , 390^Physical activity comprises recreational or occupational activities spontaneously performed, and some examples are as follows: walking to work, house cleaning, lawn mowing, climbing up and down stairs, etc. Physical exercise is a type of physical activity. For physical activity to be considered physical exercise, it must be: (a) structured; and (b) performed to achieve or improve physical fitness components, such as performance, force, power, resistance, balance, etc.^391 , 392^

•The terms “sedentary behavior” and “physical inactivity” are controversial regarding their definitions.^393^Sedentary behavior can be defined as performing activities with a very low energy expenditure, of 1.5 MET or less.^394^Typical examples include many hours watching screens (TV or tablet). Physical inactivity refers to performing a smaller volume of physical activity per week than the minimum recommended by current guidelines, which is 150 min/week of moderate-intensity physical activity or 75 min/week of vigorous-intensity physical activity and that corresponds to 450 MET-min/week.^395^Other terms for that definition can be found in the literature, such as “insufficient physical activity practice” used in the VIGITEL Brazil.^252^

•One MET is equivalent to the estimated V̇O_2_at rest, which approximates 3.5 mL.kg^- 1^.min^- 1^. The following classification related to the V̇O_2_necessary to perform a certain physical activity, that is, its intensity, can be used: (a) ≤1.5 MET = sedentary behavior; (b) >1.5 and <3 METs = light-intensity physical activity; (c) ≥3 and <6 METs = moderate-intensity physical activity; (d) ≥6 METs = vigorous-intensity physical activity. From an even more practical perspective, 2.5 to 5 hours of moderate-intensity activity per week is equivalent to approximately 7.5 to 29.5 MET-hour/week or 450 to 1770 MET-min/week (equivalent to 150 to 300 min/week of moderate-intensity exercise), and this is the standard recommendation for physical activity practice for adults.^395^

•The recommendation of physical activity for children and adolescents is an average of 60 min/day of moderate/vigorous-intensity aerobic physical activity. Older adults (>65 years of age) should do light/moderate-intensity physical activity at least 3 days/week, focusing on activities to reduce the risk of falls and improve muscle function.^395 , 396^In addition, the guidelines emphasize that more than 300 min/week of moderate-intensity physical activity provide additional health benefits.^396^

•The first guide of physical activity for the Brazilian population, published by the Brazilian Ministry of Health in 2021,^397^has similar recommendations ( **Table 12-1** ). It emphasizes the importance of including activities to strengthen muscles and bones at least 2 days/week. Children and adolescents (5-17 years of age) should engage in at least 1 hour of physical activity per day and at least 3 days/week should be intended for muscle and bone strengthening. Regarding screen time, the American Academy of Pediatrics^398^recommends that children aged less than 18 months should not spend any time watching screens, and those aged 18 months to 5 years should limit it to a maximum of 1 hour/day. For children aged 6 years or more, there is no maximum time defined, as long as screen time does not interfere with sleep or physical activity levels.

**Table 12-1:** – Recommendations for physical activity practice according to the First Guide of Physical Activity for the Brazilian Population

Physical activity	Young (6-17 years of age)	Adults	Elderly
If moderate intensity	≥60 min/day*	≥150 min/week	≥150 min/week
If vigorous intensity	NM	≥75 min/week	≥75 min/week
Muscle strengthening	Include in the 60 min/day	≥2 days/week	2-3 times/week
Balance	NM	NM	2-3 times/week

*Can be divided in blocks; NM: not mentioned. Source: VIGITEL Brazil 2021. ^252^

### Prevalence

•According to data from the VIGITEL 2021, approximately 48% of the Brazilian adult population does not meet the recommended level of physical activity, and the percentage is higher among women (56%) as compared to men (39%). The highest frequency of inactive adults was found in the city of Porto Alegre (51.8%) and the lowest, in the city of Goiânia (39.8%).^252^The percentage of physically inactive adults varied as follows: among men, from 30.3% in the city of Goiânia to 46.1% in the city of Campo Grande; while among women, from 43.4% in the city of Florianópolis to 63.1% in the city of Rio de Janeiro ( **Chart 12-1 and Table 12-2** ).

**Chart 12-1:** – Frequency of physically inactive adults aged ≥18 years in the Distrito Federal and capitals of the Brazilian states according to data from VIGITEL 2021. Data from the VIGITEL Brazil 2021. ^252^

**Table 12-2:** – Frequency of physically inactive adults (≥18 years of age), by sex, in the capitals of the Brazilian states and Distrito Federal, based on data from VIGITEL 2021

	Men		Women	
Capitals	%	95% CI	%	95% CI
Aracaju	37.6	29.1 - 46.2	49.9	44.4 - 55.5
Belém	36.2	29.1 - 43.3	54.2	48.6 - 59.9
Belo Horizonte	40.1	33.0 - 47.3	50.8	45.2 - 56.3
Boa Vista	33.7	27.3 - 40.1	49.8	44.9 - 54.7
Campo Grande	46.1	36.7 - 55.6	56.1	49.3 - 62.9
Cuiabá	44.8	36.2 - 53.3	53.1	46.5 - 59.6
Curitiba	37.8	30.9 - 44.8	54.1	47.9 - 60.3
Florianópolis	40.9	33.1 - 48.7	43.4	37.3 - 49.4
Fortaleza	36.6	29.2 - 44.0	57.1	51.1 - 63.1
Goiânia	30.3	23.4 - 37.3	48.2	42.2 - 54.1
João Pessoa	43.5	35.1 - 51.9	53	46.7 - 59.4
Macapá	38	31.5 - 44.4	52	46.5 - 57.4
Maceió	40.1	32.2 - 48.0	52	44.9 - 59.1
Manaus	37.3	29.1 - 45.4	59.2	53.5 - 64.8
Natal	33.9	26.7 - 41.1	50.4	44.3 - 56.4
Palmas	38.1	29.5 - 46.6	49.4	43.8 - 55.0
Porto Alegre	42.3	34.9 - 49.7	59.6	52.0 - 67.3
Porto Velho	40.9	33.3 - 48.4	54.9	49.2 - 60.6
Recife	34.4	26.3 - 42.5	58.1	52.1 - 64.1
Rio Branco	39.8	32.7 - 46.8	54.7	48.8 - 60.6
Rio de Janeiro	38.2	30.3 - 46.0	63.1	57.1 - 69.0
Salvador	38.1	30.5 - 45.7	55.8	50.1 - 61.4
São Luís	36	28.4 - 43.7	56.3	50.6 - 61.9
São Paulo	43.2	36.4 - 50.1	55.6	49.3 - 61.8
Teresina	37	29.3 - 44.7	57.3	51.4 - 63.2
Vitória	38	30.6 - 45.3	48.8	42.8 - 54.8
Distrito Federal	38.1	30.4 - 45.7	51.4	45.2 - 57.7

*CI: confidence interval. Source: VIGITEL Brazil 2021.^*252*^*

•For both sexes, age showed a direct association with the frequency of physical inactivity ( **Table 12-3** ). In the age group of 18-24 years, the frequency of physical inactivity was 35.6% (27.6% among men and 44.5% among women), while, in individuals aged 65 years and older, that percentage was 73% (65.6% among men and 78.2% among women).^252^

**Table 12-3:** – Frequency of physically inactive adults (≥ 18 years of age), according to sex and age group, based on data from VIGITEL 2021

	Men		Women		Total	
Age group	%	95% CI	%	95% CI	%	95% CI
18 - 24 years	27.6	22.5 - 32.7	44.5	37.6 - 51.4	35.6	31.3 - 39.8
25 - 34 years	33.9	28.0 - 39.7	50.2	44.7 - 55.6	42.6	38.5 - 46.7
35 - 44 years	37.0	31.5 - 42.5	51.4	46.9 - 55.9	45.0	41.5 - 48.6
45 - 54 years	37.8	33.1 - 42.4	53.8	50.3 - 57.4	46.3	43.3 - 49.2
55 - 64 years	48.4	43.6 - 53.3	62.8	59.6 - 65.9	56.5	53.8 - 59.3
65+ years	65.6	62.2 - 68.9	78.2	76.4 - 80.0	73.0	71.3 - 74.7

CI: confidence interval. Source: VIGITEL Brazil 2021. ^252^

•An inverse relationship was observed between educational level and physical inactivity. Among those with 0 to 8 years of schooling, the frequency of physical inactivity was 58.4% (47.7% among men and 67.9% among women), while, among those with 12 or more years of schooling, that frequency was 43.5% (39.7% among men and 46.3% among women).^252^

**•Chart 12-1** illustrates the frequency of physical inactivity in the Distrito Federal and capitals of the 26 Brazilian states, according to data from the VIGITEL 2021.^252^

•Regarding the adoption of sedentary behaviors, in 2021, 66% of the adults from the capitals of the 26 Brazilian states and Distrito Federal reported spending at least 3 hours/day of their leisure time watching screens (TV, computer, tablet, or cellular phone), and that frequency is similar among men (66.7%) and women (65.4%).^252^The lowest percentages were observed in the city of Recife (58.9%), among adults aged ≥65 years (51%), and those with 0 to 8 years of schooling (49.2%). However, the highest frequencies were found in the city of Rio de Janeiro (70.1%), in the age group of 18-24 years (83.2%), and those with 12 or more years of schooling (73%).

**•Chart 12-2** illustrates the frequency of adults (≥18 years) spending ≥3 hours/day of their leisure time watching screens (TV, computer, tablet, or cellular phone) in the capitals of the 26 Brazilian states and Distrito Federal, according to data from the VIGITEL 2021.^252^

•Data from the ELSA-Brasil study, between 2008 and 2010, from 15 105 men and women aged 35-74 years, have shown that less than half of that population practiced physical activity. That study showed a prevalence of leisure-time physical activity of 44.1% among men and of 33.8% among women. That proportion was greater among individuals aged ≥ 60 years [men (46.7%) and women (39.7%)].^399^

### Trends

•According to the time series of VIGITEL,^400^the frequency of physically inactive adults (≥18 years) remained stable between 2013 and 2020, being 49.4% and 47.2%, respectively, resulting in a mean annual variation measured in percent points per year (pp/year) of -0.48 (95% CI, -1.15;0.19). That stability was also observed when the sexes were assessed separately. In 2013, 39.9% of the men and 57.4% of the women were physically inactive, and, in 2020, the figures were 37.3% and 55.6%, respectively, corresponding to a mean annual variation of -0.50 (95% CI, -1.20;0.21) pp/year for men and of -0.47 (95% CI, -1.25;-0.30) pp/year for women.

•However, the variation between 2013 and 2020 was not similar in the different age groups and educational levels. While the frequency of physically inactive adults was stable in the age groups of 18-24 years, 25-34 years, and 55-64 years, a reduction was observed in the age groups of 35-44 years (-0.68 pp/year; 95% CI, -1.34;-0.02), of 45-54 years (-1.20 pp/year; 95% CI, -1.59;-0.81), and of 65 years or older (-0.55 pp/year; 95% CI, -0.84;-0.26).^400^Regarding educational level, among adults with 0 to 8 years and with 9 to 11 years of schooling, the percentage of physically inactive individuals was stable; however, among those with at least 12 years of schooling, there was a reduction in that percentage (-0.45 pp/year; 95% CI, -0.86;-0.04).^400^

•The variation in the percentage of physically inactive adults aged ≥18 years according to Brazilian data of the last 5 years (2017-2021) made available by VIGITEL is shown in **Chart 12-3** .^400^

**Chart 12-3:** – Percentage of physically inactive* adults aged ≥18 years in Brazil, according to sex, in the last 5 years available in the VIGITEL database. *Less than 150 min/week of moderate-intensity physical activities or less than 75 min/week of vigorous-intensity physical activities. Data from the VIGITEL Brazil 2021. ^252^

•Similarly, data from the PNS have shown a relative increase of over 30% in the proportion of Brazilian adults reporting engaging in moderate to vigorous physical activity for at least 150 min/week from 2013 to 2019, increasing from 22.7% in 2013 to 30.1% in 2019. In both years, that proportion was higher among men (27.3% and 34.2%, respectively), young individuals aged 18-24 years (35.6% and 41.0%, respectively), white individuals (23.9% and 31.6%, respectively), and individuals with high educational level (38.8% and 49.1%, respectively).^401^

•However, the PENSE has found a reduction in physical activity of Brazilian schoolchildren aged 13-17 years from 2015 to 2019. According to that study, the percentage of physical activity decreased from 31.6% in 2015 to 28.1% in 2019.^402^

•Regarding the adoption of sedentary behaviors, between 2010 and 2020, there was a reduction in the frequency of adults spending at least 3 hours/day of their leisure time watching TV [from 27.3% in 2010 to 26.5% in 2020 (-0.31 pp/year; 95% CI, -0.61;-0.01)].^400^When stratifying by sex, age, and educational level, that reduction was observed only among men (-0.54 pp/year; 95% CI, -0.76;0.32), in the age groups of 18-24 years (-1.49 pp/year; 95% CI, -1.83 to -1.15) and of 25-34 years (-0.57; 95% CI, -1.12;-0.02), and in those with schooling of 9-11 years (-0.43 pp/year; 95% CI, -0.69;-0.18) and ≥12 years (-0.46 pp/year; 95% CI, -0.79;-0.13). That frequency remained stable among women, adults older than 34 years, and those with schooling of 0-8 years.

•Comparing to the global situation, 27.5% of the adults and more than 81% of the adolescents are estimated to not meet the international recommendations for the practice of aerobic physical activity.^395^This suggests that more than 1.4 billion adults are at risk of developing or aggravating diseases related to the lack of physical activity.^403^

### Mortality due to Cardiovascular Diseases Attributable to Insufficient Physical Activity

•According to data from the GBD 2019 on Brazil,^404^regarding the assessment of the mortality rate from CVD attributable to selected risk factors, low level of physical activity, defined as <3000 MET-min/week, ranked eighth as the most important risk factor in 1990 and seventh in 2019. However, there was a reduction in the age-standardized mortality rate from CVD attributable to low level of physical activity, from 26.1 (95% UI, 12.6;41.4) per 100 000 inhabitants in 1990 to 13.7 (95% UI, 7.6;20.8) per 100 000 inhabitants in 2019 ( **Chart 12-4** ). In terms of percent change, in Brazil, that reduction was 47.6% (95% UI, -53.6;-35.0), while, in the different Brazilian states and Distrito Federal, the reductions ranged from 57.1% (75% UI, -63.1;-46.3) in São Paulo to 3.6% (75% UI, -22.9;29.8) in Maranhão ( **Table 12-4 and Chart 12-5** ). When comparing sexes, the age-standardized mortality rate from CVD was similar in men [15.4 (95% UI, 7.9;24.5) per 100 000 inhabitants] and women [12.3 (95% UI, 7.4;17.9) per 100 000 inhabitants], as was the percent reduction from 1990 to 2019 [-42.9% (95% UI, -50.3;-24.9) in men and -50.9% (95% UI, -56.7;-39.7) in women]. When comparing the age groups, there was an increase in the mortality rate attributable to low level of physical activity as age advanced in both 1990 and 2019; however, the percent reduction was similar in the different age groups, and the greatest reduction was observed in the age group of 35-39 years [-55.0% (75% UI, -65.9;-39.6)] and the smallest reduction was observed in the age group of 75-79 years [-41.0% (75% UI, -50.0;-16.8)] ( **Table 12-5** ). **Chart 12-5** shows the percent change in age-standardized mortality rate from 1990 to 2019 in the Brazilian Federative Units.

**Chart 12-4:** – Age-standardized mortality rate (per 100 000 inhabitants) from cardiovascular disease attributable to low level of physical activity in Brazil from 1990 to 2019. Source: Data derived from Global Burden of Disease Collaborative Network. Global Burden of Disease (GBD) Cardiovascular Burden Estimates 1990 and 2021, Institute for Health Metrics and Evaluation, University of Washington. ^48^

**Table 12-4:** – Number of deaths and age-standardized mortality rates (per 100 000 inhabitants) from cardiovascular diseases attributable to low level of physical activity in 1990 and 2019, and percent change of rates, in Brazil and its Federative Units

Location	1990		2019		Percent change (95% UI)
	Number (95% UI)	Rate (95% UI)	Number (95% UI)	Rate (95% UI)	
Brazil	17147.7(7861.7;28644.3)	26.1(12.6;41.4)	30228.8(16769.4;46333.5)	13.7(7.6;20.8)	-47.6%(-53.6;-35)
Acre	21.8(9.7;36.4)	21.9(10.7;34.5)	68.2(38.2;104.4)	14.2(8.3;21.1)	-35.3%(-45.2;-14.9)
Alagoas	251.4(112.3;418.9)	24(11;38.8)	509.9(275.7;799.5)	17(9.1;26.5)	-29.2%(-42.3;-4)
Amapá	12.3(5.6;20.6)	18.6(8.9;29.6)	48.8(25.9;75.6)	12(6.7;18.1)	-35.5%(-44;-19.1)
Amazonas	113.1(55;183.9)	23.5(12;36.4)	297.4(171.5;447.1)	12.2(7.3;18)	-48.2%(-56.2;-31.1)
Bahia	1080.1(499.9;1771.1)	19.4(9.2;31.2)	2055.6(1108.1;3238.8)	12.6(6.8;19.9)	-35.1%(-46.4;-14.4)
Ceará	644.7(309.2;1029.1)	17.3(8.5;27.4)	1548.5(873.3;2352.3)	15.9(9;24.2)	-8%(-26.4;26.1)
Distrito Federal	87.6(37.3;151.9)	34.7(17;54.1)	237.8(126.2;359.2)	15.2(8.6;22.3)	-56.1%(-62.6;-44.1)
Espírito Santo	264(118.2;448.6)	26.9(12.6;43.5)	559.8(290.8;885.6)	14.2(7.5;22.1)	-47.1%(-56;-33.2)
Goiás	332.8(142.5;581.3)	25.1(11.8;40.9)	764.8(394.5;1253.4)	12.6(6.7;20.1)	-49.9%(-60.5;-33.2)
Maranhão	419.2(180.7;739.7)	20.6(9.7;34.8)	1245.5(676;1929.2)	19.9(10.8;30.7)	-3.6%(-22.9;29.8)
Mato Grosso	117.7(51.3;209.9)	23.4(10.9;38.8)	315.6(170.7;506.9)	11.4(6.3;17.8)	-51.4%(-59.5;-35.6)
Mato Grosso do Sul	148.2(64.4;261.4)	25.4(12;41.7)	347.4(185;539.9)	13.3(7.3;20.4)	-47.7%(-55.5;-34.1)
Minas Gerais	1832.8(822.4;3137.1)	27.2(12.7;43.8)	2810.5(1595.3;4299.8)	10.8(6.2;16.5)	-60.3%(-66.2;-49.2)
Pará	363.1(166.7;606)	26(12.7;41.7)	841(467.1;1318)	13.5(7.7;20.9)	-48.1%(-57.2;-31.6)
Paraíba	435.4(214.1;687.8)	20.7(10.4;32.4)	814.9(492.9;1200.5)	16.2(9.7;23.9)	-21.8%(-36.2;4.4)
Paraná	972.1(430.2;1665.5)	30.9(14.5;49.5)	1575(837.5;2538.4)	13.4(7.2;21.1)	-56.5%(-62.6;-45.7)
Pernambuco	923.7(430.7;1521.5)	26.1(12.7;41.7)	1639(877.2;2531.7)	17.6(9.4;27)	-32.4%(-42.9;-16.4)
Piaui	220.5(101;381.8)	22.7(10.6;37.3)	517.8(292;786.7)	13.2(7.4;20.1)	-41.6%(-52.2;-22.1)
Rio de Janeiro	2527.4(1210.1;4104.4)	35.7(17.7;55.1)	3428.7(1923.7;5200.1)	16(9;24)	-55.2%(-61.5;-44.6)
Rio Grande do Norte	249.4(117.3;415.7)	17.4(8.3;28.7)	475.4(262;737.3)	11.8(6.5;18.3)	-32.2%(-45.2;-12.1)
Rio Grande do Sul	1347.6(614.2;2263.3)	28.1(13.6;44.9)	2051.2(1105.1;3132.1)	13.6(7.3;20.6)	-51.7%(-58.2;-39.1)
Rondônia	51.7(20.4;91.5)	37.7(18.2;59.7)	203.7(115.8;310.3)	15.8(9.2;23.7)	-58%(-66.4;-41.1)
Roraima	7.5(3.2;13.3)	27.8(13.2;44.3)	34.4(18.5;54.3)	14.5(8.2;21.7)	-47.8%(-55.6;-30.4)
Santa Catarina	519.7(238.6;858.8)	30.2(14.8;47.1)	956.6(535.1;1478.4)	13.8(7.9;21)	-54.4%(-61.4;-41.4)
São Paulo	4025(1845.2;6915.6)	30.2(14.8;48.3)	6410.1(3530.9;9836.9)	13(7.3;19.7)	-57.1%(-63.1;-46.3)
Sergipe	125.7(56.4;211.7)	23.5(11.1;38.4)	280.1(146.8;447.1)	13.3(7.1;21)	-43.4%(-53.5;-29.3)
Tocantins	53.3(23.2;95.2)	25.8(12.6;41.6)	190.8(100.9;302)	14.9(8;23.1)	-42.2%(-52.4;-25.8)

UI: uncertainty interval. Source: Data derived from Global Burden of Disease Collaborative Network. Global Burden of Disease (GBD) Cardiovascular Burden Estimates 1990 and 2021, Institute for Health Metrics and Evaluation, University of Washington. ^48^

**Chart 12-5:** – Percent change of age-standardized mortality rate from cardiovascular disease attributable to low levels of physical activity from 1990 to 2019, according to the sociodemographic index (SDI) 2019 of the Brazilian Federative Units. Source: Data derived from Global Burden of Disease Collaborative Network. Global Burden of Disease (GBD) Cardiovascular Burden Estimates 1990 and 2021, Institute for Health Metrics and Evaluation, University of Washington. ^48^

**•Chart 12-5** shows that, in the Brazilian Federative Units with the highest SDI (GBD metrics that reflects the socioeconomic conditions of a location and varies from 0 to 1, the best condition being 1), there was a higher reduction in age-standardized mortality rates attributable to low levels of physical activity from 1990 to 2019 (Pearson coefficient of correlation = 0.76, p< 0.001).

•In 1990, 6.4% of all deaths from CVD were attributed to low level of physical activity, but, in 2019, that percentage increased to 7.6%.^404^

•That percentage is similar to the one observed around the world. Globally, it is estimated that 7.6% of the cardiovascular deaths are attributable to physical inactivity, and that percentage is higher in high-income countries (9.9%) as compared to middle- (7.2%) and low- (4.6%) income countries.^405^

### Attributable Burden of Disease

•According to data from the GBD 2019,^404^in both 1990 and 2019, the low level of physical activity ranked ninth as the most important risk factor regarding the age-standardized rate of DALYs due to CVD, which was 434.5 (95% CI, 193.2;747.3) per 100 000 inhabitants in 1990 and 233.1 (95% CI, 118.2;375.7) per 100 000 inhabitants in 2019, representing a percent reduction of 46.4% (95% CI, -84.6;-30.1) in that rate.

•When comparing men and women, the age-standardized DALY rates were similar in 1990 and 2019.^404^Among men, the age-standardized DALY rate decreased from 483.4 (95% CI, 191.0;878.6) per 100 000 inhabitants in 1990 to 275.7 (95% CI, 127.9;478.4) per 100 000 inhabitants in 2019, representing a reduction of 43.0% (95% CI, -49.8;-27.6). Among women, that reduction was 49.1% (95% CI, -54.9;-38.3), from 387.4 (95% CI, 187.6;624.2) per 100 000 inhabitants in 1990 to 197.2 (95% CI, 110.7;303.3) per 100 000 inhabitants in 2019.

•In 2019, the global age-standardized DALY rates due to ischemic heart disease and stroke attributable to low level of physical activity (<3000 MET-min/week) were 96.36 (95% CI, 33.45;210.82) per 100 000 inhabitants and 31.16 (95% CI, 5.69;82.02) per 100 000 inhabitants, respectively.^406^

### Healthcare Utilization and Cost

•Physical inactivity has an important economic impact on health systems. Bielemann *et al* .^407^have carried out a study on the costs attributable to physical inactivity of the hospitalizations at the SUS due to malignant breast and colon neoplasms, circulatory system diseases, diabetes, and osteoporosis of individuals aged ≥40 years, in 2013. Of the total of 974 641 hospitalizations due to the chronic diseases assessed, 15.0% were attributed to physical inactivity. Regarding ischemic heart diseases, the attributable fraction of physical inactivity varied in men from 11.3% in the Southern region to 12.6% in the Northern and Northeastern regions, while, in women, from 16.1% in the Southern region to 18.4% in the Northern region. Regarding cerebrovascular diseases, the attributable fraction of physical inactivity varied in men from 20.7% in the Southern region to 22.8% in the Northern and Northeastern regions, while, in women, from 7.6% in the Southern region to 8.8% in the Northern region.^407^

•Regarding costs to the SUS in 2013, of the total of 1.8 billion *reais* (almost 700 million dollars), over 275 million *reais* were estimated to be due to hospitalizations attributable to physical inactivity from the mentioned diseases.^407^In all regions assessed and in both sexes, ischemic heart diseases accounted for the largest volume of total costs and physical inactivity-attributable costs. Of the total cost of 781 million *reais* with hospitalizations for ischemic heart disease and almost 155 million *reais* for cerebrovascular disease, 12% and 22%, respectively, were attributable to physical inactivity among men. Among women, of the total cost of almost 500 million *reais* with hospitalization for ischemic heart disease and 161 million *reais* for cerebrovascular disease, 17% and 8%, respectively, were attributable to physical inactivity.^407^

•In a retrospective study of 278 residents of the Presidente Prudente city (São Paulo) with CVD, Araújo *et al* .^408^have assessed the direct health-related costs and the indirect costs due to productivity loss and their association with the usual physical activity pattern measured with the Baecke’s questionnaire. Individuals who reported absenteeism spent US$ 187 more on healthcare services annually than those who did not miss work because of health reasons. In addition, it was estimated that US$ 53 could be saved annually at each increase in the score unit of physical activity pattern measured with that questionnaire.

•According to Santos *et al.* ,^409^if the prevalence of physical inactivity does not decrease, a direct physical inactivity-attributable cost to the Brazilian healthcare system between 2020 and 2023 is estimated at approximately US$ 126 million due to coronary artery disease, almost US$ 4 billion due to arterial hypertension, and more than US$ 470 million due to stroke.

### Impact of COVID-19 pandemic on physical activity

•COVID-19 pandemic had a significant impact on the increase of the time spent with sedentary behaviors in Brazil, considerably affecting the practice of physical activity in the entire country and its major geographic regions. According to a study by Silva *et al.* ^410^performed between April/24/2020 and May/24/2020, high prevalence and incidence of physical inactivity and excessive screen time were observed in the Brazilian population. Approximately 40 000 adults filled in an online questionnaire, evidencing a physical inactivity prevalence of 70.4%. This prevalence was even higher among women (74.6%) and younger individuals aged 18-29 years (78.2%).^411^

•In addition, 31.4% of the participants reported spending 4 or more hours daily watching TV, while the use of a computer or tablet for at least 4 hours daily had a prevalence of 37.9%.^410^The frequency of participants reporting TV watching for ≥4 hours/day was higher among women (33.6%), and the frequency of participants reporting a high number of hours spent on a computer/tablet was higher among young individuals aged 18-29 years (58.6%).^411^

•According to data from the VIGITEL on more than 784 000 individuals, the trend toward an increase in the prevalence of leisure-time physical activity was interrupted during the pandemic. In 2009, that prevalence was 29% and increased to 39% in 2019. However, that prevalence decreased to 36.8% in 2020 and to 36.7% in 2021. Among men, there was a reduction of 3.6%, while, among women, the reduction was 1.1% from 2019 to 2021.^412^

•According to Schuch *et al.* ,^413^Brazilians on self-isolation during the COVID-19 pandemic reduced the time dedicated to moderate/vigorous physical activity by 65 min/day as compared to the prepandemic period. However, the time spent in sedentary behaviors increased by 42%, corresponding to an increase of 152 min/day.

### Future Directions

•Physical inactivity is one of the major modifiable risk factors for noncommunicable diseases, such as stroke, arterial hypertension, type 2 diabetes, coronary artery disease, several types of cancer, dementia, depression, as well as for all-cause mortality.^414^A physically-active lifestyle plays an unequivocal role in achieving important cardiovascular benefits in both primary and secondary prevention.^415^The linear relationship between the volumes of physical activity and cardiovascular protection^416^determines that the simple fact of quitting a sedentary lifestyle and becoming physically active significantly reduces cardiovascular risk. Thus, sedentary behavior should be discouraged, and adoption of a physically active lifestyle, encouraged.

•Aiming to encourage the practice of physical activity, national actions have been implemented, such as the City Gym Program and the National Policy for Health Promotion, contributing to increase physical activity practice, with a positive impact on health, and encouraging the improvement of public spaces intended for that practice.^417^In addition, the inclusion of physical activity in Brazilian population surveys, such as VIGITEL, PNS, and PENSE, has enhanced physical activity monitoring among Brazilians, helping review and develop public health policies.

•However, despite the trend to reduce physical inactivity among Brazilians in recent years, its prevalence remains high, and that was compounded by the COVID-19 pandemic. Thus, it is necessary to continuously encourage national policies that promote healthy life habits, such as the increase in regular physical activity time, aiming to reduce the deleterious socioeconomic consequences of physical inactivity and sedentary lifestyle in Brazil.

**Table 7-1:** – Prevalence and 95% confidence interval (95% CI) of hypertension according to sociodemographic characteristics. National Health Survey, Brazil, 2019

Variables	Total		Female		Male	
	Prevalence (95% CI)	PR	Prevalence (95% CI)	PR	Prevalence (95% CI)	PR
**Total**	23.9 (23.5;24.4)		26.4 (25.7;27.2)		21.1 (20.4;21.8)	
**Age group (years)**						
18-24	2.3 (1.7;2.9)	1.0	2.1 (1.5;2.8)	1.0	2.5 (1.6;3.4)	1.0
25-39	7.3 (6.7;7.8)	3.2	7.2 (6.3;8.0)	3.5	7.4 (6.6;8.1)	3.0
40-59	27.2 (26.3;28.1)	11.5	29.5 (28.2;30.7)	13.3	24.6 (23.3;25.9)	9.9
≥ 60	55.0 (53.9;56.1)	22.1	59.4 (57.9;60.8)	24.7	49.3 (47.6;50.9)	19.7
**Educational level**						
No education/ incomplete elementary	36.6 (35.7;37.5)	1.0	43.3 (42.0;44.6)	1.0	29.2 (28.0;30.3)	1.0
Complete elementary / incomplete middle	20.4 (19.1;21.6)	1.0	24.7 (22.8;26.6)	0.9	16.2 (14.6;17.8)	1.0
Complete middle/ incomplete high	15.4 (14.7;16.2)	0.8	15.7 (14.7;16.7)	0.8	15.1 (14.0;16.2)	1.0
Complete high	18.2 (17.1;19.3)	0.7	16.3 (15.0;17.7)	0.6	20.7 (18.8;22.6)	1.0
**Self-reported race/skin color**						
White	24.4 (23.6;25.2)	1.0	26.0 (24.9;27.1)	1.0	22.5 (21.4;23.5)	1.0
Black	25.8 (24.4;27.2)	1.2	30.2 (28.2;32.2)	1.2	20.9 (19.1;22.7)	1.1
Brown	22.9 (22.2;23.6)	1.1	25.7 (24.8;26.7)	1.1	19.7 (18.8;20.6)	1.0

PR: Prevalence Ratio. Source: Brazilian Institute of Geography and Statistics – National Health Survey 2019 ^272^ and Malta et al. ^263^

**Table 7-2:** – Percentage distribution and 95% confidence intervals (95% CI) of care-related characteristics of people with self-reported hypertension (n = 38 082), National Health Survey, Brazil, 2019

Variables	%	95% CI
**Last healthcare visit**		
Less than 6 months	57.8	56.6;59.0
6 months – less than 1 year	14.4	13.6;15.2
1 year – less than 2 years	9.4	8.8;10.1
2 years – less than 3 years	2.7	2.3;3.2
3 years or more	13.7	12.9;14.5
Never	1.9	1.6;2.3
**Last healthcare visit place**		
Basic Healthcare Unit	45.8	44.4;47.2
Private office	28.8	27.5;30.1
Emergency service	9.6	8.9;10.5
Public outpatient clinic	7.1	6.5;7.8
Public polyclinic	3.6	3.1;4.2
Private emergency service	1.7	1.4;2.0
Home	1.4	1.2;1.6
Drugstore	0.9	0.7;1.2
Other service	1.0	0.7;1.4
**Instructions**		
Integrative practices	7.4	6.8;8.1
No excessive drinking	66.5	65.1;67.8
No smoking	67.2	65.8;68.6
Regular physical activity	81.7	80.7;82.7
Maintain proper weight	84.4	83.4;85.4
Regular follow-up	85.2	84.2;86.2
Healthy diet	87.2	86.3;88.2
Less salt diet	87.8	86.7;88.8
Tests and referrals		
**Blood test**	79.9	78.8;80.9
Urine test	69.9	68.5;71.2
Electrocardiogram	64.5	63.2;65.8
Exercise test	33.6	32.2;34.9
Referral to a specialist	25.0	23.7;26.2

Source: Brazilian Institute of Geography and Statistics – National Health Survey 2019 ^272^ and Malta et al. ^263^

**Table 7-3:** – Healthcare indicators and access to health services of adults with arterial hypertension according to gender. National Health Survey, Brazil, 2019

Indicators		Total				Sex		
**%**	**95%CI**	**Male (A)**		**Female (B)**		**Adjusted PR (B/A)**	**95%CI**
		**%**	**95%CI**	**%**	**95%CI**		
Prescribed medication	95.4	94.9; 95.8	938	92.9; 94.5	96.5	95.9; 97.0	1.02	1.01; 1.03
Use of medication in the previous two weeks	86.9	86.1; 87.7	831	81.7; 84.5	89.6	88.6; 90.5	1.06	1.04; 1.08
At least one medication from the “We Have a Popular Pharmacy Here” program	45.1	43.6; 46.5	437	41.7; 45.7	46.0	44.3; 47.7	1.03	0.98; 1.08
Received medical care for hypertension within the last year	72.2	71.1; 73.3	68.9	67.3; 70.5	74.5	73.1; 75.8	1.07	1.04; 1.11
Had the last physician appointment at an UBS	45.8	44.4; 47.2	41.8	39.7; 43.9	48.5	46.9; 50.2	1.11	1.05; 1.17
Had an appointment with the same physician as in the previous appointments	51.9	50.6; 53.3	52.3	50.3; 54.3	51.7	49.8; 53.5	0.99	0.94; 1.05
Appointments with a specialist	49.0	47.6; 50.4	49.5	47.4; 51.5	48.6	46.8; 50.5	1.01	0.96; 1.07
Referral for a specialist	79.1	76.8; 81.1	78.6	75.0; 81.8	79.3	76.5; 82.0	1.01	0.96; 1.06
Hospitalization for hypertension or a complication	15.0	14.2; 15.9	13.6	12.4; 15.0	15.9	14.8; 17.1	1.14	1.01; 1.28
Intense or very intense degree of limitation of daily living activities	3.0	2.7; 3.4	3.1	2.5; 3.7	3.0	2.6; 3.5	0.93	0.73; 1.17

CI: Confidence Interval; PR: Prevalence Ratio; UBS: Basic Healthcare Unit. The reference category used was ‘male’. Source: Malta et al. ^261^

**Table 7-4:** – Healthcare indicators of adults with arterial hypertension according to age group. National Health Survey, Brazil, 2019

Indicator					Age Group (years)					
	18-29 (A)		30-59 (B)		≥ 60 (C)		Adjusted PR B/A	95%CI	Adjusted PR (C/A)	95%CI
	%	95%CI	%	95%CI	%	95%CI				
Prescribed medication	68.4	61.6; 74.5	93.3	92.4; 94.1	98.8	98.4; 99.0	1.35	1.23; 1.49	1.43	1.30; 1.57
Use of medication in the previous two weeks	39.3	31.9; 47.2	81.9	80.6; 83.2	94.3	93.6; 94.9	2.05	1.69; 2.49	2.34	1.93; 2.85
At least one medication from the “Popular Pharmacy Program”	21.1	14.0; 30.6	45.7	43.3; 48.0	45.1	43.5; 46.7	2.16	1.46; 3.20	2.08	1.41; 3.08
Received medical care for hypertension within the last year	62.9	54.9; 70.3	71.1	69.5; 72.8	73.7	72.3; 75.0	1.11	0.98; 1.25	1.13	1.00; 1.27
Had the last physician appointment at UBS	47.1	38.6; 55.7	45.5	43.6; 47.4	46.0	44.3; 47.8	0.95	0.80; 1.14	0.92	0.76; 1.10
Had an appointment with the same physician as in the previous appointments	37.6	29.4; 46.6	49.6	47.6; 51.5	54.9	53.2; 56.7	1.29	1.02; 1.62	1.42	1.13; 1.79
Appointments with a specialist	64.2	48.3; 77.8	75.4	71.7; 78.8	83.4	80.6; 85.9	1.17	0.93; 1.47	1.31	1.04; 1.64
Referral for a specialist	35.8	28.0; 44.5	46.4	44.3; 48.5	52.1	50.4; 53.9	1.27	1.02; 1.58	1.47	1.18; 1.83
Hospitalization for hypertension or a complication	13.0	8.3; 19.9	14.1	12.9; 15.5	16.0	14.8; 17.2	1.02	0.65; 1.58	1.07	0.69; 1.68
Intense or very intense degree of limitation of daily living activities	2.9	1.2; 6.8	3.1	2.6; 3.8	2.9	2.5; 3.4	0.92	0.37; 2.26	0.70	0.29; 1.72

CI: Confidence Interval; PR: Prevalence Ratio; UBS: Basic Healthcare Unit. The reference category used was the ‘age group of 18-29 years’. Source: Malta et al. ^261^

**Table 7-5:** – Healthcare indicators of adults with arterial hypertension according to ethnicity. National Health Survey, Brazil, 2019

Indicator	Ethnicity									
	White (A)		Black (B)		Mixed-race (C)		Adjusted PR (B/A)	95%CI	Adjusted PR (C/A)	95%CI
	%	95%CI	%	95%CI	%	95%CI				
Prescribed medication	96.2	95.5; 96.8	95.0	93.5; 96.2	94.5	93.7; 95.2	1.00	0.98; 1.01	1.00	0.98; 1.01
Use of medication in the previous two weeks	88.5	87.2; 89.6	86.7	84.6; 88.6	85.3	84.1; 86.5	0.99	0.97; 1.01	1.00	0.98; 1.03
At least one medication from the “Popular Pharmacy Program”	44.8	42.9; 46.8	46.3	43.0; 49.7	44.8	42.8; 46.9	0.94	0.89; 1.00	0.97	0.89; 1.06
Received medical care for hypertension within the last year	71.5	69.9; 73.1	74.7	71.9; 77.3	72.1	70.5; 73.7	1.01	0.98; 1.04	1.05	1.01; 1.09
Had the last physician appointment at UBS	39.4	37.3; 41.6	51.9	48.6; 55.2	50.7	48.9; 52.5	1.01	0.96; 1.07	1.06	0.98; 1.14
Had an appointment with the same physician as in the previous appointments	56.7	54.6; 58.7	48.8	45.4; 52.2	48.2	46.3; 50.2	0.92	0.87; 0.97	0.93	0.85; 1.00
Appointments with a specialist	54.6	52.5; 56.6	47.9	44.6; 51.3	43.4	41.5; 45.3	0.96	0.91; 1.01	0.94	0.87; 1.02
Referral for a specialist	82.9	79.7; 85.7	75.4	68.9; 80.9	76.7	73.3; 79.7	0.92	0.88; 0.97	1.02	0.94; 1.09
Hospitalization for hypertension or a complication	13.2	11.9; 14.6	15.7	13.5; 18.2	16.8	15.4; 18.3	1.20	1.05; 1.38	1.14	0.95; 1.36
Intense or very intense degree of limitation of daily living activities	2.2	1.8; 2.7	3.5	2.7; 4.6	3.6	3.1; 4.3	1.37	1.06; 1.76	1.38	0.99; 1.94

CI: Confidence Interval; PR: Prevalence Ratio; UBS: Basic Healthcare Unit. The reference category used was ‘white’. Source: Malta et al. ^261^

**Table 8-2:** – Prevalence of total cholesterol ≥ 200 mg/dL according to sex, age group, educational level, skin color, and country region. Brazil, PNS 2014-2015

	Total				Men			Women		
	%	95% CI	p		%	95% CI	p	%	95% CI	p
Total	32.7	31.5 - 34.1			30.1	28.2 - 32.1		35.1	33.4 - 36.8	< 0.001
Age group (years)										
18 - 29	17.9	15.7 - 20.4		< 0.001	13.9	11.2 - 17.4	< 0.001	21.9	18.7 - 25.5	< 0.001
30 - 44	31.0	28.7 - 33.4			34.9	31.2 - 38.8		27.6	24.9 - 30.5	
45 - 59	43.4	40.8 - 46.0			39.4	35.7 - 43.4		47.0	43.5 - 50.5	
≥ 60	41.9	39.1 - 44.8			33.5	29.5 - 37.9		48.4	44.7 - 52.2	
Educational level (school years)										
0 - 8	37.1	35.2 - 39.1		< 0.001	31.6	28.9 - 34.5	0.237	42.2	39.6 - 44.8	< 0.001
9 - 11	28.6	25.5 - 32.0			26.6	22.2 - 31.6		30.6	26.4 - 35.2	
≥ 12	30.4	28.4 - 32.5			30.0	26.9 - 33.3		30.8	28.3 - 33.4	
**Skin color**										
White	33.9	31.9 - 36.0		0.146	30.8	27.8 - 33.9	0.669	36.6	33.9 - 39.4	0.196
Black	33.2	29.0 - 37.6			30.0	23.9 - 37.0		36.0	30.5 - 41.8	
Mixed	31.5	29.8 - 33.3			29.5	26.9 - 32.4		33.4	31.1 - 35.7	
Others	23.3	14.8 - 34.6			19.6	9.7 - 35.4		25.8	14.2 - 42.2	
**Region**										
North	32.5	30.4 - 34.6		0.195	31.0	27.9 - 34.3	0.376	33.9	31.2 - 36.7	0.291
Northeast	34.0	32.3 - 35.8			30.2	27.7 - 33.0		37.4	35.1 - 39.8	
Southeast	31.5	29.1 - 34.1			28.7	25.1 - 32.6		34.1	30.9 - 37.4	
South	34.7	31.7 - 37.8			33.4	28.9 - 38.3		35.8	32.0 - 39.8	
West-Central	31.7	28.7 - 34.8			30.1	25.7 - 34.9		33.0	29.1 - 37.2	

Source: Malta et al. 2019. ^275^

**Table 8-3:** – Prevalence of low HDLc (< 40 mg/dL) according to sex, age group, educational level, skin color, and Brazilian region, PNS 2014-2015

	Total			Men			Women		
	%	95% CI	P	%	95% CI	p	%	95% CI	p
**Total**	31.8	30.5 - 33.1		42.8	40.6 - 45.0		22.0	20.6 - 23.5	< 0.001
**Age group (years)**									
18 - 29	29.1	26.2 - 32.2	0.070	39.7	34.9 - 44.7	0.159	18.7	15.9 - 21.9	0.046
30 - 44	31.8	29.4 - 34.2		41.8	37.9 - 45.7		23.0	20.4 - 25.9	
45 - 59	34.1	31.6 - 36.6		44.8	40.9 - 48.8		24.3	21.5 - 27.4	
≥ 60	32.4	29.8 - 35.2		46.5	42.1 - 51.1		21.5	18.7 - 24.6	
**Educational level (school years)**									
0 - 8	33.7	31.8 - 35.7	< 0.001	43.3	40.2 - 46.4	0.006	24.9	22.8 - 27.2	< 0.001
9 - 11	38.5	34.9 - 42.2		50.0	44.3 - 55.6		27.0	22.9 - 31.5	
≥ 12	27.8	25.9 - 29.9		39.6	36.2 - 43.2		18.1	16.1 - 20.3	
**Skin color**									
White	31.0	29.0 - 33.1	0.072	43.0	39.7 - 46.5	0.586	20.6	18.4 - 23.0	0.006
Black	28.5	24.3 - 33.2		41.8	34.5 - 49.4		16.6	12.6 - 21.6	
Mixed	33.5	31.7 - 35.4		43.0	40.0 - 46.1		24.8	22.8 - 27.0	
Others	24.7	15.8 - 36.5		27.7	15.1 - 45.2		22.7	11.6 - 39.5	
**Country region**									
North	36.6	34.4 - 38.8	< 0.001	47.2	43.7 - 50.7	0.036	26.7	24.2 - 29.4	< 0.001
Northeast	34.8	33.0 - 36.6		44.3	41.4 - 47.2		26.4	24.3 - 28.6	
Southeast	30.8	28.3 - 33.4		43.1	38.9 - 47.3		20.0	17.4 - 22.9	
South	26.1	23.3 - 29.0		36.3	31.6 - 41.2		16.8	14.1 - 20.0	
West-Central	34.3	31.1 - 37.6		45.0	39.8 - 50.3		24.7	21.2 - 28.6	

HDLc: high-density-lipoprotein cholesterol. Source: Malta et al. 2019.^*275*^

**Table 8-4:** – Prevalence of high LDLc (> 130 mg/dL) according to sex, age group, educational level, skin color, and Brazilian region, PNS 2014-2015

	Total			Men			Women		
	%	95% CI	p	%	95% CI	p	%	95% CI	p
**Total**	18.6	17.5 - 19.7		17.1	15.6 - 18.8		19.9	18.5 - 21.3	0.012
**Age group (years)**									
18 - 29	8.8	7.2 - 10.7	< 0.001	6.6	4.8 - 9.0	< 0.001	11.0	8.7 - 14.0	< 0.001
30 - 44	17.5	15.7 - 19.5		20.2	17.3 - 23.6		15.2	13.0 - 17.6	
45 - 59	25.6	23.3 - 27.9		23.2	20.0 - 26.7		27.7	24.7 - 30.9	
≥ 60	24.5	22.2 - 27.0		19.5	16.3 - 23.2		28.4	25.1 - 31.9	
**Educational level (school years)**									
0 - 8	21.5	20.0 - 23.2	< 0.001	17.8	15.7 - 20.1	0.525	24.9	22.8 - 27.2	< 0.001
9 - 11	16.8	14.3 - 19.7		15.2	11.8 - 19.3		18.5	15.0 - 22.6	
≥ 12	16.7	15.1 - 18.4		17.2	14.8 - 20.0		16.2	14.2 - 18.4	
**Skin color**									
White	20.1	18.5 - 21.9	0.009	18.8	16.4 - 21.4	0.131	21.3	19.1 - 23.8	0.095
Black	16.6	13.6 - 20.2		15.2	10.9 - 20.8		17.9	13.9 - 22.7	
Mixed	17.4	16.1 - 18.8		15.9	13.9 - 18.1		18.8	17.0 - 20.7	
Others	10.1	6.0 - 16.6		8.6	3.6 - 19.1		11.2	5.7 - 20.7	
**Country region**									
North	16.2	14.7 - 17.9	0.136	15.5	13.2 - 18.1	0.355	17.0	14.9 - 19.2	0.195
Northeast	19.8	18.4 - 21.3		17.5	15.5 - 19.8		21.9	19.9 - 23.9	
Southeast	17.9	16.0 - 19.9		16.1	13.4 - 19.3		19.4	16.8 - 22.2	
South	20.0	17.6 - 22.6		19.8	16.2 - 24.0		20.1	17.1 - 23.5	
West-Central	17.8	15.4 - 20.4		17.8	14.3 - 21.9		17.8	14.8 - 21.3	

LDLc: low-density-lipoprotein cholesterol. Source: Malta et al. 2019. ^275^

**Chart 10-1:** – Prevalence of adult current smokers (≥ 18 years) according to the Brazilian Federative Units and prevalence quartiles. Data from the VIGITEL Brazil 2021. ^252^

**Chart 10-2:** – Prevalence of adult current smokers (≥ 18 years) according to the Brazilian state capitals and Distrito Federal, by sex. Data from the VIGITEL Brazil 2021. ^252^

**Table 11-1:** – Percentage of overweight adults (≥ 18 years), by sex, in Brazilian capitals and Distrito Federal, by age group

Age group	Male		Female		Total	
	%	95% CI	%	95% CI	%	95% CI
18 - 24 years	39.3	33.6 - 45.1	31.7	25.5 - 37.9	35.7	31.5 - 40.0
25 - 34 years	63.4	57.7 - 69.1	46.6	41.1 - 52.0	54.4	50.3 - 58.5
35 - 44 years	62.9	57.3 - 68.6	61.9	57.7 - 66.2	62.4	59.0 - 65.8
45 - 54 years	67.2	62.6 - 71.9	61.9	58.5 - 65.3	64.4	61.5 - 67.2
55 - 64 years	61.9	57.2 - 66.7	65.7	62.7 - 68.6	64.1	61.4 - 66.7
60 + years	60.7	57.2 - 64.2	60.7	58.6 - 62.9	60.7	58.8 - 62.6

Source: VIGITEL Brazil 2021. ^252^

**Table 11-2:** – Percentage of obese adults (≥ 18 years), by sex, in Brazilian capitals and Distrito Federal, by age group

Age group	Male		Female		Total	
	%	95% CI	%	95% CI	%	95% CI
18 - 24 years	13.1	9.3 - 17.0	11.2	6.4 - 16.0	12.2	9.2 - 15.3
25 - 34 years	25.5	19.9 - 31.1	16.6	12.8 - 20.4	20.8	17.4 - 24.1
35 - 44 years	25.1	20.7 - 29.6	25.7	21.9 - 29.5	25.5	22.5 - 28.4
45 - 54 years	24.3	20.3 - 28.3	27.9	24.7 - 31.2	26.2	23.7 - 28.8
55 - 64 years	22.1	18.3 - 26.0	29.3	26.2 - 32.5	26.2	23.8 - 28.7
60 + years	16.8	14.1 - 19.5	25.3	23.4 - 27.2	21.8	20.2 - 23.4

Source: VIGITEL Brazil 2021. ^252^

**Table 11-3:** – Percentage of overweight adults (≥ 18 years), by sex, according to the Brazilian capitals and Distrito Federal

	Male		Female		Total	
Capitals	%	95% CI	%	95% CI	%	95% CI
Aracaju	60.9	52.0 - 69.9	54.4	48.8 - 60.1	57.3	52.3 - 62.4
Belém	61.6	54.3 - 68.9	61.0	55.6 - 66.4	61.3	56.8 - 65.7
Belo Horizonte	58.4	51.1 - 65.7	58.7	53.3 - 64.1	58.6	54.1 - 63.0
Boa Vista	58.7	51.9 - 65.5	54.2	49.3 - 59.2	56.4	52.2 - 60.5
Campo Grande	61.3	51.8 - 70.7	55.4	48.6 - 62.1	58.1	52.4 - 63.8
Cuiabá	58.5	50.2 - 66.8	55.7	49.2 - 62.2	57.0	51.8 - 62.3
Curitiba	62.8	55.7 - 69.9	48.9	42.7 - 55.0	55.3	50.6 - 60.1
Florianópolis	59.5	51.6 - 67.4	54.5	48.3 - 60.8	56.9	51.9 - 61.9
Fortaleza	61.2	53.4 - 68.9	57.5	51.5 - 63.6	59.2	54.4 - 64.0
Goiânia	62.1	54.4 - 69.9	51.2	45.2 - 57.2	56.3	51.4 - 61.2
João Pessoa	66.5	58.6 - 74.3	53.9	47.6 - 60.2	59.6	54.6 - 64.6
Macapá	62.3	55.9 - 68.7	55.5	50.1 - 60.9	58.7	54.6 - 62.9
Maceió	59.2	51.3 - 67.2	57.2	50.4 - 64.0	58.1	53.0 - 63.2
Manaus	65.2	57.3 - 73.1	61.8	56.3 - 67.4	63.5	58.7 - 68.2
Natal	60.9	52.7 - 69.1	57.5	51.7 - 63.3	59.1	54.2 - 64.0
Palmas	55.9	47.8 - 64.0	45.0	39.4 - 50.6	50.1	45.2 - 55.0
Porto Alegre	64.4	57.0 - 71.8	60.4	52.9 - 67.8	62.2	56.9 - 67.4
Porto Velho	67.5	60.3 - 74.7	61.0	55.5 - 66.6	64.4	59.8 - 69.0
Recife	58.1	49.1 - 67.1	55.6	49.5 - 61.7	56.7	51.5 - 61.9
Rio Branco	63.2	55.6 - 70.8	57.8	52.0 - 63.6	60.4	55.7 - 65.0
Rio de Janeiro	64.0	56.3 - 71.7	49.4	43.1 - 55.8	56.1	51.0 - 61.2
Salvador	50.8	43.0 - 58.7	55.1	49.5 - 60.8	53.2	48.5 - 57.9
São Luís	51.4	43.2 - 59.7	47.5	41.8 - 53.2	49.3	44.4 - 54.2
São Paulo	57.6	50.7 - 64.5	57.3	51.0 - 63.5	57.4	52.8 - 62.0
Teresina	60.0	52.1 - 67.9	46.4	40.4 - 52.3	52.5	47.5 - 57.5
Vitória	55.8	48.1 - 63.6	47.8	41.9 - 53.7	51.5	46.7 - 56.3
Distrito Federal	59.4	51.7 - 67.2	53.7	47.5 - 59.8	56.4	51.5 - 61.2

Source: VIGITEL Brazil 2021. ^252^

**Table 11-4:** – Percentage of adults (≥ 18 years) with obesity, by sex, according to Brazilian state capitals and the Federal District

	Male		Female		Total	
Capitals	%	95% CI	%	95% CI	%	95% CI
Aracaju	27.9	19.2 - 36.5	22.6	18.4 - 26.9	25.0	20.4 - 29.5
Belém	23.8	17.1 - 30.4	24.2	19.2 - 29.1	24.0	19.9 - 28.0
Belo Horizonte	20.7	14,3 - 27,0	22.0	17,3 - 26,6	21.4	17,5 - 25,2
Boa Vista	25.0	19,1 - 30,8	22.3	18,3 - 26,3	23.6	20,1 - 27,1
Campo Grande	19.4	12,4 - 26,3	21.0	16,1 - 25,9	20.2	16,1 - 24,4
Cuiabá	22.6	15,3 - 29,9	25.0	19,8 - 30,1	23.8	19,4 - 28,2
Curitiba	24.3	17,6 - 30,9	21.1	16,3 - 26,0	22.6	18,6 - 26,6
Florianópolis	21.4	15,0 - 27,9	19.2	14,6 - 23,7	20.2	16,4 - 24,1
Fortaleza	23.2	16,5 - 30.0	24.1	19.1 - 29.1	23.7	19.6 - 27.8
Goiânia	26.7	19.4 - 34.0	20.3	15.7 - 24.8	23.3	19.0 - 27.5
João Pessoa	22.0	15.3 - 28.8	25.1	19.4 - 30.9	23.7	19.3 - 28.1
Macapá	23.7	18.4 - 29.0	23.7	19.2 - 28.2	23.7	20.3 - 27.1
Maceió	23.7	16.2 - 31.2	25.4	18.8 - 31.9	24.6	19.7 - 29.5
Manaus	23.3	16.7 - 29.9	26.6	21.4 - 31.7	25.0	20.8 - 29.1
Natal	23.0	16.3 - 29.8	23.1	17.7 - 28.4	23.0	18.8 - 27.3
Palmas	23.3	14.9 - 31.6	16.1	12.2 - 19.9	19.5	15.0 - 24.0
Porto Alegre	22.9	16.0 - 29.8	22.4	16.4 - 28.3	22.6	18.1 - 27.1
Porto Velho	26.6	19.8 - 33.4	26.2	21.1 - 31.3	26.4	22.1 - 30.7
Recife	17.7	11.5 - 23.9	26.5	20.7 - 32.4	22.6	18.3 - 26.9
Rio Branco	25.0	18.7 - 31.3	23.4	18.7 - 28.0	24.2	20.3 - 28.0
Rio de Janeiro	23.1	15.8 - 30.4	20.1	15.7 - 24.5	21.5	17.4 - 25.6
Salvador	18.6	12.8 - 24.3	22.0	17.7 - 26.3	20.5	17.0 - 23.9
São Luís	18.6	11.1 - 26.0	17.6	13.3 - 22.0	18.0	13.9 - 22.2
São Paulo	20.6	14.8 - 26.3	24.2	19.2 - 29.1	22.5	18.8 - 26.3
Teresina	24.1	16.9 - 31.3	17.2	12.9 - 21.5	20.3	16.3 - 24.3
Vitória	19.2	13.0 - 25.4	16.8	12.9 - 20.6	17.9	14.4 - 21.4
Distrito Federal	23.0	16.6 - 29.4	22.2	17.2 - 27.3	22.6	18.6 - 26.6

Source: VIGITEL Brazil 2021. ^252^

**Chart 11-3:** – Percentage of overweight adults (≥ 18 years), Brazil, 2006-2021. Data from the VIGITEL Brazil 2021. ^252^

**Chart 11-4:** – Percentage of adults with obesity (≥ 18 years), Brazil, 2006-2021. Data from the VIGITEL Brazil 2021. ^252^

**Table 12-5:** – Number of deaths and specific age-standardized mortality rates from cardiovascular diseases attributable to low level of physical activity, in 1990 and 2019, per age group, and percent change of rates, in Brazil

Location	1990		2019		Percent change (95% UI)
	Number (95% UI)	Rate (95% UI)	Number (95% UI)	Rate (95% UI)	
25-29 years	70.1(15.4;160)	0.5(0.1;1.2)	49.6(9.1;114.2)	0.3(0.1;0.7)	-46.9%(-62.9;-32.5)
30-34 years	144.2(29.7;331)	1.3(0.3;3)	106(18.8;243.1)	0.6(0.1;1.4)	-53.4%(-65.5;-36.5)
35-39 years	255.9(53.8;581.2)	2.7(0.6;6.1)	213(39.8;479)	1.2(0.2;2.7)	-55%(-65.9;-39.6)
40-44 years	363.9(81.8;823.1)	4.7(1.1;10.6)	340.6(68.9;754.6)	2.2(0.4;4.8)	-53.8%(-65.6;-40.3)
45-49 years	572.8(147;1240.9)	9.3(2.4;20.2)	595.2(135.9;1311.9)	4.4(1;9.6)	-53.3%(-64.2;-39.8)
50-54 years	794.9(214.2;1689.7)	15.4(4.1;32.7)	919.5(246.8;1976.8)	7.3(1.9;15.6)	-52.9%(-62.5;-38.1)
55-59 years	960.9(285.4;1991.3)	22.5(6.7;46.6)	1269.9(367.5;2625.9)	11.3(3.3;23.4)	-49.7%(-59.1;-35.1)
60-64 years	1380.4(441.6;2616.1)	38.6(12.4;73.2)	1871.2(624.5;3451.5)	20.2(6.8;37.3)	-47.6%(-56.7;-32.5)
65-69 years	1633.8(586;3029.7)	61(21.9;113.2)	2468.2(965.3;4160.9)	34.3(13.4;57.8)	-43.8%(-52.4;-23.8)
70-74 years	2083.2(884.8;3490.5)	110.1(46.8;184.5)	3348(1785;5150.2)	63.5(33.8;97.7)	-42.3%(-51;-19.9)
75-79 years	2206.7(997.2;3495.6)	172.6(78;273.3)	3636.4(2179.2;5231.9)	101.9(61.1;146.6)	-41%(-50;-16.8)
80+ years	6680.9(3508.8;10243.7)	630.9(331.4;967.4)	15411.3(9112.4;22314.1)	363(214.7;525.6)	-42.5%(-49.8;-26.4)

UI: uncertainty interval **.** Source: Data derived from Global Burden of Disease Collaborative Network. Global Burden of Disease (GBD) Cardiovascular Burden Estimates 1990 and 2021, Institute for Health Metrics and Evaluation, University of Washington. ^48^

**Chart 12-2:** – Frequency of adults aged ≥18 years who spent ≥3 hours daily of their leisure time watching television or using a computer, tablet, or cellular phone, in the Distrito Federal and capitals of the Brazilian states according to data from VIGITEL 2021. Data from the VIGITEL Brazil 2021. ^252^
